# Electronic structure of two isostructural ‘paddle-wheel’ complexes: a comparative study

**DOI:** 10.1107/S2052520618013707

**Published:** 2018-11-19

**Authors:** Peter Herich, Lukáš Bučinský, Martin Breza, Marián Gall, Marek Fronc, Václav Petřiček, Jozef Kožíšek

**Affiliations:** a Institute of Physical Chemistry and Chemical Physics, Slovak University of Technology in Bratislava, Radlinského 9, Bratislava, SK-81237, Slovakia; b Institute of Information Engineering, Automation and Mathematics, Slovak University of Technology in Bratislava, Radlinského 9, Bratislava, SK-81237, Slovakia; c Institute of Physics, Czech Academy of Sciences, Na Slovance 1999/2, 182 21 Prague 8, Czech Republic

**Keywords:** electronic structure, charge density, valence-shell electron-pair repulsion (VSEPR), quantum crystallography, classical coordination bond

## Abstract

The experimental electron density distribution in two isostructural and isomorphous complexes, tetra­kis(μ_2_-acetato)di­aquadicopper(II) [H_2_OCu(ac)_2_Cu(ac)_2_H_2_O] (I) and tetra­kis(μ_2_-acetato)diaquadichromium(II), [H_2_OCr(ac)_2_Cr(ac)_2_H_2_O] (II), has been obtained from high-resolution X-ray diffraction data in order to shed light on the bonding properties in the compounds studied. It has been shown that from accurate X-ray data it is possible to discuss the bonding capability of the metal atom (Cu/Cr) and the ligands in these complexes. A comparison of results obtained from averaged and non-averaged X-ray data demonstrates that using the non-averaged data and introducing an anisotropic correction for secondary extinction errors provides a more detailed distribution of the electron density in the area of the metal atoms. In both complexes studied, the bonding of the acetate oxygen atom to the central metal atom is significantly affected by the formation of hydrogen bonds. The electron density and its Laplacian at the bond critical point of metal–oxygen coordination bonds for those oxygen atoms not involved in the intermolecular hydrogen bonds are approximately 10% larger compared with the case when oxygen atoms take part in hydrogen bonds with the neighboring water molecules. It is shown that metal–oxygen bonds in a quasi-equatorial plane are typical coordination bonds and differ significantly from the apical metal–oxygen bond. Metal–metal inter­action with a small positive value of the electron density Laplacian at this bond critical point is mainly of electrostatic character. The metal–metal inter­action is definitely not a bond according to the classical definition. Based on a search of the Cambridge Structural Database, it can be argued that there are four typical coordination bonds in the [CuO_6_] chromophore, similar to the four Cu—O coordination bonds in the basal plane of the CuO_5_ pyramid in one of the complexes under study.

## Abstract 2

The experimental electron-density distribution in two isostructural complexes, tetra­kis(μ_2_-acetato)di­aquadicopper(II) [H_2_OCu(ac)_2_Cu(ac)_2_H_2_O] and tetra­kis(μ_2_-acetato)di­aquadichromium(II) [H_2_OCr(ac)_2_Cr(ac)_2_H_2_O], has been studied and compared.

## 1. Introduction   

It was necessary to have a good deal of imagination when Ronald James Gillespie and Ronald Sydney Nyholm developed the Valence-Shell Electron-Pair Repulsion (VSEPR) concept (Jolly, 1984 ▸). Nowadays, using experimental charge density studies, we are able to verify our findings against this concept and even to go beyond (Gillespie, 2008 ▸). Bader’s AIM (Atoms-in-Molecule) theory (Bader, 1990 ▸) gives us some individual quantifiers of electron density distribution and so we can compare the particular chemical bonds to one another. Charge density studies of small molecules allowed us to characterize, both qualitatively and quantitatively, various intra- and intermolecular interactions existing in the crystal structure of the compound studied (Gatti & Macchi, 2012 ▸; Coppens, 2013 ▸; Zhurov & Pinkerton, 2015 ▸; Cenedese *et al.*, 2015 ▸). Comparison of experimental and theoretical electronic structures gives, in principle, a good agreement and the discrepancies found could help to identify the systematic errors and improve both treatments. In the literature (Gatti & Macchi, 2012 ▸) there are some studies of experimental electronic structures of *3d* coordination compounds, but it is quite complicated to compare the particular results between each other. Some of them are more than 30 years old (Benard *et al.*, 1980 ▸). The enormous development of experimental devices, their different types with diverse detectors, as well as various integration software gives a source of experimental results with different types of small systematic errors [synchrotron (Poulsen *et al.*, 2004 ▸; Coppens *et al.*, 2005 ▸; Clausen *et al.*, 2008 ▸) or laboratory sources (Figgis *et al.*, 1993 ▸; Jensen *et al.*, 1995 ▸; Kožíšek *et al.*, 2002 ▸; Farrugia *et al.*, 2003 ▸; Pillet *et al.*, 2006 ▸; Shanmugam *et al.*, 2006 ▸; Gervasio *et al.* 2010 ▸; Farrugia *et al.*, 2012 ▸; Jørgensen *et al.*, 2013 ▸; Dos Santos *et al.*, 2016 ▸; Macchi *et al.*, 2018 ▸)]. The advantage of high-energy synchrotron radiation and small crystals is in the absence of extinction and absorption. ‘The combination of experimental and theoretical methods is an exceptionally powerful approach that can point out weaknesses in either method. It is evident that further pursuit of this approach will lead to new insight in the nature of metal–ligand and metal–metal bonding, and in the effect of intermolecular interactions on the electronic structure of molecules in solids’ (Coppens *et al.*, 2005 ▸).

The main features of charge density distribution of 3*d* coordination compounds are easily detectable, but difficulties arise in the determination of subtle effects, such as intermolecular interactions, π−π stacking interactions, hydrogen bonds, halogen bonds or metal–metal interactions. One of the reasons for these shortcomings could lie in absence of an appropriate correction for secondary extinction. The problem arose when point detectors were replaced by area detectors. Data collection with the point detector was always run in the θ–2θ scan mode with the hypothetical ψ angle equal to zero. Reflections measured several times were averaged. Reflections from the area detector are collected for an arbitrary ψ angle (0–360°) and for the anisotropic secondary extinction correction the direction cosines in the special *XD* (Volkov *et al.*, 2015 ▸) definition are needed. In our previous study (Kožíšek *et al.*, 2002 ▸), we have shown that a correction of this type improves the experiment–model agreement significantly. In the papers published to date, all the collected reflections are averaged using *SADABS* (Sheldrick, 2008 ▸; Krause *et al.*, 2015 ▸) or *SORTAV* (Blessing, 1995 ▸) software and so some pieces of the valuable information are lost (Sheldrick, 2012 ▸). Using *JANA2006* software (Petříček *et al.*, 2014 ▸) we are able to perform the secondary extinction correction (isotropic and/or anisotropic) using direction cosines. A correction of this kind is important for very intense low-order reflections which hold the information about the valence electron density. Integrated charge in the atomic basin of the central atom is strongly influenced by accurate intensities of a few strong low-order reflections.

Knowledge of the electron density distribution in ‘paddle-wheel’ carboxyl­ates should be helpful in the explanation of their bonding and magnetic properties. Charge density of tetra­kis­(μ_2_-acetato)di­aquadicopper(II) has been studied experimentally at 100 K and by density functional theory (DFT) calculations (triplet spin state only) on the isolated molecule using the AIM analysis (Bertolotti *et al.*, 2012 ▸). A more recent AIM DFT study of the electron density of the same compound (Shee *et al.*, 2015 ▸) [although the authors used pseudopotentials for copper atoms, which deformed the AIM characteristics of Cu—O bonds and Cu–Cu bonds, and presented opposite signs for the electron density Laplacians at bond critical points] concluded an antiferromagnetic exchange through direct Cu–Cu bonding and the ferromagnetic one through the acetate ligand pathway as implied by comparing the energies of triplet and ‘broken symmetry’ singlet states of the isolated complex for short and long Cu–Cu distances. We have found only one old experimental and theoretical (‘broken symmetry’ singlet restricted Hartree–Fock and configuration interaction calculations) charge density study of tetra­kis­(μ_2_-acetato)di­aquadichromium(II) by Benard *et al.* (1980 ▸).

In the present paper, we study the electron density of tetra­kis­(μ_2_-acetato)di­aquadicopper(II) and tetra­kis­(μ_2_-acetato)di­aquadichromium(II). The aim of our study is to experimentally investigate the structure and electron density topology of these isostructural coordination compounds and compare them to each other. We hope to shed light on the bonding properties in the studied compounds, *i.e.* the issues which are raised in various theoretical studies. Our experimental results might help to explain the structural and magnetic properties of copper acetate which is one of the most frequently studied dinuclear compounds of Cu^II^ (Elmali, 2000 ▸). Furthermore, it seems also that the Kepert model and the VSEPR theory could be improved by the results of charge density studies. We will show new insight into qualitatively different metal–oxygen bonds in tetra­kis­(μ_2_-acetato)diaqua­dicopper(II) and tetra­kis­(μ_2_-acetato)di­aquadichromium(II) complexes. A CSD search (Groom *et al.*, 2016 ▸) could also reveal some trends in the studied compounds with respect to apical and quasi-equatorial Cu—O dative (crystal/ligand field) interactions.

## 2. Experimental   

### 2.1. Material and methods   

A suitable recrystallized sample of tetra­kis­(μ_2_-acetato)­di­aquadicopper(II), structure (I), and tetra­kis­(μ_2_-acetato)­di­aquadichromium(II), structure (II), crystals were used for the high-resolution X-ray diffraction experiments. The data were collected at 100.0 (1) K on an Oxford Diffraction kappa geometry Gemini R diffractometer equipped with Ruby charged-coupled device area detector (for more details see section S1 in supporting information). Integration of the diffracted intensities, the Lorentz–polarization and face-absorption correction were performed with *CrysAlis PRO* (Rigaku Oxford Diffraction, 2016 ▸) software. After the data reduction, the .hkl files were treated with *JANA2006* to obtain xd.hkl files, with the direction cosines included, for the multipolar refinement. Details of the X-ray diffraction experiment conditions and the crystallographic data for (I) and (II) are given in Table 1 ▸.

### 2.2. Electron density refinements   

Both crystal structures are isostructural with approximately the same lattice parameters (Table 1 ▸). Starting atom coordinates and atom displacement parameters were taken from a routine *SHELXL* (Sheldrick, 2015 ▸) refinement and all other refinements were carried out on *F* ^2^ using the *XD* (Volkov *et al.*, 2015 ▸) suite of programs.

As the symmetry equivalent data were collected with a different value of TBAR (distance of primary and diffracted beam through the crystal), all non-averaged data were used in the refinements. Details on the XD-refinements are in Table SA1 (see Appendix I in supporting information).

In order to show benefits from multipole refinement including correction for anisotropic extinction correction we have made the calculations on averaged and non-averaged data for (I). The results are in Figs. SA2 and SA3 in Appendix I in supporting information. As may be seen, the distribution of charge density is a robust property and from both non-averaged and averaged data is very similar. The only better indicator for the averaged data is the *R* value. All others results give priority to the use of non-averaged data. Obviously, performing a correction for anisotropic extinction brings better results for error analysis and more details of the electronic structure can be depicted.

### 2.3. AIM analysis   

The electronic structure of the compound under study was investigated using AIM (Atoms-in-Molecule) topological analysis of electron density (Bader, 1990 ▸). The results were evaluated in terms of atomic charges obtained using the electron density integrated over atomic basins and bond characteristics in terms of electron density ρ at bond critical points (BCPs) corresponding to saddle points at bond paths between individual atoms, its Laplacian ∇^2^ρ
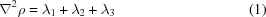
and bond ellipticity ∊
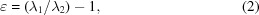
where λ_1_ < λ_2_ < 0 < λ_3_ are the eigenvalues of the electron density Hessian at BCPs. Ring critical points are saddle points with λ_1_ *<* 0 < λ_2_ < λ_3_ and cage critical points are local minima (0 < λ_1_ < λ_2_ < λ_3_) of electron density.

The BCP electron density, ρ_BCP_, is proportional to the bond strength; the value and sign of its Laplacian, ∇^2^ρ_BCP_, describes the relative electron density contribution of the bonded atoms to the bond (covalent *versus* dative bonding); its bond ellipticity, ρ_BCP_, describes its deviation from cylindrical symmetry (such as in ideal single or triple bonds) due to its double-bond character, mechanical strain and/or other perturbations.

The local source (LS) (Bader & Gatti, 1998 ▸; Bertini *et al.*, 2007 ▸; Farrugia & Macchi, 2009 ▸) function for source **r** is given by
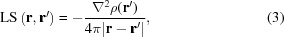
where the source operates over all other points **r**′. Most conveniently the source **r** is set to the BCP position. In general
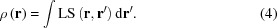
Herein, we will use the LS function plots along the bond path to characterize the *M*—O bonds of the studied systems (I) and (II). The sign of the BCP Laplacian, *i.e.* charge concentration or depletion, defines of the LS function behavior, *i.e.* its source or sink character. Hence, the electron density is pulled out of the source **r** where LS is positive, whereas negative LS regions push the electron density into the source **r**.

## 3. Results and discussion   

### 3.1. Structure description   

**Figure 1:**
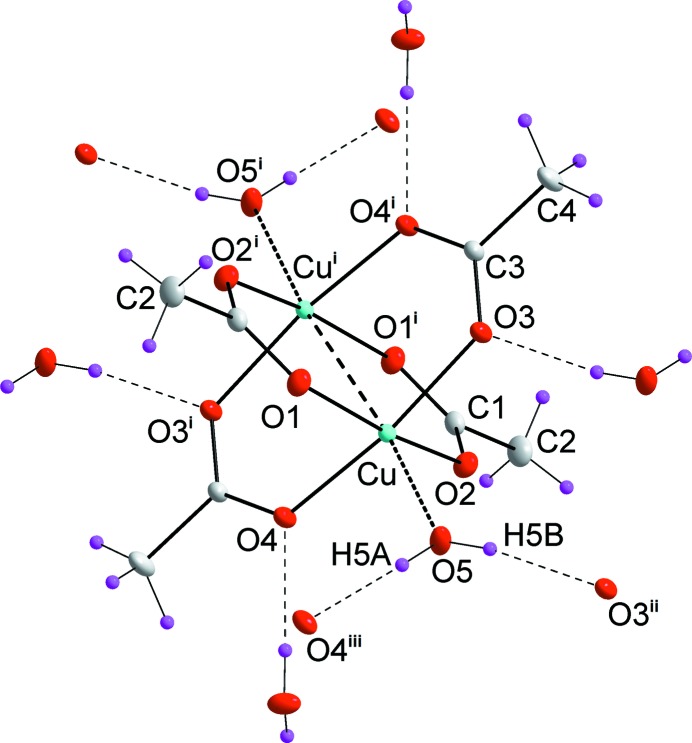
*ORTEP* plot of the compound (I). Displacement ellipsoids are drawn at 50% probability. Symmetry codes: (i) 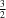 − *x*, 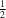 − *y*, 1 − *z*; (ii) 1 − *x*, 1 − *y*, 1 − *z*; (iii) −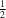 + *x*, 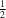 − *y*, −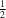 + *z*.

The coordination polyhedron of central metal atom *M* (*M* = Cu, Cr) with the ligating oxygen atoms is a tetragonal pyramid (Fig. 1 ▸) with four acetate oxygen atoms in the basal plane and the water oxygen atom in the apical position. The presence of the other metal atom at an *M*–*M* distance of 2.61082 (3) Å for (I) and 2.34779 (3) Å for (II) can be formally considered to be the sixth coordination place, *i.e.* forming a deformed octahedral coordination of each metal atom. Copper atom in (I) is shifted from the plane defined by basal plane atoms O1, O2, O3 and O4 by 0.1860 (1) Å towards the oxygen atom O5. The Cu^i^—Cu—O5 angle [here, symmetry code (i) is 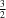 − *x*, 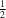 − *y*, − *z*] is 173.967 (3)°. The chromium atom in (II) is shifted from the plane defined by basal plane atoms O1, O2, O3 and O4 by only 0.0593 (1) Å. The Cr^i^—Cr—O5 angle (the same symmetry code as above) is 175.724 (3)°. Interatomic distances and angles, as well as hydrogen bonds are shown in Table 2 ▸.

**Figure 2:**
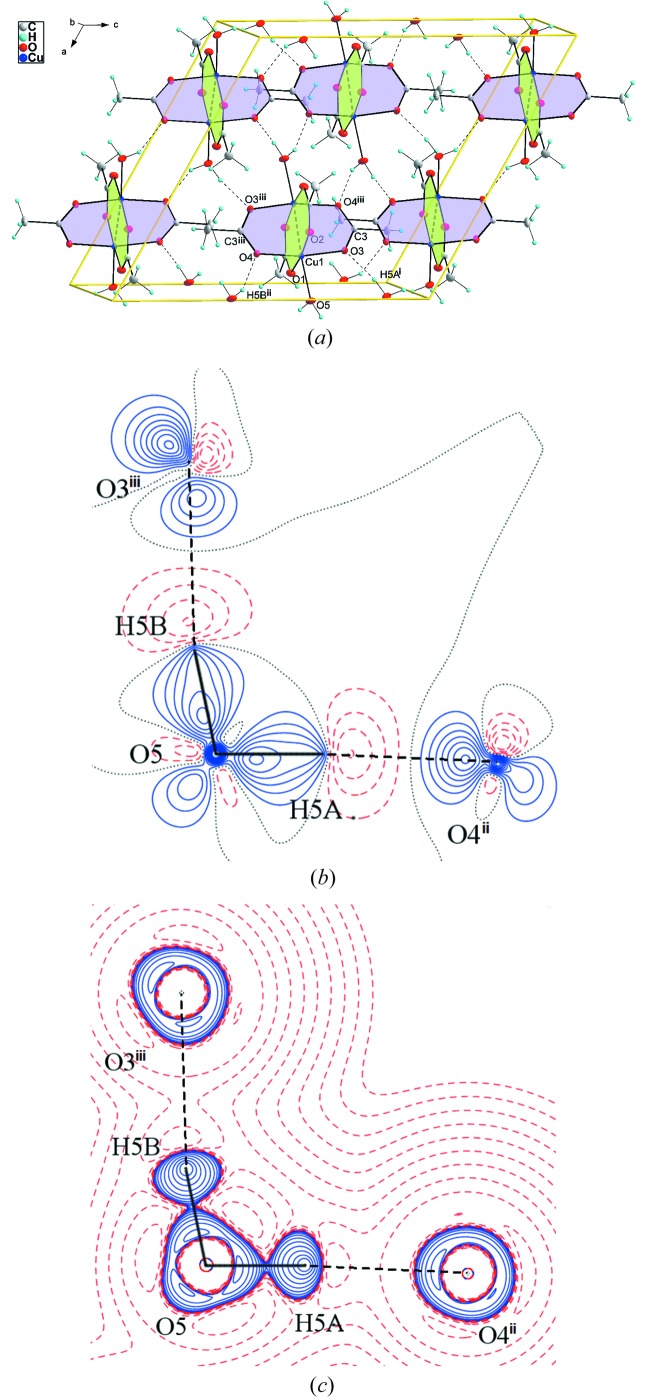
(*a*) Unit-cell content of (I). Two types of metalocycles are drawn in different colors. (*b*) Static electron deformation density of (I) in the plane defined by the atoms O5—O4^ii^—O3^iii^. Contour spacing 0.05 e Å^−3^, with positive contours drawn with a solid blue line and negative contours with a dashed red line. (*c*) Laplacian distribution L(**r**) ≃ ∇^2^ρ(**r**) of (I) in the O5—O4^ii^—O3^iii^ plane. Contours are drawn at −1.0 × 10^−3^, ±2.0 × 10^*n*^, ±4.0 × 10^*n*^, ±8.0 × 10^*n*^ (*n* = −3, −2, −1, 0, +1, +2, +3) e Å^−5^, with positive contours drawn with a solid blue line and negative contours with a dashed red line. Symmetry codes: (ii) 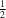 + *x*, 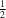 − *y*, 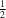 + *z*; (iii) 1 − *x*, 1 − *y*, 1 − *z*.

A multipole refinement achieved a significant improvement in the agreement between the experimental and calculated structure factors when compared with ordinary spherical atom structure refinement. Furthermore, the accuracy in the interatomic distances is increased by an order of magnitude from a routine *SHELXL* refinement. The obtained crystal structure geometry of (I) is in excellent agreement with the published X-ray data of F. R. Fronczek (2003, private communication; CSD code CUAQAC23) and the neutron diffraction study of Vives *et al.* (2003 ▸; CSD code VATNOT01). The positions of hydrogen atoms within the multipole refinement were taken from the neutron study which was carried out at 20 K. The obtained interatomic distances of non-hydrogen atoms differ by less than 1.5% compared with those of Vives *et al.* (2003 ▸). As might be seen from Figs. 2 ▸(*a*)–2 ▸(*c*), the bonding mode of acetate anions is not equal. The acetate group with oxygen atoms O1 and O2 does not form any hydrogen bonds. On the other hand, the acetate group with oxygen atoms O3 and O4 forms a three-dimensional network with water H atoms H5*A* and H5*B*. These hydrogen bonds form two eight-membered rings: *M*–O3⋯H5*B* ^ii^–O5^ii^–*M* ^ii^–O3^ii^⋯H5*B*–O5–*M* (*M* = Cu, Cr) [here symmetry code (ii): 1 − *x*, 1 − *y*, 1 − *z*] which is nearly planar, with the distance from the plane defined by *M*, O3 and O5 atoms, of 0.4877 Å for the Cu^ii^ atom and of 0.536 Å for the Cr^ii^ atom, and *M*–O4⋯H5*A* ^iii^–O5^iii^–*M* ^iii^–O4^iii^⋯H5*A*–O5–*M* (*M* = Cu, Cr) [here symmetry code (iii): 1 − *x*, *y*, 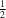 − *z*] which has a boat shape, with the distance from the plane defined by *M*, O4 and O5 atoms, of 1.657 Å for the Cu^ii^ atom and 1.737 Å for the Cr^iii^ atom. According to the electron density ρ at BCP these interactions result in *M*—O3 and *M*—O4 bonds that are weaker by 10% than *M*—O1 and *M*—O2 bonds, which seems to be due to sharing the bonding capability of the donor oxygen atom (see Table 3 ▸).

An error analysis shows the following results. The residual density calculated by fast Fourier synthesis for all reflections for (I) is 0.99 e Å^−3^ at 0.02 Å from Cu and −0.74 e Å^−3^ at 0.45 Å from Cu with a mean of 0.132 e Å^−3^ and for (II) is 0.36 e Å^−3^ at 0.18 Å from Cr and −0.44 e Å^−3^ at 0.25 Å from Cr with a mean of 0.086 e Å^−3^.

A fractal plot of the residual density (Meindl & Henn, 2008 ▸) has a symmetrical shape for the entire sinθ/λ range of the data set for (I) with ρ_min_ = −0.41 e Å^−3^, ρ_max_ = 0.43 e Å^−3^, and for (II) with ρ_min_ = −0.15 e Å^−3^, ρ_max_ = 0.14 e Å^−3^ (see Figs. S1a and S1b).

The normal probability distribution plot (Abrahams & Keve, 1971 ▸; Farrugia, 2012 ▸) shows a fairly good agreement with the supposed shape (Figs. S1a and S1b). The slope is of 45°, the function goes through the origin and is linear in the interval from −2 to 2 (Abrahams & Keve, 1971 ▸). The variation of the scale factor with respect to the resolution for (I) is about 5% smaller for the first group only and for (II) is close to unity for all groups (see Fig. S3). It could be said that the error analysis has affirmed a good agreement between the experimental and calculated structure factors.

### 3.2. Topological description of chemical bonding   

**Figure 3:**
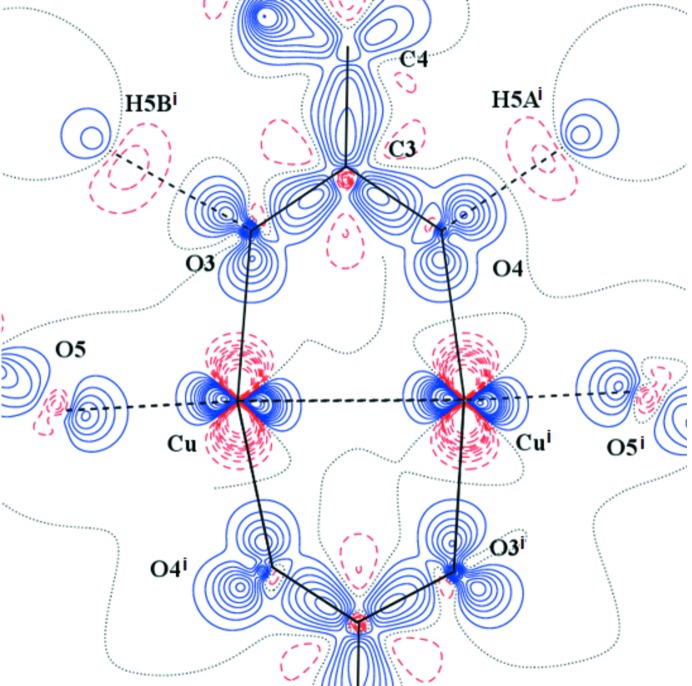
Static electron deformation density of (I) in the plane defined by the atoms Cu—Cu^i^—O3. Contour spacing as in Fig. 2 ▸(*b*). Here, symmetry code (i) is 1 − *x*, 1 − *y*, 1 − *z*.

**Figure 5:**
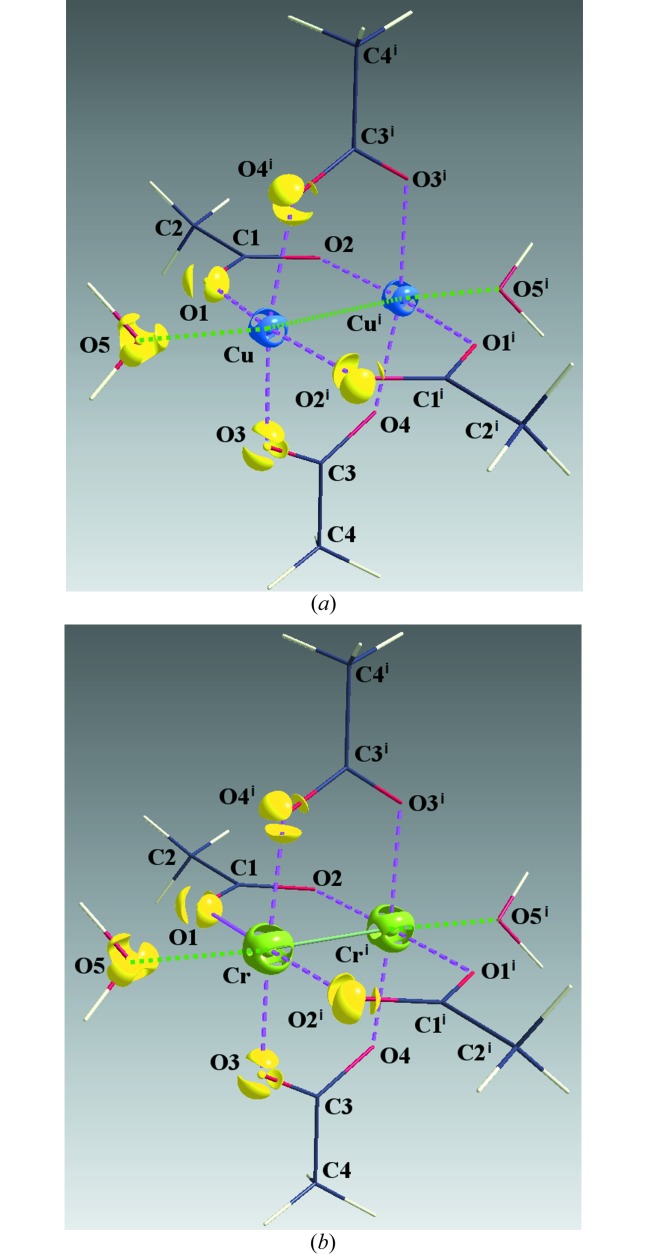
(*a*) Three-dimensional plot (Hübschle & Dittrich, 2011 ▸) of the Laplacian of electron density around Cu at the isosurface value of 1650 e Å^−5^ and around O1, O2^i^, O3, O4^i^ and O5 at the isosurface value of 90 e Å^−5^. (*b*) Three-dimensional plot (Hübschle & Dittrich, 2011 ▸) of the Laplacian of electron density around Cr at the isosurface value of 400 e Å^−5^ and around O1, O2^i^, O3, O4^i^ and O5 at the isosurface value of 90 e Å^−5^.

The AIM theory developed by Bader defines the charge density ρ_BCP_ and Laplacian ∇^2^ρ_BCP_ in BCP which are sensitive indicators of the strength and type of the bond. Our results for (I) are in good agreement with the previous study (Bertolotti *et al.*, 2012 ▸). Nevertheless, the herein reported crystal structure geometry and electronic structure is of better resolution than that reported by Bertolotti *et al.* (2012 ▸). The comparison of both isostructural compounds (I) and (II) also shows a high degree of similarity. Organic parts of both molecules are, in principle, the same. Significant differences are in the populations of *d*-orbitals of transition metal atoms only. This is visible on the maps of static deformation densities and of their Laplacians, but only in the central metal atom region. For Cr, the positive contours (solid blue lines) in static deformation densities are more diffuse and for Cu more compact (Figs. 3 ▸, 4 ▸ and S4–S9). To obtain similar shapes for three-dimensional Laplacian isosurfaces the values of 1650 e Å^−5^ for compound (I) and of 400 e Å^−5^ for compound (II) are required [Figs. 5 ▸(*a*) and 5 ▸(*b*)]. In both cases, if the isosurface value is lowered by 20%, the depletion of charge concentration towards the coordination bonds disappears. It is apparent that on the three-dimensional charge density Laplacian surface there is a hole at the central metal atom in the direction to the donor oxygen atoms (to the lone electron pair) in the basal plane. Identical results were obtained in other published results of charge density studies of Cu^II^ complexes (Kožíšek *et al.*, 2002 ▸; Pillet *et al.*, 2006 ▸; Overgaard *et al.*, 2007 ▸; Farrugia *et al.*, 2008 ▸; Bouhmaida *et al.*, 2010 ▸). Similarly, in the paper by Farrugia & Senn (2010 ▸) on charge density of Fe_3_(μ-H)(μ-COMe)(CO)_10_ their Figure 9 clearly shows the interaction of VSCC (valence-shell charge concentration) on atom C1 of the COMe ligand pointing to the depleted area of Fe2 and Fe3, respectively. This is consistent with the positive values in Table 2 ▸ for ∇^2^ρ(BCP) Fe2—C1 and Fe3—C1 and a coordination bond is expected. However, negative ∇^2^ρ(BCP) values for C1—O1 and C2—O1 of the COMe ligand indicate the covalent character of these bonds.

**Figure 6:**
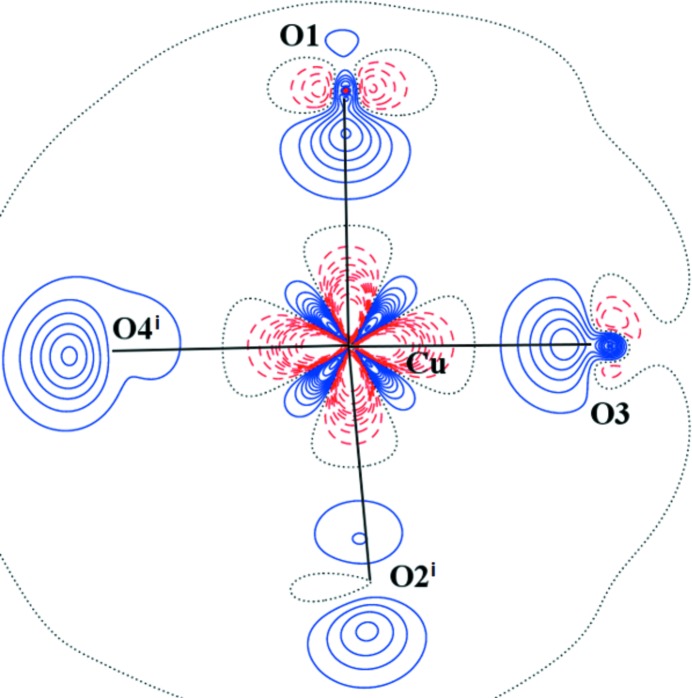
Static electron deformation density in the plane Cu–O3–O1. Contour spacing as in Fig. 2 ▸(*b*). Symmetry code as in Fig. 3 ▸.

If, for the purpose of this paper, we define the term Classical Coordination Bond (CCB) as the bond which is realized by depopulated *d*-orbital of the central metal atom and the lone electron pair of the ligand, we can say that both Cu and Cr central metal atoms have only four coordination bonds in our compounds. The existence of these four CCBs in the basal plane of the pyramid is evident from the maps of static deformation densities [Figs. 6 ▸ and 7 ▸ (Cu, O3, O1 and Cu, O5, O3) and Figs. S10 and S15 (the same planes but Cr instead of Cu)]. As the central *M* atom is not placed in the plane defined by the oxygen atoms O1, O2, O3 and O4, only two metal–oxygen coordination bonds can be exactly depicted within any plane definition. The distance of the Cu central atom from the basal plane is about three times longer than for the Cr one; thus, the lone electron pair of any oxygen atom opposite to the another one, which defines the basal plane of the pyramid in the case of the Cu complex, is less pronounced (Figs. 6 ▸ and  S10). The lone electron pair of the oxygen atom points towards the depleted central atom 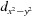 orbital. The nonbonding *d_xy_* orbital is also pronounced. The same features are also visible in electron density Laplacians (Figs. S12 and S15) and correspond with the values in Table 3 ▸. The isostructural similarity of compounds (I) and (II) is mirrored in all characteristics of AIM. In order to demonstrate the high accuracy and consistency of our results, we would like to point out the found interrelation of the binding forces of coordination and hydrogen bonds. The values of the BCP electron density ρ_BCP_ and Laplacian ∇^2^ρ_BCP_ are sensitive indicators of the quality of the bond. Nevertheless, the electron density description in terms of AIM analysis should take into account the warnings presented in the recently published review papers (Dittrich, 2017 ▸; Macchi, 2017 ▸). We agree that AIM results should be carefully compared with three-dimensional distribution of electron density and with the literature as well. Comparison with the theoretical data will be helpful as well. Our paper contains only results of the experimental study, theoretical studies are in progress.

The higher value of ρ_BCP_ and the higher positive value of ∇^2^ρ_BCP_ is observed for a stronger coordination bond. Our findings are in good agreement with the results of Farrugia *et al.* (2008 ▸), where for short basal plane bonds [1.9270 (3)–1.9474 (2) Å], the ρ_BCP_ values are in the interval from 0.53 to 0.62 e Å^−3^ and ∇^2^ρ_BCP_ values from 11.72 to 13.23 e Å^−5^. On the other hand, for longer basal plane bonds [1.9933 (3)–2.0197 (6) Å] the corresponding values are from 0.45 to 0.50 e Å^−3^ and from 9.91 to 10.36 e Å^−5^, respectively (Table 3 ▸).

Our results show the same trend and, moreover, they are also sensitive to the fact that oxygen atoms which are involved in hydrogen bonds (O3 and O4) have longer *M*—O bonds [Cu—O values of 1.9843 (1) Å and 1.9905 (1) Å for (I)] than atoms O1 and O2 without any hydrogen bonding [1.9548 (1) Å and 1.9423 (1) Å, respectively]. Analogously for (II), Cr—O3 and Cr—O4 bond lengths of 2.0294 (1) Å and 2.0292 (1) Å are longer than the Cr—O1 and Cr—O2 ones for (II) [2.0130 (1) Å and 1.9999 (1) Å, respectively] (Table 2 ▸). The values of ρ_BCP_ and ∇^2^ρ_BCP_ are smaller for the bonds involved in the hydrogen bonds. For (I) these values for the coordination bonds increase in the order O4 < O3 < O2 < O1 (0.41, 0.44, 0.45, 0.47 e Å^−3^) and O4 < O3 < O1 < O2 (10.88, 11.36, 12.46, 12.80 e Å^−5^, respectively). For (II), this trend is the same for both O4 < O3 < O1 < O2 (0.41, 0.41, 0.43, 0.44 e Å^−3^ and 11.62, 11.70, 12.18, 12.46 e Å^−5^, respectively) (Table 3 ▸).

In both crystal structures the same situation was found for C—O and C—C bonds. The ρ_BCP_ values for C—O bonds are in the interval from 2.59 to 2.72 e Å^−3^ and ∇^2^ρ_BCP_ values are in the interval from −34.00 to −30.94 e Å^−5^ for (I), which is typical for covalent delocalized single and double bonds as supposed for C(*sp* ^2^) atoms. On the other hand, the same bonds descriptors for C—C bonds are in the interval from 1.79 to 1.81 e Å^−3^ and from −12.03 to −12.77 e Å^−5^ for (II), respectively (Table 3 ▸). For O⋯H hydrogen bonds these descriptors are in the interval from 0.050 to 0.108 e Å^−3^ and from 2.38 to 5.97 e Å^−5^, respectively. In (I), the hydrogen bond O4⋯H5*A* is found to be the strongest one. Carbonyl carbon atoms have more positive charge compared with the methyl ones and about one half of atomic volume (Table 4 ▸). For bonds in organic compounds the ellipticity is an indicator which unambiguously distinguishes between the character of the bond (single, double or triple) but in cyclic structures it includes the bond strain as well. In the title compounds we presuppose single *M*—O bonding with acetate and water ligands. Thus the increased ellipticity of *M*—O bonds could express the bond strain which increases in the order O5 ∼ O1 ∼ O2 < O3 ∼ O4 for (I) and O5 < O1 ∼ O2 ∼ O3 ∼ O4 for (II). Since the acetate groups are relatively rigid and depopulated, the 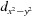 orbital of the central *M* atom forms CCBs with lone electron pairs of four acetate oxygen donor atoms. It is evident that the *M*—O_carboxyl_ bonds are responsible for stabilizing the dimer. The acetate group distances O1—O2 and O3—O4 [2.2338 (2) Å and 2.2272 (1) Å for (II), and 2.2460 (1) Å and 2.2353 (1) Å for (I), respectively] are rather similar. Electrostatic potential in planes *M*–O1–O2, as well as *M*–O3–O4 clearly shows that the *M*–*M* interaction is arranged by acetate groups. The ring critical point in the *M*–O–C–O–*M* ^ii^ plane is slightly shifted towards the longer *M*—O bond (Cu—O1, Cu—O4, Cr—O1 and Cr—O4, respectively). BCPs of *M*—O bonds are in all cases slightly shifted towards the *M*–O–C–O–*M* ^ii^ ring which indicates the *M*–*M* ^ii^ repulsion (Figs. 8 ▸, 9 ▸, S16 and S17).

**Figure 7:**
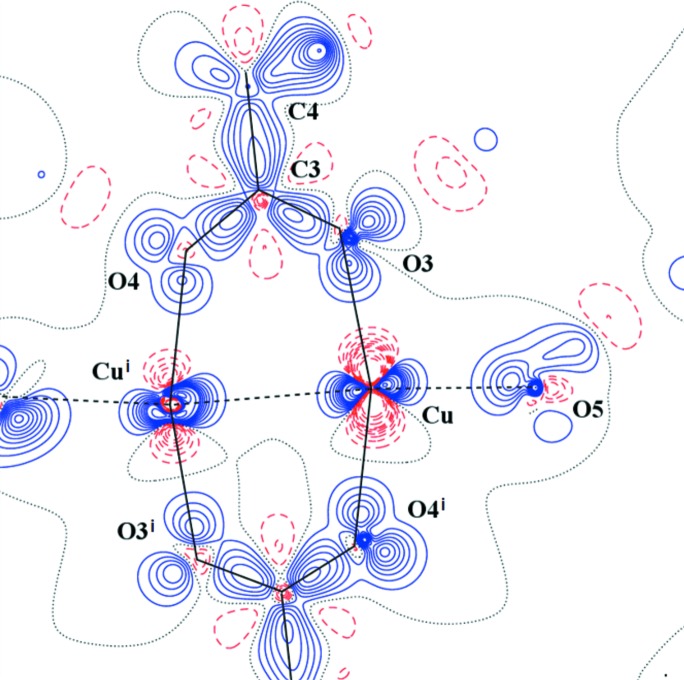
Static electron deformation density in the plane Cu–O5–O3. Contour spacing as in Fig. 2 ▸(*b*). Symmetry code as in Fig. 3 ▸.

**Figure 8:**
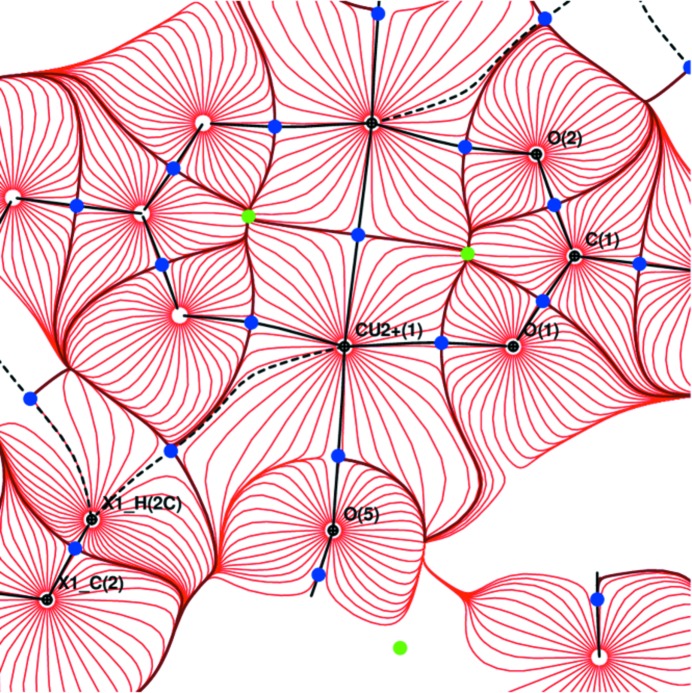
Gradient field trajectory plot of electrostatic potential in the plane Cu–O1–O2.

A totally different situation occurs in the axial direction – facing the water molecule, or towards another metal atom (Figs. 7 ▸ and S11). There is not a depletion, but a concentration of electron density at the central atom 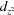 orbital. Similar features could be seen in Figs. 3 ▸ and 4 ▸ (see also Figs. S4–S9), where the planes are defined by atoms *M*–*M* ^i^–O1 or *M*–*M* ^i^–O3. The *M*–O5 (*M* = Cu, Cr) interaction is not a CCB, this interaction is just a combination of some kind of the repulsion of the fully/half-populated *M*(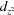) orbital with the lone electron pair of the water oxygen atom and of the attraction between the positive charge of metal cation (*M* ^*q*+^, the *q* is between 1 and 2) and the lone electron pair of the water oxygen atom. The corresponding values of *M*–O5 BCP characteristics are ρ_BCP_ (Cu–O5) = 0.30 e Å^−3^ and ∇^2^ρ_BCP_ (Cu–O5) = 7.03 e Å^−5^ for (I) and ρ_BCP_ (Cr–O5) = 0.25 e Å^−3^ and ∇^2^ρ_BCP_ (Cr–O5) = 5.69 e Å^−5^ for (II). Note also that there is no charge transfer from the water molecule to the central metal atom (water almost neutral; Table 4 ▸).

In both compounds the bonds between the central metal atom and donor oxygen atoms in the basal plane of pyramid and in the apical direction are different. The bonds in the basal plane of the pyramid give a textbook example of coordination bonding. For the apical bond we argue upon the findings that this bond is a rather weak VSCC interaction of the lone electron pair of the water molecule, or 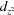 orbital of other central atom in our case, including contributions from the depopulated 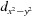 orbital [similar to the findings reported by Dos Santos *et al.* (2016) ▸; see Fig. SA4 in Appendix I in supporting information]. The same behavior was also found by Overgaard *et al.* (2007), in which the four coordination bonds in the quasi-equatorial plane are very well depicted. On the other hand, the apical bond in that paper has been interpreted [Cu—O10 bond of 2.1692 (3) Å] as the weaker bonded ligand to the Cu atom (a weaker coordination bond). They also stated: 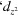 orbital is pointing in the direction of the axial water ligand’, but the mutual angle between the Cu 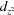 orbital and lone electron pairs of the water oxygen atom is ∼25°. Such an explanation is in good agreement with the equatorial–axial interaction (Gažo *et al.*, 1976 ▸; Valach *et al.*, 2018 ▸). Short Cu—L(quasi-equatorial) bonds correspond to larger Cu—L(axial) bonds and *vice versa*. Sharing the bonding capability is similar to that in acetate oxygen donor atoms in the case of the absence or presence of hydrogen bonds (compare O1, O2 and O3 and O4, respectively).

The apical water molecule is bonded *via* strong hydrogen bonds with two donor oxygen atoms of the acetato groups of the adjacent dimers. Hydrogen bonds push the water molecule more closely to the central metal atom in such a way that the fully [in (I)] / half- [in (II)] populated 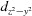 orbital points not directly towards the water oxygen atom, but about 20–30° away from it. The angle between the Cu–Cu line and the direction of the lone electron pair formed by the 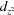 orbital is ∼20°. This might reflect the repulsion between two 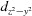 orbitals (Cu, Cu) which causes such an orientation. On the other hand, the O3 and O4 lone electron pairs (solid blue lines) point to the Cu atom areas of lower VSCC (dashed red lines) [Figs. 3 ▸, 5 ▸(*a*), 7 ▸ and S6]. Furthermore, the Cu⋯O5 interaction is not a typical coordination bond when also considering the deformation densities (solid blue *versus* solid blue) [Figs. 3 ▸, 5 ▸(*a*) and 7 ▸ and S6].

The differences in the shape of the local source function for *M*—O bonding reflect the differences in the BCP location and values of the electron density Laplacian in the region around BCP for various *M*—O bonds. Hence, since the position of the BCP is much closer to the *M* for O1, O2, O3 and O4 relative to O5 (an effect of the better alignment of the oxygen lone pair in the former four *M*—O bonds), the LS ‘drop’ around the BCP location is closer to *M* in the former four bonds (Fig. S19). This shift towards *M* is enhanced for oxygen donor atoms taking part in the hydrogen bonds (O3 and O4). In *M*—O(*X*) (*X* = 1 or 4), the LS ‘drop’ is not only shifted towards *M*, but it is much more pronounced, compared with *M*—O5, due to the almost doubled value of the electron density Laplacian at the BCPs in the four former bonds. The value of the corresponding BCP electron density Laplacian for (I) is only 7.026 (1) e Å^−5^ and the electron density is also smaller [0.295 (1) e Å^−3^] compared with the BCPs of four basal plane *M*—O coordination bonds (Table 3 ▸). The same holds for (II) [5.690 (1) e Å^−5^ and 0.252 (1) e Å^−3^, respectively]. The angles between the vectors defined by the atom nucleus and the valence-shell electron density (both in static deformation maps and in the maps of its Laplacian as well) for the central metal atom and for the water oxygen atom are in the interval 20–30°.

Fig. S19 shows the atomic graphs of the Cu atom in (I) depicting the critical points in *L*(**r**) = ∇^2^ρ_BCP_ determined from the experimental densities. Topological analysis of the Laplacian of the electron density shows the regions of VSCC and depletion around the metal center, thus qualifying in further detail the differences between the apical and equatorial coordination to the metal atom. The Laplacian concentrations in the equatorial plane (green points) maximally avoid the ligand lone electron pairs, which is consistent with the simple ligand-field approach. The cage critical points (blue) of the Laplacian are close to the Cu—O bonds in the equatorial plane. On the other hand, the low symmetry of the coordination polyhedron leads to a more complicated structure of the Laplacian topology which does not allow a pictorial interpretation about the bond and ring critical points of Fig. S19 as was performed for the metal–carbonyl complexes reported by Farrugia & Evans (2005 ▸).

In our studied compounds, we have noticed a system of strong hydrogen bonds which brings the water molecule close to the Cu central atom. Nevertheless, it cannot be ascribed to a pseudo-Jahn–Teller distortion and its destabilization effect as it is presented in many papers (Farrugia, 2012 ▸; Overgaard *et al.*, 2007 ▸), because there are definitely no CCBs in the apical position. It seems that the hydrogen bonds push the water ligands towards the central metal atoms and due to the mutual repulsion between metal 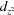 orbitals and lone electron pairs of water oxygen atoms the mutual orientations of these orbitals are declined from the direct metal–oxygen lines corresponding to CCBs. The angle between the direction of the oxygen lone pair and the *M*—O direction is between 20 and 30° and we suppose the same situation as in the case of the halogen–halogen interaction (Pavan *et al.*, 2013 ▸; Hathwar & Guru Row, 2010 ▸). We suppose that the spatial arrangement of electron density distribution on water oxygen atom O5 with *sp* ^3^ hybridization is governed by strong hydrogen bonds. Hereby, the orientation of lone electron pair, as well as the VSCC is a compromise between the repulsion (lone pair 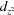 orbital) and attraction (dipole moment of water – positive charge of copper), including contributions from the depopulated 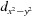 orbital towards the lone pair as well as low depopulation of the 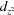 orbital; hence allowing for a weak dative Cu⋯O interaction, leading to a bent orientation of the 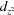 orbital with the lone pair of oxygen (which is further affected by the existence of hydrogen bonds with the apical water molecule ligand).

### 3.3. General considerations on bonding   

Both the results of multipole refinement and the results of the topological analysis are fully consistent and support the idea that the bond of the apical water molecule to the central atom differs from the other four acetate *M*—O bonds. This bond is not a CCB in the strict limit of dative interactions of a lone pair of the ligand with an empty *d* orbital of the central atom. Similarities of apical bonds in our complexes and axial bonds in the [CuO_6_] chromophore led us to study these types of crystal structures deposited in the CSD (version 5.38, update May 2017; Groom *et al.*, 2016 ▸). We have examined 232 copper crystal structures with the [CuO_6_] chromophore and an *R* value less than 0.030. Except the [Cu{(CH_3_)_2_SO}_6_]I_4_ crystal structure with the code FILDAH (Garzón-Tovar *et al.*, 2013 ▸), we have not found any case when the atom in the axial position is bonded only to the central atom and has no hydrogen bond or other interaction to the other part of the molecule (the same or the adjacent) [section S20 in the supporting information]. At first it is found that apical Cu—O distances are always longer than the quasi-equatorial ones. In addition, the position of such donor atoms is affected by other bonds or interactions. All the ligands in the axial position have other bonds as well, mainly hydrogen ones, which affect the particular location of the oxygen donor atom towards the central atom. Without the assistance of such complementary bonds, the axial/apical position appears significantly different and/or distinguished from the equatorial/quasi-equatorial one.

In the case of FILDAH (Garzón-Tovar *et al.*, 2013 ▸), there are six equivalent Cu—O bonds with equal lengths of 2.102 (3) Å due the rhombohedral symmetry. Their equivalence can be explained by a dynamical Jahn–Teller effect due to averaging of all possible orientations of a low-symmetric centrosymmetric complex cation in the crystal at real temperatures. Quantum-chemical calculations (for details see Table S21) of the isolated [Cu{(CH_3_)_2_SO}_6_]^2+^ complex give four shorter and two longer Cu—O bonds.

In copper acetate (Kožíšek *et al.*, 2013 ▸), we have found experimental evidence of metal–metal interactions of the same kind as reported for halogen–halogen ones by Guru Row and his group (Pavan *et al.*, 2013 ▸; Hathwar & Guru Row, 2010 ▸). The explanation of this interaction is based on a so-called σ-hole concept (Politzer *et al.*, 2007 ▸; Clark *et al.*, 2007 ▸; Eskandari & Zariny, 2010 ▸). In the structure with fluorine (Pavan *et al.*, 2013 ▸), the F⋯F distance is 2.824 Å, ρ_BCP_ is 0.04 e Å^−3^ and ∇^2^ρ_BCP_ is 0.9 e Å^−5^. The contact where one fluorine atom acts as a donor and the other one as the acceptor, is known as a type II contact (Eskandari & Zariny, 2010 ▸). In the structure with chlorine (Hathwar & Guru, 2010 ▸), there are three Cl⋯Cl contacts with the values (i) *d*_Cl⋯Cl_ = 3.5747 (2) Å, ρ_BCP_ = 0.03 e Å^−3^, ∇^2^ρ_BCP_ = 0.41 e Å^−5^; (ii) *d*_Cl⋯Cl_ = 3.3172 (1) Å, ρ_BCP_ = 0.0503 e Å^−3^, ∇^2^ρ_BCP_ = 0.66 e Å^−5^, and (iii) *d*_Cl⋯Cl_ = 3.4668 (2) Å, ρ_BCP_ = 0.0303 e Å^−3^ and ∇^2^ρ_BCP_ = 0.47 e Å^−5^. On the ‘surface’ of the copper atom there is no uniform charge distribution: there are areas of higher or lower charge concentrations. The less negative (relatively more positive) part of the central atom (lower shielding by non-uniformly distributed *d*-electrons) is less repulsed (or more attracted) by the negative charge of the 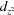 orbital [see Figs. 3 ▸ and 5 ▸(*a*)]. For the central metal atom, the relatively more positive charge on the surface of a sphere with an arbitrary radius is therefore shielded in a different manner. These particular areas interact with each other, which implies peaks at the map of static deformation density and its Laplacian at the bond critical point gains a positive value which could be explained by the electrostatic interaction of 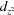 orbitals with the less negative (or more positive) area of the other central atom and *vice versa*. The repulsion between two 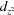 orbitals of Cu in (I), each occupied by two electrons, is higher than between orbitals occupied by a single electron only of Cr in (II). In contradiction with the known trend in ionic radii, the Cu⋯Cu interatomic distance is longer than the Cr⋯Cr one. The reason for this is in the different repulsion of negative charges concentrated in the area between two central metal atoms. Metal–metal repulsion is observable also in the position of the central atom above the basal plane defined by four donor oxygen atoms in the direction towards the water molecule which is 0.184 Å for (I) and 0.058 Å for (II). The *M*–*M* interaction gives ρ_BCP_ (Cu–Cu) = 0.06 e Å^−3^ and ∇^2^ρ_BCP_ (Cu–Cu) = 1.65 e Å^−5^ for (I) and ρ_BCP_ (Cr–Cr) = 0.17 e Å^−3^ and ∇^2^ρ_BCP_ (Cr–Cr) = 5.00 e Å^−5^ for (II) (Table 3 ▸).

### 3.4. Atomic characteristics   

The occupancies of *d*-orbitals calculated from multipole population parameters are given in Table 5 ▸. These values are very sensitive to the accurate scaling and the absorption correction. The maximum values should not exceed two electrons per orbital; this is a good check of a reliable absorption and extinction correction. The *d*-orbital populations in Table 5 ▸ are in a good agreement with the features observed in Fig. 3 ▸ and with the topological analysis in Table 3 ▸. The non-bonding orbitals 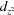 and *d_yz_* are fully populated in the case of Cu compound (I) and half populated in the case of the Cr one (II). The electron configuration of the Cu atom is nearly *d* ^9^ and of Cr atom is nearly *d* ^4^; the missing one electron in the 3*d* shell has according to the populations of *d*-orbitals a 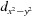 orbital character in both compounds studied (Table 5 ▸).

Integration of electron density in the atomic basin by *XDROP* program gives central atom charges of +1.49 for Cu and of +1.55 for Cr (Table 4 ▸). Charges of carboxyl oxygen atoms in both compounds are in the interval from −0.96 to −1.13. The charges of oxygen atoms of water molecules are slightly more negative, −1.23 for (I) and −1.30 for (II). Charges of carbon atoms bonded to oxygen atoms in both compounds are in the interval +1.29 to +1.43 and charges of methyl group C atoms are close to zero. The hydrogen atoms of water molecules in both compounds are positively charged, corresponding to strong hydrogen bonds with the oxygen atoms of the adjacent acetate groups.

Moreover, the charge of water oxygen atom O5 in the title compounds is, by about 15%, more negative than the charge of acetate oxygen atoms O1–O4. This is in the agreement with our statement that the Cu—O5 bond is not a classical coordination bond as it is widely supposed.

According to various sources, ionic radii are 0.84 Å (Cr^2+^) and 0.69 Å (Cu^2+^) (Prakash *et al.*, 2007 ▸) or 0.73 Å (Cr^2+^, low spin), 0.80 Å (Cr^2+^, high spin) and 0.73 Å (Cu^2+^) (Housecroft & Sharpe, 2001 ▸). These ionic radii do not obey the known rule that ionic radii having the same charge decrease with an increase in atomic numbers. Experimentally found Cr–Cr and Cu–Cu distances are 2.34798 (4) Å and 2.61043 (2) Å, respectively. According to our understanding, the direct metal–metal interaction in the title compounds is mainly electrostatic (a combination of the electrostatic repulsion of positively charged nuclei and of the repulsion of negatively charged nearly fully occupied or half-occupied 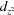 orbitals). Differences of 0.26 Å could be explained by the stronger repulsion in (I) and/or partial metal–metal bonding (sharing) interactions in (II), as rigid acetate groups act similarly for both (I) and (II).

## 4. Conclusion   

We have proved by means of charge density studies that in the title compounds the metal atoms have four CCBs. If the acetate donor oxygen atom is involved in hydrogen bonding, the strength of its bond with the central atom decreases. The apical bond of the water molecule to the central metal atom is not a mutual CCB. The mutual interaction between central metal atoms and water oxygen atoms is a compromise between repulsion (lone pair 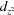 orbital) and attraction (dipole moment – charge on central atom as well as lone-pair 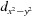 orbital) interactions. The interaction between the two central metal atoms is prevailingly established by four acetate groups bonded to two central metal atoms *via* CCBs. The direct *M*–*M* interaction in the title compounds is mainly of electrostatic character. The nucleus is positively charged and its shielding by *d*-electrons (which are not regularly distributed) leads to different local charges at the surface of a sphere of an arbitrary diameter. The direction of the fully/half-occupied 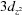 orbital of the central *M* atom is not parallel to the *M*–*M* line. Due to the center of symmetry between both *M* atoms, their negatively charged 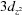 orbitals are directed towards the area of the more positive (less negative) part of the opposite *M* atom. This situation corresponds to an electrostatic interaction instead of a chemical bond. Motivated by the properties of axial Cu⋯O interactions, we have examined coordination compounds in the CSD with a [CuO_6_] chromophore, finding that all (but one) have four quasi-equatorial CCBs only, as in our case (while the remaining two axial ones have almost exactly the same properties as the axial interaction in the compounds under study). Hence, a weak sharing of the bonding capacity of the 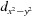 orbital with the 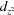 one could explain the quasi-equatorial–axial interactions towards the apical lone pair of oxygen O5. We have also proved that averaging the data does not bias the organic part of the molecule, but has a significant influence on the central atom.

## Supplementary Material

Crystal structure: contains datablock(s) global, I, II. DOI: 10.1107/S2052520618013707/px5002sup1.cif

Charge density and theoretical calculations. DOI: 10.1107/S2052520618013707/px5002sup2.pdf

CSD search on [CuO6] chromophore. DOI: 10.1107/S2052520618013707/px5002sup3.pdf

Appendix I. DOI: 10.1107/S2052520618013707/px5002sup4.pdf

xd.hkl data with direction cosines for Cu structure. DOI: 10.1107/S2052520618013707/px5002sup5.txt

xd.hkl data with direction cosines for Cr structure. DOI: 10.1107/S2052520618013707/px5002sup6.txt

CCDC references: 1811668, 1811669

## Figures and Tables

**Figure 4:**
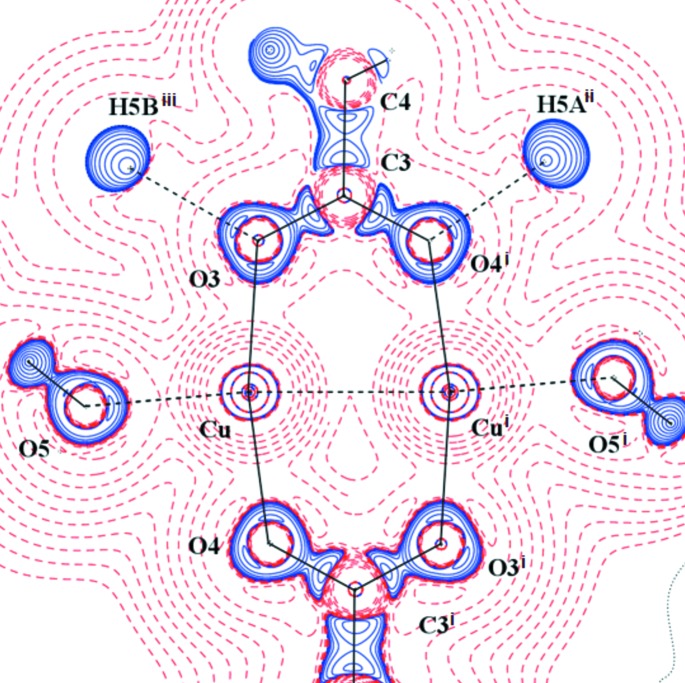
Laplacian distribution L(**r**) ≃ ∇^2^ρ(**r**) of (I) in the Cu–Cu^i^–O3 plane. Contour spacing as in Fig. 2 ▸(*c*). Here, symmetry code (i) is 1 − *x*, 1 − *y*, 1 − *z*.

**Figure 9:**
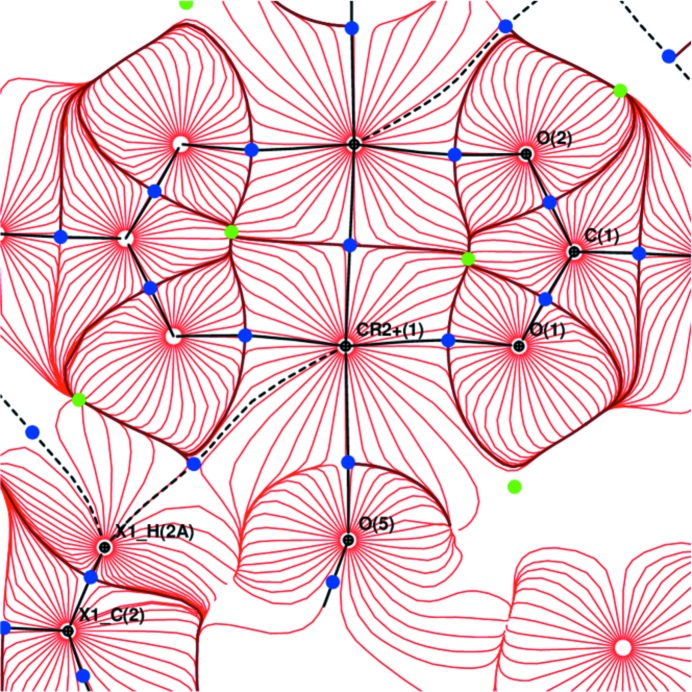
Gradient field trajectory plot of electrostatic potential in the plane Cr–O1–O2.

**Table 1:** Experimental details For both complexes: monoclinic, space group *C*2/*c*, *Z* = 4, Mo *K*α radiation. Crystal–detector distance was 60 mm. H atoms treated by a mixture of independent and constrained refinement.

	(I)	(II)
Crystal data		
Chemical formula	C_8_H_16_Cu_2_O_10_	C_8_H_16_Cr_2_O_10_
*M*_r_	399.31	376.20
Temperature (K)	100	100
*a*, *b*, *c* (Å)	13.08438 (10), 8.50319 (10), 14.05022 (10)	13.10149 (13), 8.56324 (11), 14.14837 (15)
β (°)	119.3086 (6)	119.1207 (14)
*V* (Å^3^)	1363.11 (2)	1386.68 (3)
*F*(000)	808	768
μ (mm^−1^)	3.17	1.61
Crystal size (mm)	0.43 × 0.17 × 0.13	0.37 × 0.22 × 0.12

Data collection		
Diffractometer	Xcalibur	Xcalibur
Scan method	ω scans	ω scans
		
Absorption correction	Analytical (*CrysAlis PRO*)†	Analytical (*CrysAlis PRO*)†
*T*_min_, *T*_max_	0.392, 0.721	0.684, 0.851
No. of measured, independent and observed [*I* > 3σ(*I*)] reflections	404 013, 12 312, 11 774	150 754, 11 225, 10 843
Redundancy	31.9	13.7
*R*_int_	0.026	0.020
*R*(σ)	0.005	0.008
(sin θ/λ)_max_ (Å^−1^)	1.295	1.234

Refinement		
*R*[*F* ^2^ > 2σ(*F* ^2^)], *wR*(*F* ^2^), *S*	0.019, 0.046, 1.20	0.018, 0.03, 1.26
No. of reflections	339 265	131 766
No. of parameters	282	282
Δρ_max_, Δρ_min_ (e Å^−3^)	0.99, −0.74	0.36, −0.44

Computer programs: *CrysAlis PRO* (Rigaku Oxford Diffraction, 2016 ▸), *SHELXS* (Sheldrick, 2016 ▸), Volkov *et al.* (2015 ▸).

† Analytical numeric absorption correction using a multifaceted crystal model based on expressions derived by Clark & Reid (1995 ▸).

**Table 2:** Selected interatomic distances (Å), angles (°) and hydrogen bonds (Å, °)

	(I) *M* = Cu	(II) *M* = Cr		(I) *M* = Cu	(II) *M* = Cr
*M*—O1	1.95475 (8)	2.01299 (10)	*M* ^i^—*M*—O1	83.050 (3)	87.575 (3)
*M*—O2^i^	1.94228 (8)	1.99985 (10)	*M* ^i^—*M*—O2	86.253 (3)	89.164 (3)
*M*—O3	1.98431 (9)	2.02938 (10)	*M* ^i^—*M*—O3	86.771 (2)	89.145 (3)
*M*—O4^i^	1.99045 (10)	2.02917 (11)	*M* ^i^—*M*—O4	82.431 (2)	87.418 (3)
*M*—O5	2.14884 (8)	2.25798 (11)	*M* ^i^—*M*—O5	173.967 (3)	175.724 (3)
*M*—*M* ^i^	2.61082 (3)	2.34779 (3)	O1—*M*—O2^iii^	169.249 (4)	176.648 (4)
O1—C1	1.26778 (13)	1.26941 (13)	O3—*M*—O4^iii^	169.030 (3)	176.117 (4)
O2—C1	1.26249 (13)	1.26608 (13)	O1—C1—O2	125.324 (8)	123.515 (9)
O3—C3	1.26409 (12)	1.26658 (13)	O3—C3—O4	124.245 (9)	122.744 (10)
O4—C3	1.26763 (11)	1.27060 (13)	*M*—O1—C1	124.122 (7)	120.281 (7)
C1—C2	1.50415 (12)	1.49966 (13)	*M* ^i^—O2—C1	121.185 (7)	119.387 (7)
C3—C4	1.50570 (15)	1.49970 (15)	*M*—O3—C3	120.885 (6)	119.415 (8)
H5*A*⋯O4^ii^	1.73078 (13)	1.73104 (15)	*M* ^i^—O4—C3	125.469 (7)	121.077 (8)
H5*B*⋯O3^iii^	1.83444 (12)	1.88261 (14)	*M*—O3⋯H5*B* ^iii^	116.392 (6)	115.460 (6)
			C3—O3⋯H5*B* ^iii^	122.433 (8)	124.867 (9)
			*M* ^i^—O4⋯H5*A* ^ii^	116.803 (6)	121.668 (7)
			C3—O4⋯H5*A* ^ii^	117.684 (9)	117.232 (10)

Here, symmetry codes: (i) 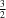 − *x*, 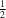 − *y*, 1−*z*; (ii) 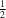 + *x*, 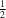 − *y*, 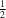 − *z*; (iii) 1 − *x*, 1 − *y*, 1 − *z*.

**Table 3:** AIM electron density properties at bond critical points # indicates e.s.d.’s less than 0.0005.

			BCP characteristics					
	Atom 1	Atom 2	*d*_12_ (Å)	ρ_BCP_ (e Å^−3^)	∇^2^ρ_BCP_ (e Å^−5^)	∊	*d*_1_ (Å)	*d*_2_ (Å)
(I)	Cu	O1	1.9559	0.468 (1)	12.462 (1)	0.12	0.9572	0.9987
(II)	Cr	O1	2.0135	0.430 (1)	12.181 (2)	0.28	0.9946	1.0188
(I)	Cu	O2^i^	1.9427	0.448 (1)	12.800 (2)	0.10	0.9536	0.9891
(II)	Cr	O2^i^	1.9999	0.440 (1)	12.456 (2)	0.29	0.9906	1.0093
(I)	Cu	O3	1.9852	0.435 (1)	11.363 (1)	0.17	0.9665	1.0188
(II)	Cr	O3	2.0298	0.412 (1)	11.695 (3)	0.27	0.9996	1.0302
(I)	Cu	O4^i^	1.9911	0.410 (1)	10.878 (1)	0.16	0.9729	1.0182
(II)	Cr	O4^i^	2.0293	0.412 (1)	11.624 (2)	0.28	1.0023	1.0269
(I)	Cu	O5	2.1487	0.295 (#)	7.026 (1)	0.10	1.0560	1.0928
(II)	Cr	O5	2.2581	0.252 (#)	5.690 (1)	0.07	1.1302	1.1279
(I)	Cu	Cu^i^	2.6111	0.057 (#)	1.648 (#)	0.11	1.3055	1.3056
(II)	Cr	Cr^i^	2.3478	0.167 (#)	4.944 (1)	0.01	1.1739	1.1739
(I)	O1	C1	1.2680	2.717 (8)	−33.38 (4)	0.13	0.7749	0.4930
(II)	O1	C1	1.2695	2.708 (9)	−33.24 (5)	0.07	0.7790	0.4905
(I)	O2i	C1	1.2615	2.646 (8)	−32.95 (5)	0.08	0.7935	0.4679
(II)	O2^i^	C1	1.2665	2.662 (9)	−32.85 (5)	0.09	0.8005	0.4661
(I)	O3	C3	1.2636	2.585 (8)	−30.94 (5)	0.11	0.8086	0.4550
(II)	O3	C3	1.2666	2.676 (9)	−34.18 (5)	0.11	0.7959	0.4710
(I)	O4^i^	C3	1.2662	2.698 (8)	−34.00 (4)	0.14	0.7846	0.4816
(II)	O4^i^	C3	1.2706	2.661 (9)	−34.04 (5)	0.10	0.7943	0.4763
(I)	C1	C2	1.5032	1.802 (5)	−12.67 (1)	0.10	0.7896	0.7136
(II)	C1	C2	1.4997	1.793 (6)	−12.03 (1)	0.12	0.7777	0.7220
(I)	C3	C4	1.5045	1.787 (5)	−11.92 (1)	0.10	0.7776	0.7269
(II)	C3	C4	1.4998	1.808 (6)	−12.77 (1)	0.18	0.7788	0.7210
(I)	O3	H5*B* ^ii^	1.8711	0.070 (#)	4.063 (3)	0.27	1.2714	0.5998
(II)	O3	H5*B* ^ii^	2.0252	0.050 (#)	2.383 (#)	0.96	1.3417	0.6835
(I)	O4	H5*A* ^iii^	1.7338	0.108 (3)	5.927 (4)	0.01	1.1996	0.5342
(II)	O4	H5*A* ^iii^	1.7413	0.074 (3)	5.966 (4)	0.07	1.2226	0.5187

Symmetry codes: (i) 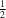 − *x*, 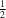 − *y*, − *z*; (ii) − *x*, − *y*, − *z*; (iii) 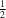 + *x*, 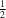 − *y*, 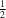 + *z*.

**Table 5:** Population of the *d*-orbitals on central atoms

Orbital	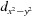	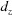	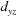	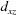	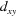	Σ
(I) (e^−^)	1.363 (4)	2.015 (4)	2.086 (4)	1.909 (4)	1.985 (4)	9.36
(II) (e^−^)	0.353 (5)	0.992 (5)	0.989 (5)	0.999 (5)	1.042 (5)	4.38

**Table 4:** AIM atomic charges and atomic volumes

	*M*	O1	O2	O3	O4	O5	C1	C2	C3	C4	H5*A*	H5*B*
Charge (e^−^)												
(I)	1.49	−0.96	−0.99	−1.02	−1.10	−1.23	1.29	−0.10	1.43	−0.12	0.59	0.59
(II)	1.55	−1.09	−1.06	−1.10	−1.13	−1.30	1.38	−0.01	1.41	0.02	0.67	0.67

V001 (Å^3^)												
(I)	8.32	15.61	15.98	15.77	16.18	18.70	6.09	11.07	5.90	11.35	1.94	1.91
(II)	14.17	16.19	18.33	16.43	18.39	19.48	6.07	11.03	5.92	10.57	1.61	1.56
