# Supplementary material for: Electronic structure of two isostructural ‘paddle-wheel’ complexes: a comparative study
Source: Acta Crystallogr B Struct Sci Cryst Eng Mater. 2018 Nov 19;74(Pt 6):681–92. doi: 10.1107/S2052520618013707 (PMC6289242; doi:10.1107/S2052520618013707)
Supplement: Supplementary file 2 [file b-74-00681-sup2.pdf]

Acta Cryst B
STRUCTURAL SCIENCE
CRYSTAL ENGINEERING
MATERIALS

Volume 74 (2018)

Supporting information for article:

Electronic structure of two isostructural `paddle-wheel' complexes: a comparative study

Peter Herich, Lukáš Bučinský, Martin Breza, Marián Gall, Marek Fronc, Václav Petříček and Jozef Kožíšek

Contents

a) Charge density studies details

b) Theoretical Calculations of $[Cu(DMSO)_6]^q$ chromophore

c) CSD search on $[CuO_6]$ chromophore (different .pdf file)

d) Appendix I. Comparison of multipole refinement for **(I)** using the non-averaged and merged data.

a) Charge density studies details

S1 **Data collection and crystal structure refinement details**

Data Collection. A suitable recrystallized sample of a tetrakis(μ_2-acetato)-diaqua-di-copper(II) (I) and tetrakis(μ_2-acetato)-diaqua-di-chromium(II) (II) crystals were used for the high-resolution X-ray diffraction experiments. The data were collected at 100.0(1) K on an Oxford Diffraction Kappa geometry GEMINI R diffractometer equipped with Rubi CCD area detector using graphite monochromated MoKα X-radiation (wavelength λ = 0.71073 Å) at 50 kV and 40 mA. Crystal-to-detector distance was of 60 mm. The diffraction data were collected with a huge and equally distributed redundancy at six different detector positions for (I) ($2\theta = \pm18.695^o, \pm60.0^o, -90.0^o$ and $+96.250^o$) and eight detector positions for (II) ($2\theta = -18.94^o, 19.02^o, \pm30.0^o, -48.97^o, 49.12^o, -72.0^o$ and 81.17^o where θ is the Bragg angle). The strategy was based on 198 omega runs for (I) and 70 omega runs for (II) with the frame width of 1.0^o. According to the θ-dependence of the diffracted intensities, the chosen exposure time was of 15, 60 and 80 seconds for A and of 20, 32, 90 and 180 seconds for (II), respectively. The maximum resolution reached at this experimental setting was of 1.297 Å^{-1} for (I) and of 1.220 Å^{-1} for (II). 405560 total number of reflections were collected in this long-term experiment for (I) and 153981 for (II), respectively. Integration of the diffracted intensities, the Lorentz-polarization and FACE-absorption correction were performed with CrysAlis PRO software (Rigaku OD, 2016). Average redundancy was of 31.9 with the R_{int} = 0.026 and $R(\sigma)$ = 0.005 for (I), as well as of 13.7 and R_{int} = 0.020 and $R(\sigma)$ = 0.008 for (II), respectively. After the data reduction, the .hkl files were treated with JANA2006 to obtain xd.hkl files, with the direction cosines included, for the multipolar refinement. Details of the X-ray diffraction experiment conditions and the crystallographic data for (I) and (II) are given in Table 1.

S2. Multipole model refinement details.

1. Both crystal structures are isostructural with approximately the same lattice parameters (Table 1). Starting atom coordinates and atom displacement parameters (ADP's) were taken from a routine SHELXL (Sheldrick, 2015) refinement and all other refinements were carried out on F^2 using the XD (Volkov, *et al.*, 2015) suite of programs. As the equivalent data are collected with a different value of TBAR (distance of primary and diffracted beam through the crystal), all non-averaged data were used in the refinements. Details on the XD-refinements are
2.
3. A complete atom-centered multipole refinement was carried out with the non-spherical atomic electron density given by the formula (Hansen & Coppens, 1978):

4. $\rho_{at}(\mathbf{r}) = P_c\,\rho_{core}(\mathbf{r}) + P_v\,\kappa^3\,\rho_{valence}(\kappa\mathbf{r}) + \quad \kappa'^3\,R_l(\kappa'\mathbf{r}) \quad P_{lm\pm}d_{lm\pm}(\theta,\varphi)$ (1)

5. H atoms were treated with one bond-directed dipole (l = 1), other atoms were refined up to octupoles, for Cu and Cr atom the hexadecapole level (l_{max} = 4) was used. The local coordinate systems to define multipoles were used as follows:
6. For non-hydrogen atoms: *x-axis* - direction to the closest atom, *y-axis* - perpendicular to the *x-axis* and oriented towards the second closest atom;
7. For Cu and Cr atom *z-axis* – direction to O(5) [for atom labels see Fig. 1], y-axis - perpendicular to the *x-axis* and oriented towards O(1).
8. For hydrogen atoms: *z-axis* - direction to the bonding carbon atom and *x-axis* - perpendicular to the *z-axis*. The same type of hydrogen atoms [sp^3 hybridization: H(2A), H(2B), H(2C), H(4A), H(4B), H(4C)] were constrained to have identical multipole expansions.

Figure S1a, S1b. Fractal analysis of the residual density for **(I)** and **(II)**.

Figure S2a, S2b. Normal probability distribution plot for **(I) and (II)**.

Figure S3a, S3b. Variation of scale factor for **(I) and (II)**.

Figure S4. Static electron deformation densities of **(II)** in the plane Cu-Cu*-O1.

Figure S5. Laplacian distribution $L(\mathbf{r}) \approx \nabla^2 \rho(\mathbf{r})$ of **(II)** in the plane Cu-Cu*-O3.

Figure S6. Static electron deformation densities of **(II)** in the plane Cr-Cr*-O1*.

Figure S7. Laplacian distribution $L(\mathbf{r}) \approx \nabla^2 \rho(\mathbf{r})$ of **(II)** in the plane Cr-Cr*-O1*.

Figure S8. Static electron deformation densities of **(II)** in the plane Cu-O1-O3.

Figure S9. Laplacian distribution $L(\mathbf{r}) \approx \nabla^2 \rho(\mathbf{r})$ of **(II)** in the plane Cu-O1-O3.

Figure S10. Static electron deformation densities of **(II)** in the plane Cr-O1-O3.

Figure S11. Laplacian distribution $L(\mathbf{r}) \approx \nabla^2 \rho(\mathbf{r})$ of **(II)** in the plane Cr-O1-O3.

Figure S12. Static electron deformation densities of **(II)** in the plane Cu-O5-O3.

Figure S13. Laplacian distribution $L(\mathbf{r}) \approx \nabla^2 \rho(\mathbf{r})$ of **(II)** in the plane Cu-O5-O3.

Figure S14. Static electron deformation densities of **(II)** in the plane Cr-O5-O3.

Figure S15. Laplacian distribution $L(\mathbf{r}) \approx \nabla^2 \rho(\mathbf{r})$ of **(II)** in the plane Cr-O5-O3.

Figure S16. Gradient field trajectory plot of electrostatic potential in the plane Cu-O3-O4

Figure S17. Gradient field trajectory plot of electrostatic potential in the plane Cr-O3-O4

Figure S18a-f. Local Source function of **(I)** and **(II)**.

Figure S19a-c. Atomic graph of the Cu atom in **(I)**

b) Theoretical Calculations of $[Cu(DMSO)_6]^q$ chromophore

Table S21. Theoretical Calculations of $[Cu(DMSO)_6]^q$ chromophore

c) CSD search on $[CuO_6]$ chromophore

S20. CSD_232str.pdf

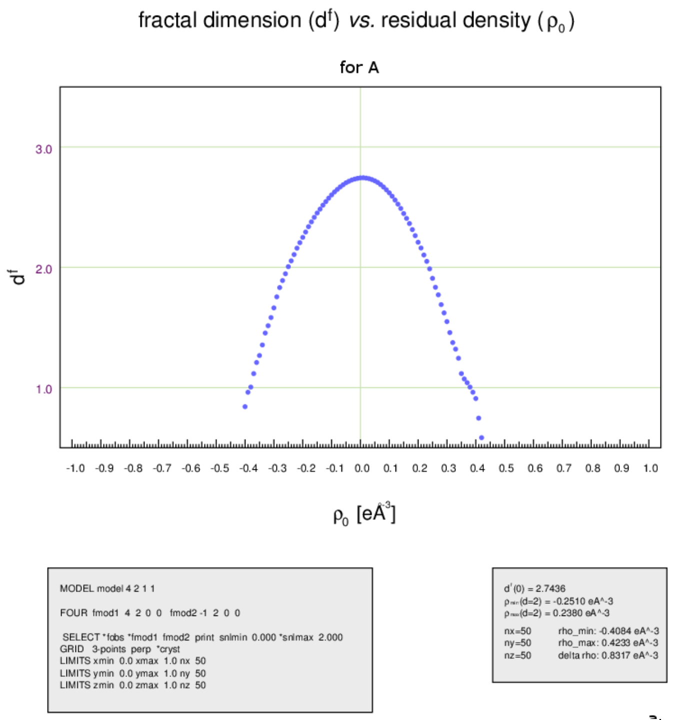

Figure S1a. Fractal analysis of the residual density (Meindl, & Henn, 2008) for **(I)**.

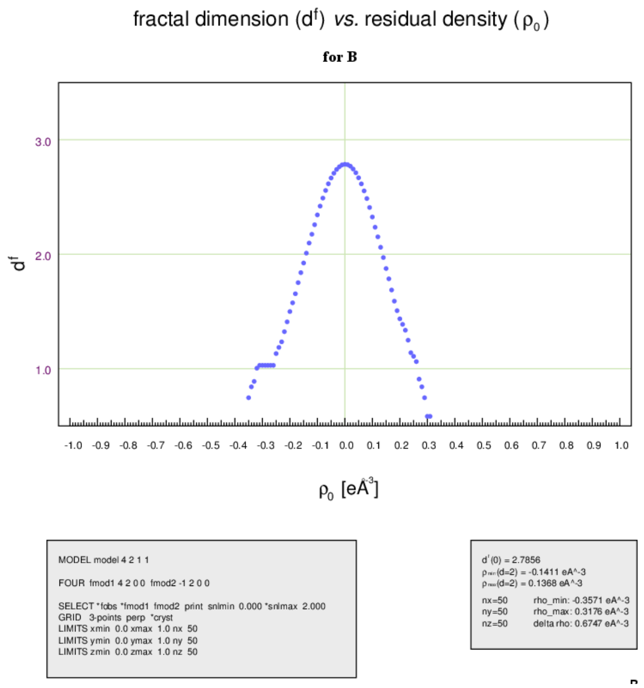

Figure S1b. Fractal analysis of the residual density (Meindl, & Henn, 2008) for **(II)**.

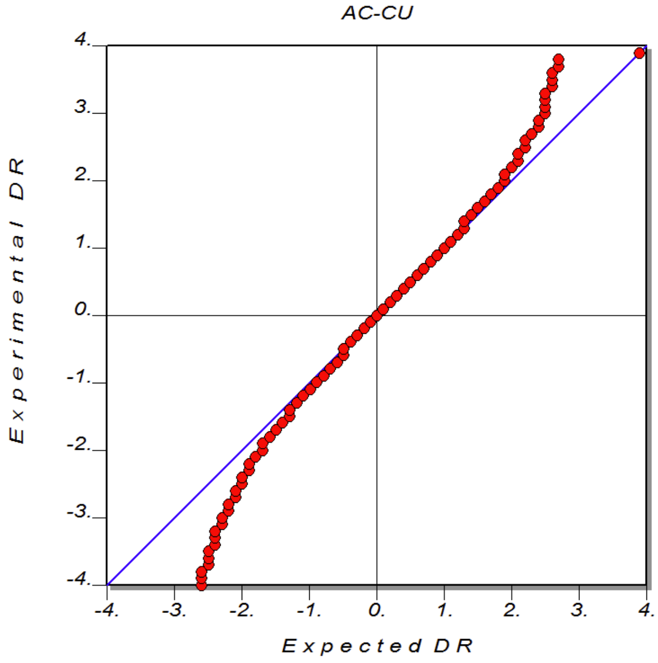

Figure S2a. Normal probability distribution plot (Abrahams & Keve, 1971) for **(I)**.

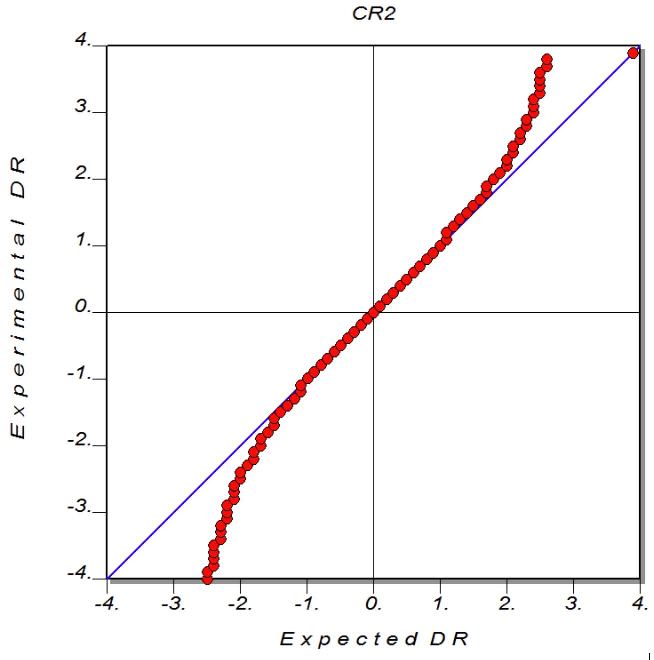

Figure S2b. Normal probability distribution plot (Abrahams & Keve, 1971) for **(II)**.

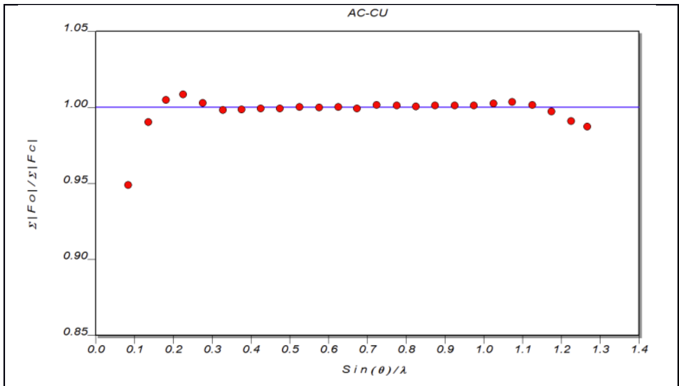

Figure S3a. Variation of scale factor[3] for **(I)**.

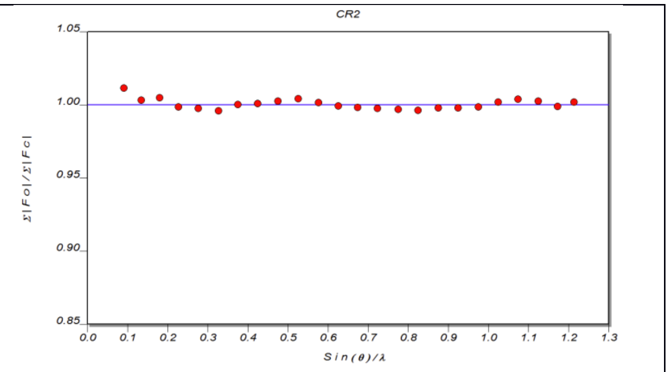

Figure S3b. Variation of scale factor[3] for **(II)**.

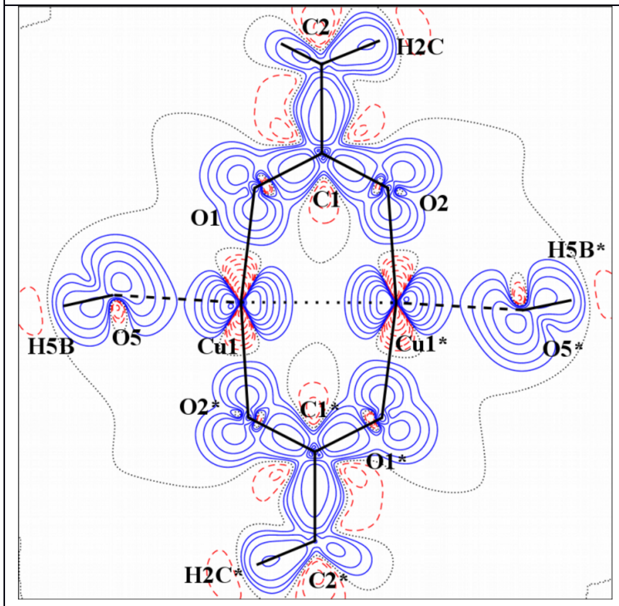

Figure S4. Static electron deformation density of **(II)** in the plane Cu-Cu*-O1. Contour spacing as in Fig. 2b. Symmetry code as in Fig. 3.

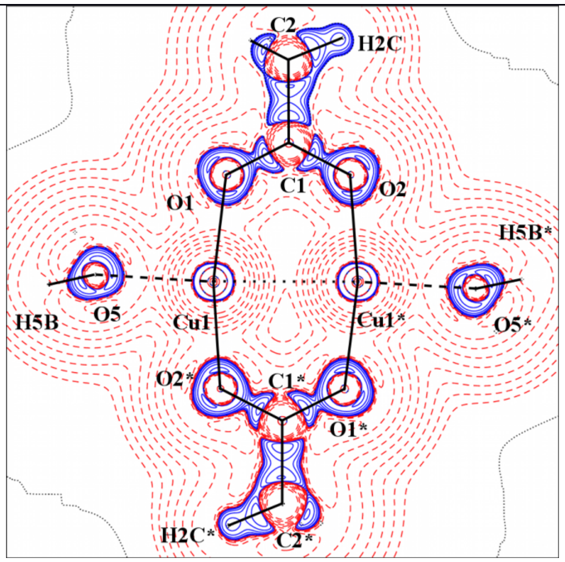

Figure S5. Laplacian distribution $L(\mathbf{r}) \approx \nabla^2 \rho(\mathbf{r})$ of **(II)** in the plane Cu-Cu*-O1. Contour spacing as in Fig. 2c and the symmetry code as in Fig. 4.

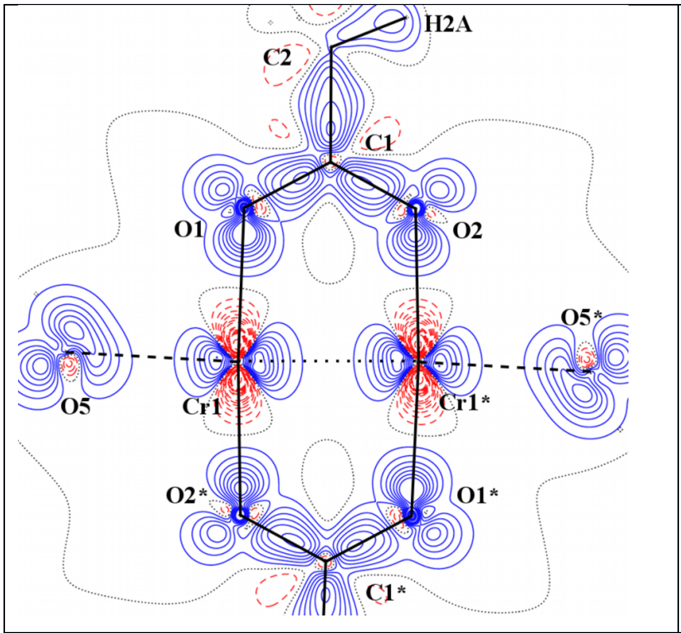

Figure S6. Static electron deformation density of **(II)** in the plane Cr-Cr*-O1*. Contour spacing as in Fig. 2b. Symmetry code as in Fig. 3.

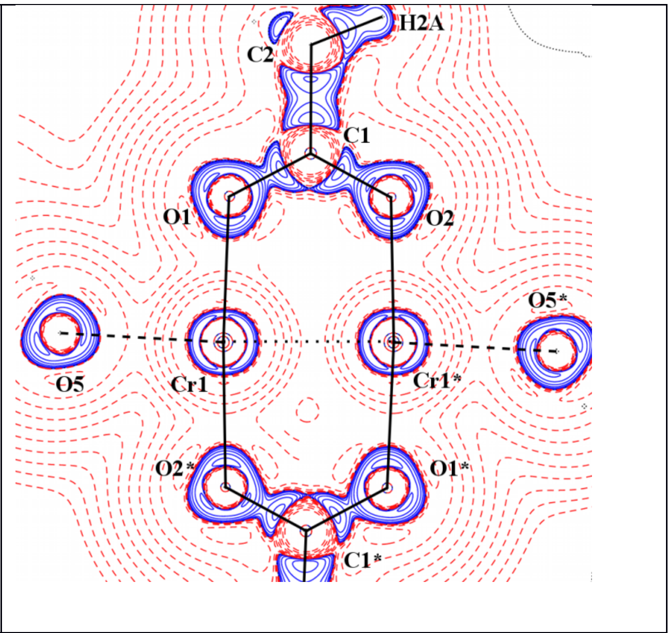

Figure S7. Laplacian distribution L(**r**) ≈ ∇^2 ρ(**r**) of **(II)** in the plane Cr-Cr*-O1*. Contour spacing as in Fig. 2c and the symmetry code as in Fig. 4.

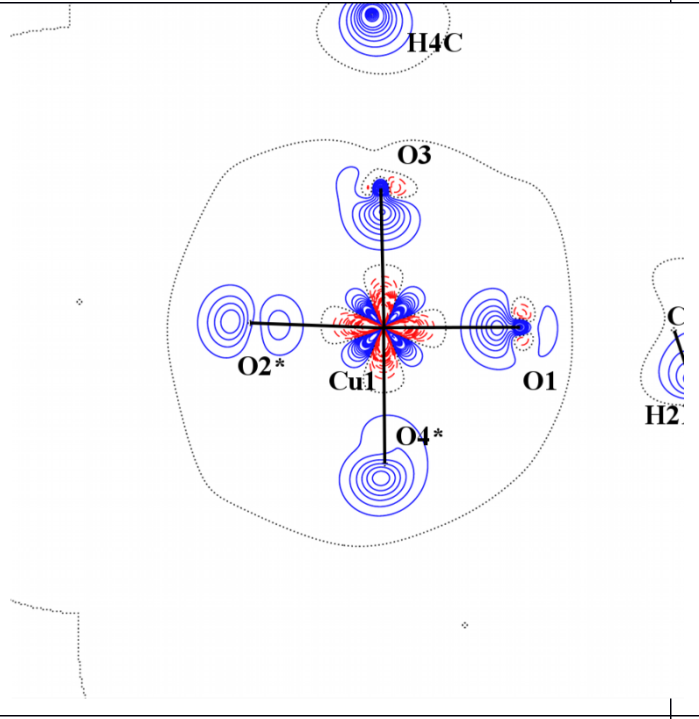

Figure S8. Static electron deformation density of **(II)** in the plane Cu-O1-O3. Contour spacing as in Fig. 2b. Symmetry code as in Fig. 3.

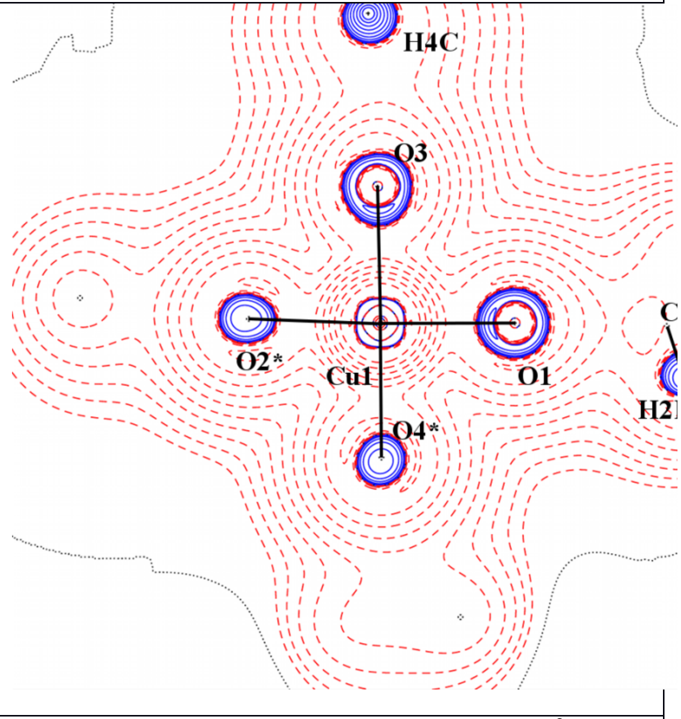

Figure S9. Laplacian distribution L(**r**) ≈ ∇^2 ρ(**r**) of **(II)** in the plane Cu-O1-O3. Contour spacing as in Fig. 2c and the symmetry code as in Fig. 4.

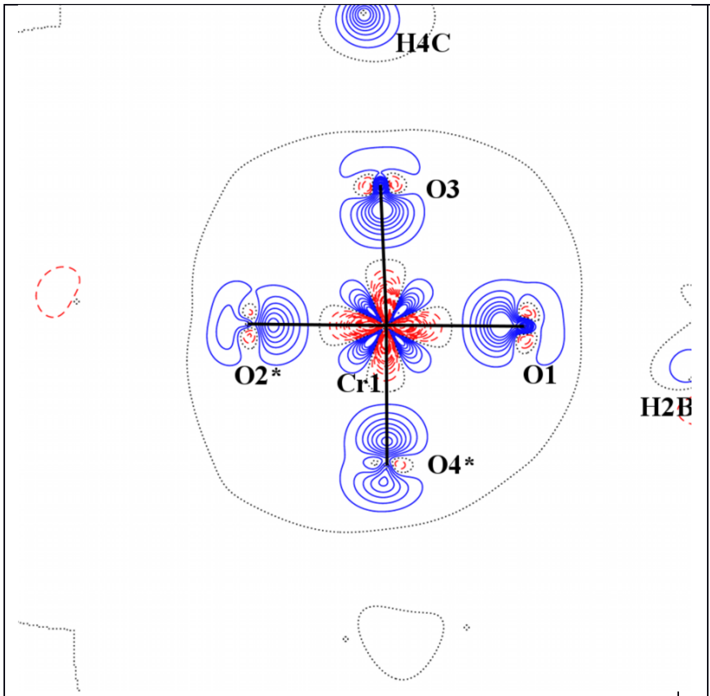

Figure S10. Static electron deformation densitiy of **(II)** in the plane Cr-O1-O3. Contour spacing as in Fig. 2b. Symmetry code as in Fig. 3.

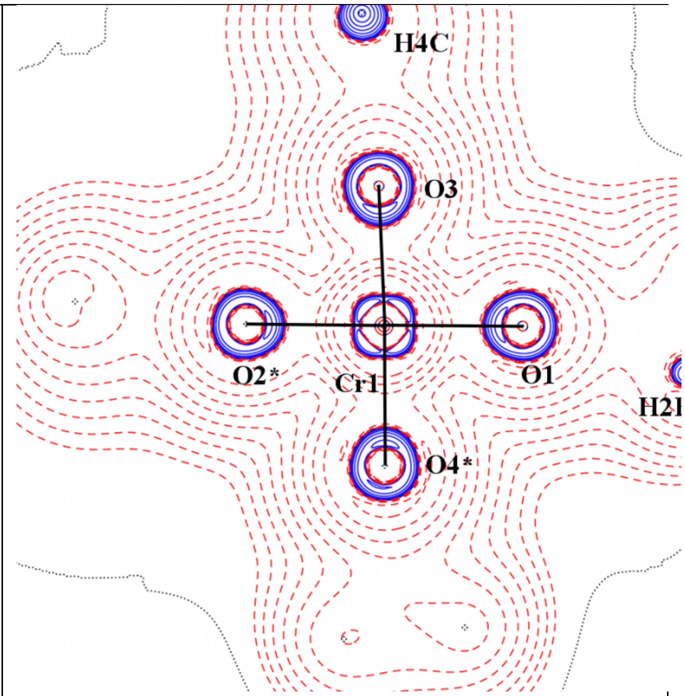

Figure S11. Laplacian distribution L(**r**) ≈ ∇^2 ρ(**r**) of **(II)** in the plane Cr-O1-O3. Contour spacing as in Fig. 2c and the symmetry code as in Fig. 4.

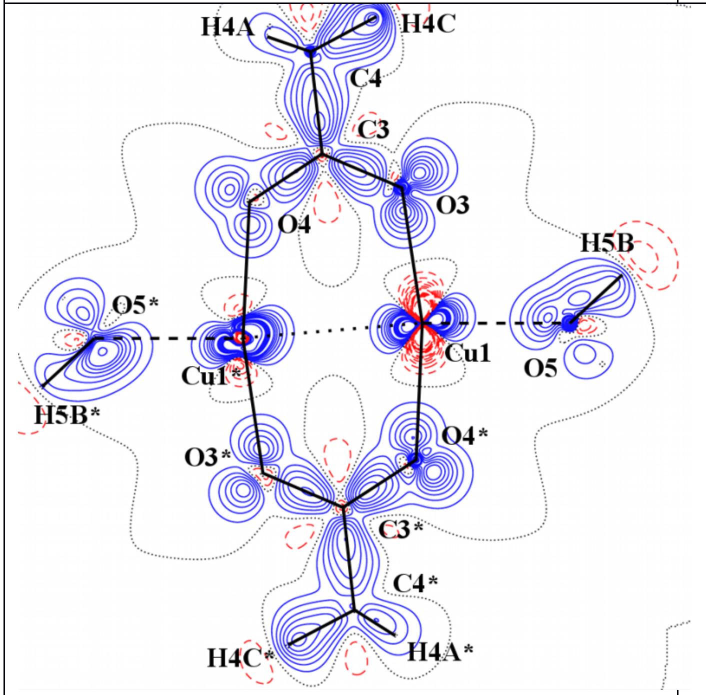

Figure S12. Static electron deformation density of **(II)** in the plane Cu-O5-O3. Contour spacing as in Fig. 2b. Symmetry code as in Fig. 3.

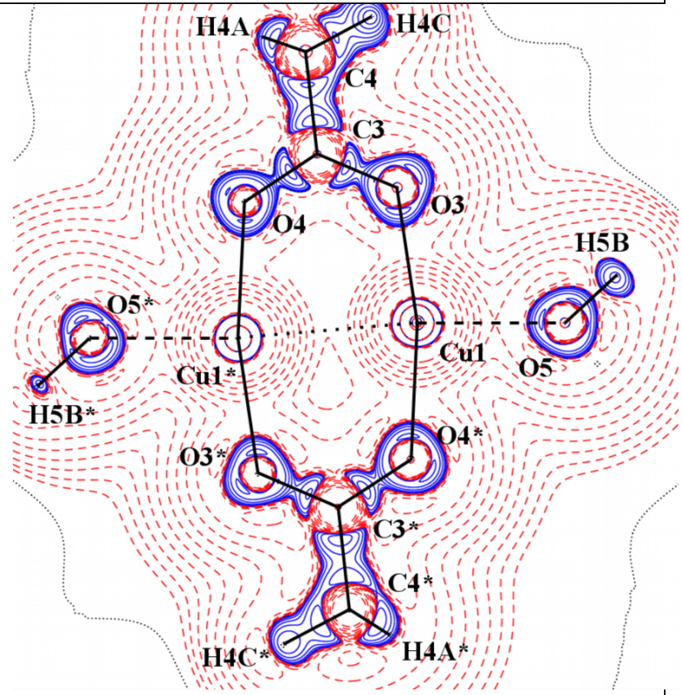

Figure S13. Laplacian distribution L(**r**) ≈ ∇^2 ρ(**r**) of **(II)** in the plane Cu-O5-O3. Contour spacing as in Fig. 2c and the symmetry code as in Fig. 4.

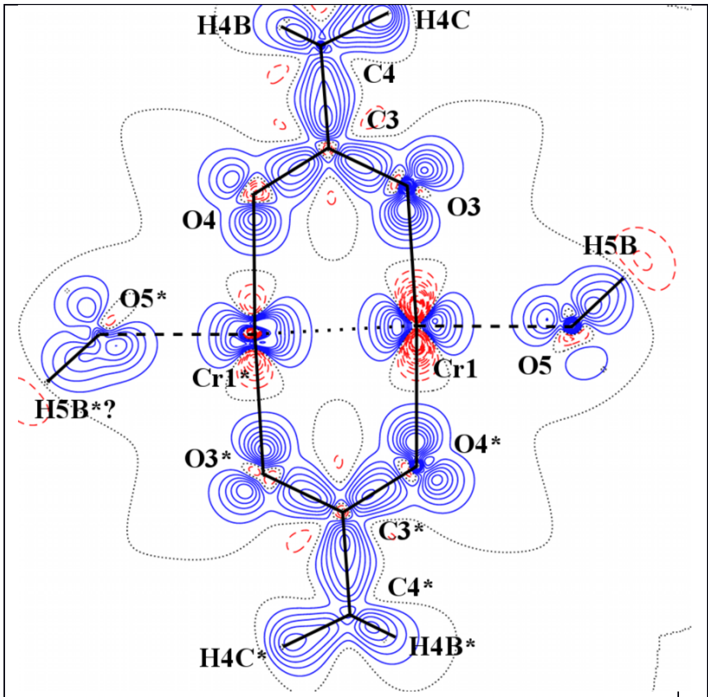

Figure S14. Static electron deformation density of **(II)** in the plane Cr-O5-O3. Contour spacing as in Fig. 2b. Symmetry code as in Fig. 3.

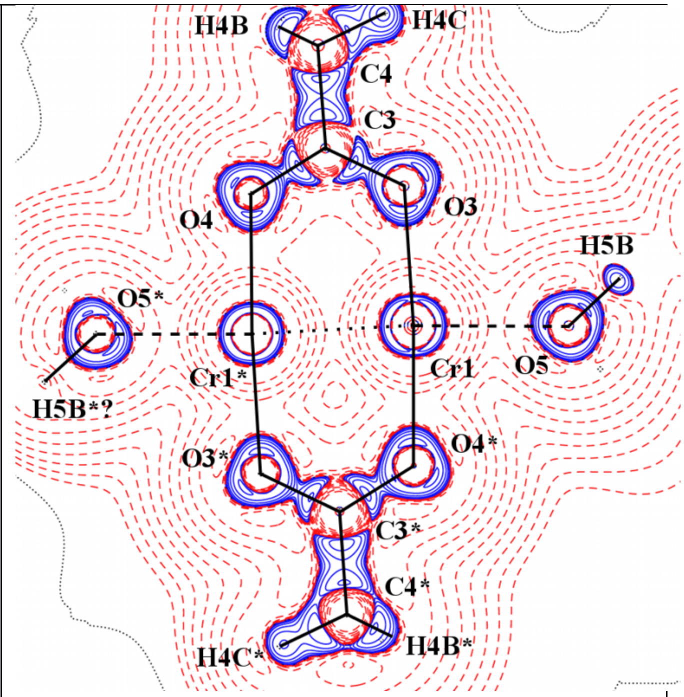

Figure S15. Laplacian distribution L(**r**) ≈ ∇^2 ρ(**r**) of **(II)** in the plane Cr-O5-O3. Contour spacing as in Fig. 2c and the symmetry code as in Fig. 4.

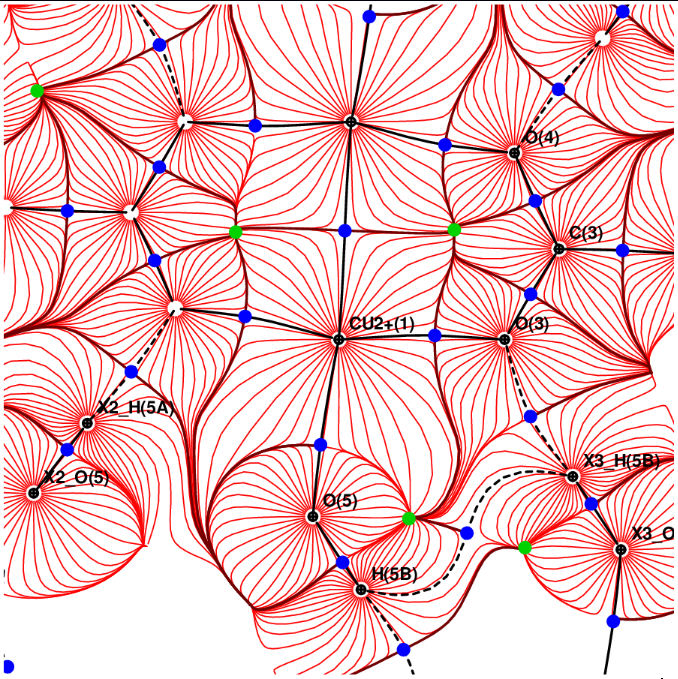

Figure S16. Gradient field trajectory plot of electrostatic potential in the plane Cu-O3-O4

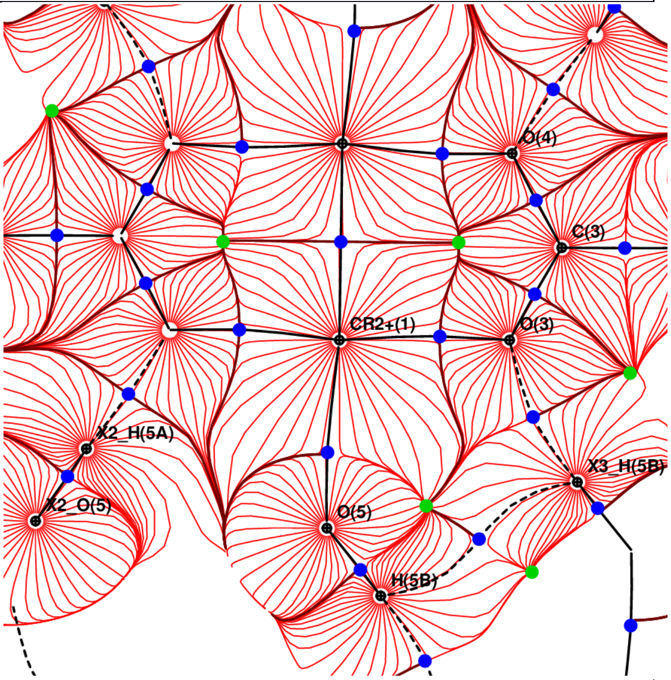

Figure S17. Gradient field trajectory plot of electrostatic potential in the plane Cr-O3-O4

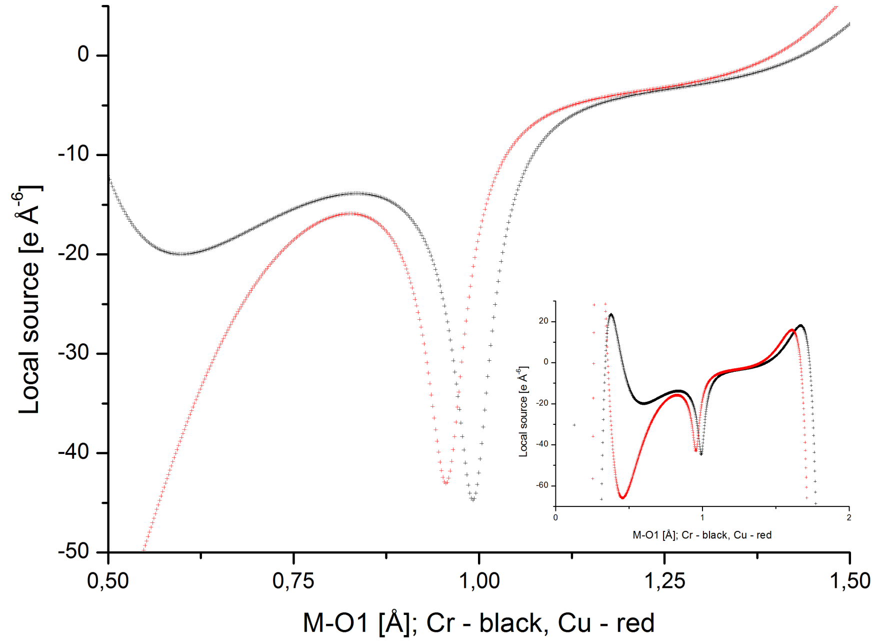

Figure S18a. Local Source function of **(I)** and **(II)**.

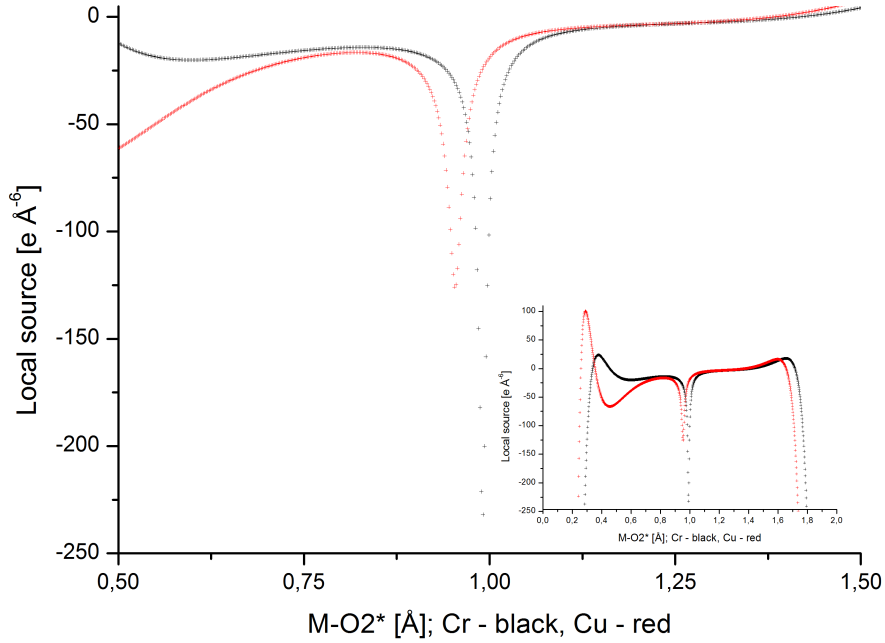

Figure S18b. Local Source function of **(I)** and **(II)**.

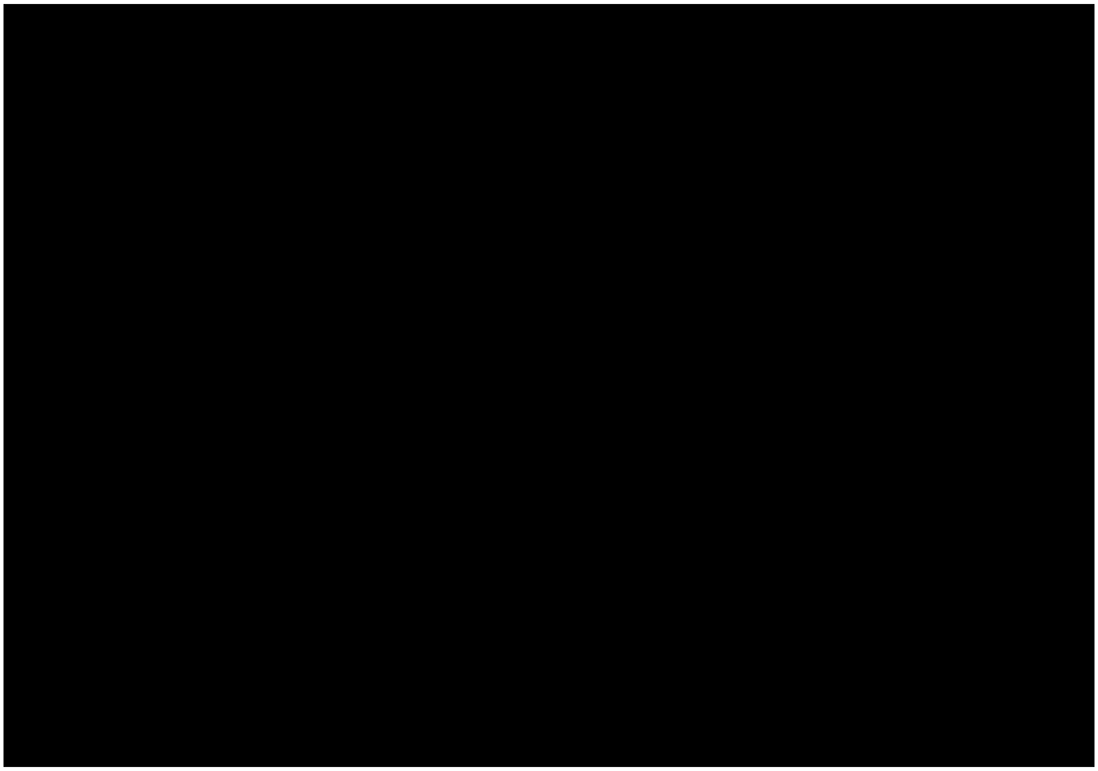

Figure S18c. Local Source function of **(I) and (II)**.

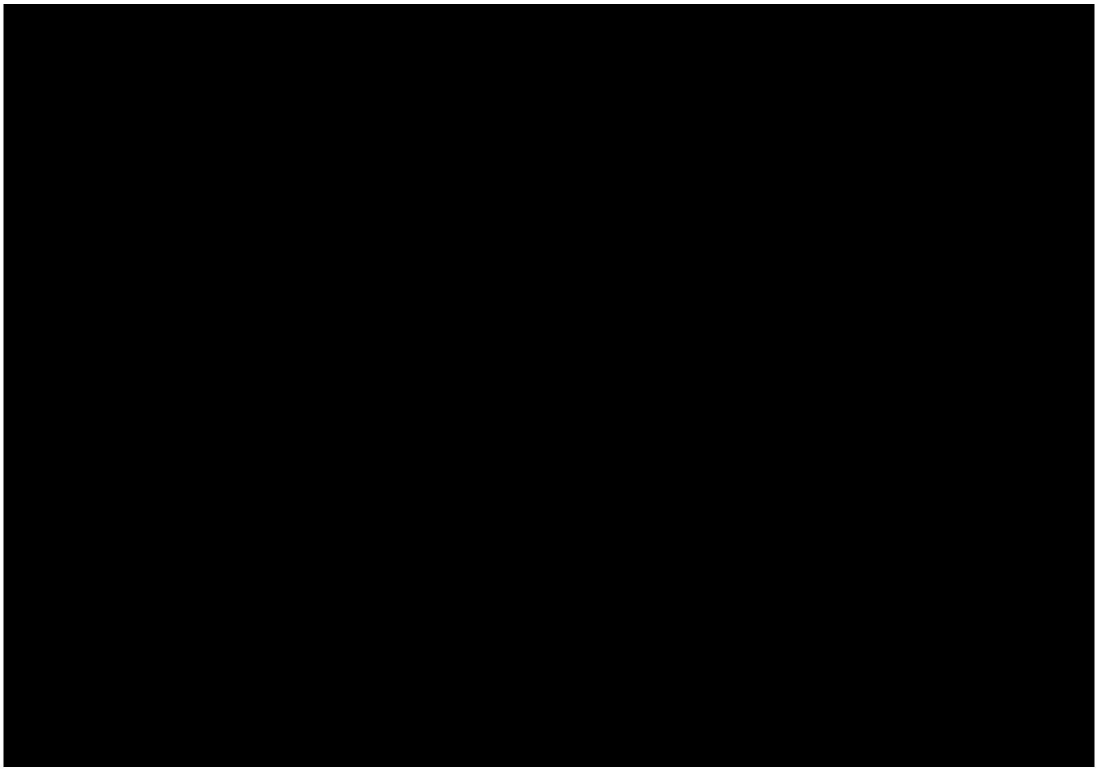

Figure S18d. Local Source function of **(I) and (II)**.

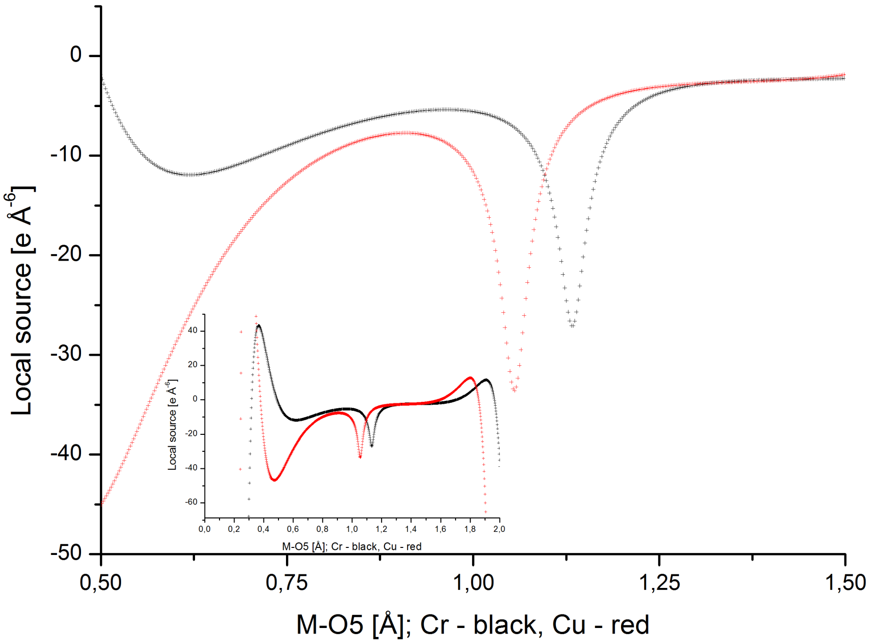

Figure S18e. Local Source function of **(I) and (II)**.

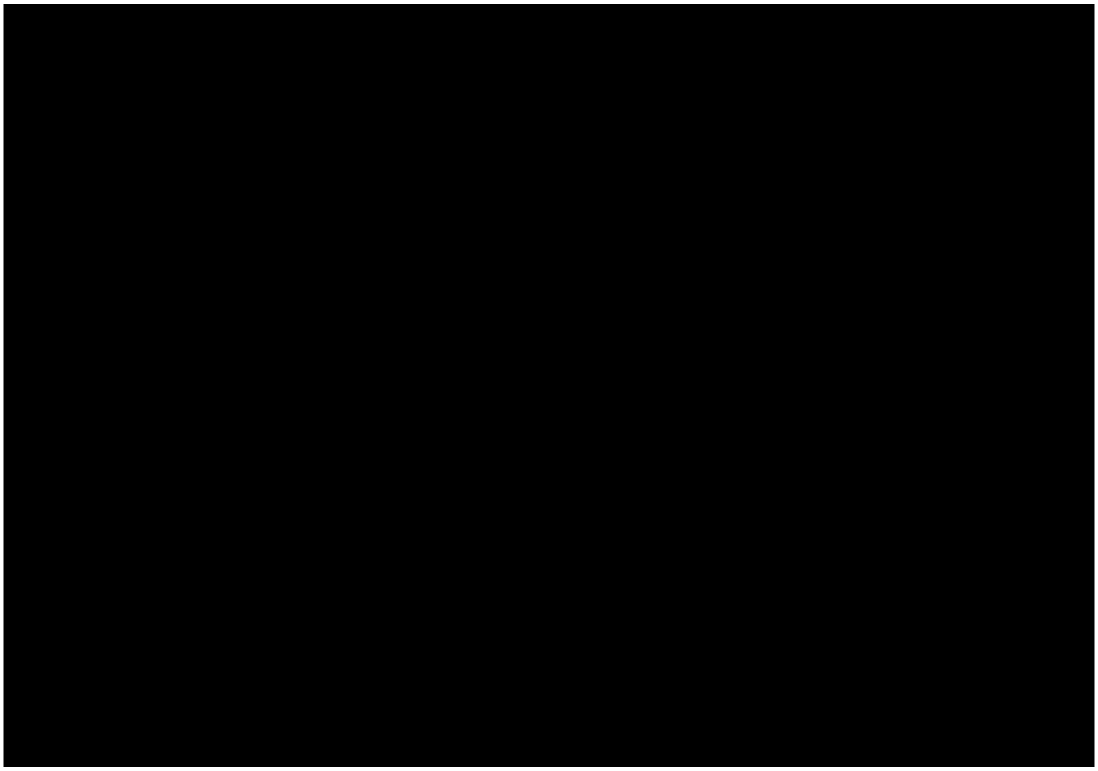

Figure S18f. Local Source function of **(I) and (II)**.

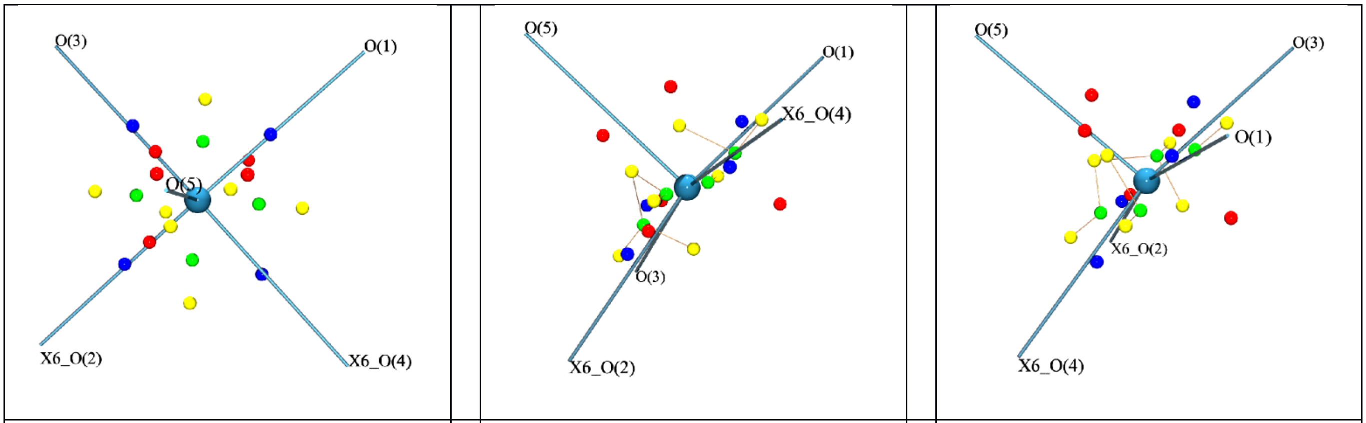

a) b) c)

Figure S19. Atomic graph of the Cu atom in **A** showing the critical points in $L(\mathbf{r}) = \nabla^2\rho_{BCP}$ in the VSCC. Color coding is (3,-3) green, (3,-1) yellow, (3,+1) red, (3,+3) blue.

b) S21. Theoretical Calculations of $[Cu(DMSO)_6]^q$ chromophore

The geometry of $[Cu(DMSO)_6]^q$, q = 1 or 2, in the lowest ground spin state was optimized at the MP2 level of theory (Head-Gordon *et al.*, 1988; Saebø & Almlöf, 1989; Frisch *et al.*, 1990; Head-Gordon, M. & Head-Gordon, T., 1994) starting from the experimental X-ray structure of $[Cu(DMSO)_6]I_4$ [5] using the Gaussian09 program Package (Frisch *et al.*, 2009). Standard 6-311G* basis sets from the Gaussian09 library (Frisch *et al.*, 2009) were used for copper and sulfur atoms and the 6-31G* ones for the remaining atoms. The stability of the obtained structures has been tested by vibrational analysis (no imaginary vibrations).

Results

Garzón-Tovar *et al.* (Garzón-Tovar *et al.,* 2013) suppose the existence of $[Cu(DMSO)_6]^{2+}$ complex cations of S_6 symmetry in $[Cu(DMSO)_6]I_4$ crystals. MP2 calculations indicate its subgroup C_i symmetry of these cations (Table S21). This discrepancy may be explained by the Jahn-Teller effect of the high-symmetric S_6 structure of this cation in degenerate ^2E_g electron ground state which is unstable. Consequently, it is stabilized by a symmetry descent to its C_i subgroup and the degenerate electron state is split into ^2A_g non-degenerate ones. An analogous $[Cu(DMSO)_6]^+$ monocation of S_6 symmetry is in a non-degenerate ^2A_g ground electron state and so its high symmetry is preserved (see Table S21). Experimentally observed S_6 symmetry of the $[Cu(DMSO)_6]^{2+}$ complex cation may be explained by a dynamical Jahn-Teller effect where its three possible stable C_i structures are separated by relatively small energy barriers in comparison with the vibrational energy at the temperature of the X-ray experiment and so the transitions between them are enabled (i.e. shortening/elongation of individual Cu-O bonds). Consequently an averaged $[Cu(DMSO)_6]^{2+}$ structure of S_6 symmetry is observed.

An alternative explanation of a high-symmetric S_6 structure of the complex cations in $[Cu(DMSO)_6]I_4$ might be based on an electron density transfer from iodine anions to $[Cu(DMSO)_6]^{2+}$ cations resulting in the $[Cu(DMSO)_6]^+$ ones formation which do not undergo the above mentioned Jahn-Teller effect. This idea is supported by relatively small standard deviations of Cu-O bond lengths in the experimental structure (Table S21). Too long Cu-O bonds in the MP2 optimized $[Cu(DMSO)_6]^+$ structure in vacuum in comparison with the X-ray data may be explained by missing solid state effects.

Additional magnetic measurements are desirable to distinguish between both above mentioned explanations.

Table S21. Symmetry groups, electron states, spin squares (<S^2>), MP2 energies (E_{MP2}), free energies at 298 K (G_{298}), Cu-O bond lengths and O-Cu-O bond angles of the systems under study.

Method	MP2 calculations		X-ray [5]
System	$[Cu(DMSO)_6]^{2+}$	$[Cu(DMSO)_6]^{+}$	$[Cu(DMSO)_6]I_4$
Symmetry	C_i (-1)	S_6 (-3)	S_6 (-3)
Electron state	^2A_g	^1A_g	-
<S^2>	0.752 (0.750)[a)]	0	
E_{MP2} [hartree]	-4951.93689 (-4951.93694)[a)]	-4952.21501	-
G_{298} [hartree]	-4951.51790	-4951.80112	-
Bond lengths [Å]			
Cu-O_{ax}	2.261	2.279	2.102(2)
Cu-O_{eq1}	2.031	2.279	2.102(2)
Cu-O_{eq2}	1.978	2.279	2.102(2)
Bond angles [°]			
O_{ax}-Cu-O_{eq1}	85.81	87.07	87.22(9)
O_{ax}-Cu-O_{eq2}	89.47	87.07	87.22(9)
O_{eq1}-Cu-O_{eq2}	89.67	87.07	87.22(9)

Remarks:
[a)] projected after correction to spin contamination

References

Abrahams, S. C., Keve, E. T. (1971). *Acta Cryst.* A**27***,* 157-165.

Farrugia, L. J., *J.* (2012). *Appl. Cryst.* **45**, 849-854.

Frisch, M. J., Head-Gordon, M., Pople, J. A. (1990). *Chem. Phys. Lett.* **166**, 275-280.

Frisch, M. J., Head-Gordon, M., Pople, J. A. (1990). *Chem. Phys. Lett.* **166**, 281-289.

Frisch, M. J., *et al.,* (2009). Gaussian 09, Revision D.01; Gaussian, Inc., Wallingford, CT, 2013 (available via the Internet at: www.gaussian.com).

Head-Gordon, M., Pople, J. A., Frisch, M. J. (1988). *Chem. Phys. Lett. 153*, 503-506.

Head-Gordon, M., Head-Gordon, T. (1994). *Chem. Phys. Lett.* **220**, 122-128.

Meindl, K., Henn, J. (2008). *Acta Cryst.* A**64***,* 404-418.

Saebø, S., Almlöf, J. (1989). *Chem. Phys. Lett. 154*, 83-89

c) S20. CSD search on $[CuO_6]$ chromophore (different .pdf file)

d) Appendix I. Comparison of results obtained from averaged and non-averaged data (different .pdf)

Table SA1. Comparison of multipole refinement for **(I)** from non-averaged and merged data.

Figures SA2. Residual densities in the plane defined by the atoms: *atom1 – atom2 – atom3*.

Figures SA3. Error analysis for **(I)**.

Figures SA4 (see on the next pages). Static electron deformation densities of **(I)** in the plane defined by the atoms O(2)*,O(1), O(3).
