# Supplementary material for: Electronic structure of two isostructural ‘paddle-wheel’ complexes: a comparative study
Source: Acta Crystallogr B Struct Sci Cryst Eng Mater. 2018 Nov 19;74(Pt 6):681–92. doi: 10.1107/S2052520618013707 (PMC6289242; doi:10.1107/S2052520618013707)
Supplement: Supplementary file 3 [file b-74-00681-sup3.pdf]

Search Overview

Search: search2

Date/Time done: Wed Jul 26 09:11:25 2017

Database(s): CSD version 5.38 updates (Nov 2016)
CSD version 5.38 (November 2016)
CSD version 5.38 (November 2016)
CSD version 5.38 updates (Feb 2017)
CSD version 5.38 updates (May 2017)

Restriction Info: No refcode restrictions applied

Filters: None

Percentage Completed: 100%

Number of Hits: 232

Summary of queries used. Search found structures that:

match

Query 1 **Query 2**

do not match

Query 3

Query 1

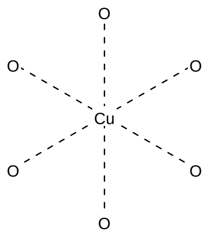

Query 2

R-factor **< 3 %**
Powder structures **exclude**

Query 3

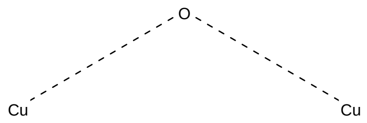

Search: search2 (Wed Jul 26 09:11:25 2017): Hit 1

UTAKIL

Reference: M.S.Henriques, D.I.Gorbunov, A.N.Ponomaryov, A.Saneei, M.Pourayoubi, M.Dusek, S.Zvyagin, M.Uhlarz, J.Wosnitza (2016) *Polyhedron* ,**118,**154

Formula: C_{30} H_{60} Cu_1 N_8 O_8 P_2

Compound Name: bis(nitrato-O,O')-bis(tripiperidinophosphine oxide-O)-copper(ii)

Space Group:	P21/n	***Cell:***	***a***	8.883(0)	***b***	17.136(0)	***c***	12.598(0)
Space Group No.:	14	***(Å,°)***	α	90.00	β	103.13(0)	γ	90.00

R-Factor (%): 2.52 ***Temperature(K):*** 120 ***Density(g/cm^3):*** 1.398

Parameters

Fragment 1	
**DIST1 (D)**	1.980
**DIST2 (D)**	1.980
**DIST3 (D)**	1.920
**DIST4 (D)**	1.920
**DIST5 (D)**	2.494
**DIST6 (D)**	2.494

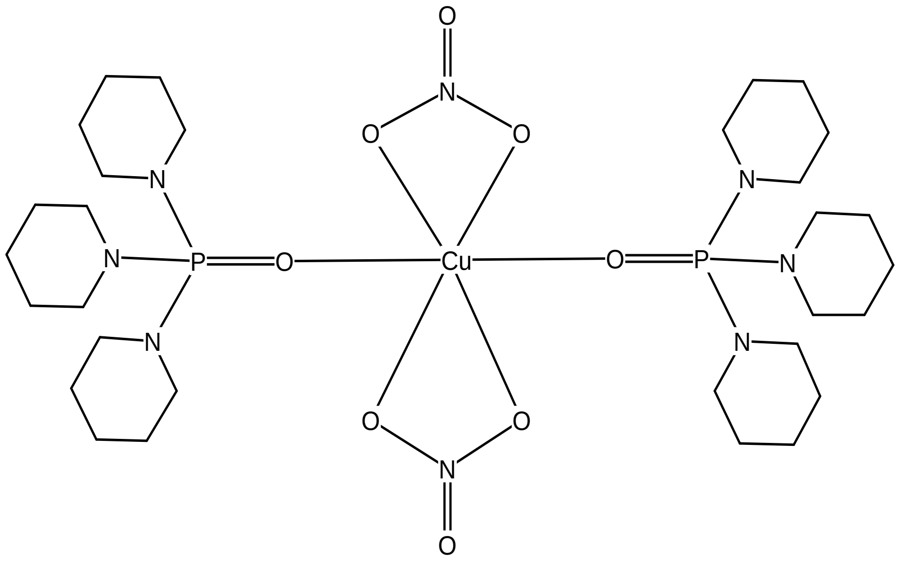

Search: search2 (Wed Jul 26 09:11:25 2017): Hit 2

ABACUF01

Reference: R.Baggio, D.Stoilova, G.Polla, G.Leyva, M.T.Garland
(2004) *J.Mol.Struct.* ,**697,**173

Formula: $(C_6\ H_{14}\ Ba_2\ Cu_1\ O_{16})n$

Compound Name: catena-(bis((μ_4-Formato)-(μ_3-formato)-(μ_2-formato-O,O)-bis(μ_2-aqua)-barium)-copper)

Space Group:	P-1	***Cell:***	***a***	8.817(0)	***b***	7.128(0)	***c***	6.928(0)
Space Group No.:	2	***(Å,°)***	α	98.00(0)	β	108.94(0)	γ	82.55(0)

R-Factor (%): 2.23 ***Temperature(K):*** 293 ***Density(g/cm^3):*** 2.782

Parameters

Fragment 1

**DIST1 (D)**	2.015
**DIST2 (D)**	1.972
**DIST3 (D)**	2.015
**DIST4 (D)**	1.972
**DIST5 (D)**	2.330
**DIST6 (D)**	2.330

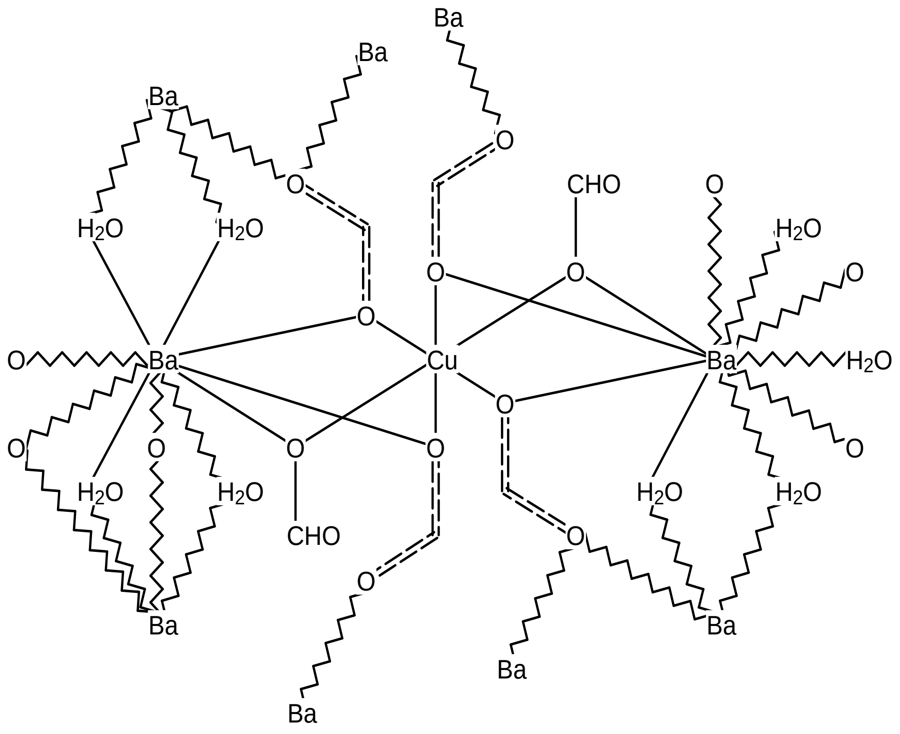

Search: search2 (Wed Jul 26 09:11:25 2017): Hit 3

ACUFUR01

Reference:	M.Yamanaka, H.Uekusa, S.Ohba, Y.Saito, S.Iwata, M.Kato, T.Tokii, Y.Muto, O.W.Steward (1991) *Acta Crystallogr.,Sect.B:Struct.Sci.* ,**47,**344
Formula:	$(C_4\ H_{12}\ Cu_2\ O_{12})n,4n(C_1\ H_4\ N_2\ O_1)$
Compound Name:	catena(Diaqua-bis((μ_2-formato-O,O')-copper(ii) (urea)))

Space Group:	P21/c	***Cell:***	***a*** 8.158(0)	***b*** 8.368(0)	***c*** 8.088(5)
Space Group No.:	14	***(Å,°)***	α 90.00	β 95.81(0)	γ 90.00
R-Factor (%):	2.40	***Temperature(K):*** 120		***Density(g/cm³):*** 1.872	

Parameters

Fragment 1

**DIST1 (D)**	1.985
**DIST2 (D)**	1.989
**DIST3 (D)**	1.985
**DIST4 (D)**	1.989
**DIST5 (D)**	2.408
**DIST6 (D)**	2.408

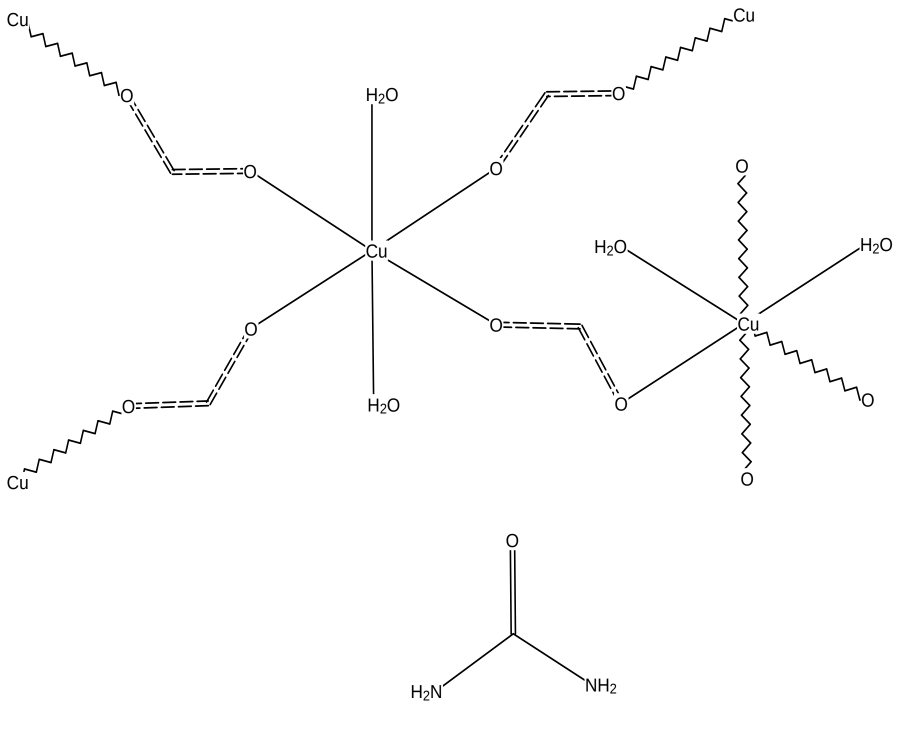

Search: search2 (Wed Jul 26 09:11:25 2017): Hit 4

AGAMAY

Reference:	M.S.Zavakhina, D.G.Samsonenko, D.N.Dybtsev, M.P.Yutkin, A.V.Virovets, V.P.Fedin (2013) *Izv.Akad.Nauk SSSR,Ser.Khim.(Russ.)(Russ.Chem.Bull.)* ,716
Formula:	$(C_6 H_{12} Cu_1 O_{10})n,3n(H_2 O_1)$
Compound Name:	catena-((μ_2-D-Glucarato)-diaqua-copper trihydrate)

Space Group:	P1	***Cell:***	***a***	6.636(0)	***b***	7.154(0)	***c***	7.408(0)
Space Group No.:	1	***(Å,°)***	α	100.12(0)	β	103.45(0)	γ	108.06(0)

R-Factor (%): 1.91 ***Temperature(K):*** 150 ***Density(g/cm³):*** 1.917

Parameters

Fragment 1

**DIST1 (D)**	1.981
**DIST2 (D)**	1.925
**DIST3 (D)**	1.990
**DIST4 (D)**	1.929
**DIST5 (D)**	2.303
**DIST6 (D)**	2.548

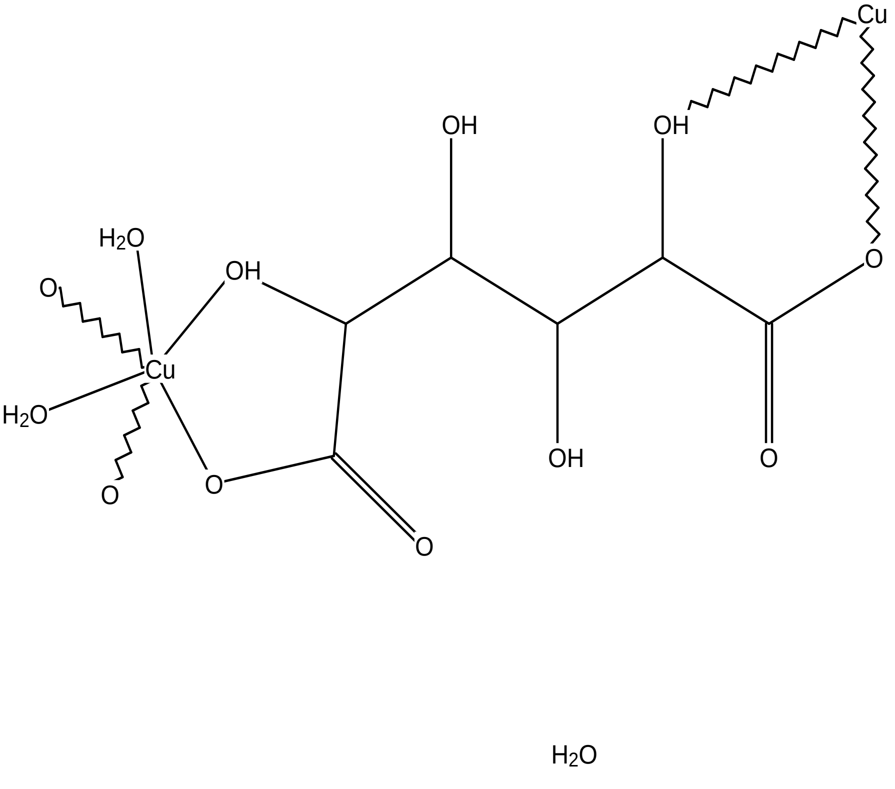

Search: search2 (Wed Jul 26 09:11:25 2017): Hit 5

AMATUF

Reference: T.J.Bednarchuk, V.Kinzhybalo, O.Bednarchuk, A.Pietraszko (2016) *J.Mol.Struct.* ,**1120,**138

Formula: $H_{12}\ Cu_1\ O_6\ ^{2+}$,2($C_5\ H_7\ N_2\ ^{1+}$),2($O_4\ S_1\ ^{2-}$)

Compound Name: hexaaqua-copper(ii) bis(4-aminopyridinium) bis(sulfate)

Space Group:	P-1	***Cell:***	***a*** 6.179(3)	***b*** 7.059(3)	***c*** 12.442(5)		
Space Group No.:	2	***(Å,°)***	α 99.80(3)	β 98.87(3)	γ 99.66(3)		
R-Factor (%):	2.34	***Temperature(K):***	297	***Density(g/cm^3):***	1.777		

Parameters

Fragment 1

**DIST1 (D)**	1.978
**DIST2 (D)**	2.036
**DIST3 (D)**	1.978
**DIST4 (D)**	2.036
**DIST5 (D)**	2.248
**DIST6 (D)**	2.248

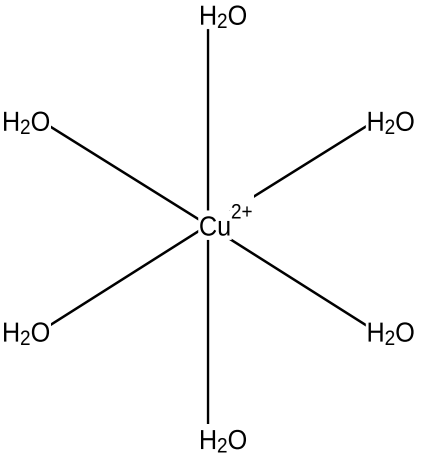

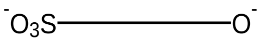

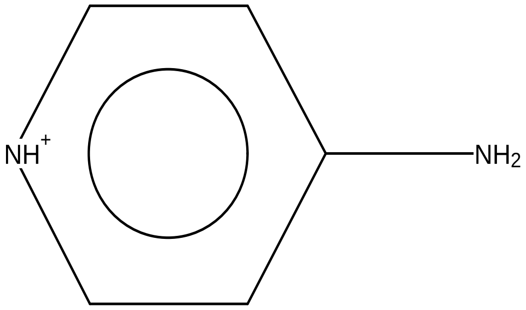

Search: search2 (Wed Jul 26 09:11:25 2017): Hit 6

ANIYUS

Reference:	R.V.Gudavarthy, N.Burla, E.A.Kulp, S.J.Limmer, E.Sinn, J.A.Switzer (2011) *J.Mater.Chem.* ,**21,**6209
Formula:	$(C_8\ H_{10}\ Cu_1\ O_{10})n,2n(H_2\ O_1)$
Compound Name:	catena-(bis(μ_2-L-Hydrogen malato)-copper(ii) dihydrate)

Space Group:	P21	***Cell:***	***a*** 7.584(4)	***b*** 10.214(5)	***c*** 8.169(4)		
Space Group No.:	4	***(Å,°)***	α 90.00	β 92.78(0)	γ 90.00		
R-Factor (%):	2.41	***Temperature(K):***	298	***Density(g/cm³):***	1.922		

Parameters

Fragment 1

**DIST1 (D)**	1.910
**DIST2 (D)**	1.947
**DIST3 (D)**	1.970
**DIST4 (D)**	1.937
**DIST5 (D)**	2.679
**DIST6 (D)**	2.336

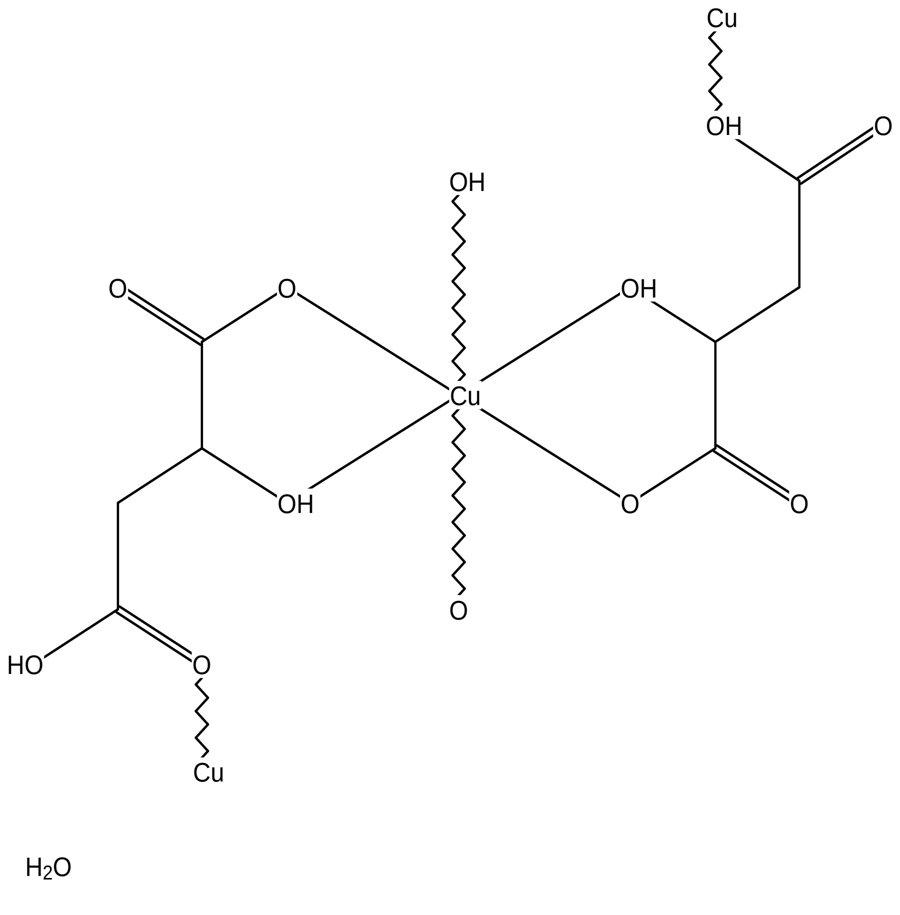

Search: search2 (Wed Jul 26 09:11:25 2017): Hit 7

AQOXAG

Reference:	T.W.T.Muesmann, C.Zitzer, A.Mietrach, T.Kluner, J.Christoffers, M.S.Wickleder (2011) *Dalton Trans.* ,**40,**3128
Formula:	$(C_6 H_8 Cl_4 Cu_1 O_{10} S_2)n$
Compound Name:	catena-[(μ_2-2,3,5,6-Tetrachlorobenzene-1,4-disulfonato-O,O')-tetra-aqua-copper(ii)]

Space Group:	Pnma	***Cell:***	***a*** 7.213(0)	***b***	21.478(0)	***c***	9.794(0)
Space Group No.:	62	***(Å,°)***	α 90.00	β	90.00	γ	90.00
R-Factor (%):	2.66	***Temperature(K):***	153	***Density(g/cm³):***	2.231		

Parameters

Fragment 1	
**DIST1 (D)**	1.940
**DIST2 (D)**	1.958
**DIST3 (D)**	1.940
**DIST4 (D)**	1.958
**DIST5 (D)**	2.447
**DIST6 (D)**	2.447

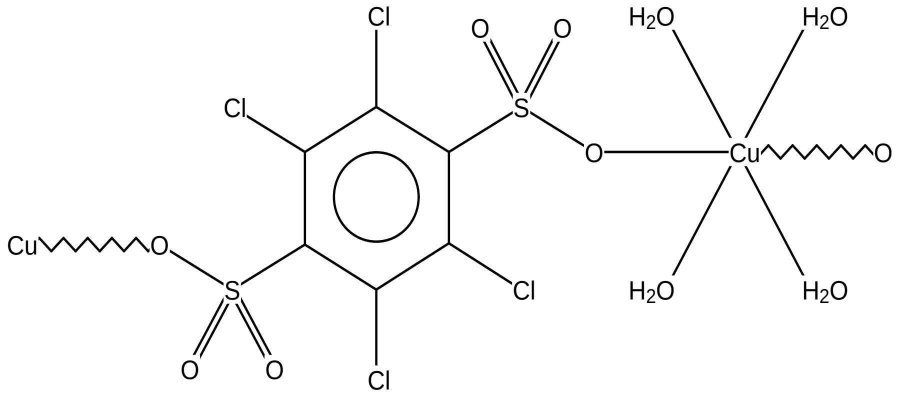

Search: search2 (Wed Jul 26 09:11:25 2017): Hit 8

AQOXIO

Reference: T.W.T.Muesmann, C.Zitzer, A.Mietrach, T.Kluner, J.Christoffers, M.S.Wickleder (2011) *Dalton Trans.* ,**40,**3128

Formula: $H_{12}\ Cu_1\ O_6\ ^{2+}, C_6\ F_4\ O_6\ S_2\ ^{2-}$

Compound Name: Hexa-aqua-copper(ii) 2,3,5,6-tetrafluorobenzene-1,4-disulfonate

Space Group:	P-1	***Cell:***	***a***	5.104(0)	***b***	7.447(0)	***c***	10.778(0)
Space Group No.:	2	***(Å,°)***	α	85.63(0)	β	77.45(0)	γ	76.02(0)

R-Factor (%): 2.65 ***Temperature(K):*** 153 ***Density(g/cm³):*** 2.054

Parameters

Fragment 1

**DIST1 (D)**	1.948
**DIST2 (D)**	1.979
**DIST3 (D)**	1.948
**DIST4 (D)**	1.979
**DIST5 (D)**	2.380
**DIST6 (D)**	2.380

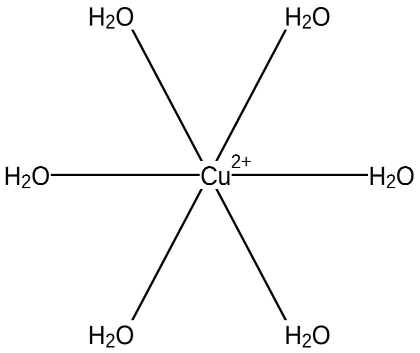

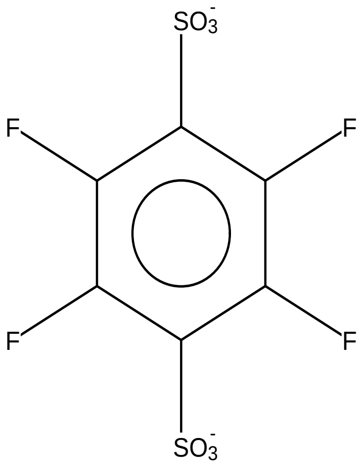

Search: search2 (Wed Jul 26 09:11:25 2017): Hit 9

AXAFIO

Reference: F.Pointillart, C.Train, M.Gruselle, F.Villain, H.W.Schmalle, D.Talbot, P.Gredin, S.Decurtins, M.Verdaguer (2004) *Chem.Mater.* ,**16,** 832

Formula: $(C_{30}\ H_{24}\ N_6\ Ru_1\ ^{2+})n,n(C_6\ Cu_2\ O_{12}\ ^{2-})$

Compound Name: catena-[tris(2,2'-Bipyridyl)-ruthenium(ii) tris(μ_2-oxalato)-di-copper(ii)]

Space Group:	P4132	***Cell:***	***a***	15.297(0)	***b***	15.297(0)	***c***	15.297(0)
Space Group No.:	213	***(Å,°)***	α	90.00	β	90.00	γ	90.00

R-Factor (%): 2.72 ***Temperature(K):*** 183 ***Density(g/cm³):*** 1.783

Parameters

Fragment 1

**DIST1 (D)**	2.109
**DIST2 (D)**	2.087
**DIST3 (D)**	2.087
**DIST4 (D)**	2.109
**DIST5 (D)**	2.109
**DIST6 (D)**	2.087

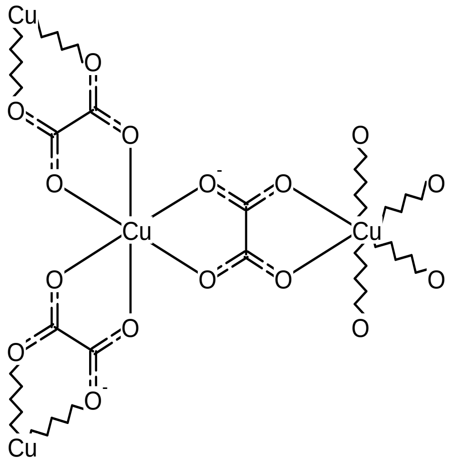

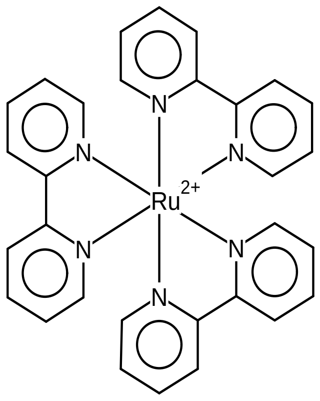

Search: search2 (Wed Jul 26 09:11:25 2017): Hit 10

BESNOC01

Reference:	Xin-Yan Zhang (2007)<br>*Acta Crystallogr.,Sect.E:Struct.Rep.Online* ,**63,**m1254
Formula:	$C_8 H_{14} Cu_1 O_{12}$
Compound Name:	Diaqua-bis(malato-O,O')-copper(ii)

Space Group:	P21/c	***Cell:***	***a***	8.476(0)	***b***	7.438(0)	***c***	10.312(0)
Space Group No.:	14	***(Å,°)***	α	90.00	β	102.68(0)	γ	90.00

R-Factor (%): 2.17 ***Temperature(K):*** 293 ***Density(g/cm^3):*** 1.915

Parameters

Fragment 1

**DIST1 (D)**	1.913
**DIST2 (D)**	1.951
**DIST3 (D)**	1.951
**DIST4 (D)**	1.913
**DIST5 (D)**	2.515
**DIST6 (D)**	2.515

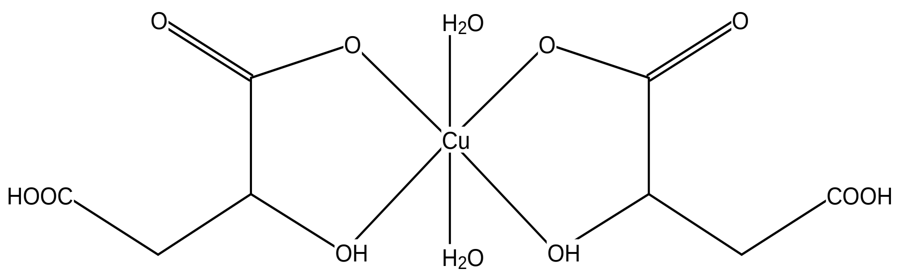

Search: search2 (Wed Jul 26 09:11:25 2017): Hit 11

BOVTAH02

Reference:	W.Clegg, J.M.Holcroft, N.C.Martin (2015) *CrystEngComm* , **17,**2857
Formula:	$(C_{10}\ H_{14}\ Cu_2\ O_{14})n,4(H_2\ O_1)$
Compound Name:	catena-((μ_4-1,2,4,5-Benzenetetracarboxylato-O,O',O'',O''')-bis(triaqua-copper) tetrahydrate)

Space Group:	C2/c	***Cell:***	***a*** 12.152(0)	***b*** 18.141(1)	***c*** 9.582(0)		
Space Group No.:	15	***(Å,°)***	α 90.00	β 113.50(0)	γ 90.00		
R-Factor (%):	2.35	***Temperature(K):***	160	***Density(g/cm^3):***	1.911		

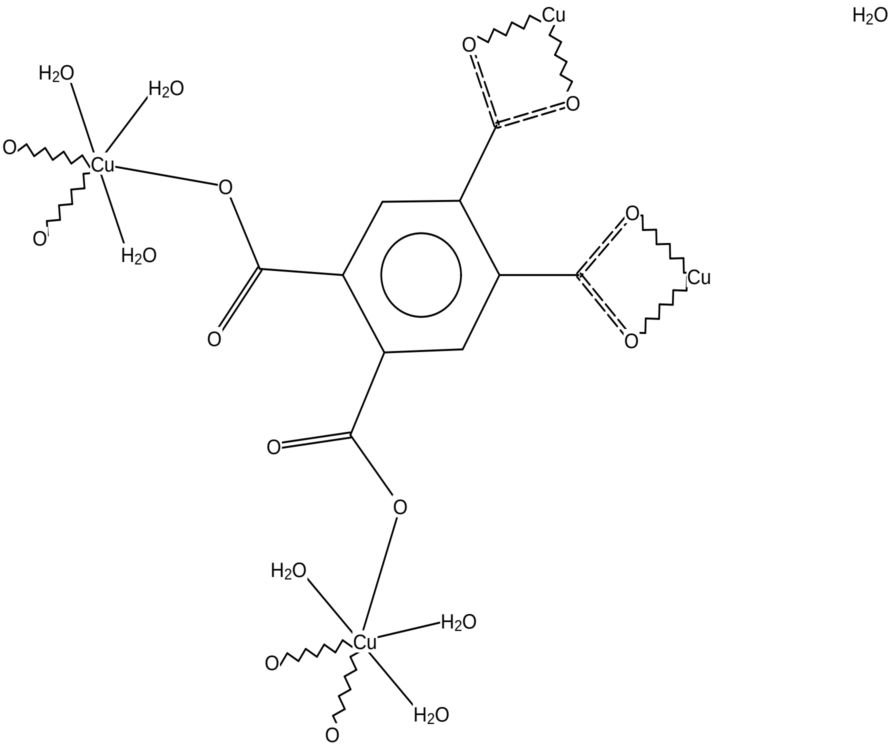

Parameters

Fragment 1

**DIST1 (D)**	1.952
**DIST2 (D)**	1.955
**DIST3 (D)**	1.955
**DIST4 (D)**	2.009
**DIST5 (D)**	2.243
**DIST6 (D)**	2.676

Search: search2 (Wed Jul 26 09:11:25 2017): Hit 12

BUDSCU

Reference: F.Charbonnier, R.Faure, H.Loiseleur (1977)
Acta Crystallogr.,Sect.B:Struct.Crystallogr.Cryst.Chem. ,**33,**3759

Formula: $(C_4\ H_{16}\ Cu_1\ O_{10}\ S_2)n$

Compound Name: catena-((μ_2-Butane-1,4-disulfonato-O,O')-tetra-aqua-copper(ii))

Space Group:	P21/c	***Cell:***	***a***	6.213(0)	***b***	9.081(2)	***c***	11.324(3)
Space Group No.:	14	***(Å,°)***	α	90.00	β	108.65(1)	γ	90.00

R-Factor (%): 2.30 ***Temperature(K):*** 295 ***Density(g/cm³):*** 1.930

Parameters

Fragment 1

**DIST1 (D)**	1.950
**DIST2 (D)**	1.947
**DIST3 (D)**	1.950
**DIST4 (D)**	1.947
**DIST5 (D)**	2.360
**DIST6 (D)**	2.360

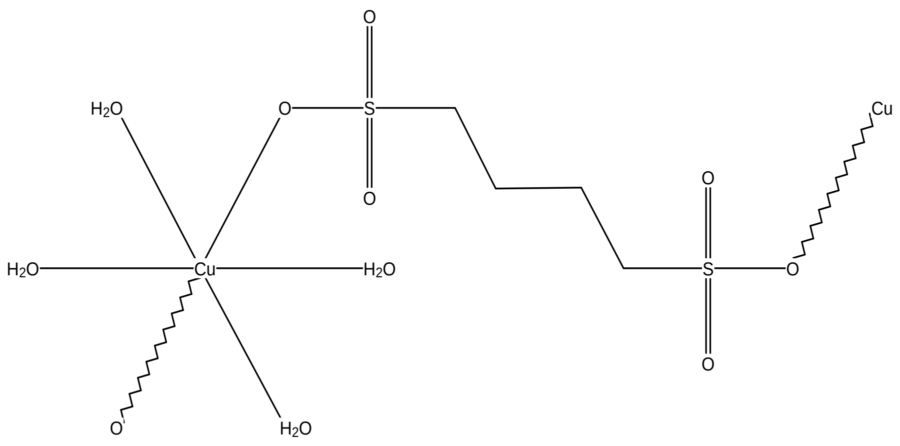

Search: search2 (Wed Jul 26 09:11:25 2017): Hit 13

BUGCEM

Reference:	A.Mietrach, T.W.T.Muesmann, J.Christoffers, M.S.Wickleder (2009) *Eur.J.Inorg.Chem.* ,5328
Formula:	$(C_6\ H_{12}\ Cu_1\ O_{10}\ S_2)n$
Compound Name:	catena-((μ_2-benzene-1,4-disulfonato)-tetraaqua-copper(ii))

Space Group:	P21/n	***Cell:***	***a*** 5.975(0)	***b*** 10.936(0)	***c*** 9.922(0)
Space Group No.:	14	***(Å,°)***	α 90.00	β 105.17(0)	γ 90.00
R-Factor (%):	1.98	***Temperature(K):*** 153		***Density(g/cm³):*** 1.973	

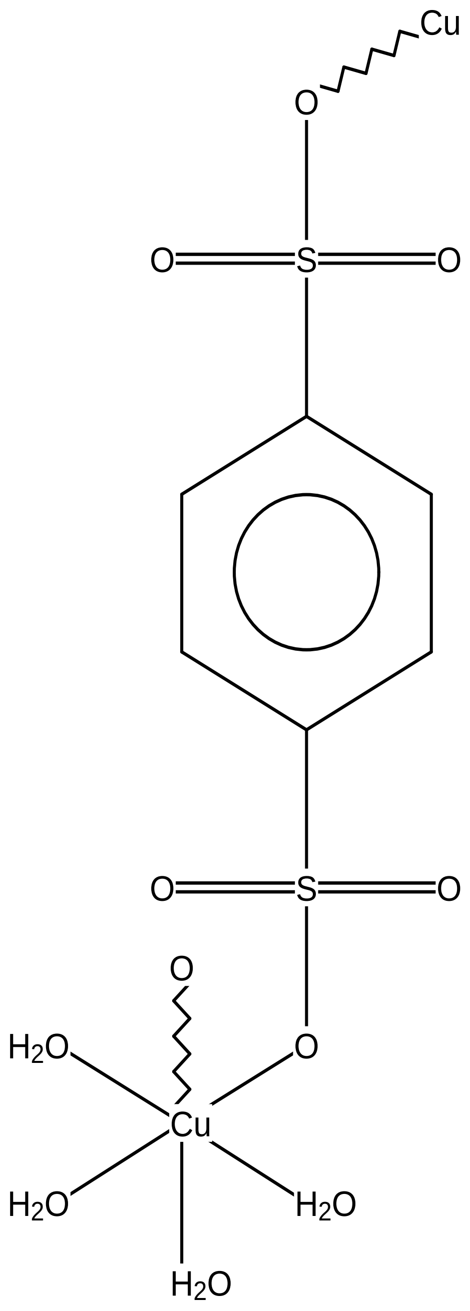

Parameters

Fragment 1

**DIST1 (D)**	1.963
**DIST2 (D)**	1.938
**DIST3 (D)**	1.963
**DIST4 (D)**	1.938
**DIST5 (D)**	2.412
**DIST6 (D)**	2.412

Search: search2 (Wed Jul 26 09:11:25 2017): Hit 14

BUGCIQ

Reference: A.Mietrach, T.W.T.Muesmann, J.Christoffers, M.S.Wickleder (2009) *Eur.J.Inorg.Chem.* ,5328

Formula: $C_6 H_{14} Cu_1 O_{11} S_2,H_2 O_1$

Compound Name: Pentaaqua-(benzene-1,3-disulfonato)-copper(ii) monohydrate

Space Group:	P21/n	***Cell:***	***a*** 6.321(0)	***b***	20.226(1)	***c***	11.283(0)
Space Group No.:	14	***(Å,°)***	α 90.00	β	99.26(0)	γ	90.00

R-Factor (%): 1.95 ***Temperature(K):*** 153 ***Density(g/cm³):*** 1.903

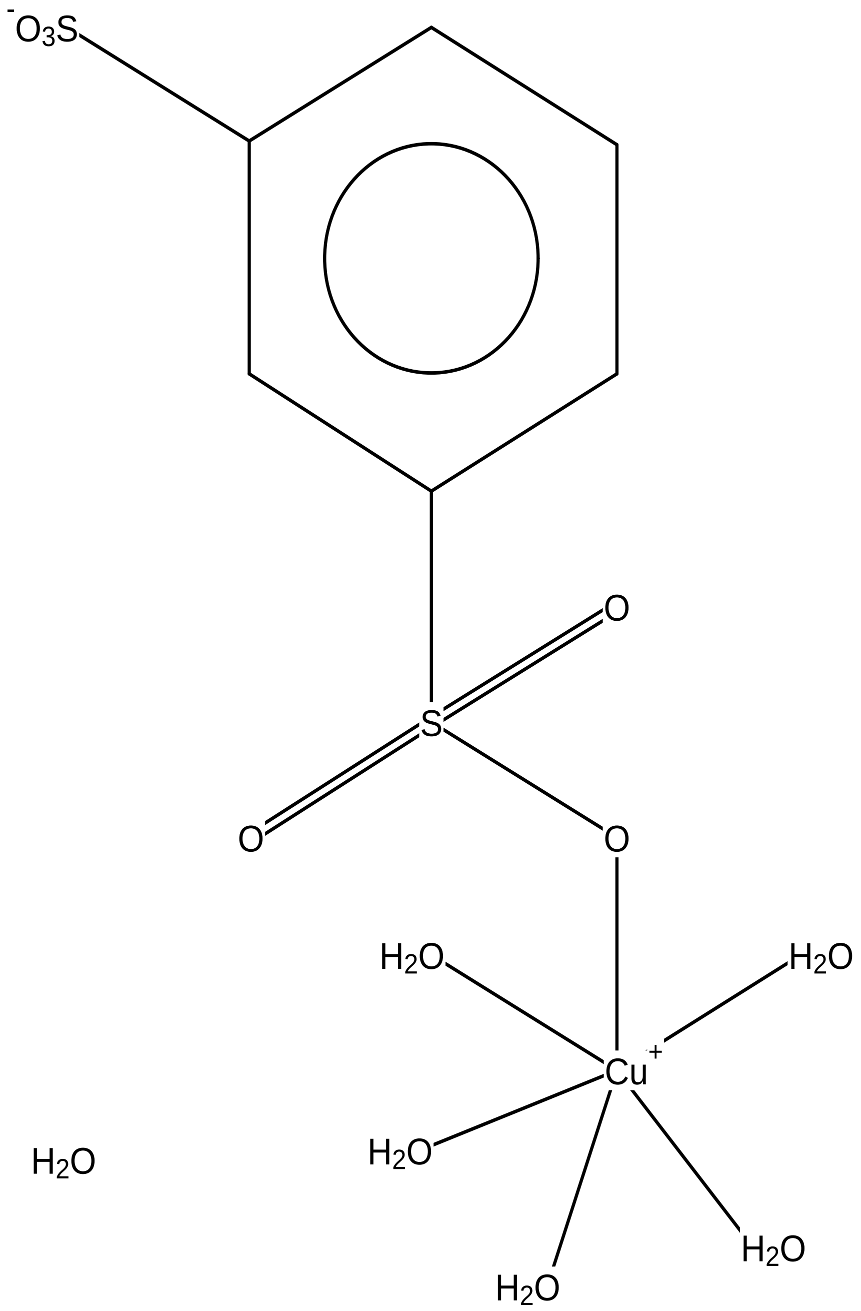

Parameters

Fragment 1

**DIST1 (D)**	1.948
**DIST2 (D)**	2.000
**DIST3 (D)**	2.004
**DIST4 (D)**	1.962
**DIST5 (D)**	2.337
**DIST6 (D)**	2.292

Search: search2 (Wed Jul 26 09:11:25 2017): Hit 15

BUSWUI

Reference:	Yue Ma, XiaoPing Chen, Dong Cao, ShiPing Yan, DaiZheng Liao (2009) *Sci.China,Ser.B:Chem.* ,**52,**1438
Formula:	$(C_{40}\ H_{40}\ Cl_8\ Cu_2\ N_6\ O_{16})n,2n(H_2\ O_1)$
Compound Name:	catena-(bis(μ_2-3,4,5,6-tetrachlorophthalato)-bis(2-(3'-pyridyl)-4,4,5,5-tetramethylimidazoline-1-oxyl-3-oxide-N)-tetra-aqua-di-copper(ii) dihydrate)

Space Group:	P-1	***Cell:***	***a***	7.424(2)	***b***	11.256(2)	***c***	16.495(4)
Space Group No.:	2	***(Å,°)***	α	70.32(0)	β	86.06(0)	γ	80.10(0)

R-Factor (%): 2.80 ***Temperature(K):*** 293 ***Density(g/cm³):*** 1.698

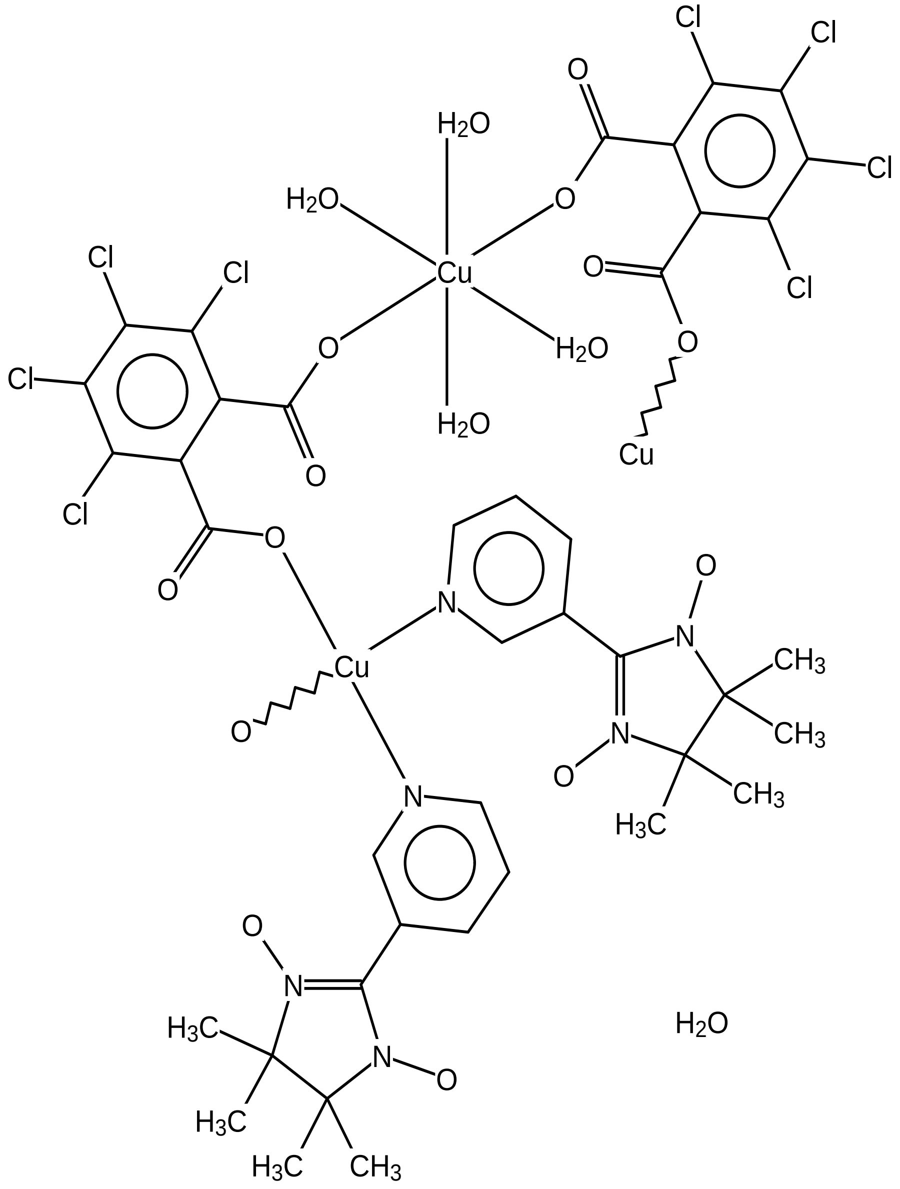

Parameters

Fragment 1

**DIST1 (D)**	2.070
**DIST2 (D)**	1.937
**DIST3 (D)**	1.937
**DIST4 (D)**	2.070
**DIST5 (D)**	2.302
**DIST6 (D)**	2.302

Search: search2 (Wed Jul 26 09:11:25 2017): Hit 16

CABYEJ01

Reference: Yu-fang Pan, Wen-guan Lu (2006)
Jiangxi Shifan Daxue Xuebao Ziran Kexueban(Chin.)(J.Jiangxi Normal Univ.(Nat.Sci.))
30,209

Formula: $(C_8 H_{12} Cu_2 O_{14})n,4n(H_2 O_1)$

Compound Name: catena-((μ_4-(S,S)-Tartrato)-(μ_2-(S,S)-tartrato)-diaqua-di-copper(ii) tetrahydrate)

Space Group:	P21	***Cell:***	***a***	8.370(2)	***b***	8.755(2)	***c***	12.127(3)
Space Group No.:	4	***(Å,°)***	α	90.00	β	104.54(2)	γ	90.00

R-Factor (%): 2.32 ***Temperature(K):*** 296 ***Density(g/cm³):*** 2.051

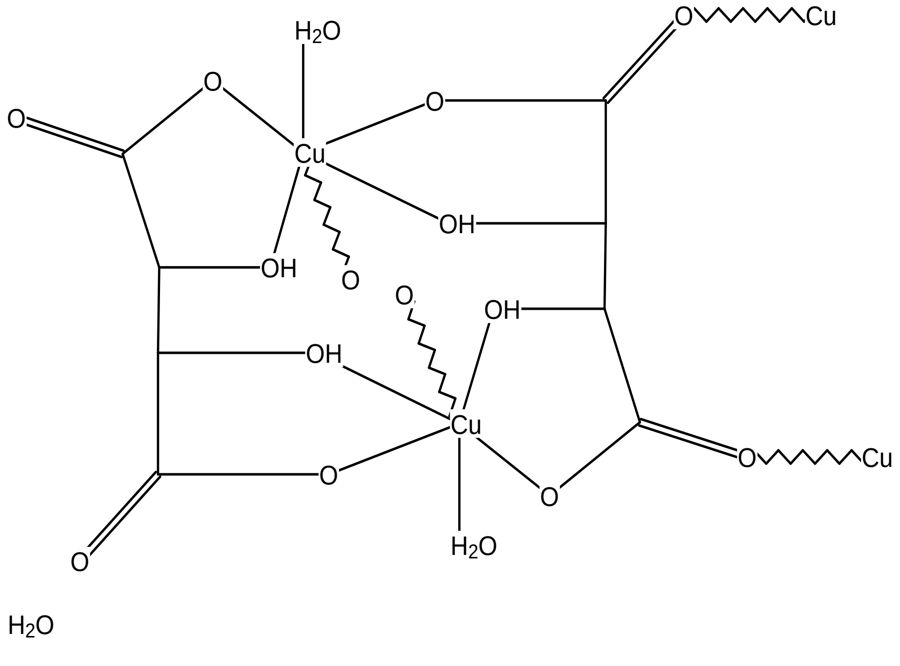

Parameters

Fragment 1

**DIST1 (D)**	1.934
**DIST2 (D)**	1.938
**DIST3 (D)**	2.027
**DIST4 (D)**	1.980
**DIST5 (D)**	2.397
**DIST6 (D)**	2.385

Fragment 2

**DIST1 (D)**	1.936
**DIST2 (D)**	1.924
**DIST3 (D)**	2.006
**DIST4 (D)**	1.976
**DIST5 (D)**	2.431
**DIST6 (D)**	2.344

Search: search2 (Wed Jul 26 09:11:25 2017): Hit 17

CAMZUM

Reference:	S.Bieller, H.-W.Lerner, M.Bolte (2005)<br>*Acta Crystallogr.,Sect.E:Struct.Rep.Online* ,**61,**m928
Formula:	C_{12} H_{36} Cu_1 O_6 S_6 $^{2+}$,2(H_1 O_4 S_1 $^{1-}$)
Compound Name:	hexakis(Dimethylsulfoxide)copper(ii) bis(hydrogen sulfate)

Space Group:	P-1	***Cell:***	***a*** 8.227(0)	***b*** 9.466(0)	***c*** 9.595(0)		
Space Group No.:	2	***(Å,°)***	α 97.56(0)	β 90.42(0)	γ 90.24(0)		
R-Factor (%):	2.32	***Temperature(K):***	173	***Density(g/cm³):***	1.629		

Parameters

Fragment 1

**DIST1 (D)**	1.976
**DIST2 (D)**	2.022
**DIST3 (D)**	1.976
**DIST4 (D)**	2.022
**DIST5 (D)**	2.344
**DIST6 (D)**	2.344

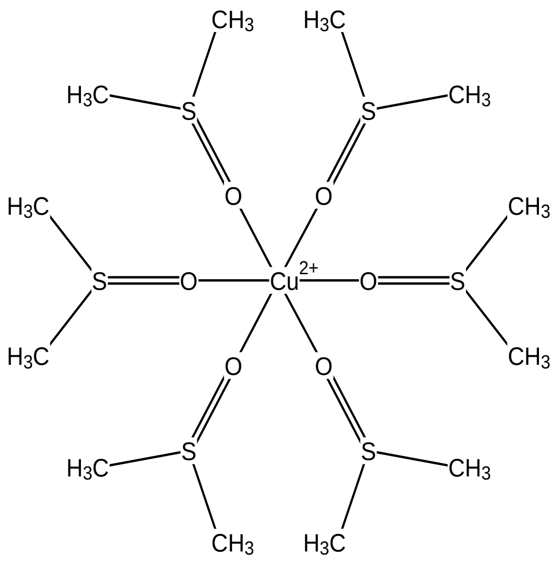

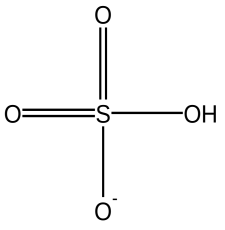

Search: search2 (Wed Jul 26 09:11:25 2017): Hit 18

CDACDH25

Reference:	C.J.Simmons, H.Stratemeier, M.A.Hitchman, D.Reinen, V.M.Masters, M.J.Riley (2011) *Inorg.Chem.* ,**50,**4900
Formula:	$C_6 H_{14} Cu_1 O_8$
Compound Name:	trans-Diaqua-bis(2-methoxyacetato-O,O')-copper(ii)

Space Group:	P21/n	***Cell:***	***a*** 6.974(0)	***b*** 10.181(0)	***c*** 7.242(0)		
Space Group No.:	14	***(Å,°)***	α 90.00	β 96.88(0)	γ 90.00		
R-Factor (%):	2.20	***Temperature(K):***	320	***Density(g/cm^3):***	1.806		

Parameters

Fragment 1

**DIST1 (D)**	1.928
**DIST2 (D)**	1.928
**DIST3 (D)**	2.133
**DIST4 (D)**	2.169
**DIST5 (D)**	2.133
**DIST6 (D)**	2.169

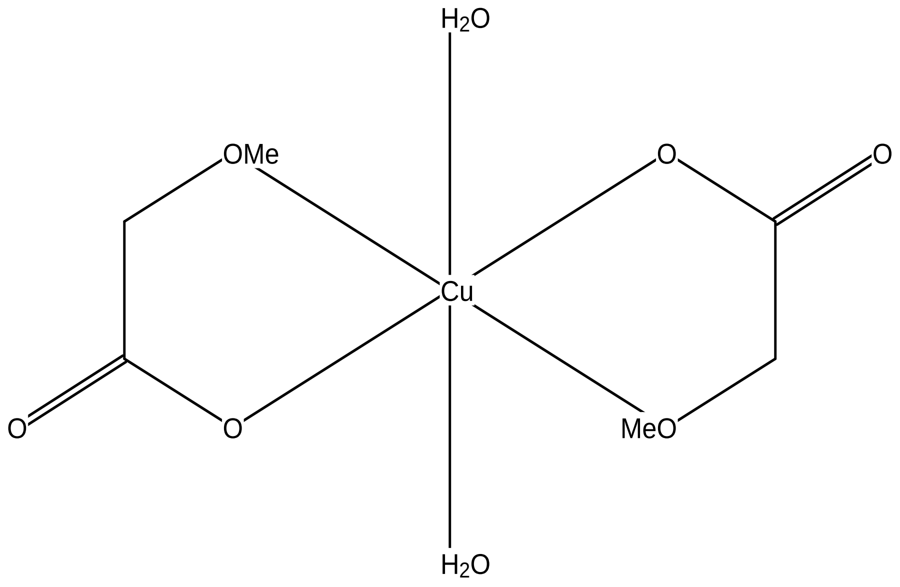

Search: search2 (Wed Jul 26 09:11:25 2017): Hit 19

CICYET01

Reference:	M.Fainerman-Melnikova, J.K.Clegg, A.A.H.Pakchung, P.Jensen, R.Codd (2010) *CrystEngComm* ,**12,**4217
Formula:	$(C_{14}\ H_{20}\ Cu_2\ O_{20})n$
Compound Name:	catena-[bis(μ_2-4-Pyranone-2,6-dicarboxylato)-octaaqua-di-copper]

Space Group:	P-1	***Cell:***	***a*** 4.966(1)	***b*** 9.909(3)	***c*** 10.491(4)		
Space Group No.:	2	***(Å,°)***	α 94.10(0)	β 94.91(0)	γ 97.22(0)		
R-Factor (%):	2.57	***Temperature(K):***	150	***Density(g/cm³):***	2.075		

Parameters

Fragment 1

**DIST1 (D)**	1.973
**DIST2 (D)**	1.983
**DIST3 (D)**	1.983
**DIST4 (D)**	1.973
**DIST5 (D)**	2.455
**DIST6 (D)**	2.455

Fragment 2

**DIST1 (D)**	1.911
**DIST2 (D)**	1.962
**DIST3 (D)**	1.911
**DIST4 (D)**	1.962
**DIST5 (D)**	2.534
**DIST6 (D)**	2.534

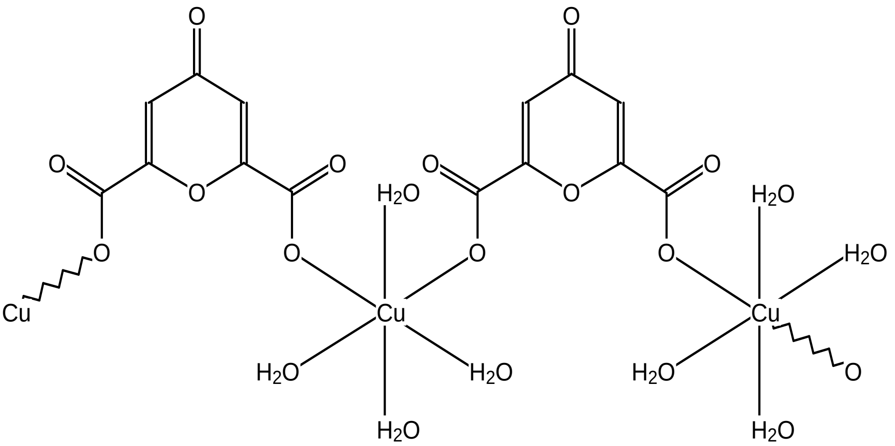

Search: search2 (Wed Jul 26 09:11:25 2017): Hit 20

CIWTOT

Reference:	D.Fischer, T.M.Klapotke, M.Reymann, J.Stierstorfer (2014) *Chem.-Eur.J.* ,**20,**6401
Formula:	$(C_4\ H_8\ Cu_1\ N_{10}\ O_{10})n$
Compound Name:	catena-[(μ_2-(diazene-1,2-diyldi-1,2,5-oxadiazole-4,3-diyl) bis(nitroazanide))-tetraaqua-copper(ii)]

Space Group:	P-1	***Cell:***	***a*** 4.931(0)	***b*** 7.937(0)	***c*** 8.493(0)		
Space Group No.:	2	***(Å,°)***	α 68.91(0)	β 83.97(0)	γ 86.09(0)		
R-Factor (%):	2.62	***Temperature(K):***	100	***Density(g/cm³):***	2.261		

Parameters

Fragment 1

**DIST1 (D)**	1.947
**DIST2 (D)**	1.991
**DIST3 (D)**	1.947
**DIST4 (D)**	1.991
**DIST5 (D)**	2.349
**DIST6 (D)**	2.349

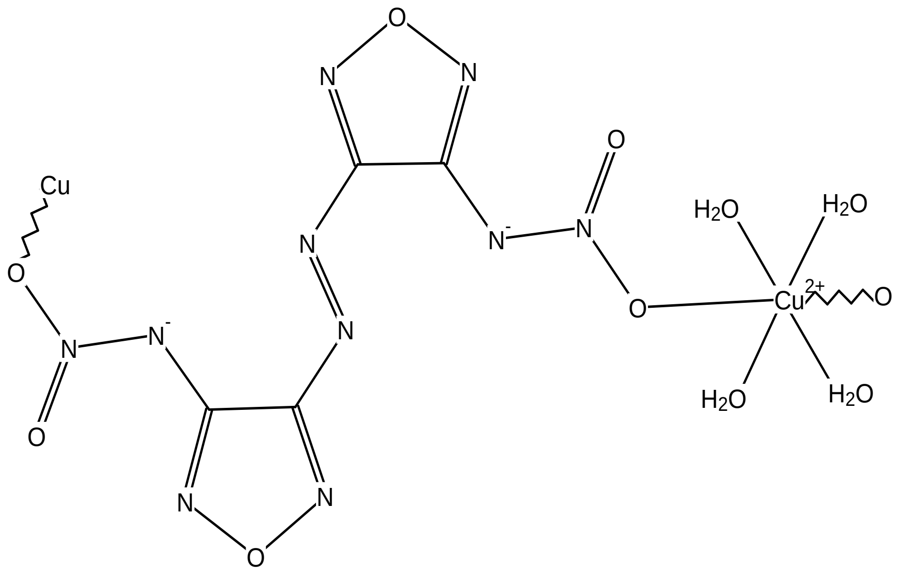

Search: search2 (Wed Jul 26 09:11:25 2017): Hit 21

COWTUE

Reference:	Chih-Chieh Wang, Meu-Ju Ke, Cheng-Hsiao Tsai, I-Hsuan Chen, Shin-I Lin, Tzuen-Yeuan Lin, Li-Mei Wu, Gene-Hsiang Lee, Hwo-Shuenn Sheu, V.E.Fedorov (2009) *Cryst.Growth Des.* ,**9,**1013
Formula:	$(C_{14} H_{20} Cu_2 N_4 O_{12})n$
Compound Name:	catena-(bis(μ_2-croconato)-diaaqua-bis(ethylenediamine)-di-copper(ii))
Synonym:	catena-(bis(μ_2-4,5-dioxycyclopent-4-ene-1,2,3-trione)-diaqua-bis(ethylenediamine)-di-copper(ii))

Space Group:	P21/c	***Cell:***	***a*** 10.007(0)	***b*** 13.944(0)	***c*** 7.010(0)
Space Group No.:	14	***(Å,°)***	α 90.00	β 98.08(0)	γ 90.00
R-Factor (%):	2.58	***Temperature(K):*** 295		***Density(g/cm^3):*** 1.932	

Parameters

Fragment 1	
**DIST1 (D)**	1.957
**DIST2 (D)**	1.991
**DIST3 (D)**	1.991
**DIST4 (D)**	1.957
**DIST5 (D)**	2.417
**DIST6 (D)**	2.417

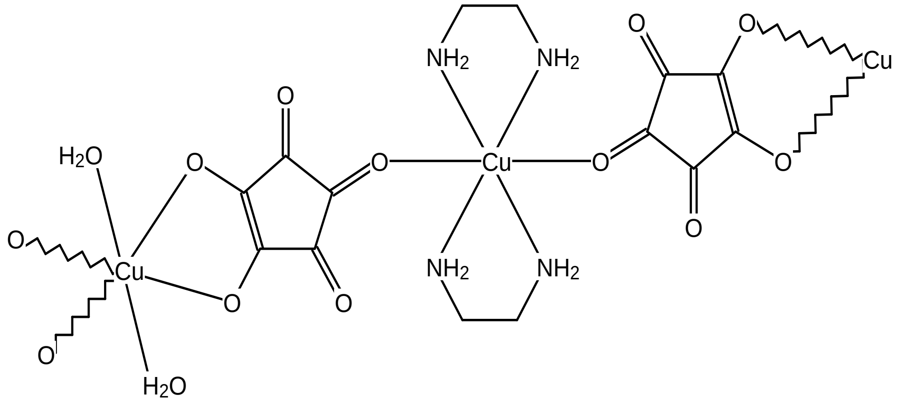

Search: search2 (Wed Jul 26 09:11:25 2017): Hit 22

CUACDH03

Reference:	K.Prout, A.Edwards, V.Mtetwa, J.Murray, J.F.Saunders, F.J.C.Rossotti (1997) *Inorg.Chem.* ,**36,**2820
Formula:	$C_6\ H_{14}\ Cu_1\ O_8$
Compound Name:	trans-Diaqua-bis(methoxyacetato)-copper(ii)

Space Group:	P1121/n	***Cell:***	***a***	6.900(2)	***b***	7.190(1)	***c***	9.874(2)
Space Group No.:	14	***(Å,°)***	α	90.00	β	90.00	γ	95.70(1)

R-Factor (%):	2.45	***Temperature(K):***	125	***Density(g/cm³):***	1.892

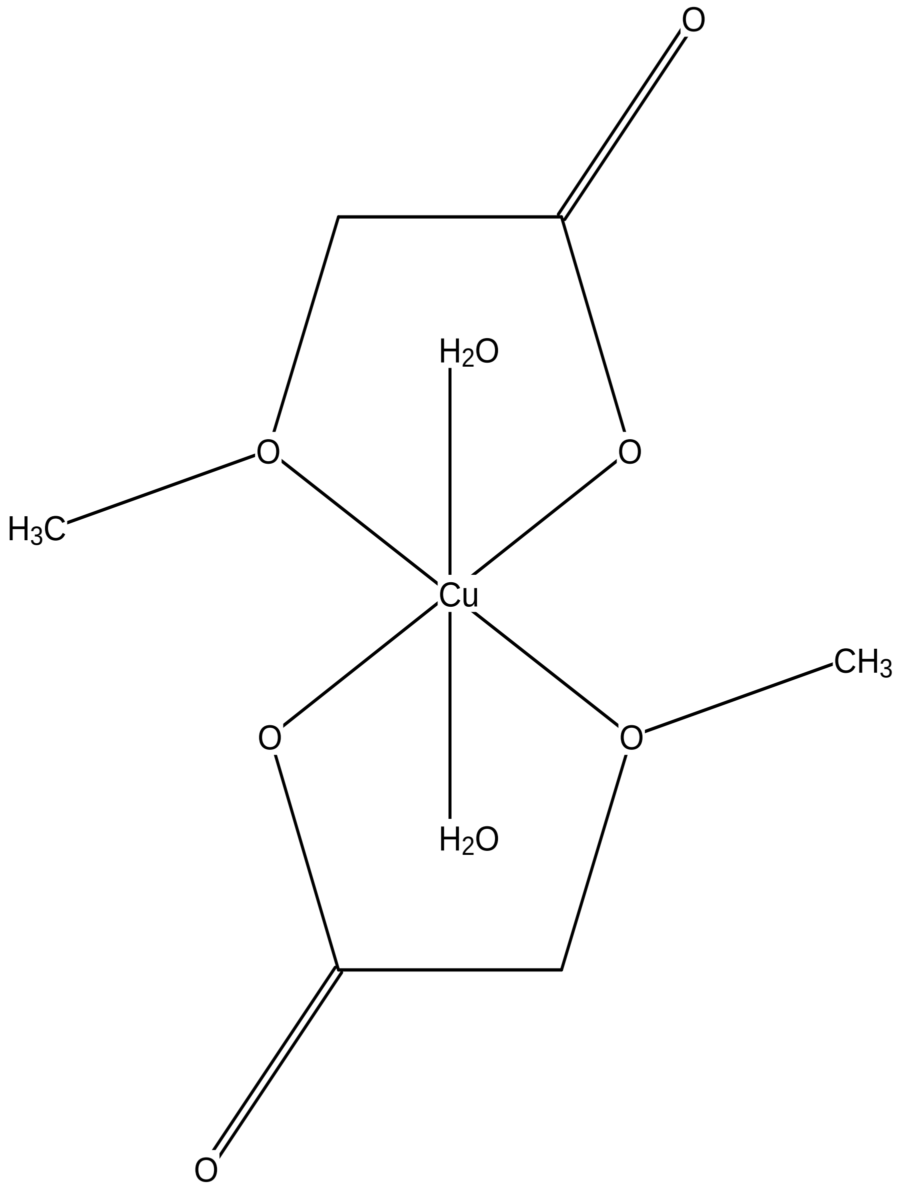

Parameters

Fragment 1

**DIST1 (D)**	2.048
**DIST2 (D)**	1.952
**DIST3 (D)**	1.952
**DIST4 (D)**	2.048
**DIST5 (D)**	2.205
**DIST6 (D)**	2.205

Search: search2 (Wed Jul 26 09:11:25 2017): Hit 23

CUACDH11

Reference:	H.-L.Zhu, Q.-F.Zeng, D.-S.Xia, X.-L.Zhu, X.Liu, D.-Q.Wang (2003) *Z.Kristallogr.-New Cryst.Struct.* ,**218,**315
Formula:	$C_6 H_{14} Cu_1 O_8$
Compound Name:	Diaqua-bis(2-methoxyacetato-O,O')-copper(ii)

Space Group:	P21/n	***Cell:***	***a*** 6.955(1)	***b*** 10.123(2)	***c*** 7.226(1)		
Space Group No.:	14	***(Å,°)***	α 90.00	β 96.57(1)	γ 90.00		
R-Factor (%):	2.57	***Temperature(K):***	292	***Density(g/cm³):***	1.825		

Parameters

Fragment 1

**DIST1 (D)**	1.932
**DIST2 (D)**	1.932
**DIST3 (D)**	2.147
**DIST4 (D)**	2.138
**DIST5 (D)**	2.147
**DIST6 (D)**	2.138

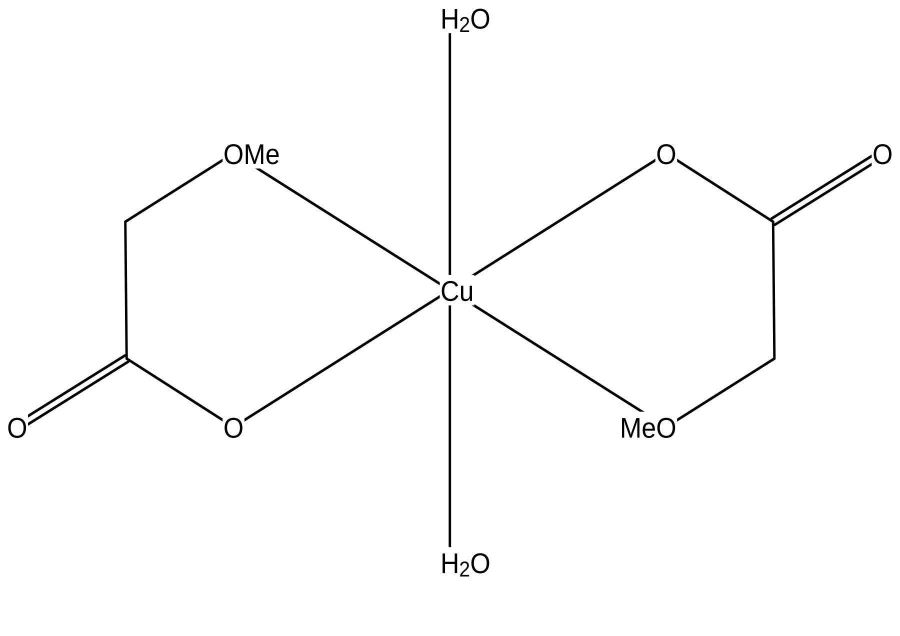

Search: search2 (Wed Jul 26 09:11:25 2017): Hit 24

CUACDH13

Reference: C.J.Simmons, H.Stratemeier, M.A.Hitchman, D.Reinen, V.M.Masters, M.J.Riley (2011) *Inorg.Chem.* ,**50,**4900

Formula: $C_6\ H_{14}\ Cu_1\ O_8$

Compound Name: trans-Diaqua-bis(2-methoxyacetato-O,O')-copper(ii)

Space Group:	P21/n	***Cell:***	***a*** 6.898(0)		***b*** 9.858(0)		***c*** 7.197(0)
Space Group No.:	14	***(Å,°)***	α 90.00		β 95.71(0)		γ 90.00
R-Factor (%):	1.80	***Temperature(K):***	90		***Density(g/cm³):***		1.894

Parameters

Fragment 1

**DIST1 (D)**	2.042
**DIST2 (D)**	1.955
**DIST3 (D)**	1.955
**DIST4 (D)**	2.042
**DIST5 (D)**	2.205
**DIST6 (D)**	2.205

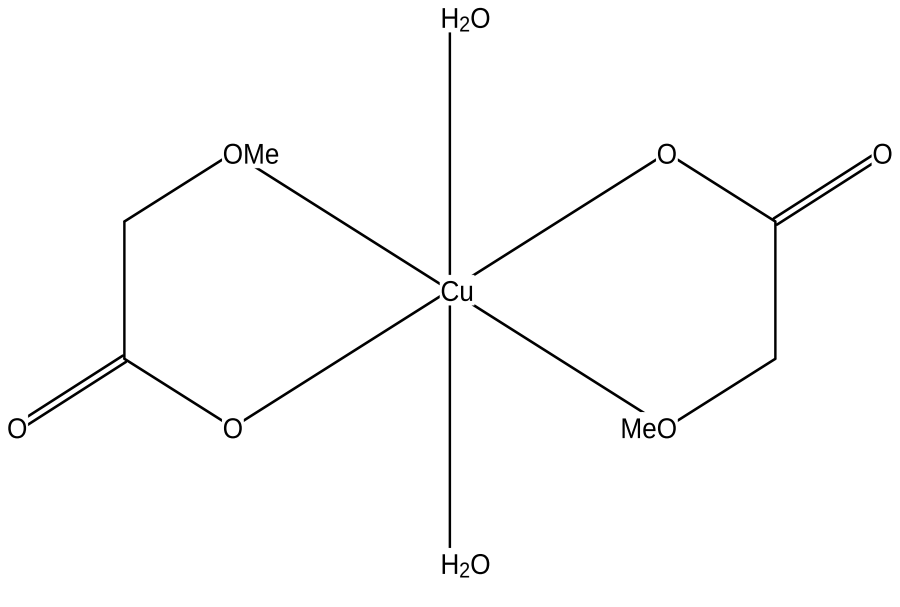

Search: search2 (Wed Jul 26 09:11:25 2017): Hit 25

CUACDH14

Reference:	C.J.Simmons, H.Stratemeier, M.A.Hitchman, D.Reinen, V.M.Masters, M.J.Riley (2011) *Inorg.Chem.* ,**50,**4900
Formula:	C_6 H_{14} Cu_1 O_8
Compound Name:	trans-Diaqua-bis(2-methoxyacetato-O,O')-copper(ii)

Space Group:	P21/n	***Cell:***	***a*** 6.902(0)	***b*** 9.881(0)	***c*** 7.198(0)
Space Group No.:	14	***(Å,°)***	α 90.00	β 95.73(0)	γ 90.00

R-Factor (%): 1.80 ***Temperature(K):*** 120 ***Density(g/cm^3^):*** 1.888

Parameters

Fragment 1

**DIST1 (D)**	2.047
**DIST2 (D)**	1.954
**DIST3 (D)**	1.954
**DIST4 (D)**	2.047
**DIST5 (D)**	2.203
**DIST6 (D)**	2.203

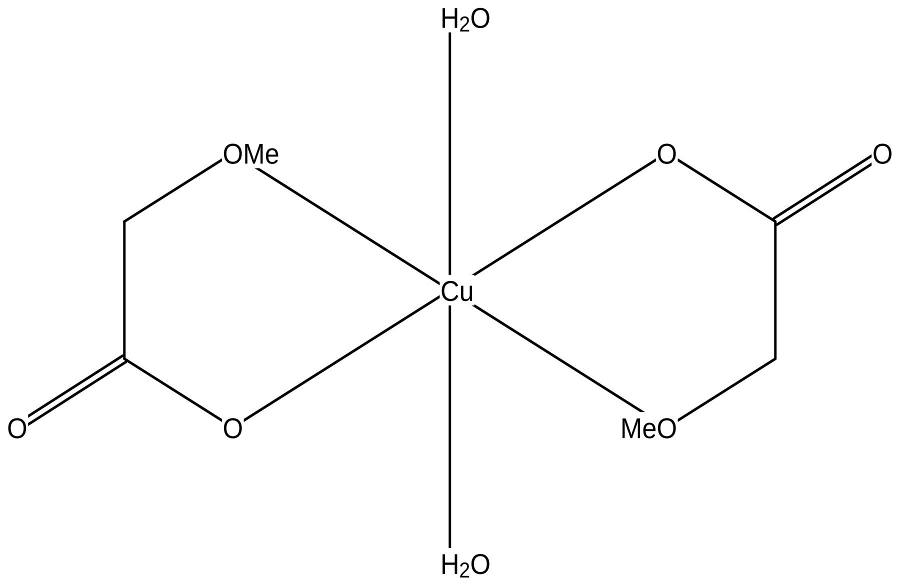

Search: search2 (Wed Jul 26 09:11:25 2017): Hit 26

CUACDH15

Reference:	C.J.Simmons, H.Stratemeier, M.A.Hitchman, D.Reinen, V.M.Masters, M.J.Riley (2011) *Inorg.Chem.* ,**50,**4900
Formula:	$C_6 H_{14} Cu_1 O_8$
Compound Name:	trans-Diaqua-bis(2-methoxyacetato-O,O')-copper(ii)

Space Group:	P21/n	***Cell:***	***a*** 6.905(0)	***b*** 9.899(0)	***c*** 7.200(0)		
Space Group No.:	14	***(Å,°)***	α 90.00	β 95.75(0)	γ 90.00		
R-Factor (%):	1.85	***Temperature(K):***	140	***Density(g/cm^3):***	1.884		

Parameters

Fragment 1	
**DIST1 (D)**	2.052
**DIST2 (D)**	1.952
**DIST3 (D)**	1.952
**DIST4 (D)**	2.052
**DIST5 (D)**	2.201
**DIST6 (D)**	2.201

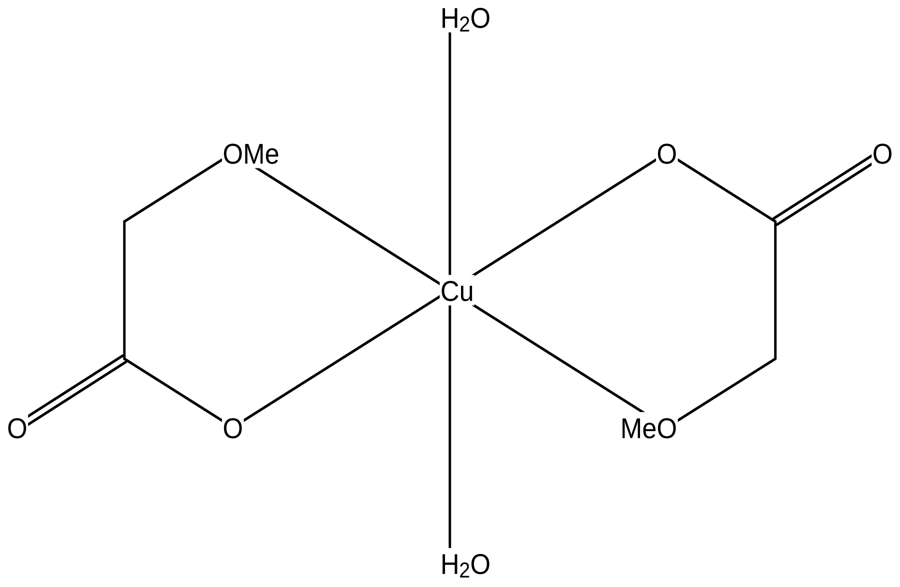

Search: search2 (Wed Jul 26 09:11:25 2017): Hit 27

CUACDH16

Reference: C.J.Simmons, H.Stratemeier, M.A.Hitchman, D.Reinen, V.M.Masters, M.J.Riley (2011) *Inorg.Chem.* ,**50,**4900

Formula: $C_6 H_{14} Cu_1 O_8$

Compound Name: trans-Diaqua-bis(2-methoxyacetato-O,O')-copper(ii)

Space Group:	P21/n	***Cell:***	***a***	6.908(0)	***b***	9.920(0)	***c***	7.201(0)
Space Group No.:	14	***(Å,°)***	α	90.00	β	95.78(0)	γ	90.00

R-Factor (%): 1.90 ***Temperature(K):*** 160 ***Density(g/cm^3):*** 1.878

Parameters

Fragment 1

**DIST1 (D)**	1.951
**DIST2 (D)**	1.951
**DIST3 (D)**	2.199
**DIST4 (D)**	2.059
**DIST5 (D)**	2.199
**DIST6 (D)**	2.059

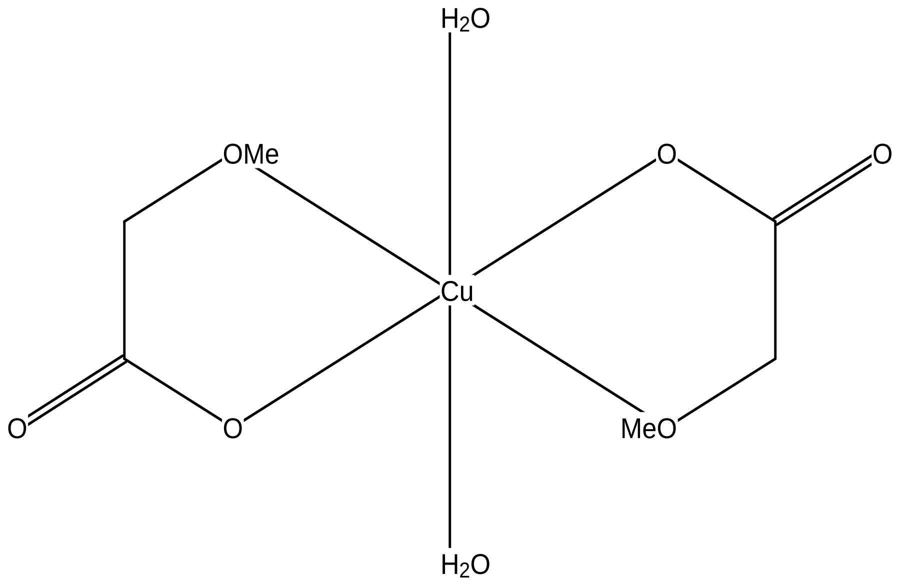

Search: search2 (Wed Jul 26 09:11:25 2017): Hit 28

CUACDH17

Reference:	C.J.Simmons, H.Stratemeier, M.A.Hitchman, D.Reinen, V.M.Masters, M.J.Riley (2011) *Inorg.Chem.* ,**50,**4900
Formula:	$C_6 H_{14} Cu_1 O_8$
Compound Name:	trans-Diaqua-bis(2-methoxyacetato-O,O')-copper(ii)

Space Group:	P21/n	***Cell:***	***a***	6.913(0)	***b***	9.945(0)	***c***	7.204(0)
Space Group No.:	14	***(Å,°)***	α	90.00	β	95.84(0)	γ	90.00
R-Factor (%):	1.98	***Temperature(K):***	180		***Density(g/cm³):***	1.872		

Parameters

Fragment 1

**DIST1 (D)**	1.949
**DIST2 (D)**	1.949
**DIST3 (D)**	2.191
**DIST4 (D)**	2.066
**DIST5 (D)**	2.191
**DIST6 (D)**	2.066

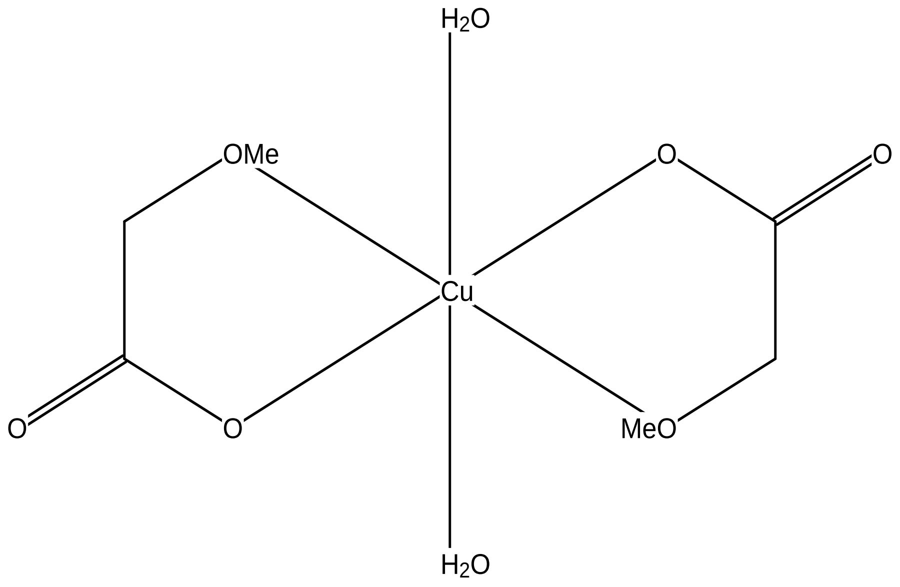

Search: search2 (Wed Jul 26 09:11:25 2017): Hit 29

CUACDH18

Reference:	C.J.Simmons, H.Stratemeier, M.A.Hitchman, D.Reinen, V.M.Masters, M.J.Riley (2011) *Inorg.Chem.* ,**50,**4900
Formula:	$C_6 H_{14} Cu_1 O_8$
Compound Name:	trans-Diaqua-bis(2-methoxyacetato-O,O')-copper(ii)

Space Group:	P21/n	***Cell:***	***a*** 6.919(0)	***b*** 9.973(0)	***c*** 7.207(0)
Space Group No.:	14	***(Å,°)***	α 90.00	β 95.94(0)	γ 90.00

R-Factor (%): 2.00 ***Temperature(K):*** 200 ***Density(g/cm³):*** 1.865

Parameters

Fragment 1

**DIST1 (D)**	1.946
**DIST2 (D)**	1.946
**DIST3 (D)**	2.187
**DIST4 (D)**	2.074
**DIST5 (D)**	2.187
**DIST6 (D)**	2.074

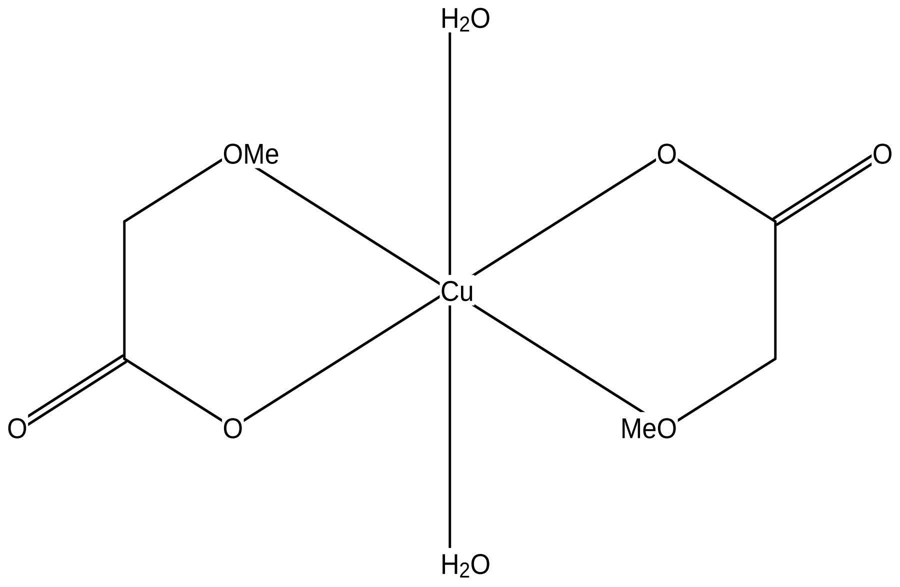

Search: search2 (Wed Jul 26 09:11:25 2017): Hit 30

CUACDH19

Reference:	C.J.Simmons, H.Stratemeier, M.A.Hitchman, D.Reinen, V.M.Masters, M.J.Riley (2011) *Inorg.Chem.* ,**50,**4900
Formula:	$C_6 H_{14} Cu_1 O_8$
Compound Name:	trans-Diaqua-bis(2-methoxyacetato-O,O')-copper(ii)

Space Group:	P21/n	***Cell:***	***a*** 6.926(0)	***b*** 10.005(0)	***c*** 7.211(0)		
Space Group No.:	14	***(Å,°)***	α 90.00	β 96.06(0)	γ 90.00		
R-Factor (%):	2.08	***Temperature(K):***	220	***Density(g/cm³):***	1.856		

Parameters

Fragment 1

**DIST1 (D)**	1.943
**DIST2 (D)**	1.943
**DIST3 (D)**	2.180
**DIST4 (D)**	2.087
**DIST5 (D)**	2.180
**DIST6 (D)**	2.087

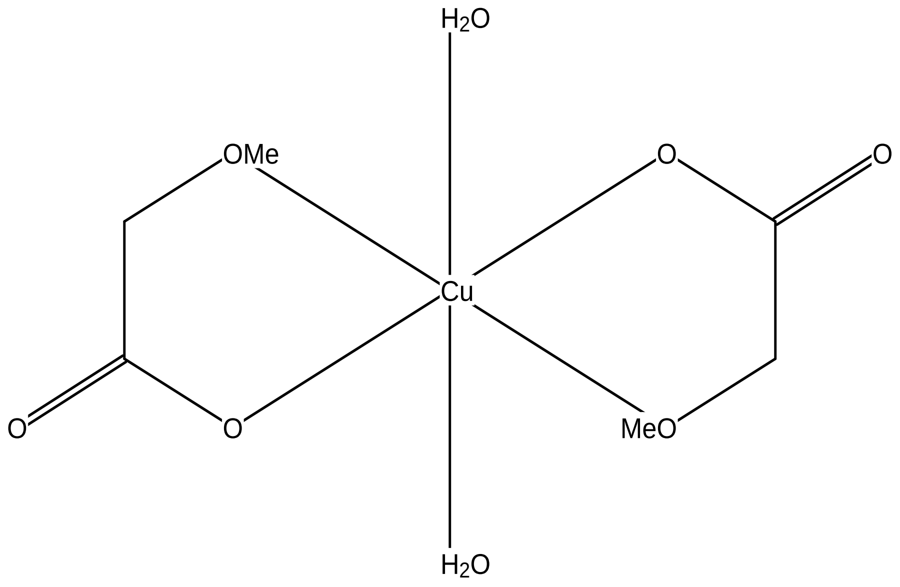

Search: search2 (Wed Jul 26 09:11:25 2017): Hit 31

CUACDH20

Reference:	C.J.Simmons, H.Stratemeier, M.A.Hitchman, D.Reinen, V.M.Masters, M.J.Riley (2011) *Inorg.Chem.* ,**50,**4900
Formula:	$C_6 H_{14} Cu_1 O_8$
Compound Name:	trans-Diaqua-bis(2-methoxyacetato-O,O')-copper(ii)

Space Group:	P21/n	***Cell:***	***a***	6.934(0)	***b***	10.038(0)	***c***	7.216(0)
Space Group No.:	14	***(Å,°)***	α	90.00	β	96.19(0)	γ	90.00

R-Factor (%): 2.05 ***Temperature(K):*** 240 ***Density(g/cm³):*** 1.847

Parameters

Fragment 1

**DIST1 (D)**	1.939
**DIST2 (D)**	1.939
**DIST3 (D)**	2.172
**DIST4 (D)**	2.102
**DIST5 (D)**	2.172
**DIST6 (D)**	2.102

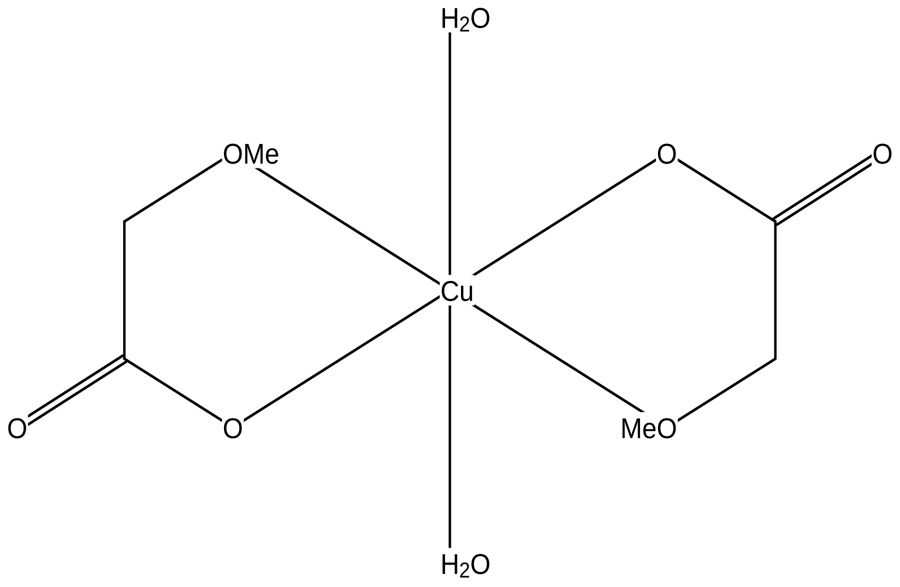

Search: search2 (Wed Jul 26 09:11:25 2017): Hit 32

CUACDH21

Reference:	C.J.Simmons, H.Stratemeier, M.A.Hitchman, D.Reinen, V.M.Masters, M.J.Riley (2011) *Inorg.Chem.* ,**50,**4900
Formula:	$C_6\ H_{14}\ Cu_1\ O_8$
Compound Name:	trans-Diaqua-bis(2-methoxyacetato-O,O')-copper(ii)

Space Group:	P21/n	***Cell:***	***a*** 6.942(0)	***b*** 10.072(0)	***c*** 7.221(0)		
Space Group No.:	14	***(Å,°)***	α 90.00	β 96.34(0)	γ 90.00		
R-Factor (%):	2.20	***Temperature(K):***	260	***Density(g/cm³):***	1.838		

Parameters

Fragment 1	
**DIST1 (D)**	1.937
**DIST2 (D)**	1.937
**DIST3 (D)**	2.163
**DIST4 (D)**	2.114
**DIST5 (D)**	2.163
**DIST6 (D)**	2.114

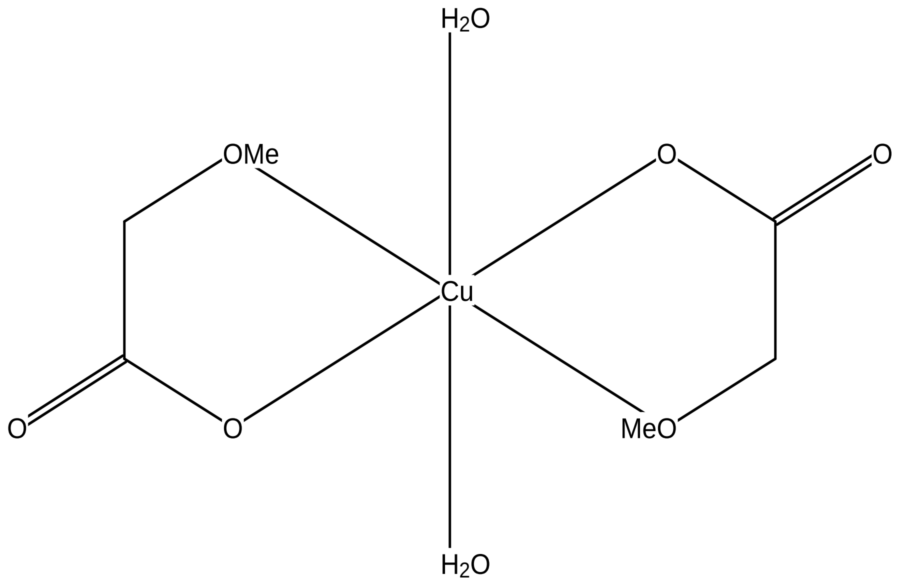

Search: search2 (Wed Jul 26 09:11:25 2017): Hit 33

CUACDH22

Reference: C.J.Simmons, H.Stratemeier, M.A.Hitchman, D.Reinen, V.M.Masters, M.J.Riley (2011) *Inorg.Chem.* ,**50,**4900

Formula: $C_6 H_{14} Cu_1 O_8$

Compound Name: trans-Diaqua-bis(2-methoxyacetato-O,O')-copper(ii)

Space Group:	P21/n	***Cell:***	***a*** 6.953(0)	***b*** 10.110(0)	***c*** 7.227(0)		
Space Group No.:	14	***(Å,°)***	α 90.00	β 96.52(0)	γ 90.00		

R-Factor (%): 2.19 ***Temperature(K):*** 280 ***Density(g/cm^3):*** 1.828

Parameters

Fragment 1

**DIST1 (D)**	1.933
**DIST2 (D)**	1.933
**DIST3 (D)**	2.150
**DIST4 (D)**	2.134
**DIST5 (D)**	2.150
**DIST6 (D)**	2.134

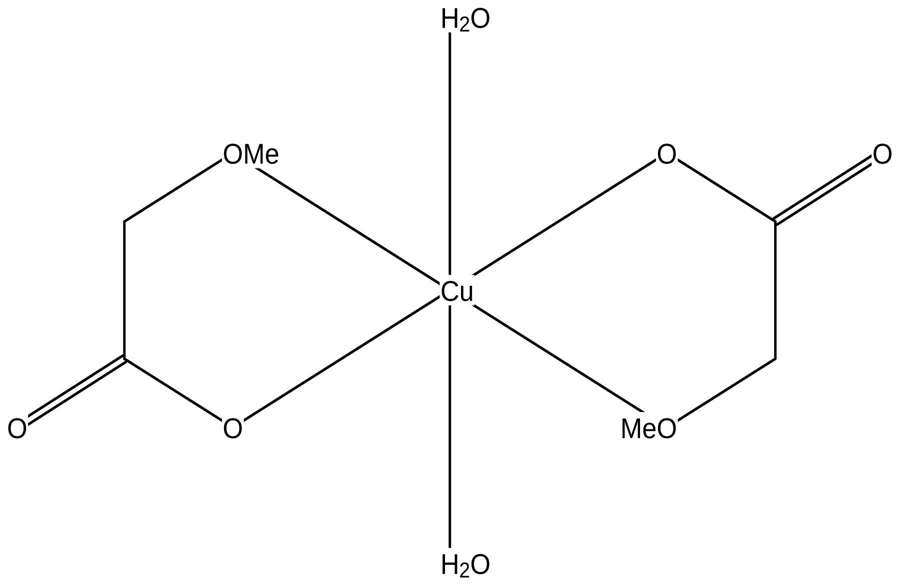

Search: search2 (Wed Jul 26 09:11:25 2017): Hit 34

CUACDH23

Reference:	C.J.Simmons, H.Stratemeier, M.A.Hitchman, D.Reinen, V.M.Masters, M.J.Riley (2011) *Inorg.Chem.* ,**50,**4900
Formula:	C_6 H_{14} Cu_1 O_8
Compound Name:	trans-Diaqua-bis(2-methoxyacetato-O,O')-copper(ii)

Space Group:	P21/n	***Cell:***	***a*** 6.960(0)	***b*** 10.141(0)	***c*** 7.232(0)		
Space Group No.:	14	***(Å,°)***	α 90.00	β 96.67(0)	γ 90.00		
R-Factor (%):	2.17	***Temperature(K):***	298	***Density(g/cm³):***	1.819		

Parameters

Fragment 1

**DIST1 (D)**	1.931
**DIST2 (D)**	1.931
**DIST3 (D)**	2.143
**DIST4 (D)**	2.146
**DIST5 (D)**	2.143
**DIST6 (D)**	2.146

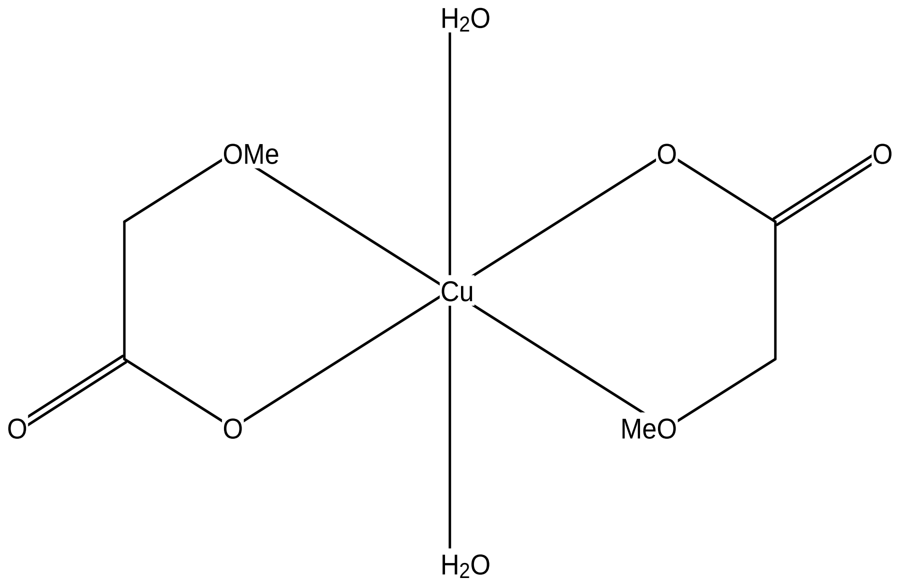

Search: search2 (Wed Jul 26 09:11:25 2017): Hit 35

CUACDH24

Reference:	C.J.Simmons, H.Stratemeier, M.A.Hitchman, D.Reinen, V.M.Masters, M.J.Riley (2011) *Inorg.Chem.* ,**50,**4900
Formula:	$C_6\ H_{14}\ Cu_1\ O_8$
Compound Name:	trans-Diaqua-bis(2-methoxyacetato-O,O')-copper(ii)

Space Group:	P21/n	***Cell:***	***a*** 6.968(0)	***b*** 10.163(0)	***c*** 7.238(0)		
Space Group No.:	14	***(Å,°)***	α 90.00	β 96.78(0)	γ 90.00		
R-Factor (%):	2.30	***Temperature(K):***	310	***Density(g/cm³):***	1.812		

Parameters

Fragment 1	
**DIST1 (D)**	1.930
**DIST2 (D)**	1.930
**DIST3 (D)**	2.138
**DIST4 (D)**	2.159
**DIST5 (D)**	2.138
**DIST6 (D)**	2.159

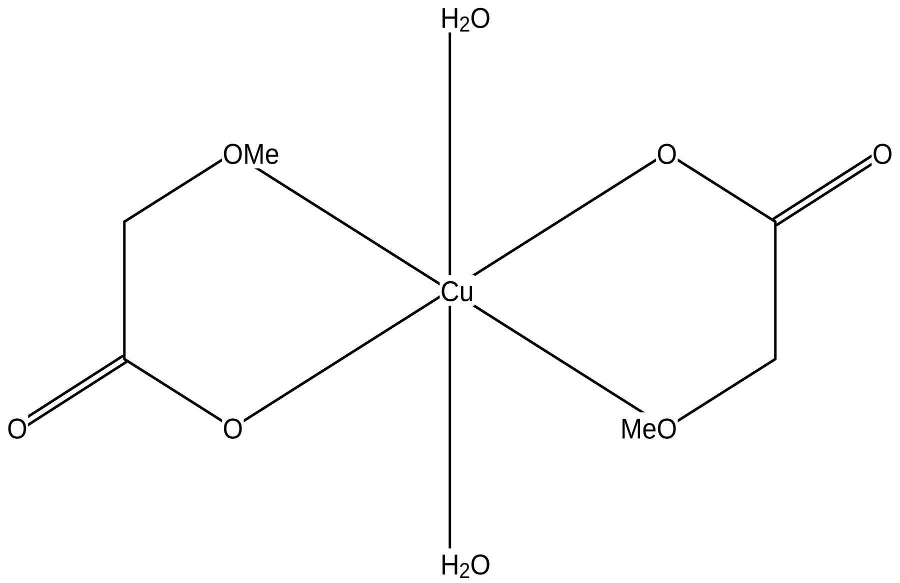

Search: search2 (Wed Jul 26 09:11:25 2017): Hit 36

CUACDH26

Reference:	C.J.Simmons, H.Stratemeier, M.A.Hitchman, D.Reinen, V.M.Masters, M.J.Riley (2011) *Inorg.Chem.* ,**50,**4900
Formula:	$C_6 H_{14} Cu_1 O_8$
Compound Name:	trans-Diaqua-bis(2-methoxyacetato-O,O')-copper(ii)

Space Group:	P21/n	***Cell:***	***a*** 6.980(0)	***b*** 10.201(0)	***c*** 7.247(0)		
Space Group No.:	14	***(Å,°)***	α 90.00	β 96.98(0)	γ 90.00		
R-Factor (%):	2.37	***Temperature(K):***	330	***Density(g/cm^3):***	1.801		

Parameters

Fragment 1

**DIST1 (D)**	1.927
**DIST2 (D)**	1.927
**DIST3 (D)**	2.126
**DIST4 (D)**	2.175
**DIST5 (D)**	2.126
**DIST6 (D)**	2.175

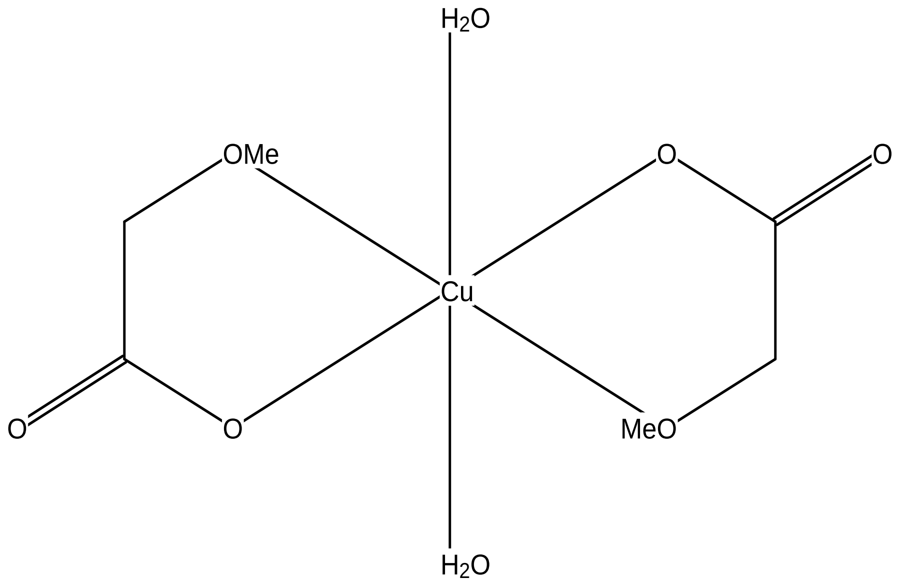

Search: search2 (Wed Jul 26 09:11:25 2017): Hit 37

CUACDH27

Reference:	C.J.Simmons, H.Stratemeier, M.A.Hitchman, D.Reinen, V.M.Masters, M.J.Riley (2011) *Inorg.Chem.* ,**50,**4900
Formula:	$C_6 H_{14} Cu_1 O_8$
Compound Name:	trans-Diaqua-bis(2-methoxyacetato-O,O')-copper(ii)

Space Group:	P21/n	***Cell:***	***a*** 6.986(0)	***b*** 10.218(0)	***c*** 7.252(0)		
Space Group No.:	14	***(Å,°)***	α 90.00	β 97.07(0)	γ 90.00		
R-Factor (%):	2.30	***Temperature(K):***	340	***Density(g/cm³):***	1.795		

Parameters

Fragment 1

**DIST1 (D)**	1.927
**DIST2 (D)**	1.927
**DIST3 (D)**	2.122
**DIST4 (D)**	2.185
**DIST5 (D)**	2.122
**DIST6 (D)**	2.185

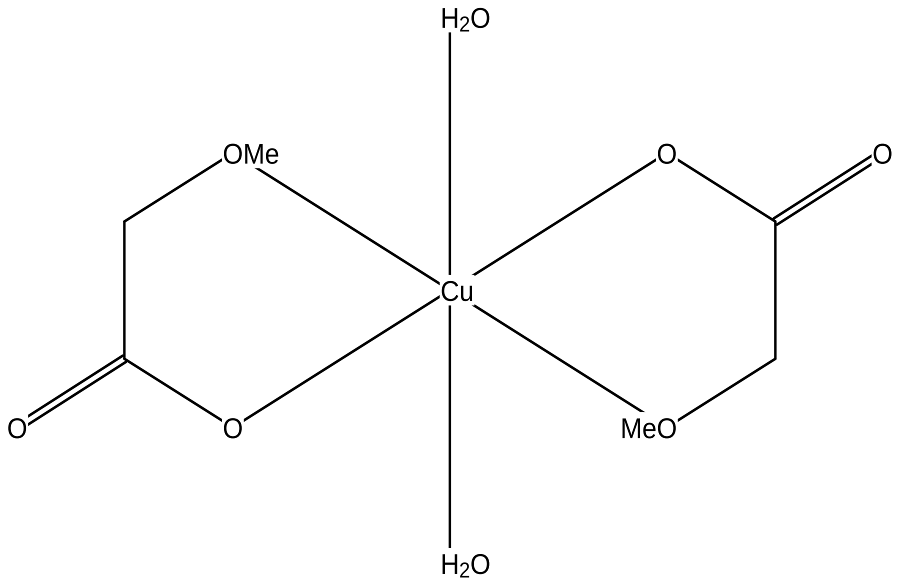

Search: search2 (Wed Jul 26 09:11:25 2017): Hit 38

CUACDH28

Reference:	C.J.Simmons, H.Stratemeier, M.A.Hitchman, D.Reinen, V.M.Masters, M.J.Riley (2011) *Inorg.Chem.* ,**50,**4900
Formula:	$C_6\ H_{14}\ Cu_1\ O_8$
Compound Name:	trans-Diaqua-bis(2-methoxyacetato-O,O')-copper(ii)

Space Group:	P21/n	***Cell:***	***a*** 6.992(0)	***b*** 10.233(0)	***c*** 7.256(0)		
Space Group No.:	14	***(Å,°)***	α 90.00	β 97.17(0)	γ 90.00		
R-Factor (%):	2.40	***Temperature(K):***	350	***Density(g/cm³):***	1.791		

Parameters

Fragment 1

**DIST1 (D)**	1.927
**DIST2 (D)**	1.927
**DIST3 (D)**	2.115
**DIST4 (D)**	2.196
**DIST5 (D)**	2.115
**DIST6 (D)**	2.196

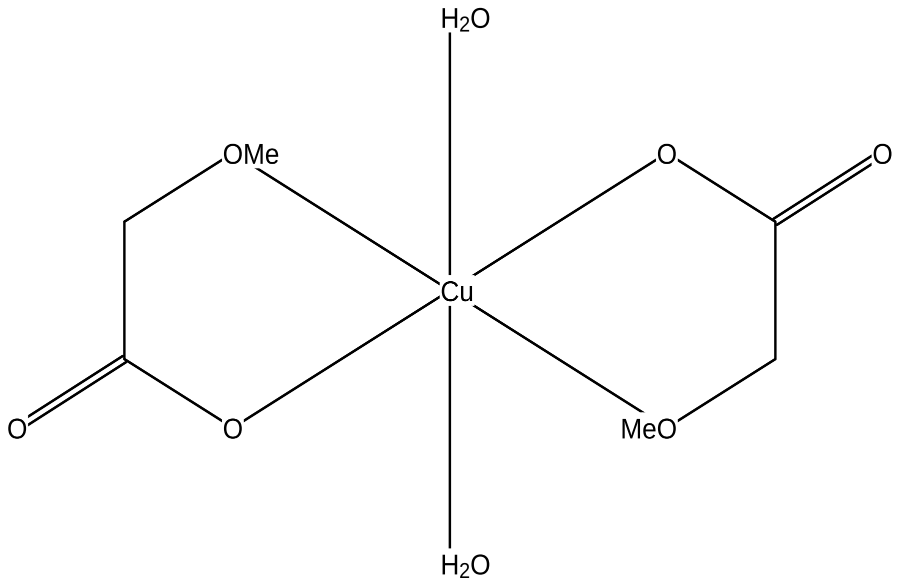

Search: search2 (Wed Jul 26 09:11:25 2017): Hit 39

CUCROC01

Reference:	A.Cornia, A.C.Fabretti, A.Giusti, F.Ferraro, D.Gatteschi (1993) *Inorg.Chim.Acta* ,**212,**87
Formula:	$(C_{10} H_{12} Cu_2 O_{16})n$
Compound Name:	catena-(Tri-aqua-(μ_2-croconato-O,O,O')-copper(ii))

Space Group:	Pbca	***Cell:***	***a*** 11.770(2)	***b*** 8.085(2)	***c*** 15.571(2)		
Space Group No.:	61	***(Å,°)***	α 90.00	β 90.00	γ 90.00		
R-Factor (%):	2.60	***Temperature(K):***	295	***Density(g/cm^3):***	2.310		

Parameters

Fragment 1

**DIST1 (D)**	2.010
**DIST2 (D)**	1.957
**DIST3 (D)**	1.979
**DIST4 (D)**	2.037
**DIST5 (D)**	2.309
**DIST6 (D)**	2.334

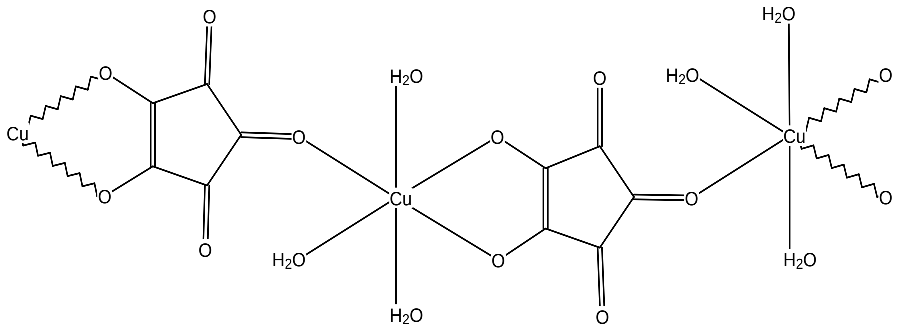

Search: search2 (Wed Jul 26 09:11:25 2017): Hit 40

CUDTAR05

Reference: Yen-Hsiang Liu, Szu-Hsuan Lee, Jung-Chun Chiang, Po-Chen Chen, P.-H.Chien, Chen-I Yang (2013) *Dalton Trans.* ,**42,** 16857

Formula: $(C_8 H_{12} Cu_2 O_{14})n,4(H_2 O_1)$

Compound Name: catena-((μ_4-(R,R)-Tartrato)-(μ_2-(R,R)-tartrato)-diaqua-di-copper(ii) tetrahydrate)

Space Group:	P21	***Cell:***	***a***	8.350(1)	***b***	8.747(1)	***c***	12.039(1)
Space Group No.:	4	***(Å,°)***	α	90.00	β	104.28(0)	γ	90.00

R-Factor (%): 2.33 ***Temperature(K):*** 200 ***Density(g/cm³):*** 2.071

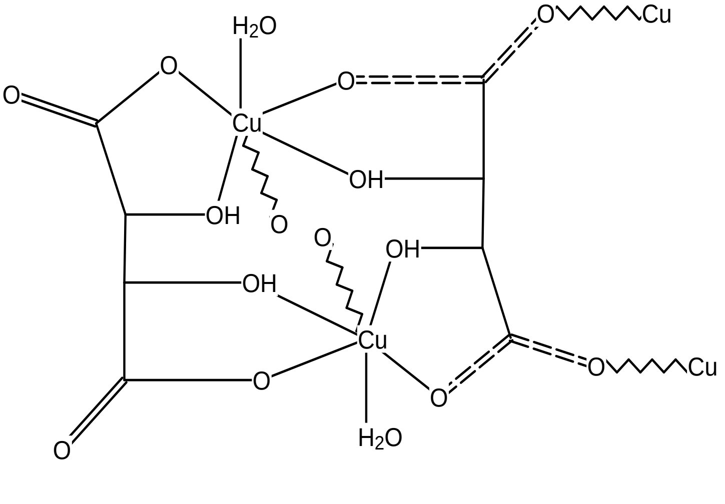

Parameters

Fragment 1

**DIST1 (D)**	1.941
**DIST2 (D)**	1.920
**DIST3 (D)**	2.007
**DIST4 (D)**	1.971
**DIST5 (D)**	2.423
**DIST6 (D)**	2.329

Fragment 2

**DIST1 (D)**	1.932
**DIST2 (D)**	1.939
**DIST3 (D)**	2.019
**DIST4 (D)**	1.969
**DIST5 (D)**	2.402
**DIST6 (D)**	2.369

H_2O

Search: search2 (Wed Jul 26 09:11:25 2017): Hit 41

CUFOHY02

Reference: M.I.Kay (1975) *Ferroelectrics* ,**9,**171

Formula: $(C_2 H_6 Cu_1 O_6)n,2n(H_2 O_1)$

Compound Name: catena-(Diaqua-bis(μ_2-formato-O,O')-copper(ii) dihydrate)

Space Group:	P21/a	***Cell:***	***a*** 8.150	***b*** 8.180	***c*** 6.350
Space Group No.:	14	***(Å,°)***	α 90.00	β 101.08	γ 90.00

R-Factor (%): 2.30 ***Temperature(K):*** 295 ***Density(g/cm^3):*** 1.804

Parameters

Fragment 1

**DIST1 (D)**	1.999
**DIST2 (D)**	1.965
**DIST3 (D)**	1.965
**DIST4 (D)**	1.999
**DIST5 (D)**	2.401
**DIST6 (D)**	2.401

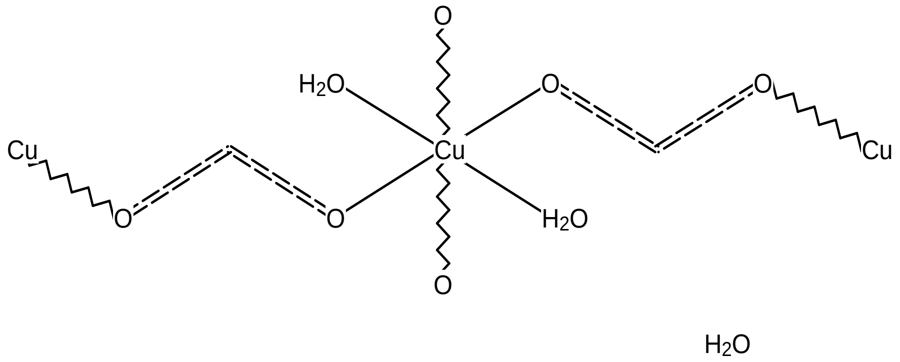

Search: search2 (Wed Jul 26 09:11:25 2017): Hit 42

CUFOHY06

Reference: N.Burger, H.Fuess (1979) *Ferroelectrics* ,**22,**847

Formula: $(C_2 D_6 Cu_1 O_6)n,2n(D_2 O_1)$

Compound Name: catena-(bis(Dideutero-aqua)-bis(μ_2-deutero-formato-O,O')-copper) bis(deuterium oxide) solvate

Space Group:	P21/a	***Cell:***	***a*** 8.184(4)	***b*** 8.137(4)	***c*** 6.323(4)
Space Group No.:	14	***(Å,°)***	α 90.00	β 100.79(4)	γ 90.00

R-Factor (%): 2.20 ***Temperature(K):*** 296 ***Density(g/cm^3):*** 1.891

Parameters

Fragment 1

**DIST1 (D)**	1.995
**DIST2 (D)**	1.964
**DIST3 (D)**	1.964
**DIST4 (D)**	1.995
**DIST5 (D)**	2.386
**DIST6 (D)**	2.386

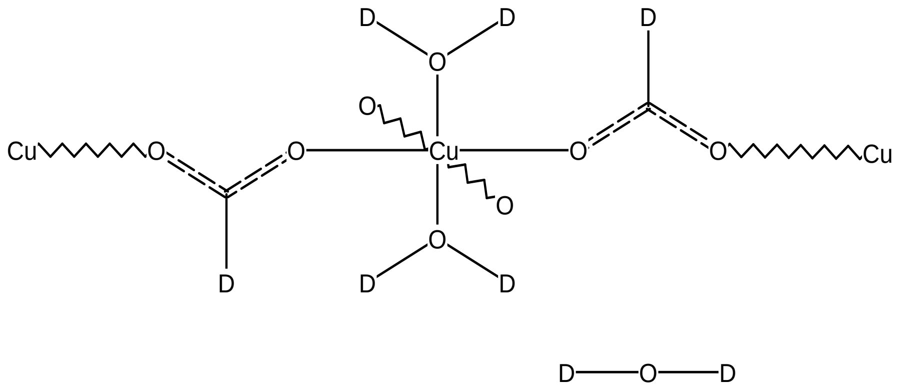

Search: search2 (Wed Jul 26 09:11:25 2017): Hit 43

CUGLYC11

Reference: Qing-Song Ye, Ming-Jin Xie, Wei-Ping Liu, Xi-Zhu Chen, Qiao-Wen Chang, Yao Yu (2010) *Z.Kristallogr.-New Cryst.Struct.* ,**225,** 481

Formula: $(C_4 H_6 Cu_1 O_6)n$

Compound Name: catena-(bis(μ_2-Glycolato)-copper(ii))

Space Group:	P21/n	***Cell:***	***a*** 5.110(0)	***b*** 8.677(1)	***c*** 7.746(1)
Space Group No.:	14	***(Å,°)***	α 90.00	β 106.84(0)	γ 90.00

R-Factor (%): 2.58 ***Temperature(K):*** 293 ***Density(g/cm^3):*** 2.159

Parameters

Fragment 1

**DIST1 (D)**	1.932
**DIST2 (D)**	1.919
**DIST3 (D)**	1.919
**DIST4 (D)**	1.932
**DIST5 (D)**	2.546
**DIST6 (D)**	2.546

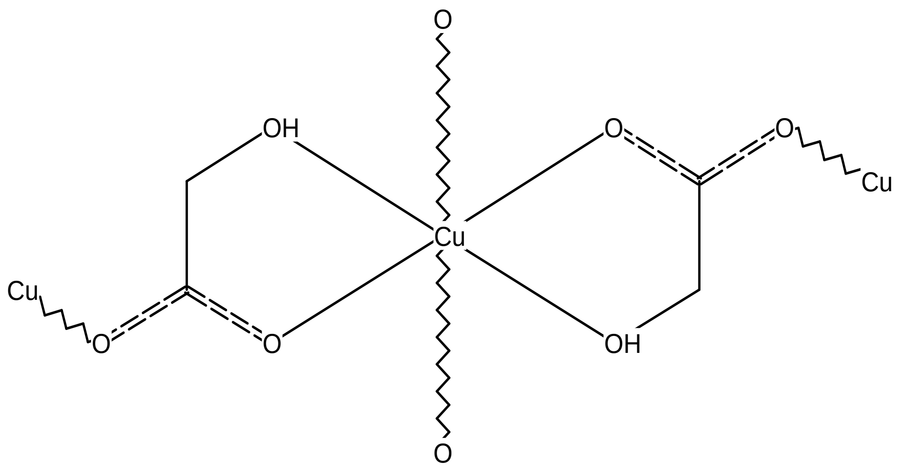

Search: search2 (Wed Jul 26 09:11:25 2017): Hit 44

CUGLYC13

Reference: S.Yoneyama, T.Kodama, K.Kikuchi, Y.Kawabata, K.Kikuchi, T.Ono, Y.Hosokoshi, W.Fujita (2013) *CrystEngComm* ,**15,** 10193

Formula: $(C_4 H_6 Cu_1 O_6)n$

Compound Name: catena-(bis(μ_2-Glycolato)-copper(ii))

Space Group:	P21/n	***Cell:***	***a***	5.091(0)	***b***	8.694(1)	***c***	7.730(1)
Space Group No.:	14	***(Å,°)***	α	90.00	β	107.14(0)	γ	90.00

R-Factor (%): 2.55 ***Temperature(K):*** 249 ***Density(g/cm³):*** 2.170

Parameters

Fragment 1

**DIST1 (D)**	1.935
**DIST2 (D)**	1.922
**DIST3 (D)**	1.922
**DIST4 (D)**	1.935
**DIST5 (D)**	2.529
**DIST6 (D)**	2.529

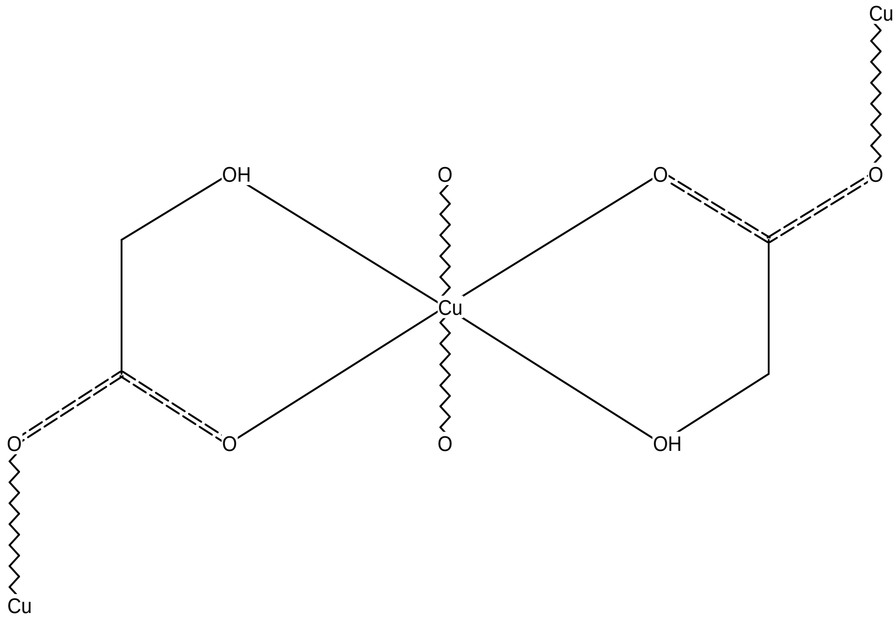

Search: search2 (Wed Jul 26 09:11:25 2017): Hit 45

CUHMAM01

Reference: R.V.Gudavarthy, N.Burla, E.A.Kulp, S.J.Limmer, E.Sinn, J.A.Switzer (2011) *J.Mater.Chem.* ,**21,**6209

Formula: $(C_8 H_{10} Cu_1 O_{10})n,2n(H_2 O_1)$

Compound Name: catena-(bis(μ_2-D-Hydrogen malato)-copper(ii) dihydrate)

Space Group:	P21	***Cell:***	***a*** 7.591(6)	***b*** 10.230(8)	***c*** 8.184(6)		
Space Group No.:	4	***(Å,°)***	α 90.00	β 92.80(1)	γ 90.00		
R-Factor (%):	2.40	***Temperature(K):***	298	***Density(g/cm³):***	1.913		

Parameters

Fragment 1

**DIST1 (D)**	1.913
**DIST2 (D)**	1.951
**DIST3 (D)**	1.970
**DIST4 (D)**	1.940
**DIST5 (D)**	2.683
**DIST6 (D)**	2.338

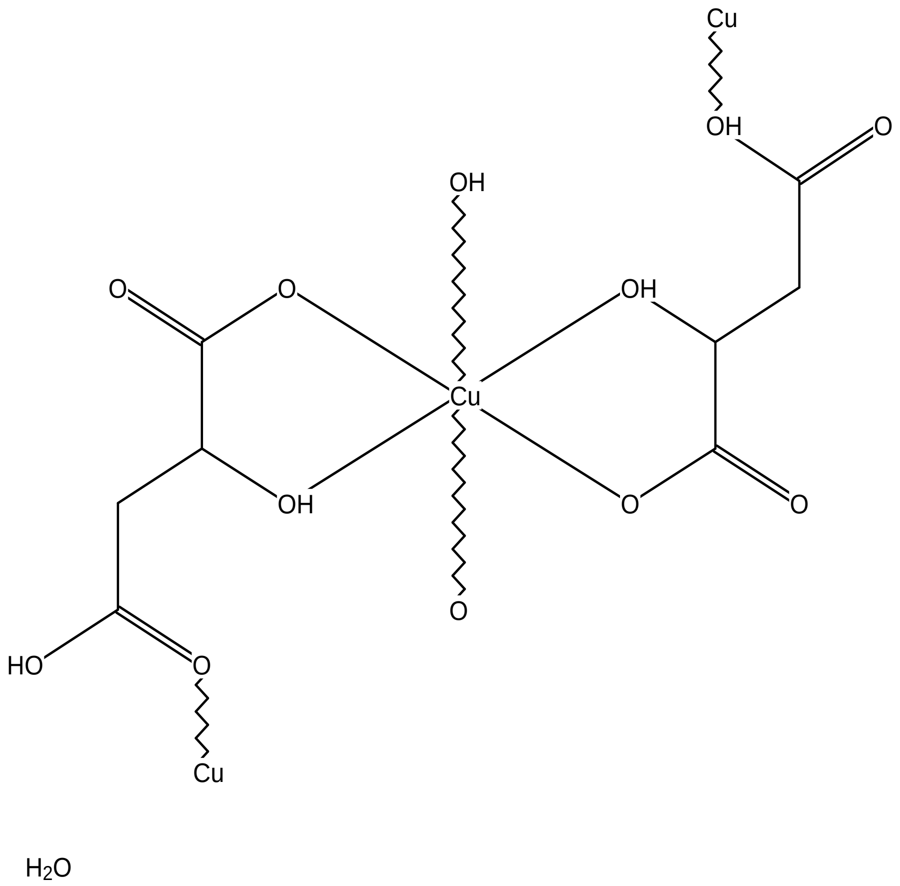

Search: search2 (Wed Jul 26 09:11:25 2017): Hit 46

CUHOPT03

Reference:	H.Bartl, H.Kuppers (1980)<br>*Z.Kristallogr.,Kristallgeom.,Kristallphys.,Kristallchem.* ,**152,**161
Formula:	C_{16} H_{14} Cu_1 O_{10}
Compound Name:	bis(Hydrogen phthalato-O,O')-diaqua-copper(ii)

Space Group:	P21/c	***Cell:***	***a*** 8.360(2)	***b*** 14.395(4)	***c*** 7.086(2)		
Space Group No.:	14	***(Å,°)***	α 90.00	β 112.18(5)	γ 90.00		
R-Factor (%):	2.90	***Temperature(K):***	295	***Density(g/cm³):***	1.808		

Parameters

Fragment 1

**DIST1 (D)**	1.931
**DIST2 (D)**	1.925
**DIST3 (D)**	1.925
**DIST4 (D)**	1.931
**DIST5 (D)**	2.637
**DIST6 (D)**	2.637

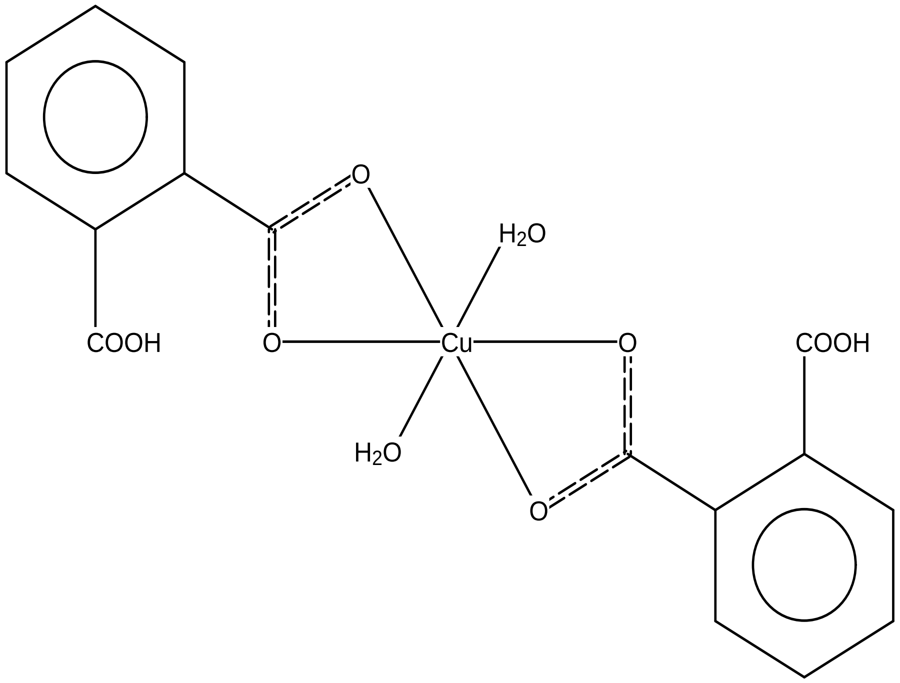

Search: search2 (Wed Jul 26 09:11:25 2017): Hit 47

CUXZEB

Reference: T.D.Keene, I.Zimmermann, A.Neels, O.Sereda, J.Hauser, Shi-Xia Liu, S.Decurtins (2010) *Cryst.Growth Des.* ,**10,**1854

Formula: $2(C_7 H_{10} N_1{}^{1+}),C_6 H_8 Cu_1 O_{10}{}^{2-}$

Compound Name: bis(Benzylammonium) diaqua-bis(malonato)-copper(ii)

Space Group:	P212121	***Cell:***	***a*** 8.458(0)	***b***	11.712(0)	***c***	22.049(1)
Space Group No.:	19	***(Å,°)***	α 90.00	β	90.00	γ	90.00

R-Factor (%): 2.75 ***Temperature(K):*** 173 ***Density(g/cm^3):*** 1.581

Parameters

Fragment 1

**DIST1 (D)**	1.941
**DIST2 (D)**	1.938
**DIST3 (D)**	1.941
**DIST4 (D)**	1.936
**DIST5 (D)**	2.406
**DIST6 (D)**	2.691

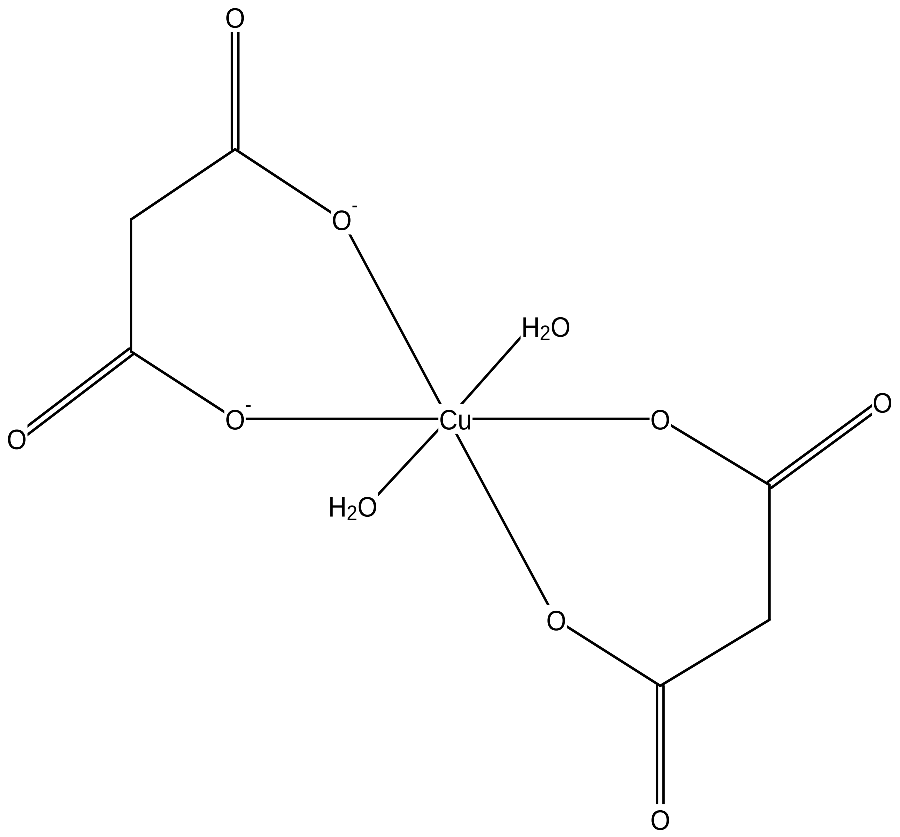

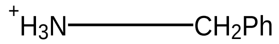

Search: search2 (Wed Jul 26 09:11:25 2017): Hit 48

CUXZOL

Reference:	T.D.Keene, I.Zimmermann, A.Neels, O.Sereda, J.Hauser, Shi-Xia Liu, S.Decurtins (2010) *Cryst.Growth Des.* ,**10,**1854
Formula:	2(C_8 H_{12} N_1 $^{1+}$),C_6 H_8 Cu_1 O_{10} $^{2-}$
Compound Name:	bis(4-Methylbenzylammonium) diaqua-bis(malonato)-copper(ii)

Space Group:	P-1	***Cell:***	***a*** 8.448(0)	***b*** 11.675(1)	***c*** 13.117(1)		
Space Group No.:	2	***(Å,°)***	α 88.50(1)	β 72.58(0)	γ 88.84(1)		
R-Factor (%):	2.83	***Temperature(K):***	173	***Density(g/cm³):***	1.475		

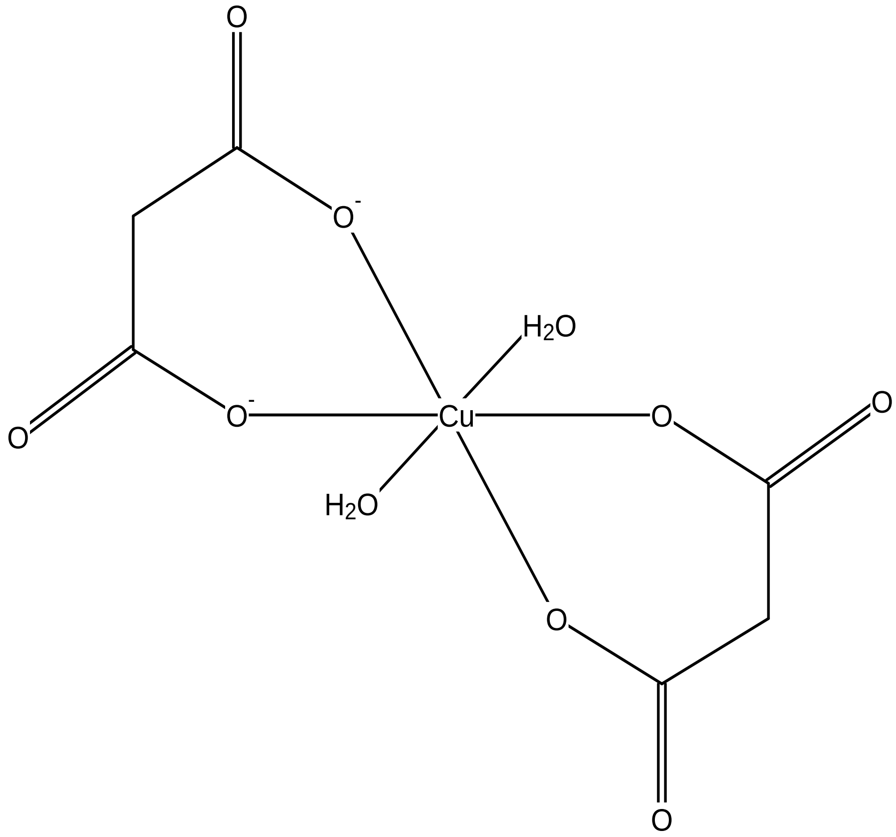

Parameters

Fragment 1

**DIST1 (D)**	1.946
**DIST2 (D)**	1.926
**DIST3 (D)**	1.926
**DIST4 (D)**	1.946
**DIST5 (D)**	2.532
**DIST6 (D)**	2.532

Fragment 2

**DIST1 (D)**	1.941
**DIST2 (D)**	1.943
**DIST3 (D)**	1.943
**DIST4 (D)**	1.941
**DIST5 (D)**	2.527
**DIST6 (D)**	2.527

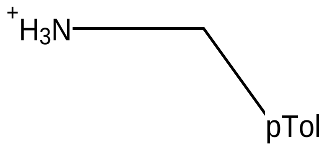

Search: search2 (Wed Jul 26 09:11:25 2017): Hit 49

CUXZUR

Reference:	T.D.Keene, I.Zimmermann, A.Neels, O.Sereda, J.Hauser, Shi-Xia Liu, S.Decurtins (2010) *Cryst.Growth Des.* ,**10,**1854
Formula:	$2(C_8\ H_{10}\ N_1\ O_2\ ^{1+}),C_6\ H_8\ Cu_1\ O_{10}\ ^{2-}$
Compound Name:	bis(4-Carboxybenzylammonium) diaqua-bis(malonato)-copper(ii)

Space Group:	P21/c	***Cell:***	***a***	13.864(0)	***b***	8.245(0)	***c***	11.765(0)
Space Group No.:	14	***(Å,°)***	α	90.00	β	102.54(0)	γ	90.00

R-Factor (%):	2.85	***Temperature(K):***	173	***Density(g/cm^3):***	1.538

Parameters

Fragment 1

**DIST1 (D)**	1.943
**DIST2 (D)**	1.947
**DIST3 (D)**	1.943
**DIST4 (D)**	1.947
**DIST5 (D)**	2.486
**DIST6 (D)**	2.486

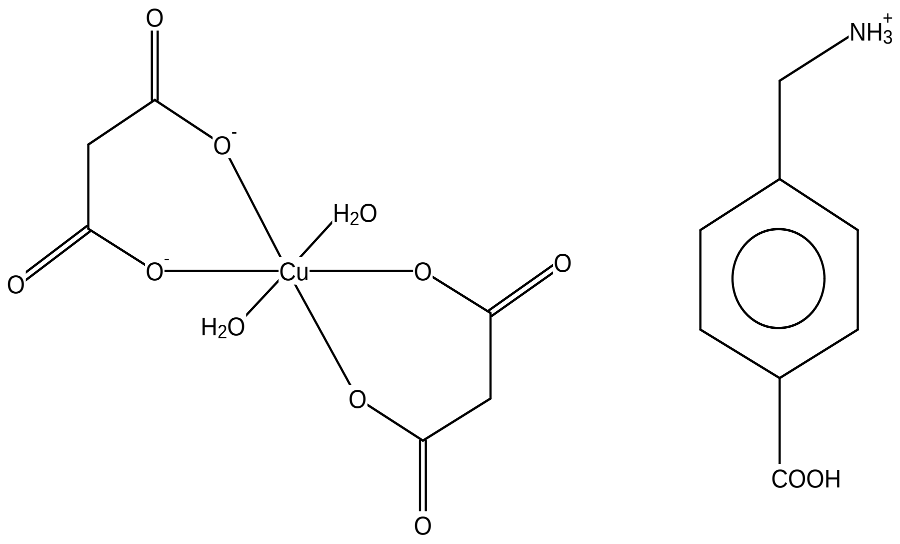

Search: search2 (Wed Jul 26 09:11:25 2017): Hit 50

CUYBII

Reference:	T.D.Keene, I.Zimmermann, A.Neels, O.Sereda, J.Hauser, Shi-Xia Liu, S.Decurtins (2010) *Cryst.Growth Des.* ,**10,**1854
Formula:	$2(C_8\ H_{12}\ N_1\ ^{1+}),C_6\ H_8\ Cu_1\ O_{10}\ ^{2-}$
Compound Name:	bis(Phenylethylammonium) diaqua-bis(malonato)-copper(ii)

Space Group:	P21	***Cell:***	***a*** 11.426(1)	***b*** 8.356(0)	***c*** 12.868(1)
Space Group No.:	4	***(Å,°)***	α 90.00	β 95.21(1)	γ 90.00
R-Factor (%):	2.74	***Temperature(K):*** 173		***Density(g/cm³):*** 1.488	

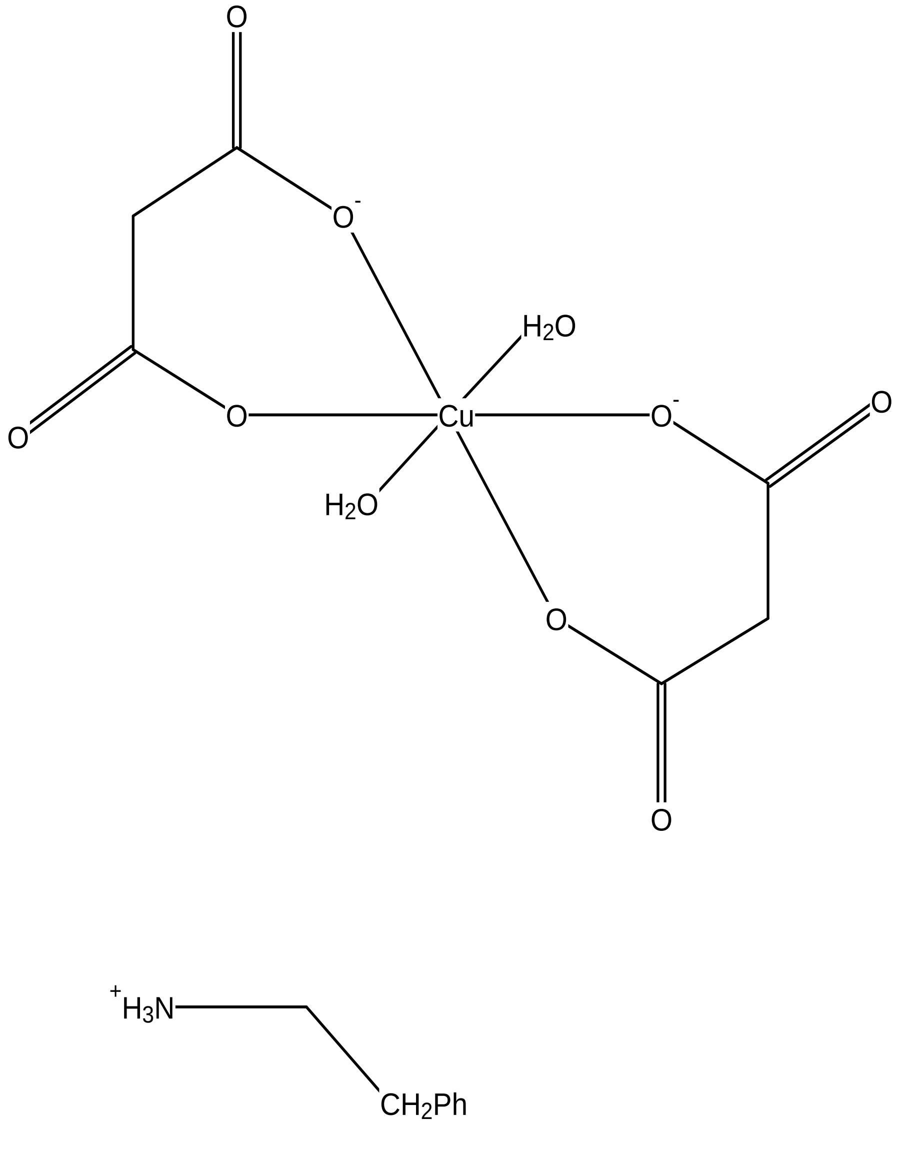

Parameters

Fragment 1

**DIST1 (D)**	1.938
**DIST2 (D)**	1.961
**DIST3 (D)**	1.952
**DIST4 (D)**	1.967
**DIST5 (D)**	2.421
**DIST6 (D)**	2.380

Search: search2 (Wed Jul 26 09:11:25 2017): Hit 51

DAQNUE

Reference:	Qinglin He, Enbo Wang (1999) *Inorg.Chem.Commun.* ,**2,** 399						
Formula:	2($C_5 H_6 N_1{}^{1+}$),2($H_3 O_1{}^{1+}$),$As_6 Cu_1 Mo_6 O_{30}{}^{4-}$						
Compound Name:	bis(Pyridinium) dioxonium hexakis(μ_4-oxo)-dodecakis(μ_2-oxo)-dodecaoxo-arsenic(iii)-copper(ii)-molybdenum(vi)						
Space Group:	P21/n	***Cell:***	***a*** 9.303(2)	***b*** 20.731(4)	***c*** 12.617(3)		
Space Group No.:	14	***(Å,°)***	α 90.00	β 104.17(3)	γ 90.00		
R-Factor (%):	2.69	***Temperature(K):***	295	***Density(g/cm³):***	2.487		

Parameters

Fragment 1

**DIST1 (D)**	2.069
**DIST2 (D)**	2.069
**DIST3 (D)**	2.048
**DIST4 (D)**	2.048
**DIST5 (D)**	2.051
**DIST6 (D)**	2.051

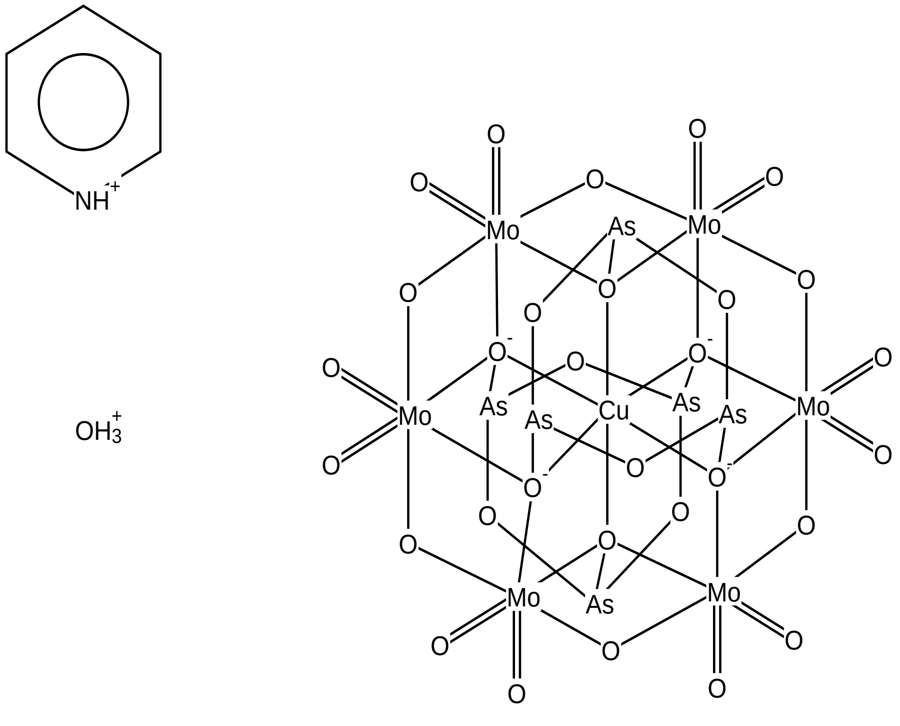

Search: search2 (Wed Jul 26 09:11:25 2017): Hit 52

DICWER

Reference: B.L.Rodrigues, M.D.D.Costa, N.G.Fernandes (1999)
Acta Crystallogr.,Sect.C:Cryst.Struct.Commun. ,**55,**1997

Formula: $(C_{16} H_{14} Cu_1 O_{10})n$

Compound Name: catena-[β-(bis(μ_2-Hydrogenphthalato-O,O')-diaqua-copper(ii))]

Space Group:	P21/c	***Cell:***	***a***	13.177(2)	***b***	5.111(1)	***c***	12.972(2)
Space Group No.:	14	***(Å,°)***	α	90.00	β	116.63(1)	γ	90.00

R-Factor (%): 2.90 ***Temperature(K):*** 295 ***Density(g/cm^3):*** 1.828

Parameters

Fragment 1

**DIST1 (D)**	1.974
**DIST2 (D)**	1.974
**DIST3 (D)**	1.944
**DIST4 (D)**	1.944
**DIST5 (D)**	2.461
**DIST6 (D)**	2.461

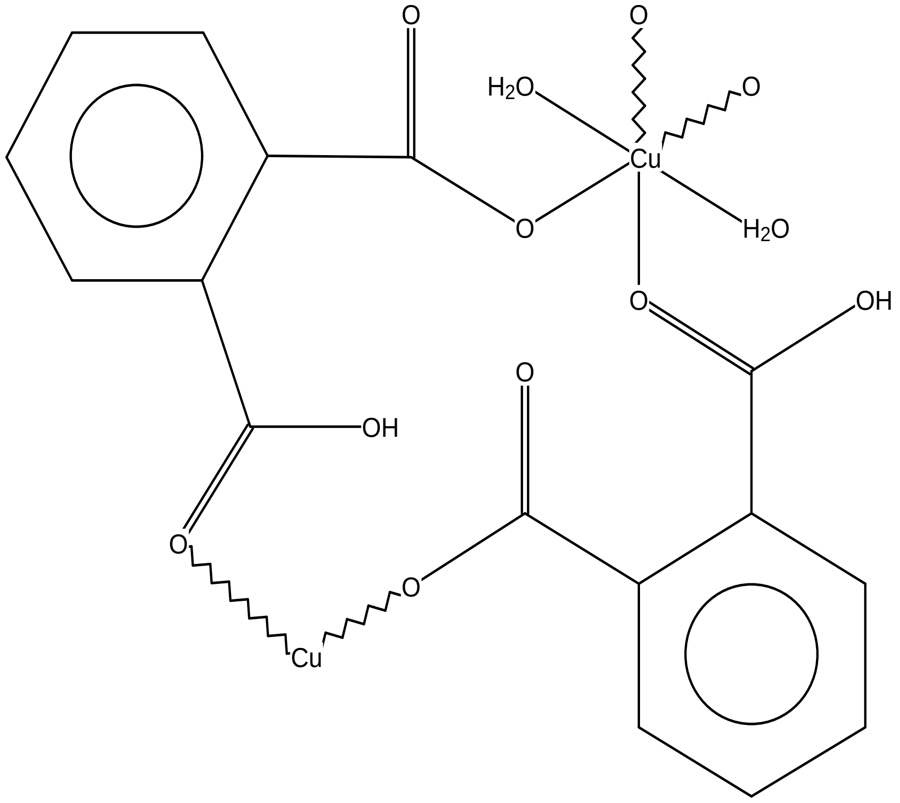

Search: search2 (Wed Jul 26 09:11:25 2017): Hit 53

DILCIL

Reference: Yin-Qiu Liu, Xi-Rui Zeng (2007)
Acta Crystallogr.,Sect.E:Struct.Rep.Online ,**63,**m2414

Formula: $H_{12}\ Cu_1\ O_6\ ^{2+}$,2($C_6\ H_5\ O_4\ S_1\ ^{1-}$)

Compound Name: Hexaaqua-copper(ii) bis(4-hydroxybenzenesulfonate)

Space Group:	P21/n	***Cell:***	***a***	6.930(1)	***b***	6.187(1)	***c***	23.531(4)
Space Group No.:	14	***(Å,°)***	α	90.00	β	91.53(0)	γ	90.00

R-Factor (%): 2.73 ***Temperature(K):*** 293 ***Density(g/cm3):*** 1.706

Parameters

Fragment 1

**DIST1 (D)**	1.942
**DIST2 (D)**	1.965
**DIST3 (D)**	1.942
**DIST4 (D)**	1.965
**DIST5 (D)**	2.384
**DIST6 (D)**	2.384

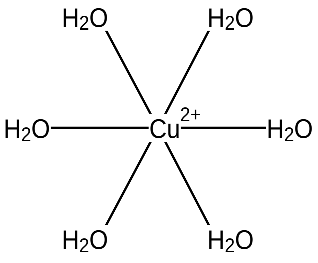

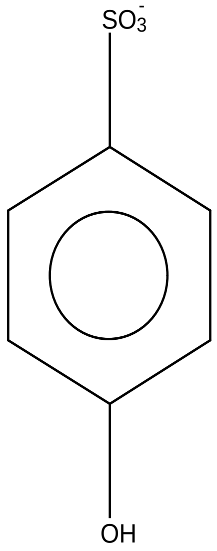

Search: search2 (Wed Jul 26 09:11:25 2017): Hit 54

DIXGIB

Reference:	Zhong-Xiang Du, Jun-Xia Li, Jian-Hua Qin (2008)<br>*Z.Kristallogr.-New Cryst.Struct.* ,**223,**105						
Formula:	$(C_{20}\ H_{24}\ Cu_1\ Na_2\ O_{24})n,2n(H_2\ O_1)$						
Compound Name:	catena-(hexakis(μ_2-aqua)-bis(μ_2-dihydrogen-1,2,4,5-benzenetetracarboxylate-O,O')-diaqua-di-sodium(i)-copper(ii) dihydrate)						
Space Group:	C2/m	***Cell:***	***a*** 7.356(0)	***b*** 20.271(2)	***c*** 10.464(1)		
Space Group No.:	12	***(Å,°)***	α 90.00	β 104.49(0)	γ 90.00		
R-Factor (%):	2.38	***Temperature(K):***	291	***Density(g/cm^3):***	1.746		

Parameters

Fragment 1

**DIST1 (D)**	1.974
**DIST2 (D)**	2.030
**DIST3 (D)**	2.030
**DIST4 (D)**	1.974
**DIST5 (D)**	2.301
**DIST6 (D)**	2.301

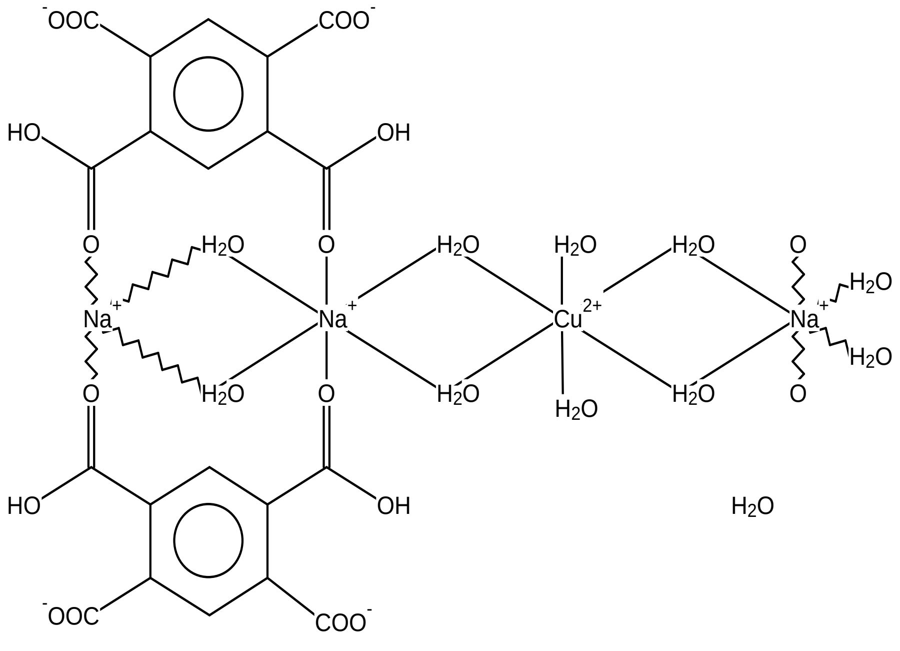

Search: search2 (Wed Jul 26 09:11:25 2017): Hit 55

DIZYER

Reference: G.Steinhauser, K.Karaghiosoff, T.M.Klapotke (2008)
Z.Anorg.Allg.Chem. ,**634,**892

Formula: $2(C_1\ H_6\ N_1\ ^{1+}),Cu_1\ N_4\ O_{12}\ ^{2-}$

Compound Name: bis(Methylammonium) bis(nitrato-O,O')-bis(nitrato-O)-copper(ii)

Space Group:	P21/c	***Cell:***	***a***	8.453(0)	***b***	8.692(0)	***c***	9.394(0)
Space Group No.:	14	***(Å,°)***	α	90.00	β	104.82(0)	γ	90.00

R-Factor (%): 2.39 ***Temperature(K):*** 200 ***Density(g/cm^3):*** 1.870

Parameters

Fragment 1

**DIST1 (D)**	1.957
**DIST2 (D)**	1.967
**DIST3 (D)**	1.967
**DIST4 (D)**	1.957
**DIST5 (D)**	2.527
**DIST6 (D)**	2.527

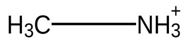

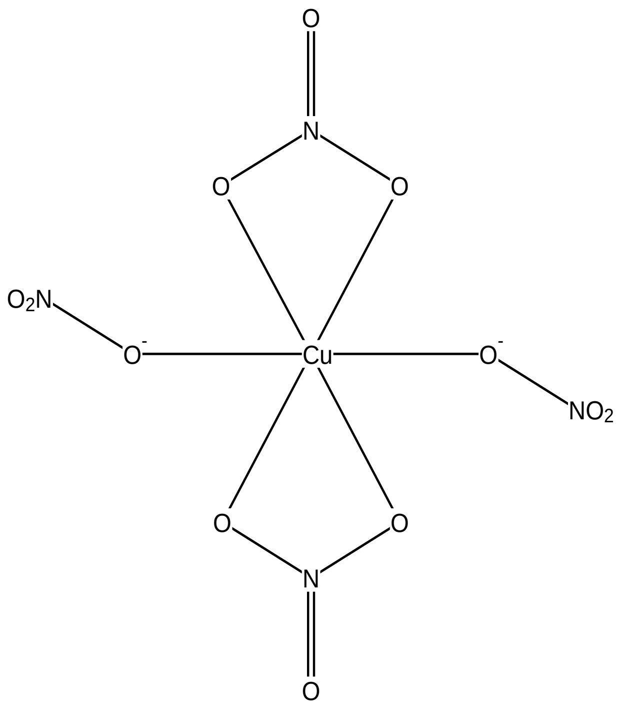

Search: search2 (Wed Jul 26 09:11:25 2017): Hit 56

DODDOP

Reference:	I.I.Zviedre, V.K.Bel'skii, E.M.Shvarts (1985)<br>*Latv.PSR Zinat.Akad.Vestis,Khim.Ser.* ,672
Formula:	$(H_{12}\ Cu_1\ O_6\ ^{2+})n,2n(C_5\ H_8\ B_1\ O_4\ ^{1-}),4n(H_2\ O_1)$
Compound Name:	poly(Penta-erythritol-borate) hexa-aqua-copper(ii) tetrahydrate

Space Group:	P-1	***Cell:***	***a*** 5.636(1)	***b*** 11.155(2)	***c*** 8.847(2)		
Space Group No.:	2	***(Å,°)***	α 109.06(1)	β 104.24(1)	γ 92.60(10)		
R-Factor (%):	2.90	***Temperature(K):***	295	***Density(g/cm³):***	1.742		

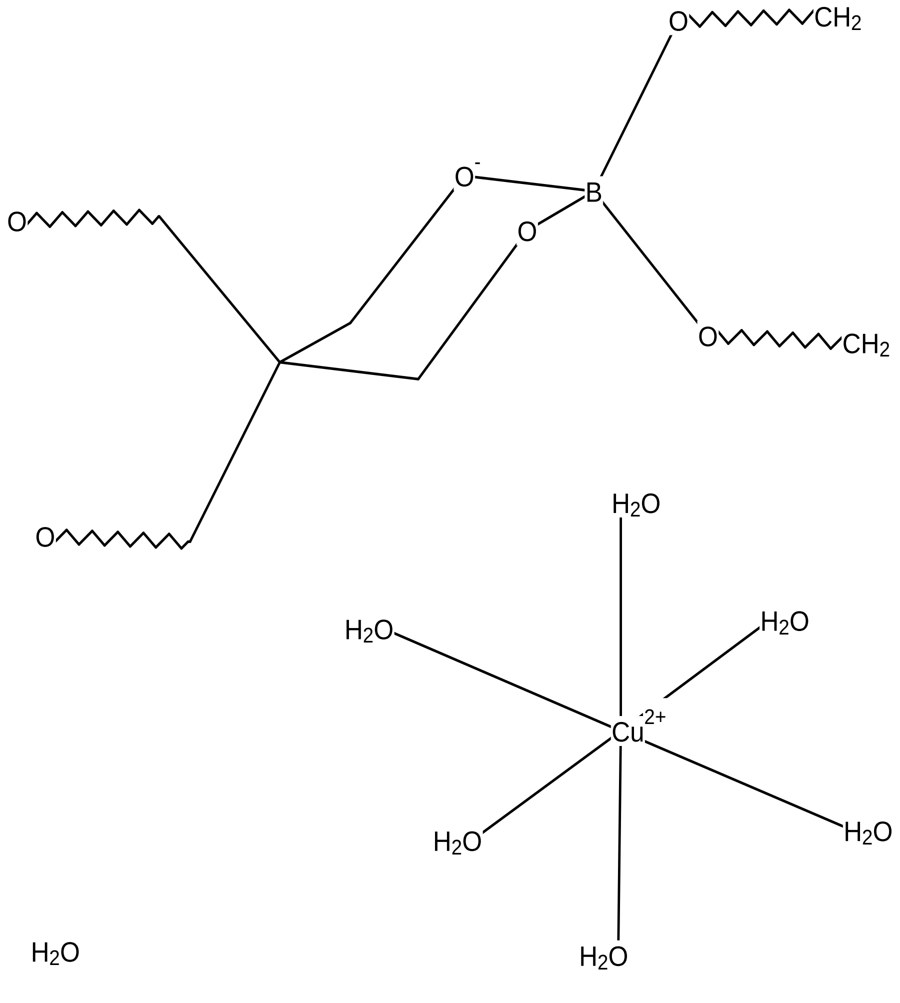

Parameters

Fragment 1

**DIST1 (D)**	1.939
**DIST2 (D)**	1.997
**DIST3 (D)**	1.939
**DIST4 (D)**	1.997
**DIST5 (D)**	2.412
**DIST6 (D)**	2.412

Search: search2 (Wed Jul 26 09:11:25 2017): Hit 57

DOGYUU01

Reference: Y.Li, D.Sun, H.Zang, L.Han, G.Su (2010)
Acta Crystallogr.,Sect.E:Struct.Rep.Online ,**66,**m1576

Formula: $(C_{10}\ H_{18}\ Cu_3\ N_4\ O_{16}\ P_4)n$

Compound Name: catena-(bis(μ_4-1-hydroxy-2-(imidazol-3-ium-1-yl)ethane-1,1-diyldiphosphonato)-diaqua-tri-copper(ii))

Space Group:	P-1	***Cell:***	***a*** 7.417(0)	***b*** 8.150(1)	***c*** 9.523(1)
Space Group No.:	2	***(Å,°)***	α 104.75(0)	β 107.66(0)	γ 101.48(0)

R-Factor (%): 2.74 ***Temperature(K):*** 293 ***Density(g/cm³):*** 2.510

Parameters

Fragment 1

**DIST1 (D)**	1.950
**DIST2 (D)**	1.959
**DIST3 (D)**	1.950
**DIST4 (D)**	1.959
**DIST5 (D)**	2.575
**DIST6 (D)**	2.575

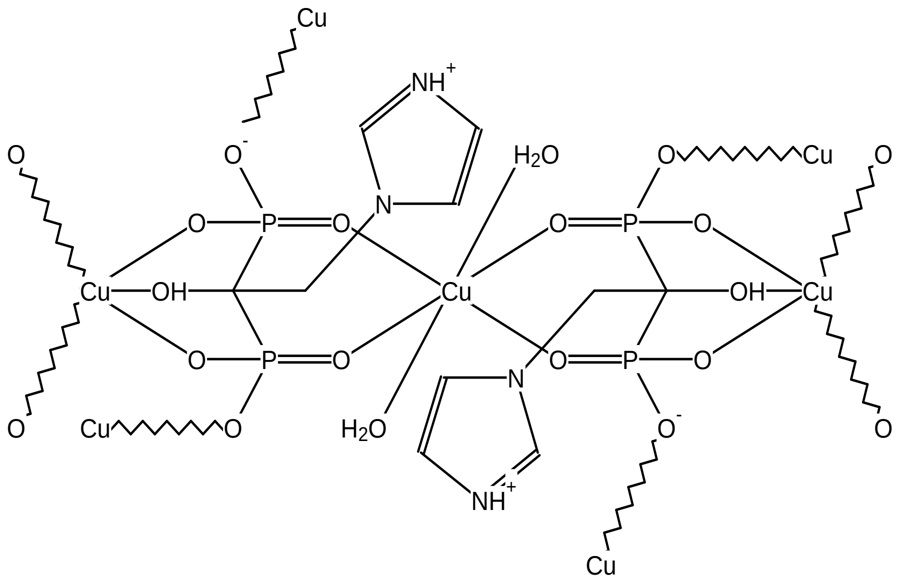

Search: search2 (Wed Jul 26 09:11:25 2017): Hit 58

DOKPOI01

Reference:	N.Lah, P.Segedin, I.Leban (2002) *Acta Chim.Slov.* ,**49,**251								
Formula:	$(C_4\ H_6\ Cu_1\ O_8)n$								
Compound Name:	catena-(bis(μ_2-Formato-O,O')-bis(formic acid-O)-copper(ii))								
Space Group:	Pbca	***Cell:***	***a***	7.877(0)	***b***	8.478(0)	***c***	12.142(0)	
Space Group No.:	61	***(Å,°)***	α	90.00	β	90.00	γ	90.00	
R-Factor (%):	2.24	***Temperature(K):***	150		***Density(g/cm³):***	2.012			

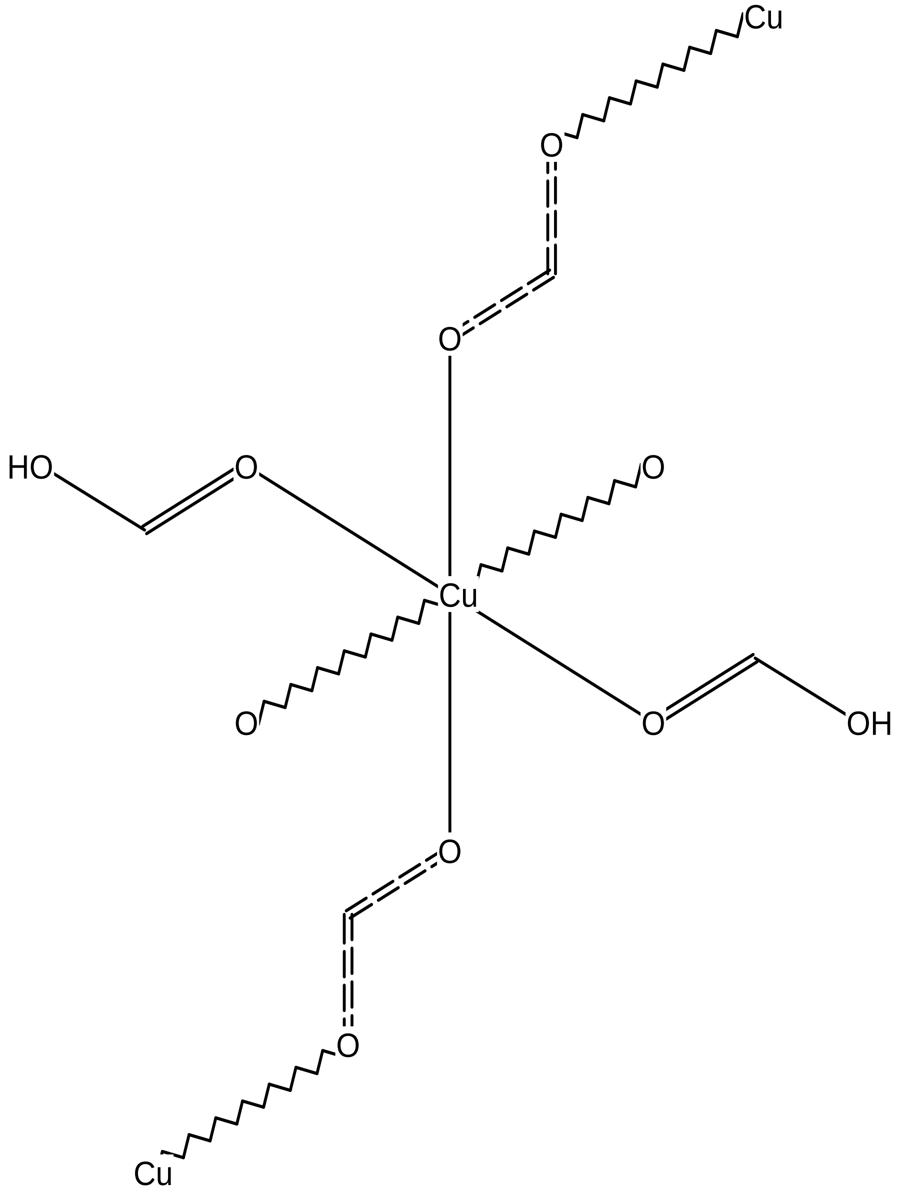

Parameters

Fragment 1

**DIST1 (D)**	1.975
**DIST2 (D)**	1.945
**DIST3 (D)**	1.945
**DIST4 (D)**	1.975
**DIST5 (D)**	2.435
**DIST6 (D)**	2.435

Search: search2 (Wed Jul 26 09:11:25 2017): Hit 59

EJEWAT

Reference: Chun-Hong Chen, Rong Mu, Li Zhang, Guan-Cheng Xu (2016) *CrystEngComm* ,**18,**2719

Formula: $H_8 Cu_1 O_{12} S_2{}^{2-}$,2($C_3 H_{12} N_2{}^{2+}$),2($H_1 O_4 S_1{}^{1-}$)

Compound Name: bis(propane-1,3-diaminium) bis(hydrogen sulfate) tetra-aqua-bis(sulfato)-copper

Space Group:	P-1	***Cell:***	***a*** 10.649(4)	***b*** 10.677(4)	***c*** 10.799(4)
Space Group No.:	2	***(Å,°)***	α 94.77(0)	β 100.76(0)	γ 95.75(0)

R-Factor (%): 2.61 ***Temperature(K):*** 100 ***Density(g/cm^3):*** 1.876

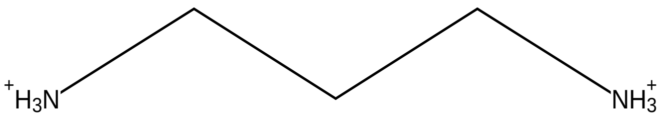

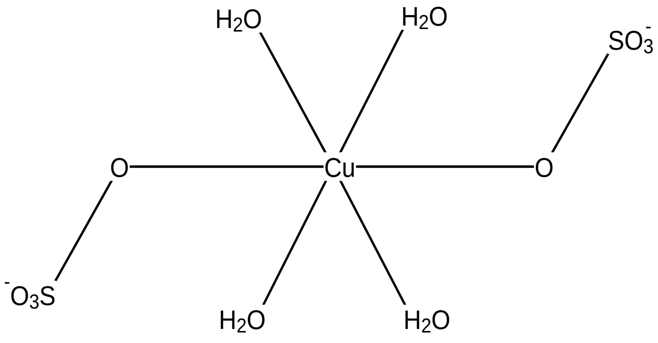

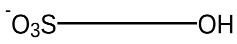

Parameters

Fragment 1

**DIST1 (D)**	1.997
**DIST2 (D)**	1.949
**DIST3 (D)**	1.997
**DIST4 (D)**	1.949
**DIST5 (D)**	2.442
**DIST6 (D)**	2.442

Fragment 2

**DIST1 (D)**	1.996
**DIST2 (D)**	1.948
**DIST3 (D)**	1.996
**DIST4 (D)**	1.948
**DIST5 (D)**	2.472
**DIST6 (D)**	2.472

Search: search2 (Wed Jul 26 09:11:25 2017): Hit 60

ELEHOU

Reference: K.Matelkova, L.Kuckova, A.Maslejova, Jan Moncol, V.Jorik, J.Kozisek (2016) *Chem.Papers* ,**70,**82

Formula: $(C_4\,Cu_1\,O_8\,^{2-})n,2n(K_1\,^{1+})$

Compound Name: catena-(Di-potassium bis(μ_2-oxalato)-copper)

Space Group:	P21/c	***Cell:***	***a***	4.880(0)	***b***	6.652(0)	***c***	13.502(0)
Space Group No.:	14	***(Å,°)***	α	90.00	β	104.50(0)	γ	90.00

R-Factor (%): 1.83 ***Temperature(K):*** 150 ***Density(g/cm³):*** 2.487

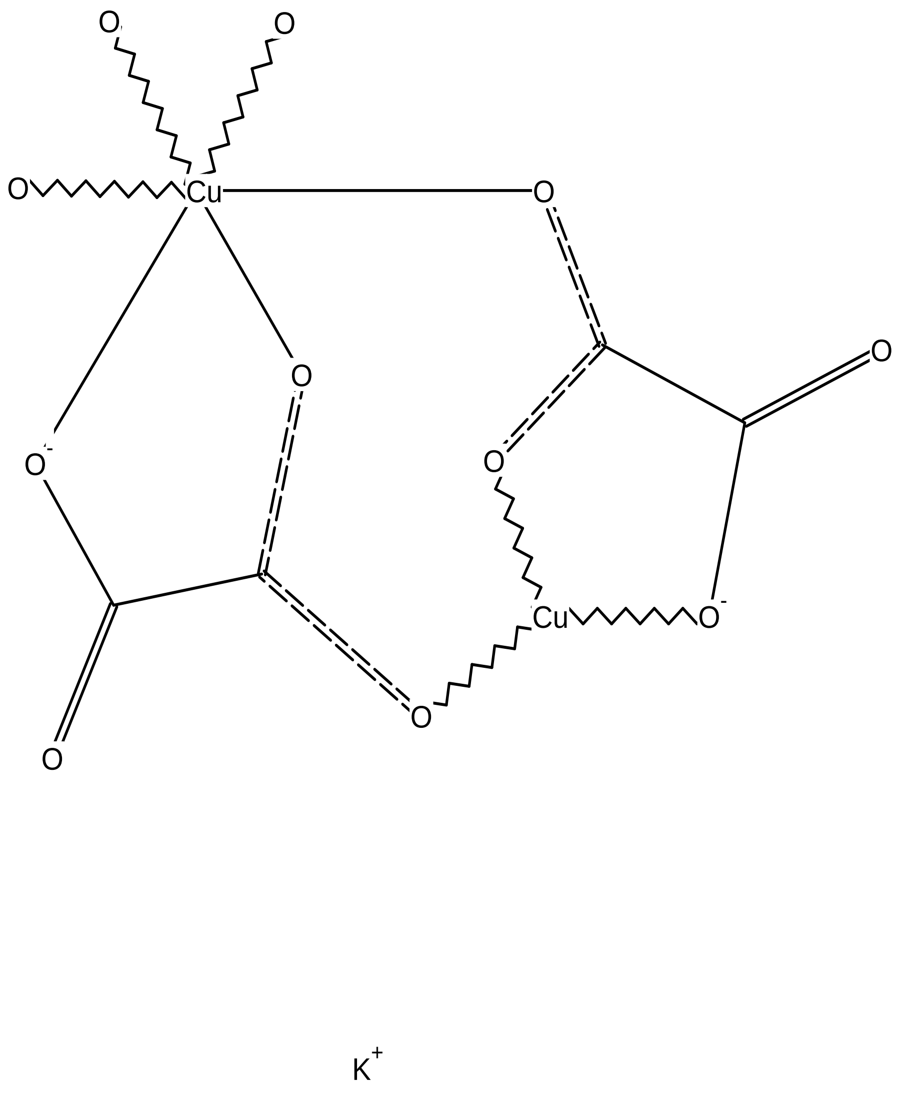

Parameters

Fragment 1

**DIST1 (D)**	1.936
**DIST2 (D)**	1.927
**DIST3 (D)**	1.936
**DIST4 (D)**	1.927
**DIST5 (D)**	2.648
**DIST6 (D)**	2.648

Search: search2 (Wed Jul 26 09:11:25 2017): Hit 61

ELOHES

Reference: F.S.Delgado, J.Sanchiz, C.Ruiz-Perez, F.Lloret, M.Julve (2003) *Inorg.Chem.* ,**42,**5938

Formula: $(C_{12} H_{12} N_2{}^{2+})n,n(C_6 H_4 Cu_1 O_8{}^{2-}),4n(H_2 O_1)$

Compound Name: catena-(1,2-bis(4-pyridinium)ethene bis(μ_2-malonato)-copper(ii) tetrahydrate)

Space Group:	P-1	***Cell:***	***a***	4.883(1)		***b***	9.585(1)		***c***	11.813(2)	
Space Group No.:	2	***(Å,°)***	α	77.29(3)		β	82.18(3)		γ	84.92(3)	

R-Factor (%): 2.81 ***Temperature(K):*** 293 ***Density(g/cm^3):*** 1.631

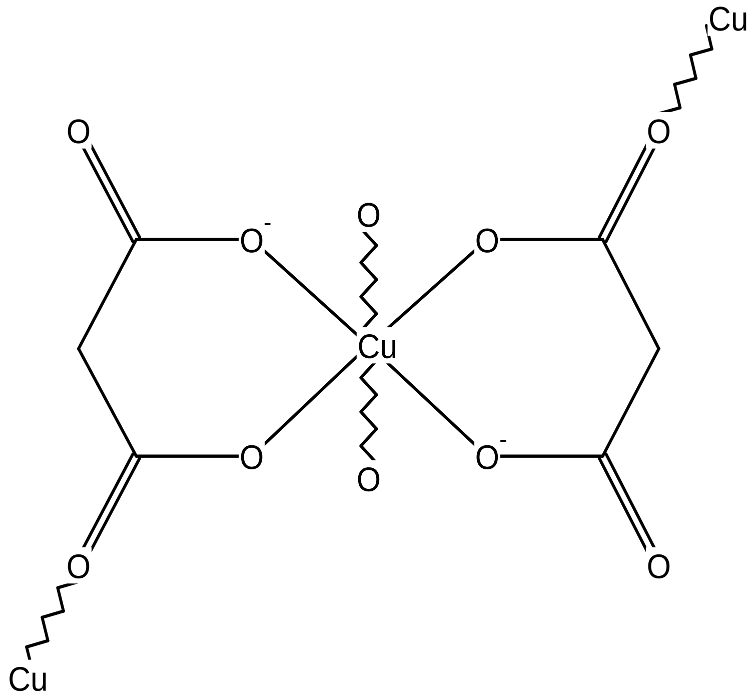

Parameters

Fragment 1

**DIST1 (D)**	1.937
**DIST2 (D)**	1.931
**DIST3 (D)**	1.931
**DIST4 (D)**	1.937
**DIST5 (D)**	2.611
**DIST6 (D)**	2.611

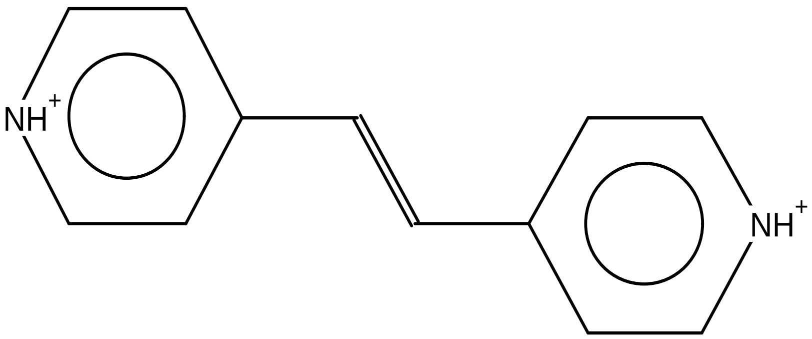

H_2O

Search: search2 (Wed Jul 26 09:11:25 2017): Hit 62

ELUBEU

Reference:	T.G.Horner, P.Klufers (2016) *Eur.J.Inorg.Chem.* ,1798
Formula:	$C_8 H_4 Cu_1 O_{12}{}^{6-}$,12($H_2 O_1$),4($Na_1{}^{1+}$),2($K_1{}^{1+}$)
Compound Name:	di-potassium tetra-sodium bis(L-tartrato)-copper(ii) dodecahydrate
Synonym:	di-potassium tetra-sodium bis(2,3-dioxidobutanedioato)-copper(ii) dodecahydrate

Space Group:	P1	***Cell:***	***a*** 8.973(0)		***b*** 8.983(0)		***c*** 9.393(0)
Space Group No.:	1	***(Å,°)***	α 69.77(0)		β 65.96(0)		γ 76.43(0)
R-Factor (%):	2.21	***Temperature(K):***	100		***Density(g/cm³):***		1.911

Parameters

Fragment 1

**DIST1 (D)**	1.927
**DIST2 (D)**	1.996
**DIST3 (D)**	1.923
**DIST4 (D)**	1.971
**DIST5 (D)**	2.553
**DIST6 (D)**	2.680

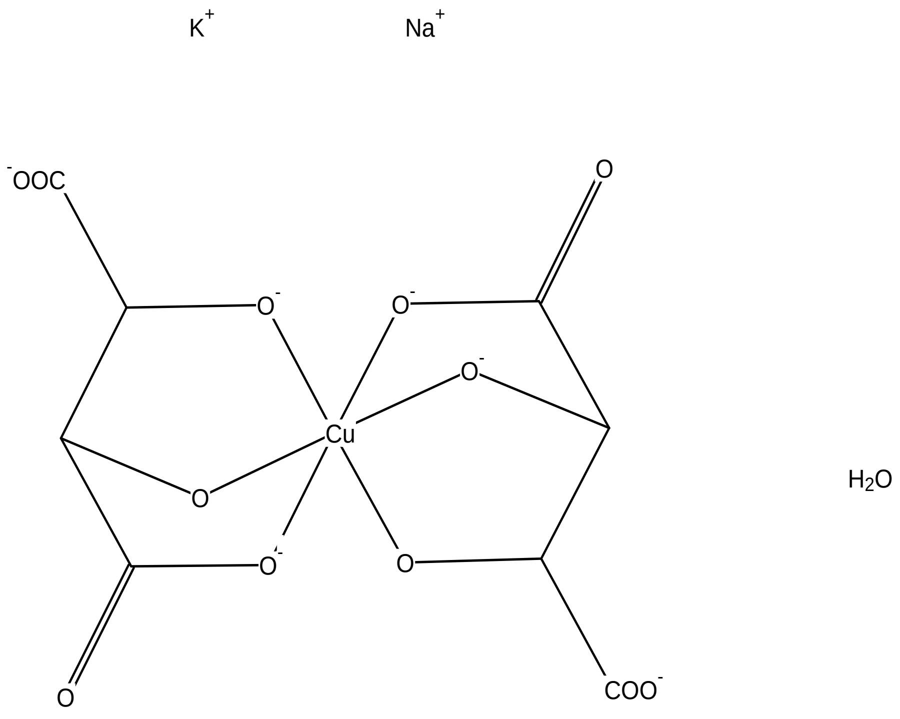

Search: search2 (Wed Jul 26 09:11:25 2017): Hit 63

ENIXUU01

Reference: U.Kolitsch (2004)
Acta Crystallogr.,Sect.C:Cryst.Struct.Commun. ,**60,**m129

Formula: $(C_8 H_8 Cu_2 O_{20} Rb_4)n$

Compound Name: catena-(bis(μ_8-Oxalato)-bis(μ_6-oxalato)-bis(μ_3-aqua)-bis(μ_2-aqua)-di-copper(ii)-tetra-rubidium(i))

Space Group:	P-1	***Cell:***	***a*** 7.000(1)	***b*** 8.949(2)	***c*** 8.982(2)
Space Group No.:	2	***(Å,°)***	α 108.05(3)	β 97.69(3)	γ 97.99(3)

R-Factor (%): 2.41 ***Temperature(K):*** 293 ***Density(g/cm³):*** 2.850

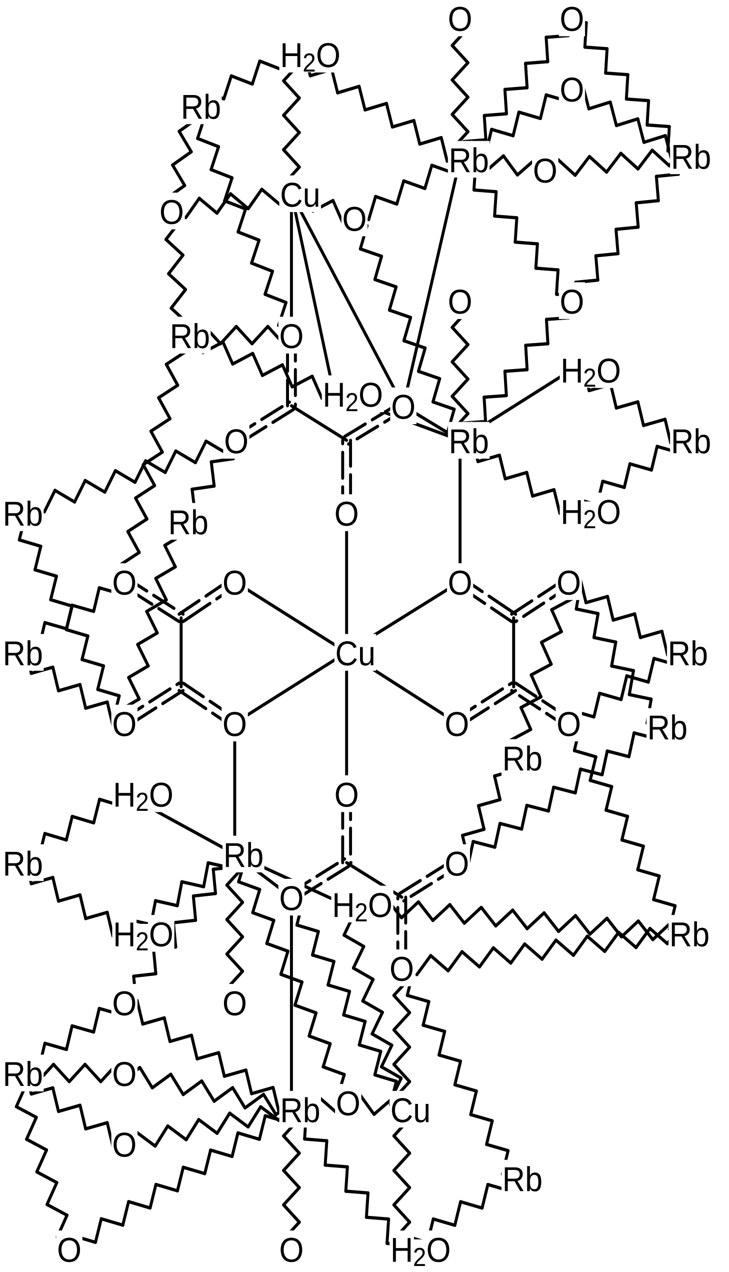

Parameters

Fragment 1

**DIST1 (D)**	1.936
**DIST2 (D)**	1.936
**DIST3 (D)**	1.949
**DIST4 (D)**	1.949
**DIST5 (D)**	2.508
**DIST6 (D)**	2.508

Fragment 2

**DIST1 (D)**	1.943
**DIST2 (D)**	1.944
**DIST3 (D)**	1.944
**DIST4 (D)**	1.943
**DIST5 (D)**	2.651
**DIST6 (D)**	2.651

Search: search2 (Wed Jul 26 09:11:25 2017): Hit 64

ERIGUI

Reference: Yue Cui, Qian Gao, Huan-Huan Wang, Lin Wang, Ya-Bo Xie (2011) *Acta Crystallogr.,Sect.E:Struct.Rep.Online* ,**67,**m782

Formula: $C_{20} H_{14} Cu_1 O_{10}$

Compound Name: Diaquabis(2-oxo-2H-chromene-3-carboxylato)copper(ii)

Space Group:	P-1	***Cell:***	***a***	6.588(1)	***b***	6.830(1)	***c***	10.460(2)
Space Group No.:	2	***(Å,°)***	α	85.98(3)	β	89.79(3)	γ	65.38(3)

R-Factor (%): 2.63 ***Temperature(K):*** 293 ***Density(g/cm^3):*** 1.860

Parameters

Fragment 1

**DIST1 (D)**	2.001
**DIST2 (D)**	1.942
**DIST3 (D)**	1.942
**DIST4 (D)**	2.001
**DIST5 (D)**	2.323
**DIST6 (D)**	2.323

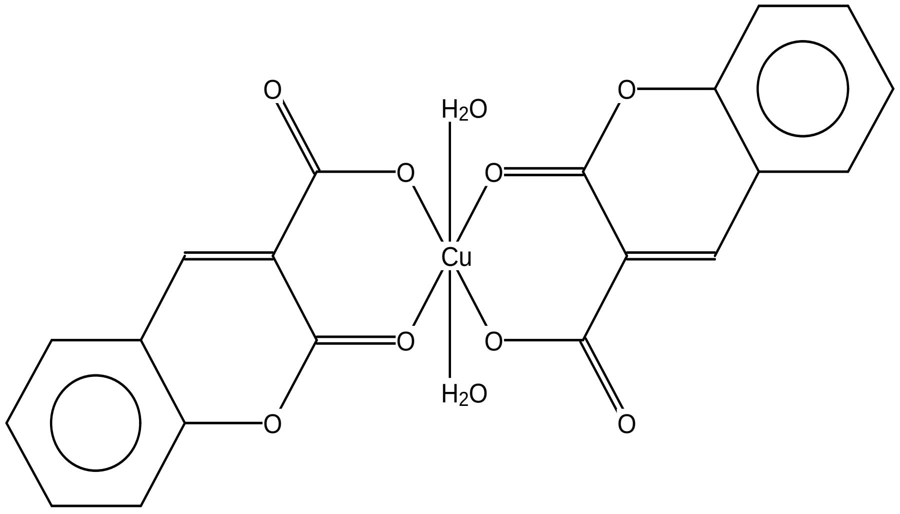

Search: search2 (Wed Jul 26 09:11:25 2017): Hit 65

FASYIJ

Reference: Hui Zhang, Yi-Min Feng (2012)
Z.Kristallogr.-New Cryst.Struct. ,**227,**237

Formula: $(C_6 H_4 Cu_1 O_8)n,2n(C_3 H_4 N_2),2n(H_2 O_1)$

Compound Name: catena(bis(μ_2-malonato)-copper(ii) imidazole dihydrate)

Space Group:	P21/c	***Cell:***	***a***	12.290(3)	***b***	7.778(1)	***c***	9.100(1)
Space Group No.:	14	***(Å,°)***	α	90.00	β	101.16(3)	γ	90.00

R-Factor (%): 2.77 ***Temperature(K):*** 293 ***Density(g/cm³):*** 1.712

Parameters

Fragment 1

**DIST1 (D)**	1.938
**DIST2 (D)**	1.950
**DIST3 (D)**	1.950
**DIST4 (D)**	1.938
**DIST5 (D)**	2.495
**DIST6 (D)**	2.495

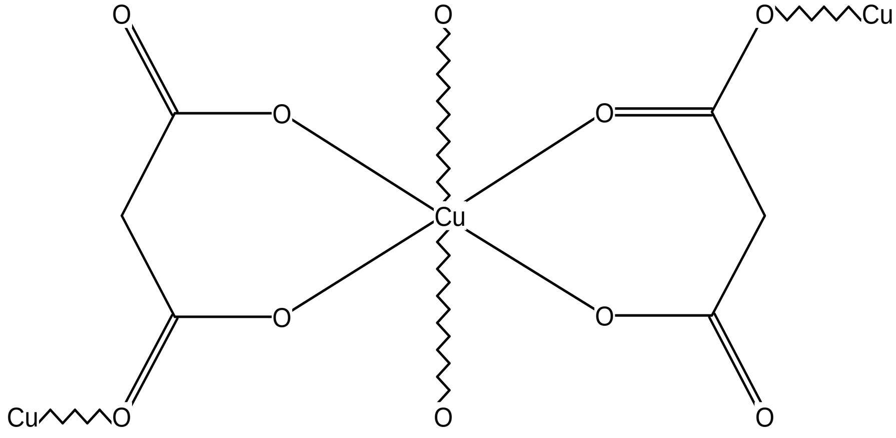

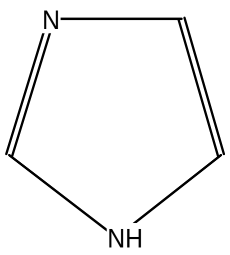

H_2O

Search: search2 (Wed Jul 26 09:11:25 2017): Hit 66

FEJXIC01

Reference: Z.S.Sahin, O.Sahin, Ozge Dagli, D.A.Kose (2016)
Polyhedron ,**117,**214

Formula: $(C_{14} H_{16} Cu_1 O_8)n$

Compound Name: catena-((μ-biphenyl-2,2'-dicarboxylato)-tetra-aqua-copper)

Space Group:	P21	***Cell:***	***a***	14.076(5)	***b***	7.612(4)	***c***	15.597(5)
Space Group No.:	4	***(Å,°)***	α	90.00	β	114.25(0)	γ	90.00

R-Factor (%): 2.89 ***Temperature(K):*** 296 ***Density(g/cm³):*** 1.638

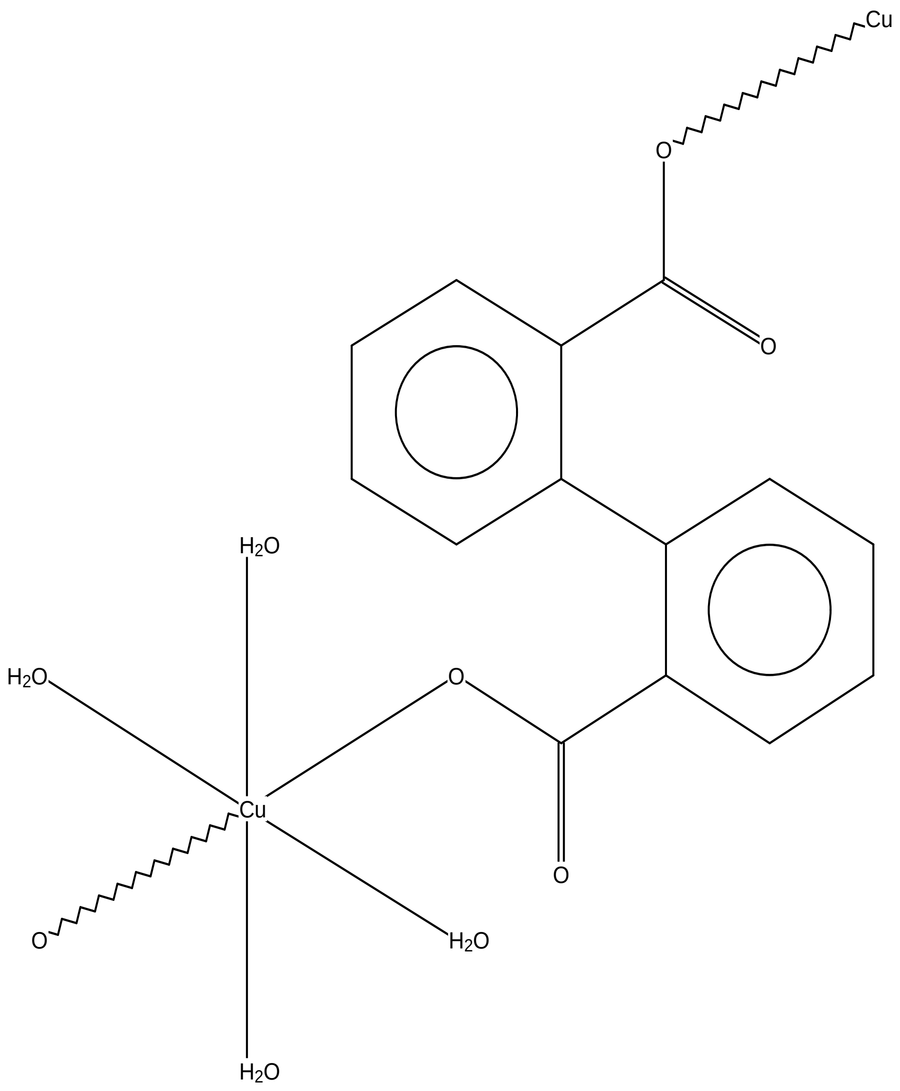

Parameters

Fragment 1

**DIST1 (D)**	2.014
**DIST2 (D)**	2.030
**DIST3 (D)**	1.941
**DIST4 (D)**	1.947
**DIST5 (D)**	2.341
**DIST6 (D)**	2.477

Fragment 2

**DIST1 (D)**	1.982
**DIST2 (D)**	1.968
**DIST3 (D)**	1.975
**DIST4 (D)**	1.964
**DIST5 (D)**	2.293
**DIST6 (D)**	2.567

Search: search2 (Wed Jul 26 09:11:25 2017): Hit 67

FILDAH

Reference:	L.Garzon-Tovar, A.Duarte-Ruiz, K.Wurst (2013) *Inorg.Chem.Commun.* ,**32,**64
Formula:	$C_{12} H_{36} Cu_1 O_6 S_6$ $^{2+}$,I_2,2(I_1 $^{1-}$)
Compound Name:	hexakis(Dimethylsulfoxide)-copper(ii) bis(iodide) di-iodine

Space Group:	R-3	***Cell:***	***a***	11.841(0)	***b***	11.841(0)	***c***	19.830(5)
Space Group No.:	148	***(Å,°)***	α	90.00	β	90.00	γ	120.00
R-Factor (%):	2.68	***Temperature(K):***	296		***Density(g/cm³):***	2.151		

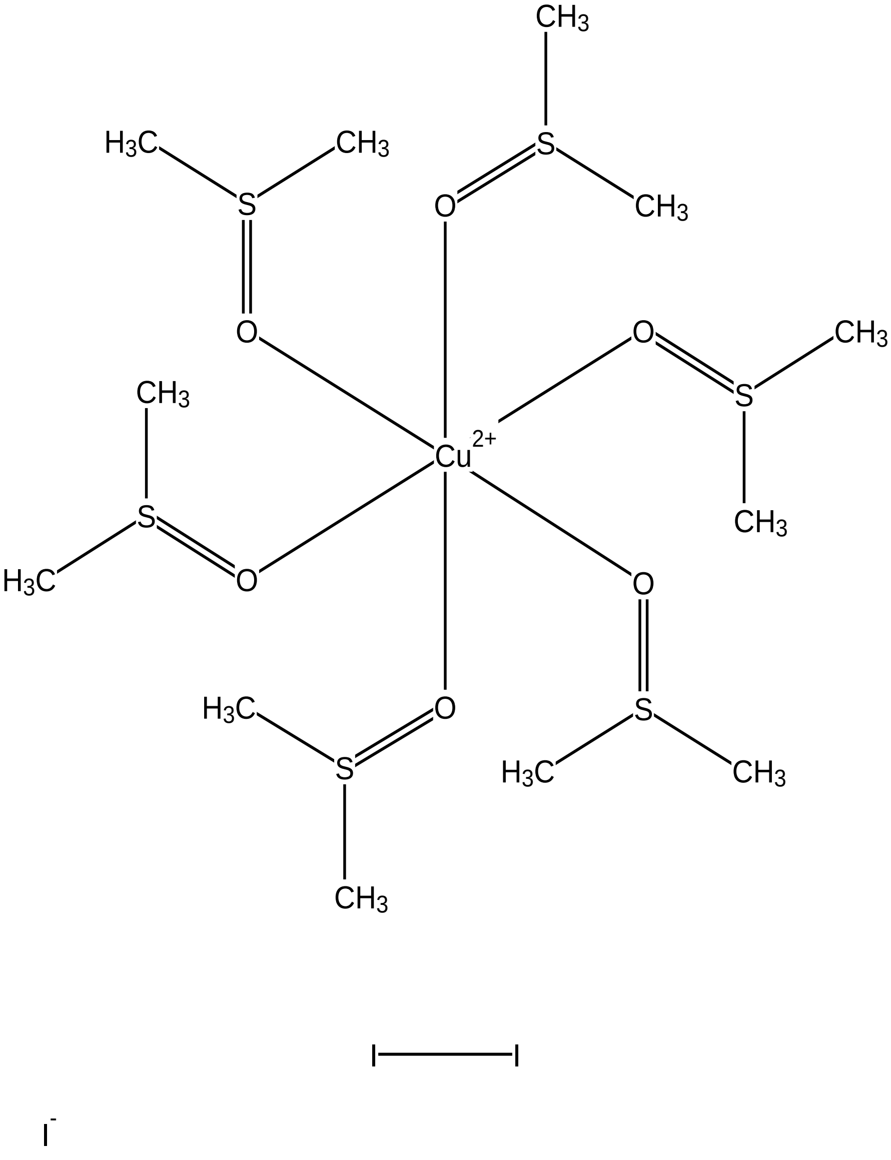

Parameters

Fragment 1

**DIST1 (D)**	2.102
**DIST2 (D)**	2.102
**DIST3 (D)**	2.102
**DIST4 (D)**	2.102
**DIST5 (D)**	2.102
**DIST6 (D)**	2.102

Search: search2 (Wed Jul 26 09:11:25 2017): Hit 68

FOFSUQ

Reference:	E.Brana, C.Mendoza, P.Vitoria, J.Gonzalez-Platas, S.Dominguez, C.Kremer (2014) *Inorg.Chim.Acta* ,**417,**192
Formula:	$(C_{18}\ H_{31}\ Cu_3\ N_5\ O_{24}\ ^{2-})n,2n(H_3\ O_1\ ^{1+})$
Compound Name:	catena-[(μ_3-3,3',3''-nitrilotripropanoato)-(μ_3-3,3',3''-ammoniotripropanoato)-tris(μ_2-nitrato)-triaqua-tri-copper dioxonium]

Space Group:	P63/m	***Cell:***	***a*** 11.040(0)	***b*** 11.040(0)	***c*** 14.698(0)
Space Group No.:	176	***(Å,°)***	α 90.00	β 90.00	γ 120.00

R-Factor (%):	2.17	***Temperature(K):***	100	***Density(g/cm³):***	1.991

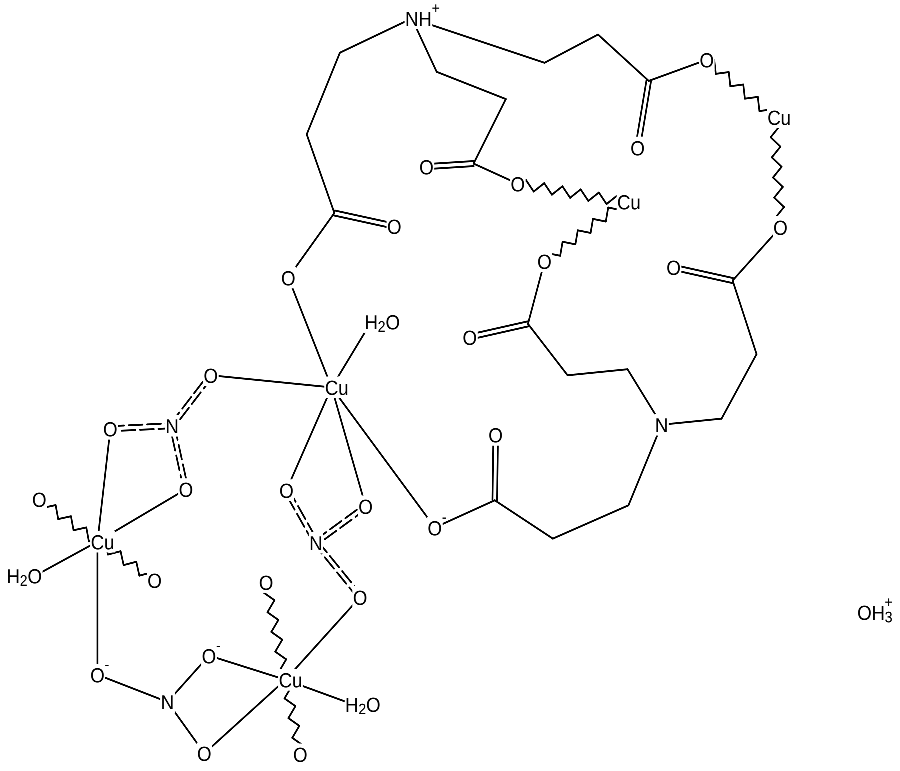

Parameters

Fragment 1

**DIST1 (D)**	1.943
**DIST2 (D)**	1.921
**DIST3 (D)**	2.000
**DIST4 (D)**	1.943
**DIST5 (D)**	2.640
**DIST6 (D)**	2.381

Search: search2 (Wed Jul 26 09:11:25 2017): Hit 69

FUDPOK

Reference: Ming-Yang He, Sheng-Chun Chen, Zhi-Hui Zhang, Kun-Lin Huang, Fang-Hua Yin, Qun Chen (2009) *Inorg.Chim.Acta* ,**362,** 2569

Formula: $(C_{12} H_{12} Cl_4 Cu_1 O_8)n,n(C_4 H_8 O_2)$

Compound Name: catena-((μ_2-1,4-dioxane)-(μ_2-2,3,4-tetrachloro-1,4-benzenedicarboxylato)-diaqua-copper(ii) 1,4-dioxane solvate)

Space Group:	P-1	***Cell:***	***a***	7.583(3)	***b***	7.936(4)	***c***	10.134(5)
Space Group No.:	2	***(Å,°)***	α	76.49(0)	β	76.10(0)	γ	70.86(0)

R-Factor (%): 2.66 ***Temperature(K):*** 296 ***Density(g/cm^3):*** 1.740

Parameters

Fragment 1

**DIST1 (D)**	1.979
**DIST2 (D)**	1.979
**DIST3 (D)**	1.942
**DIST4 (D)**	1.942
**DIST5 (D)**	2.575
**DIST6 (D)**	2.575

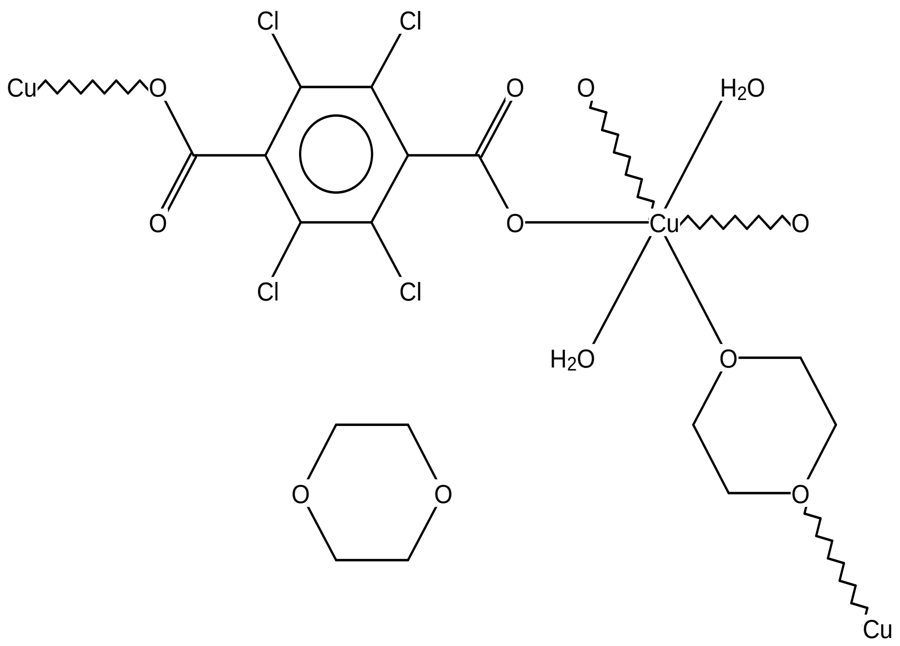

Search: search2 (Wed Jul 26 09:11:25 2017): Hit 70

GACJAX

Reference:	T.K.Prasad, M.V.Rajasekharan (2010) *Inorg.Chim.Acta* , **363,**2971
Formula:	$(C_{42}\ H_{34}\ Ce_2\ Cu_2\ N_6\ O_{32})n,16n(H_2\ O_1)$
Compound Name:	catena-(tetrakis(μ_2-pyridine-2,6-dicarboxylato)-bis(pyridine-2,6-dicarboxylato)-octaaqua-di-cerium-di-copper hexadecahydrate)

Space Group:	P-1	***Cell:***	***a*** 10.238(0)	***b*** 12.410(0)	***c*** 13.790(1)		
Space Group No.:	2	***(Å,°)***	α 84.33(0)	β 89.56(0)	γ 68.32(0)		
R-Factor (%):	2.50	***Temperature(K):***	100	***Density(g/cm³):***	1.877		

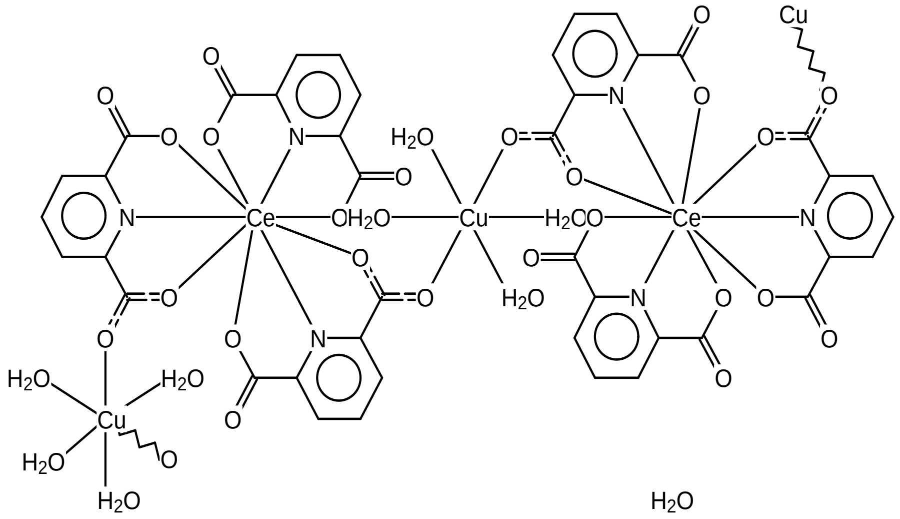

Parameters

Fragment 1

**DIST1 (D)**	1.953
**DIST2 (D)**	1.953
**DIST3 (D)**	1.987
**DIST4 (D)**	1.987
**DIST5 (D)**	2.369
**DIST6 (D)**	2.369

Fragment 2

**DIST1 (D)**	1.997
**DIST2 (D)**	1.955
**DIST3 (D)**	1.997
**DIST4 (D)**	1.955
**DIST5 (D)**	2.353
**DIST6 (D)**	2.353

Search: search2 (Wed Jul 26 09:11:25 2017): Hit 71

GACJEB

Reference:	T.K.Prasad, M.V.Rajasekharan (2010) *Inorg.Chim.Acta* , **363,**2971
Formula:	C_{42} H_{22} Ce_2 Cu_1 N_6 O_{26} $^{2-}$,H_{12} Cu_1 O_6 $^{2+}$,8(H_2 O_1)
Compound Name:	bis(μ_2-pyridine-2,6-dicarboxylato)-tetrakis(pyridine-2,6-dicarboxylato)-diaqua-copper-di-cerium hexaaqua-copper(ii) octahydrate

Space Group:	P-1	***Cell:***	***a*** 10.121(2)	***b*** 12.094(3)	***c*** 13.448(3)		
Space Group No.:	2	***(Å,°)***	α 79.61(0)	β 71.28(0)	γ 74.72(0)		
R-Factor (%):	2.33	***Temperature(K):***	298	***Density(g/cm^3):***	1.872		

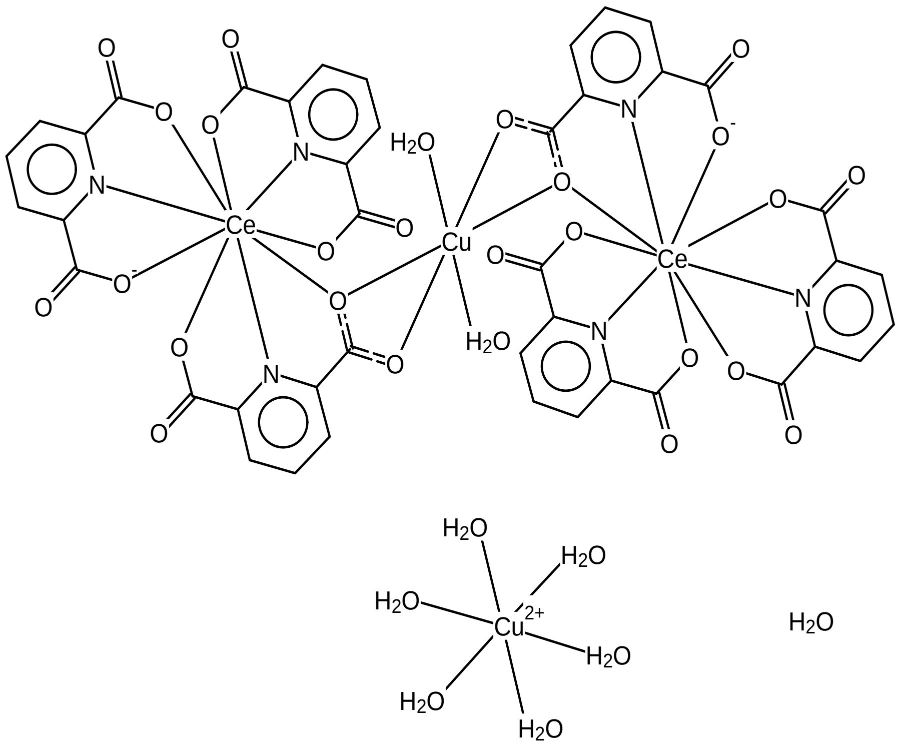

Parameters

Fragment 1

**DIST1 (D)**	1.920
**DIST2 (D)**	2.005
**DIST3 (D)**	2.005
**DIST4 (D)**	1.920
**DIST5 (D)**	2.464
**DIST6 (D)**	2.464

Fragment 2

**DIST1 (D)**	1.959
**DIST2 (D)**	1.956
**DIST3 (D)**	1.959
**DIST4 (D)**	1.956
**DIST5 (D)**	2.350
**DIST6 (D)**	2.350

Search: search2 (Wed Jul 26 09:11:25 2017): Hit 72

GEKRUL

Reference: E.V.Makotchenko, E.A.Bykova, E.Yu.Semitut, Yu.V.Shubin, P.V.Snytnikov, P.E.Plyusnin (2011) *Zh.Strukt.Khim.(Russ.)(J.Struct.Chem.)* ,**52,**952

Formula: $2(C_4 H_{16} Au_1 N_4{}^{3+}),C_4 H_4 Cu_1 O_{10}{}^{2-},2(C_4 H_2 Cu_1 O_9{}^{2-}),4(H_2 O_1)$

Compound Name: bis(Ethylenediamine-N,N')-gold(iii) bis(aqua-bis(oxalato-O,O')-copper(ii)) diaqua-bis(oxalato)-copper(ii) tetrahydrate

Space Group:	P21/c	***Cell:***	***a*** 9.176(0)	***b*** 16.975(0)	***c*** 13.447(0)
Space Group No.:	14	***(Å,°)***	α 90.00	β 104.33(0)	γ 90.00

R-Factor (%): 1.51 ***Temperature(K):*** 150 ***Density(g/cm³):*** 2.450

Parameters

Fragment 1

**DIST1 (D)**	1.988
**DIST2 (D)**	1.957
**DIST3 (D)**	1.988
**DIST4 (D)**	1.957
**DIST5 (D)**	2.356
**DIST6 (D)**	2.356

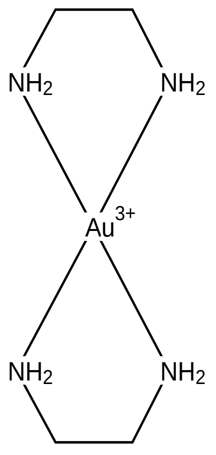

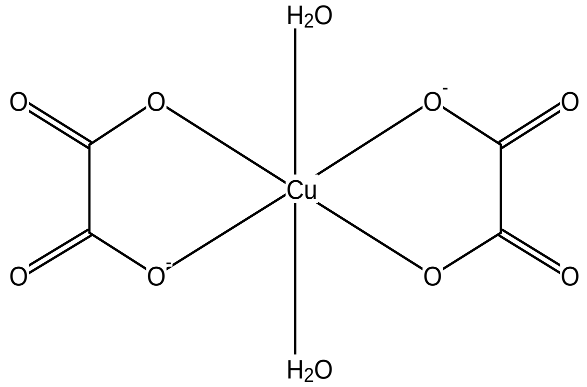

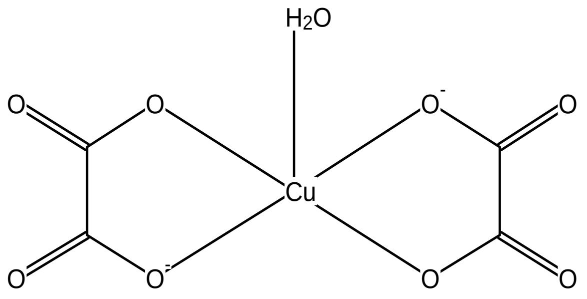

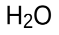

Search: search2 (Wed Jul 26 09:11:25 2017): Hit 73

GELMUH

Reference:	J.Li, Y.Liu, J.Luo, J.W.Zhao, P.T.Ma, J.Y.Niu (2011) *Koord.Khim.(Russ.)(Coord.Chem.)* ,**37,**852
Formula:	$(H_4 N_1{}^{1+})6n,2n(H_3 O_1{}^{1+}),n(C_4 Cu_1 Mo_7 O_{32}{}^{8-}),7n(H_2 O_1)$
Compound Name:	catena-(Hexaammonium bis(oxonium) bis(μ_4-oxo)-tetrakis(μ_3-oxo)-hexakis(μ_2-oxo)-bis(oxalato)-dodecaoxo-heptamolybdenum(iv)-copper(ii) heptahydrate)

Space Group:	P21/m	***Cell:***	***a*** 10.146(0)	***b*** 18.262(0)	***c*** 10.499(0)
Space Group No.:	11	***(Å,°)***	α 90.00	β 94.34(0)	γ 90.00

R-Factor (%): 2.68 ***Temperature(K):*** 295 ***Density(g/cm^3):*** 2.684

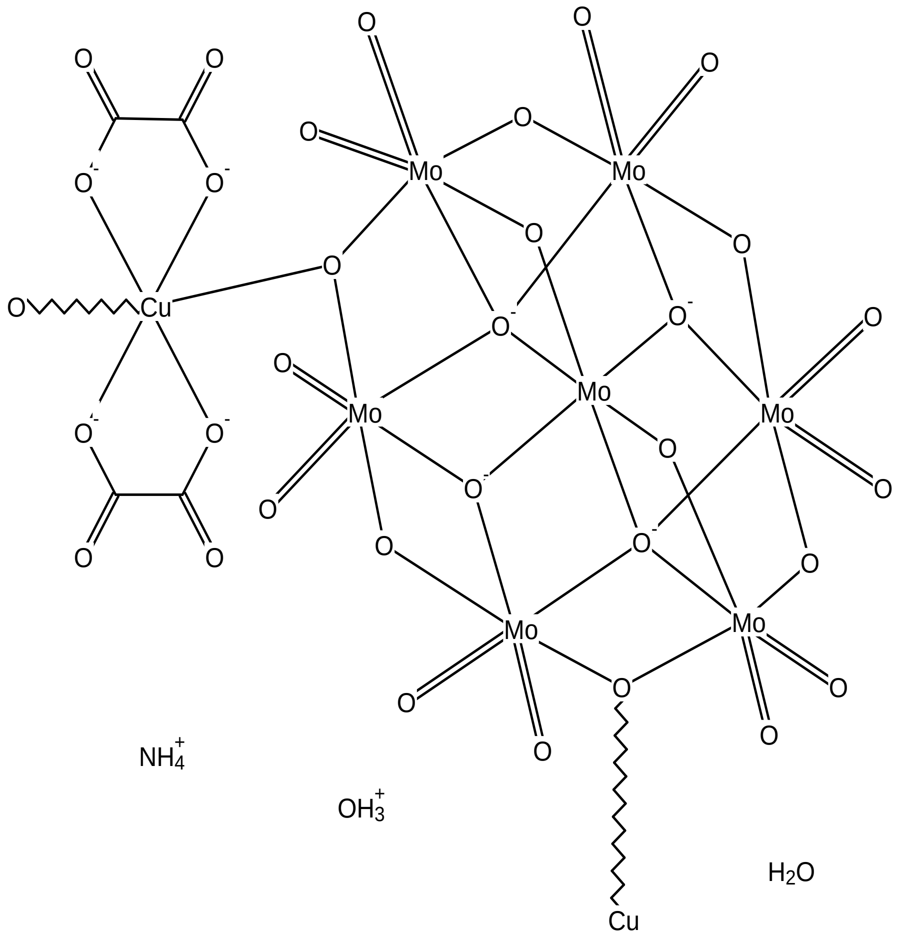

Parameters

Fragment 1

**DIST1 (D)**	1.935
**DIST2 (D)**	1.942
**DIST3 (D)**	1.935
**DIST4 (D)**	1.942
**DIST5 (D)**	2.773
**DIST6 (D)**	2.773

Search: search2 (Wed Jul 26 09:11:25 2017): Hit 74

GIKMAP

Reference:	Qing-Yan Liu, Da-Qiang Yuan, Li Xu (2007)<br>*Cryst.Growth Des.* ,**7,**1832
Formula:	C_{16} H_{34} Cu_3 O_{28} S_2
Compound Name:	bis(μ_2-5-Sulfoisophthalato)-tetradecaaqua-tri-copper(ii)

Space Group:	P-1	***Cell:***	***a***	6.614(1)	***b***	10.699(2)	***c***	12.180(3)
Space Group No.:	2	***(Å,°)***	α	71.61(0)	β	74.76(0)	γ	75.41(0)

R-Factor (%): 2.38 ***Temperature(K):*** 293 ***Density(g/cm^3):*** 1.989

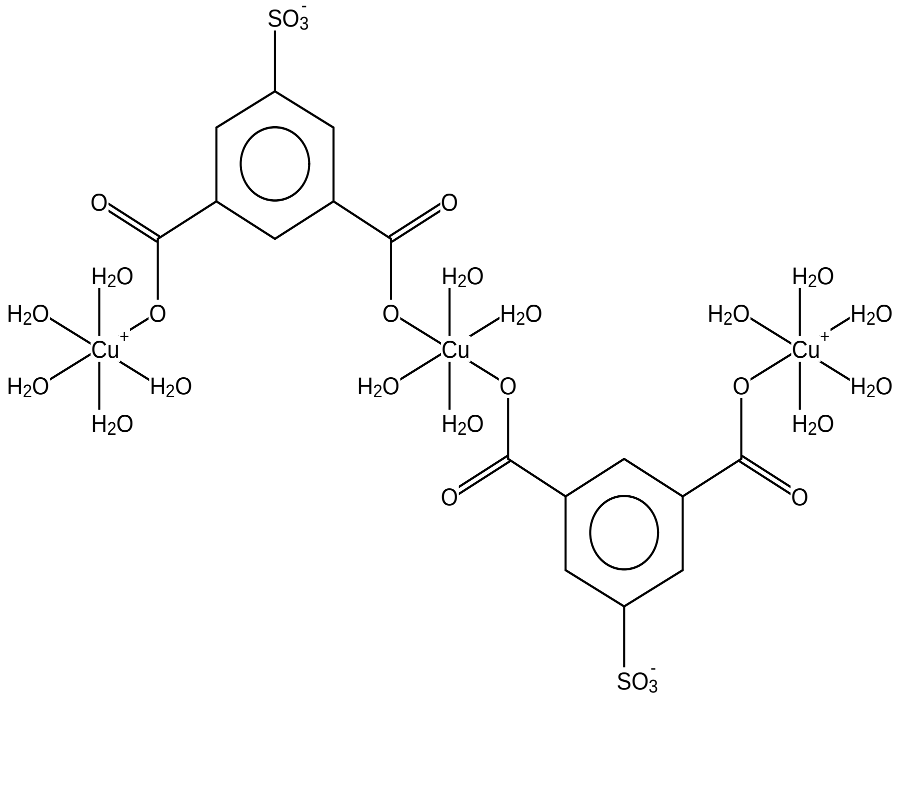

Parameters

Fragment 1	
**DIST1 (D)**	1.948
**DIST2 (D)**	1.987
**DIST3 (D)**	1.987
**DIST4 (D)**	1.948
**DIST5 (D)**	2.588
**DIST6 (D)**	2.588

Fragment 2	
**DIST1 (D)**	1.938
**DIST2 (D)**	1.999
**DIST3 (D)**	1.948
**DIST4 (D)**	1.934
**DIST5 (D)**	2.397
**DIST6 (D)**	2.473

Search: search2 (Wed Jul 26 09:11:25 2017): Hit 75

GIKMET

Reference:	Qing-Yan Liu, Da-Qiang Yuan, Li Xu (2007)<br>*Cryst.Growth Des.* ,**7,**1832
Formula:	$(C_{16} H_{22} Cu_2 O_{22} S_2\ ^{2-})n,n(C_2 H_{10} N_2\ ^{2+})$
Compound Name:	catena-(bis(μ_2-5-Sulfoisophthalato)-octaaqua-di-copper(ii) 1,2-ethylenediammonium clathrate)

Space Group:	P21/n	***Cell:***	***a*** 6.953(1)	***b*** 16.301(4)	***c*** 13.265(3)
Space Group No.:	14	***(Å,°)***	α 90.00	β 101.84(0)	γ 90.00
R-Factor (%):	2.27	***Temperature(K):*** 293	***Density(g/cm^3):*** 1.850		

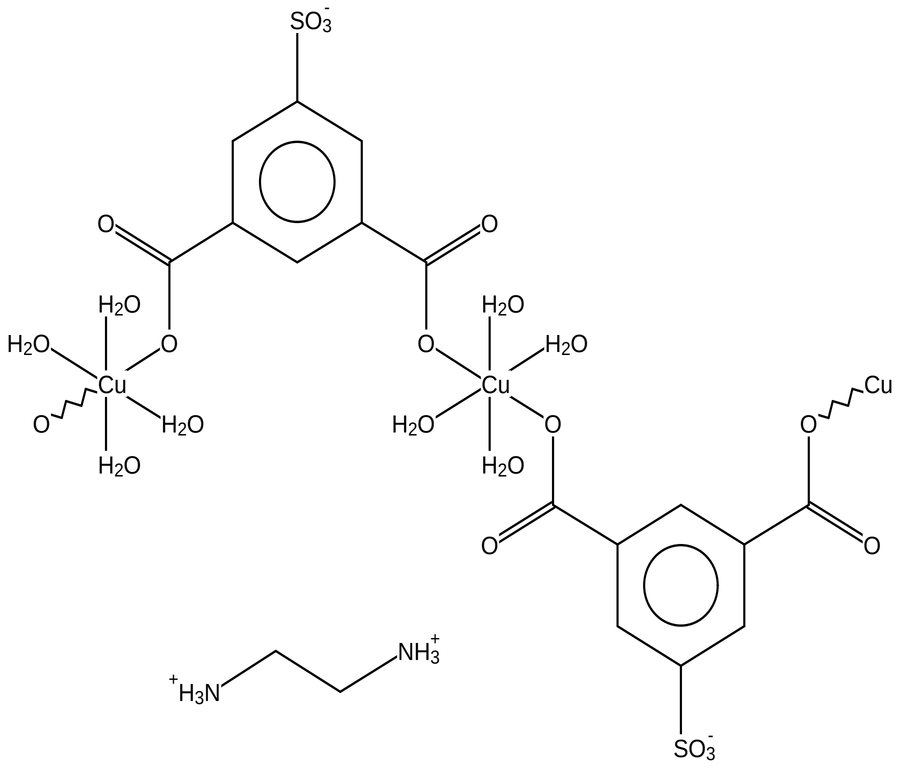

Parameters

Fragment 1

**DIST1 (D)**	1.949
**DIST2 (D)**	2.007
**DIST3 (D)**	1.975
**DIST4 (D)**	1.968
**DIST5 (D)**	2.427
**DIST6 (D)**	2.531

Search: search2 (Wed Jul 26 09:11:25 2017): Hit 76

GURZID

Reference: B.Dey, A.Das, S.R.Choudhury, A.D.Jana, Li-Ping Lu, Miao-Li Zhu, S.Mukhopadhyay (2010) *Inorg.Chim.Acta* ,**363,**981

Formula: $(C_6\ H_{12}\ Cd_1\ Cu_1\ O_{12})n$

Compound Name: catena-(bis(μ_3-malonato)-tetraaqua-cadmium(ii)-copper(ii))

Space Group:	Pbcn	***Cell:***	***a*** 6.626(1)	***b***	13.958(2)	***c***	13.052(2)
Space Group No.:	60	***(Å,°)***	α 90.00	β	90.00	γ	90.00

R-Factor (%): 2.25 ***Temperature(K):*** 298 ***Density(g/cm^3):*** 2.488

Parameters

Fragment 1

**DIST1 (D)**	1.947
**DIST2 (D)**	1.909
**DIST3 (D)**	1.947
**DIST4 (D)**	1.909
**DIST5 (D)**	2.538
**DIST6 (D)**	2.538

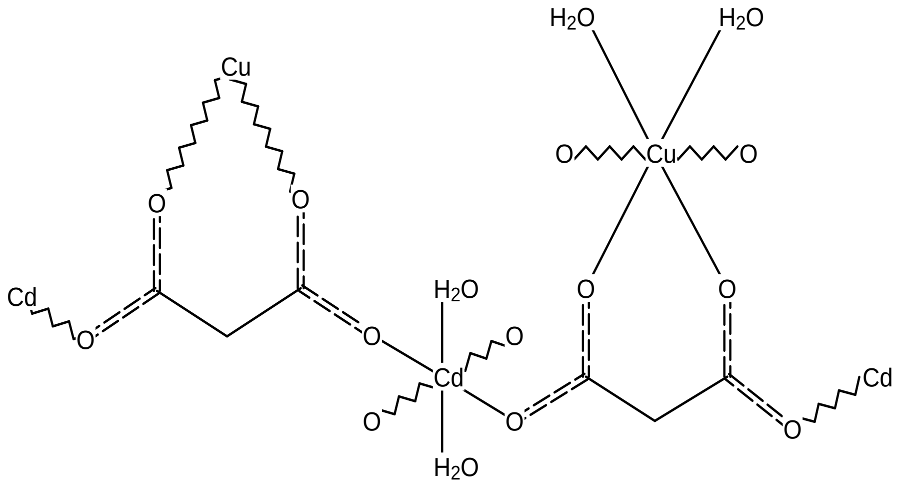

Search: search2 (Wed Jul 26 09:11:25 2017): Hit 77

GURZID01

Reference:	V.S.Dhanya, M.R.Sudarsana Kumar, M.R.P.Kurup, M.Sithambaresan, S.M.Eapen, S.M.Roy (2014) *Inorg.Chim.Acta* ,**409,** 367
Formula:	$(C_6 H_{12} Cd_1 Cu_1 O_{12})n$
Compound Name:	catena-[bis(μ_3-malonato)-tetrakis(aqua)-cadmium-copper]

Space Group:	Pbcn	***Cell:***	***a***	6.622(0)	***b***	13.977(0)	***c***	13.041(0)
Space Group No.:	60	***(Å,°)***	α	90.00	β	90.00	γ	90.00

R-Factor (%):	1.62	***Temperature(K):***	296	***Density(g/cm^3):***	2.488

Parameters

Fragment 1

**DIST1 (D)**	1.908
**DIST2 (D)**	1.945
**DIST3 (D)**	1.908
**DIST4 (D)**	1.945
**DIST5 (D)**	2.540
**DIST6 (D)**	2.540

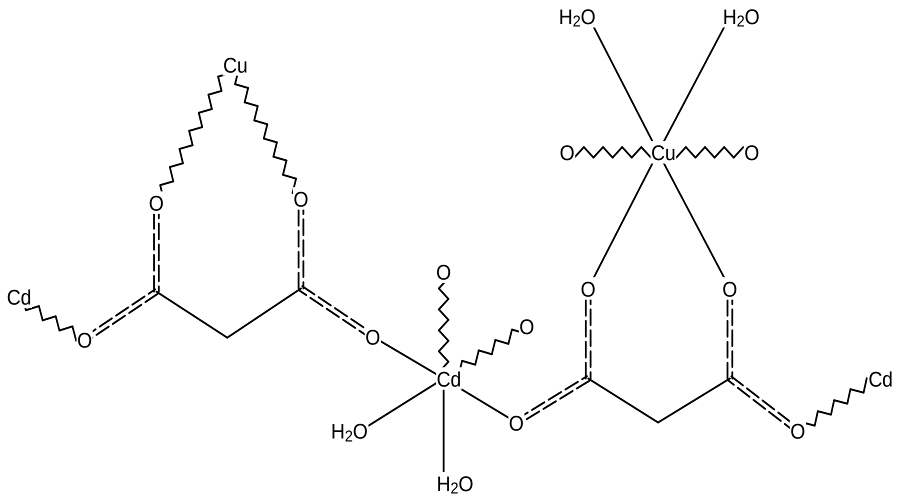

Search: search2 (Wed Jul 26 09:11:25 2017): Hit 78

HABHEY

Reference:	K.Slepokura, A.Piatkowska, T.Lis (2002) *Z.Kristallogr.* , **217**,614
Formula:	$C_4\ H_{12}\ Cu_1\ O_{12}\ P_2$
Compound Name:	Diaqua-bis(phosphonoacetato-O,O')-copper(ii)

Space Group:	P-1	***Cell:***	***a*** 6.556(2)	***b*** 8.828(3)	***c*** 10.560(3)		
Space Group No.:	2	***(Å,°)***	α 79.00(3)	β 84.09(3)	γ 89.35(3)		
R-Factor (%):	2.15	***Temperature(K):***	150	***Density(g/cm³):***	2.102		

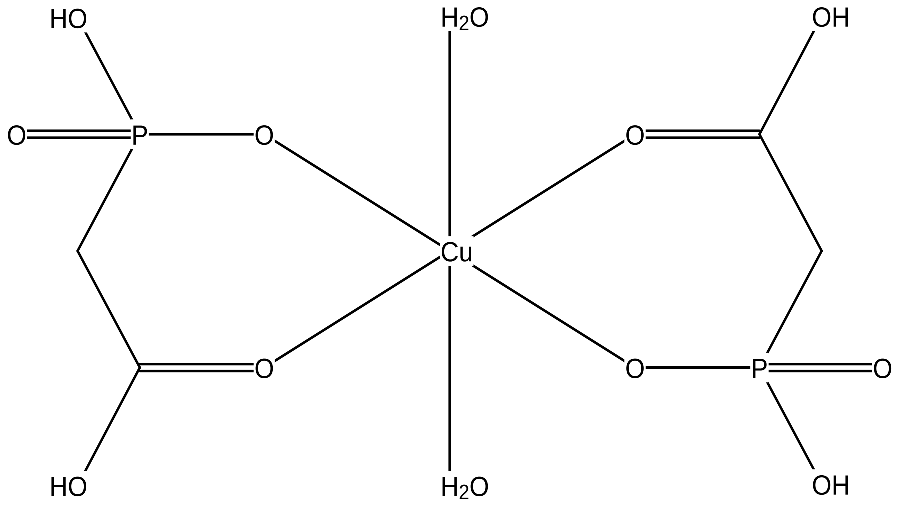

Parameters

Fragment 1

**DIST1 (D)**	2.011
**DIST2 (D)**	1.929
**DIST3 (D)**	1.929
**DIST4 (D)**	2.011
**DIST5 (D)**	2.398
**DIST6 (D)**	2.398

Fragment 2

**DIST1 (D)**	2.008
**DIST2 (D)**	1.928
**DIST3 (D)**	1.928
**DIST4 (D)**	2.008
**DIST5 (D)**	2.439
**DIST6 (D)**	2.439

Search: search2 (Wed Jul 26 09:11:25 2017): Hit 79

HACGEZ

Reference:	R.A.Allao, A.K.Jordao, J.A.L.C.Resende, A.C.Cunha, V.F.Ferreira, M.A.Novak, C.Sangregorio, L.Sorace, M.G.F.Vaz (2011) *Dalton Trans.* ,**40,**10843
Formula:	$(C_{38} H_{38} Cu_2 F_{24} N_8 O_{10})n$
Compound Name:	catena-(bis(μ_2-4-azido-2,2,6,6-tetramethylpiperidine-1-oxyl)-tetrakis(hexafluoroacetylacetonato)-di-copper)

Space Group:	P-1	***Cell:***	***a*** 8.654(0)	***b*** 12.129(0)	***c*** 12.755(3)
Space Group No.:	2	***(Å,°)***	α 77.66(0)	β 85.33(0)	γ 74.22(0)

R-Factor (%): 2.53 ***Temperature(K):*** 153 ***Density(g/cm^3):*** 1.781

Parameters

Fragment 1

**DIST1 (D)**	1.937
**DIST2 (D)**	1.941
**DIST3 (D)**	1.937
**DIST4 (D)**	1.941
**DIST5 (D)**	2.509
**DIST6 (D)**	2.509

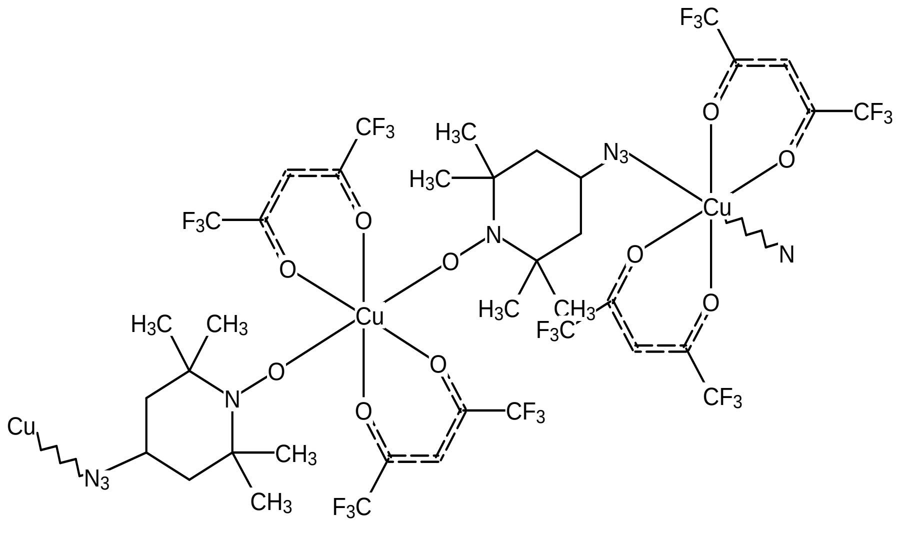

Search: search2 (Wed Jul 26 09:11:25 2017): Hit 80

HAJRAM

Reference:	F.S.Delgado, J.Sanchiz, C.Ruiz-Perez, F.Lloret, M.Julve<br>(2004) *CrystEngComm* ,**6,**443
Formula:	$(C_6\ H_6\ Cu_1\ O_8)n$
Compound Name:	catena-(bis(μ_2-malonato)-copper(ii))

Space Group:	Pbca	***Cell:***	***a***	6.742(0)	***b***	9.382(0)	***c***	12.452(1)
Space Group No.:	61	***(Å,°)***	α	90.00	β	90.00	γ	90.00
R-Factor (%):	2.78	***Temperature(K):***	293		***Density(g/cm³):***	2.274		

Parameters

Fragment 1

**DIST1 (D)**	1.944
**DIST2 (D)**	1.932
**DIST3 (D)**	1.932
**DIST4 (D)**	1.944
**DIST5 (D)**	2.543
**DIST6 (D)**	2.543

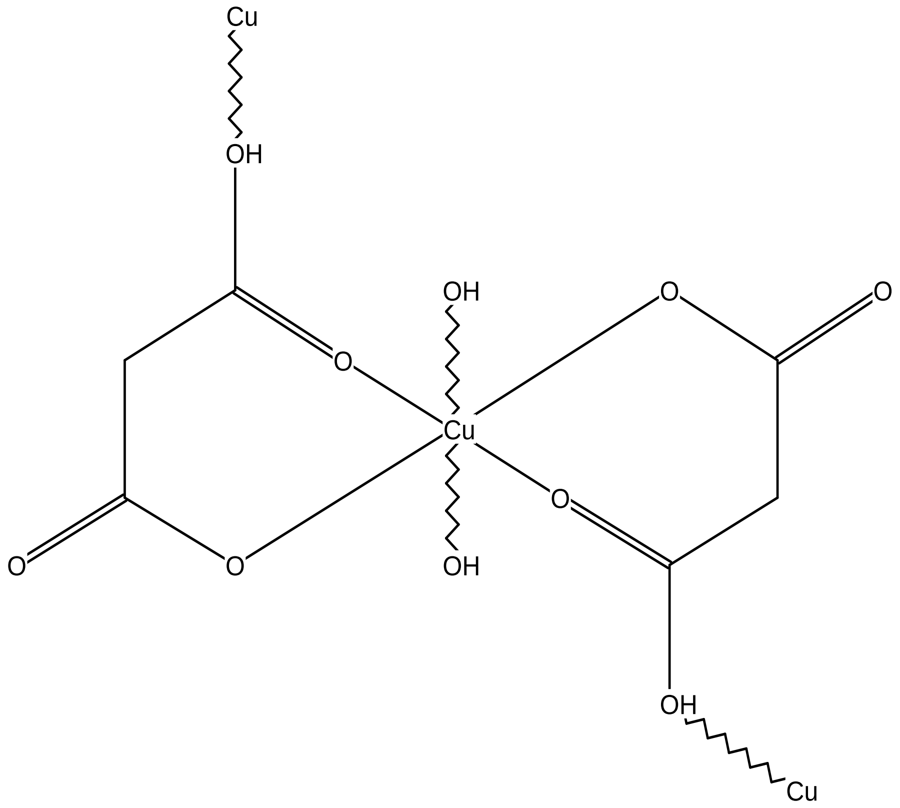

Search: search2 (Wed Jul 26 09:11:25 2017): Hit 81

HESKIZ

Reference:	S.Kawata, S.Kitagawa, M.Kondo, I.Furuchi, M.Munakata (1994) *Angew.Chem.,Int.Ed.* ,**33,**1759
Formula:	$(C_8\ H_8\ Cl_2\ Cu_1\ O_6)n$
Compound Name:	catena-((μ_2-3,6-Dichloro-2,5-dihydroxy-p-benzoquinonato)-bis(methanol-O)-copper(ii))

Space Group:	P-1	***Cell:***	***a***	7.672(0)	***b***	7.780(2)	***c***	5.108(0)
Space Group No.:	2	***(Å,°)***	α	99.89(2)	β	98.41(1)	γ	100.25(2)

R-Factor (%):	2.90	***Temperature(K):***	295	***Density(g/cm^3):***	1.912

Parameters

Fragment 1

**DIST1 (D)**	1.963
**DIST2 (D)**	1.963
**DIST3 (D)**	2.005
**DIST4 (D)**	2.005
**DIST5 (D)**	2.279
**DIST6 (D)**	2.279

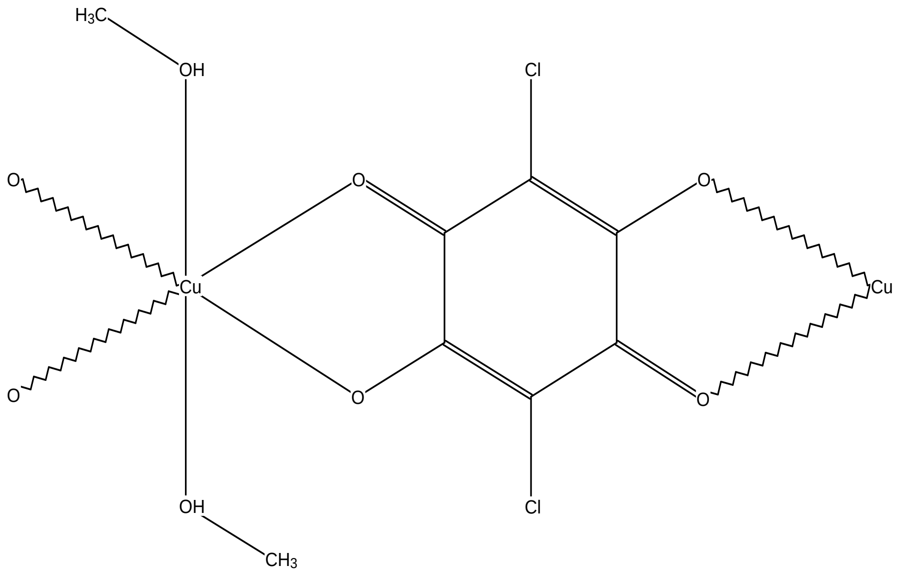

Search: search2 (Wed Jul 26 09:11:25 2017): Hit 82

HINWEH

Reference:	S.Balboa, A.Castineiras, P.S.Herle, J.Strahle (2007) *Z.Anorg.Allg.Chem.* ,**633,**2420						
Formula:	$(C_6 H_{10} Cu_1 O_6)n$						
Compound Name:	catena-(bis(μ_2-Lactato-O,O',O'')-copper(ii))						
Space Group:	P21/c	***Cell:***	***a*** 5.185(1)	***b*** 8.362(1)	***c*** 8.841(4)		
Space Group No.:	14	***(Å,°)***	α 90.00	β 110.86(3)	γ 90.00		
R-Factor (%):	2.63	***Temperature(K):***	208	***Density(g/cm^3):***	2.241		

Parameters

Fragment 1

**DIST1 (D)**	1.947
**DIST2 (D)**	1.926
**DIST3 (D)**	1.926
**DIST4 (D)**	1.947
**DIST5 (D)**	2.499
**DIST6 (D)**	2.499

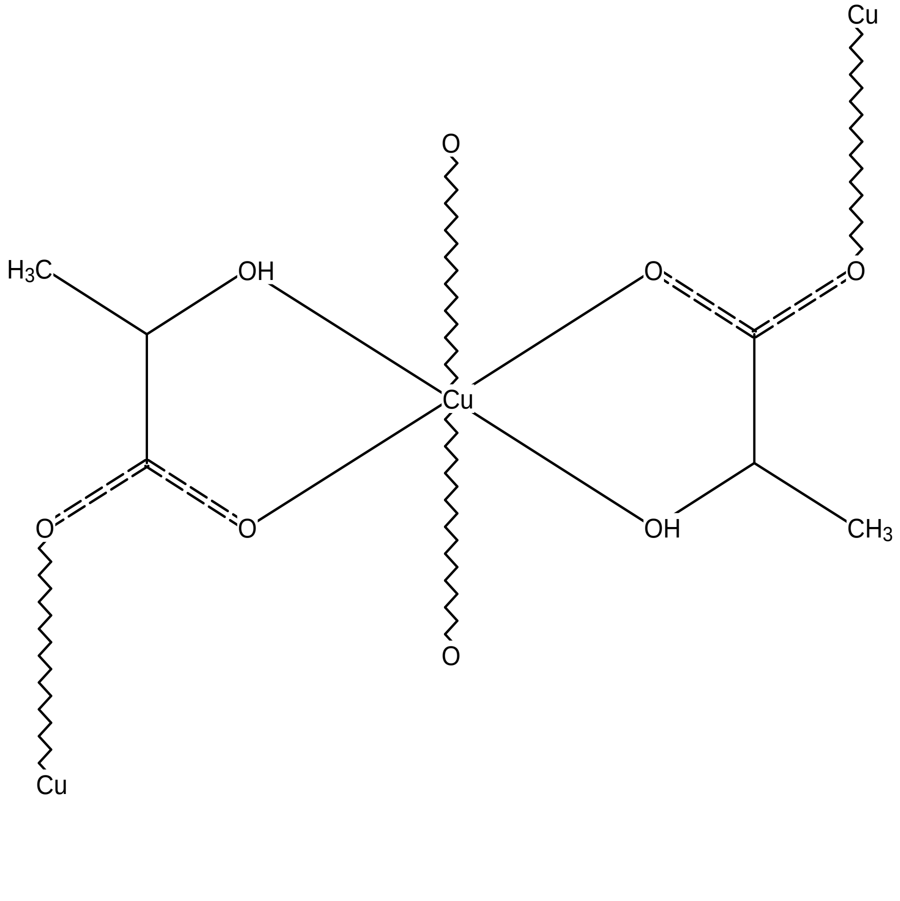

Search: search2 (Wed Jul 26 09:11:25 2017): Hit 83

HOWFEE

Reference: V.S.Sergienko, G.G.Aleksandrov, E.G.Afonin (1999)
Koord.Khim.(Russ.)(Coord.Chem.) ,**25,**451

Formula: $C_8 H_{28} Cu_2 O_{30} P_8{}^{4-}$,4($K_1{}^{1+}$),4($H_2 O_1$)

Compound Name: Tetra-potassium bis(aqua-(μ_2-(1-hydroxyethane-1,1-diphosphonato-O, O'))-(1-hydroxyethane-1,1-diphosphonato-O,O)-copper(ii)) tetrahydrate

Space Group:	P21/c	***Cell:***	***a***	12.530(1)	***b***	12.143(1)	***c***	12.787(1)
Space Group No.:	14	***(Å,°)***	α	90.00	β	108.95(1)	γ	90.00

R-Factor (%): 2.45 ***Temperature(K):*** 295 ***Density(g/cm³):*** 2.180

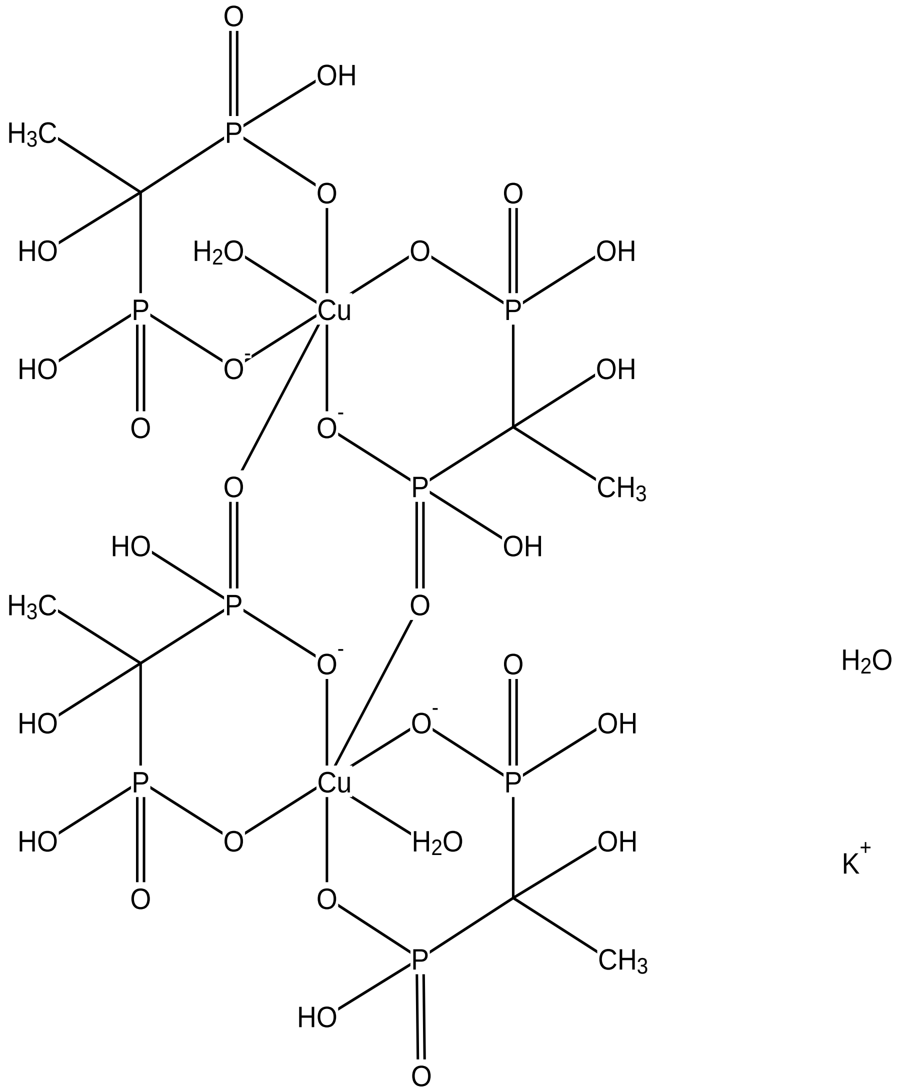

Parameters

Fragment 1

**DIST1 (D)**	1.984
**DIST2 (D)**	1.960
**DIST3 (D)**	1.985
**DIST4 (D)**	2.033
**DIST5 (D)**	2.359
**DIST6 (D)**	2.327

Search: search2 (Wed Jul 26 09:11:25 2017): Hit 84

HULMAC

Reference: Y.Rodriguez-Martin, J.Sanchiz, C.Ruiz-Perez, F.Lloret, M.Julve (2002) *CrystEngComm* ,**4,**631

Formula: $H_{12}\,Co_1\,O_6\,^{2+}, C_6\,H_8\,Cu_1\,O_{10}\,^{2-}$

Compound Name: Hexa-aqua-cobalt(ii) diaqua-bis(malonato-O,O')-copper(ii)

Space Group:	P-1	***Cell:***	***a***	5.266(1)	***b***	7.495(1)	***c***	10.332(2)
Space Group No.:	2	***(Å,°)***	α	107.14(0)	β	98.99(0)	γ	96.17(0)

R-Factor (%): 2.88 ***Temperature(K):*** 293 ***Density(g/cm³):*** 2.058

Parameters

Fragment 1

**DIST1 (D)**	1.946
**DIST2 (D)**	1.947
**DIST3 (D)**	1.946
**DIST4 (D)**	1.947
**DIST5 (D)**	2.459
**DIST6 (D)**	2.459

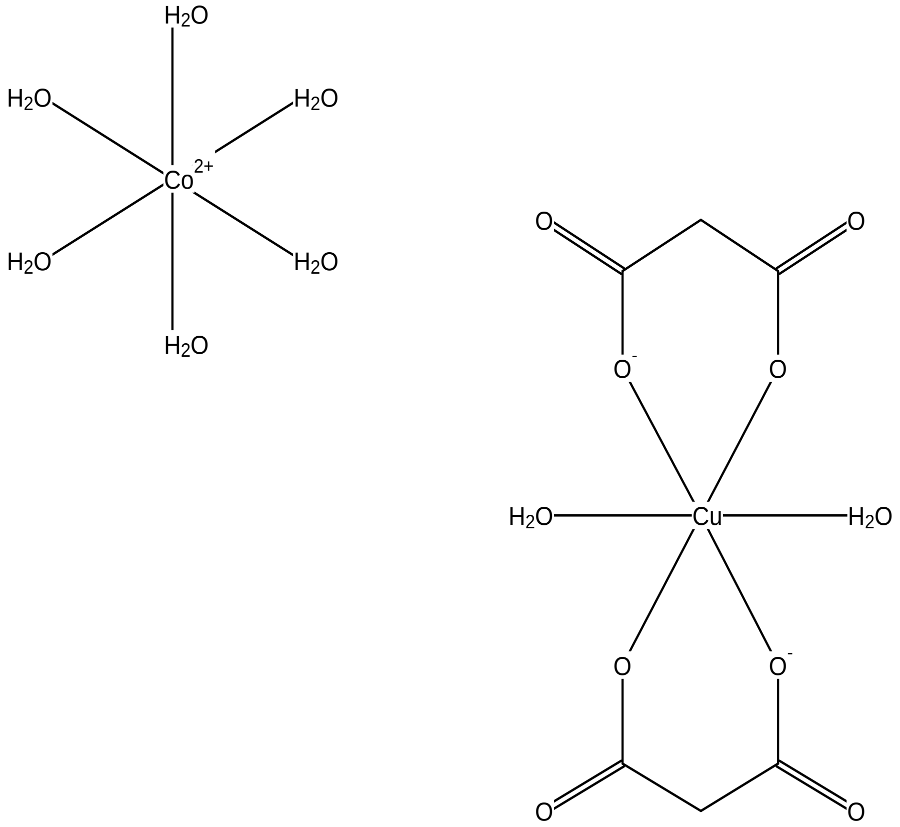

Search: search2 (Wed Jul 26 09:11:25 2017): Hit 85

HULMEG

Reference: Y.Rodriguez-Martin, J.Sanchiz, C.Ruiz-Perez, F.Lloret, M.Julve (2002) *CrystEngComm* ,**4,**631

Formula: $H_{12}\ Ni_1\ O_6\ ^{2+}, C_6\ H_8\ Cu_1\ O_{10}\ ^{2-}$

Compound Name: Hexa-aqua-nickel(ii) diaqua-bis(malonato-O,O')-copper(ii)

Space Group:	P-1	***Cell:***	***a*** 5.242(1)	***b*** 7.480(3)	***c*** 10.278(4)		
Space Group No.:	2	***(Å,°)***	α 107.15(3)	β 98.77(3)	γ 96.34(3)		

R-Factor (%): 2.73 ***Temperature(K):*** 293 ***Density(g/cm^3):*** 2.081

Parameters

Fragment 1

**DIST1 (D)**	1.946
**DIST2 (D)**	1.947
**DIST3 (D)**	1.946
**DIST4 (D)**	1.947
**DIST5 (D)**	2.445
**DIST6 (D)**	2.445

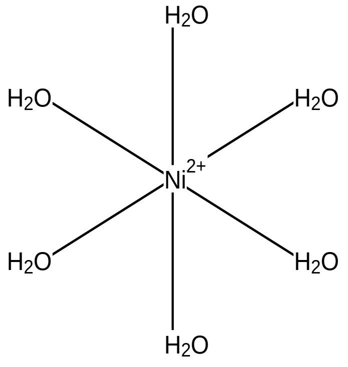

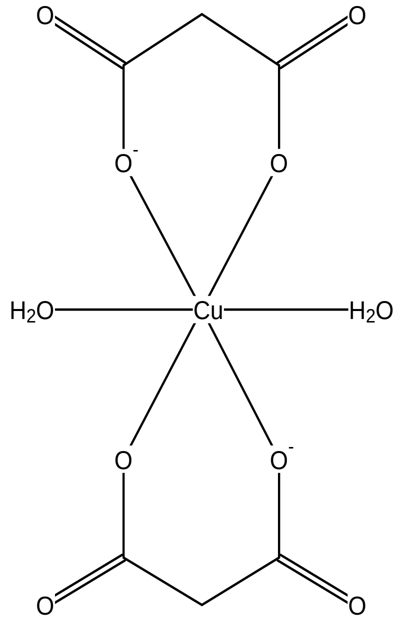

Search: search2 (Wed Jul 26 09:11:25 2017): Hit 86

HUXWAY

Reference: B.Barja, R.Baggio, M.T.Garland, P.F.Aramendia, O.Pena, M.Perec (2003) *Inorg.Chim.Acta* ,**346,**187

Formula: $(C_{24} H_{36} Cu_3 O_{36} Tb_2)n,12n(H_2 O_1)$

Compound Name: catena-[hexakis(μ_3-2,2'-Oxydiacetato)-hexa-aqua-tri-copper(ii)-di-terbium(iii) dodecahydrate]

Space Group:	P6/mcc	***Cell:***	***a***	14.693(2)	***b***	14.693(2)	***c***	15.125(2)
Space Group No.:	192	***(Å,°)***	α	90.00	β	90.00	γ	120.00

R-Factor (%): 2.74 ***Temperature(K):*** 293 ***Density(g/cm³):*** 1.909

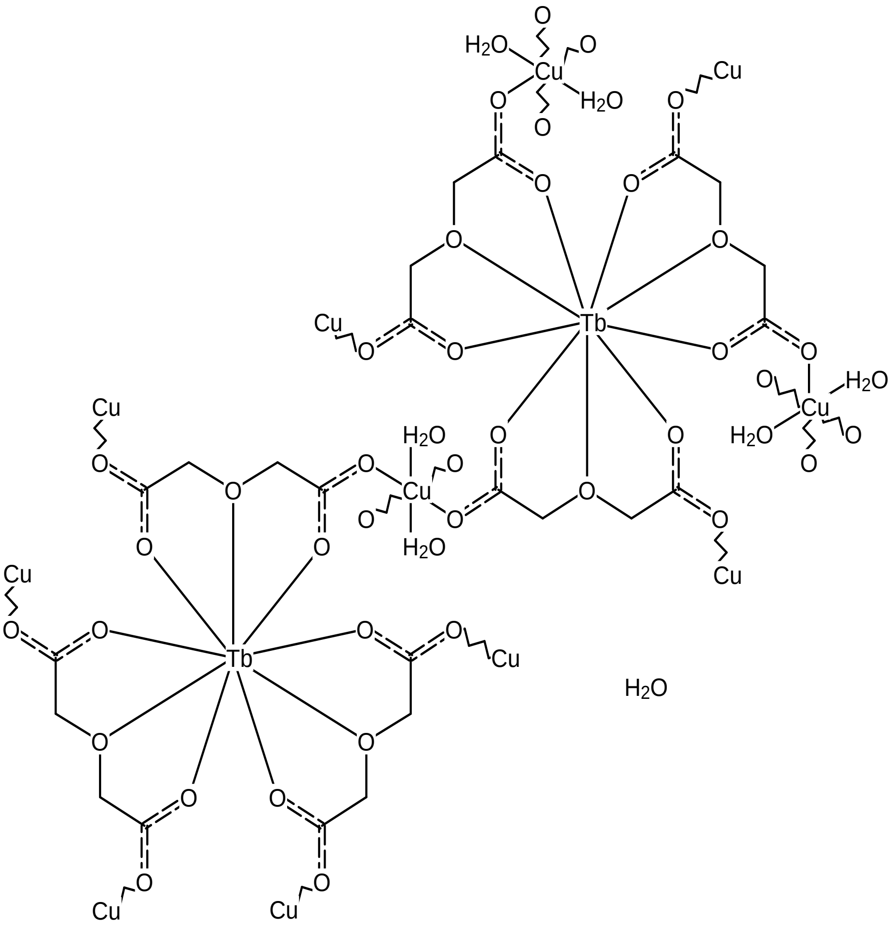

Parameters

Fragment 1

**DIST1 (D)**	1.951
**DIST2 (D)**	1.951
**DIST3 (D)**	1.951
**DIST4 (D)**	1.951
**DIST5 (D)**	2.500
**DIST6 (D)**	2.500

Search: search2 (Wed Jul 26 09:11:25 2017): Hit 87

ICAMOQ

Reference: Wei Chen, Jinfeng Chu, I.Mutikainen, J.Reedijk, U.Turpeinen, Yu-Fei Song (2011) *CrystEngComm* ,**13,**7299

Formula: $C_{26} H_{32} Cu_2 N_{12} O_2{}^{4+}$,2($H_2 O_1$),$C_4 Cl_2 Cu_1 O_{16}{}^{4-}$

Compound Name: bis(ethanedioato)-bis(perchlorato)-copper bis(μ_2-1,1-bis(1H-imidazol-2-yl)-N-((pyridin-2-yl)methyl)methanamine)-diaqua-di-copper dihydrate

Space Group:	P21/n	***Cell:***	***a***	10.145(1)	***b***	16.441(2)	***c***	13.180(2)
Space Group No.:	14	***(Å,°)***	α	90.00	β	109.54(0)	γ	90.00

R-Factor (%): 2.89 ***Temperature(K):*** 93 ***Density(g/cm^3):*** 1.838

Parameters

Fragment 1

**DIST1 (D)**	1.968
**DIST2 (D)**	1.926
**DIST3 (D)**	1.968
**DIST4 (D)**	1.926
**DIST5 (D)**	2.463
**DIST6 (D)**	2.463

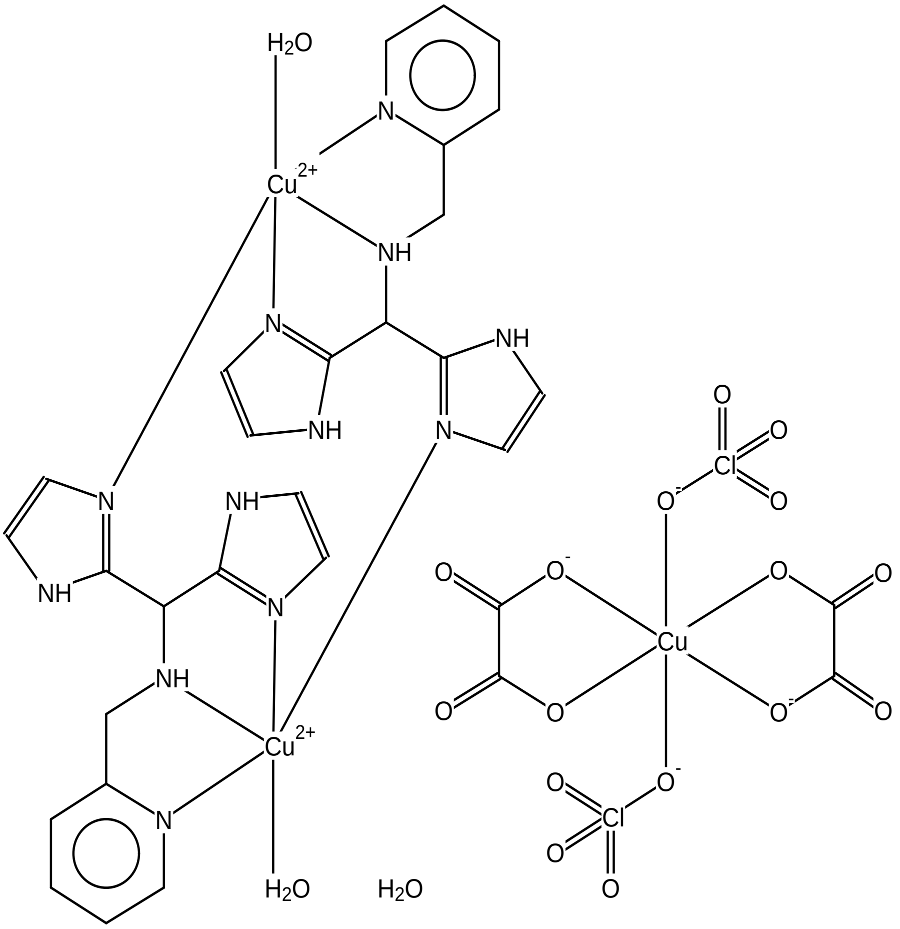

Search: search2 (Wed Jul 26 09:11:25 2017): Hit 88

IFAXOC

Reference:	O.Moers, D.Henschel, A.Blaschette, P.G.Jones (2002) *Z.Anorg.Allg.Chem.* ,**628,**505						
Formula:	C_{12} H_{16} Cu_1 N_2 O_{12} S_4,2(H_2 O_1)						
Compound Name:	bis(1,1,3,3-Tetraoxo-1,3,2-benzodithiazole)-tetraaqua-copper(ii) dihydrate						
Synonym:	bis(benzol-1,2-bis(sulfonyl)amide)-tetraaqua-copper(ii) dihydrate						
Space Group:	P-1	***Cell:***	***a*** 7.054(0)	***b*** 7.518(0)	***c*** 11.193(1)		
Space Group No.:	2	***(Å,°)***	α 79.24(0)	β 81.10(0)	γ 78.54(0)		
R-Factor (%):	2.25	***Temperature(K):***	173	***Density(g/cm³):***	1.780		

Parameters

Fragment 1

**DIST1 (D)**	1.960
**DIST2 (D)**	1.941
**DIST3 (D)**	1.960
**DIST4 (D)**	1.941
**DIST5 (D)**	2.404
**DIST6 (D)**	2.404

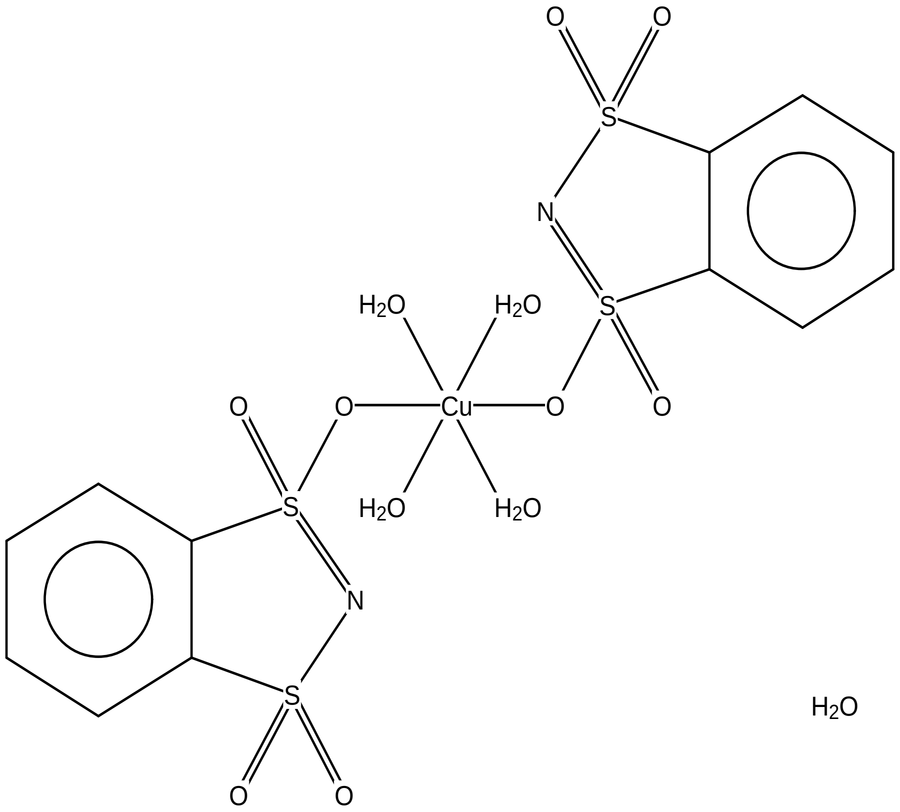

Search: search2 (Wed Jul 26 09:11:25 2017): Hit 89

IKIPEX02

Reference:	M.A.Ershov, V.G.Skvortsov, Y.Yu.Pilchikova, O.V.Koltsova, K.Yu.Suponitsky (2006) *Acta Crystallogr.,Sect.E:Struct.Rep.Online* ,**62,**m3073
Formula:	C_6 H_{12} Cu_1 N_6 O_{10}
Compound Name:	bis(malonamide-κ^{2}O,O')bis(nitrato-κO)copper(ii)

Space Group:	P-1	***Cell:***	***a*** 6.852(0)	***b*** 7.070(0)	***c*** 7.461(0)
Space Group No.:	2	***(Å,°)***	α 89.66(0)	β 69.49(0)	γ 73.48(0)
R-Factor (%):	2.41	***Temperature(K):***	120	***Density(g/cm^3):***	2.015

Parameters

Fragment 1

**DIST1 (D)**	1.973
**DIST2 (D)**	1.934
**DIST3 (D)**	1.973
**DIST4 (D)**	1.934
**DIST5 (D)**	2.396
**DIST6 (D)**	2.396

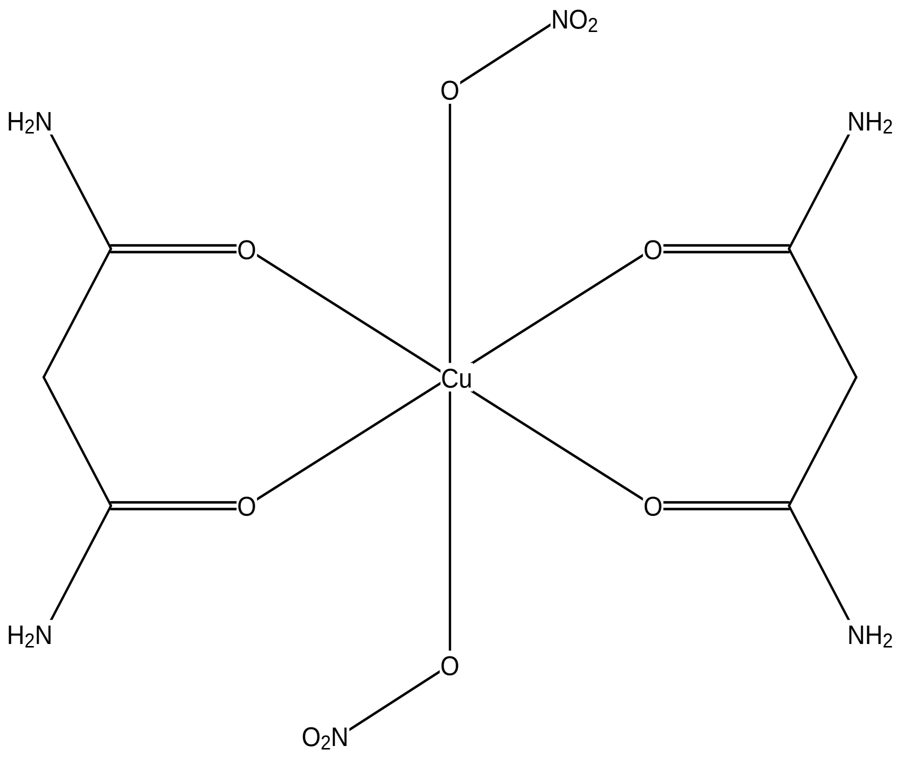

Search: search2 (Wed Jul 26 09:11:25 2017): Hit 90

ININID

Reference: Chong-Bo Liu, Hui-Liang Wen, Yun-Nan Gong, Xiao-Ming Liu, Sheng-Shui Tan (2011) *Z.Anorg.Allg.Chem.* ,**637,**122

Formula: $C_{22} H_{26} Cu_1 O_{12}$

Compound Name: Diaqua-bis(2-(4-(2-carboxyethoxy)phenoxy)acetato)-copper(ii)

Space Group:	P-1	***Cell:***	***a***	5.337(1)	***b***	6.710(1)	***c***	16.386(3)
Space Group No.:	2	***(Å,°)***	α	87.15(0)	β	82.17(0)	γ	73.13(0)

R-Factor (%): 2.53 ***Temperature(K):*** 291 ***Density(g/cm3):*** 1.630

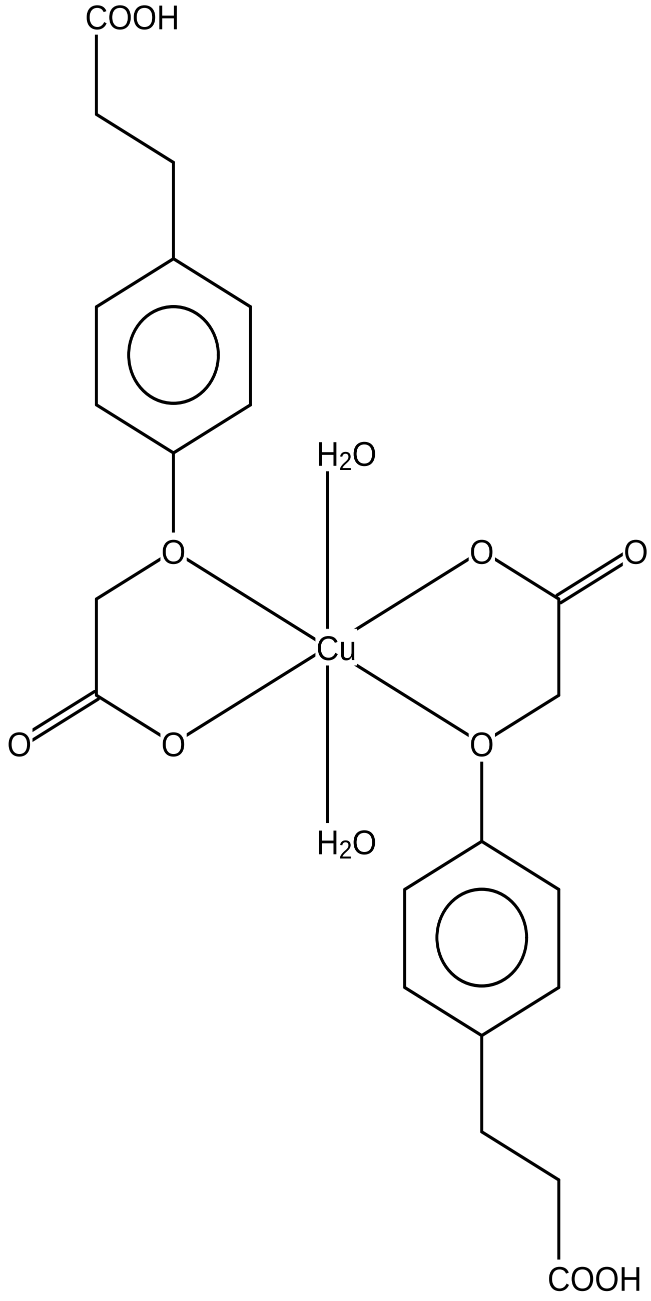

Parameters

Fragment 1

**DIST1 (D)**	1.947
**DIST2 (D)**	1.963
**DIST3 (D)**	1.963
**DIST4 (D)**	1.947
**DIST5 (D)**	2.420
**DIST6 (D)**	2.420

Search: search2 (Wed Jul 26 09:11:25 2017): Hit 91

IPOWOA

Reference: Yue-Qing Zheng, Xia-Xia Guo, Xiao-Yan Han, Jia-Hui Xu
(2011) *Inorg.Chim.Acta* ,**370,**36

Formula: $(C_{24} H_{34} Cu_5 O_{34})n,4n(H_2 O_1)$

Compound Name: catena(-bis(μ_6-6-Carboxyl-benzene-1,2,3,4,5-pentacarboxylato)-deca-aqua-penta-copper tetrahydrate)

Space Group:	C2/c	***Cell:***	***a*** 17.101(3)	***b***	8.397(1)	***c***	27.654(6)
Space Group No.:	15	***(Å,°)***	α 90.00	β	104.63(3)	γ	90.00

R-Factor (%): 2.65 ***Temperature(K):*** 293 ***Density(g/cm³):*** 2.172

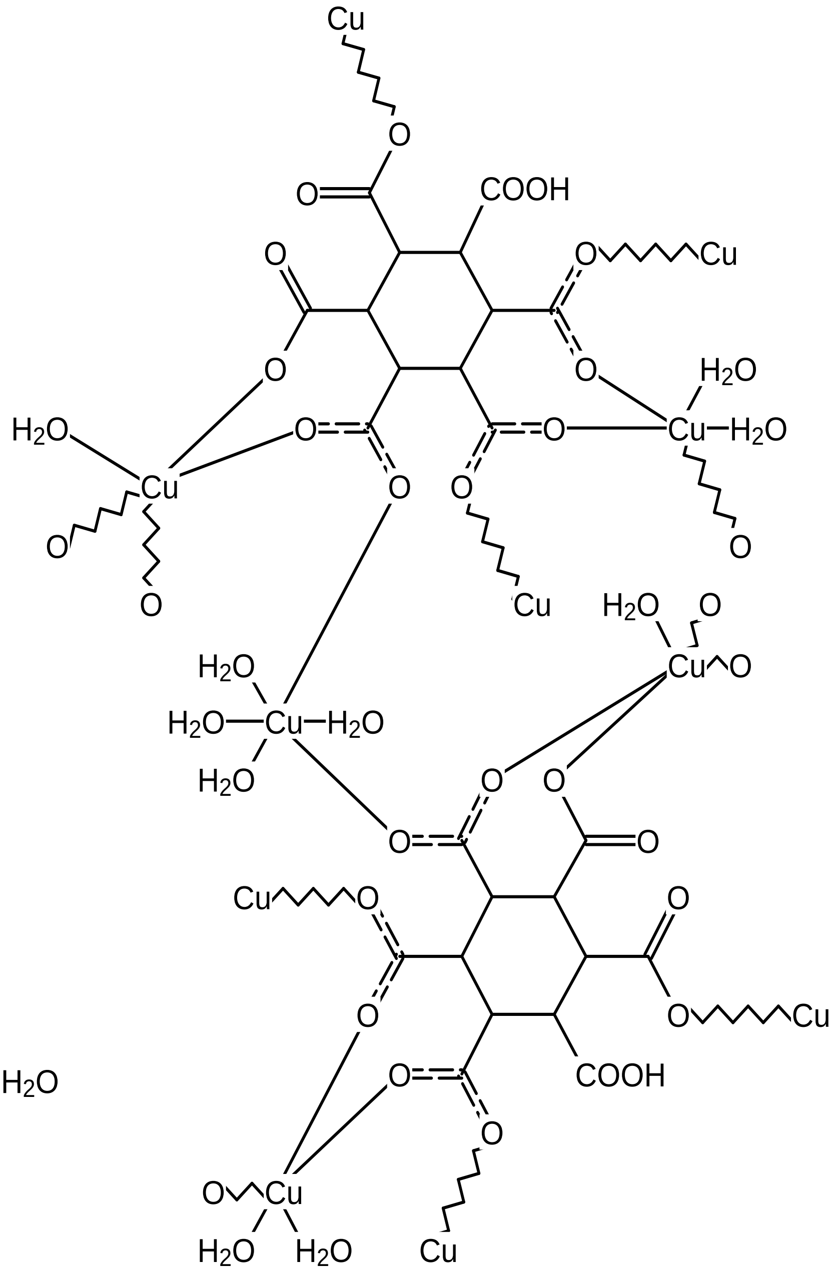

Parameters

Fragment 1

**DIST1 (D)**	1.953
**DIST2 (D)**	1.950
**DIST3 (D)**	1.950
**DIST4 (D)**	1.935
**DIST5 (D)**	2.408
**DIST6 (D)**	2.408

Search: search2 (Wed Jul 26 09:11:25 2017): Hit 92

JEGNOZ

Reference: Zhu-Yan Zhang, Shan Gao, Li-Hua Huo, Hui Zhao, Jing-Gui Zhao (2006) *Wuji Huaxue Xuebao(Chin.)(Chin.J.Inorg.Chem.)* ,**22,**905

Formula: $(C_{14} H_{12} Cu_1 N_2 O_6)n$

Compound Name: catena-(bis(μ_2-(4-Oxo-4H-pyridin-1-yl)acetato-O,O',O'')-copper(ii))

Space Group:	P-1	***Cell:***	***a*** 7.132(1)	***b*** 7.398(1)	***c*** 7.530(1)
Space Group No.:	2	***(Å,°)***	α 91.46(3)	β 109.14(3)	γ 111.31(3)

R-Factor (%): 2.86 ***Temperature(K):*** 295 ***Density(g/cm^3):*** 1.771

Parameters

Fragment 1

**DIST1 (D)**	1.941
**DIST2 (D)**	1.960
**DIST3 (D)**	1.960
**DIST4 (D)**	1.941
**DIST5 (D)**	2.716
**DIST6 (D)**	2.716

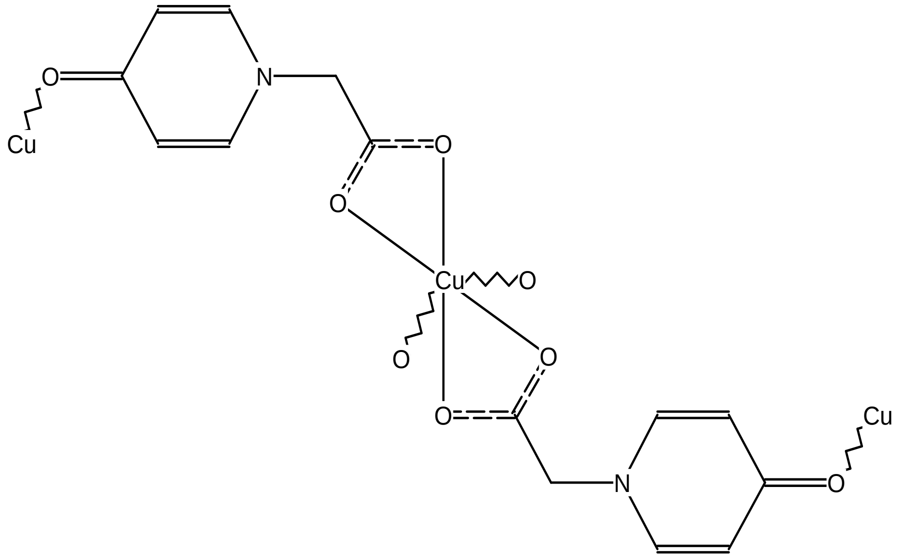

Search: search2 (Wed Jul 26 09:11:25 2017): Hit 93

JUFXOZ

Reference:	W.Clegg, J.M.Holcroft, N.C.Martin (2015) *CrystEngComm* , **17,**2857
Formula:	$(C_{10} H_8 Cu_1 K_2 O_{11})n,H_2 O_1$
Compound Name:	catena-[(μ-benzene-1,2,4,5-tetracarboxylato)-bis(μ-aqua)-aqua-di-potassium-copper monohydrate]

Space Group:	P-1	***Cell:***	***a*** 7.739(1)	***b*** 10.600(1)	***c*** 10.616(1)
Space Group No.:	2	***(Å,°)***	α 112.87(1)	β 90.41(1)	γ 104.19(1)
R-Factor (%):	2.85	***Temperature(K):***	295	***Density(g/cm³):***	1.994

Parameters

Fragment 1

**DIST1 (D)**	1.991
**DIST2 (D)**	1.948
**DIST3 (D)**	1.935
**DIST4 (D)**	1.977
**DIST5 (D)**	2.601
**DIST6 (D)**	2.611

n-$CuK_2[\mu_{12}$-$C_{10}H_2O_8][\mu_2$-$H_2O]_2[H_2O]$

H_2O

Search: search2 (Wed Jul 26 09:11:25 2017): Hit 94

JUFXUF

Reference: W.Clegg, J.M.Holcroft, N.C.Martin (2015) *CrystEngComm* , **17,**2857

Formula: $(C_{10}\ H_{10}\ Cu_{1}\ Na_{2}\ O_{12})n$

Compound Name: catena-[(μ-benzene-1,2,4,5-tetracarboxylato)-tri-aqua-copper-di-sodium]

Space Group:	C2/c	***Cell:***	***a*** 6.894(0)	***b***	16.338(1)	***c***	12.938(0)
Space Group No.:	15	***(Å,°)***	α 90.00	β	99.67(0)	γ	90.00

R-Factor (%): 2.05 ***Temperature(K):*** 160 ***Density(g/cm³):*** 1.996

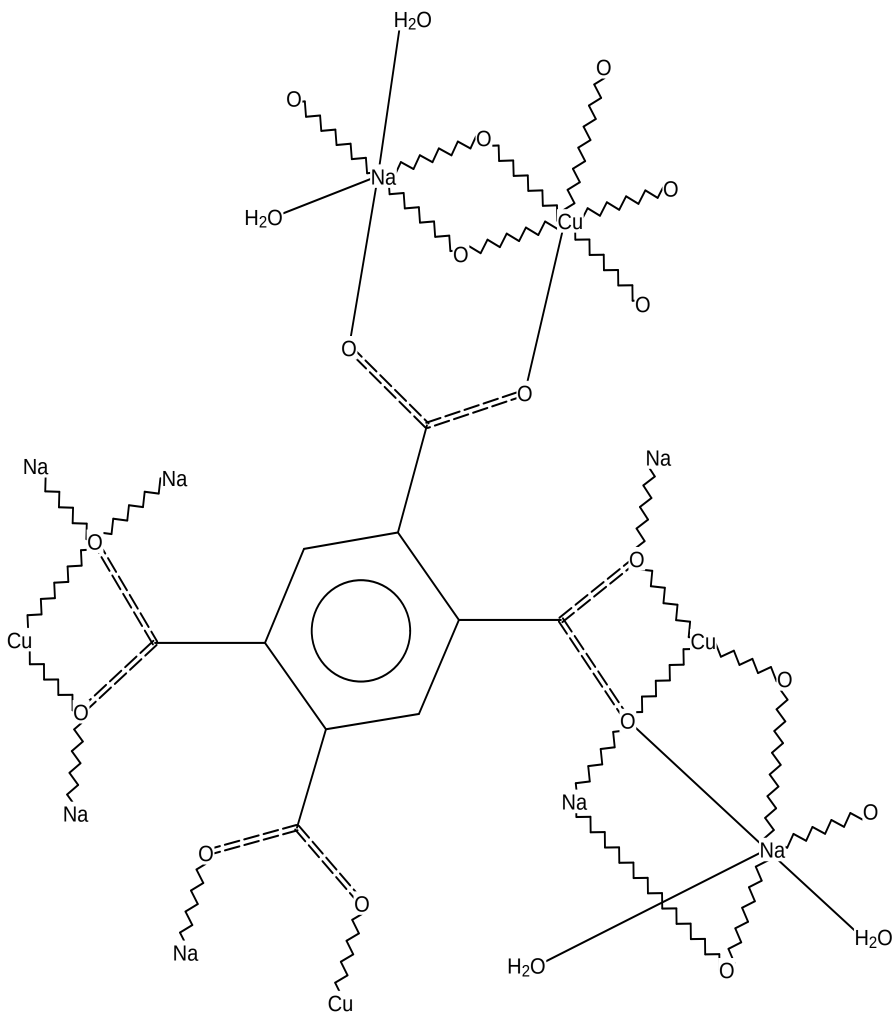

Parameters

Fragment 1

**DIST1 (D)**	1.940
**DIST2 (D)**	1.955
**DIST3 (D)**	1.940
**DIST4 (D)**	1.955
**DIST5 (D)**	2.767
**DIST6 (D)**	2.767

Search: search2 (Wed Jul 26 09:11:25 2017): Hit 95

JUQNEO

Reference:	E.V.Karpova, A.I.Boltalin, M.A.Zakharov, N.I.Sorokina, Y.M.Korenev, S.I.Troyanov (1998) *Z.Anorg.Allg.Chem.* ,**624,**741
Formula:	$C_4 H_8 Cu_1 F_6 O_8$
Compound Name:	Tetra-aqua-bis(trifluoroacetato-O)-copper(ii)

Space Group:	P21	***Cell:***	***a*** 5.064(1)	***b*** 12.091(2)	***c*** 9.537(2)		
Space Group No.:	4	***(Å,°)***	α 90.00	β 99.10(3)	γ 90.00		
R-Factor (%):	2.50	***Temperature(K):***	295	***Density(g/cm^3):***	2.083		

Parameters

Fragment 1

**DIST1 (D)**	1.943
**DIST2 (D)**	1.967
**DIST3 (D)**	2.003
**DIST4 (D)**	1.931
**DIST5 (D)**	2.436
**DIST6 (D)**	2.459

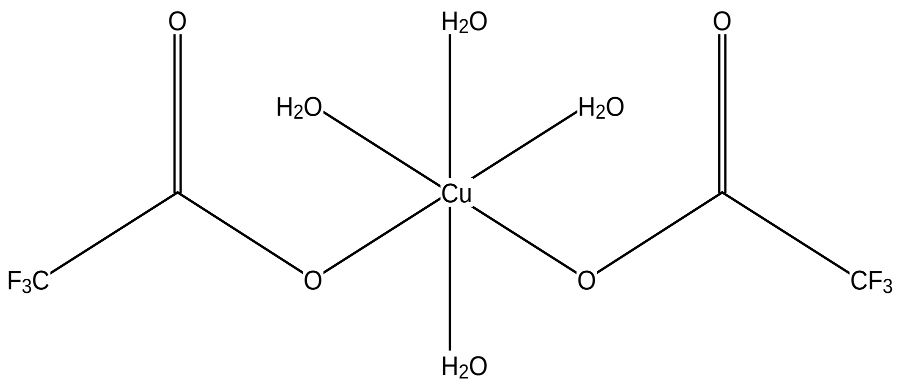

Search: search2 (Wed Jul 26 09:11:25 2017): Hit 96

KCUOXD02

Reference: Jian Wu, Jian-Qing Liu (2010)
Synth.React.Inorg.,Met.-Org.,Nano-Met.Chem. ,**40,**237

Formula: $(C_8 H_8 Cu_2 K_4 O_{20})n$

Compound Name: catena-[bis(μ_8-Oxalato)-bis(μ_5-oxalato)-bis(μ_3-aqua)-bis(μ_2-aqua)-di-copper(ii)-tetra-potassium(i)]

Space Group:	P-1	***Cell:***	***a*** 6.938(2)	***b*** 8.703(3)	***c*** 9.022(3)
Space Group No.:	2	***(Å,°)***	α 108.25(0)	β 99.96(0)	γ 97.19(0)

R-Factor (%): 2.25 ***Temperature(K):*** 296 ***Density(g/cm³):*** 2.350

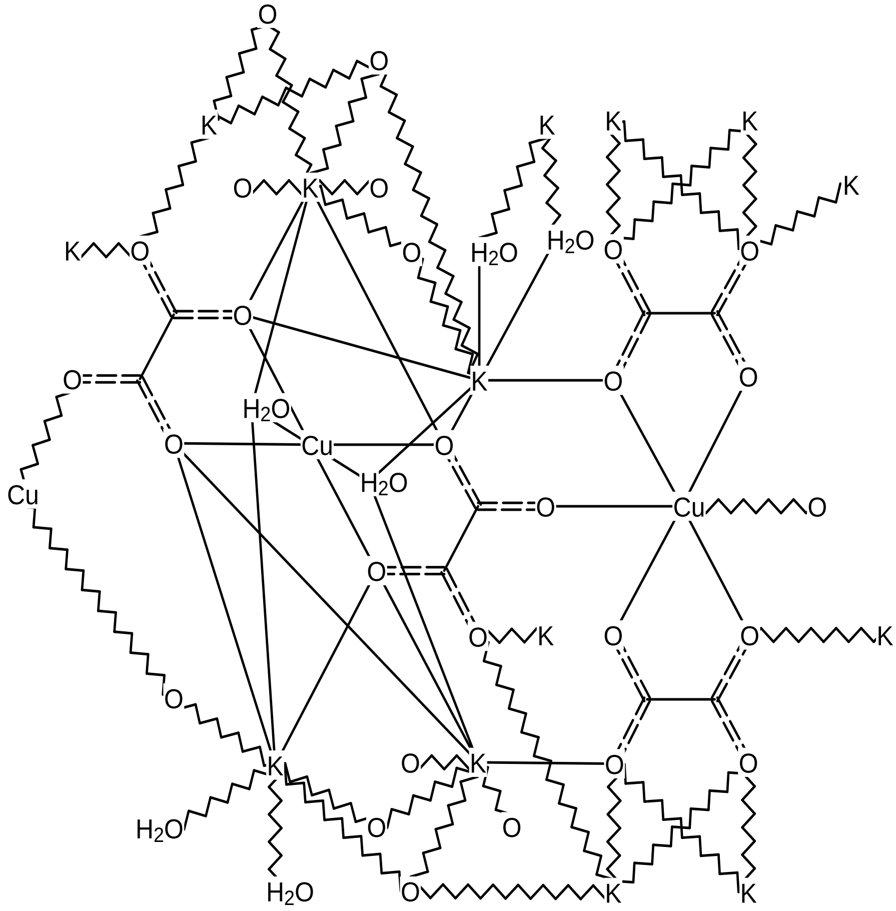

Parameters

Fragment 1

**DIST1 (D)**	1.940
**DIST2 (D)**	1.940
**DIST3 (D)**	1.946
**DIST4 (D)**	1.946
**DIST5 (D)**	2.458
**DIST6 (D)**	2.458

Fragment 2

**DIST1 (D)**	1.950
**DIST2 (D)**	1.934
**DIST3 (D)**	1.950
**DIST4 (D)**	1.934
**DIST5 (D)**	2.622
**DIST6 (D)**	2.622

Search: search2 (Wed Jul 26 09:11:25 2017): Hit 97

KCUOXD03

Reference:	B.Zhang, Y.Zhang, J.Zhang, X.Yan, D.Zhu (2016) *CrystEngComm* ,**18,**5062
Formula:	$(C_8 H_8 Cu_2 K_4 O_{20})n$
Compound Name:	catena-[tetrakis(μ-Oxalato)-tetrakis(μ-aqua)-di-copper-tetra-potassium]

Space Group:	P-1	***Cell:***	***a*** 6.904(2)	***b*** 8.660(3)	***c*** 8.929(3)		
Space Group No.:	2	***(Å,°)***	α 107.95(0)	β 99.64(0)	γ 97.64(0)		
R-Factor (%):	2.83	***Temperature(K):***	173	***Density(g/cm³):***	2.394		

$n\text{-}Cu_2K_4[\mu_8\text{-}C_2O_4]_2[\mu_5\text{-}C_2O_4]_2[\mu_3\text{-}H_2O]_2[\mu_2\text{-}H_2O]_2$

Parameters

Fragment 1

**DIST1 (D)**	1.942
**DIST2 (D)**	1.941
**DIST3 (D)**	1.942
**DIST4 (D)**	1.941
**DIST5 (D)**	2.424
**DIST6 (D)**	2.424

Fragment 2

**DIST1 (D)**	1.931
**DIST2 (D)**	1.951
**DIST3 (D)**	1.931
**DIST4 (D)**	1.951
**DIST5 (D)**	2.583
**DIST6 (D)**	2.583

Search: search2 (Wed Jul 26 09:11:25 2017): Hit 98

KOHXAH

Reference: Junwei Ye, Jingying Zhang, Ling Ye, Dong Xie, Tao Xu, Gang Li, Guiling Ning (2008) *Dalton Trans.* ,5342

Formula: $(C_{48} H_{28} Cu_2 La_2 N_6 O_{40})n,4n(H_2 O_1)$

Compound Name: catena-(bis(μ_4-5-Nitroisophthalato-O,O',O'',O''')-bis(μ_3-5-nitroisophthalato-O,O',O'',O''')-bis(μ_3-hydrogen 5-nitroisophthalato-O,O',O'')-bis(μ_2-aqua)-diaqua-di-copper(ii)-di-lanthanum(iii) tetrahydrate)

Space Group:	P-1	***Cell:***	***a*** 10.845(2)	***b*** 12.041(2)	***c*** 13.634(3)
Space Group No.:	2	***(Å,°)***	α 108.07(3)	β 108.82(3)	γ 103.88(3)

R-Factor (%): 2.22 ***Temperature(K):*** 293 ***Density(g/cm³):*** 2.021

Parameters

Fragment 1

**DIST1 (D)**	1.967
**DIST2 (D)**	1.920
**DIST3 (D)**	1.967
**DIST4 (D)**	1.920
**DIST5 (D)**	2.696
**DIST6 (D)**	2.696

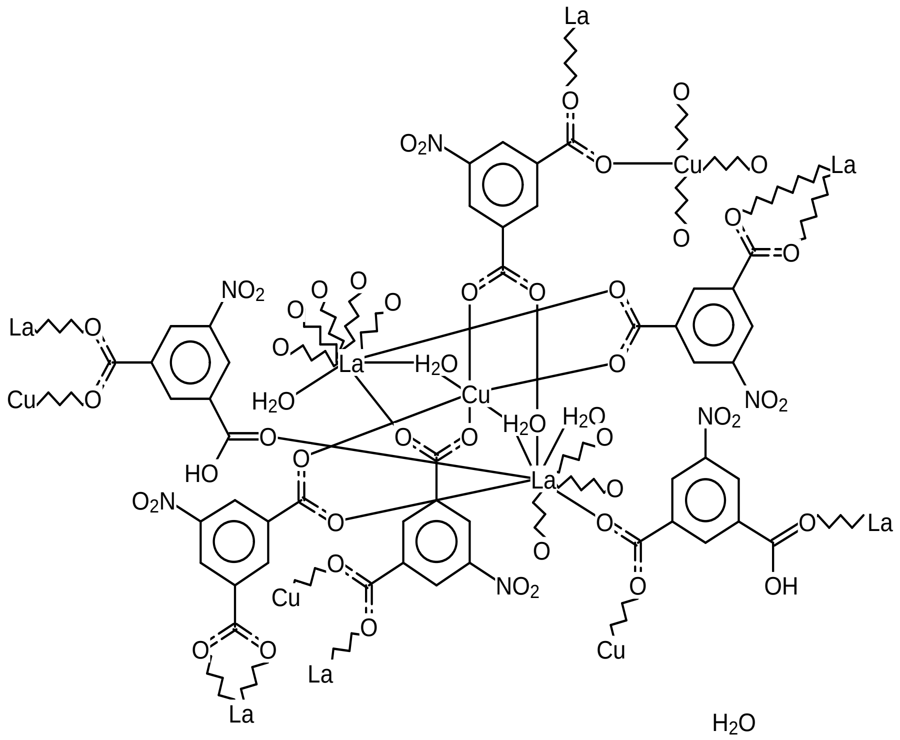

Search: search2 (Wed Jul 26 09:11:25 2017): Hit 99

LAJPEU

Reference: Wenqi Zhao, Zhanhua Su, Zhifeng Zhao, Bowen Cong, Lin Xia, Baibin Zhou (2015) *J.Inorg.Organomet.Polym.Mater.* ,**25,**1373

Formula: $(C_{28} H_{40} As_6 Cu_5 Mo_6 N_{24} O_{30})n,2n(H_2 O_1)$

Compound Name: catena-(bis(μ7- 1,3,5,2,4,6-Trioxatriarsinane-2,4,6-triolato)-tetrakis(μ2-1, 1'-propane-1,3-diylbis(1H-1,2,4-triazole))-decakis(μ2-oxo)-octaoxo-penta-copper-hexa-molybdenum dihydrate)

Space Group:	P-1	***Cell:***	***a*** 9.335(0)	***b*** 13.093(0)	***c*** 14.644(0)
Space Group No.:	2	***(Å,°)***	α 100.94	β 90.73	γ 109.56

R-Factor (%): 2.69 ***Temperature(K):*** 296 ***Density(g/cm³):*** 2.588

Parameters

Fragment 1

**DIST1 (D)**	2.178
**DIST2 (D)**	2.034
**DIST3 (D)**	2.178
**DIST4 (D)**	2.034
**DIST5 (D)**	2.025
**DIST6 (D)**	2.025

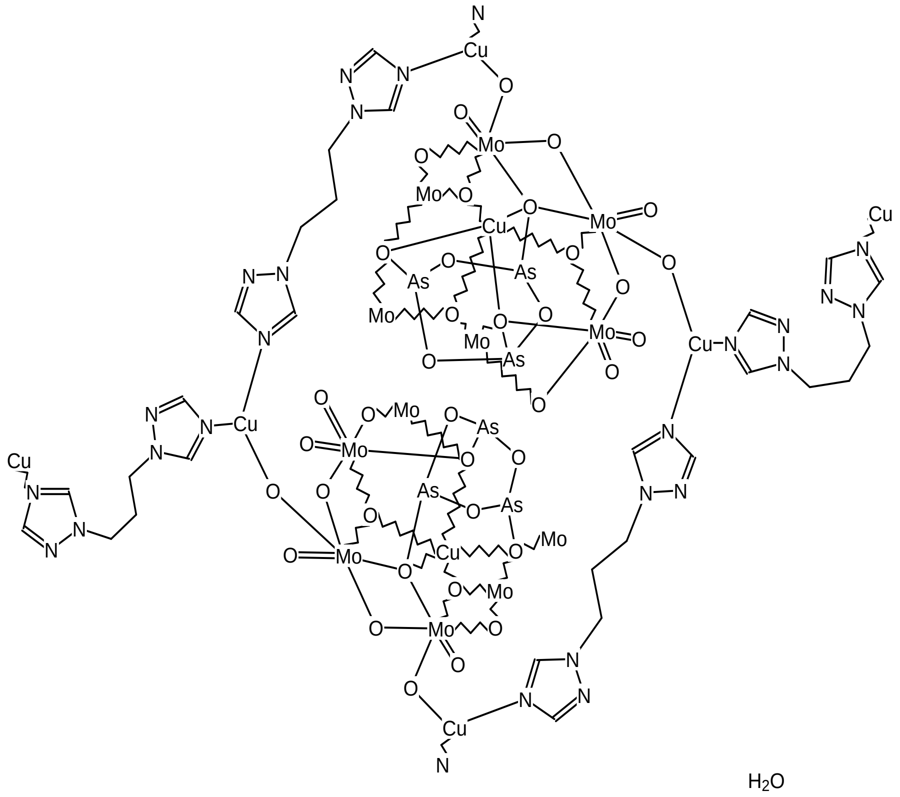

Search: search2 (Wed Jul 26 09:11:25 2017): Hit 100

LICNAN

Reference:	S.R.Choudhury, J.Bhattacharyya, S.Das, B.Dey, S.Mukhopadhyay, Li-Ping Lu, Miao-Li Zhu (2007) *Acta Crystallogr.,Sect.E:Struct.Rep.Online* ,**63,**m1331
Formula:	$2(C_5 H_7 N_2{}^{1+}),C_6 H_8 Cu_1 O_{10}{}^{2-}$
Compound Name:	bis(2-Aminopyridinium) diaqua-bis(malonato-κ^{2}O,O')-copper(ii)

Space Group:	P-1	***Cell:***	***a*** 7.043(1)	***b*** 7.912(1)	***c*** 9.577(1)
Space Group No.:	2	***(Å,°)***	α 96.10(0)	β 107.87(0)	γ 103.74(0)
R-Factor (%):	2.75	***Temperature(K):***	298	***Density(g/cm^3):***	1.694

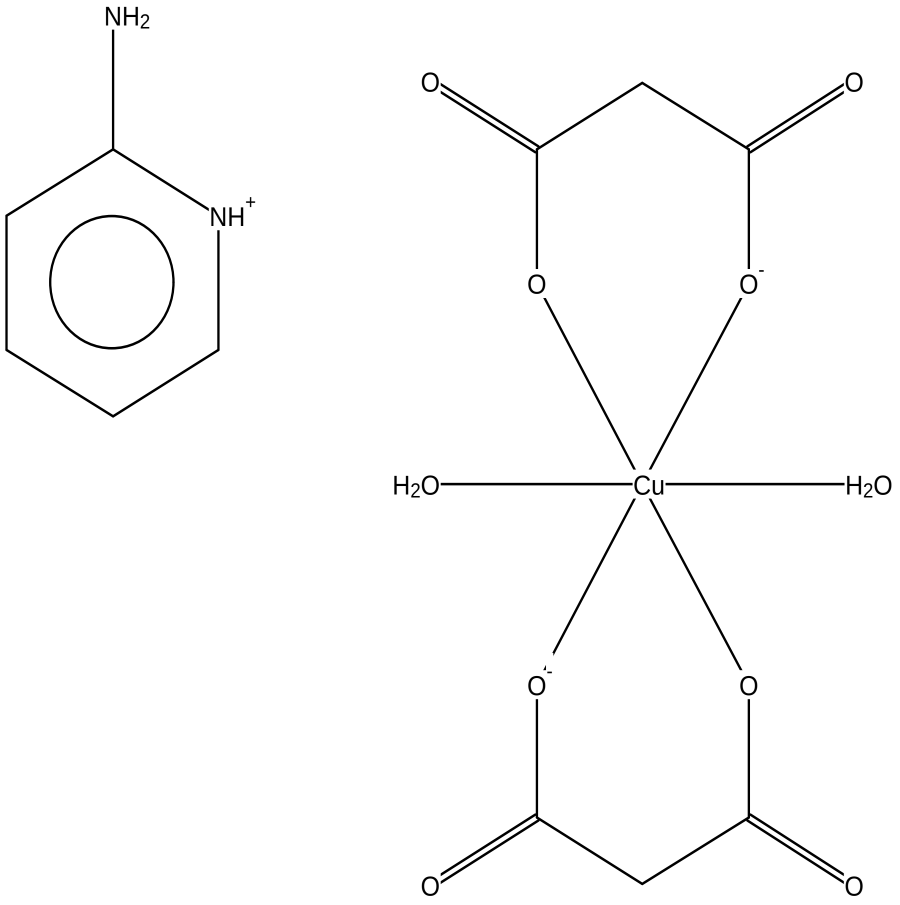

Parameters

Fragment 1

**DIST1 (D)**	1.929
**DIST2 (D)**	1.934
**DIST3 (D)**	1.934
**DIST4 (D)**	1.929
**DIST5 (D)**	2.665
**DIST6 (D)**	2.665

Search: search2 (Wed Jul 26 09:11:25 2017): Hit 101

LICNAN01

Reference: S.R.Choudhury, P.Gamez, A.Robertazzi, Chih-Yuan Chen, Hon Man Lee, S.Mukhopadhyay (2008) *Cryst.Growth Des.* ,**8,**3773

Formula: $2(C_5 H_7 N_2{}^{1+}),C_6 H_8 Cu_1 O_{10}{}^{2-}$

Compound Name: bis(2-Aminopyridinium) trans-diaqua-bis(malonato)-copper(ii)

Space Group:	P-1	***Cell:***	***a*** 6.995(0)	***b*** 7.887(0)	***c*** 9.556(1)
Space Group No.:	2	***(Å,°)***	α 96.30(0)	β 108.47(0)	γ 104.09(0)

R-Factor (%): 2.15 ***Temperature(K):*** 150 ***Density(g/cm³):*** 1.727

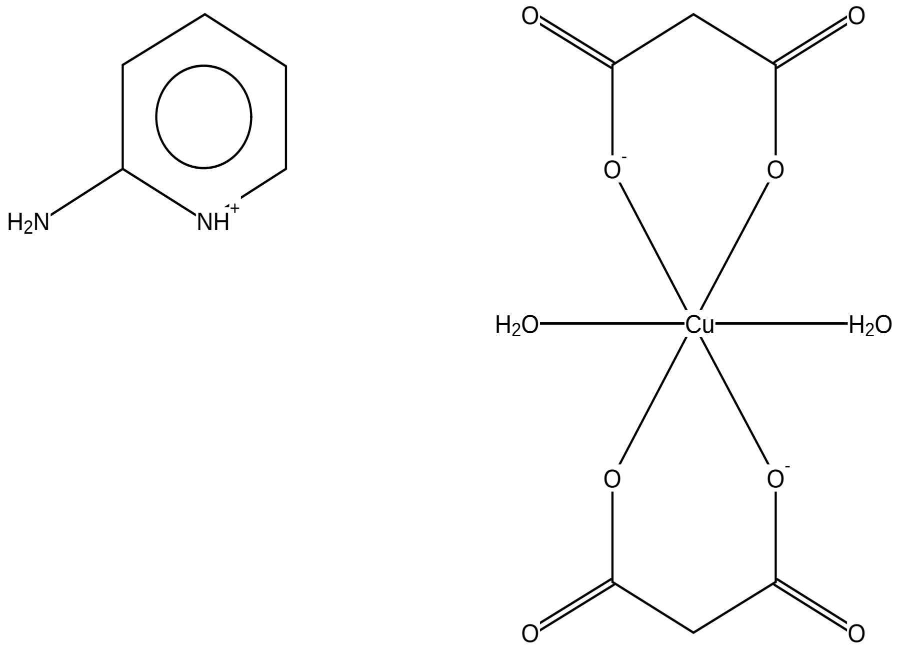

Parameters

Fragment 1

**DIST1 (D)**	1.937
**DIST2 (D)**	1.933
**DIST3 (D)**	1.937
**DIST4 (D)**	1.933
**DIST5 (D)**	2.621
**DIST6 (D)**	2.621

Search: search2 (Wed Jul 26 09:11:25 2017): Hit 102

LIKZAI

Reference:	Shujuan An, Bing Yin, Ping Liu, Xiangnan Li, Chen Li, Jianli Li, Zhen Shi (2013) *Synthesis* ,**45,**2525
Formula:	$(C_{16} H_{12} Cu_1 O_6)n$
Compound Name:	catena-[bis(μ_2-oxido(phenyl)acetato)-copper]

Space Group:	P21/c	***Cell:***	***a*** 15.619(3)	***b*** 4.954(1)	***c*** 9.548(1)		
Space Group No.:	14	***(Å,°)***	α 90.00	β 102.76(0)	γ 90.00		
R-Factor (%):	2.75	***Temperature(K):***	296	***Density(g/cm³):***	1.677		

Parameters

Fragment 1

**DIST1 (D)**	1.936
**DIST2 (D)**	2.005
**DIST3 (D)**	2.005
**DIST4 (D)**	1.936
**DIST5 (D)**	2.452
**DIST6 (D)**	2.452

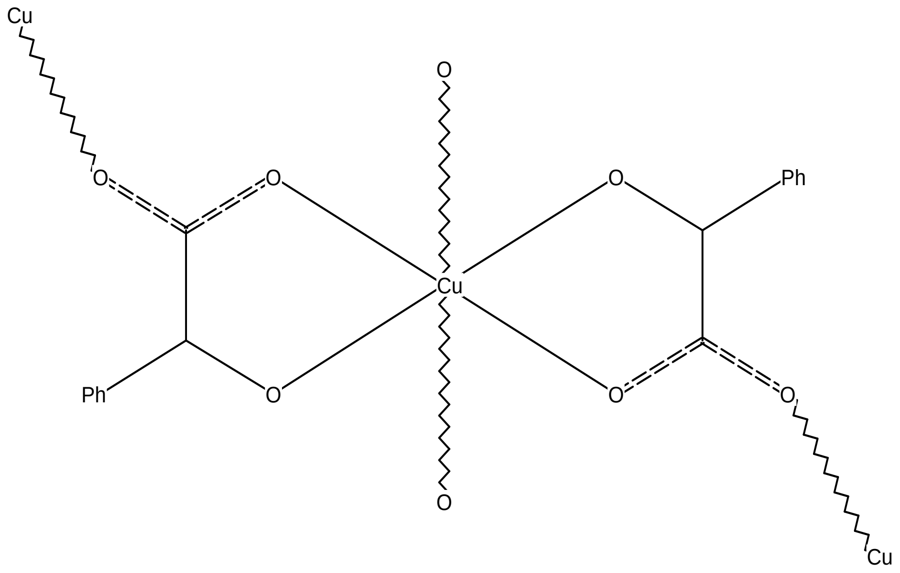

Search: search2 (Wed Jul 26 09:11:25 2017): Hit 103

LUCWAJ

Reference:	I.Yousuf, F.Arjmand, S.Tabassum, L.Toupet, R.A.Khan, M.A.Siddiqui (2015) *Dalton Trans.* ,**44,**10330
Formula:	C_{24} H_{26} Cu_{1} O_{10}
Compound Name:	bis(Methanol)-bis(3-(oxymethylene)-2-methoxy-2,3-dihydro-4H-chromen-4-one)-copper(ii)

Space Group:	P21/c	***Cell:***	***a*** 8.751(0)	***b*** 5.077(0)	***c*** 26.223(0)
Space Group No.:	14	***(Å,°)***	α 90.00	β 96.30(0)	γ 90.00
R-Factor (%):	2.84	***Temperature(K):*** 140		***Density(g/cm³):*** 1.543	

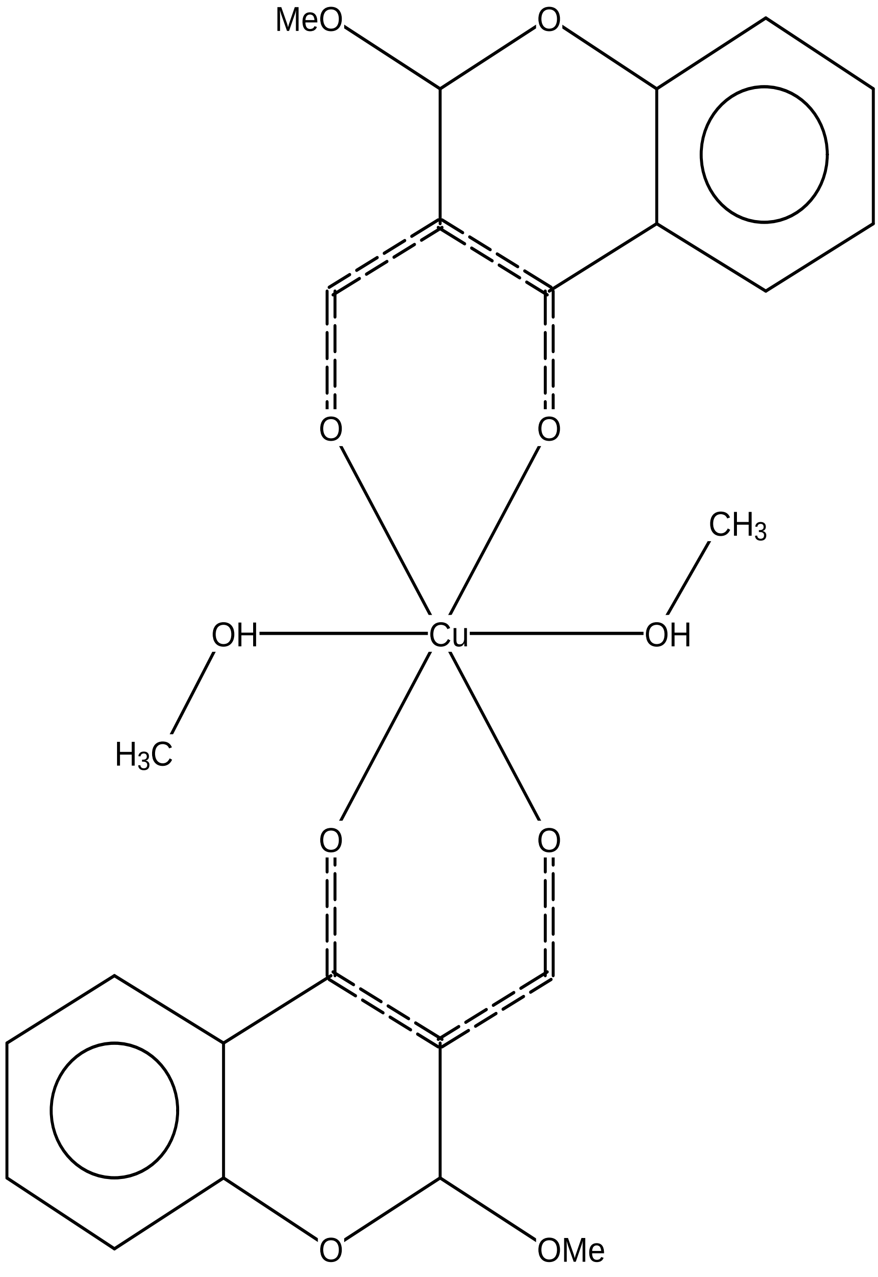

Parameters

Fragment 1

**DIST1 (D)**	1.944
**DIST2 (D)**	1.951
**DIST3 (D)**	1.944
**DIST4 (D)**	1.951
**DIST5 (D)**	2.397
**DIST6 (D)**	2.397

Search: search2 (Wed Jul 26 09:11:25 2017): Hit 104

LUTTEA

Reference: Yue-Qing Zheng, Xiao-Yan Han, Hong-Lin Zhu (2010)
Polyhedron ,**29,**911

Formula: $(C_8\ H_{16}\ Cu_1\ Na_2\ O_{15})n$

Compound Name: catena-[(μ_8-Tetrahydrofuran-2,3,4,5-tetracarboxylato)-(μ_2-aqua)-pentaaqua-copper(ii)-di-sodium]

Space Group:	P21/c	***Cell:***	***a*** 8.276(1)	***b***	12.727(3)	***c***	15.090(3)
Space Group No.:	14	***(Å,°)***	α 90.00	β	90.08(3)	γ	90.00

R-Factor (%): 2.72 ***Temperature(K):*** 293 ***Density(g/cm^3):*** 1.930

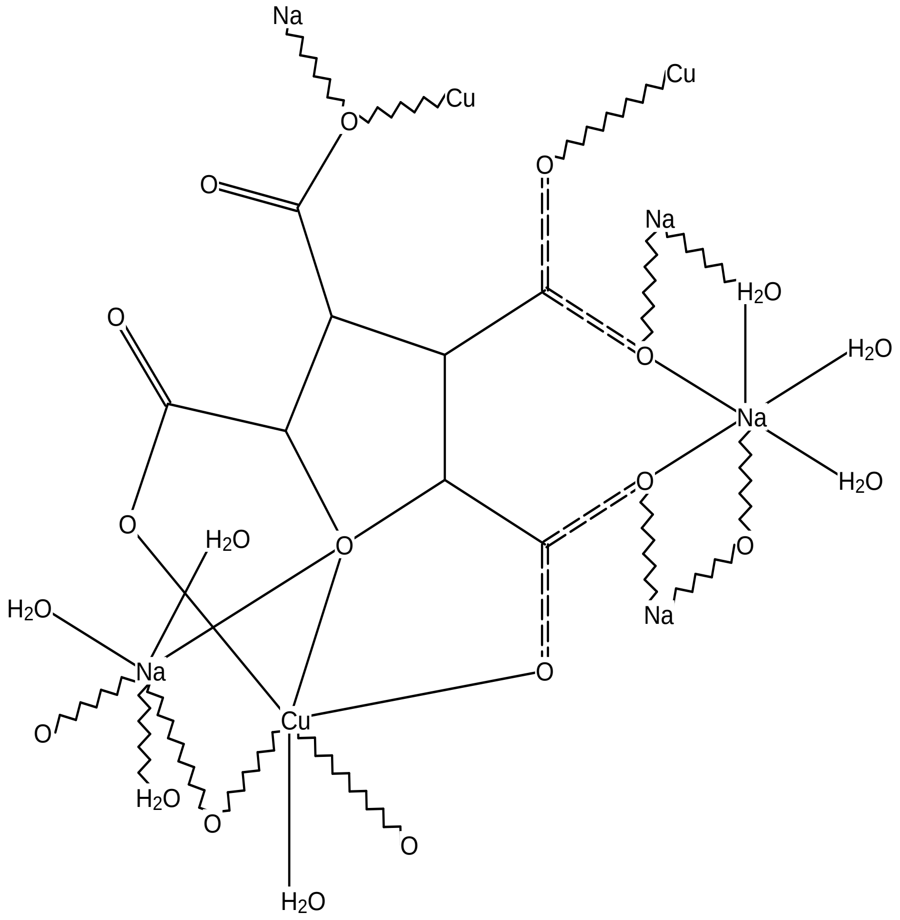

Parameters

Fragment 1

**DIST1 (D)**	1.979
**DIST2 (D)**	1.976
**DIST3 (D)**	1.966
**DIST4 (D)**	1.924
**DIST5 (D)**	2.426
**DIST6 (D)**	2.544

Search: search2 (Wed Jul 26 09:11:25 2017): Hit 105

LUVMUL01

Reference:	B.Demoro, F.Caruso, M.Rossi, D.Benitez, M.Gonzalez, H.Cerecetto, B.Parajan-Costa, J.Castiglioni, M.Galizzi, R.Docampo, L.Otero, D.Gambino (2010) *J.Inorg.Biochem.* ,**104,**1252
Formula:	$C_{14}\ H_{20}\ Cu_1\ N_2\ O_{14}\ P_4,4(H_2\ O_1)$
Compound Name:	bis(dihydrogen 1-hydroxy-2-(3-pyridinio)ethane-1,1-diyldiphosphonate)-copper(ii) tetrahydrate
Synonym:	bis(risedronato)-copper(ii) tetrahydrate

Space Group:	P-1	***Cell:***	***a***	7.651(0)	***b***	8.587(0)	***c***	10.279(0)
Space Group No.:	2	***(Å,°)***	α	65.95(0)	β	84.97(0)	γ	78.88(0)

R-Factor (%): 2.43 ***Temperature(K):*** 125 ***Density(g/cm³):*** 1.921

Parameters

Fragment 1

**DIST1 (D)**	1.946
**DIST2 (D)**	1.960
**DIST3 (D)**	1.946
**DIST4 (D)**	1.960
**DIST5 (D)**	2.655
**DIST6 (D)**	2.655

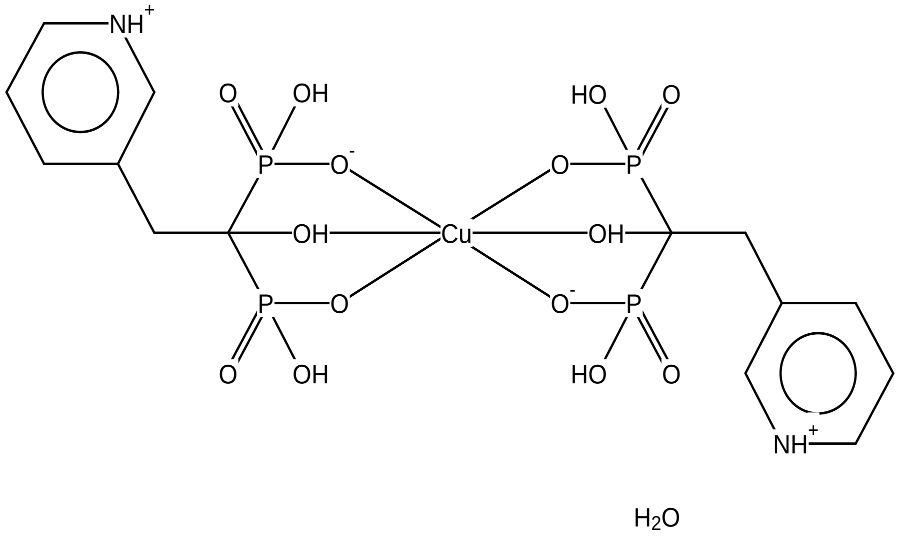

Search: search2 (Wed Jul 26 09:11:25 2017): Hit 106

LUVZUY

Reference:	T.E.Knight, J.K.McCusker (2010) *J.Am.Chem.Soc.* ,**132,** 2208
Formula:	$C_{48} H_{36} Cu_1 F_6 N_6 O_{16} Re_2 S_2,2(C_2 H_3 N_1)$
Compound Name:	bis(μ_2-3-(4-Pyridyl)acetylacetonato)-bis(2,2'-bipyridine)-hexacarbonyl-bis(trifluoromethanesulfonato)-copper(ii)-di-rhenium(ii) acetonitrile solvate

Space Group:	P21/c	***Cell:***	***a*** 10.321(1)	***b*** 24.532(3)	***c*** 12.161(1)
Space Group No.:	14	***(Å,°)***	α 90.00	β 104.03(0)	γ 90.00

R-Factor (%): 1.89 ***Temperature(K):*** 173 ***Density(g/cm³):*** 1.833

Parameters

Fragment 1

**DIST1 (D)**	1.920
**DIST2 (D)**	1.914
**DIST3 (D)**	1.920
**DIST4 (D)**	1.914
**DIST5 (D)**	2.705
**DIST6 (D)**	2.705

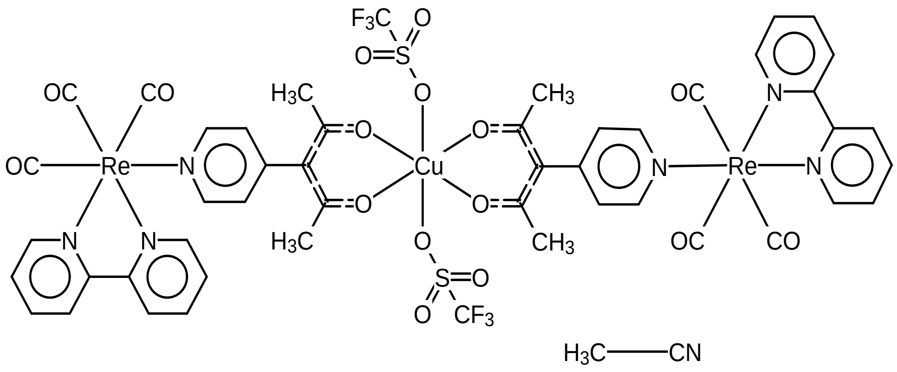

Search: search2 (Wed Jul 26 09:11:25 2017): Hit 107

MACUFR

Reference:	E.Sletten, L.H.Jensen (1973)<br>*Acta Crystallogr.,Sect.B:Struct.Crystallogr.Cryst.Chem. ,***29,**1752						
Formula:	$(C_3 H_3 Cu_1 O_6{}^{1-})n,n(C_2 H_8 N_1{}^{1+})$						
Compound Name:	catena-(dimethylammonium tris(μ_2-formato)-copper(ii))						
Space Group:	I2/c	***Cell:***	***a*** 11.420(0)		***b*** 8.714(0)		***c*** 8.850(0)
Space Group No.:	15	***(Å,°)***	α 90.00		β 96.25(0)		γ 90.00
R-Factor (%):	1.70	***Temperature(K):***	295		***Density(g/cm^3):***		1.856

Parameters

Fragment 1

**DIST1 (D)**	1.971
**DIST2 (D)**	1.969
**DIST3 (D)**	1.971
**DIST4 (D)**	1.969
**DIST5 (D)**	2.491
**DIST6 (D)**	2.491

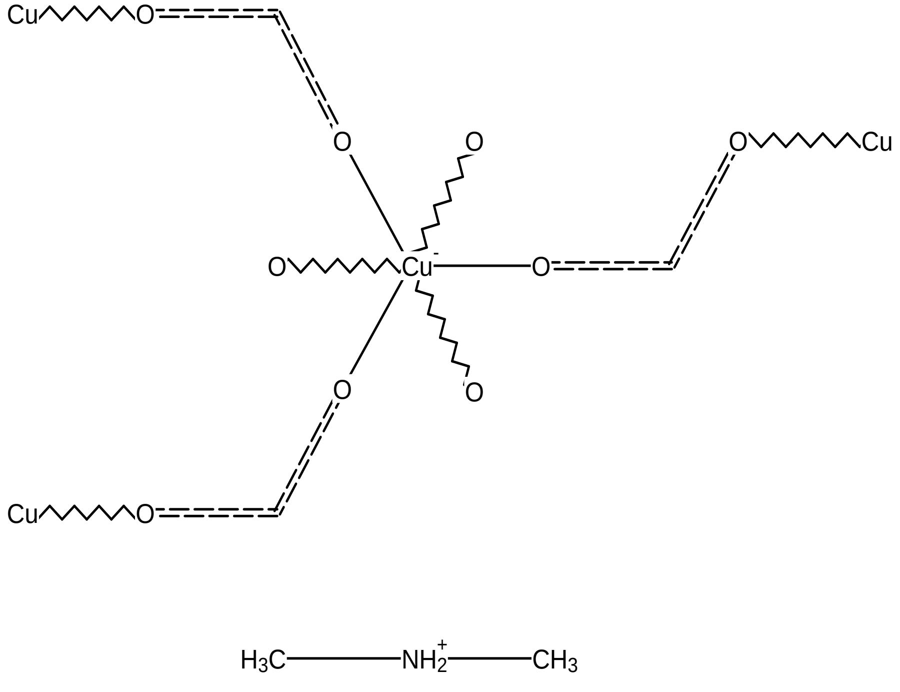

Search: search2 (Wed Jul 26 09:11:25 2017): Hit 108

MACUFR01

Reference: Deyuan Kong, J.Zon, J.McBee, A.Clearfield (2006)
Inorg.Chem. ,**45,**977

Formula: $(C_3\ H_3\ Cu_1\ O_6\ ^{1-})n,n(C_2\ H_8\ N_1\ ^{1+})$

Compound Name: catena-(dimethylammonium tris(μ_2-formato)-copper)

Space Group:	C2/c	***Cell:***	***a*** 13.733(12)	***b***	8.659(8)	***c***	8.808(8)
Space Group No.:	15	***(Å,°)***	α 90.00	β	124.08(1)	γ	90.00

R-Factor (%): 1.83 ***Temperature(K):*** 110 ***Density(g/cm^3):*** 1.874

Parameters

Fragment 1

**DIST1 (D)**	1.975
**DIST2 (D)**	1.975
**DIST3 (D)**	1.978
**DIST4 (D)**	1.978
**DIST5 (D)**	2.456
**DIST6 (D)**	2.456

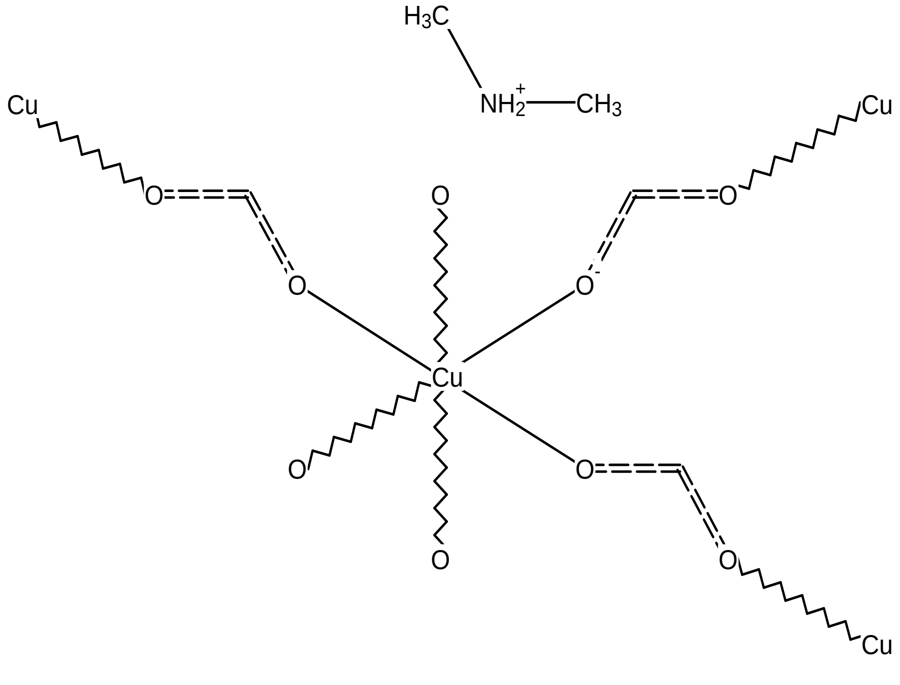

Search: search2 (Wed Jul 26 09:11:25 2017): Hit 109

MACUFR03

Reference:	F.R.Fronczek (2015)<br>*CSD Communication(Private Communication) ,*
Formula:	$(C_3\ H_3\ Cu_1\ O_6\ ^{1-})n,n(C_2\ H_8\ N_1\ ^{1+})$
Compound Name:	catena-[dimethylammonium tris(μ-formato)-copper]

Space Group:	C2/c	***Cell:***	***a***	13.695(0)	***b***	8.632(0)	***c***	8.769(0)
Space Group No.:	15	***(Å,°)***	α	90.00	β	124.19(0)	γ	90.00
R-Factor (%):	1.45	***Temperature(K):***	90		***Density(g/cm³):***	1.895		

Parameters

Fragment 1

**DIST1 (D)**	1.967
**DIST2 (D)**	1.970
**DIST3 (D)**	1.967
**DIST4 (D)**	1.970
**DIST5 (D)**	2.447
**DIST6 (D)**	2.447

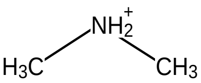

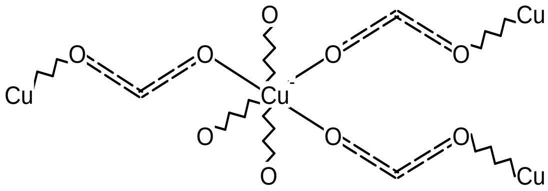

Search: search2 (Wed Jul 26 09:11:25 2017): Hit 110

MAGMOZ

Reference: Ran Shang, Sa Chen, Bing-Wu Wang, Zhe-Ming Wang, Song Gao (2016) *Angew.Chem.,Int.Ed.* ,**55,**2097

Formula: $(C_3 H_3 Cu_1 O_6{}^{1-})n,C_2 H_8 N_1{}^{1+}$

Compound Name: catena-[ethylammonium tris(μ_2-formato)-copper]

Space Group:	Pna21	***Cell:***	***a*** 9.201(0)	***b*** 8.115(0)	***c*** 11.442(0)		
Space Group No.:	33	***(Å,°)***	α 90.00	β 90.00	γ 90.00		
R-Factor (%):	2.09	***Temperature(K):***	291	***Density(g/cm³):***	1.902		

Parameters

Fragment 1

**DIST1 (D)**	1.958
**DIST2 (D)**	2.015
**DIST3 (D)**	1.963
**DIST4 (D)**	2.009
**DIST5 (D)**	2.486
**DIST6 (D)**	2.407

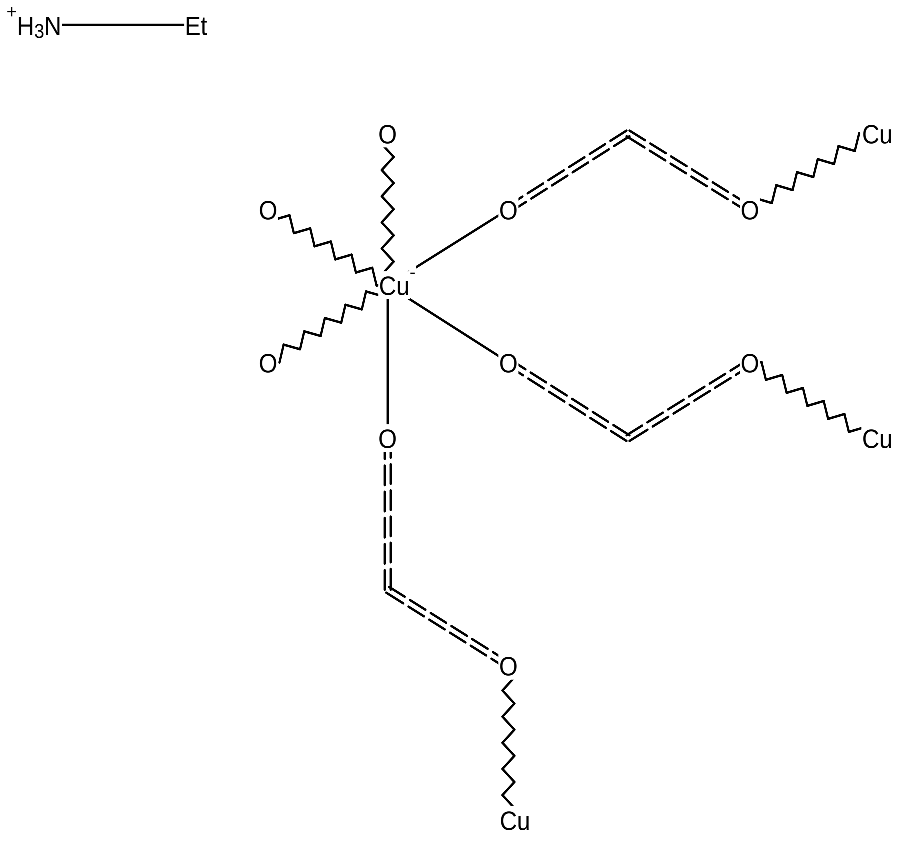

Search: search2 (Wed Jul 26 09:11:25 2017): Hit 111

MAGMOZ01

Reference: Ran Shang, Sa Chen, Bing-Wu Wang, Zhe-Ming Wang, Song Gao (2016) *Angew.Chem.,Int.Ed.* ,**55,**2097

Formula: $(C_3 H_3 Cu_1 O_6{}^{1-})n,C_2 H_8 N_1{}^{1+}$

Compound Name: catena-[ethylammonium tris(μ_2-formato)-copper]

Space Group:	Pna21	***Cell:***	***a***	9.216(0)	***b***	8.118(0)	***c***	11.459(0)
Space Group No.:	33	***(Å,°)***	α	90.00	β	90.00	γ	90.00

R-Factor (%): 2.65 ***Temperature(K):*** 320 ***Density(g/cm3):*** 1.896

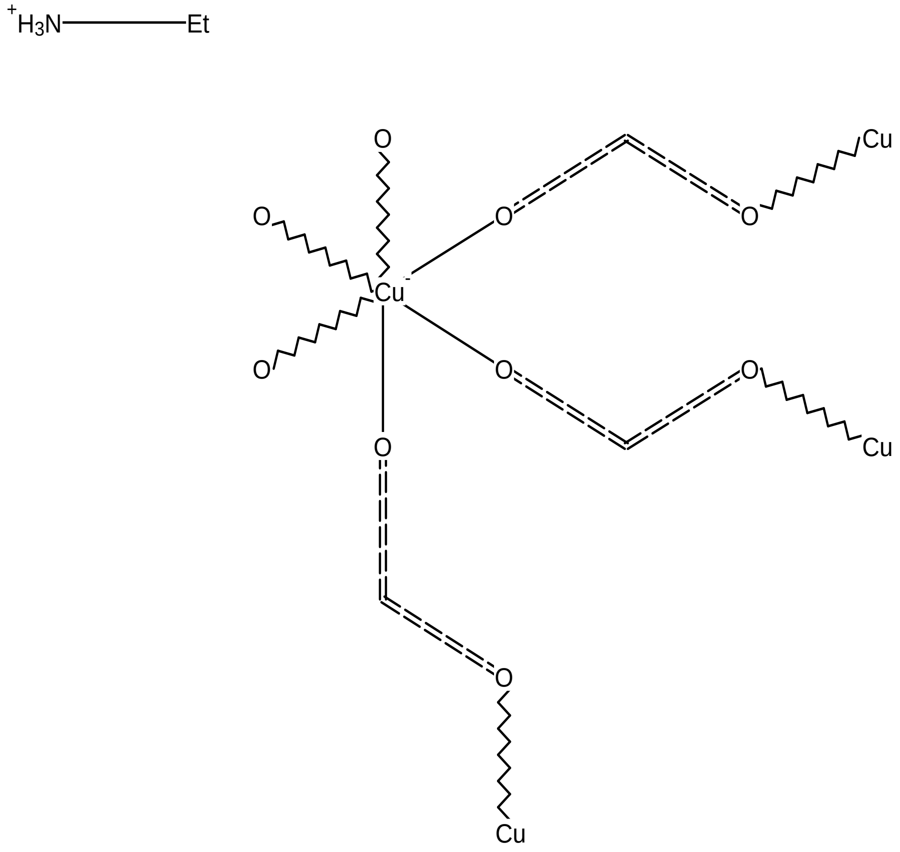

Parameters

Fragment 1

**DIST1 (D)**	1.954
**DIST2 (D)**	2.020
**DIST3 (D)**	1.962
**DIST4 (D)**	2.008
**DIST5 (D)**	2.495
**DIST6 (D)**	2.416

Search: search2 (Wed Jul 26 09:11:25 2017): Hit 112

MAGMOZ02

Reference:	Ran Shang, Sa Chen, Bing-Wu Wang, Zhe-Ming Wang, Song Gao (2016) *Angew.Chem.,Int.Ed.* ,**55,**2097
Formula:	$(C_3\ H_3\ Cu_1\ O_6\ ^{1-})n,C_2\ H_8\ N_1\ ^{1+}$
Compound Name:	catena-[ethylammonium tris(μ_2-formato)-copper]

Space Group:	Pna21	***Cell:***	***a*** 9.209(0)	***b*** 8.110(0)	***c*** 11.445(0)
Space Group No.:	33	***(Å,°)***	α 90.00	β 90.00	γ 90.00
R-Factor (%):	2.36	***Temperature(K):***	340	***Density(g/cm³):***	1.901

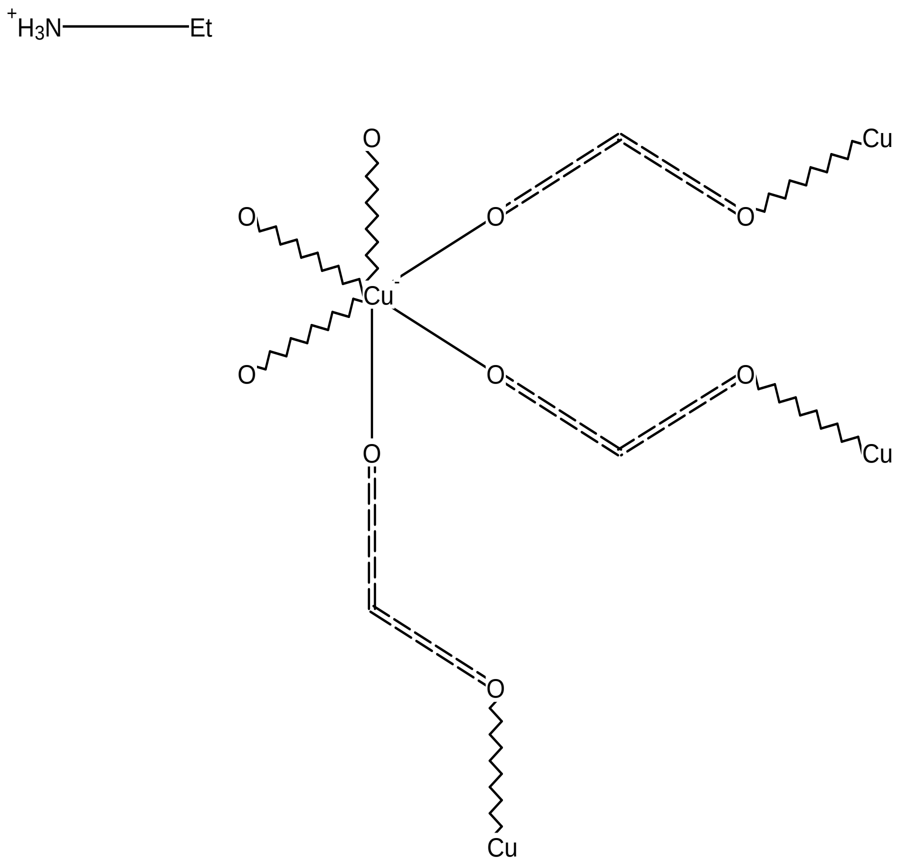

Parameters

Fragment 1

**DIST1 (D)**	1.951
**DIST2 (D)**	2.012
**DIST3 (D)**	1.956
**DIST4 (D)**	2.005
**DIST5 (D)**	2.491
**DIST6 (D)**	2.418

Search: search2 (Wed Jul 26 09:11:25 2017): Hit 113

MAGMOZ03

Reference: Ran Shang, Sa Chen, Bing-Wu Wang, Zhe-Ming Wang, Song Gao (2016) *Angew.Chem.,Int.Ed.* ,**55,**2097

Formula: $(C_3\ H_3\ Cu_1\ O_6\ ^{1-})n,C_2\ H_8\ N_1\ ^{1+}$

Compound Name: catena-[ethylammonium tris(μ-formato)-copper]

Space Group:	Pna21	***Cell:***	***a***	9.103(0)	***b***	8.093(0)	***c***	11.396(0)
Space Group No.:	33	***(Å,°)***	α	90.00	β	90.00	γ	90.00

R-Factor (%): 1.87 ***Temperature(K):*** 93 ***Density(g/cm^3):*** 1.936

Parameters

Fragment 1

**DIST1 (D)**	1.960
**DIST2 (D)**	2.017
**DIST3 (D)**	1.962
**DIST4 (D)**	2.008
**DIST5 (D)**	2.450
**DIST6 (D)**	2.366

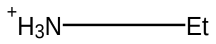

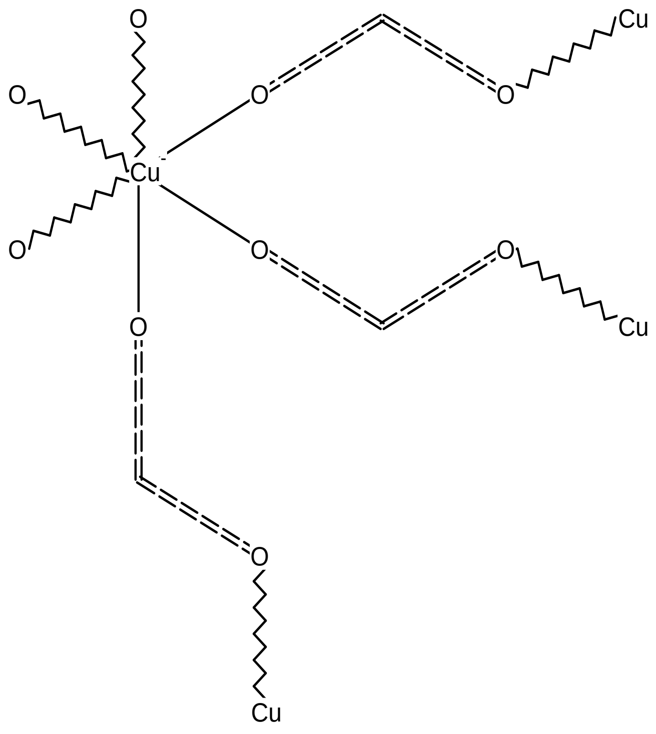

Search: search2 (Wed Jul 26 09:11:25 2017): Hit 114

MAGMOZ04

Reference: Ran Shang, Sa Chen, Bing-Wu Wang, Zhe-Ming Wang, Song Gao (2016) *Angew.Chem.,Int.Ed.* ,**55,**2097

Formula: $(C_3\ H_3\ Cu_1\ O_6\ ^{1-})n,C_2\ H_8\ N_1\ ^{1+}$

Compound Name: catena-[ethylammonium tris(μ-formato)-copper]

Space Group:	Pna21	***Cell:***	***a*** 9.142(0)	***b*** 8.102(0)	***c*** 11.415(0)
Space Group No.:	33	***(Å,°)***	α 90.00	β 90.00	γ 90.00

R-Factor (%): 1.96 ***Temperature(K):*** 180 ***Density(g/cm³):*** 1.922

Parameters

Fragment 1

**DIST1 (D)**	1.959
**DIST2 (D)**	2.017
**DIST3 (D)**	1.962
**DIST4 (D)**	2.009
**DIST5 (D)**	2.465
**DIST6 (D)**	2.382

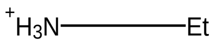

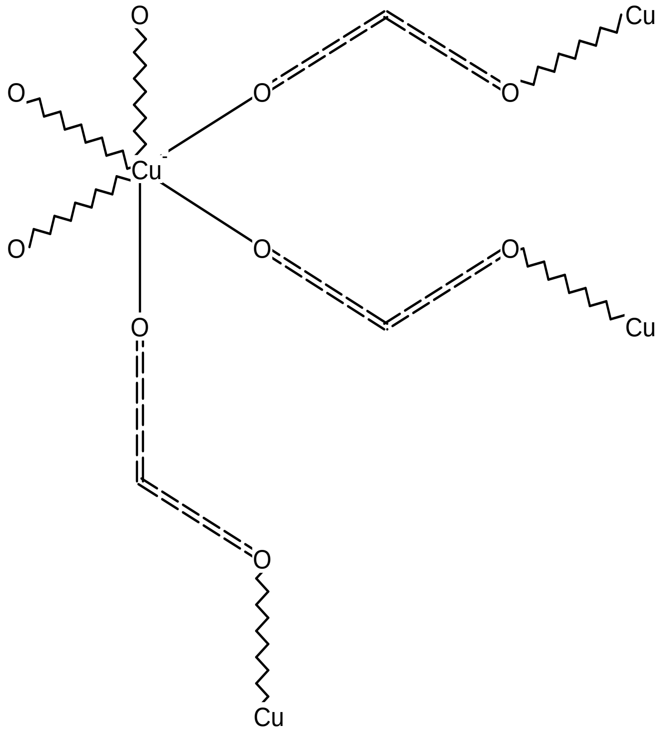

Search: search2 (Wed Jul 26 09:11:25 2017): Hit 115

MAGNEQ

Reference:	Ran Shang, Sa Chen, Bing-Wu Wang, Zhe-Ming Wang, Song Gao (2016) *Angew.Chem.,Int.Ed.* ,**55,**2097
Formula:	$(C_3\ H_3\ Cu_1\ O_6\ ^{1-})n,C_2\ H_8\ N_1\ ^{1+}$
Compound Name:	catena-[ethylammonium bis(μ_2-formato)-(formato)-copper]

Space Group:	P212121	***Cell:***	***a*** 10.034(0)	***b*** 8.283(0)	***c*** 11.158(0)
Space Group No.:	19	***(Å,°)***	α 90.00	β 90.00	γ 90.00
R-Factor (%):	2.86	***Temperature(K):*** 100	***Density(g/cm³):*** 1.753		

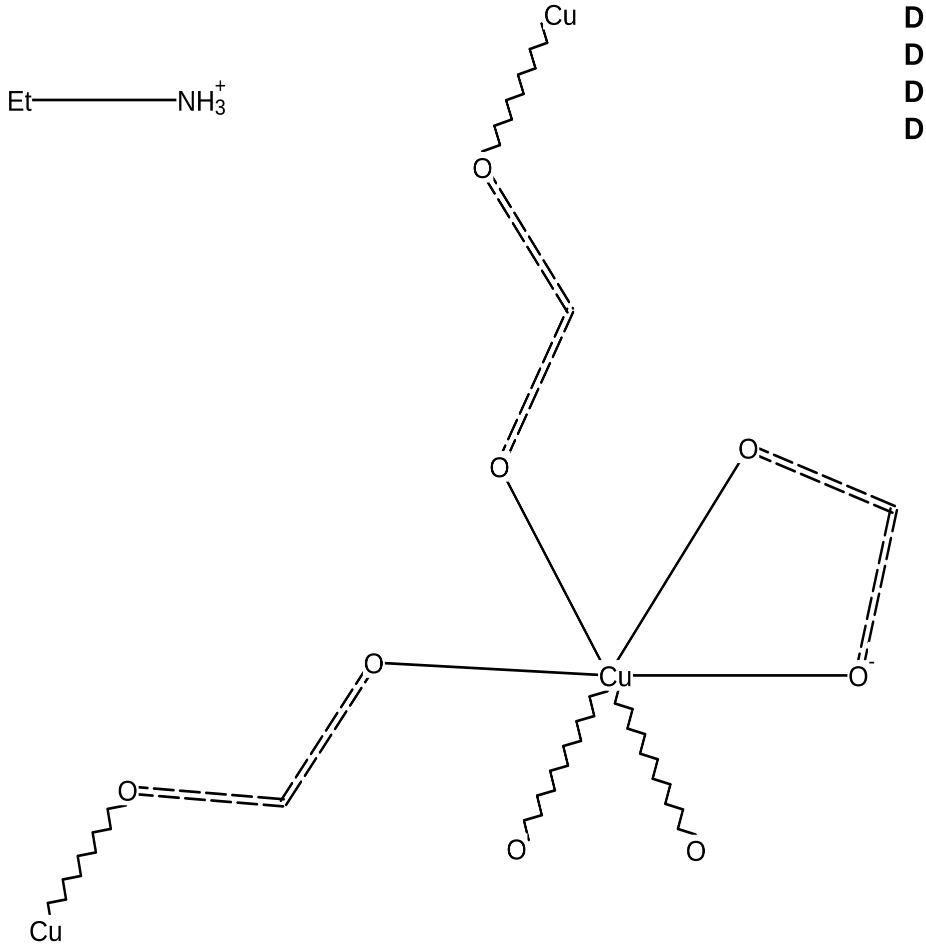

Parameters

Fragment 1

**DIST1 (D)**	1.954
**DIST2 (D)**	2.006
**DIST3 (D)**	1.959
**DIST4 (D)**	1.992
**DIST5 (D)**	2.713
**DIST6 (D)**	2.261

Search: search2 (Wed Jul 26 09:11:25 2017): Hit 116

MAGNEQ01

Reference: Ran Shang, Sa Chen, Bing-Wu Wang, Zhe-Ming Wang, Song Gao (2016) *Angew.Chem.,Int.Ed.* ,**55,**2097

Formula: $(C_3 H_3 Cu_1 O_6{}^{1-})n, C_2 H_8 N_1{}^{1+}$

Compound Name: catena-[ethylammonium bis(μ_2-formato)-(formato)-copper]

Space Group:	P212121	***Cell:***	***a*** 10.073(0)	***b***	8.279(0)	***c***	11.154(0)
Space Group No.:	19	***(Å,°)***	α 90.00	β	90.00	γ	90.00

R-Factor (%): 2.58 ***Temperature(K):*** 180 ***Density(g/cm3):*** 1.747

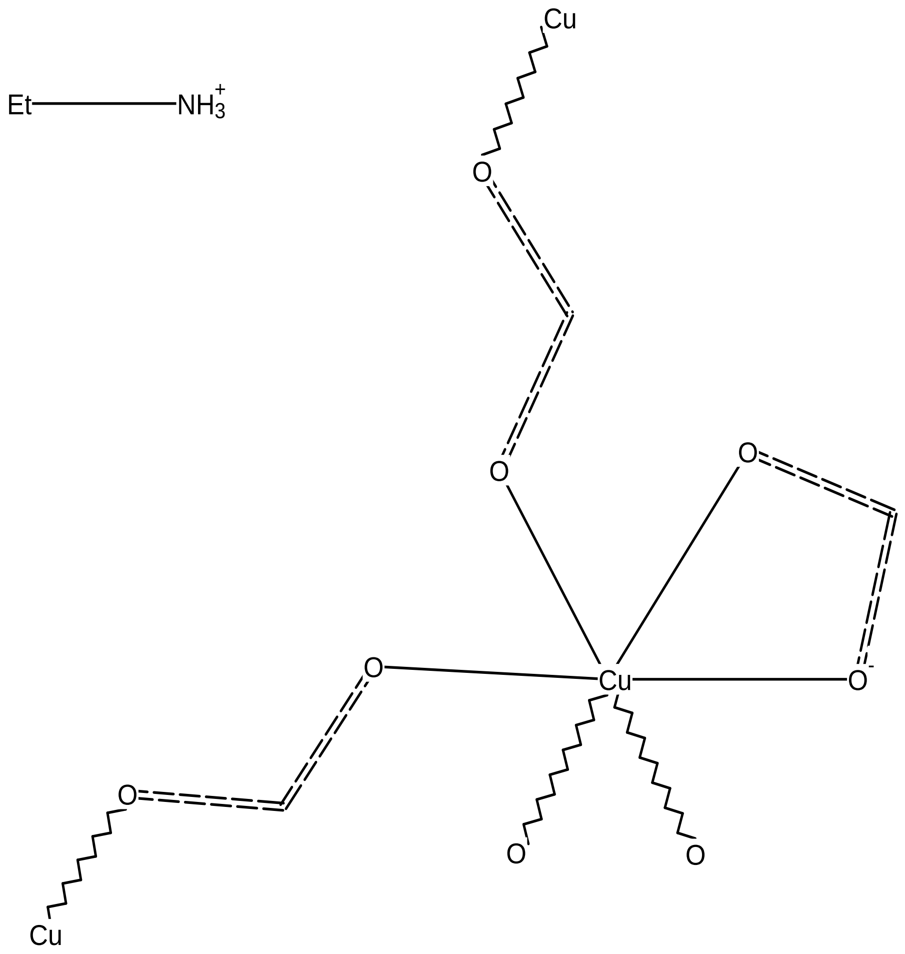

Parameters

Fragment 1

**DIST1 (D)**	1.953
**DIST2 (D)**	2.003
**DIST3 (D)**	1.957
**DIST4 (D)**	1.986
**DIST5 (D)**	2.717
**DIST6 (D)**	2.268

Search: search2 (Wed Jul 26 09:11:25 2017): Hit 117

MAGNEQ02

Reference: Ran Shang, Sa Chen, Bing-Wu Wang, Zhe-Ming Wang, Song Gao (2016) *Angew.Chem.,Int.Ed.* ,**55,**2097

Formula: (C_3 H_3 Cu_1 O_6 $^{1-}$)n,C_2 H_8 N_1 $^{1+}$

Compound Name: catena-[ethylammonium bis(μ_2-formato)-(formato)-copper]

Space Group:	P212121	***Cell:***	***a***	10.144(0)	***b***	8.289(0)	***c***	11.160(0)
Space Group No.:	19	***(Å,°)***	α	90.00	β	90.00	γ	90.00

R-Factor (%): 2.86 ***Temperature(K):*** 290 ***Density(g/cm³):*** 1.732

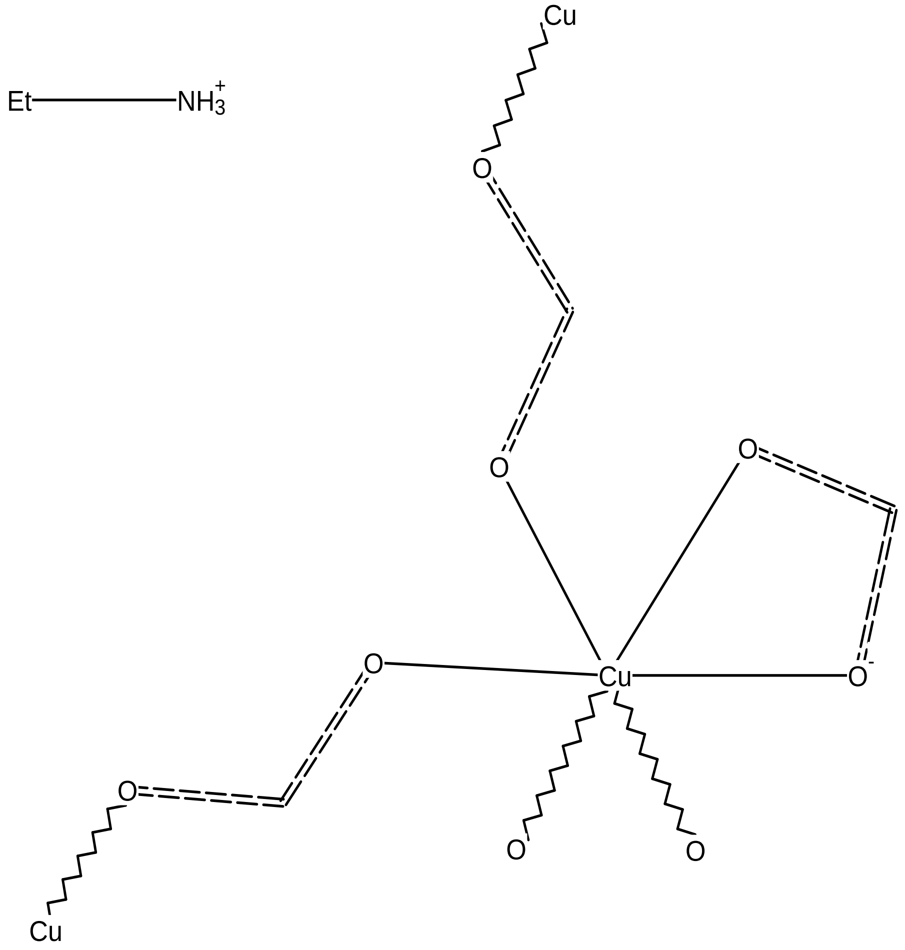

Parameters

Fragment 1

**DIST1 (D)**	1.954
**DIST2 (D)**	2.002
**DIST3 (D)**	1.956
**DIST4 (D)**	1.987
**DIST5 (D)**	2.732
**DIST6 (D)**	2.283

Search: search2 (Wed Jul 26 09:11:25 2017): Hit 118

MAGNEQ03

Reference: Ran Shang, Sa Chen, Bing-Wu Wang, Zhe-Ming Wang, Song Gao (2016) *Angew.Chem.,Int.Ed.* ,**55,**2097

Formula: $(C_3 H_3 Cu_1 O_6{}^{1-})n, C_2 H_8 N_1{}^{1+}$

Compound Name: catena-[ethylammonium bis(μ_2-formato)-(formato)-copper]

Space Group:	P212121	***Cell:***	***a***	10.165(0)	***b***	8.296(0)	***c***	11.181(0)
Space Group No.:	19	***(Å,°)***	α	90.00	β	90.00	γ	90.00

R-Factor (%): 2.90 ***Temperature(K):*** 320 ***Density(g/cm³):*** 1.724

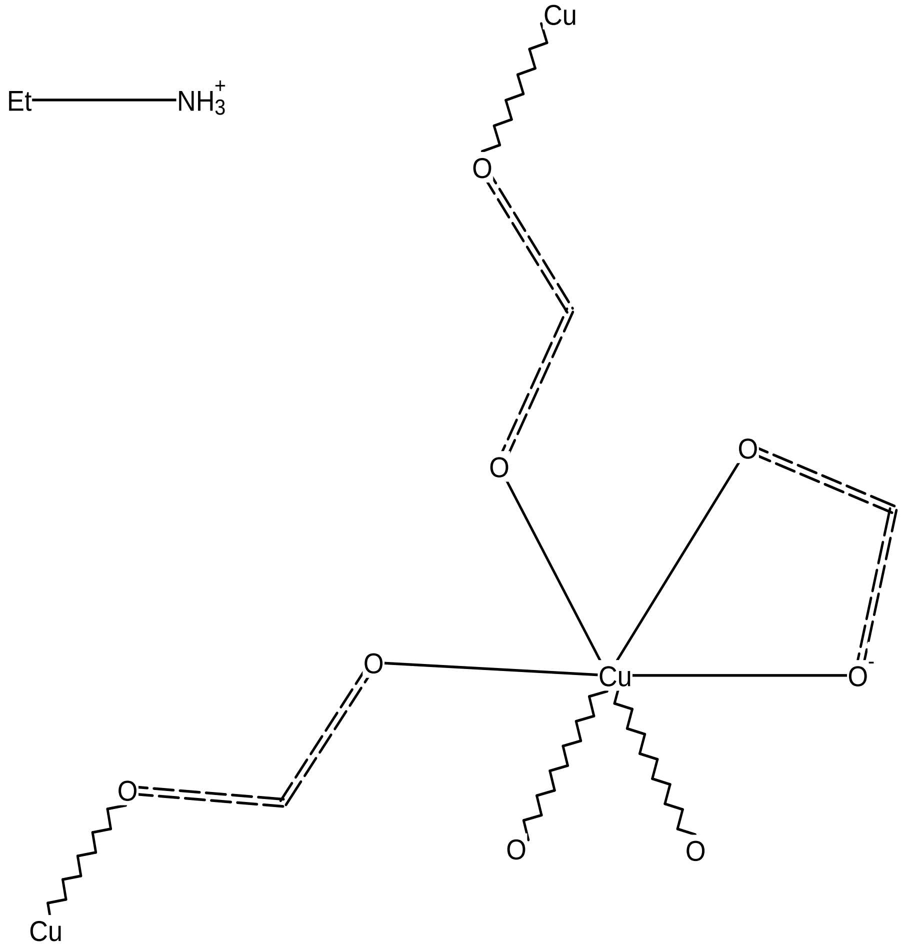

Parameters

Fragment 1

**DIST1 (D)**	1.954
**DIST2 (D)**	2.004
**DIST3 (D)**	1.957
**DIST4 (D)**	1.991
**DIST5 (D)**	2.738
**DIST6 (D)**	2.287

Search: search2 (Wed Jul 26 09:11:25 2017): Hit 119

MOHHIB

Reference: S.R.Choudhury, A.D.Jana, Chih-Yuan Chen, A.Dutta, E.Colacio, Hon Man Lee, G.Mostafa, S.Mukhopadhyay (2008) *CrystEngComm* ,**10,**1358

Formula: $(C_6 H_9 N_2{}^{1+})2n,n(C_6 H_4 Cu_1 O_8{}^{2-}),2n(H_2 O_1)$

Compound Name: catena-(bis(2-Amino-4-methylpyridinium) bis(μ_2-malonato)-copper(ii) dihydrate)

Space Group:	P21/c	***Cell:***	***a*** 15.491(0)	***b***	9.638(0)	***c***	7.451(0)
Space Group No.:	14	***(Å,°)***	α 90.00	β	102.59(0)	γ	90.00

R-Factor (%): 2.28 ***Temperature(K):*** 273 ***Density(g/cm³):*** 1.597

Parameters

Fragment 1

**DIST1 (D)**	1.922
**DIST2 (D)**	1.946
**DIST3 (D)**	1.946
**DIST4 (D)**	1.922
**DIST5 (D)**	2.614
**DIST6 (D)**	2.614

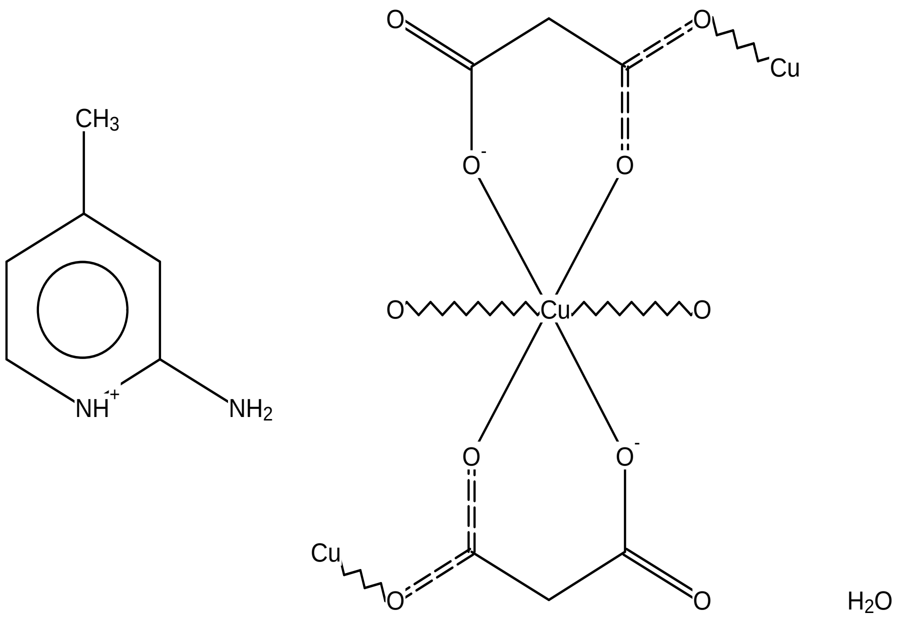

Search: search2 (Wed Jul 26 09:11:25 2017): Hit 120

MOLHIG

Reference: A.Hori, K.Nakajima, Y.Akimoto, K.Naganuma, H.Yuge (2014) *CrystEngComm* ,**16,**8805

Formula: C_{44} H_{22} Cu_1 F_{16} O_6

Compound Name: bis(1,3-dioxo-1,3-bis(2,3,5,6-tetrafluorophenyl)propan-2-ide)-bis(methoxybenzene)-copper(ii)

Space Group:	P-1	***Cell:***	***a*** 7.728(2)	***b*** 11.089(3)	***c*** 12.681(4)
Space Group No.:	2	***(Å,°)***	α 106.69(0)	β 104.54(0)	γ 92.43(0)

R-Factor (%): 2.69 ***Temperature(K):*** 100 ***Density(g/cm^3):*** 1.684

Parameters

Fragment 1

**DIST1 (D)**	1.925
**DIST2 (D)**	1.923
**DIST3 (D)**	1.925
**DIST4 (D)**	1.923
**DIST5 (D)**	2.614
**DIST6 (D)**	2.614

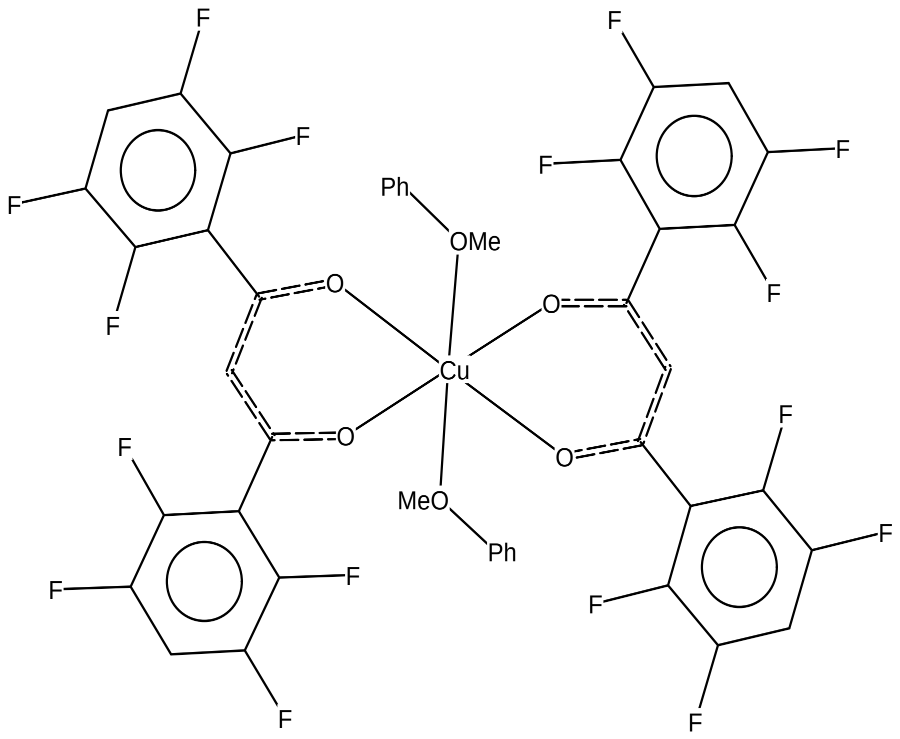

Search: search2 (Wed Jul 26 09:11:25 2017): Hit 121

MOLJUU

Reference: A.Hori, K.Nakajima, Y.Akimoto, K.Naganuma, H.Yuge (2014) *CrystEngComm* ,**16,**8805

Formula: C_{44} H_{18} Cu_{1} F_{20} O_{6}

Compound Name: bis(anisole)-bis(1,3-bis(pentafluorophenyl)propane-1,3-dionato)-copper(ii)

Space Group:	P-1	***Cell:***	***a*** 7.702(1)	***b***	11.163(3)	***c***	11.884(3)
Space Group No.:	2	***(Å,°)***	α 106.86(0)	β	92.79(0)	γ	94.26(0)

R-Factor (%): 2.71 ***Temperature(K):*** 100 ***Density(g/cm^3):*** 1.855

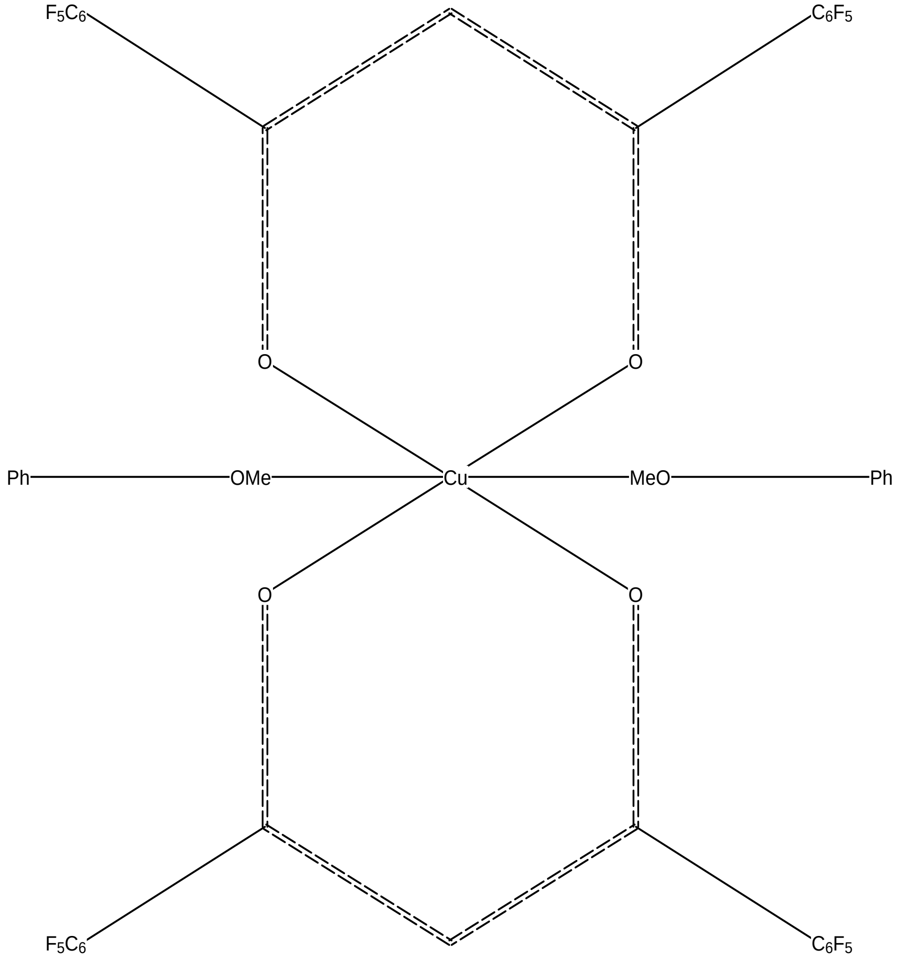

Parameters

Fragment 1

**DIST1 (D)**	1.923
**DIST2 (D)**	1.945
**DIST3 (D)**	1.923
**DIST4 (D)**	1.945
**DIST5 (D)**	2.577
**DIST6 (D)**	2.577

Search: search2 (Wed Jul 26 09:11:25 2017): Hit 122

MUFDAU

Reference:	N.V.Somov, F.F.Chausov (2015)<br>*Kristallografiya(Russ.)(Crystallogr.Rep.)* ,**60,**233
Formula:	$(C_3\ H_{16}\ Cu_1\ N_1\ O_{12}\ P_3)n$
Compound Name:	catena-[(μ-phosphono-N-((phosphono)methyl)-N-((phosphono)methyl) methanaminiumato)-triaqua-copper]

Space Group:	P21/c	***Cell:***	***a*** 9.251(0)	***b*** 15.982(0)	***c*** 9.547(0)		
Space Group No.:	14	***(Å,°)***	α 90.00	β 113.70(0)	γ 90.00		
R-Factor (%):	1.97	***Temperature(K):***	293	***Density(g/cm³):***	2.131		

Parameters

Fragment 1

**DIST1 (D)**	2.018
**DIST2 (D)**	2.049
**DIST3 (D)**	1.959
**DIST4 (D)**	1.948
**DIST5 (D)**	2.309
**DIST6 (D)**	2.297

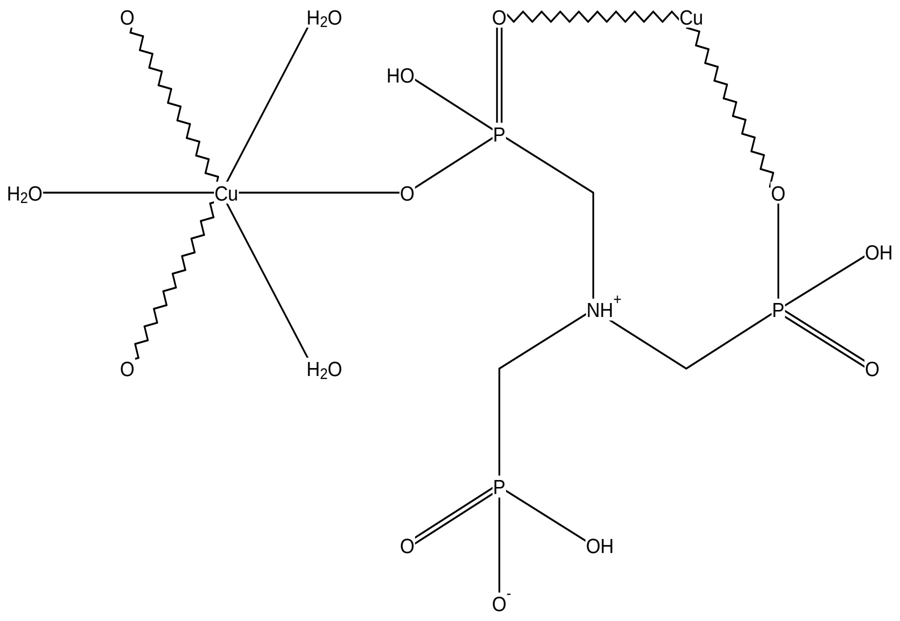

Search: search2 (Wed Jul 26 09:11:25 2017): Hit 123

NACSEP

Reference:	Jinkwon Kim, Jin Mook Lim, Yong-Kook Choi, Youngkyu Do (1996) *Angew.Chem.,Int.Ed.* ,**35,**998
Formula:	$(C_{14}\,H_{24}\,Cu_1\,Mn_1\,O_{12}\,S_4)n$
Compound Name:	catena-((bis(μ_3-bis(Methylthio)methylene)malonato)-dimethanol-copper(ii)-diaqua-manganese(ii))

Space Group:	P-1	***Cell:***	***a*** 7.278(0)	***b*** 7.338(2)	***c*** 11.392(0)		
Space Group No.:	2	***(Å,°)***	α 102.70(1)	β 91.17(1)	γ 93.37(1)		

R-Factor (%): 2.14 ***Temperature(K):*** 295 ***Density(g/cm^3):*** 1.770

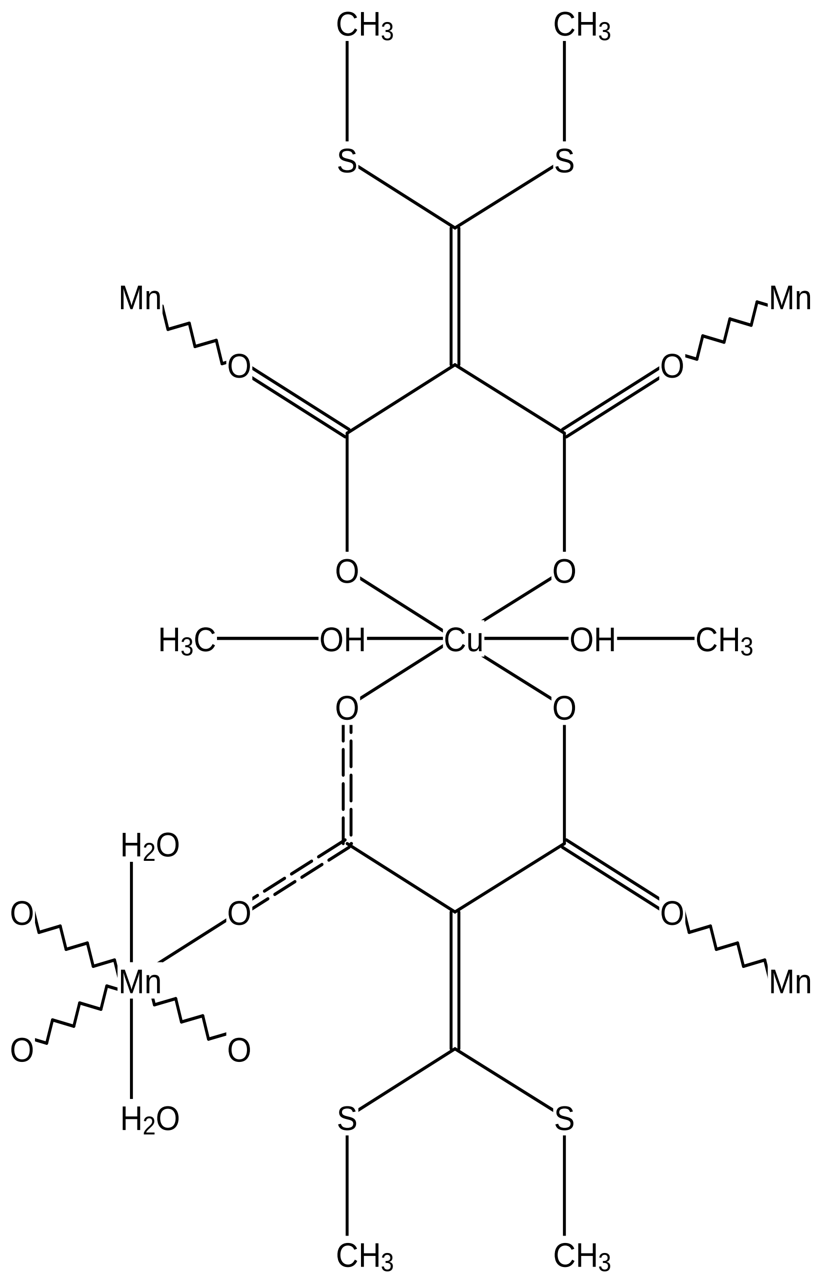

Parameters

Fragment 1

**DIST1 (D)**	1.971
**DIST2 (D)**	1.971
**DIST3 (D)**	1.949
**DIST4 (D)**	1.949
**DIST5 (D)**	2.482
**DIST6 (D)**	2.482

Search: search2 (Wed Jul 26 09:11:25 2017): Hit 124

NAFXAU

Reference:	Shan Gao, Yu-Mei Yue, Dong-Sheng Ma, Jin-Sheng Gao, Peng-Fei Yan (2004) *Jiegou Huaxue(Chin.)(Chin.J.Struct.Chem.)* ,**23,** 825
Formula:	C_{18} H_{18} Cu_{1} O_{12}
Compound Name:	diaqua-bis(4-carboxyphenoxyacetato)-copper(ii)

Space Group:	C2/c	***Cell:***	***a***	12.298(3)	***b***	7.380(1)	***c***	21.413(4)
Space Group No.:	15	***(Å,°)***	α	90.00	β	94.00(3)	γ	90.00

R-Factor (%):	2.79	***Temperature(K):***	293	***Density(g/cm^3):***	1.678

Parameters

Fragment 1

**DIST1 (D)**	1.920
**DIST2 (D)**	1.941
**DIST3 (D)**	1.941
**DIST4 (D)**	1.955
**DIST5 (D)**	2.382
**DIST6 (D)**	2.382

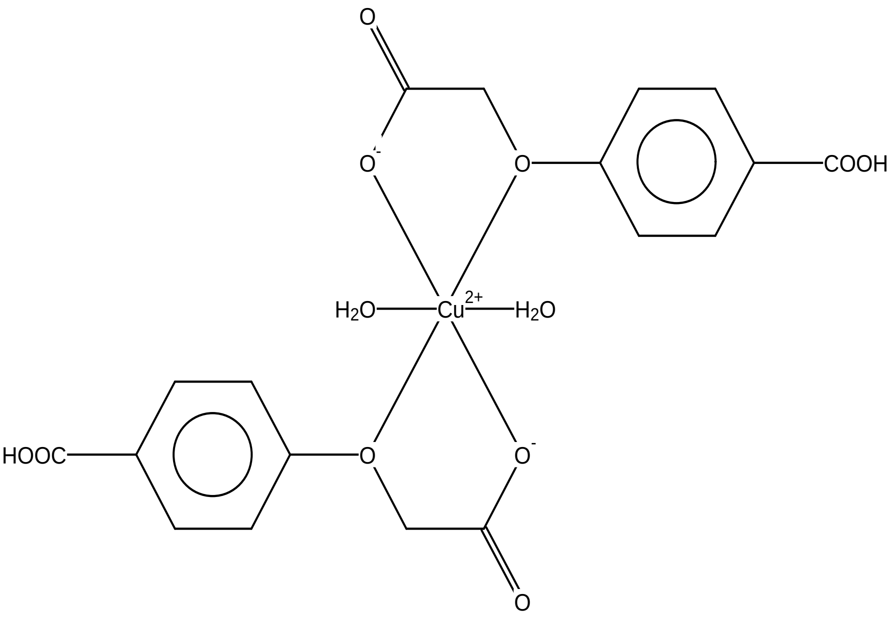

Search: search2 (Wed Jul 26 09:11:25 2017): Hit 125

NAPCIR

Reference:	E.V.Dikarev, Haitao Zhang, Bo Li (2005) *J.Am.Chem.Soc.* , **127,**6156
Formula:	C_{40} H_8 Bi_2 Cu_1 F_{48} O_{16}
Compound Name:	bis(μ_2-1,1,1,5,5,5-Hexafluoroacetylacetonato)-hexakis(1,1,1,5,5,5-hexafluoro-acetylacetonato)-di-bismuth(iii)-copper(ii)

Space Group:	P-1	***Cell:***	***a*** 8.696(0)	***b***	11.243(0)	***c***	16.877(0)
Space Group No.:	2	***(Å,°)***	α 73.72(0)	β	76.46(0)	γ	69.63(0)
R-Factor (%):	2.10	***Temperature(K):***	90	***Density(g/cm^3):***	2.419		

Parameters

Fragment 1

**DIST1 (D)**	1.928
**DIST2 (D)**	1.928
**DIST3 (D)**	1.926
**DIST4 (D)**	1.926
**DIST5 (D)**	2.588
**DIST6 (D)**	2.588

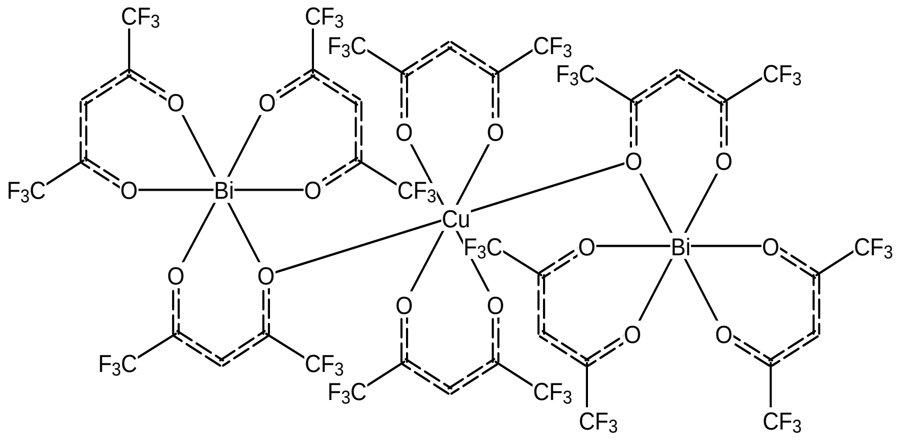

Search: search2 (Wed Jul 26 09:11:25 2017): Hit 126

NEDPAO

Reference:	B.Kozlevcar, M.Humar, P.Strauch, I.Leban (2005)<br>*Z.Naturforsch.,B:Chem.Sci.* ,**60,**1273
Formula:	C_{16} H_{18} Cu_1 O_8
Compound Name:	cis-Diaqua-bis(vanillinato)-copper(ii)
Synonym:	cis-Diaqua-bis(4-formyl-2-methoxyphenolato)-copper(ii)

Space Group:	C2/c	***Cell:***	***a*** 22.042(0)	***b*** 10.689(0)	***c*** 7.667(0)		
Space Group No.:	15	***(Å,°)***	α 90.00	β 104.85(0)	γ 90.00		

R-Factor (%): 2.56 ***Temperature(K):*** 293 ***Density(g/cm^3):*** 1.529

Parameters

Fragment 1

**DIST1 (D)**	2.087
**DIST2 (D)**	1.909
**DIST3 (D)**	1.909
**DIST4 (D)**	2.087
**DIST5 (D)**	2.260
**DIST6 (D)**	2.260

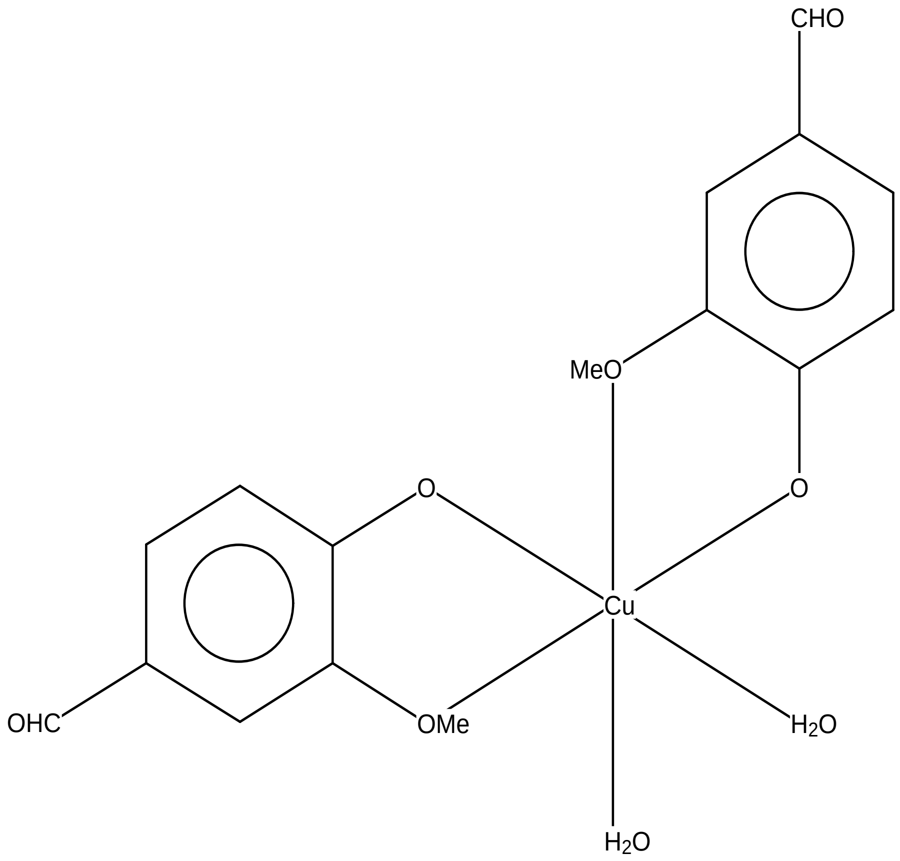

Search: search2 (Wed Jul 26 09:11:25 2017): Hit 127

NEDPAO01

Reference:	B.Kozlevcar, A.Golobic, P.Strauch (2006) *Polyhedron* ,**25,** 2824
Formula:	C_{16} H_{18} Cu_1 O_8
Compound Name:	cis-diaqua-bis(4-formyl-2-methoxyphenolato-O,O')-copper(ii)
Synonym:	cis-diaqua-bis(vanillinato)-copper(ii)

Space Group:	P21/c	***Cell:***	***a***	7.740(0)		***b***	10.492(0)		***c***	21.240(0)	
Space Group No.:	14	***(Å,°)***	α	90.00		β	95.63(0)		γ	90.00	

R-Factor (%):	2.60	***Temperature(K):***	115	***Density(g/cm³):***	1.555

Parameters

Fragment 1

**DIST1 (D)**	1.919
**DIST2 (D)**	1.914
**DIST3 (D)**	2.151
**DIST4 (D)**	2.020
**DIST5 (D)**	2.354
**DIST6 (D)**	2.163

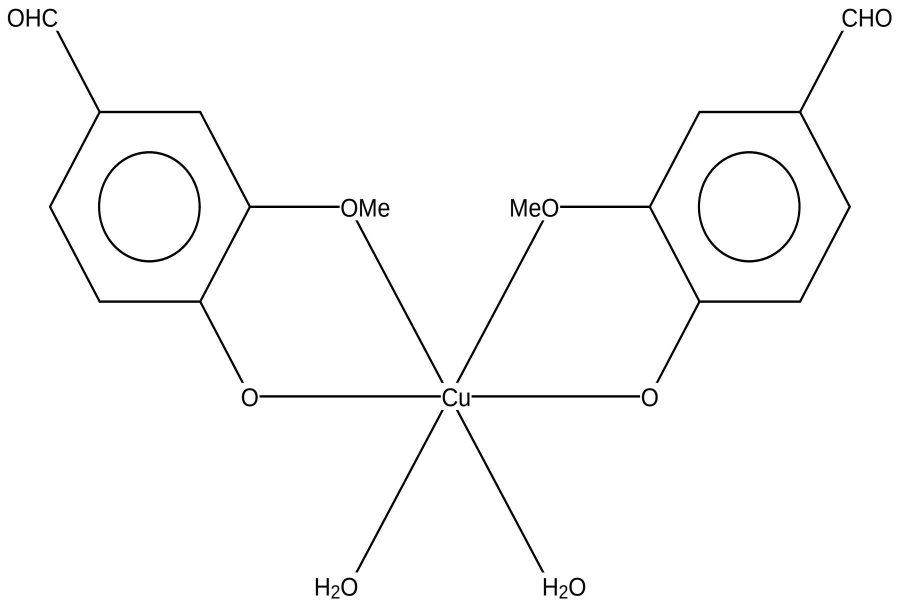

Search: search2 (Wed Jul 26 09:11:25 2017): Hit 128

NENBEO

Reference:	F.S.Delgado, C.Ruiz-Perez, J.Sanchiz, F.Lloret, M.Julve (2006) *CrystEngComm* ,**8,**530
Formula:	$(C_1\ H_6\ N_1\ ^{1+})2n,n(C_6\ H_4\ Cu_1\ O_8\ ^{2-})$
Compound Name:	catena-(bis(methylammonium) bis(μ_2-malonato)-copper(ii))

Space Group:	P21/c	***Cell:***	***a*** 8.687(0)	***b*** 9.059(0)	***c*** 8.968(0)		
Space Group No.:	14	***(Å,°)***	α 90.00	β 115.20(0)	γ 90.00		
R-Factor (%):	2.28	***Temperature(K):***	293	***Density(g/cm³):***	1.726		

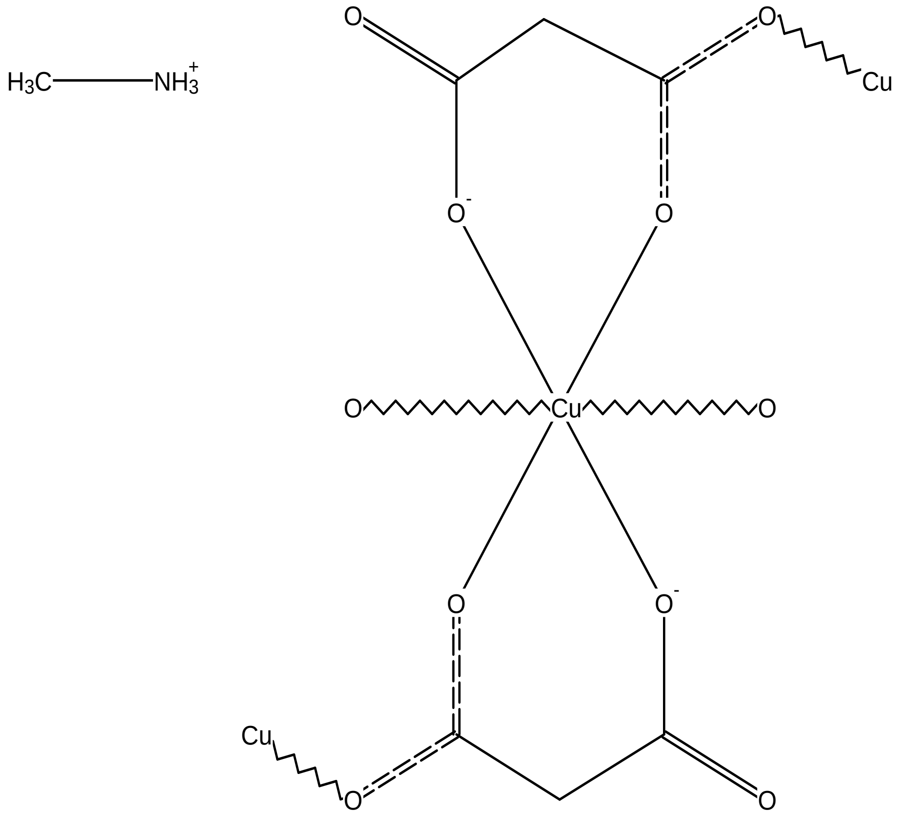

Parameters

Fragment 1

**DIST1 (D)**	1.961
**DIST2 (D)**	1.924
**DIST3 (D)**	1.924
**DIST4 (D)**	1.961
**DIST5 (D)**	2.561
**DIST6 (D)**	2.561

Search: search2 (Wed Jul 26 09:11:25 2017): Hit 129

NERKUQ

Reference: Bao-Qing Ma, Hao-Ling Sun, Song Gao, Guang-Xian Xu
(2001) *Inorg.Chem.* ,**40,**6247

Formula: $2(C_{10} H_8 N_2 O_2),H_{12} Cu_1 O_6{}^{2+},2(Cl_1{}^{1-}),2(H_2 O_1)$

Compound Name: Hexa-aqua-copper(ii) dichloride (4,4'-bipyridine-N,N'-dioxide) solvate dihydrate

Space Group:	P-1	***Cell:***	***a***	6.732(0)	***b***	10.254(1)	***c***	10.423(4)
Space Group No.:	2	***(Å,°)***	α	85.09(2)	β	72.81(1)	γ	89.23(1)

R-Factor (%): 2.88 ***Temperature(K):*** 293 ***Density(g/cm^3):*** 1.588

Parameters

Fragment 1

**DIST1 (D)**	1.968
**DIST2 (D)**	1.968
**DIST3 (D)**	1.968
**DIST4 (D)**	1.968
**DIST5 (D)**	2.376
**DIST6 (D)**	2.376

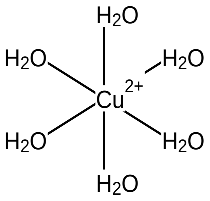

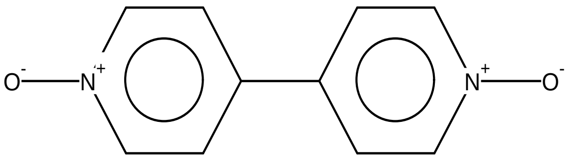

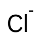

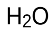

Search: search2 (Wed Jul 26 09:11:25 2017): Hit 130

NERWAK

Reference:	A.V.Pestov, P.A.Slepukhin, Yu.G.Yatluk (2012)<br>*Koord.Khim.(Russ.)(Coord.Chem.)* ,**38,**874
Formula:	$C_8\ H_{18}\ Cu_1\ O_{10}$
Compound Name:	Diaqua-bis((2-hydroxyethoxy)acetato-O,O')-copper(ii)

Space Group:	P-1	***Cell:***	***a***	6.957(0)	***b***	7.277(0)	***c***	7.309(0)
Space Group No.:	2	***(Å,°)***	α	75.38(0)	β	64.18(1)	γ	75.09(0)

R-Factor (%):	2.35	***Temperature(K):***	295	***Density(g/cm^3):***	1.766

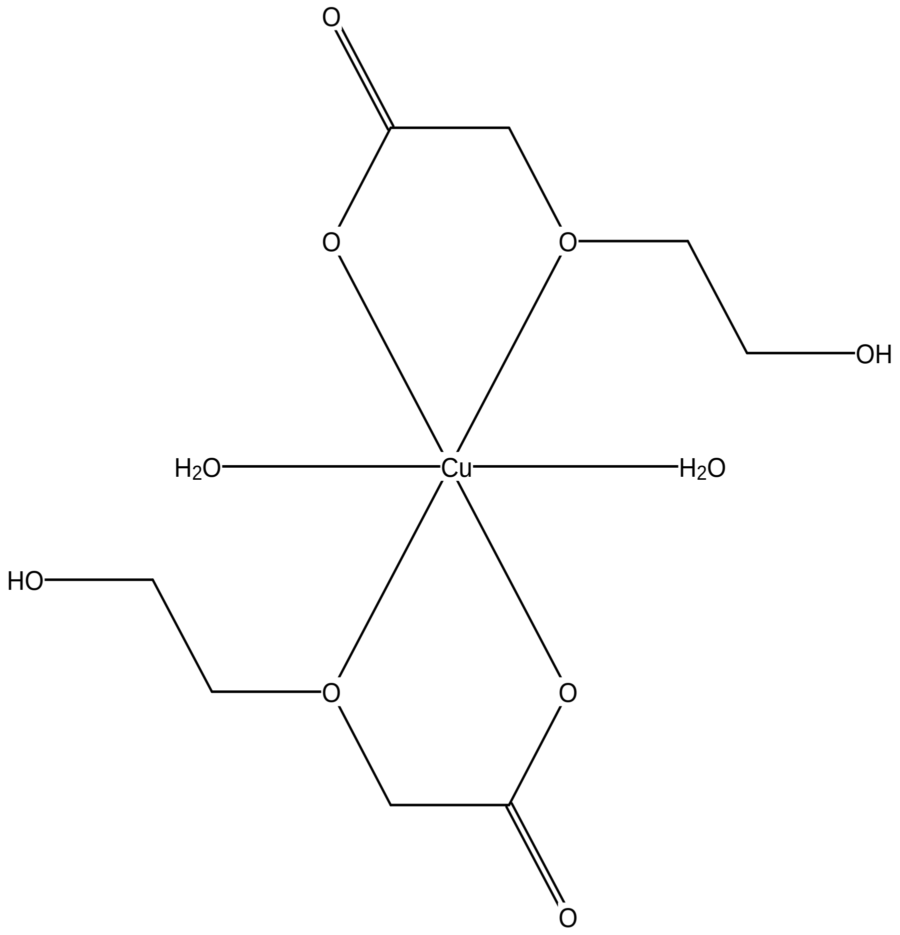

Parameters

Fragment 1

**DIST1 (D)**	1.993
**DIST2 (D)**	1.952
**DIST3 (D)**	1.952
**DIST4 (D)**	1.993
**DIST5 (D)**	2.375
**DIST6 (D)**	2.375

Search: search2 (Wed Jul 26 09:11:25 2017): Hit 131

NOMGAZ

Reference: Gui-lin Zhuang, Li Tan, Wu-lin Chen, Jun Zheng, Hong-zhou Yao, Xing Zhong, Jian-guo Wang (2014) *Inorg.Chem.Front.* ,**1,**526

Formula: $(C_8 H_{12} Cu_1 N_2 O_8)n$

Compound Name: catena-[(μ-2,2'-(2,5-dioxopiperazine-1,4-diyl)diacetato)-diaqua-copper]

Space Group:	C2/c	***Cell:***	***a*** 20.548(0)	***b***	4.657(0)	***c***	13.977(0)
Space Group No.:	15	***(Å,°)***	α 90.00	β	124.40(0)	γ	90.00

R-Factor (%): 2.25 ***Temperature(K):*** 301 ***Density(g/cm³):*** 1.972

Parameters

Fragment 1

**DIST1 (D)**	1.957
**DIST2 (D)**	1.957
**DIST3 (D)**	1.950
**DIST4 (D)**	1.950
**DIST5 (D)**	2.615
**DIST6 (D)**	2.615

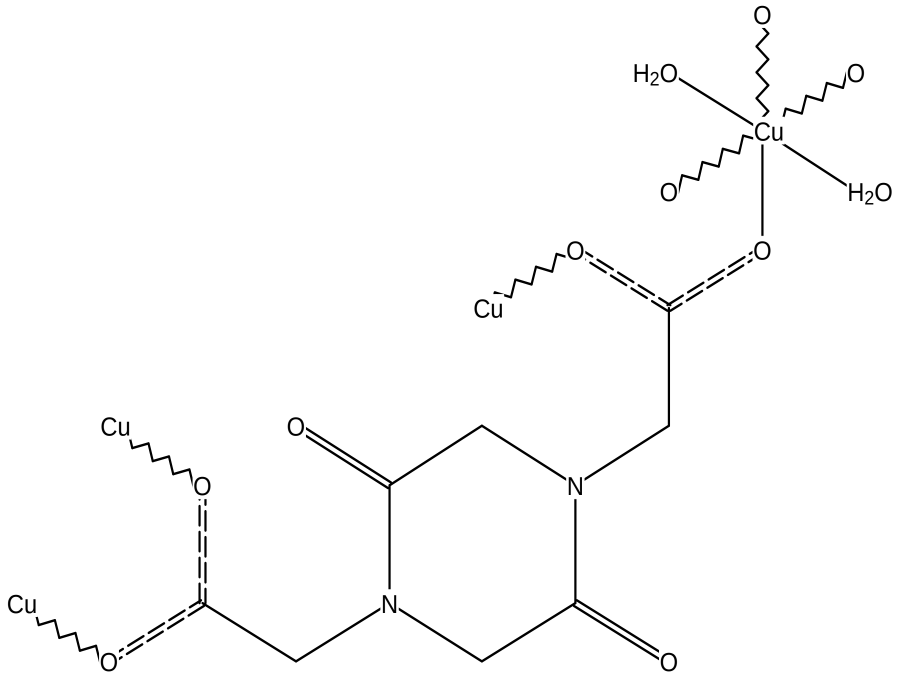

Search: search2 (Wed Jul 26 09:11:25 2017): Hit 132

OFEJIU

Reference: Yan-Yan Jia, Bin Liu, Xue-Mei Liu, Jian-Hui Yang (2013) *CrystEngComm* ,**15,**7936

Formula: $(C_4 H_4 Br_2 Cu_1 K_2 Li_1 O_{19} Ru_2)n$

Compound Name: catena-[tetrakis(μ_4-carbonato)-bis(μ_4-bromo)-bis(μ_2-aqua)-pentakis(μ_2-oxo)-copper-di-potassium-lithium-di-ruthenium]

Space Group:	Pccn	***Cell:***	***a***	10.976(0)	***b***	11.284(1)	***c***	17.248(1)
Space Group No.:	56	***(Å,°)***	α	90.00	β	90.00	γ	90.00

R-Factor (%): 2.41 ***Temperature(K):*** 296 ***Density(g/cm^3):*** 2.695

Parameters

Fragment 1

**DIST1 (D)**	2.009
**DIST2 (D)**	2.009
**DIST3 (D)**	2.059
**DIST4 (D)**	2.059
**DIST5 (D)**	2.312
**DIST6 (D)**	2.312

$n\text{-}Ru_2CuK_2Li[\mu_4\text{-}CO_3\text{-}\kappa^4O,O',O'']_4[\mu_4\text{-}Br]_2\text{--}$
$\text{--}[\mu_2\text{-}H_2O]_2[\mu_2\text{-}O]_5$

Search: search2 (Wed Jul 26 09:11:25 2017): Hit 133

OJUXUO

Reference: Junqing Zhang, Cun Li, Juanjuan Wang, Mei Zhu, Licun Li
(2016) *Eur.J.Inorg.Chem.* ,1383

Formula: C_{46} H_{20} Cu_1 Dy_2 F_{36} O_{18}

Compound Name: bis(μ-1,1,1,5,5,5-hexafluoroacetylacetonato)-bis(μ-2-oxy-4-methoxybenzaldehyde)-tetrakis(1,1,1,5,5,5-hexafluoroacetylacetonato)-copper-di-dysprosium

Space Group:	P-1	***Cell:***	***a*** 9.883(2)	***b*** 11.104(2)	***c*** 15.995(3)
Space Group No.:	2	***(Å,°)***	α 100.51(3)	β 99.61(3)	γ 113.18(3)

R-Factor (%): 2.65 ***Temperature(K):*** 113 ***Density(g/cm³):*** 2.098

Parameters

Fragment 1

**DIST1 (D)**	1.905
**DIST2 (D)**	1.949
**DIST3 (D)**	1.905
**DIST4 (D)**	1.949
**DIST5 (D)**	2.494
**DIST6 (D)**	2.494

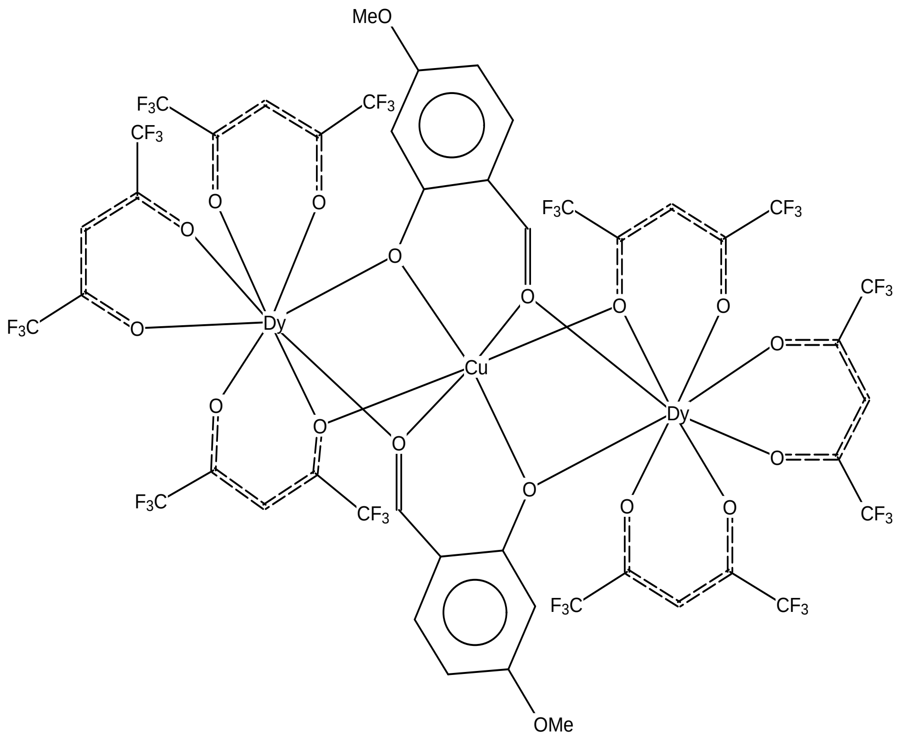

Search: search2 (Wed Jul 26 09:11:25 2017): Hit 134

ORIWES

Reference:	A.Fonari, E.S.Leonova, M.Yu.Antipin (2011) *Polyhedron* , **30,**1710
Formula:	C_{18} H_{22} Cu_1 N_4 O_{16} S_2
Compound Name:	diaqua-bis(dimethyl sulfoxide)-bis(3,5-dinitrobenzoato-O)-copper(ii)

Space Group:	P21/n	***Cell:***	***a*** 10.624(1)	***b*** 5.282(0)	***c*** 22.573(3)
Space Group No.:	14	***(Å,°)***	α 90.00	β 91.93(0)	γ 90.00
R-Factor (%):	2.52	***Temperature(K):***	100	***Density(g/cm³):***	1.779

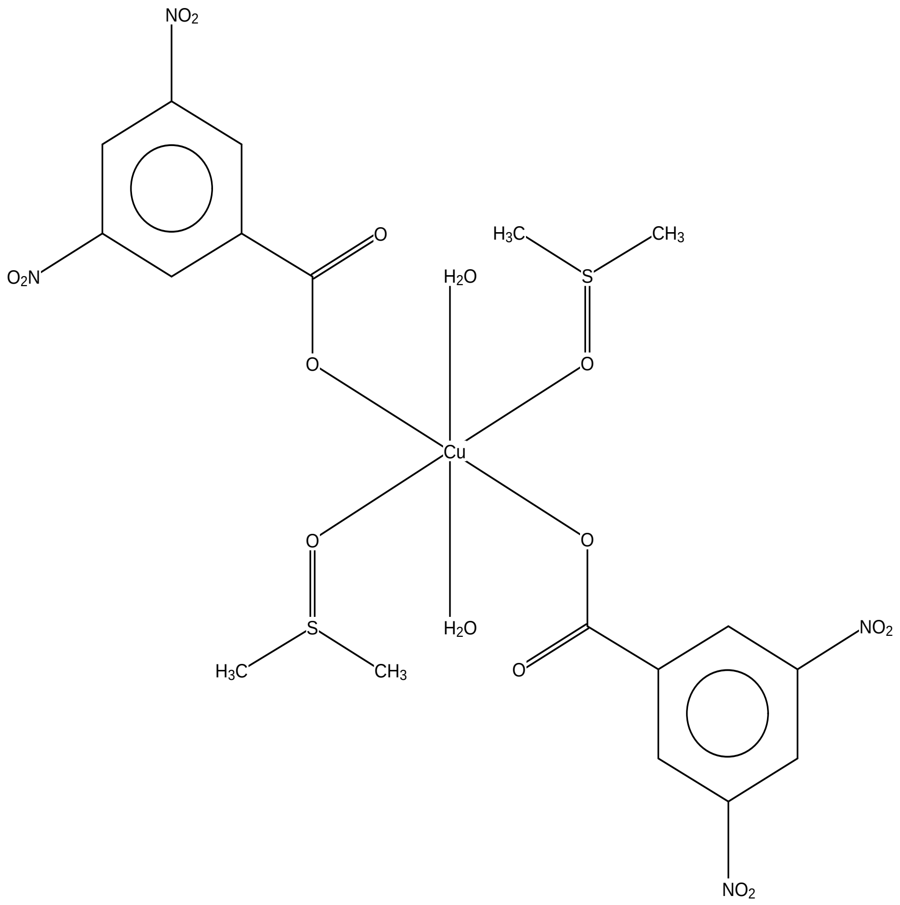

Parameters

Fragment 1

**DIST1 (D)**	1.971
**DIST2 (D)**	1.968
**DIST3 (D)**	1.968
**DIST4 (D)**	1.971
**DIST5 (D)**	2.371
**DIST6 (D)**	2.371

Search: search2 (Wed Jul 26 09:11:25 2017): Hit 135

PAGKAK

Reference:	V.T.Yilmaz, E.Senel, C.Kazak (2004)<br>*Solid State Sciences* ,**6,**859
Formula:	C_{10} H_{20} Cu_1 N_2 O_{10}
Compound Name:	trans-bis(2-Ammonioethanol)-bis(malonato)-copper(ii)

Space Group:	P21/n	***Cell:***	***a*** 8.663(0)	***b*** 7.512(0)	***c*** 11.439(1)		
Space Group No.:	14	***(Å,°)***	α 90.00	β 95.53(0)	γ 90.00		
R-Factor (%):	2.80	***Temperature(K):***	293	***Density(g/cm³):***	1.756		

Parameters

Fragment 1

**DIST1 (D)**	1.955
**DIST2 (D)**	1.933
**DIST3 (D)**	1.955
**DIST4 (D)**	1.933
**DIST5 (D)**	2.465
**DIST6 (D)**	2.465

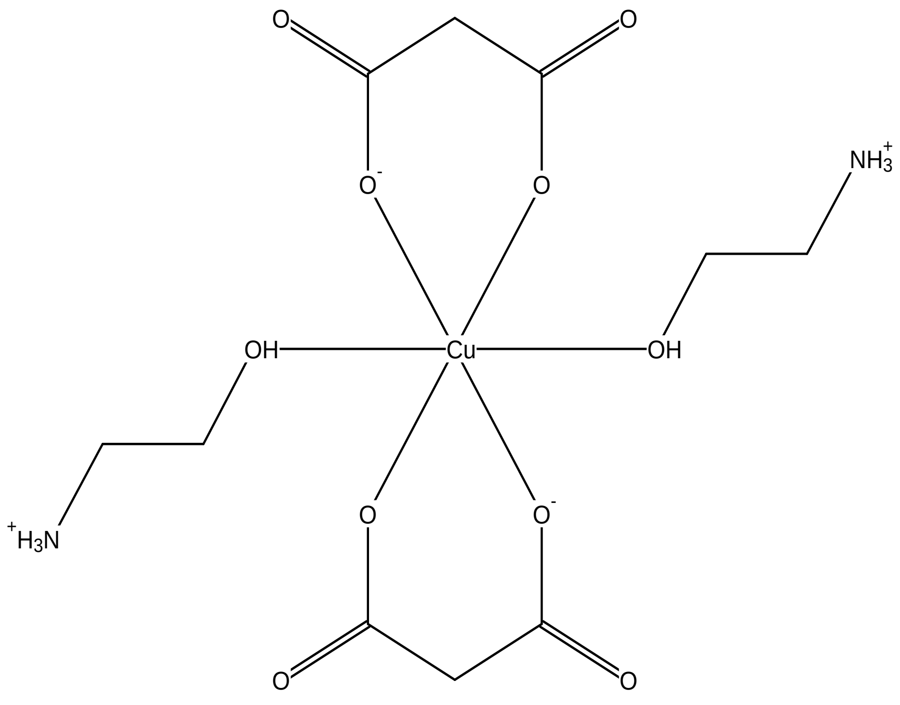

Search: search2 (Wed Jul 26 09:11:25 2017): Hit 136

PAKLAO

Reference:	A.Bouayad, C.Brouca-Cabarrecq, J.-C.Trombe, A.Gleizes (1992) *Inorg.Chim.Acta* ,**195,**193
Formula:	$(C_{16}\ H_{32}\ Cu_1\ La_2\ O_{32})n,2n(H_2\ O_1)$
Compound Name:	catena-(tetrakis(μ_2-Squarato-O,O')-hexadeca-aqua-di-lanthanum(iii)-copper(ii) dihydrate)

Space Group:	P21/c	***Cell:***	***a***	6.780(2)	***b***	32.311(4)	***c***	8.162(2)
Space Group No.:	14	***(Å,°)***	α	90.00	β	111.55(2)	γ	90.00

R-Factor (%):	1.80	***Temperature(K):***	295	***Density(g/cm³):***	2.224

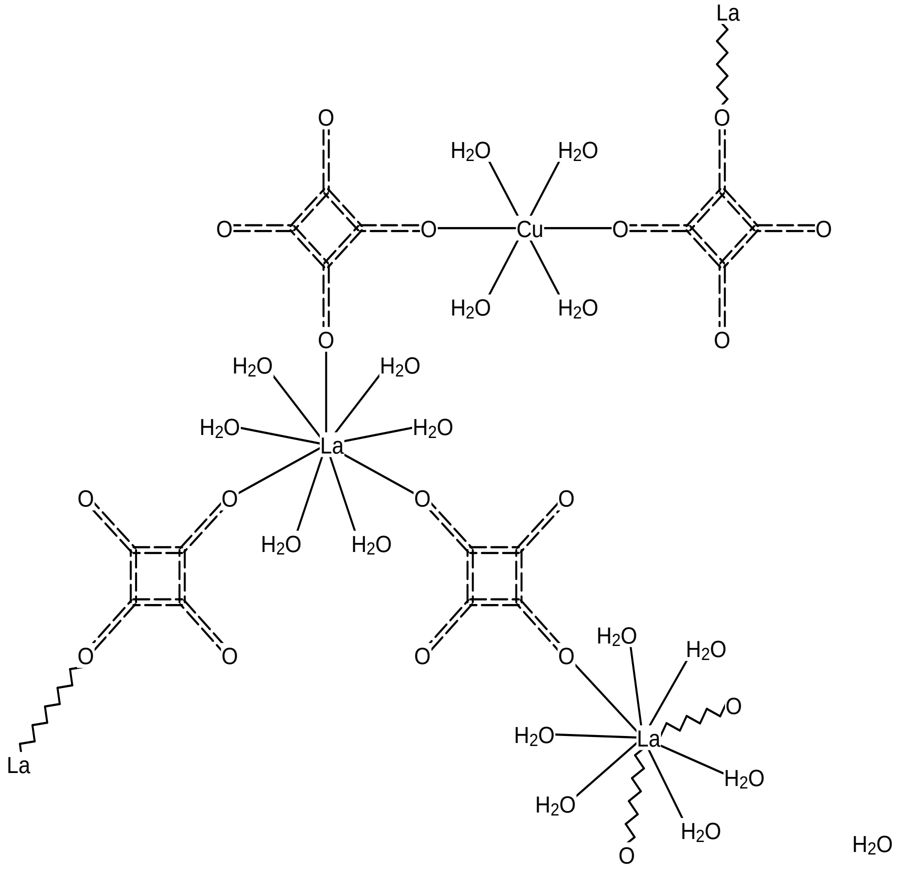

Parameters

Fragment 1

**DIST1 (D)**	1.977
**DIST2 (D)**	1.963
**DIST3 (D)**	1.963
**DIST4 (D)**	1.977
**DIST5 (D)**	2.426
**DIST6 (D)**	2.426

Search: search2 (Wed Jul 26 09:11:25 2017): Hit 137

PAKLES

Reference: A.Bouayad, C.Brouca-Cabarrecq, J.-C.Trombe, A.Gleizes (1992) *Inorg.Chim.Acta* ,**195,**193

Formula: $(C_{16} H_{24} Cu_1 Gd_2 O_{28})n,2n(H_2 O_1)$

Compound Name: catena-(bis(μ_3-Squarato-O,O',O'')-bis(μ_2-squarato-O,O')-dodeca-aqua-di-gadolinium(iii)-copper(ii) dihydrate)

Space Group:	C2/c	***Cell:***	***a***	13.784(1)	***b***	8.219(1)	***c***	26.268(2)
Space Group No.:	15	***(Å,°)***	α	90.00	β	94.20(7)	γ	90.00

R-Factor (%): 2.40 ***Temperature(K):*** 295 ***Density(g/cm^3):*** 2.413

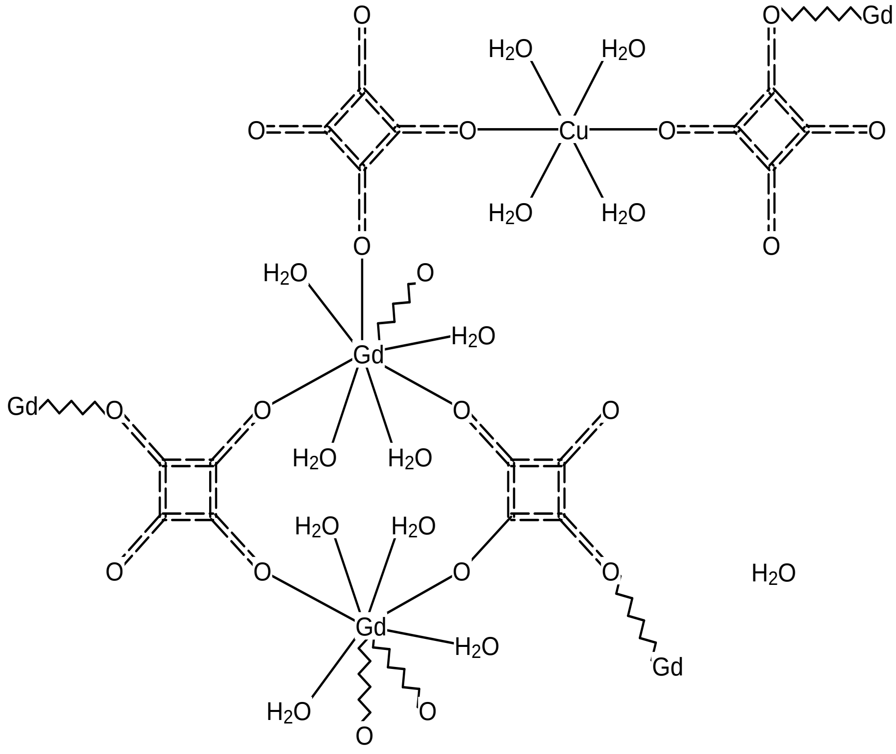

Parameters

Fragment 1

**DIST1 (D)**	1.965
**DIST2 (D)**	1.962
**DIST3 (D)**	1.962
**DIST4 (D)**	1.965
**DIST5 (D)**	2.377
**DIST6 (D)**	2.377

Search: search2 (Wed Jul 26 09:11:25 2017): Hit 138

PAMSEB

Reference:	H.Paulus, H.Fuess, P.Baran, D.Valigura (1992) *Z.Kristallogr.* ,**202,**140
Formula:	C_{10} H_{10} Cu_1 N_4 O_9
Compound Name:	Aqua-(2,2'-bipyridine-N,N'-dioxide-O,O')-(nitrato-O,O')-(nitrato-O)-copper(ii)

Space Group:	P21/c	***Cell:***	***a***	6.635(2)	***b***	7.188(2)	***c***	29.038(3)
Space Group No.:	14	***(Å,°)***	α	90.00	β	95.54(1)	γ	90.00

R-Factor (%):	2.60	***Temperature(K):***	295	***Density(g/cm³):***	1.897

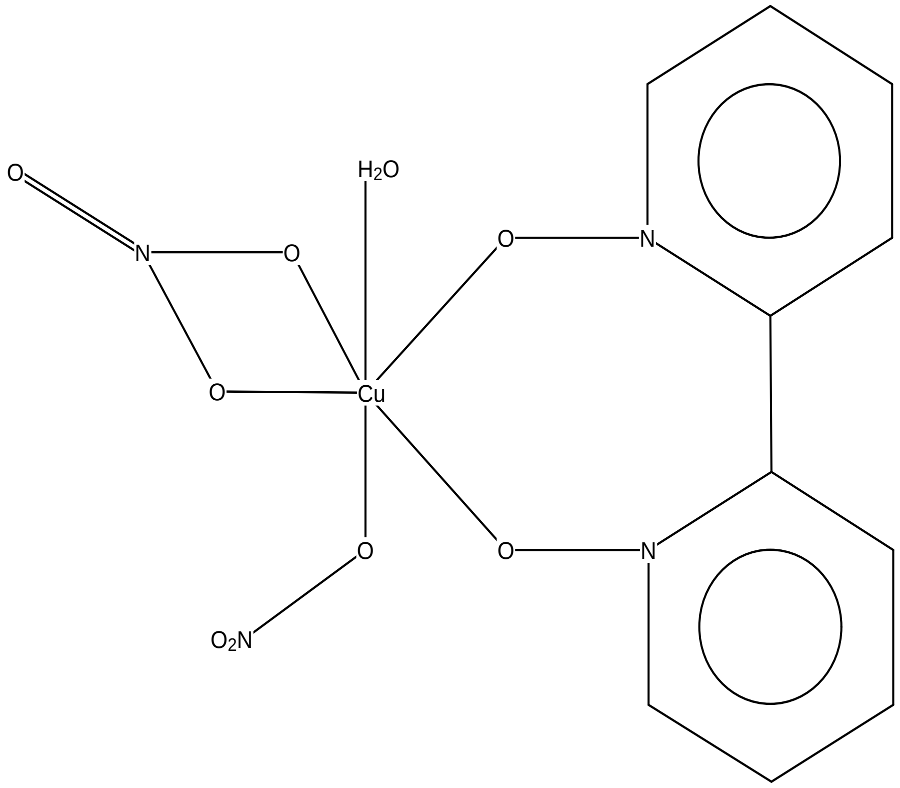

Parameters

Fragment 1

**DIST1 (D)**	1.990
**DIST2 (D)**	1.947
**DIST3 (D)**	1.944
**DIST4 (D)**	1.948
**DIST5 (D)**	2.529
**DIST6 (D)**	2.371

Search: search2 (Wed Jul 26 09:11:25 2017): Hit 139

PASNEE

Reference: Feifei Xing, Yueling Bai, Xiang He, Juan Jia, Dong Zhou, Min Shao, Shourong Zhu (2012) *CrystEngComm* ,**14,**4312

Formula: $(C_{24} H_{34} Cu_5 O_{34})n,4n(H_2 O_1)$

Compound Name: catena(-bis(μ_5-6-Carboxyl-benzene-1,2,3,4,5-pentacarboxylato)-deca-aqua-penta-copper tetrahydrate)

Space Group:	C2/c	***Cell:***	***a*** 17.098(1)	***b***	8.386(0)	***c***	27.613(3)
Space Group No.:	15	***(Å,°)***	α 90.00	β	104.65(0)	γ	90.00

R-Factor (%): 2.54 ***Temperature(K):*** 296 ***Density(g/cm³):*** 2.178

Parameters

Fragment 1

**DIST1 (D)**	1.956
**DIST2 (D)**	1.945
**DIST3 (D)**	1.945
**DIST4 (D)**	1.937
**DIST5 (D)**	2.404
**DIST6 (D)**	2.404

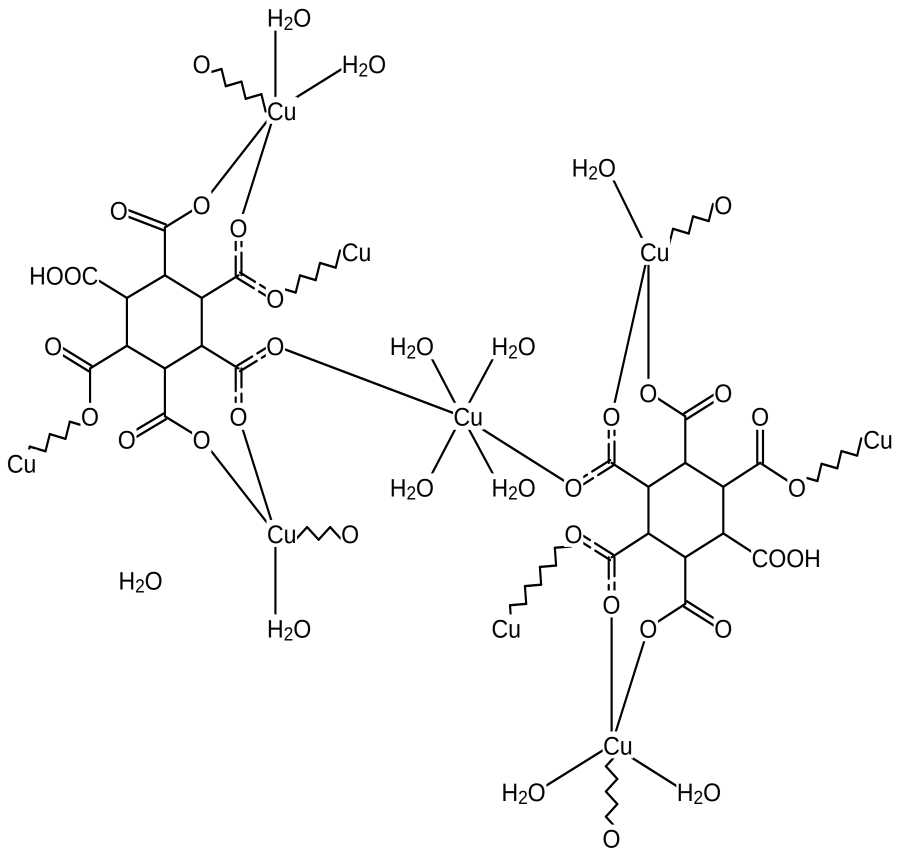

Search: search2 (Wed Jul 26 09:11:25 2017): Hit 140

PASZAL

Reference:	Li Wang, Xiao-Lan Yu, Jiwen Cai, Jin-Wang Huang (2005)<br>*J.Chem.Cryst.* ,**35,**481
Formula:	$(C_{10}\ H_{14}\ Cu_1\ O_{10}\ S_2)n$
Compound Name:	catena-((μ_2-1,5-Naphthalenedisulfonato-O,O')-tetra-aqua-copper(ii))

Space Group:	Pbca	***Cell:***	***a*** 12.956(1)	***b*** 9.885(1)	***c*** 22.797(3)		
Space Group No.:	61	***(Å,°)***	α 90.00	β 90.00	γ 90.00		
R-Factor (%):	2.57	***Temperature(K):***	293	***Density(g/cm^3):***	1.920		

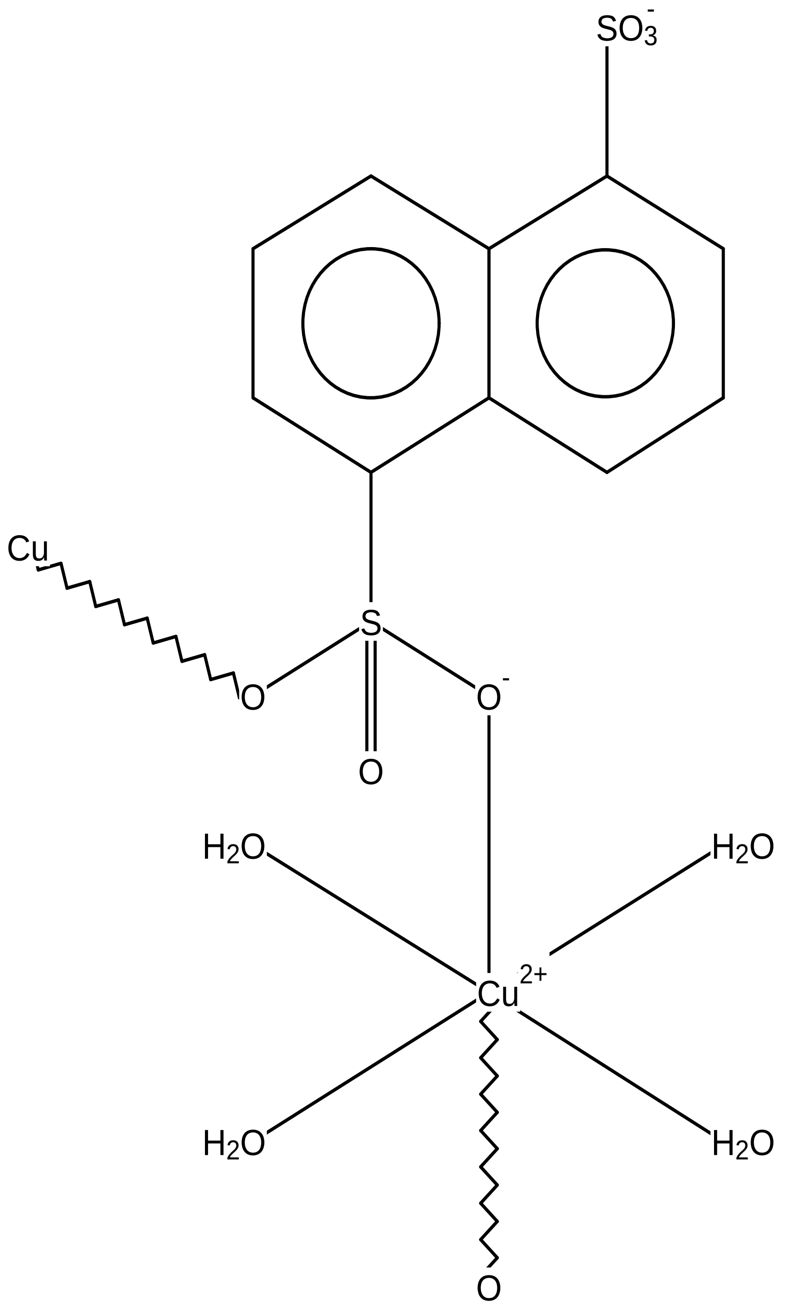

Parameters

Fragment 1

**DIST1 (D)**	1.955
**DIST2 (D)**	1.954
**DIST3 (D)**	1.953
**DIST4 (D)**	1.979
**DIST5 (D)**	2.337
**DIST6 (D)**	2.449

Search: search2 (Wed Jul 26 09:11:25 2017): Hit 141

PAXTUE01

Reference:	B.Kozlevcar, M.Humar, P.Strauch, I.Leban (2005) *Z.Naturforsch.,B:Chem.Sci.* ,**60,**1273
Formula:	$C_{16} H_{18} Cu_1 O_8$,2($H_2 O_1$)
Compound Name:	trans-Diaqua-bis(vanillinato)-copper(ii) dihydrate
Synonym:	trans-Diaqua-bis(4-formyl-2-methoxyphenolato)-copper(ii) dihydrate

Space Group:	P21/a	***Cell:***	***a*** 6.696(0)	***b*** 12.825(0)	***c*** 10.953(1)
Space Group No.:	14	***(Å,°)***	α 90.00	β 100.45(0)	γ 90.00
R-Factor (%):	2.87	***Temperature(K):*** 293	***Density(g/cm³):*** 1.572		

Parameters

Fragment 1

**DIST1 (D)**	1.994
**DIST2 (D)**	1.950
**DIST3 (D)**	1.950
**DIST4 (D)**	1.994
**DIST5 (D)**	2.334
**DIST6 (D)**	2.334

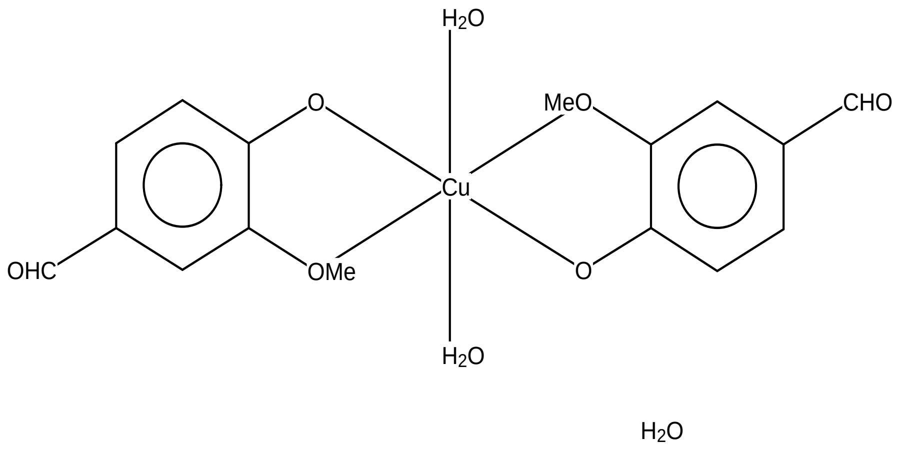

Search: search2 (Wed Jul 26 09:11:25 2017): Hit 142

PAXTUE02

Reference: B.Kozlevcar, M.Humar, P.Strauch, I.Leban (2005)
Z.Naturforsch.,B:Chem.Sci. ,**60,**1273

Formula: C_{16} H_{18} Cu_1 O_8,2(H_2 O_1)

Compound Name: trans-Diaqua-bis(vanillinato)-copper(ii) dihydrate

Synonym: trans-Diaqua-bis(4-formyl-2-methoxyphenolato)-copper(ii) dihydrate

Space Group:	P21/a	***Cell:***	***a*** 6.543(0)	***b***	12.885(0)	***c***	10.885(0)
Space Group No.:	14	***(Å,°)***	α 90.00	β	98.61(0)	γ	90.00

R-Factor (%): 2.39 ***Temperature(K):*** 150 ***Density(g/cm^3):*** 1.603

Parameters

Fragment 1

**DIST1 (D)**	1.983
**DIST2 (D)**	1.959
**DIST3 (D)**	1.959
**DIST4 (D)**	1.983
**DIST5 (D)**	2.327
**DIST6 (D)**	2.327

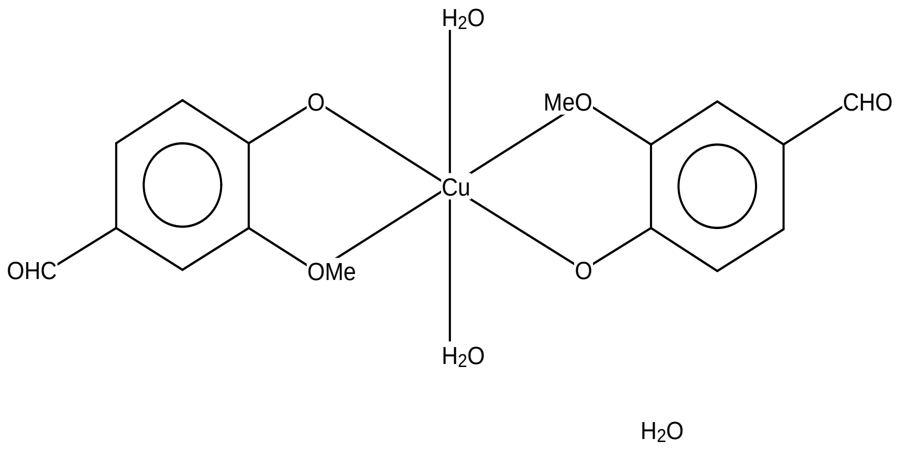

Search: search2 (Wed Jul 26 09:11:25 2017): Hit 143

PEWDUS

Reference: J.Olchowka, C.Falaise, C.Volkringer, N.Henry, T.Loiseau (2013) *Chem.-Eur.J.* ,**19,**2012

Formula: $(C_8\ H_8\ Cu_1\ O_{13}\ U_2)n,n(H_2\ O_1)$

Compound Name: catena-[(μ_3- Isophthalato)-bis(μ_3-oxo)-tris(μ_2-oxo)-diaqua-dioxo-copper-di-uranium monohydrate]

Space Group:	P-1	***Cell:***	***a*** 8.564(0)	***b*** 8.750(0)	***c*** 12.423(0)
Space Group No.:	2	***(Å,°)***	α 84.92(0)	β 77.17(0)	γ 61.93(0)

R-Factor (%): 2.39 ***Temperature(K):*** 293 ***Density(g/cm³):*** 3.607

Parameters

Fragment 1

**DIST1 (D)**	2.001
**DIST2 (D)**	1.912
**DIST3 (D)**	1.977
**DIST4 (D)**	1.991
**DIST5 (D)**	2.350
**DIST6 (D)**	2.692

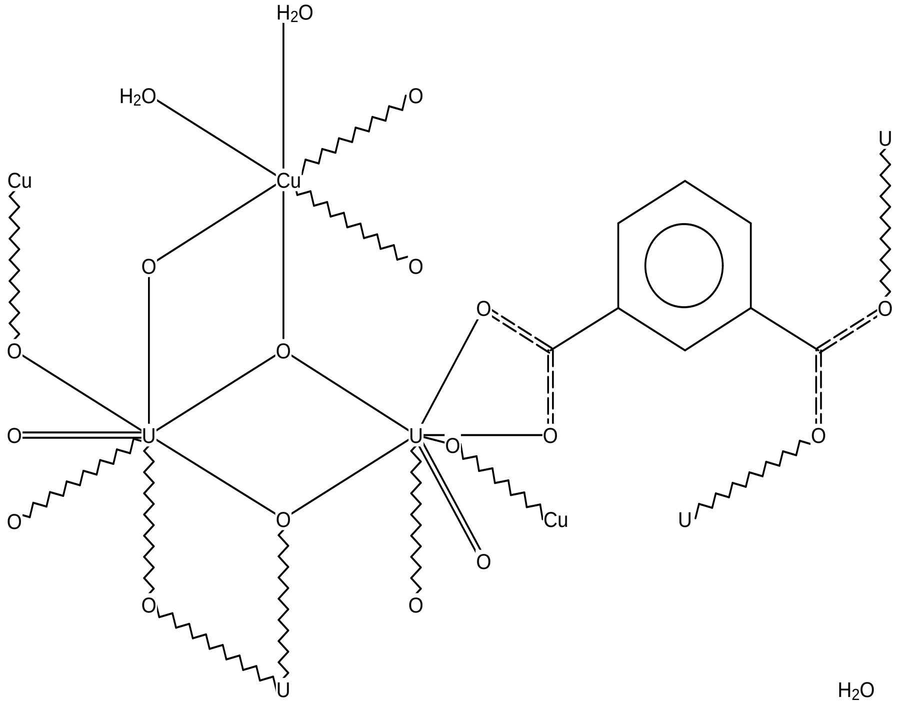

Search: search2 (Wed Jul 26 09:11:25 2017): Hit 144

PEWFAA

Reference:	J.Olchowka, C.Falaise, C.Volkringer, N.Henry, T.Loiseau (2013) *Chem.-Eur.J.* ,**19,**2012
Formula:	$(C_{12}\ H_8\ Cu_1\ O_{20}\ U_2)n$
Compound Name:	catena-[(μ_8-Benzene-1,2,3,4,5,6-hexacarboxylato)-bis(μ_2-oxo)-tetra-aqua-dioxo-copper-di-uranium]

Space Group:	P21/c	***Cell:***	***a*** 7.984(0)	***b*** 11.546(0)	***c*** 10.972(0)
Space Group No.:	14	***(Å,°)***	α 90.00	β 105.35(0)	γ 90.00

R-Factor (%): 1.85 ***Temperature(K):*** 293 ***Density(g/cm³):*** 3.445

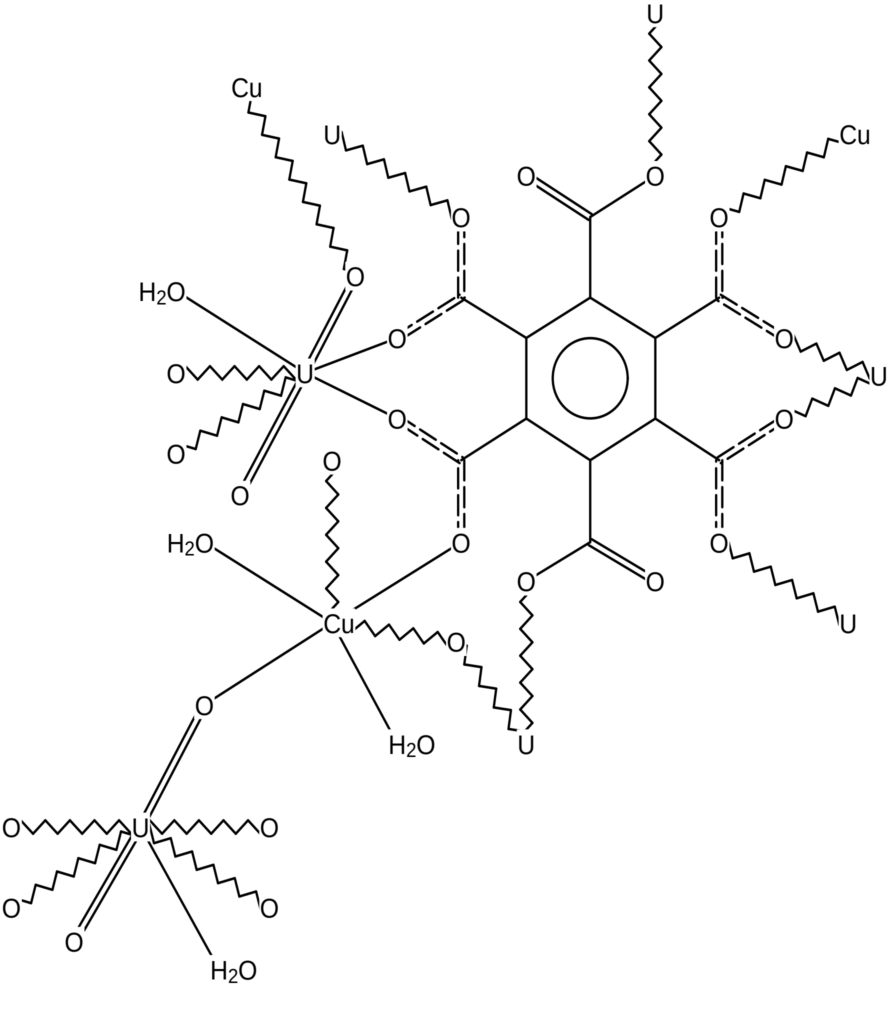

Parameters

Fragment 1

**DIST1 (D)**	1.972
**DIST2 (D)**	1.972
**DIST3 (D)**	1.917
**DIST4 (D)**	1.917
**DIST5 (D)**	2.561
**DIST6 (D)**	2.561

Search: search2 (Wed Jul 26 09:11:25 2017): Hit 145

PEWFEE

Reference: J.Olchowka, C.Falaise, C.Volkringer, N.Henry, T.Loiseau (2013) *Chem.-Eur.J.* ,**19,**2012

Formula: $(C_{10} H_2 Cu_1 O_{10} U_1)n$

Compound Name: catena-[(μ_8-Benzene-1,2,4,5-tetracarboxylato)-bis(μ_2-oxo)-copper-uranium]

Space Group:	P-1	***Cell:***	***a*** 4.996(0)	***b*** 6.971(0)	***c*** 8.514(0)
Space Group No.:	2	***(Å,°)***	α 97.31(0)	β 106.42(0)	γ 91.96(0)

R-Factor (%): 2.67 ***Temperature(K):*** 293 ***Density(g/cm³):*** 3.439

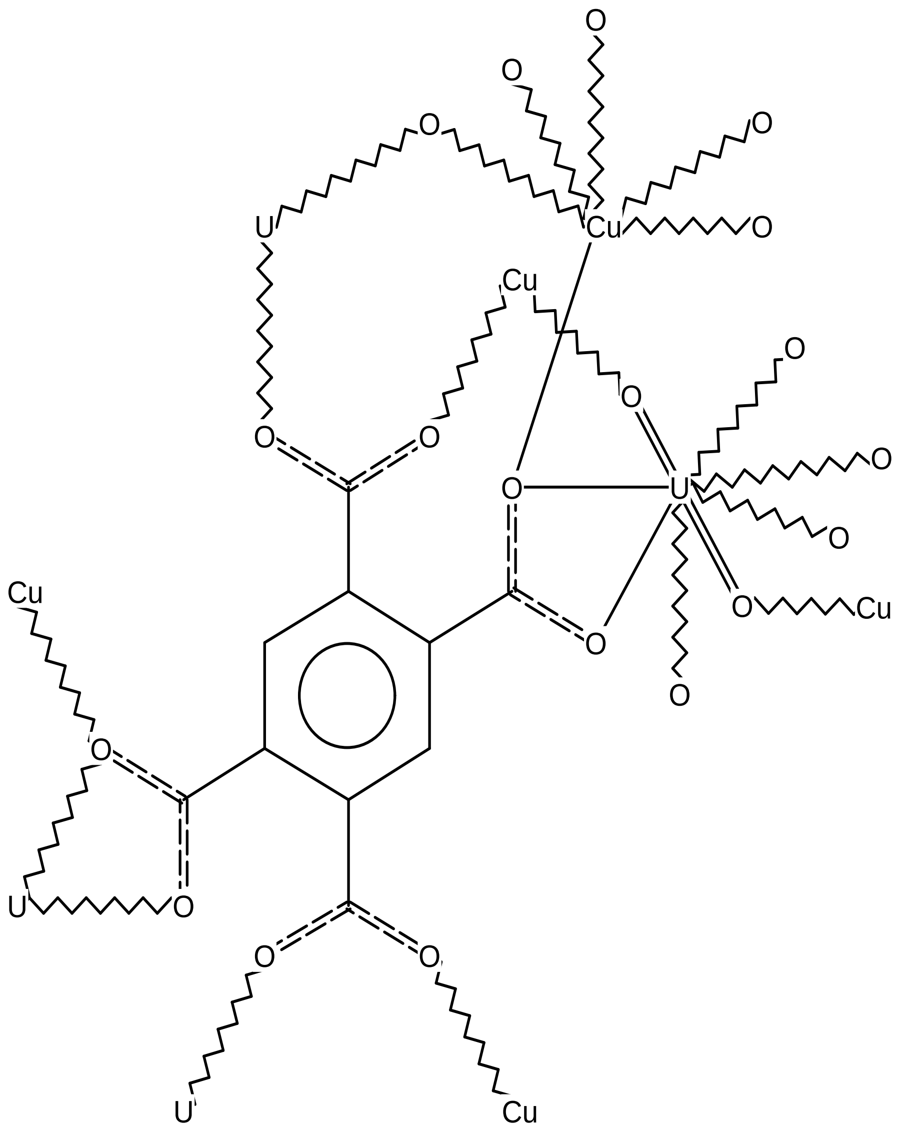

Parameters

Fragment 1

**DIST1 (D)**	1.922
**DIST2 (D)**	1.969
**DIST3 (D)**	1.969
**DIST4 (D)**	1.922
**DIST5 (D)**	2.745
**DIST6 (D)**	2.745

Search: search2 (Wed Jul 26 09:11:25 2017): Hit 146

PEWFII

Reference:	J.Olchowka, C.Falaise, C.Volkringer, N.Henry, T.Loiseau<br>(2013) *Chem.-Eur.J.* ,**19,**2012
Formula:	$(C_{10}\ H_6\ Cu_1\ O_{12}\ U_1)$n,4n$(H_2\ O_1)$
Compound Name:	catena-((μ_6-Benzene-1,2,4,5-tetracarboxylato)-bis(μ_2-oxo)-diaqua-copper-uranium tetrahydrate)

Space Group:	P-1	***Cell:***	***a*** 7.107(0)	***b*** 7.223(0)	***c*** 9.205(0)
Space Group No.:	2	***(Å,°)***	α 95.38(0)	β 91.26(0)	γ 114.89(0)

R-Factor (%):	1.76	***Temperature(K):***	293	***Density(g/cm³):***	2.698

Parameters

Fragment 1

**DIST1 (D)**	1.937
**DIST2 (D)**	1.937
**DIST3 (D)**	1.969
**DIST4 (D)**	1.969
**DIST5 (D)**	2.438
**DIST6 (D)**	2.438

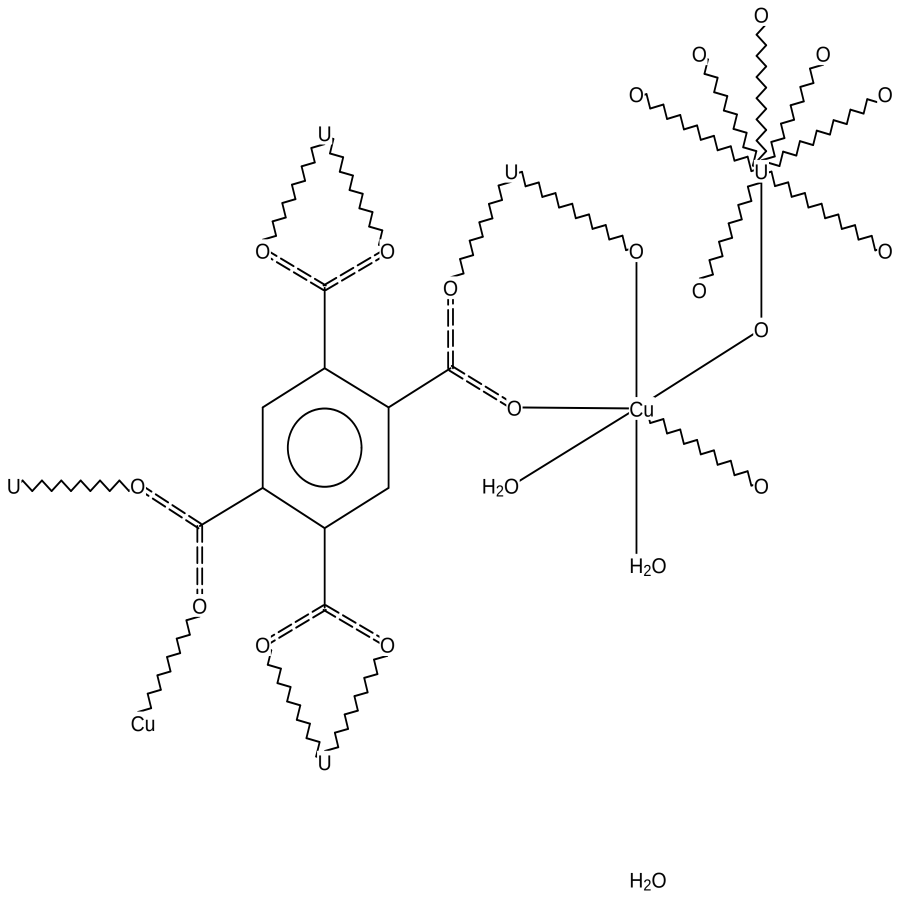

Search: search2 (Wed Jul 26 09:11:25 2017): Hit 147

PEWFII01

Reference: Qing Lin Guan, Xue Gao, Jing Liu, Wen Juan Wei, Yong Heng Xing, Feng Ying Bai (2016) *J.Coord.Chem.* ,**69,**1026

Formula: $(C_{10} H_6 Cu_1 O_{12} U_1)n,4(H_2 O_1)$

Compound Name: catena-[(μ-benzene-1,2,4,5-tetracarboxylato)-bis(μ-oxo)-diaqua-copper-uranium tetrahydrate]

Space Group:	P-1	***Cell:***	***a*** 7.116(0)	***b*** 7.235(0)	***c*** 9.214(0)
Space Group No.:	2	***(Å,°)***	α 95.36(0)	β 91.22(0)	γ 114.93(0)

R-Factor (%): 2.33 ***Temperature(K):*** 296 ***Density(g/cm^3):*** 2.688

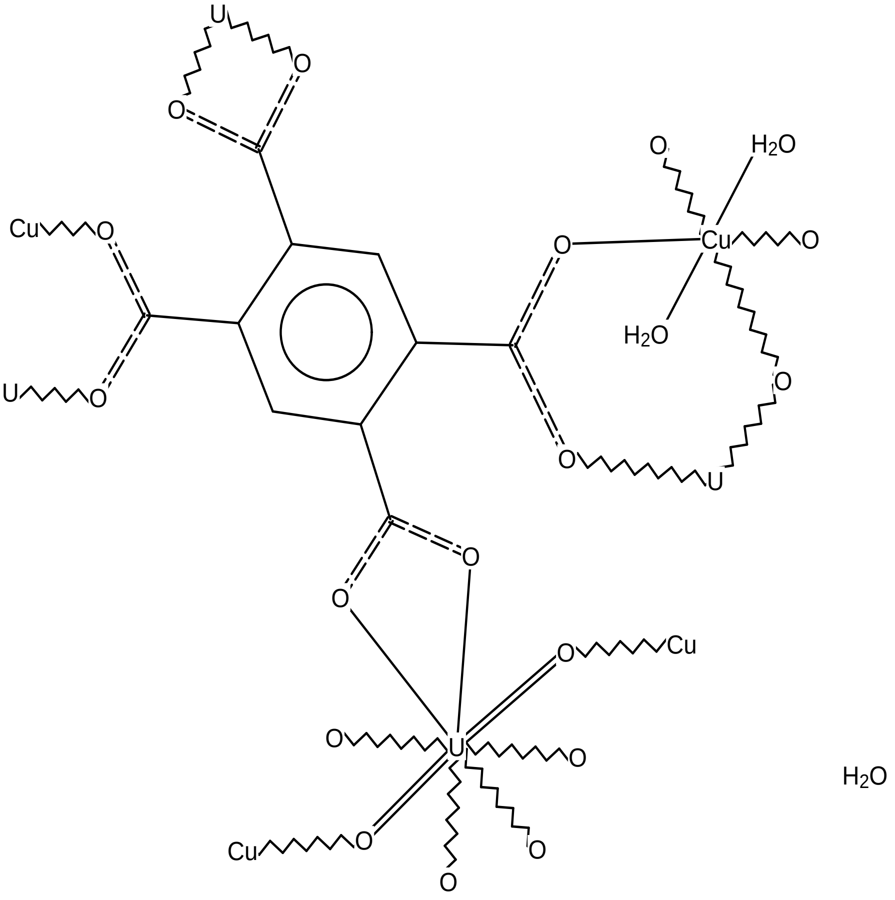

Parameters

Fragment 1

**DIST1 (D)**	1.940
**DIST2 (D)**	1.972
**DIST3 (D)**	1.940
**DIST4 (D)**	1.972
**DIST5 (D)**	2.439
**DIST6 (D)**	2.439

Search: search2 (Wed Jul 26 09:11:25 2017): Hit 148

PEWFOO

Reference: J.Olchowka, C.Falaise, C.Volkringer, N.Henry, T.Loiseau (2013) *Chem.-Eur.J.* ,**19,**2012

Formula: $(C_{16} H_{12} Cu_1 O_{12} U_1)n$

Compound Name: catena-(bis(μ_3-Phthalato)-bis(μ_2-oxo)-diaqua-copper-uranium)

Space Group:	P-1	***Cell:***	***a*** 6.990(0)	***b*** 7.091(0)	***c*** 10.071(1)
Space Group No.:	2	***(Å,°)***	α 100.54(0)	β 96.43(0)	γ 114.22(0)

R-Factor (%): 2.78 ***Temperature(K):*** 293 ***Density(g/cm^3^):*** 2.658

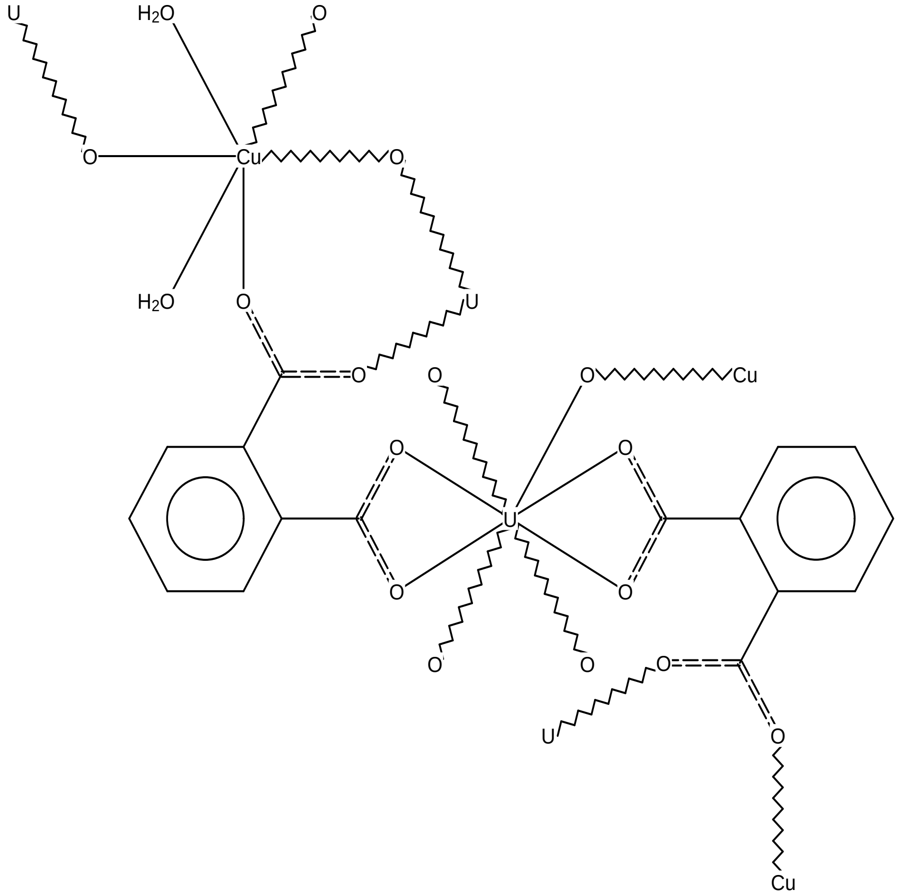

Parameters

Fragment 1

**DIST1 (D)**	1.973
**DIST2 (D)**	1.973
**DIST3 (D)**	1.923
**DIST4 (D)**	1.923
**DIST5 (D)**	2.517
**DIST6 (D)**	2.517

Search: search2 (Wed Jul 26 09:11:25 2017): Hit 149

PEWLEI

Reference: R.Kaplonek, G.Fechtel, U.Baumeister, H.Hartung (1993)
Z.Anorg.Allg.Chem. ,**619,**1616

Formula: $(C_8 H_{12} Cu_1 O_9)n,2n(H_2 O_1)$

Compound Name: catena((Diaqua-5-exo,6-cis-dihydroxy-7-oxabicyclo(2.2.1)heptane-2,3-dicarboxylato-O,O',O'')-copper dihydrate)

Space Group:	P-1	***Cell:***	***a***	7.273(2)	***b***	7.457(2)	***c***	12.135(3)
Space Group No.:	2	***(Å,°)***	α	74.22(1)	β	79.93(1)	γ	70.11(1)

R-Factor (%): 2.70 ***Temperature(K):*** 295 ***Density(g/cm³):*** 1.970

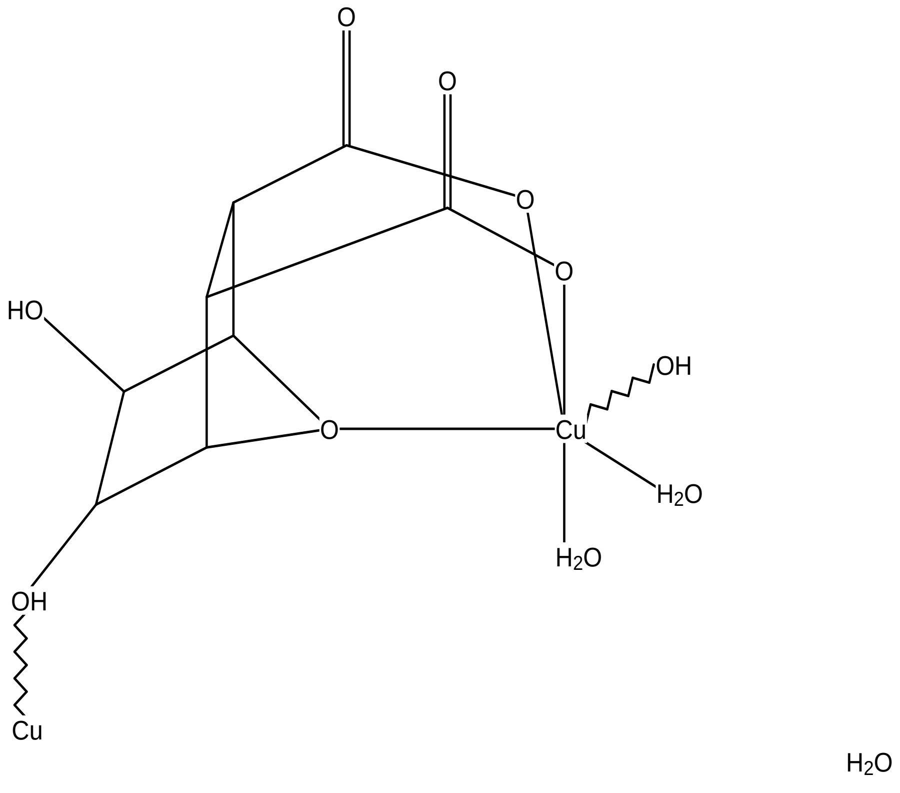

Parameters

Fragment 1

**DIST1 (D)**	2.006
**DIST2 (D)**	1.946
**DIST3 (D)**	2.006
**DIST4 (D)**	1.958
**DIST5 (D)**	2.316
**DIST6 (D)**	2.384

Search: search2 (Wed Jul 26 09:11:25 2017): Hit 150

PHXCUA01

Reference:	Yong-hong Zhou (2010)<br>*Huaxue Yanjiu(Chin.)(Chem.Res.)* ,**21,**1-1
Formula:	C_{16} H_{16} Cl_2 Cu_1 O_8
Compound Name:	Diaqua-bis(((4-chlorophenyl)oxy)acetato)-copper(ii)

Space Group:	P-1	***Cell:***	***a***	5.115(0)	***b***	7.064(0)	***c***	13.264(0)
Space Group No.:	2	***(Å,°)***	α	81.78(0)	β	86.36(0)	γ	76.40(0)

R-Factor (%): 2.64 ***Temperature(K):*** 298 ***Density(g/cm³):*** 1.696

Parameters

Fragment 1

**DIST1 (D)**	1.949
**DIST2 (D)**	1.951
**DIST3 (D)**	1.951
**DIST4 (D)**	1.949
**DIST5 (D)**	2.402
**DIST6 (D)**	2.402

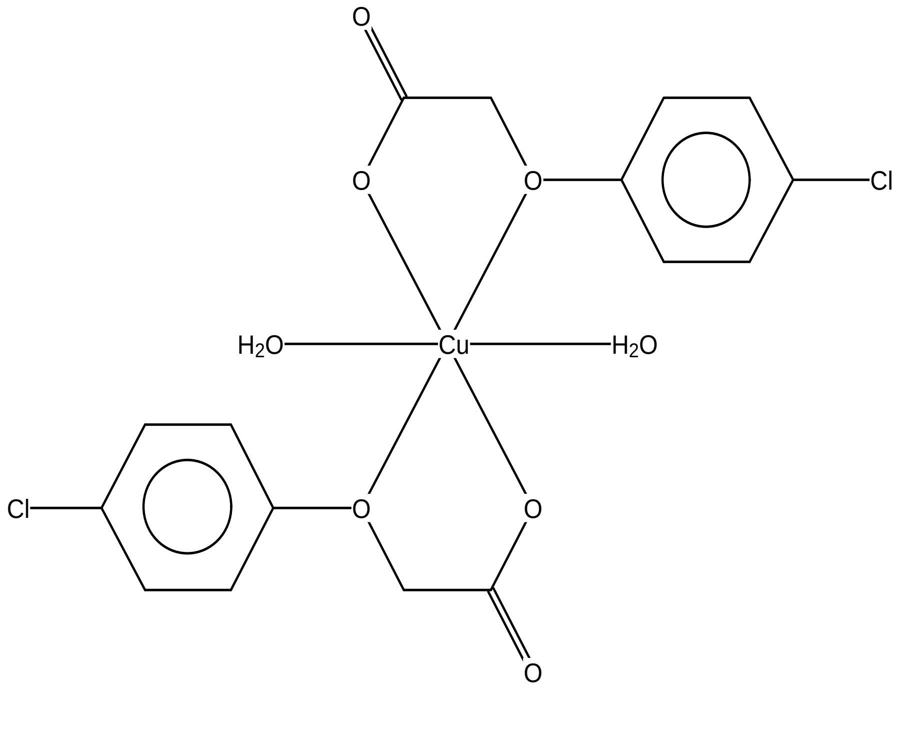

Search: search2 (Wed Jul 26 09:11:25 2017): Hit 151

PIDWUV

Reference:	Chuan-Bi Li, Ying-Chun Gao (2007)<br>*Acta Crystallogr.,Sect.E:Struct.Rep.Online* ,**63,**m1337
Formula:	$2(C_6 H_7 N_2 O_1{}^{1+}),C_4 H_4 Cu_1 O_{10}{}^{2-}$
Compound Name:	bis(4-Carboxamidopyridinium) diaqua-dioxalato-copper(ii)

Space Group:	P-1	***Cell:***	***a*** 6.788(2)	***b*** 6.957(2)	***c*** 10.849(3)		
Space Group No.:	2	***(Å,°)***	α 76.13(0)	β 75.20(0)	γ 79.48(0)		
R-Factor (%):	2.50	***Temperature(K):***	295	***Density(g/cm³):***	1.817		

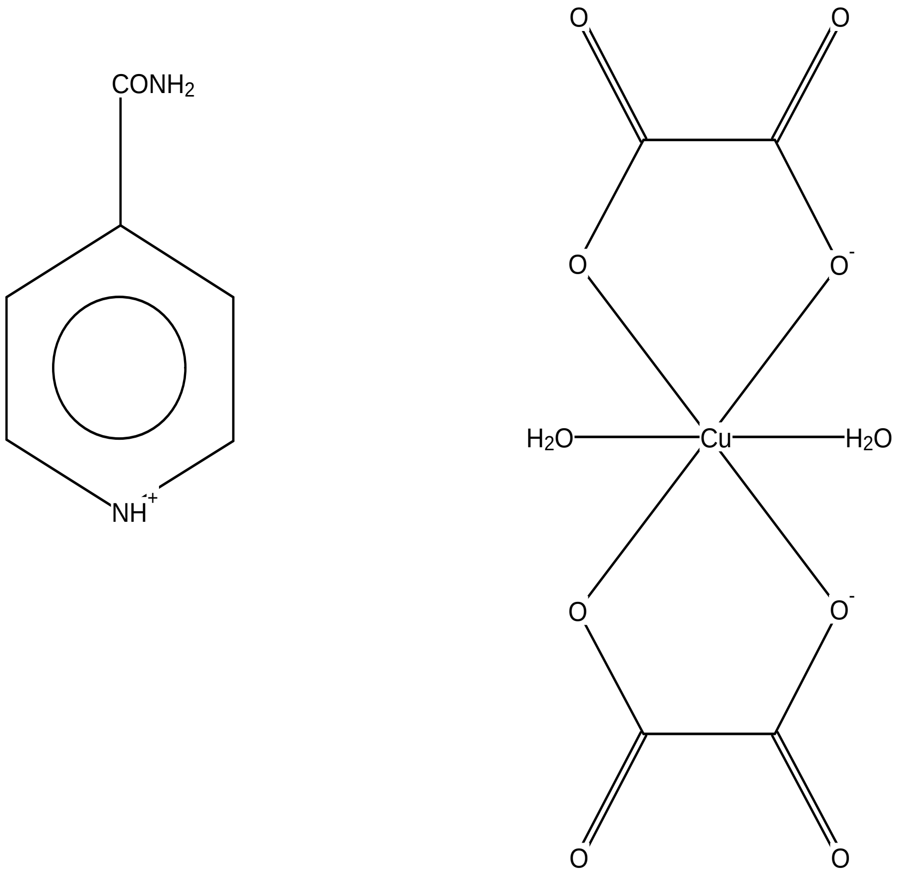

Parameters

Fragment 1

**DIST1 (D)**	1.943
**DIST2 (D)**	1.987
**DIST3 (D)**	1.943
**DIST4 (D)**	1.987
**DIST5 (D)**	2.488
**DIST6 (D)**	2.488

Search: search2 (Wed Jul 26 09:11:25 2017): Hit 152

PIJSIL

Reference: Jiang-Bo She, Guo-Fang Zhang, Feng-Qi Zhao, Zhong-Li Lei, Xue-Zhong Fan (2007) *Struct.Chem.* ,**18,**373

Formula: C_{10} H_{12} Cu_1 N_6 O_{16}

Compound Name: tetra-aqua-(3,5-dinitro-4-pyridone-N-hydroxylate)-copper(ii)

Space Group:	P21/n	***Cell:***	***a***	8.370(1)	***b***	9.931(1)	***c***	10.577(1)
Space Group No.:	14	***(Å,°)***	α	90.00	β	98.02(0)	γ	90.00

R-Factor (%): 2.76 ***Temperature(K):*** 273 ***Density(g/cm^3):*** 2.044

Parameters

Fragment 1

**DIST1 (D)**	2.088
**DIST2 (D)**	1.942
**DIST3 (D)**	1.942
**DIST4 (D)**	2.088
**DIST5 (D)**	2.233
**DIST6 (D)**	2.233

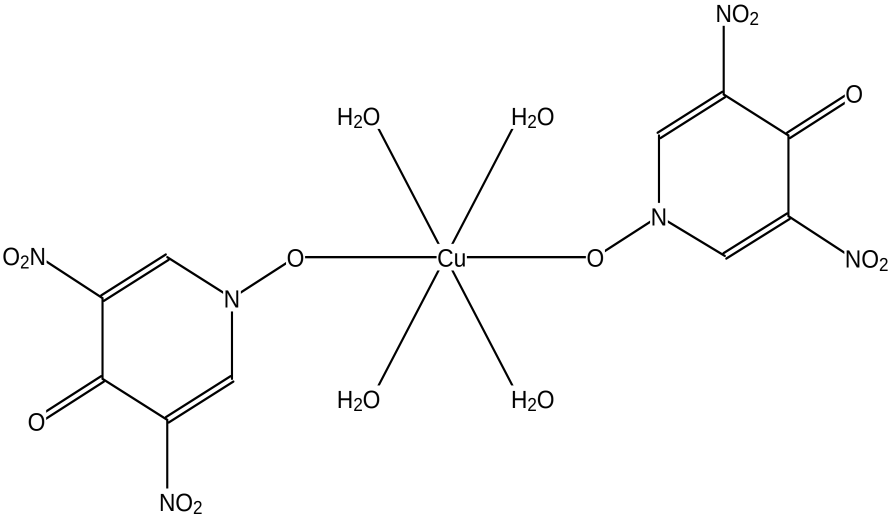

Search: search2 (Wed Jul 26 09:11:25 2017): Hit 153

QEKFUI01

Reference:	F.S.Delgado, C.Ruiz-Perez, J.Sanchiz, F.Lloret, M.Julve<br>(2006) *CrystEngComm* ,**8,**507
Formula:	$(C_6\ H_{12}\ Cs_2\ Cu_1\ O_{12})n$
Compound Name:	catena-(bis((μ_6-Malonato)-(μ_2-aqua)-(aqua))-copper(ii)-di-cesium)

Space Group:	P-1	***Cell:***	***a***	7.117(0)	***b***	7.132(0)	***c***	7.521(0)
Space Group No.:	2	***(Å,°)***	α	87.33(0)	β	79.43(0)	γ	86.70(0)

R-Factor (%): 2.87 ***Temperature(K):*** 293 ***Density(g/cm³):*** 2.686

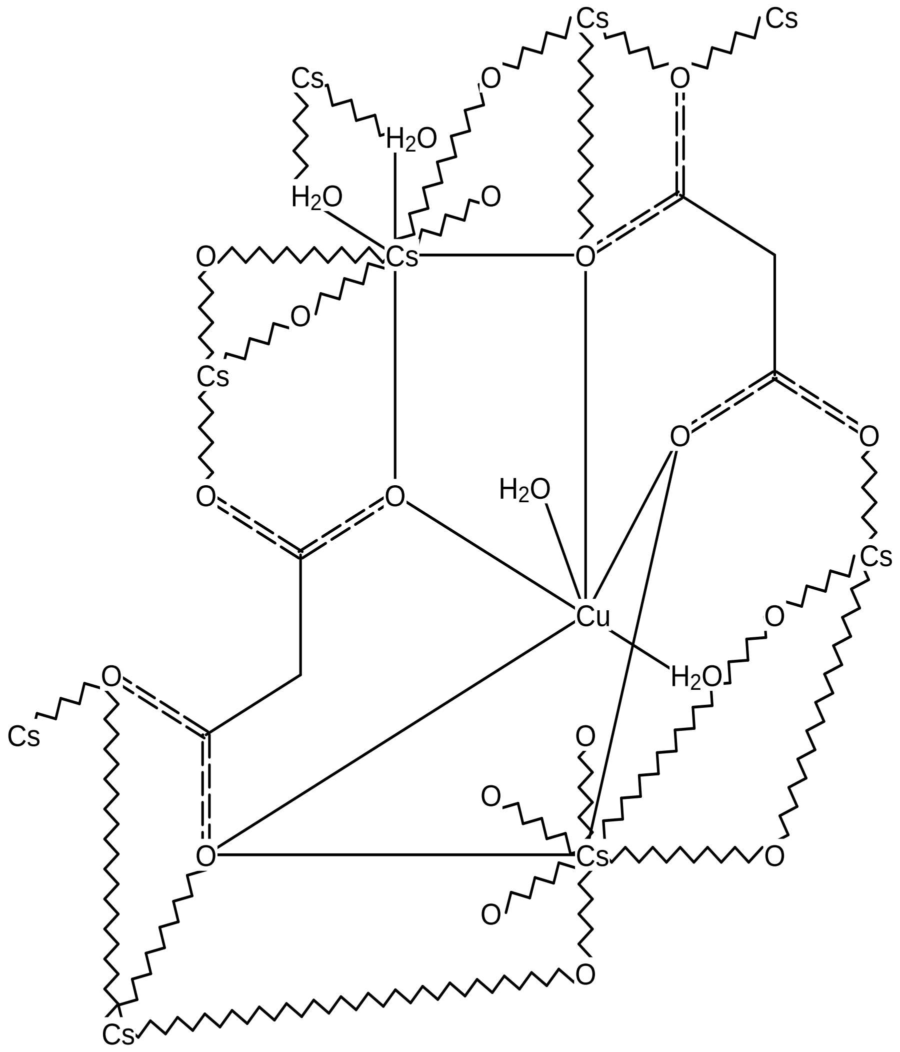

Parameters

Fragment 1

**DIST1 (D)**	1.970
**DIST2 (D)**	1.935
**DIST3 (D)**	1.970
**DIST4 (D)**	1.935
**DIST5 (D)**	2.414
**DIST6 (D)**	2.414

Search: search2 (Wed Jul 26 09:11:25 2017): Hit 154

QIGFER

Reference: D.V.Baxter, K.G.Caulton, Wen-Chung Chiang, M.H.Chisholm, V.F.DiStasi, S.G.Dutremez, J.D.Martin, W.E.Streib (2001) *New J.Chem.* ,**25,**400

Formula: $(C_{14} H_{18} Cu_1 O_{12})n$

Compound Name: catena-(bis(μ_2-tris(Methoxycarbonyl)methanato-O,O',O'')-copper(ii))

Space Group:	P21/n	***Cell:***	***a*** 6.622(0)	***b*** 10.554(0)	***c*** 13.220(1)		
Space Group No.:	14	***(Å,°)***	α 90.00	β 102.72(0)	γ 90.00		
R-Factor (%):	2.72	***Temperature(K):***	151	***Density(g/cm³):***	1.628		

Parameters

Fragment 1

**DIST1 (D)**	1.912
**DIST2 (D)**	1.925
**DIST3 (D)**	1.912
**DIST4 (D)**	1.925
**DIST5 (D)**	2.584
**DIST6 (D)**	2.584

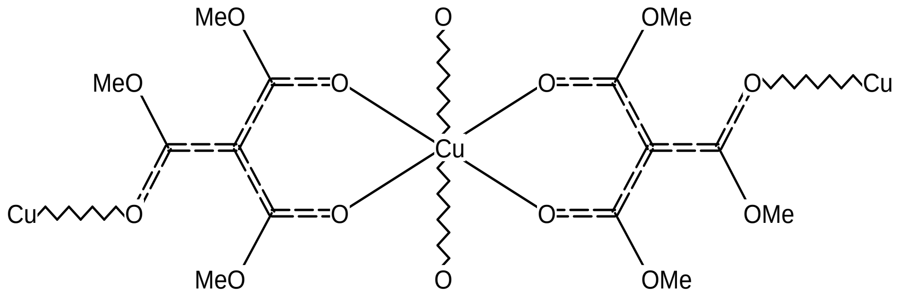

Search: search2 (Wed Jul 26 09:11:25 2017): Hit 155

QIXNUI

Reference: Zhi-Kun Qu, Kai Yu, Zhi-Feng Zhao, Zhan-hua Su, Jing-Quan Sha, Chun-Mei Wang, Bai-Bin Zhou (2014) *Dalton Trans.* , **43,**6744

Formula: $(C_{84} H_{48} Cu_{8} Mo_{12} N_{12} O_{62})n$

Compound Name: catena-[bis(μ_6-oxido)-hexakis(μ_2-oxalato)-hexatricontakis(μ_2-oxido)-hexakis(1,10-phenanthroline)-octa-copper-dodeca-molybdenum]

Space Group:	R-3c	***Cell:***	***a*** 14.972(0)	***b*** 14.972(0)	***c*** 80.713(3)		
Space Group No.:	167	***(Å,°)***	α 90.00	β 90.00	γ 120.00		
R-Factor (%):	2.87	***Temperature(K):***	293	***Density(g/cm^3):***	2.465		

Parameters

Fragment 1

**DIST1 (D)**	2.080
**DIST2 (D)**	2.080
**DIST3 (D)**	2.080
**DIST4 (D)**	2.080
**DIST5 (D)**	2.080
**DIST6 (D)**	2.080

n-$Mo_{12}Cu_8[\mu_6\text{-}O]_2[\mu_2\text{-}C_2O_4\text{-}\kappa^4O,O',O'',O''']_6$--

--$[\mu_2\text{-}O]_{36}[C_{12}H_8N_2\text{-}\kappa^2N,N']_6$

Search: search2 (Wed Jul 26 09:11:25 2017): Hit 156

QQQEXG01

Reference:	F.S.Delgado, C.Ruiz-Perez, J.Sanchiz, F.Lloret, M.Julve<br>(2006) *CrystEngComm* ,**8,**530
Formula:	$(H_4\ N_1\ ^{1+})$2n,n$(C_6\ H_4\ Cu_1\ O_8\ ^{2-})$
Compound Name:	catena-(Diammonium bis(μ_2-malonato)-copper(ii))

Space Group:	Fdd2	***Cell:***	***a*** 13.848(0)	***b*** 16.454(0)	***c*** 9.107(0)
Space Group No.:	43	***(Å,°)***	α 90.00	β 90.00	γ 90.00
R-Factor (%):	1.97	***Temperature(K):***	293	***Density(g/cm³):***	1.944

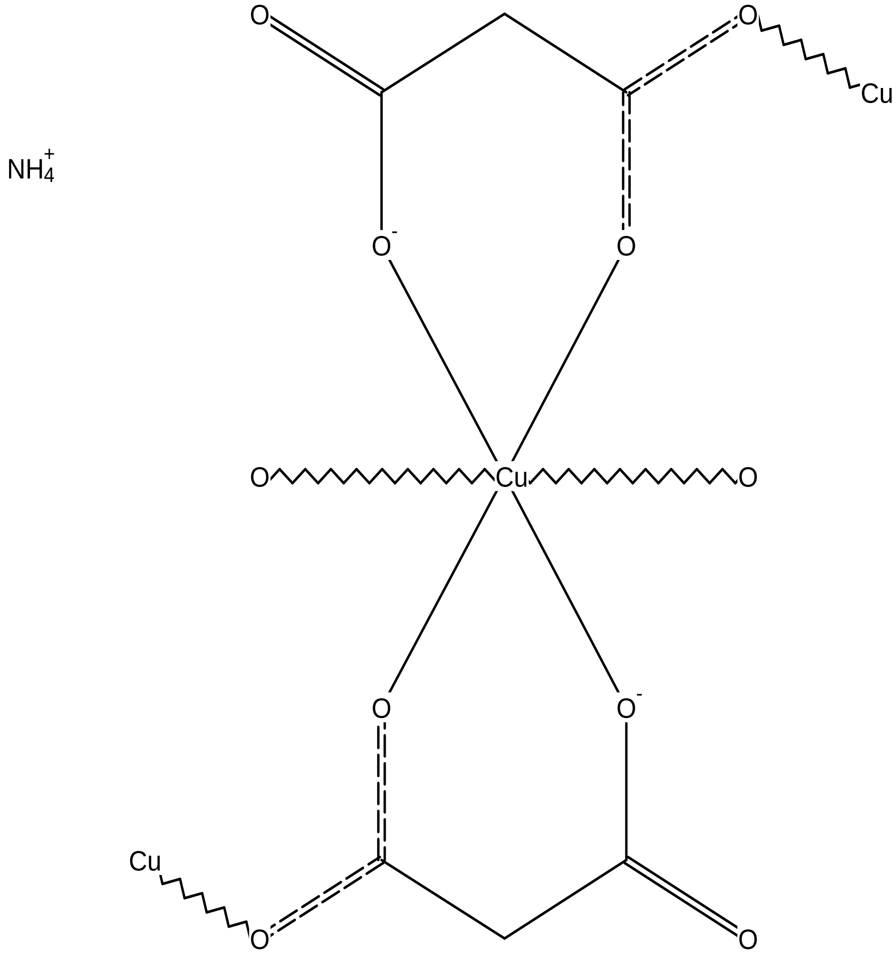

Parameters

Fragment 1

**DIST1 (D)**	1.955
**DIST2 (D)**	1.939
**DIST3 (D)**	1.939
**DIST4 (D)**	1.955
**DIST5 (D)**	2.481
**DIST6 (D)**	2.481

Search: search2 (Wed Jul 26 09:11:25 2017): Hit 157

QQQEXG02

Reference: F.S.Delgado, C.Ruiz-Perez, J.Sanchiz, F.Lloret, M.Julve (2006) *CrystEngComm* ,**8,**530

Formula: $(H_4 N_1 {}^{1+})2n,n(C_6 H_4 Cu_1 O_8 {}^{2-})$

Compound Name: catena-(Diammonium bis(μ_2-malonato)-copper(ii))

Space Group:	Fdd2	***Cell:***	***a*** 13.656(1)	***b*** 16.444(0)	***c*** 9.088(0)
Space Group No.:	43	***(Å,°)***	α 90.00	β 90.00	γ 90.00

R-Factor (%): 2.26 ***Temperature(K):*** 100 ***Density(g/cm^3):*** 1.977

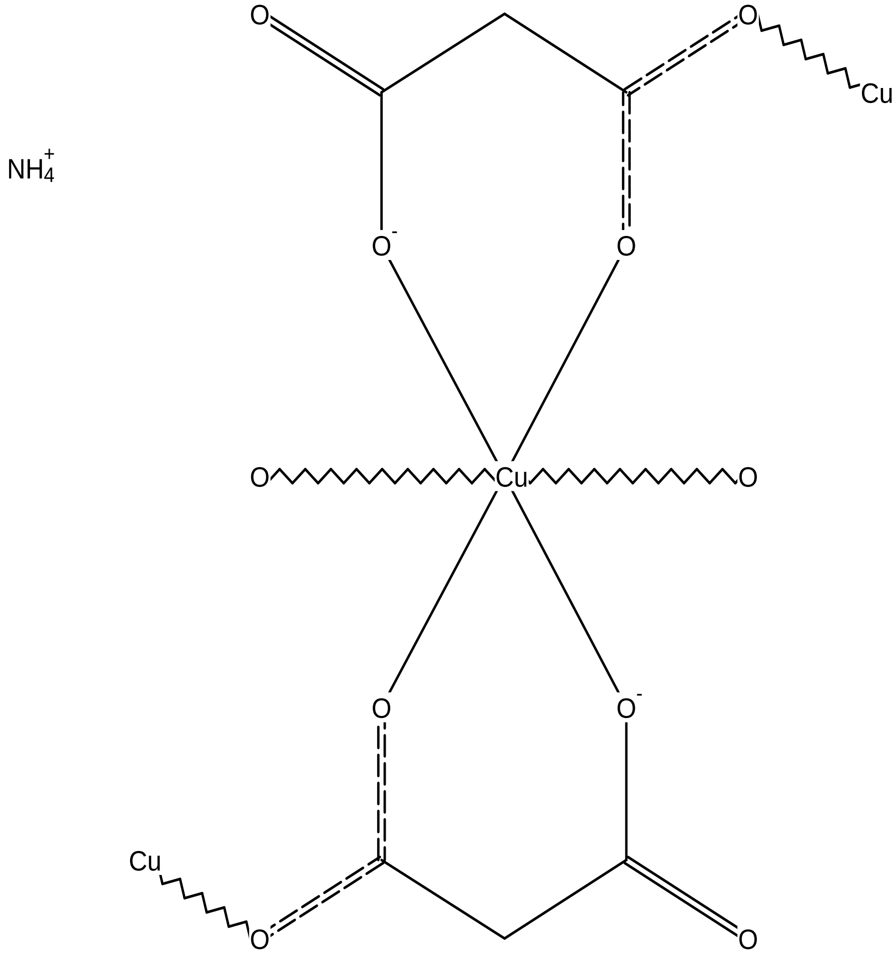

Parameters

Fragment 1

**DIST1 (D)**	1.958
**DIST2 (D)**	1.942
**DIST3 (D)**	1.942
**DIST4 (D)**	1.958
**DIST5 (D)**	2.436
**DIST6 (D)**	2.436

Search: search2 (Wed Jul 26 09:11:25 2017): Hit 158

QUTQAZ

Reference:	Junqing Zhang, Cun Li, Juanjuan Wang, Mei Zhu, Licun Li (2016) *Eur.J.Inorg.Chem.* ,1383
Formula:	C_{46} H_{20} Cu_1 F_{36} O_{18} Tb_2
Compound Name:	bis(μ_3-2-Oxy-4-methoxybenzaldehyde)-bis(μ_2-hexafluoroacetylacetonato-O,O')-tetrakis(hexafluoroacetylacetonato)-copper-di-terbium

Space Group:	P-1	***Cell:***	***a***	9.881(2)	***b***	11.136(2)	***c***	15.946(3)
Space Group No.:	2	***(Å,°)***	α	100.44(3)	β	99.11(3)	γ	113.24(3)

R-Factor (%):	2.67	***Temperature(K):***	113	***Density(g/cm³):***	2.086

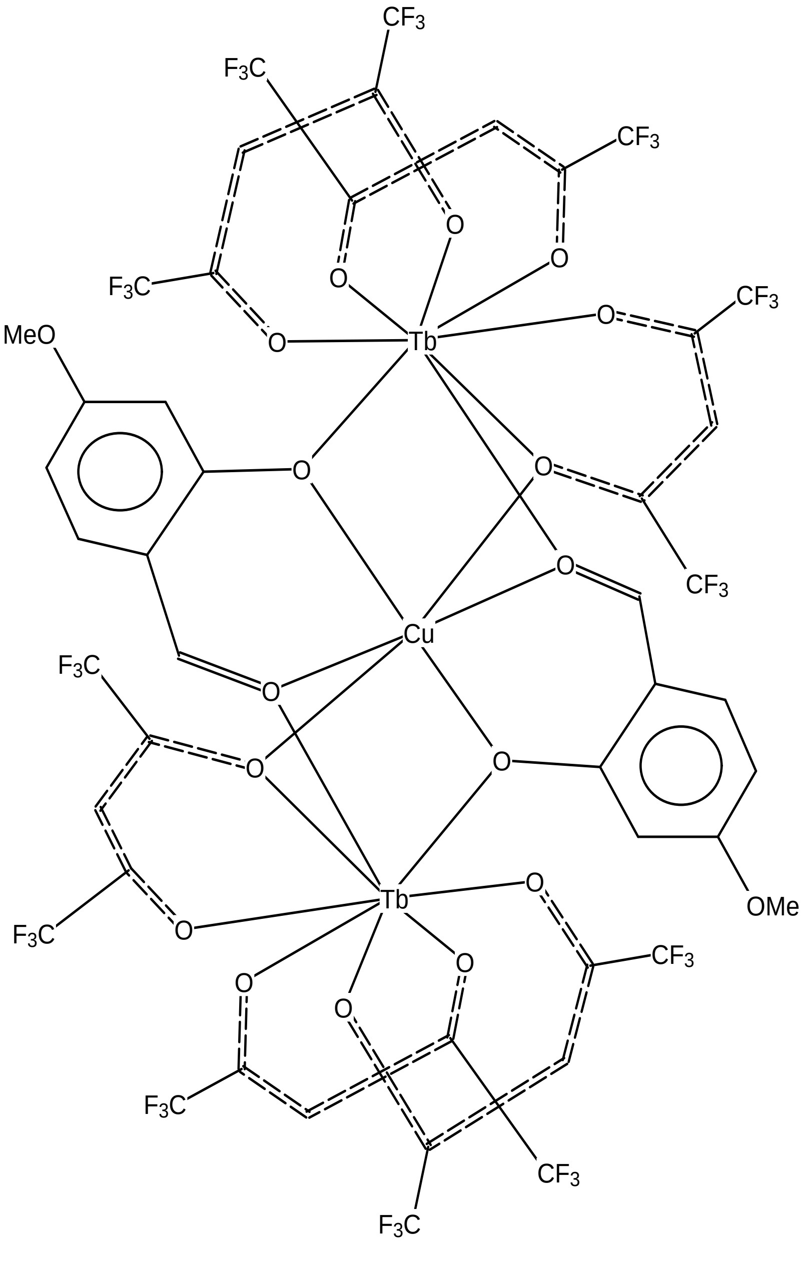

Parameters

Fragment 1

**DIST1 (D)**	1.912
**DIST2 (D)**	1.946
**DIST3 (D)**	1.912
**DIST4 (D)**	1.946
**DIST5 (D)**	2.487
**DIST6 (D)**	2.487

Search: search2 (Wed Jul 26 09:11:25 2017): Hit 159

QUTQED

Reference:	Junqing Zhang, Cun Li, Juanjuan Wang, Mei Zhu, Licun Li (2016) *Eur.J.Inorg.Chem.* ,1383
Formula:	C_{46} H_{20} Cu_1 F_{36} Gd_2 O_{18}
Compound Name:	bis(μ_3-2-Oxy-4-methoxybenzaldehyde)-bis(μ_2-hexafluoroacetylacetonato-O,O')-tetrakis(hexafluoroacetylacetonato)-copper-di-gadolinium

Space Group:	P-1	***Cell:***	***a*** 9.879(2)	***b***	11.137(3)	***c***	15.918(4)
Space Group No.:	2	***(Å,°)***	α 100.33(0)	β	98.88(0)	γ	113.42(0)
R-Factor (%):	2.20	***Temperature(K):***	113	***Density(g/cm³):***	2.086		

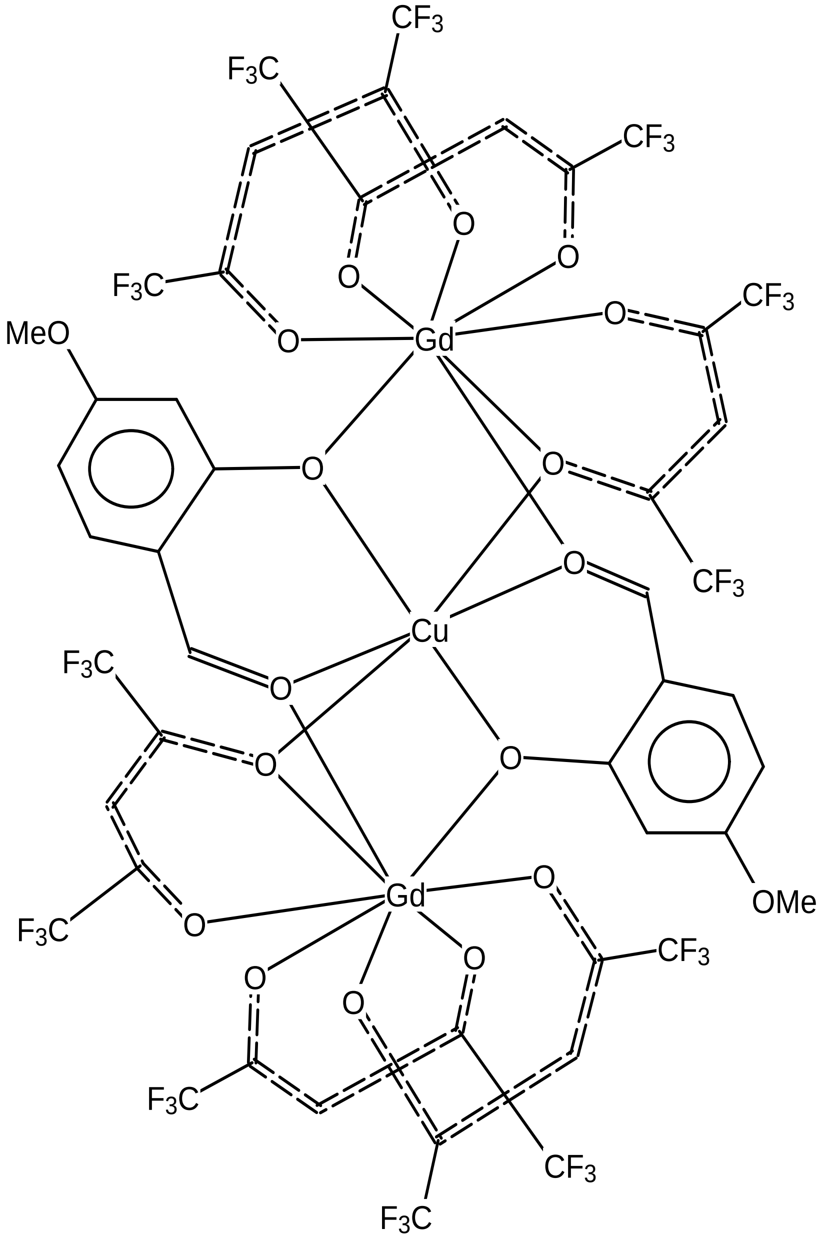

Parameters

Fragment 1

**DIST1 (D)**	1.906
**DIST2 (D)**	1.945
**DIST3 (D)**	1.906
**DIST4 (D)**	1.945
**DIST5 (D)**	2.482
**DIST6 (D)**	2.482

Search: search2 (Wed Jul 26 09:11:25 2017): Hit 160

QUXKUQ

Reference: M.T.M.Al-Dajani, H.H.Abdallah, N.Mohamed, M.Hemamalini, H.-K.Fun (2010) *Acta Crystallogr.,Sect.E:Struct.Rep.Online* ,**66,**m774

Formula: $C_8 H_{14} Cu_1 O_{14}$,2($H_2 O_1$)

Compound Name: Diaqua-bis(hydrogen tartrato)-copper(ii) dihydrate

Space Group:	P21/c	***Cell:***	***a*** 7.158(0)	***b*** 14.099(1)	***c*** 7.891(0)
Space Group No.:	14	***(Å,°)***	α 90.00	β 109.14(0)	γ 90.00

R-Factor (%): 2.31 ***Temperature(K):*** 100 ***Density(g/cm³):*** 1.915

Parameters

Fragment 1

**DIST1 (D)**	1.933
**DIST2 (D)**	1.964
**DIST3 (D)**	1.964
**DIST4 (D)**	1.933
**DIST5 (D)**	2.465
**DIST6 (D)**	2.465

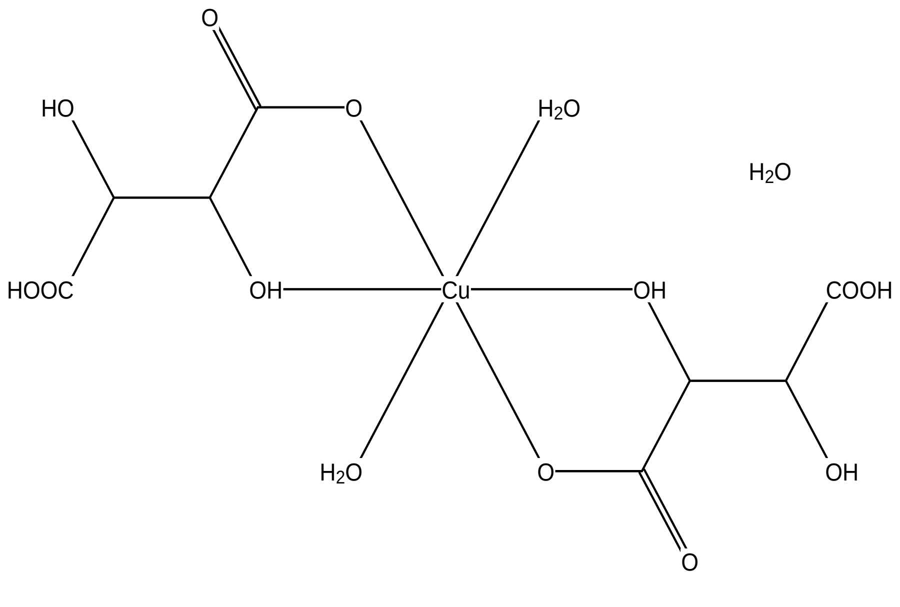

Search: search2 (Wed Jul 26 09:11:25 2017): Hit 161

RAGXAA

Reference:	E.E.Martsinko, A.G.Pesaroglo, L.Kh.Minacheva, I.I.Seifullina, V.S.Sergienko, A.V.Churakov (2011) *Zh.Neorg.Khim.(Russ.)(Russ.J.Inorg.Chem.)* ,**56,**228
Formula:	$H_{12}\,Cu_1\,O_6\,^{2+}$,$C_{10}\,H_{14}\,Cu_2\,Ge_1\,O_{18}\,^{2-}$,4($H_2\,O_1$)
Compound Name:	Hexa-aqua-copper(ii) bis(μ_3-triolatoglutarato)-tetra-aqua-di-copper(ii)-germanium(iv) tetrahydrate

Space Group:	P21/n	***Cell:***	***a*** 10.216(2)	***b*** 12.272(3)	***c*** 10.679(2)
Space Group No.:	14	***(Å,°)***	α 90.00	β 93.13(3)	γ 90.00

R-Factor (%): 2.61 ***Temperature(K):*** 100 ***Density(g/cm³):*** 2.150

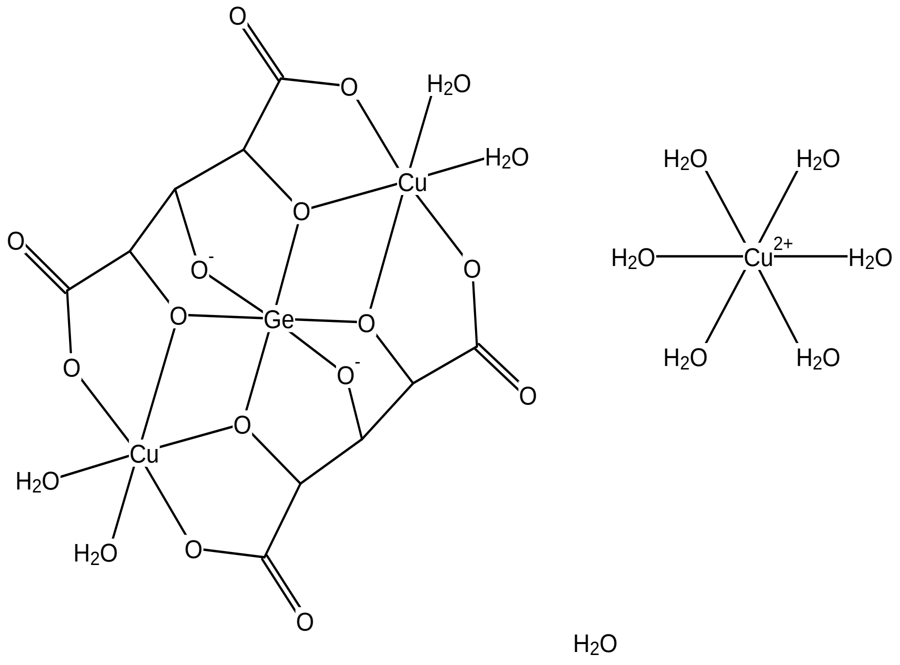

Parameters

Fragment 1

**DIST1 (D)**	1.948
**DIST2 (D)**	1.938
**DIST3 (D)**	1.948
**DIST4 (D)**	1.938
**DIST5 (D)**	2.515
**DIST6 (D)**	2.515

Fragment 2

**DIST1 (D)**	1.984
**DIST2 (D)**	1.950
**DIST3 (D)**	2.004
**DIST4 (D)**	1.976
**DIST5 (D)**	2.481
**DIST6 (D)**	2.364

Search: search2 (Wed Jul 26 09:11:25 2017): Hit 162

RELCUG01

Reference:	J.Bebendorf, H.-B.Burgi, E.Gamp, M.A.Hitchman, A.Murphy, D.Reinen, M.J.Riley, H.Stratemeier (1996) *Inorg.Chem.* ,**35,** 7419
Formula:	$C_{12} H_{24} Cu_1 O_6{}^{2+}$,2($C_7 H_7 O_3 S_1{}^{1-}$)
Compound Name:	bis(1,3,5-Trihydroxycyclohexane)-copper(ii) ditosylate

Space Group:	P-1	***Cell:***	***a***	6.385(4)	***b***	9.401(7)	***c***	12.410(9)
Space Group No.:	2	***(Å,°)***	α	76.82(6)	β	87.68(5)	γ	77.06(6)

R-Factor (%): 2.60 ***Temperature(K):*** 93 ***Density(g/cm³):*** 1.575

Parameters

Fragment 1

**DIST1 (D)**	1.968
**DIST2 (D)**	2.022
**DIST3 (D)**	1.968
**DIST4 (D)**	2.022
**DIST5 (D)**	2.223
**DIST6 (D)**	2.223

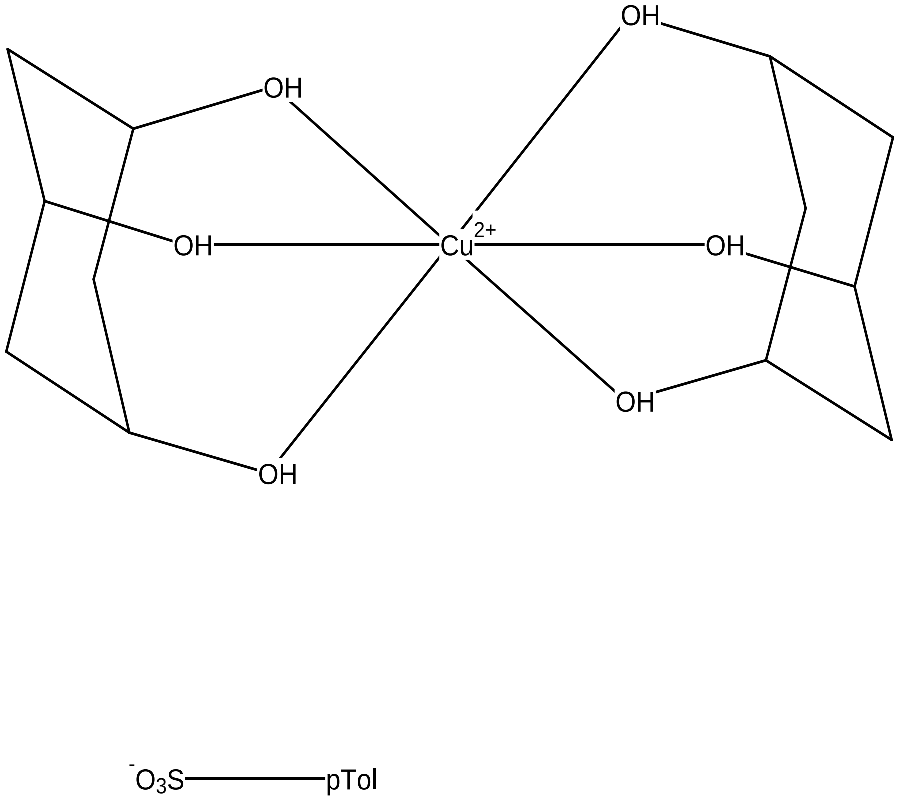

Search: search2 (Wed Jul 26 09:11:25 2017): Hit 163

ROLCEC

Reference:	Ran Shang, Sa Chen, Zhe-Ming Wang, Song Gao (2014)<br>*Chem.-Eur.J.* ,**20,**15872
Formula:	$(C_3 H_3 Cu_1 O_6{}^{1-})n,H_4 N_1{}^{1+}$
Compound Name:	catena-(Ammonium tris(μ-formato)-copper)

Space Group:	P212121	***Cell:***	***a*** 7.020(0)	***b*** 13.369(0)	***c*** 8.068(0)		
Space Group No.:	19	***(Å,°)***	α 90.00	β 90.00	γ 90.00		
R-Factor (%):	2.59	***Temperature(K):***	94	***Density(g/cm³):***	1.900		

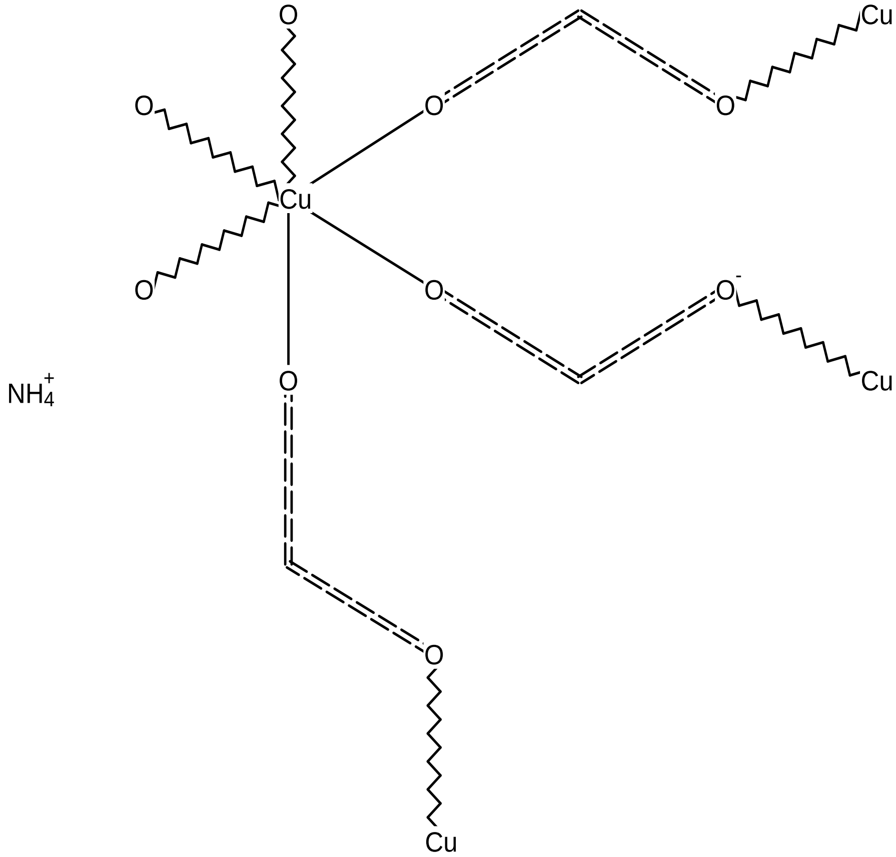

Parameters

Fragment 1

**DIST1 (D)**	1.956
**DIST2 (D)**	1.983
**DIST3 (D)**	1.989
**DIST4 (D)**	1.975
**DIST5 (D)**	2.371
**DIST6 (D)**	2.386

Search: search2 (Wed Jul 26 09:11:25 2017): Hit 164

ROLCEC01

Reference: Ran Shang, Sa Chen, Zhe-Ming Wang, Song Gao (2014)
Chem.-Eur.J. ,**20,**15872

Formula: $(C_3 H_3 Cu_1 O_6{}^{1-})n,H_4 N_1{}^{1+}$

Compound Name: catena-(Ammonium tris(μ-formato)-copper)

Space Group:	P212121	***Cell:***	***a***	7.030(0)	***b***	13.349(0)	***c***	8.077(0)
Space Group No.:	19	***(Å,°)***	α	90.00	β	90.00	γ	90.00

R-Factor (%): 2.46 ***Temperature(K):*** 140 ***Density(g/cm³):*** 1.898

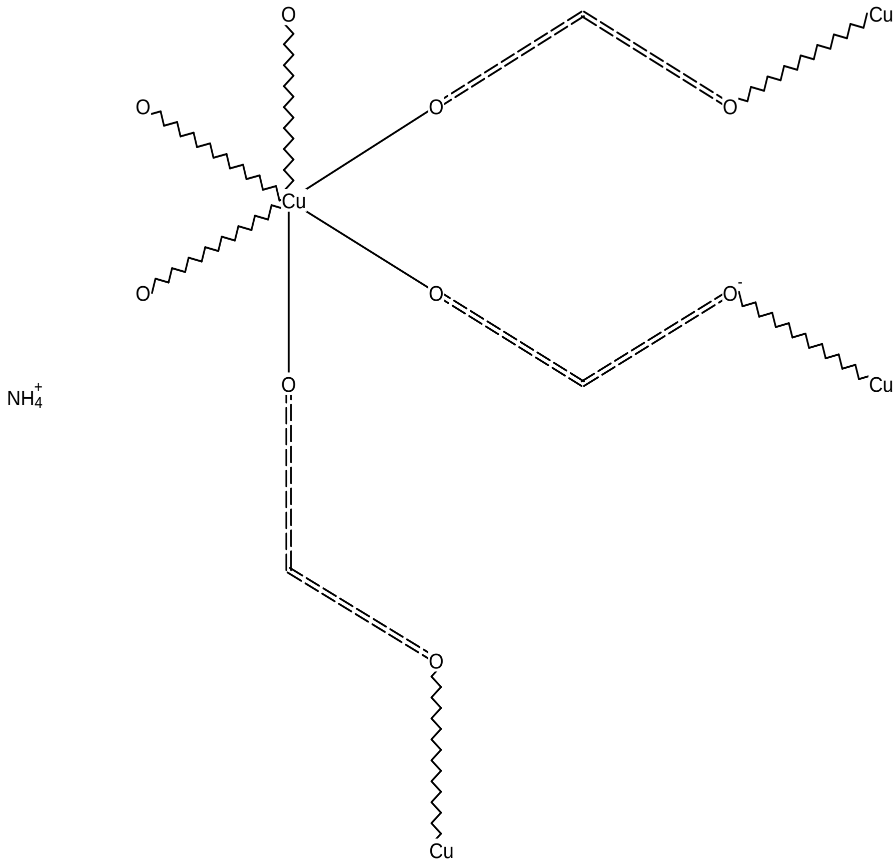

Parameters

Fragment 1

**DIST1 (D)**	1.959
**DIST2 (D)**	1.977
**DIST3 (D)**	1.987
**DIST4 (D)**	1.977
**DIST5 (D)**	2.377
**DIST6 (D)**	2.391

Search: search2 (Wed Jul 26 09:11:25 2017): Hit 165

ROLCEC02

Reference:	Ran Shang, Sa Chen, Zhe-Ming Wang, Song Gao (2014)<br>*Chem.-Eur.J.* ,**20,**15872
Formula:	$(C_3 H_3 Cu_1 O_6{}^{1-})n,H_4 N_1{}^{1+}$
Compound Name:	catena-(Ammonium tris(μ-formato)-copper)

Space Group:	P212121	***Cell:***	***a*** 7.046(0)	***b*** 13.301(0)	***c*** 8.085(0)		
Space Group No.:	19	***(Å,°)***	α 90.00	β 90.00	γ 90.00		
R-Factor (%):	2.74	***Temperature(K):***	180	***Density(g/cm^3):***	1.899		

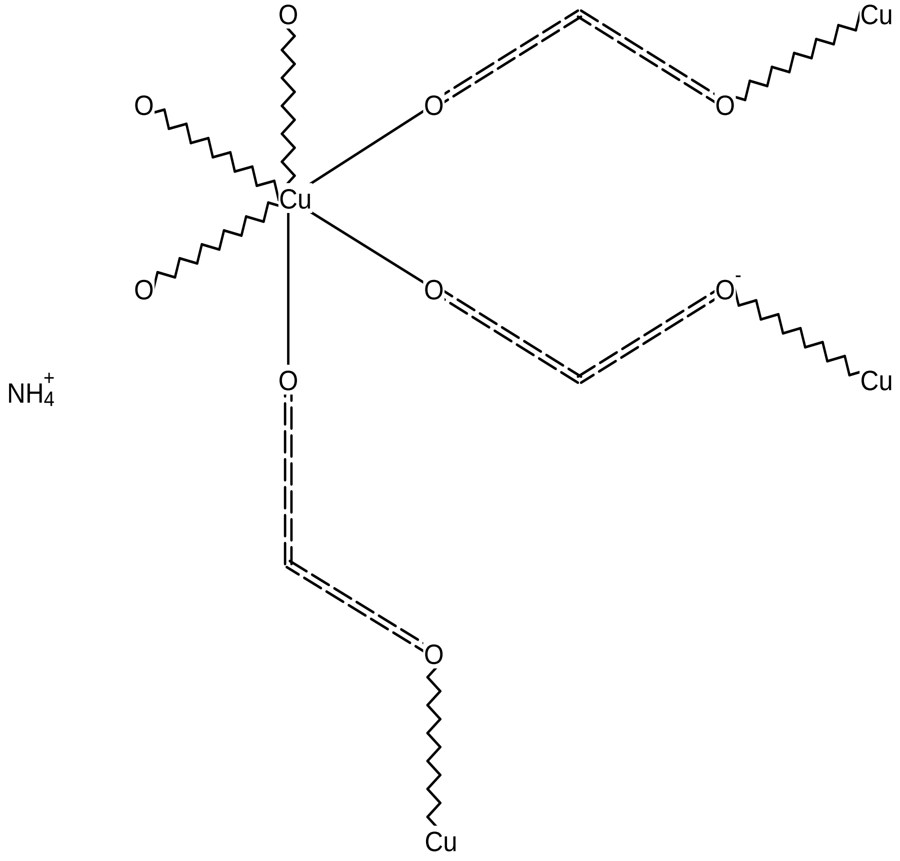

Parameters

Fragment 1

**DIST1 (D)**	1.960
**DIST2 (D)**	1.976
**DIST3 (D)**	1.986
**DIST4 (D)**	1.979
**DIST5 (D)**	2.380
**DIST6 (D)**	2.398

Search: search2 (Wed Jul 26 09:11:25 2017): Hit 166

ROLCEC03

Reference:	Ran Shang, Sa Chen, Zhe-Ming Wang, Song Gao (2014) *Chem.-Eur.J.* ,**20,**15872
Formula:	$(C_3\ H_3\ Cu_1\ O_6\ ^{1-})n,H_4\ N_1\ ^{1+}$
Compound Name:	catena-(Ammonium tris(μ-formato)-copper)

Space Group:	P212121	***Cell:***	***a*** 7.065(0)	***b*** 13.247(0)	***c*** 8.103(0)		
Space Group No.:	19	***(Å,°)***	α 90.00	β 90.00	γ 90.00		
R-Factor (%):	2.59	***Temperature(K):***	200	***Density(g/cm3):***	1.897		

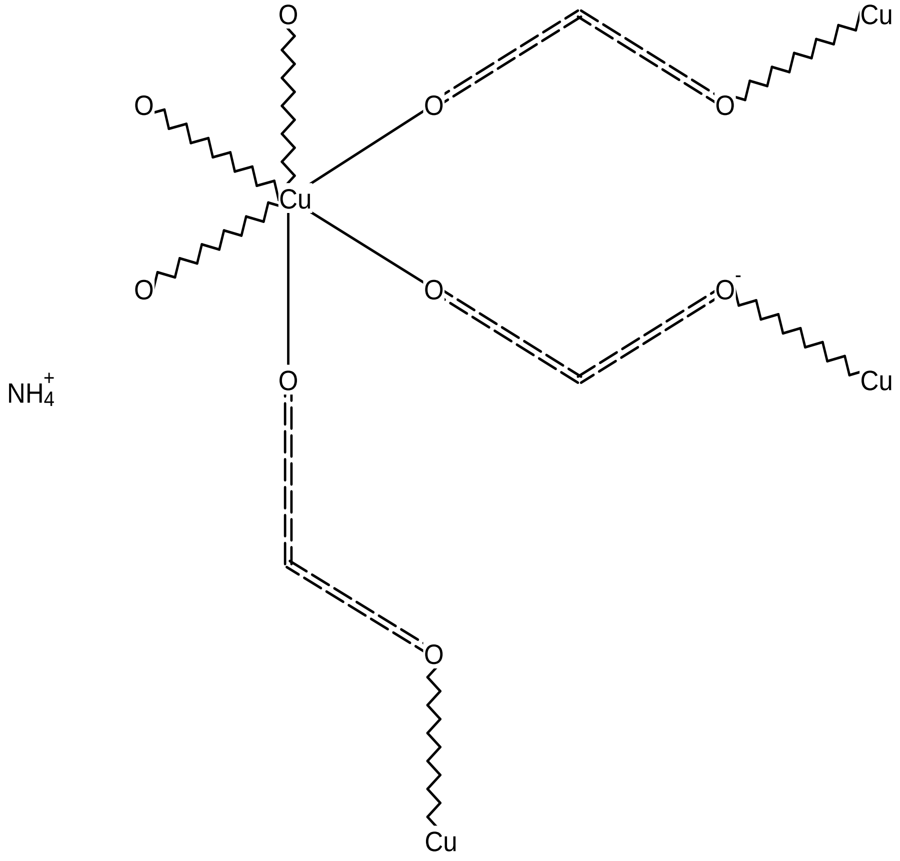

Parameters

Fragment 1

**DIST1 (D)**	1.963
**DIST2 (D)**	1.974
**DIST3 (D)**	1.987
**DIST4 (D)**	1.983
**DIST5 (D)**	2.386
**DIST6 (D)**	2.401

Search: search2 (Wed Jul 26 09:11:25 2017): Hit 167

ROLCEC04

Reference: Ran Shang, Sa Chen, Zhe-Ming Wang, Song Gao (2014)
Chem.-Eur.J. ,**20,**15872

Formula: $(C_3 H_3 Cu_1 O_6{}^{1-})n,H_4 N_1{}^{1+}$

Compound Name: catena-(Ammonium tris(μ-formato)-copper)

Space Group:	P212121	***Cell:***	***a***	7.095(0)	***b***	13.148(0)	***c***	8.123(0)
Space Group No.:	19	***(Å,°)***	α	90.00	β	90.00	γ	90.00

R-Factor (%): 2.64 ***Temperature(K):*** 220 ***Density(g/cm³):*** 1.899

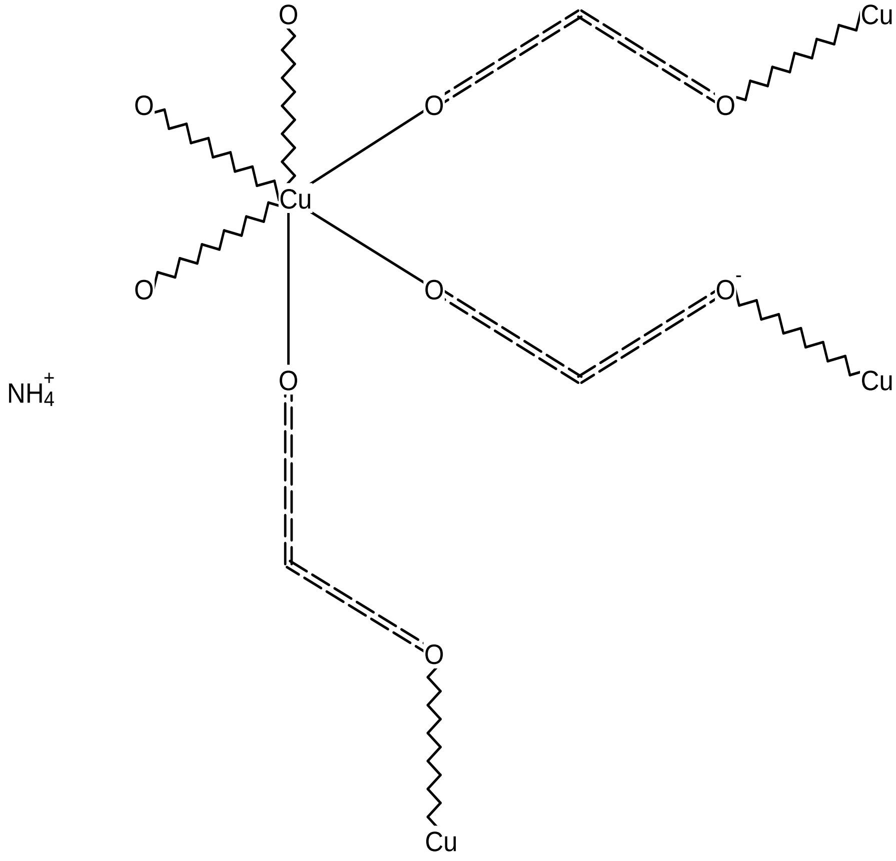

Parameters

Fragment 1

**DIST1 (D)**	1.962
**DIST2 (D)**	1.969
**DIST3 (D)**	1.983
**DIST4 (D)**	1.983
**DIST5 (D)**	2.385
**DIST6 (D)**	2.409

Search: search2 (Wed Jul 26 09:11:25 2017): Hit 168

ROLCEC05

Reference: Ran Shang, Sa Chen, Zhe-Ming Wang, Song Gao (2014)
Chem.-Eur.J. ,**20,**15872

Formula: $(C_3\ H_3\ Cu_1\ O_6\ ^{1-})n,H_4\ N_1\ ^{1+}$

Compound Name: catena-(Ammonium tris(μ-formato)-copper)

Space Group:	P212121	***Cell:***	***a*** 7.128(0)	***b*** 13.050(0)	***c*** 8.141(0)
Space Group No.:	19	***(Å,°)***	α 90.00	β 90.00	γ 90.00

R-Factor (%): 2.70 ***Temperature(K):*** 240 ***Density(g/cm³):*** 1.900

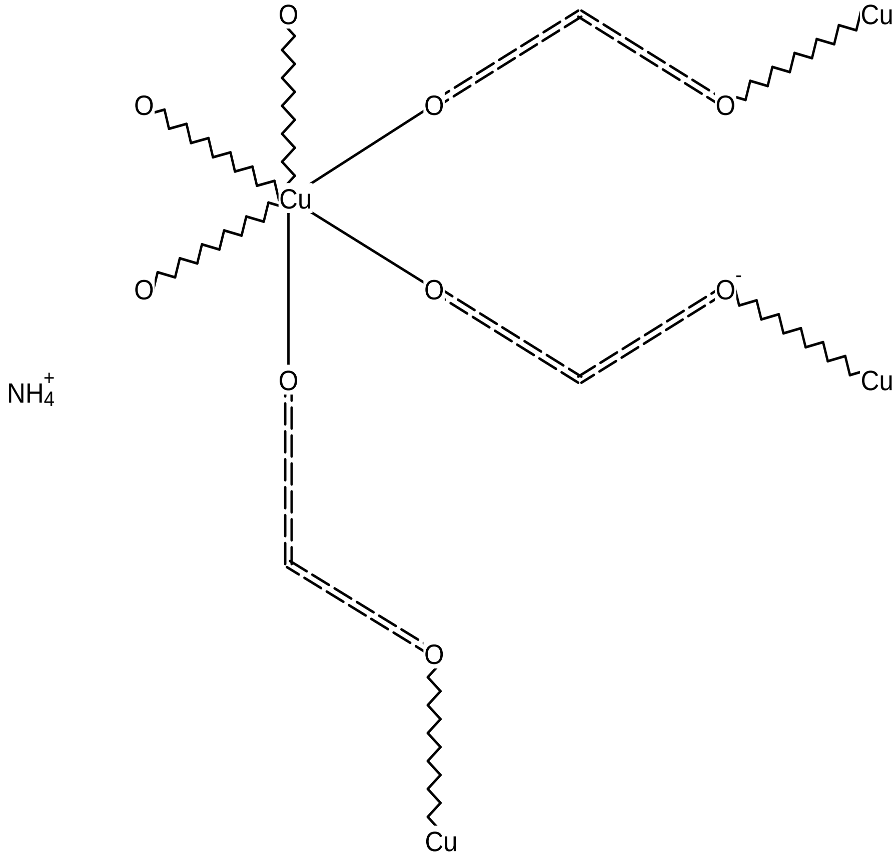

Parameters

Fragment 1

**DIST1 (D)**	1.964
**DIST2 (D)**	1.964
**DIST3 (D)**	1.983
**DIST4 (D)**	1.987
**DIST5 (D)**	2.392
**DIST6 (D)**	2.416

Search: search2 (Wed Jul 26 09:11:25 2017): Hit 169

ROLCEC06

Reference:	Ran Shang, Sa Chen, Zhe-Ming Wang, Song Gao (2014)<br>*Chem.-Eur.J.* ,**20,**15872
Formula:	$(C_3 H_3 Cu_1 O_6 {}^{1-})n,H_4 N_1 {}^{1+}$
Compound Name:	catena-(Ammonium tris(μ-formato)-copper)

Space Group:	P212121	***Cell:***	***a*** 7.151(0)	***b*** 13.000(0)	***c*** 8.145(0)		
Space Group No.:	19	***(Å,°)***	α 90.00	β 90.00	γ 90.00		
R-Factor (%):	2.81	***Temperature(K):***	260	***Density(g/cm³):***	1.901		

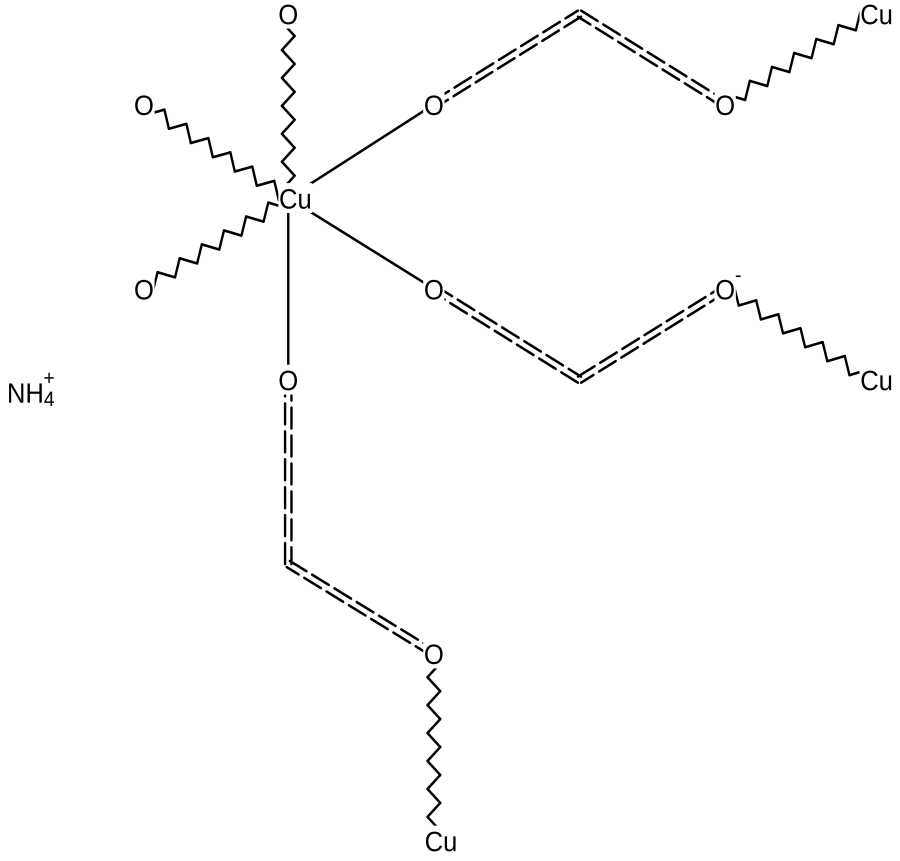

Parameters

Fragment 1

**DIST1 (D)**	1.961
**DIST2 (D)**	1.965
**DIST3 (D)**	1.981
**DIST4 (D)**	1.985
**DIST5 (D)**	2.391
**DIST6 (D)**	2.417

Search: search2 (Wed Jul 26 09:11:25 2017): Hit 170

ROLCEC07

Reference: Ran Shang, Sa Chen, Zhe-Ming Wang, Song Gao (2014)
Chem.-Eur.J. ,**20,**15872

Formula: $(C_3 H_3 Cu_1 O_6{}^{1-})n,H_4 N_1{}^{1+}$

Compound Name: catena-(Ammonium tris(μ-formato)-copper)

Space Group:	P212121	***Cell:***	***a***	7.171(0)	***b***	12.964(0)	***c***	8.143(0)
Space Group No.:	19	***(Å,°)***	α	90.00	β	90.00	γ	90.00

R-Factor (%): 2.83 ***Temperature(K):*** 290 ***Density(g/cm³):*** 1.901

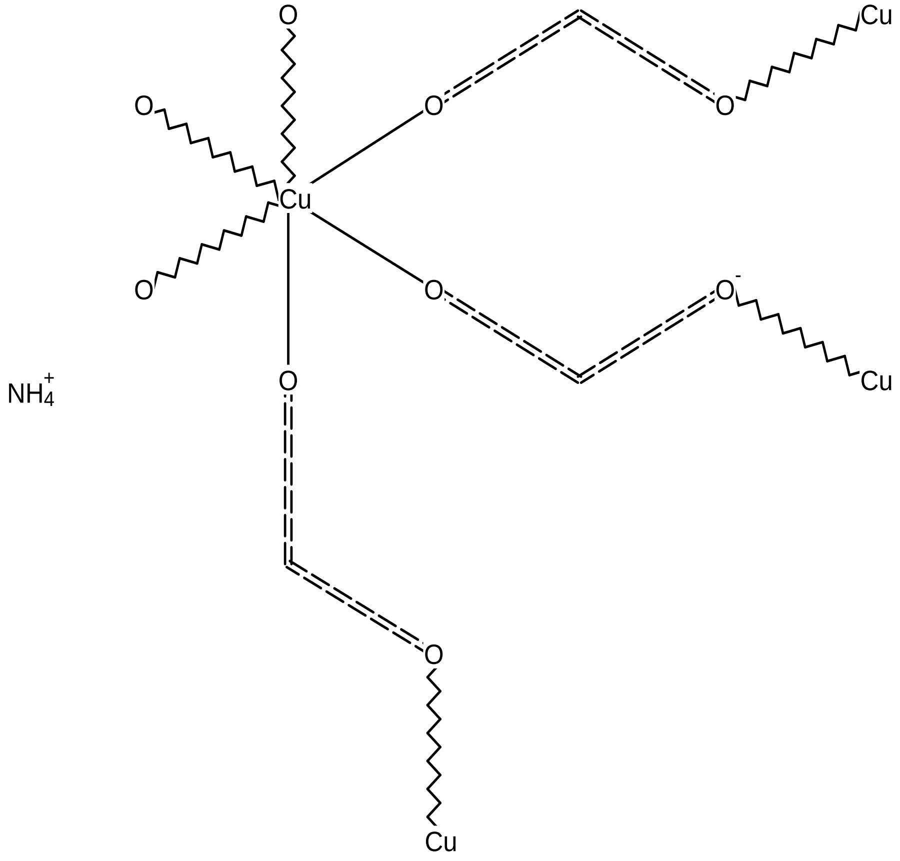

Parameters

Fragment 1

**DIST1 (D)**	1.964
**DIST2 (D)**	1.963
**DIST3 (D)**	1.983
**DIST4 (D)**	1.987
**DIST5 (D)**	2.399
**DIST6 (D)**	2.419

Search: search2 (Wed Jul 26 09:11:25 2017): Hit 171

ROLSAN

Reference: Zhiqin Ji, Shaopeng Wei, Wenjun Wu (2009)
Acta Crystallogr.,Sect.E:Struct.Rep.Online ,**65,**m182

Formula: C_{16} H_{28} Cu_1 N_2 O_{10}

Compound Name: bis(4-Ammonio-4-methylpentan-2-one)-bis(oxalato-O,O')-copper(ii)

Space Group:	C2/m	***Cell:***	***a***	13.639(3)	***b***	7.975(1)	***c***	10.958(2)
Space Group No.:	12	***(Å,°)***	α	90.00	β	113.27(3)	γ	90.00

R-Factor (%): 2.62 ***Temperature(K):*** 113 ***Density(g/cm^3):*** 1.431

Parameters

Fragment 1

**DIST1 (D)**	1.938
**DIST2 (D)**	1.938
**DIST3 (D)**	1.938
**DIST4 (D)**	1.938
**DIST5 (D)**	2.663
**DIST6 (D)**	2.663

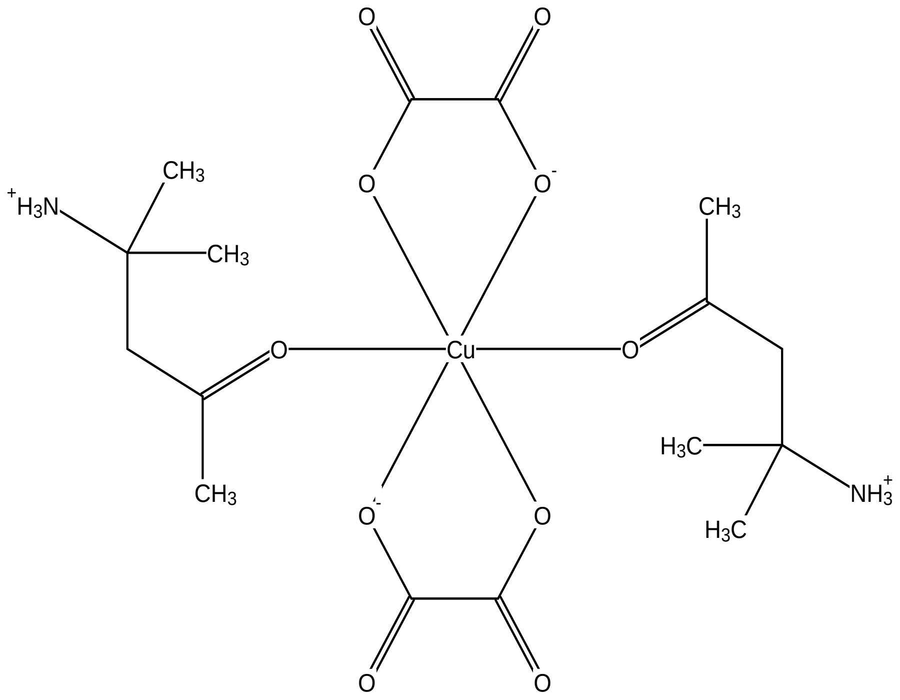

Search: search2 (Wed Jul 26 09:11:25 2017): Hit 172

RUSPEB

Reference: Xiao-Hua Xie, Bai-Mu Wang, S.W.Ng (2010)
Acta Crystallogr.,Sect.E:Struct.Rep.Online ,**66,**m271

Formula: $C_{12} H_{32} Cu_2 N_2 O_{27} Ti_2$,7($H_2 O_1$)

Compound Name: bis(μ_2-Nitrilotriacetato)-(μ_2-oxo)-bis(peroxo-O,O')-deca-aqua-di-copper(ii)-di-titanium(iv) heptahydrate

Space Group:	C2/c	***Cell:***	***a*** 14.931(1)	***b*** 13.289(0)	***c*** 17.445(1)
Space Group No.:	15	***(Å,°)***	α 90.00	β 100.83(0)	γ 90.00

R-Factor (%): 2.58 ***Temperature(K):*** 293 ***Density(g/cm³):*** 1.925

Parameters

Fragment 1

**DIST1 (D)**	1.986
**DIST2 (D)**	2.016
**DIST3 (D)**	1.964
**DIST4 (D)**	1.931
**DIST5 (D)**	2.269
**DIST6 (D)**	2.446

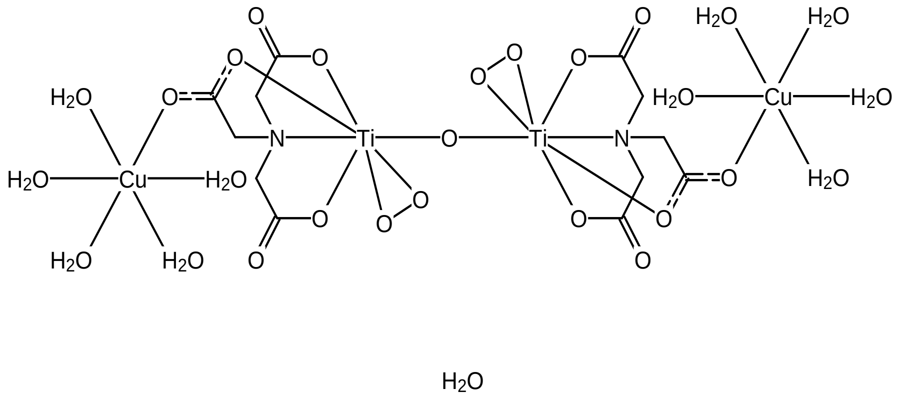

Search: search2 (Wed Jul 26 09:11:25 2017): Hit 173

RUWMUR03

Reference:	D.Ghoshal, T.K.Maji, T.Mallah, Tian-Huey Lu, G.Mostafa, N.R.Chaudhuri (2005) *Inorg.Chim.Acta* ,**358,**1027
Formula:	$(C_6 H_8 Cu_1 Na_2 O_{10})n$
Compound Name:	catena-(bis(μ_6-Malonato)-bis(μ_2-aqua)-copper(ii)-di-sodium)

Space Group:	Pbca	***Cell:***	***a*** 6.827(0)	***b*** 9.512(1)	***c*** 16.399(2)
Space Group No.:	61	***(Å,°)***	α 90.00	β 90.00	γ 90.00
R-Factor (%):	2.28	***Temperature(K):***	296	***Density(g/cm^3):***	2.181

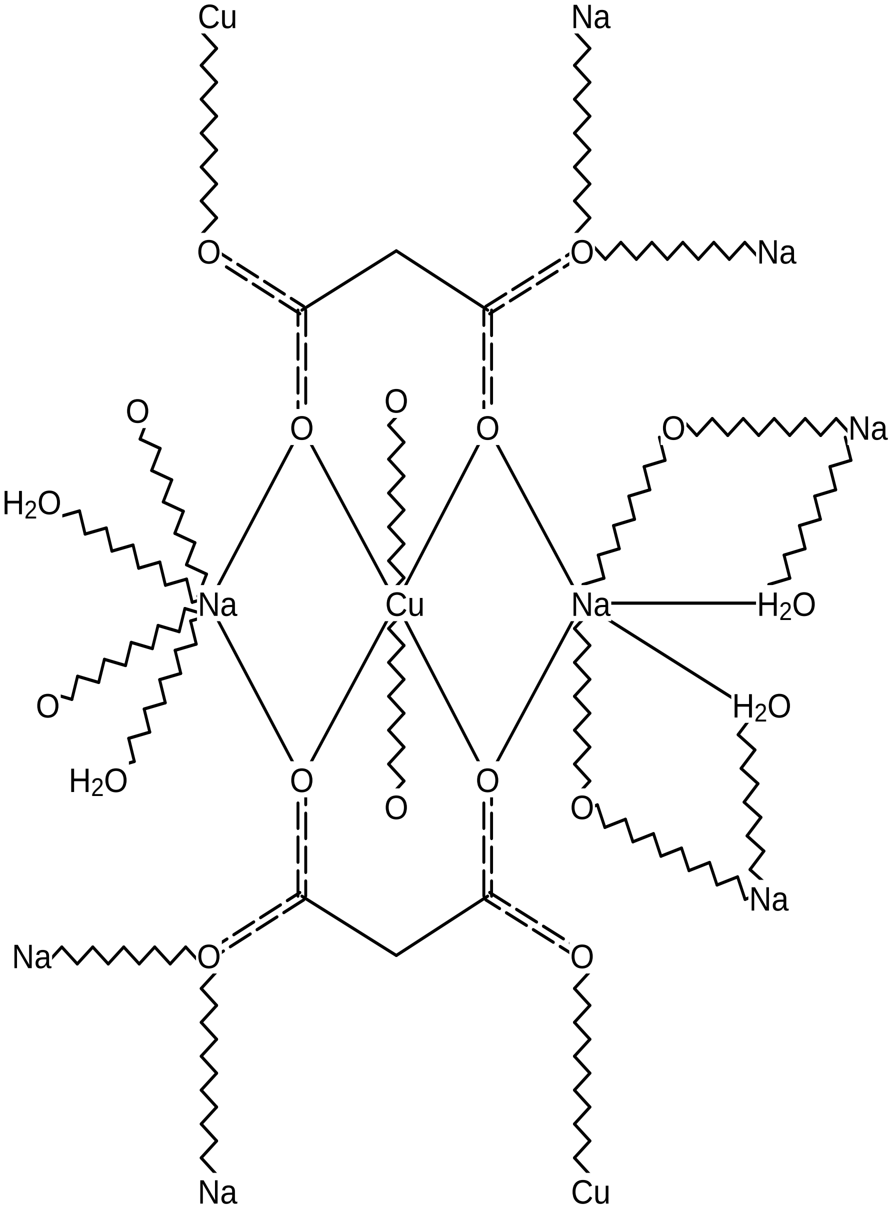

Parameters

Fragment 1

**DIST1 (D)**	1.944
**DIST2 (D)**	1.953
**DIST3 (D)**	1.944
**DIST4 (D)**	1.953
**DIST5 (D)**	2.602
**DIST6 (D)**	2.602

Search: search2 (Wed Jul 26 09:11:25 2017): Hit 174

SANMAX

Reference: Meng-Yuan Li, M.Kurmoo, Zhe-Ming Wang, Song Gao
(2011) *Chem.Asian J.* ,**6,**3084

Formula: $(C_4 H_{14} N_2{}^{2+})$n,n$(C_6 H_6 Cu_2 O_{12}{}^{2-})$

Compound Name: catena-[1,2-bis(Methylammonio)ethane hexakis(μ_2-formato-O,O')-copper(ii)]

Space Group:	C2/c	***Cell:***	***a*** 8.661(0)	***b***	13.640(0)	***c***	14.583(0)
Space Group No.:	15	***(Å,°)***	α 90.00	β	90.88(0)	γ	90.00

R-Factor (%): 2.70 ***Temperature(K):*** 293 ***Density(g/cm^3):*** 1.879

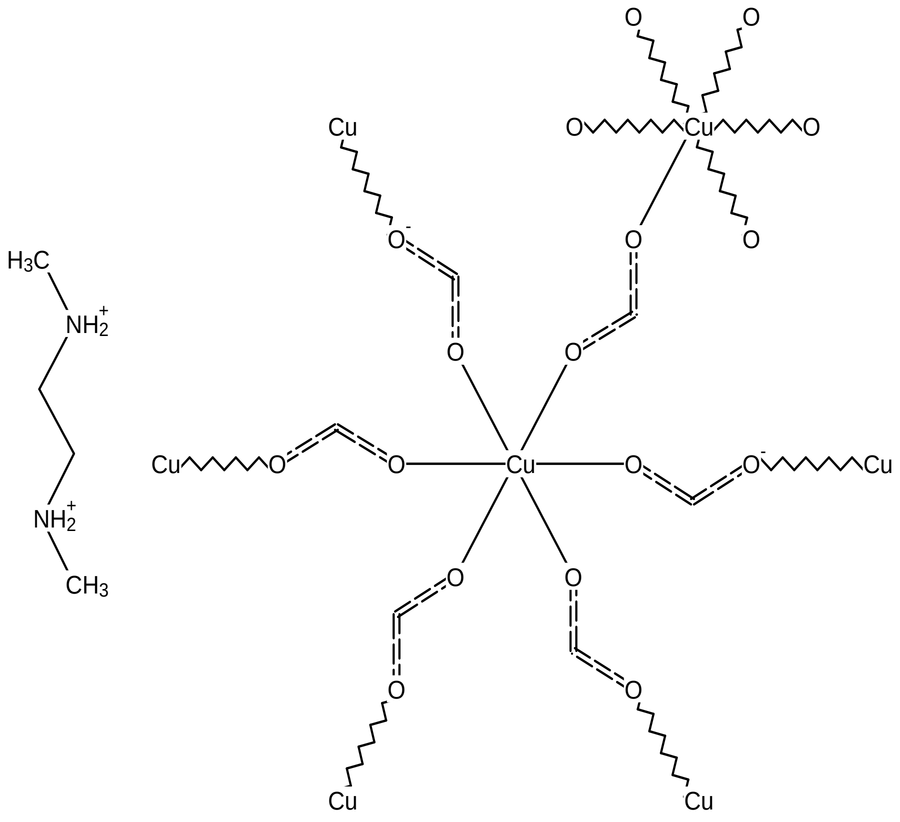

Parameters

Fragment 1

**DIST1 (D)**	1.975
**DIST2 (D)**	1.980
**DIST3 (D)**	1.975
**DIST4 (D)**	1.980
**DIST5 (D)**	2.414
**DIST6 (D)**	2.414

Fragment 2

**DIST1 (D)**	1.964
**DIST2 (D)**	1.961
**DIST3 (D)**	1.961
**DIST4 (D)**	1.964
**DIST5 (D)**	2.517
**DIST6 (D)**	2.517

Search: search2 (Wed Jul 26 09:11:25 2017): Hit 175

SAVTEP

Reference:	V.Shivaiah, S.K.Das (2005) *Inorg.Chem.* ,**44,**7313
Formula:	$C_{24}\ H_{40}\ Cu_{1}\ O_{12}\ ^{2+}$,$Mo_{6}\ O_{19}\ ^{2-}$
Compound Name:	(Dibenzo-24-crown-8)-tetraaqua-copper(ii) (μ_6-oxo)-dodecakis(μ_2-oxo)-hexaoxo-hexa-molybdenum(vi)

Space Group:	P-1	***Cell:***	***a*** 9.329(0)	***b*** 10.878(0)	***c*** 12.249(0)
Space Group No.:	2	***(Å,°)***	α 101.63(0)	β 107.70(0)	γ 111.91(0)

R-Factor (%): 2.41 ***Temperature(K):*** 298 ***Density(g/cm³):*** 2.368

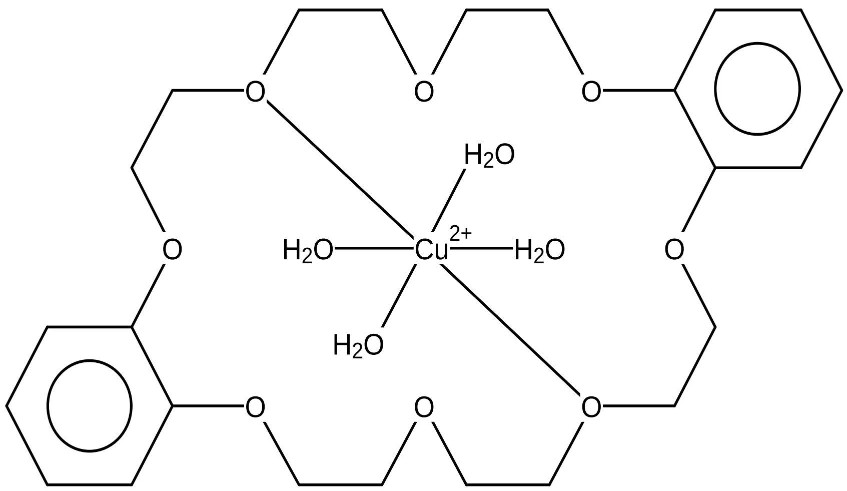

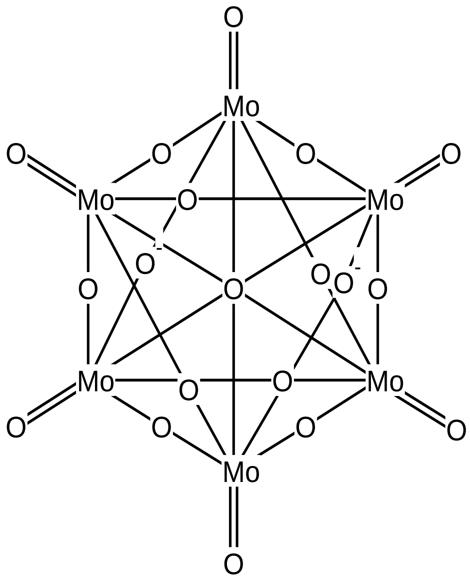

Parameters

Fragment 1

**DIST1 (D)**	1.940
**DIST2 (D)**	1.973
**DIST3 (D)**	1.940
**DIST4 (D)**	1.973
**DIST5 (D)**	2.383
**DIST6 (D)**	2.383

Search: search2 (Wed Jul 26 09:11:25 2017): Hit 176

SOHTUF

Reference: V.I.Ovcharenko, G.V.Romanenko, K.Yu.Maryunina, A.S.Bogomyakov, E.V.Gorelik (2008) *Inorg.Chem.* ,**47,**9537

Formula: $(C_{48} H_{50} Cu_2 F_{24} N_8 O_{12})n,n(C_5 H_{11} I_1)$

Compound Name: catena-(bis(μ_2-4,4,5,5-Tetramethyl-2-(1-butylpyrazole-4-yl)-4,5-dihydro-1H-imidazol-3-oxide-1-oxyl)-tetrakis(1,1,1,5,5,5-hexafluoropentan-2,4-dionato)-di-copper(ii) iodopentane solvate)

Space Group:	P-1	***Cell:***	***a*** 10.632(1)	***b*** 13.265(1)	***c*** 13.309(1)
Space Group No.:	2	***(Å,°)***	α 98.58(0)	β 108.68(0)	γ 98.25(0)

R-Factor (%): 2.71 ***Temperature(K):*** 100 ***Density(g/cm^3):*** 1.652

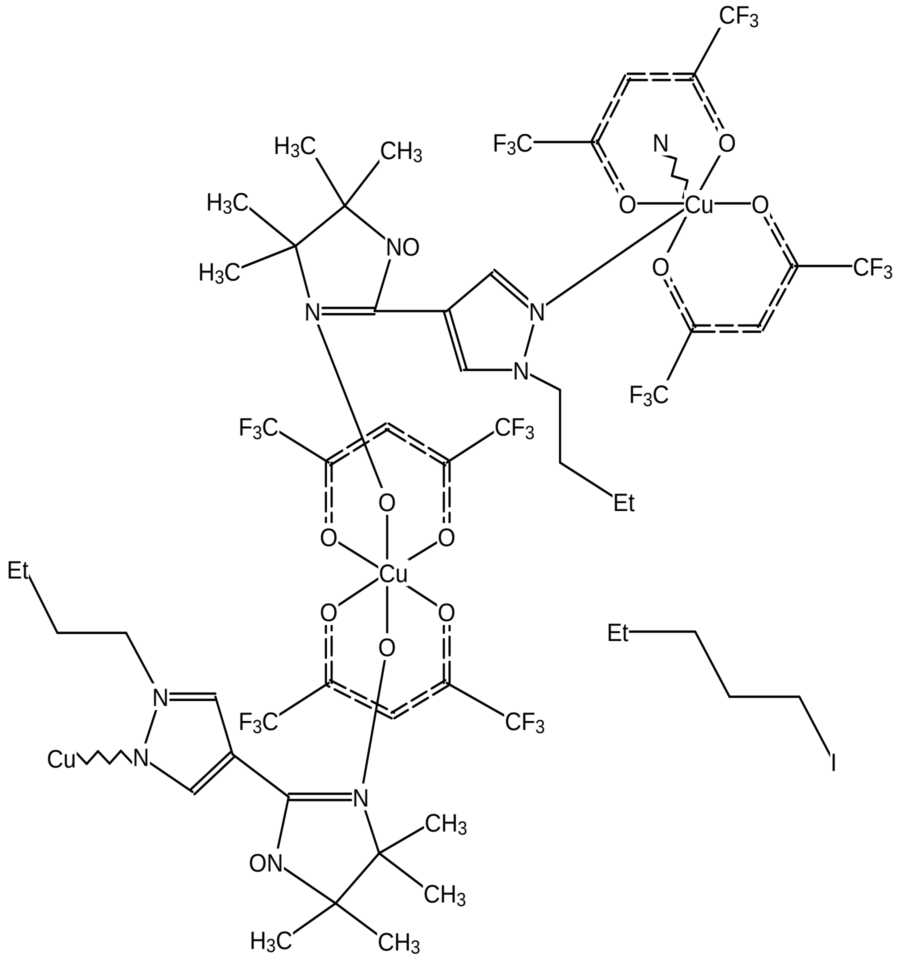

Parameters

Fragment 1

**DIST1 (D)**	1.968
**DIST2 (D)**	1.965
**DIST3 (D)**	1.968
**DIST4 (D)**	1.965
**DIST5 (D)**	2.377
**DIST6 (D)**	2.377

Search: search2 (Wed Jul 26 09:11:25 2017): Hit 177

SOLDAZ

Reference:	A.Castineiras, I.Garcia-Santos, J.M.Gonzalez-Perez, J.Niclos-Gutierrez (2008) *CSD Communication(Private Communication) ,*
Formula:	$(C_5 H_6 N_5{}^{1+})2n,n(C_6 H_4 Cu_1 O_8{}^{2-}),2n(H_2 O_1)$
Compound Name:	catena-(bis(Adeninium) bis(μ_2-malonato)-copper dihydrate)

Space Group:	P-1	***Cell:***	***a***	5.060(0)	***b***	9.337(0)	***c***	11.267(0)
Space Group No.:	2	***(Å,°)***	α	92.65(0)	β	96.30(0)	γ	95.43(0)

R-Factor (%):	2.73	***Temperature(K):***	100	***Density(g/cm³):***	1.819

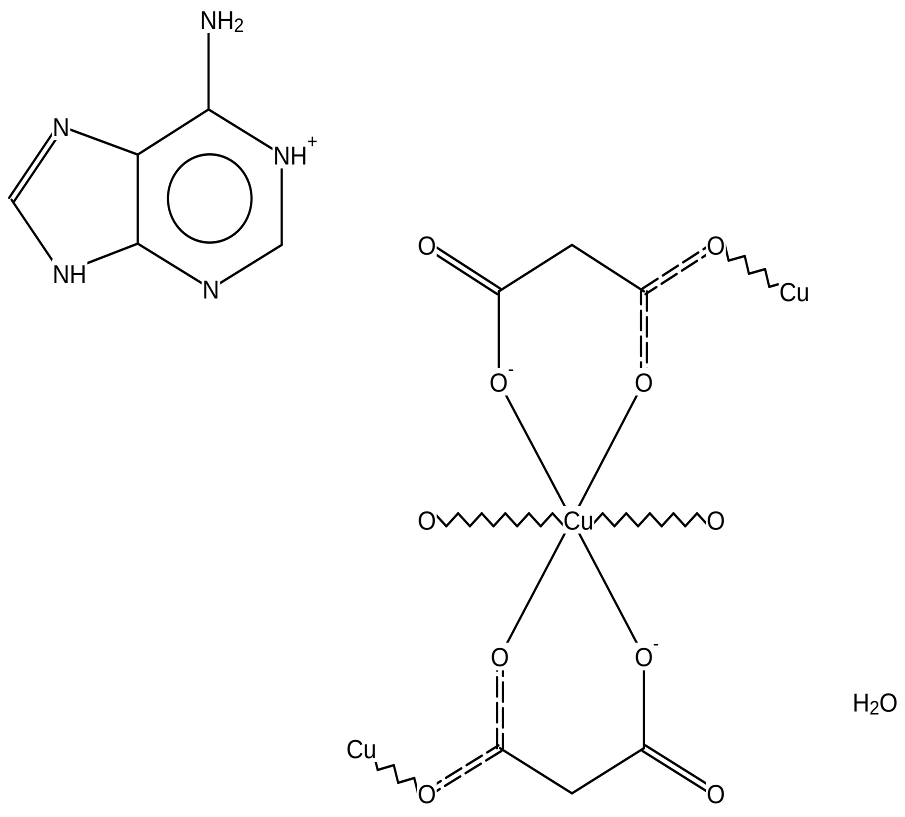

Parameters

Fragment 1

**DIST1 (D)**	1.926
**DIST2 (D)**	1.938
**DIST3 (D)**	1.938
**DIST4 (D)**	1.926
**DIST5 (D)**	2.608
**DIST6 (D)**	2.608

Search: search2 (Wed Jul 26 09:11:25 2017): Hit 178

SOPKOY

Reference: K.V.Yusenko, E.Yu.Filatov, D.B.Vasilchenko, I.A.Baidina, A.V.Zadesenez, Yu.V.Shubin (2007) *Z.Kristallogr.* ,**222,**289

Formula: $H_{12}\ N_4\ Pd_1{}^{2+}, C_4\ H_4\ Cu_1\ O_{10}{}^{2-}, H_2\ O_1$

Compound Name: Tetraammine-palladium diaqua-bis(ethanedioato-κ^{2}O^1,O^2)-copper(ii) monohydrate

Space Group:	I222	***Cell:***	***a***	6.718(0)	***b***	7.432(0)	***c***	14.968(0)
Space Group No.:	23	***(Å,°)***	α	90.00	β	90.00	γ	90.00

R-Factor (%): 2.00 ***Temperature(K):*** 293 ***Density(g/cm^3):*** 2.081

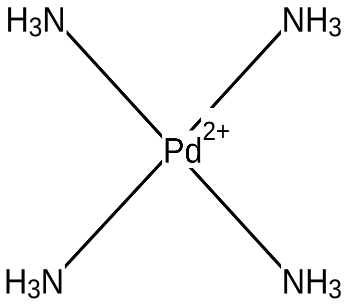

Parameters

Fragment 1

**DIST1 (D)**	1.939
**DIST2 (D)**	1.939
**DIST3 (D)**	1.939
**DIST4 (D)**	1.939
**DIST5 (D)**	2.357
**DIST6 (D)**	2.357

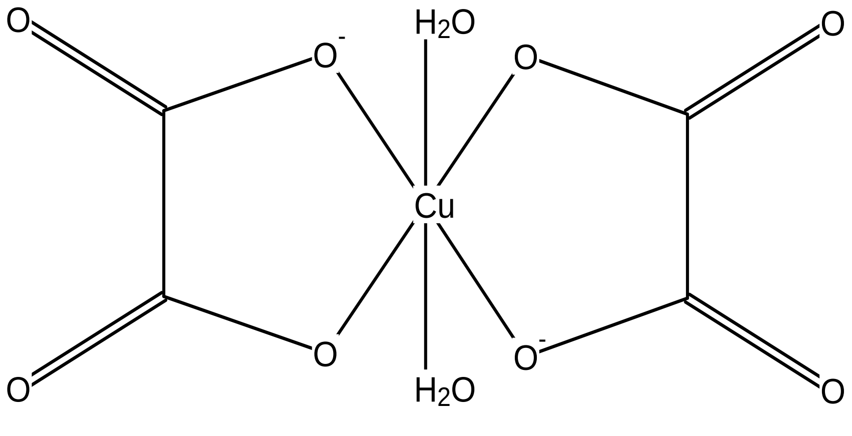

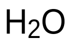

Search: search2 (Wed Jul 26 09:11:25 2017): Hit 179

SOPKUE

Reference:	K.V.Yusenko, E.Yu.Filatov, D.B.Vasilchenko, I.A.Baidina, A.V.Zadesenez, Yu.V.Shubin (2007) *Z.Kristallogr.* ,**222,**289
Formula:	$H_{12} N_4 Pt_1{}^{2+}, C_4 H_4 Cu_1 O_{10}{}^{2-}, H_2 O_1$
Compound Name:	Tetraammine-platinum diaqua-bis(ethanedioato-κ^{2}O^1,O^2)-copper(ii) monohydrate

Space Group:	I222	***Cell:***	***a*** 6.738(0)	***b*** 7.411(0)	***c*** 14.989(0)		
Space Group No.:	23	***(Å,°)***	α 90.00	β 90.00	γ 90.00		
R-Factor (%):	1.81	***Temperature(K):***	293	***Density(g/cm^3):***	2.471		

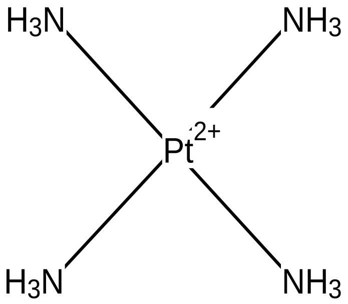

Parameters

Fragment 1

**DIST1 (D)**	1.946
**DIST2 (D)**	1.946
**DIST3 (D)**	1.946
**DIST4 (D)**	1.946
**DIST5 (D)**	2.260
**DIST6 (D)**	2.260

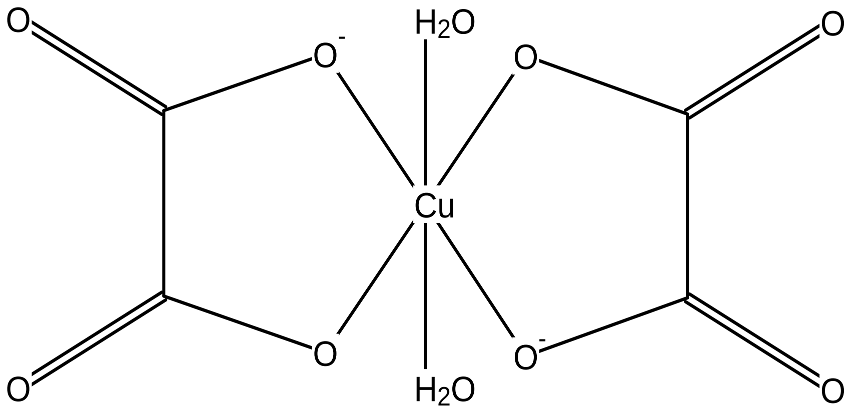

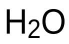

Search: search2 (Wed Jul 26 09:11:25 2017): Hit 180

TAMSCU10

Reference: F.Charbonnier, R.Faure, H.Loiseleur (1977)
Acta Crystallogr.,Sect.B:Struct.Crystallogr.Cryst.Chem. ,**33,**1845

Formula: $C_2 H_{14} Cu_1 O_{10} S_2$

Compound Name: Tetra-aqua-bis(methanesulfonato)-copper(ii)

Space Group:	P21/c	***Cell:***	***a***	9.306(0)	***b***	9.632(2)	***c***	7.308(0)
Space Group No.:	14	***(Å,°)***	α	90.00	β	122.18(1)	γ	90.00

R-Factor (%): 2.60 ***Temperature(K):*** 295 ***Density(g/cm³):*** 1.952

Parameters

Fragment 1

**DIST1 (D)**	1.969
**DIST2 (D)**	1.947
**DIST3 (D)**	1.969
**DIST4 (D)**	1.947
**DIST5 (D)**	2.387
**DIST6 (D)**	2.387

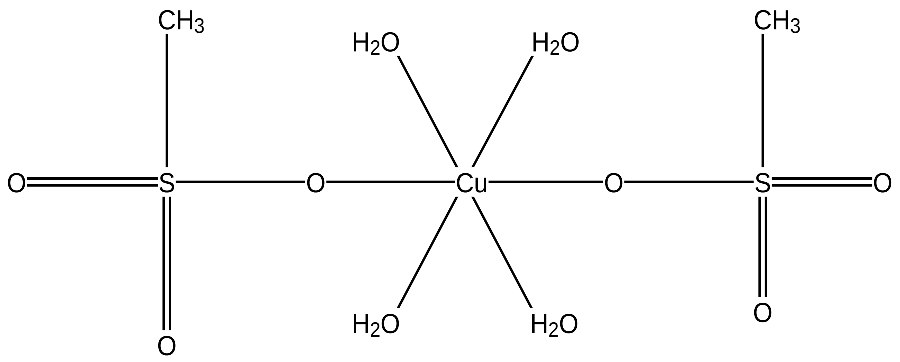

Search: search2 (Wed Jul 26 09:11:25 2017): Hit 181

TAYSAN

Reference:	D.Henschel, K.Linoh, K.-H.Nagel, A.Blaschette, P.G.Jones (1996) *Z.Anorg.Allg.Chem.* ,**622,**1065
Formula:	$C_4 H_{20} Cu_1 N_2 O_{12} S_4$
Compound Name:	Tetra-aqua-bis(mesylamido)-copper(ii)

Space Group:	P-1	***Cell:***	***a*** 7.298(4)	***b*** 7.913(4)	***c*** 8.392(3)		
Space Group No.:	2	***(Å,°)***	α 69.12(3)	β 74.19(3)	γ 64.96(3)		
R-Factor (%):	2.20	***Temperature(K):***	143	***Density(g/cm³):***	1.963		

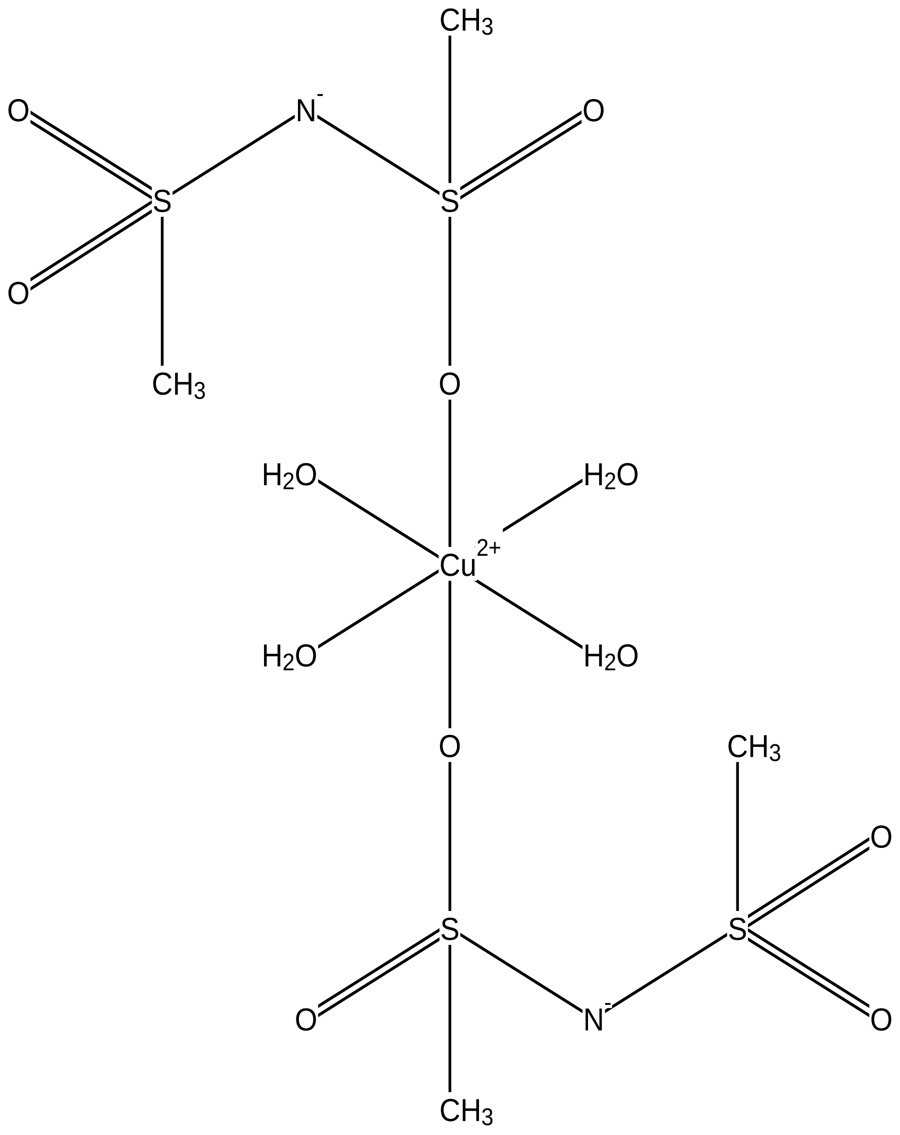

Parameters

Fragment 1

**DIST1 (D)**	1.959
**DIST2 (D)**	1.977
**DIST3 (D)**	1.959
**DIST4 (D)**	1.977
**DIST5 (D)**	2.326
**DIST6 (D)**	2.326

Search: search2 (Wed Jul 26 09:11:25 2017): Hit 182

TEHXEL

Reference: Yue-Ling Bai, Feifei Xing, Shourong Zhu, Jun Tao (2012) *Inorg.Chem.Commun.* ,**20,**50

Formula: $(C_{16} H_{28} Cu_1 O_{26} Tb_2)n,2n(H_2 O_1)$

Compound Name: catena-[bis(μ_3-D-Malato)-bis(μ_2-D-malato)-hexa-aqua-copper(ii)-di-terbium(iii) dihydrate]

Space Group:	C2	***Cell:***	***a*** 15.188(4)	***b*** 7.771(1)	***c*** 12.701(3)		
Space Group No.:	5	***(Å,°)***	α 90.00	β 113.99(0)	γ 90.00		

R-Factor (%): 2.51 ***Temperature(K):*** 298 ***Density(g/cm^3):*** 2.555

Parameters

Fragment 1

**DIST1 (D)**	1.960
**DIST2 (D)**	1.994
**DIST3 (D)**	1.960
**DIST4 (D)**	1.994
**DIST5 (D)**	2.435
**DIST6 (D)**	2.435

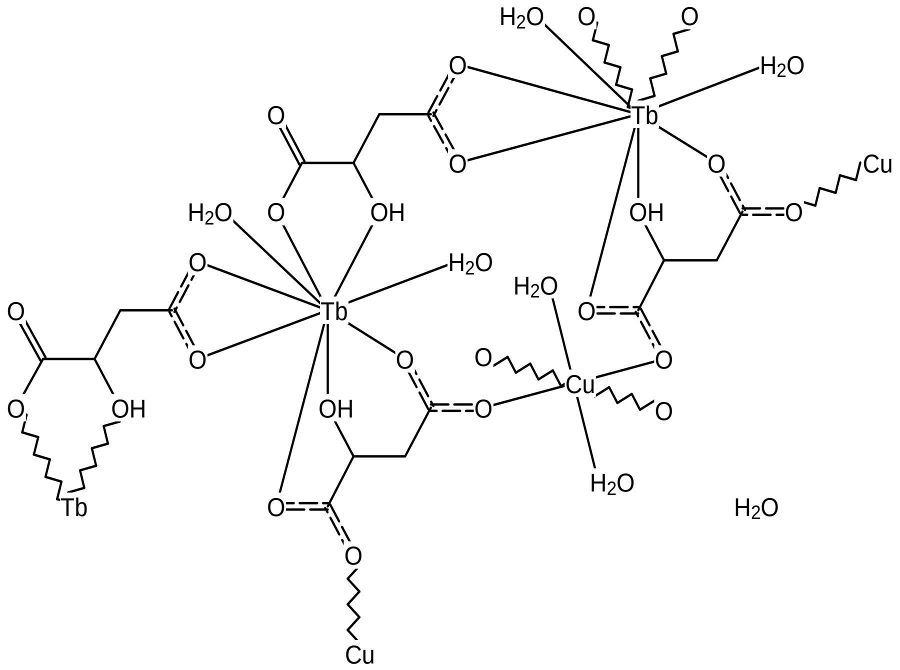

Search: search2 (Wed Jul 26 09:11:25 2017): Hit 183

TINPOV

Reference: S.Kawata, S.Kitagawa, H.Kumagai, C.Kudo, H.Kamesaki, T.Ishiyama, R.Suzuki, M.Kondo, M.Katada (1996) *Inorg.Chem.* ,**35,** 4449

Formula: $(C_{12} H_8 N_2{}^{2+})n,n(C_6 H_4 Cl_2 Cu_1 O_6{}^{2-})$

Compound Name: catena(Phenazinium (μ_2-3,6-dichloro-1,2,4,5-tetrahydroxyphenyl)-diaqua-copper(ii))

Space Group:	P-1	***Cell:***	***a***	8.071(0)	***b***	11.266(1)	***c***	4.990(0)
Space Group No.:	2	***(Å,°)***	α	97.80(1)	β	99.58(1)	γ	83.02(0)

R-Factor (%): 2.70 ***Temperature(K):*** 295 ***Density(g/cm³):*** 1.833

Parameters

Fragment 1

**DIST1 (D)**	1.963
**DIST2 (D)**	2.073
**DIST3 (D)**	2.073
**DIST4 (D)**	1.963
**DIST5 (D)**	2.228
**DIST6 (D)**	2.228

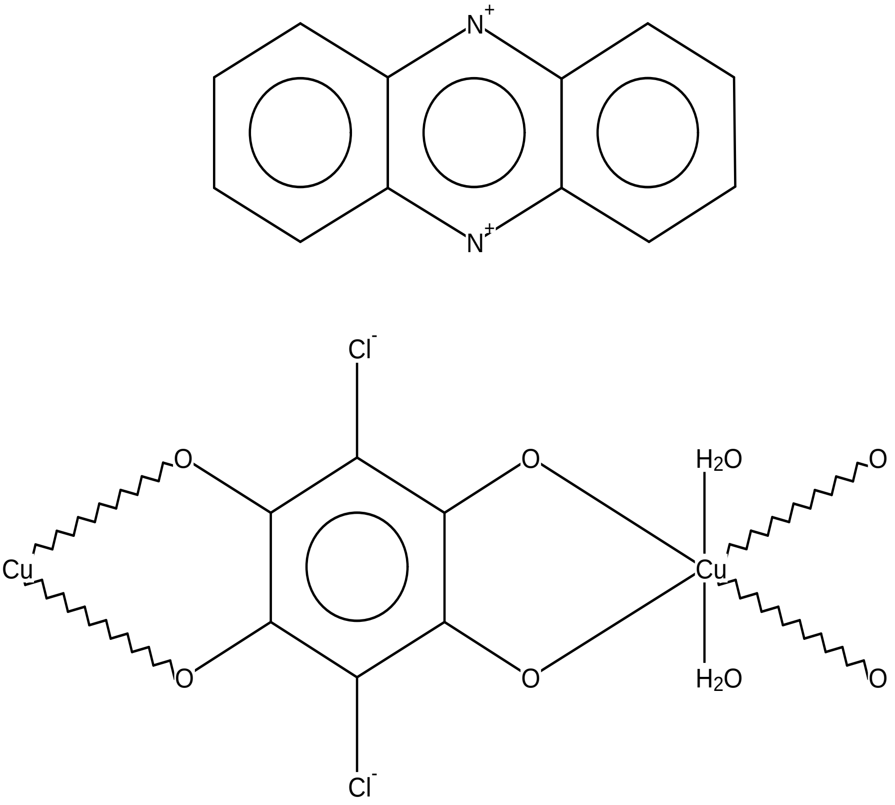

Search: search2 (Wed Jul 26 09:11:25 2017): Hit 184

TIYKUJ

Reference: N.L.Nkhili, W.Rekik, T.Mhiri, K.T.Mahmudov, M.N.Kopylovich, H.Naili (2014) *Inorg.Chim.Acta* ,**412,**27

Formula: $C_4 H_{12} N_2{}^{2+}, H_8 Cu_1 O_{12} Se_2{}^{2-}, 2(H_2 O_1)$

Compound Name: Piperazinediium tetra-aqua-bis(selenato)-copper dihydrate

Space Group:	P21/n	***Cell:***	***a*** 7.033(1)	***b*** 11.687(0)	***c*** 10.623(0)		
Space Group No.:	14	***(Å,°)***	α 90.00	β 104.13(1)	γ 90.00		
R-Factor (%):	2.01	***Temperature(K):***	293	***Density(g/cm³):***	2.140		

Parameters

Fragment 1

**DIST1 (D)**	1.992
**DIST2 (D)**	1.962
**DIST3 (D)**	1.992
**DIST4 (D)**	1.962
**DIST5 (D)**	2.290
**DIST6 (D)**	2.290

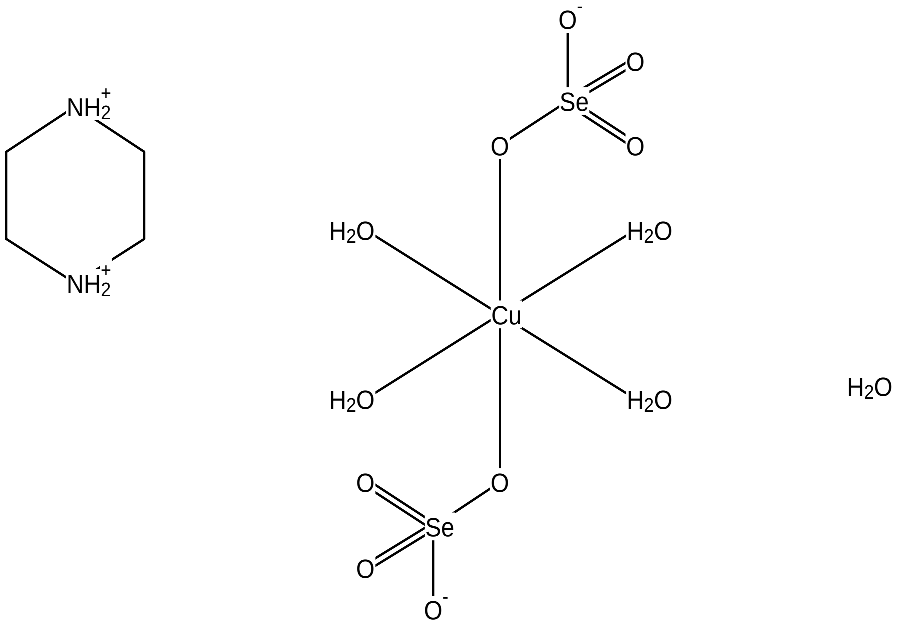

Search: search2 (Wed Jul 26 09:11:25 2017): Hit 185

TIZZOS

Reference:	A.N.Chekhlov (2007)<br>*Zh.Neorg.Khim.(Russ.)(Russ.J.Inorg.Chem.)* ,**52,**768
Formula:	C_{18} H_{38} N_2 O_6 $^{2+}$,Cu_1 N_4 O_{12} $^{2-}$
Compound Name:	4,7,13,16,21,24-Hexaoxa-1,10-diazoniabicyclo(8.8.8)-hexacosane bis(nitrato-O,O)-dinitrato-copper(ii)
Synonym:	dihydrogen (2,2,2-cryptand) bis(nitrato-O,O)-dinitrato-copper(ii)

Space Group:	P21/c	***Cell:***	***a***	10.264(2)	***b***	14.798(2)	***c***	19.516(3)
Space Group No.:	14	***(Å,°)***	α	90.00	β	100.20(2)	γ	90.00

R-Factor (%):	2.50	***Temperature(K):***	295	***Density(g/cm³):***	1.571

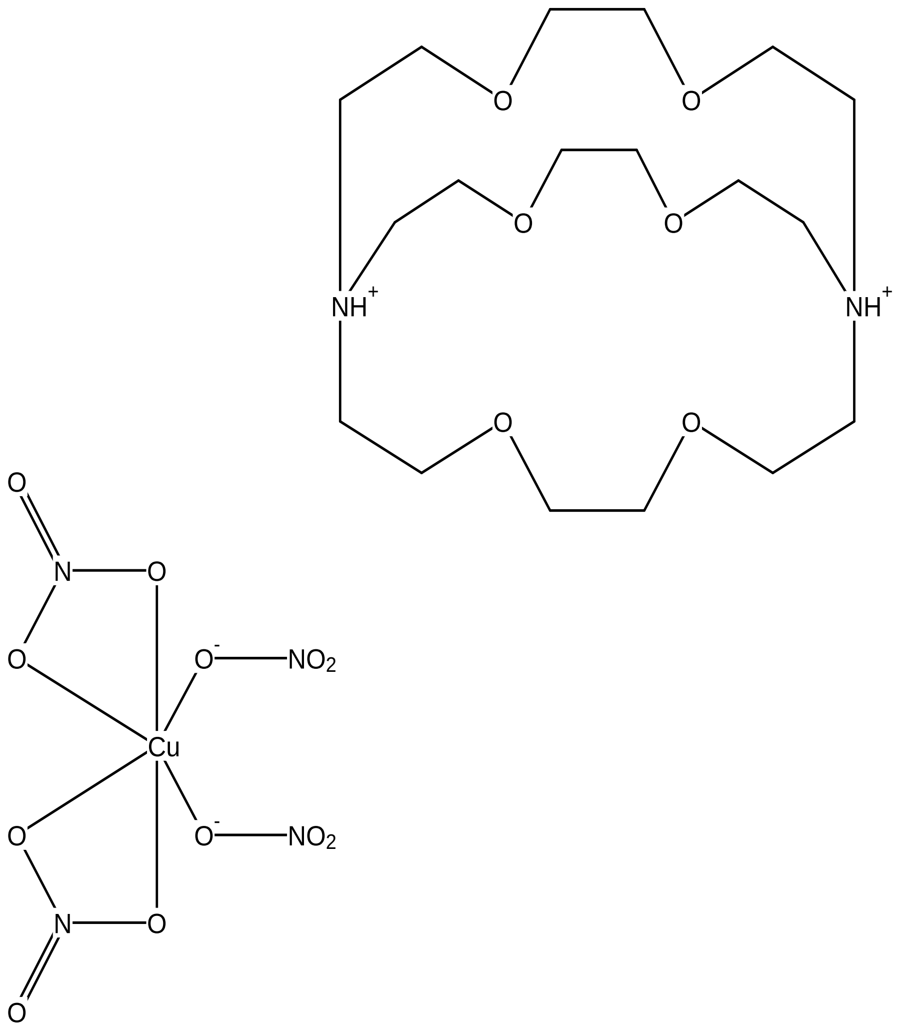

Parameters

Fragment 1

**DIST1 (D)**	1.965
**DIST2 (D)**	1.965
**DIST3 (D)**	1.977
**DIST4 (D)**	1.965
**DIST5 (D)**	2.563
**DIST6 (D)**	2.599

Search: search2 (Wed Jul 26 09:11:25 2017): Hit 186

UNIXEW

Reference: S.R.Bajpe, S.Henke, Jung-Hoon Lee, P.D.Bristowe, A.K.Cheetham (2016) *CrystEngComm* ,**18,**5327

Formula: H_{20} Cu_2 O_{50} Si_1 W_{12},6(H_2 O_1)

Compound Name: (μ-silicato)-hexacosakis(μ-oxo)-deca-aqua-decaoxo-di-copper-dodeca-tungsten hexahydrate

Space Group:	P-1	***Cell:***	***a***	12.890(0)	***b***	14.007(0)	***c***	15.338(0)
Space Group No.:	2	***(Å,°)***	α	63.17(0)	β	71.08(0)	γ	63.83(0)

R-Factor (%): 2.66 ***Temperature(K):*** 125 ***Density(g/cm^3):*** 4.987

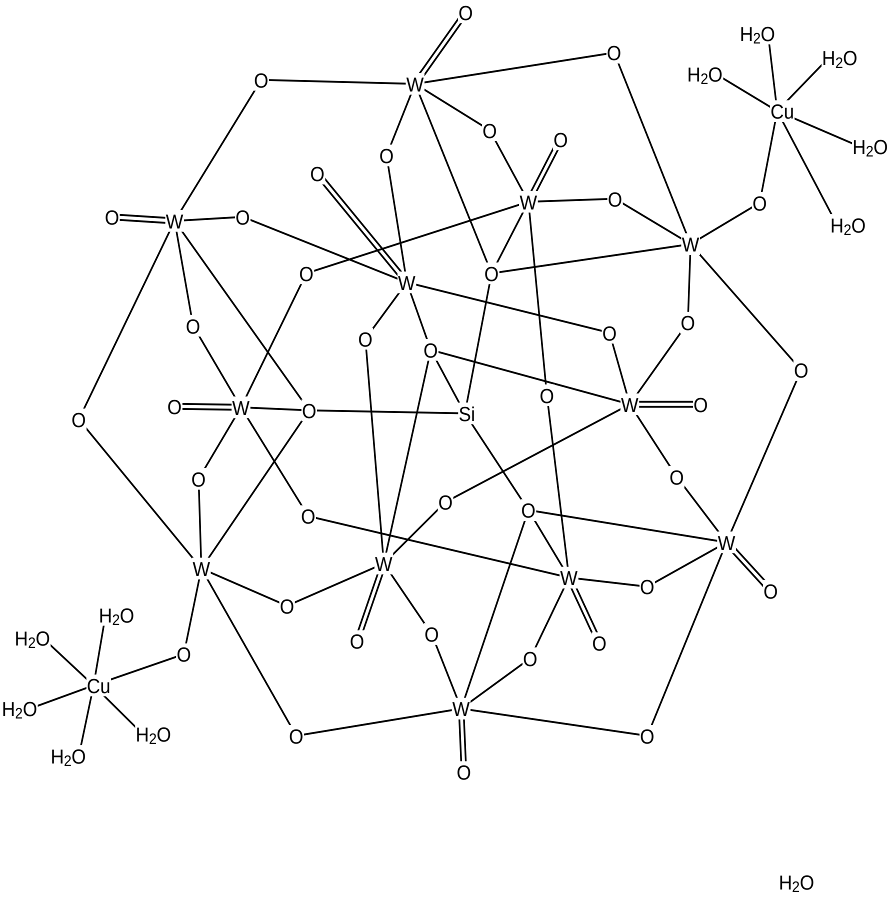

Parameters

Fragment 1

**DIST1 (D)**	1.979
**DIST2 (D)**	1.949
**DIST3 (D)**	1.996
**DIST4 (D)**	1.962
**DIST5 (D)**	2.413
**DIST6 (D)**	2.525

Fragment 2

**DIST1 (D)**	1.959
**DIST2 (D)**	1.972
**DIST3 (D)**	1.957
**DIST4 (D)**	1.950
**DIST5 (D)**	2.596
**DIST6 (D)**	2.473

Search: search2 (Wed Jul 26 09:11:25 2017): Hit 187

UNIXIA01

Reference:	S.R.Bajpe, S.Henke, Jung-Hoon Lee, P.D.Bristowe, A.K.Cheetham (2016) *CrystEngComm* ,**18,**5327
Formula:	H_{20} Cu_2 O_{50} Si_1 W_{12},6(H_2 O_1)
Compound Name:	(μ-silicato)-hexacosakis(μ-oxo)-deca-aqua-decaoxo-di-copper-dodeca-tungsten hexahydrate

Space Group:	P21/n	***Cell:***	***a*** 12.901(0)	***b*** 22.195(0)	***c*** 15.347(0)		
Space Group No.:	14	***(Å,°)***	α 90.00	β 94.43(0)	γ 90.00		
R-Factor (%):	2.76	***Temperature(K):***	124	***Density(g/cm3):***	4.987		

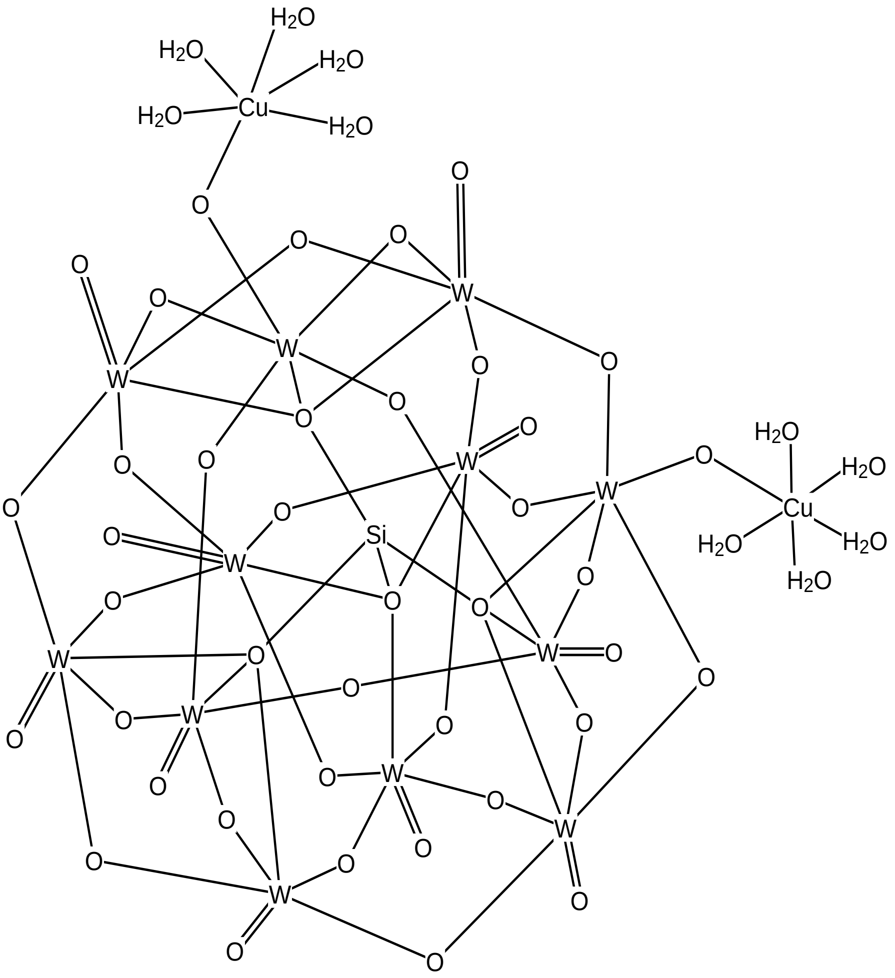

Parameters

Fragment 1

**DIST1 (D)**	1.977
**DIST2 (D)**	1.945
**DIST3 (D)**	1.960
**DIST4 (D)**	1.953
**DIST5 (D)**	2.672
**DIST6 (D)**	2.458

Fragment 2

**DIST1 (D)**	1.960
**DIST2 (D)**	1.964
**DIST3 (D)**	1.932
**DIST4 (D)**	1.990
**DIST5 (D)**	2.526
**DIST6 (D)**	2.519

H_2O

Search: search2 (Wed Jul 26 09:11:25 2017): Hit 188

VITKOA

Reference:	R.Koeferstein, C.Robl (2014) *Z.Anorg.Allg.Chem.* ,**640,**310
Formula:	$(C_{20} H_{12} Cu_3 O_{20}{}^{2-})n,n(C_6 H_{18} N_2{}^{2+}),6n(H_2 O_1)$
Compound Name:	catena-[hexane-1,6-diaminium (μ_6-benzene-1,2,4,5-tetracarboxylato)-(μ_4-benzene-1,2,4,5-tetracarboxylato)-tetraaqua-tri-copper hexahydrate]

Space Group:	P-1	***Cell:***	***a*** 7.726(0)	***b*** 11.104(0)	***c*** 11.120(0)		
Space Group No.:	2	***(Å,°)***	α 98.72(0)	β 108.25(0)	γ 95.56(0)		
R-Factor (%):	2.58	***Temperature(K):***	293	***Density(g/cm³):***	1.857		

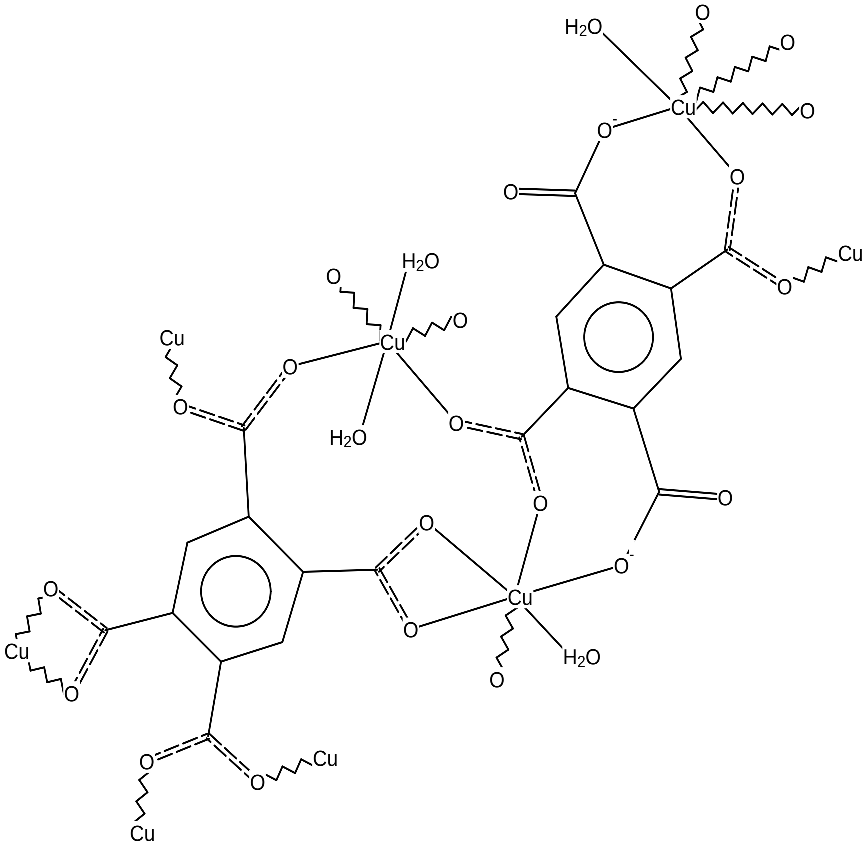

Parameters

Fragment 1

**DIST1 (D)**	1.937
**DIST2 (D)**	1.983
**DIST3 (D)**	1.960
**DIST4 (D)**	1.926
**DIST5 (D)**	2.701
**DIST6 (D)**	2.400

Fragment 2

**DIST1 (D)**	2.020
**DIST2 (D)**	1.944
**DIST3 (D)**	2.020
**DIST4 (D)**	1.944
**DIST5 (D)**	2.431
**DIST6 (D)**	2.431

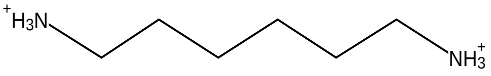

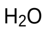

Search: search2 (Wed Jul 26 09:11:25 2017): Hit 189

VUBNIP

Reference: G.M.Frankenbach, M.A.Beno, A.M.Kini, J.M.Williams, U.Welp, J.E.Thompson, M.-H.Whangbo (1992) *Inorg.Chim.Acta* ,**192,** 195

Formula: $(C_4 H_8 Cu_1 O_8)n$

Compound Name: catena-(Tetra-aqua-(μ_2-squarato-O,O')-copper(ii))

Space Group:	P21/c	***Cell:***	***a*** 6.676(2)	***b*** 7.946(4)	***c*** 7.668(2)
Space Group No.:	14	***(Å,°)***	α 90.00	β 110.39(2)	γ 90.00

R-Factor (%): 2.00 ***Temperature(K):*** 295 ***Density(g/cm³):*** 2.157

Parameters

Fragment 1

**DIST1 (D)**	1.961
**DIST2 (D)**	1.948
**DIST3 (D)**	1.961
**DIST4 (D)**	1.948
**DIST5 (D)**	2.468
**DIST6 (D)**	2.468

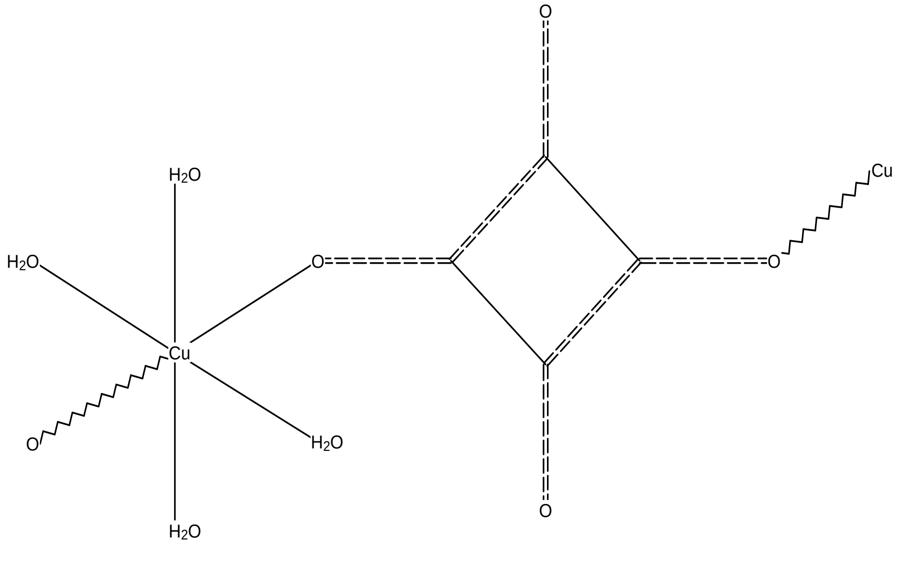

Search: search2 (Wed Jul 26 09:11:25 2017): Hit 190

WAJPAY

Reference:	V.Kh.Sabirov, M.A.Porai-Koshits, Yu.T.Struchkov, A.N.Yunuskhodzhaev, A.F.Dusmatov (1992) *Koord.Khim.(Russ.)(Coord.Chem.)* ,**18,**292
Formula:	C_{12} H_{24} Cu_1 N_6 O_{12}
Compound Name:	Diaqua-bis(2-oxo-1-pyrrolidineacetamide-O)-bis(nitrato-O)-copper(ii)
Synonym:	Diaqua-bis(piracetam)-bis(nitrato-O)-copper(ii)

Space Group:	P21/n	***Cell:***	***a*** 9.231(2)	***b*** 6.045(1)	***c*** 17.759(4)
Space Group No.:	14	***(Å,°)***	α 90.00	β 94.70(2)	γ 90.00
R-Factor (%):	2.90	***Temperature(K):*** 153		***Density(g/cm³):*** 1.708	

Parameters

Fragment 1

**DIST1 (D)**	1.941
**DIST2 (D)**	1.963
**DIST3 (D)**	1.963
**DIST4 (D)**	1.941
**DIST5 (D)**	2.421
**DIST6 (D)**	2.421

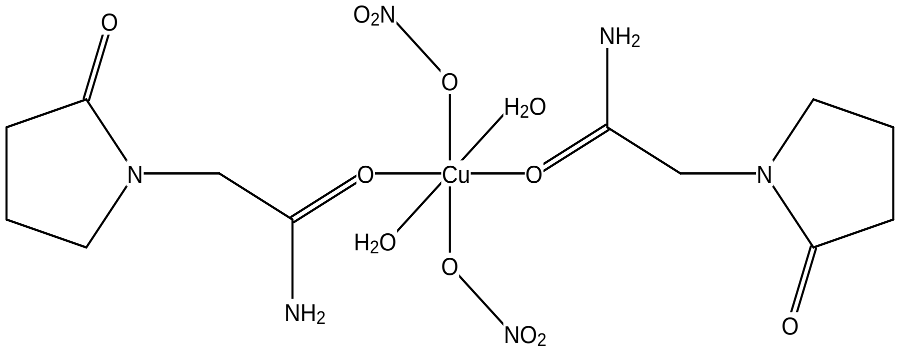

Search: search2 (Wed Jul 26 09:11:25 2017): Hit 191

WEVQOD

Reference:	R.Baggio, M.T.Garland, Y.Moreno, O.Pena, M.Perec, E.Spodine (2000) *J.Chem.Soc.,Dalton Trans.* ,2061
Formula:	$(C_{24} H_{36} Cu_3 Gd_2 O_{36})n,12n(H_2 O_1)$
Compound Name:	catena-(hexakis(μ_3-2,2'-Oxydiacetato)-hexa-aqua-tri-copper(ii)-di-gadolinium(iii) dodecahydrate)

Space Group:	P6/mcc	***Cell:***	***a***	14.717(2)	***b***	14.717(2)	***c***	15.169(3)
Space Group No.:	192	***(Å,°)***	α	90.00	β	90.00	γ	120.00

R-Factor (%): 2.40 ***Temperature(K):*** 295 ***Density(g/cm³):*** 1.893

Parameters

Fragment 1

**DIST1 (D)**	1.955
**DIST2 (D)**	1.955
**DIST3 (D)**	1.955
**DIST4 (D)**	1.955
**DIST5 (D)**	2.510
**DIST6 (D)**	2.510

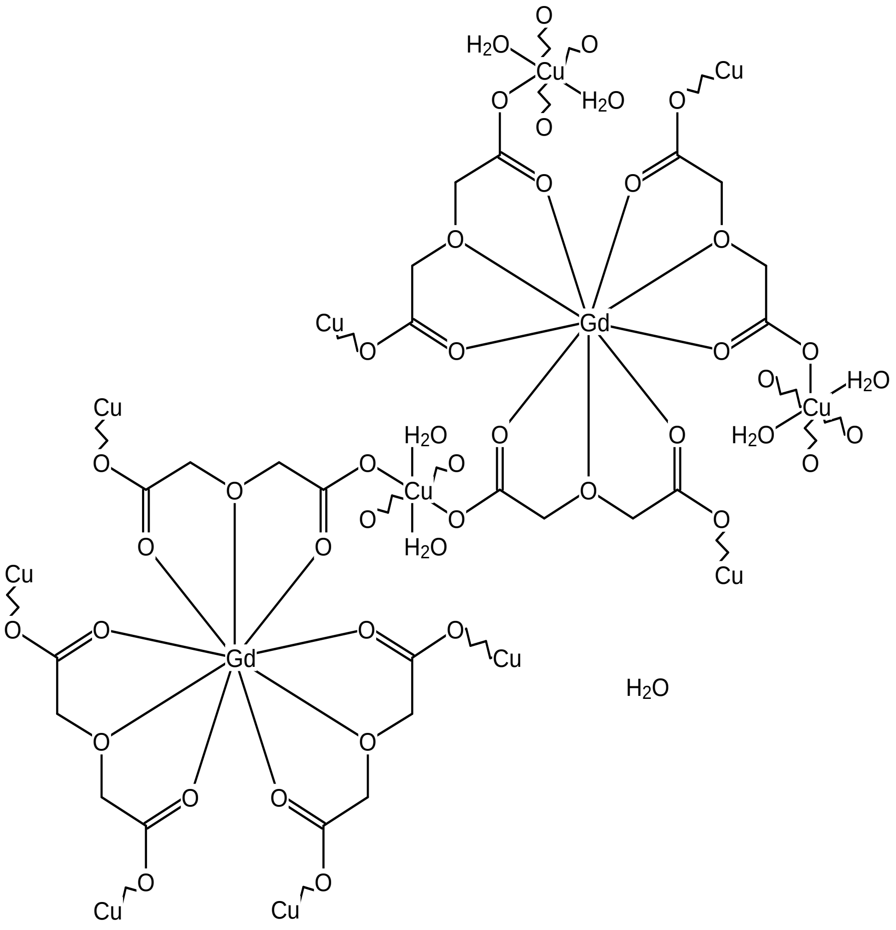

Search: search2 (Wed Jul 26 09:11:25 2017): Hit 192

WIZGES

Reference:	Ming-Lin Guo, Hong-Jun Zang (2008)<br>*Acta Crystallogr.,Sect.C:Cryst.Struct.Commun.* ,**64,**m173
Formula:	$(C_{10} H_{16} Cu_1 O_{14} P_2)$n,2n$(H_2 O_1)$
Compound Name:	catena-[bis(μ$_2$-1-Oxo-2,6,7-trioxa-1-phosphabicyclo(2.2.2)octane-4-carboxylato-O,O')-diaqua-copper(ii) dihydrate]

Space Group:	P21/c	***Cell:***	***a*** 8.340(3)	***b*** 8.863(3)	***c*** 12.565(4)		
Space Group No.:	14	***(Å,°)***	α 90.00	β 99.58(0)	γ 90.00		
R-Factor (%):	2.65	***Temperature(K):***	294	***Density(g/cm³):***	1.892		

Parameters

Fragment 1

**DIST1 (D)**	1.941
**DIST2 (D)**	1.908
**DIST3 (D)**	1.908
**DIST4 (D)**	1.941
**DIST5 (D)**	2.704
**DIST6 (D)**	2.704

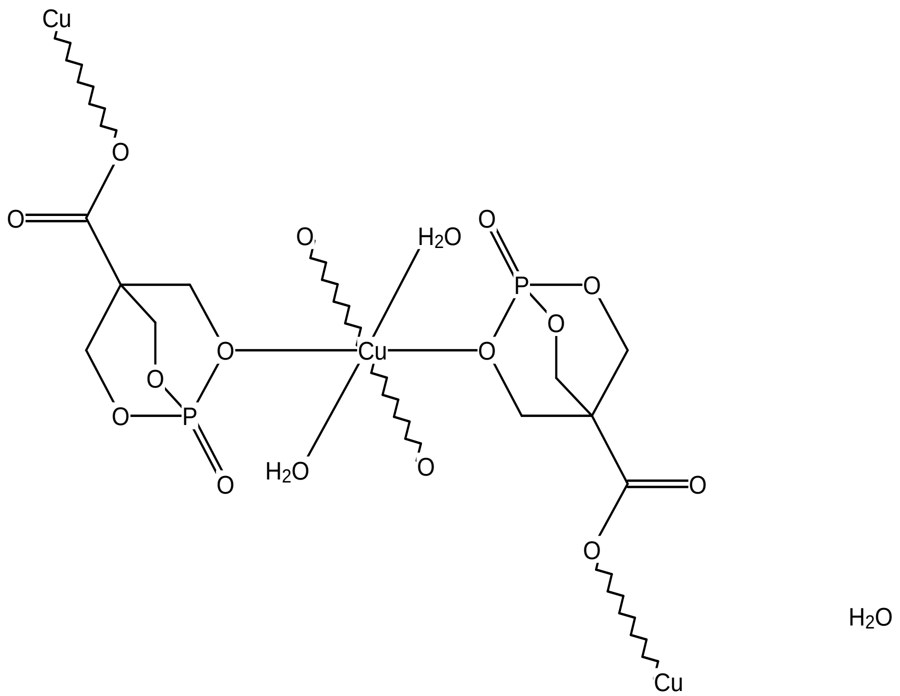

Search: search2 (Wed Jul 26 09:11:25 2017): Hit 193

WOQMAR

Reference:	Yun-Yun Wang, Rui-Ding Hu, Yan-Jun Wang (2009)<br>*Acta Crystallogr.,Sect.E:Struct.Rep.Online* ,**65,**m169
Formula:	$(C_8 H_{12} Cu_1 O_7)n$
Compound Name:	catena-((μ_2-7-Oxabicyclo[2.2.1]heptane-2,3-dicarboxylato-O,O',O'')-diaqua-copper(ii))

Space Group:	Iba2	***Cell:***	***a*** 10.551(0)	***b*** 19.339(0)	***c*** 9.744(0)		
Space Group No.:	45	***(Å,°)***	α 90.00	β 90.00	γ 90.00		
R-Factor (%):	2.23	***Temperature(K):***	296	***Density(g/cm³):***	1.896		

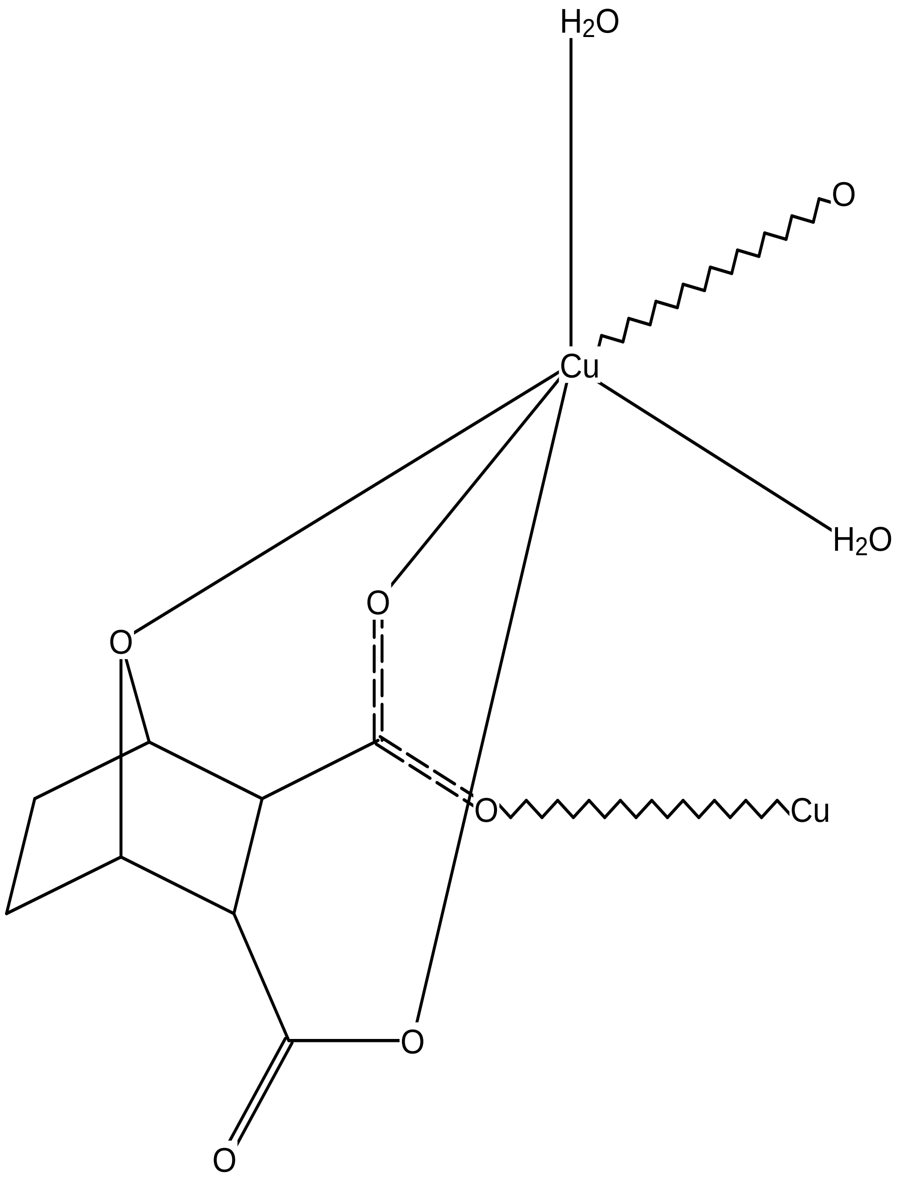

Parameters

Fragment 1

**DIST1 (D)**	1.990
**DIST2 (D)**	1.952
**DIST3 (D)**	2.054
**DIST4 (D)**	1.931
**DIST5 (D)**	2.315
**DIST6 (D)**	2.373

Search: search2 (Wed Jul 26 09:11:25 2017): Hit 194

WULSAY

Reference: Wen Zhang, Li-Zhuang Chen, Ren-Gen Xiong, T.Nakamura, S.D.Huang (2009) *J.Am.Chem.Soc.* ,**131,**12544

Formula: $C_6 H_{14} N_2{}^{2+}$,$H_{12} Cu_1 O_6{}^{2+}$,2($O_4 Se_1{}^{2-}$)

Compound Name: 1,4-Diazoniabicyclo[2.2.2]octane hexa-aqua-copper(ii) bis(selenate)

Space Group:	P21	***Cell:***	***a***	7.147(0)	***b***	12.615(0)	***c***	9.952(0)
Space Group No.:	4	***(Å,°)***	α	90.00	β	91.73(0)	γ	90.00

R-Factor (%): 2.81 ***Temperature(K):*** 103 ***Density(g/cm^3):*** 2.117

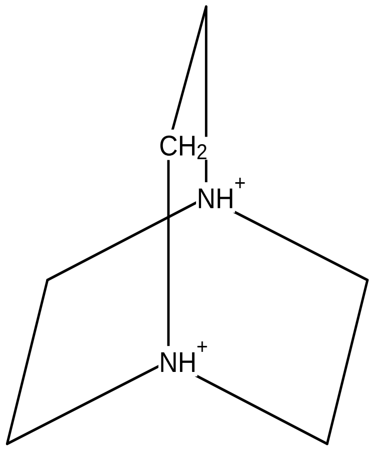

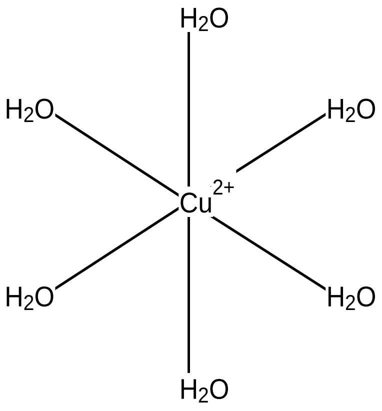

Parameters

Fragment 1

**DIST1 (D)**	1.989
**DIST2 (D)**	1.964
**DIST3 (D)**	1.989
**DIST4 (D)**	1.975
**DIST5 (D)**	2.375
**DIST6 (D)**	2.383

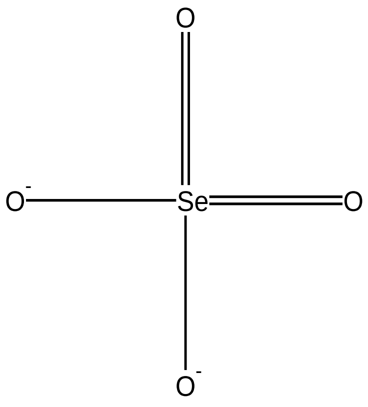

Search: search2 (Wed Jul 26 09:11:25 2017): Hit 195

WULSAY02

Reference:	Wen Zhang, Li-Zhuang Chen, Ren-Gen Xiong, T.Nakamura, S.D.Huang (2009) *J.Am.Chem.Soc.* ,**131,**12544
Formula:	$C_6 H_{12} D_2 N_2{}^{2+}, D_{12} Cu_1 O_6{}^{2+}, 2(O_4 Se_1{}^{2-})$
Compound Name:	Dideutero-1,4-diazoniabicyclo[2.2.2]octane hexakis(dideuterium oxide)-copper(ii) bis(selenate)

Space Group:	P21	***Cell:***	***a*** 7.140(0)	***b***	12.602(0)	***c***	9.944(0)
Space Group No.:	4	***(Å,°)***	α 90.00	β	91.94(0)	γ	90.00
R-Factor (%):	2.66	***Temperature(K):***	93	***Density(g/cm³):***	2.175		

Parameters

Fragment 1

**DIST1 (D)**	1.979
**DIST2 (D)**	1.971
**DIST3 (D)**	1.986
**DIST4 (D)**	1.986
**DIST5 (D)**	2.390
**DIST6 (D)**	2.370

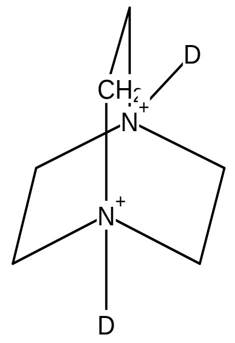

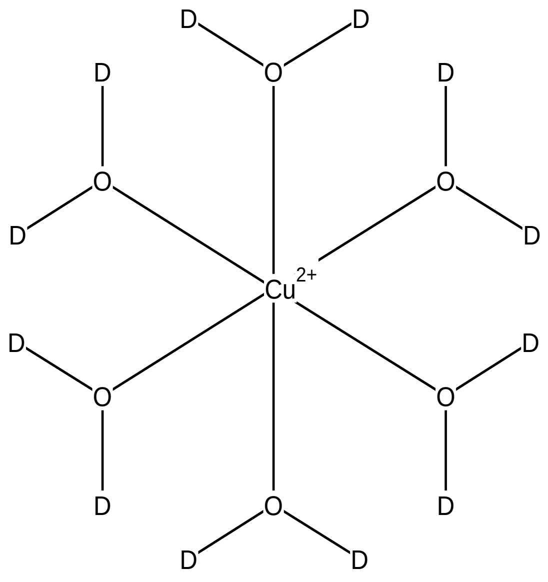

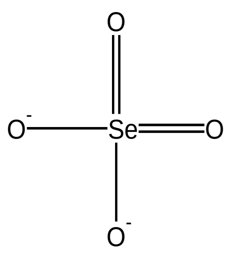

Search: search2 (Wed Jul 26 09:11:25 2017): Hit 196

WUZXOF

Reference: N.V.Tsaryk, A.V.Dudko, A.N.Kozachkova, V.V.Bon, V.I.Pekhnyo (2010) *Acta Crystallogr.,Sect.E:Struct.Rep.Online* ,**66,** m1533

Formula: $C_{10} H_{32} Cu_1 N_2 O_{14} P_4$

Compound Name: trans-Diaqua-(bis(1,1-bis(phosphono)pentan-1-aminiumato))-copper(ii)

Space Group:	P-1	***Cell:***	***a*** 5.563(0)	***b*** 10.024(0)	***c*** 10.524(0)
Space Group No.:	2	***(Å,°)***	α 69.31(0)	β 86.67(0)	γ 88.40(0)

R-Factor (%): 2.34 ***Temperature(K):*** 173 ***Density(g/cm³):*** 1.793

Parameters

Fragment 1

**DIST1 (D)**	1.952
**DIST2 (D)**	1.938
**DIST3 (D)**	1.952
**DIST4 (D)**	1.938
**DIST5 (D)**	2.567
**DIST6 (D)**	2.567

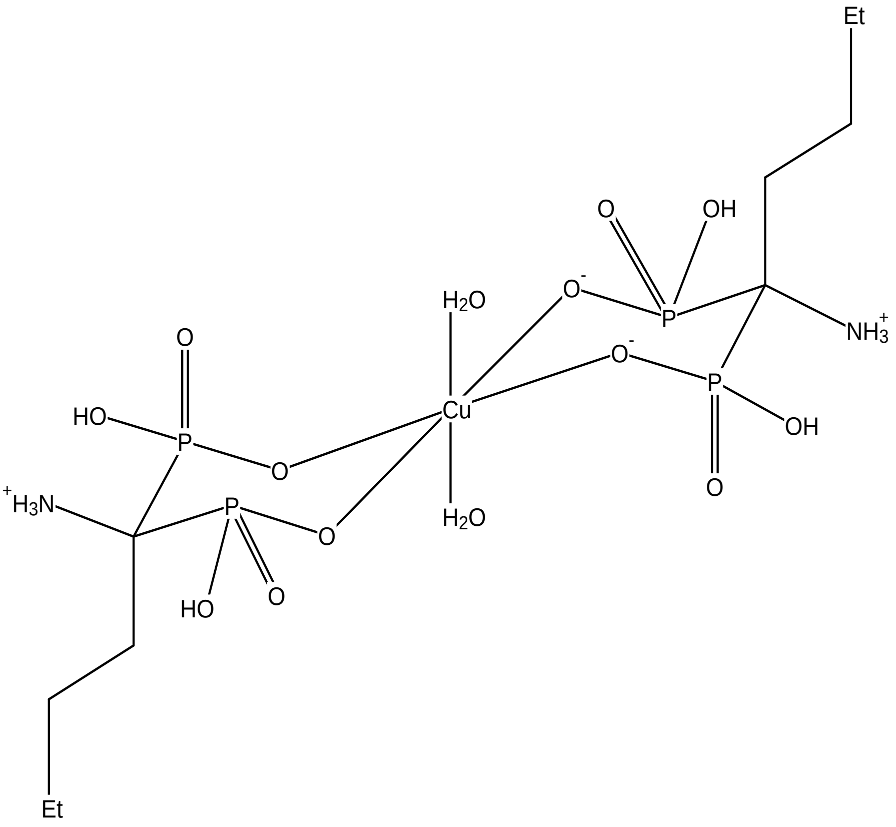

Search: search2 (Wed Jul 26 09:11:25 2017): Hit 197

XACMIA

Reference: Xiuli Wang, Danna Liu, Hongyan Lin, Guocheng Liu, Xiang Wang, Mao Le, Xing Rong (2016) *CrystEngComm* ,**18,**888

Formula: $(C_{24} H_{22} Cu_2 Mo_8 N_6 O_{34})n$

Compound Name: catena-(bis(μ_5-Oxo)-hexakis(μ_3-oxo)-bis(μ_2-3-(pyridin-3-ylcarbamoyl) pyridine-2-carboxylato)-bis(μ_2-hydroxo)-tetrakis(μ_2-oxo)-diaqua-dodecaoxo-di-copper-octa-molybdenum)

Space Group:	P-1	***Cell:***	***a***	9.942(0)	***b***	10.231(0)	***c***	11.093(0)
Space Group No.:	2	***(Å,°)***	α	84.35(0)	β	89.57(0)	γ	88.02(0)

R-Factor (%): 2.43 ***Temperature(K):*** 296 ***Density(g/cm³):*** 2.712

Parameters

Fragment 1

**DIST1 (D)**	1.985
**DIST2 (D)**	1.976
**DIST3 (D)**	1.976
**DIST4 (D)**	1.985
**DIST5 (D)**	2.390
**DIST6 (D)**	2.390

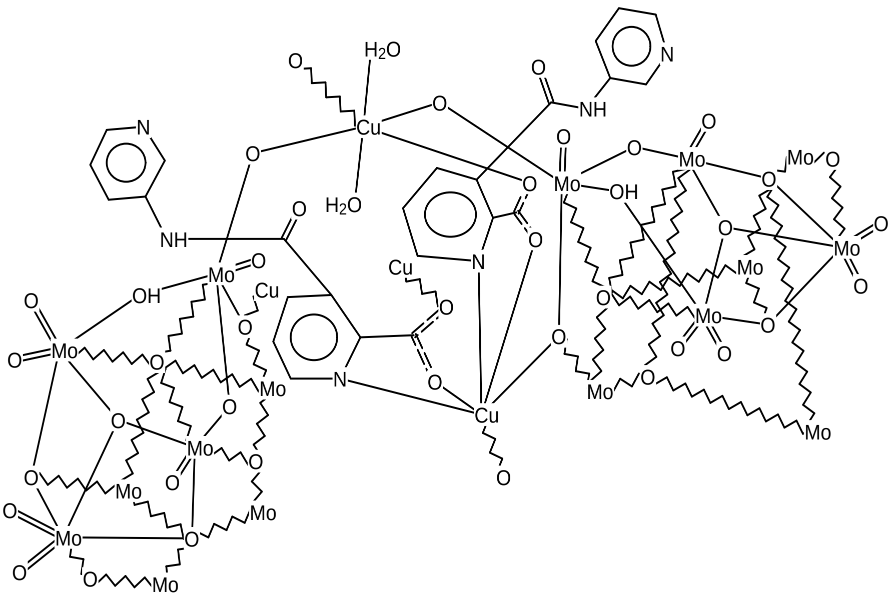

Search: search2 (Wed Jul 26 09:11:25 2017): Hit 198

XAXMEO

Reference: Jian Huang, Yi-Zhi Li, Gang-Chun Sun, Rong-Bin Dai, Qin-Xi Li, Liu-Fang Wang, Chun-Gu Xia (2000) *Acta Crystallogr.,Sect.C:Cryst.Struct.Commun.* ,**56,**e489

Formula: $C_{12} H_{16} Cu_1 F_2 N_4 O_{12}$,4($H_2 O_1$)

Compound Name: Tetra-aqua-bis(5-fluorouracil-1-acetato-O)-copper(ii) tetrahydrate

Space Group:	P21/c	***Cell:***	***a*** 8.304(1)	***b*** 12.044(2)	***c*** 11.082(2)
Space Group No.:	14	***(Å,°)***	α 90.00	β 92.47(1)	γ 90.00

R-Factor (%): 2.00 ***Temperature(K):*** 295 ***Density(g/cm³):*** 1.745

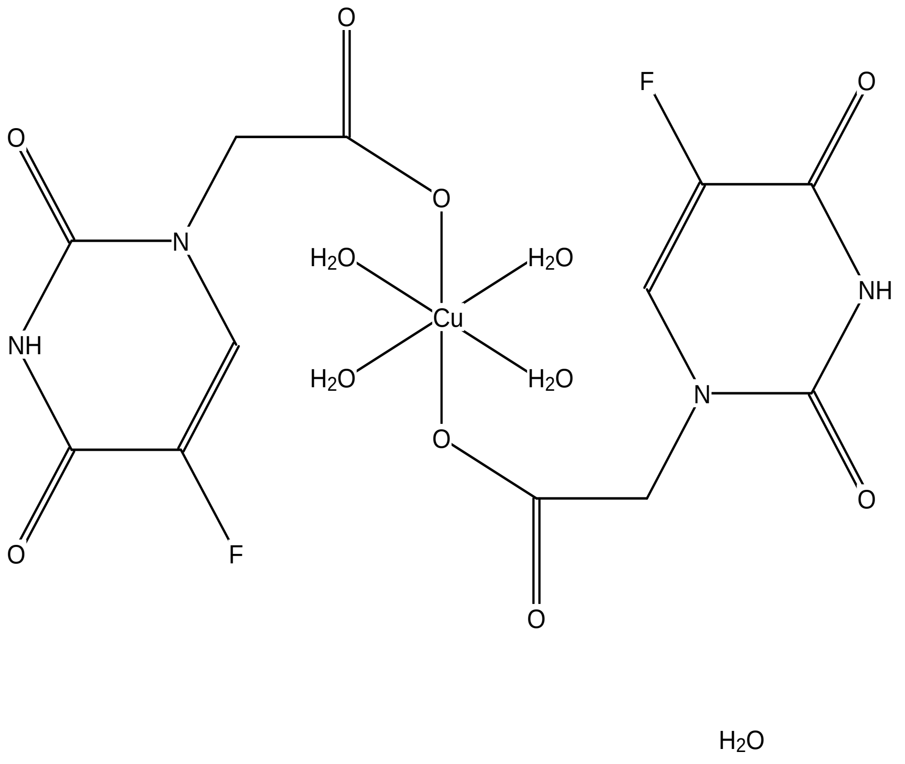

Parameters

Fragment 1

**DIST1 (D)**	1.924
**DIST2 (D)**	1.955
**DIST3 (D)**	1.955
**DIST4 (D)**	1.924
**DIST5 (D)**	2.577
**DIST6 (D)**	2.577

Search: search2 (Wed Jul 26 09:11:25 2017): Hit 199

XEHTIO

Reference:	Yun Gong, Wang Tang, Wenbin Hou, Zhongyong Zha, Changwen Hu (2006) *Inorg.Chem.* ,**45,**4987
Formula:	$(C_6\ H_{20}\ Cu_3\ N_2\ O_{16}\ P_4)n$
Compound Name:	catena-(bis(μ_4-3-Ammonium-1-hydroxypropylidene-1,1-bisphosphonato) -diaqua-tri-copper(ii))

Space Group:	P21/n	***Cell:***	***a*** 10.232(4)	***b***	7.961(3)	***c***	11.016(4)
Space Group No.:	14	***(Å,°)***	α 90.00	β	101.45(0)	γ	90.00

R-Factor (%):	2.37	***Temperature(K):***	298	***Density(g/cm³):***	2.608

Parameters

Fragment 1

**DIST1 (D)**	1.954
**DIST2 (D)**	1.968
**DIST3 (D)**	2.147
**DIST4 (D)**	1.975
**DIST5 (D)**	2.396
**DIST6 (D)**	2.358

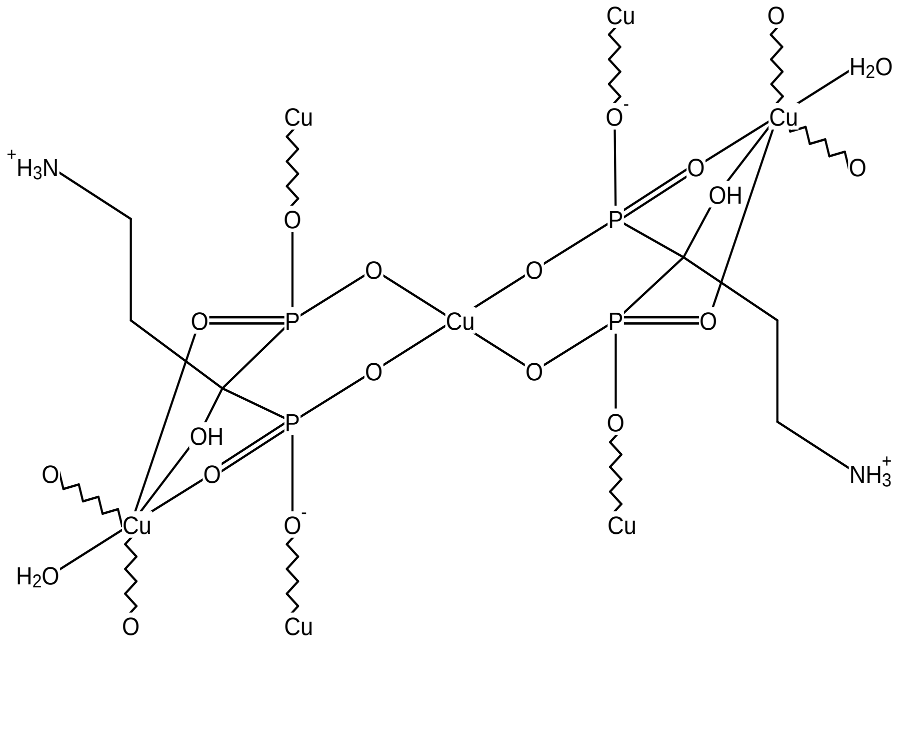

Search: search2 (Wed Jul 26 09:11:25 2017): Hit 200

XOQHIW

Reference:	Qi-Lin Lu, Jian Luan, Xiu-Li Wang, Hong-Yan Lin, Mao Le, Guo-Cheng Liu (2014) *Polyhedron* ,**83,**108
Formula:	$(C_{34}\ H_{34}\ Cu_2\ N_4\ O_{12})n$
Compound Name:	catena-((μ_2-N,N'-(Hexane-1,6-diyl)dinicotinamide)-bis(μ_2-terephthalato)-diaqua-di-copper)

Space Group:	P-1	***Cell:***	***a***	8.540(0)	***b***	9.066(0)	***c***	11.650(1)
Space Group No.:	2	***(Å,°)***	α	86.83(0)	β	69.09(0)	γ	87.44(0)

R-Factor (%): 2.78 ***Temperature(K):*** 296 ***Density(g/cm^3):*** 1.615

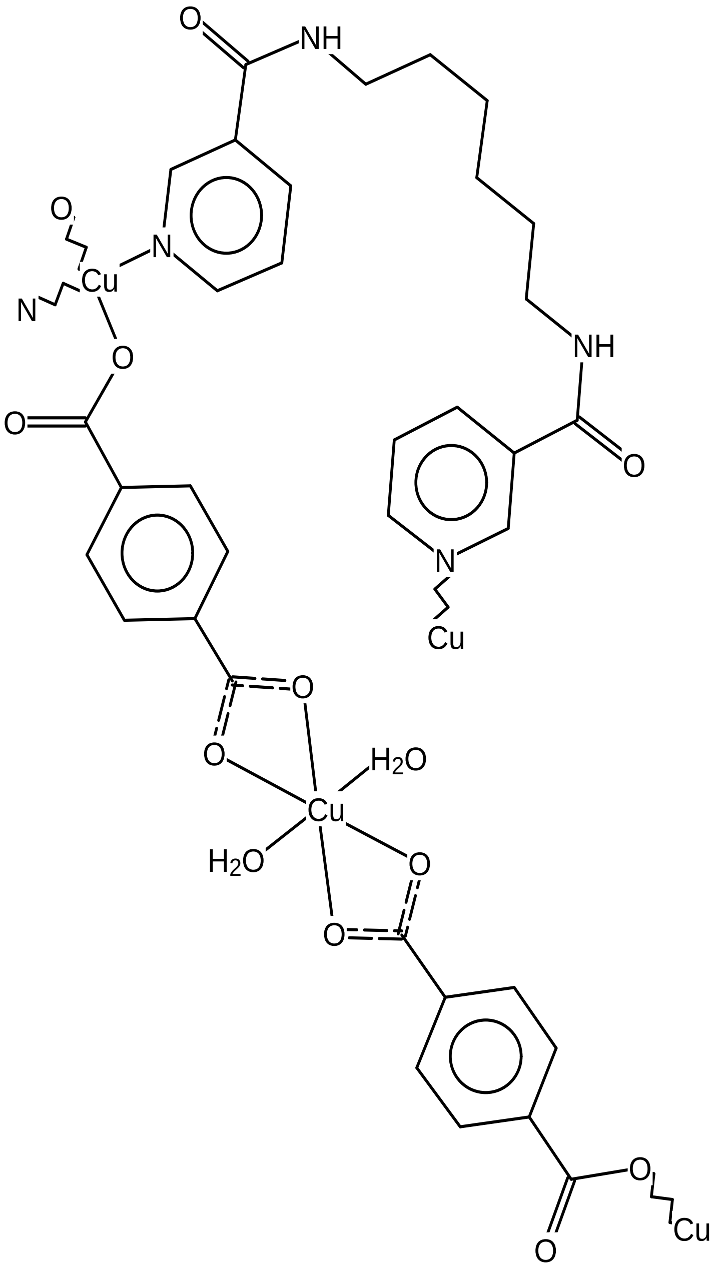

Parameters

Fragment 1

**DIST1 (D)**	1.977
**DIST2 (D)**	1.978
**DIST3 (D)**	1.978
**DIST4 (D)**	1.977
**DIST5 (D)**	2.496
**DIST6 (D)**	2.496

Search: search2 (Wed Jul 26 09:11:25 2017): Hit 201

XOZVEN

Reference: L.R.Falvello, R.Garde, M.Tomas (2002) *Inorg.Chem.* ,**41,** 4599

Formula: $C_6 H_{10} Cu_1 N_4 O_{10}, 2(H_2 O_1)$

Compound Name: trans-Diaqua-bis(oxalurato-O,O')-copper(ii) dihydrate

Space Group:	P-1	***Cell:***	***a***	5.258(0)	***b***	6.833(1)	***c***	10.314(2)
Space Group No.:	2	***(Å,°)***	α	72.69(2)	β	83.39(2)	γ	71.27(1)

R-Factor (%): 2.82 ***Temperature(K):*** 148 ***Density(g/cm³):*** 1.972

Parameters

Fragment 1

**DIST1 (D)**	1.961
**DIST2 (D)**	1.957
**DIST3 (D)**	1.957
**DIST4 (D)**	1.961
**DIST5 (D)**	2.379
**DIST6 (D)**	2.379

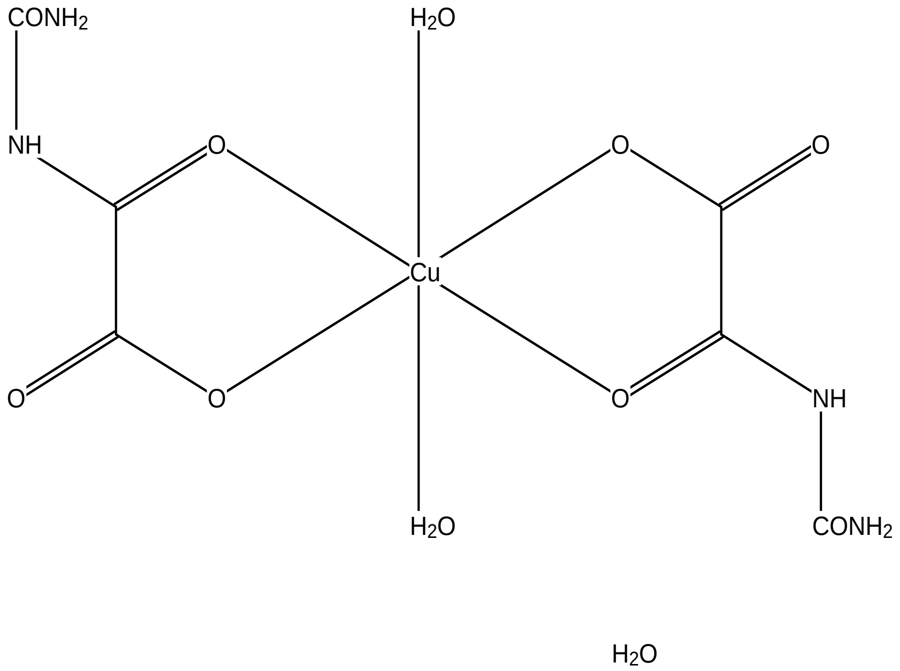

Search: search2 (Wed Jul 26 09:11:25 2017): Hit 202

YEDZOX

Reference:	Qi-Hua Zhao, Lin Du, Rui-Bing Fang (2006)<br>*Acta Crystallogr.,Sect.E:Struct.Rep.Online* ,**62,**m360
Formula:	$2(C_3 H_{12} N_2{}^{2+}),H_{12} Cu_1 O_6{}^{2+},O_{28} V_{10}{}^{6-},7(H_2 O_1)$
Compound Name:	bis(Trimethylenediammonium) hexaaqua-copper(ii) decavanadate heptahydrate

Space Group:	P-1	***Cell:***	***a*** 10.351(2)	***b*** 11.125(2)	***c*** 18.536(4)
Space Group No.:	2	***(Å,°)***	α 92.62(3)	β 103.14(3)	γ 100.17(3)
R-Factor (%):	2.76	***Temperature(K):*** 253		***Density(g/cm^3):*** 2.294	

Parameters

Fragment 1

**DIST1 (D)**	2.139
**DIST2 (D)**	2.080
**DIST3 (D)**	2.040
**DIST4 (D)**	2.111
**DIST5 (D)**	2.098
**DIST6 (D)**	2.070

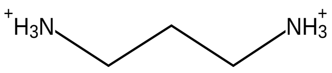

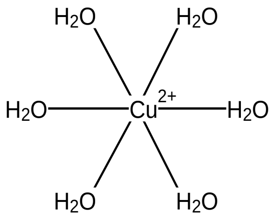

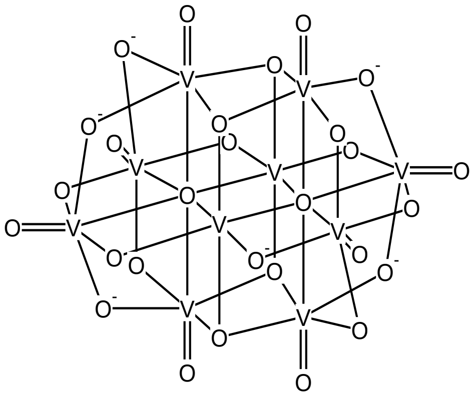

H_2O

Search: search2 (Wed Jul 26 09:11:25 2017): Hit 203

YEYTAY

Reference: Ai-E Shi, Bai-Yan Li, Guang-Feng Hou, Jin-Sheng Gao
(2007) *Acta Crystallogr.,Sect.E:Struct.Rep.Online* ,**63,**m471

Formula: $C_{18} H_{24} Cu_1 N_2 O_{12}$,4($H_2 O_1$)

Compound Name: trans-Tetraaqua-bis(2,6-dimethylpyridinium-3,5-dicarboxylato)-copper(ii) tetrahydrate

Space Group:	P-1	***Cell:***	***a*** 6.867(5)	***b*** 9.203(6)	***c*** 11.108(8)
Space Group No.:	2	***(Å,°)***	α 111.22(3)	β 102.55(3)	γ 96.83(3)

R-Factor (%): 2.90 ***Temperature(K):*** 291 ***Density(g/cm³):*** 1.587

Parameters

Fragment 1

**DIST1 (D)**	1.951
**DIST2 (D)**	1.954
**DIST3 (D)**	1.954
**DIST4 (D)**	1.951
**DIST5 (D)**	2.503
**DIST6 (D)**	2.503

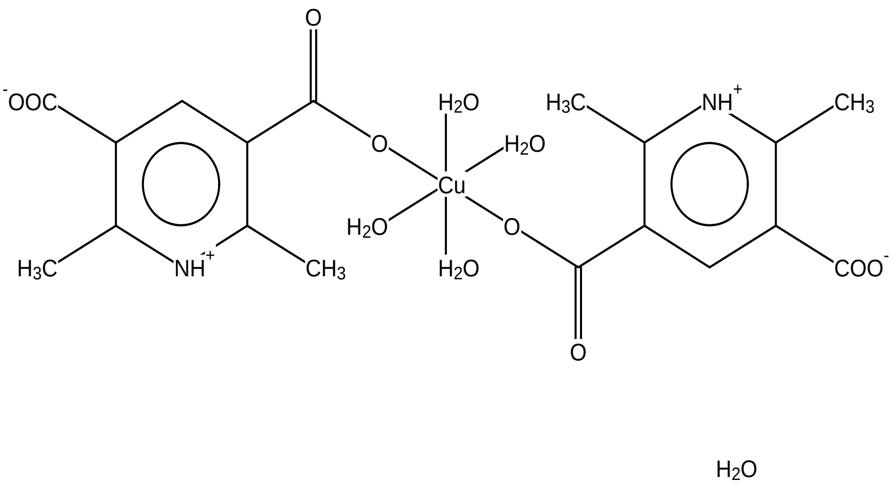

Search: search2 (Wed Jul 26 09:11:25 2017): Hit 204

YEYTAY01

Reference:	Cui-Ling Chen, Ye Zou, Ping Qiu, Yu-Hua Wen, Jun-Yong Li, Ze-Hong Hong, Xiao-Ming Lin, An-Xwu Xu, Yue-Peng Cai (2009) *J.Coord.Chem.* ,**62,**2480
Formula:	C_{18} H_{24} Cu_1 N_2 O_{12},4(H_2 O_1)
Compound Name:	Tetra-aqua-bis(2,6-dimethylpyridinium-3,5-dicarboxylato-O)-copper(ii) tetrahydrate

Space Group:	P-1	***Cell:***	***a*** 7.531(0)	***b*** 8.755(0)	***c*** 10.828(0)		
Space Group No.:	2	***(Å,°)***	α 69.77(0)	β 69.67(0)	γ 83.59(0)		
R-Factor (%):	2.67	***Temperature(K):***	298	***Density(g/cm³):***	1.575		

Parameters

Fragment 1

**DIST1 (D)**	2.049
**DIST2 (D)**	2.046
**DIST3 (D)**	2.046
**DIST4 (D)**	2.049
**DIST5 (D)**	2.210
**DIST6 (D)**	2.210

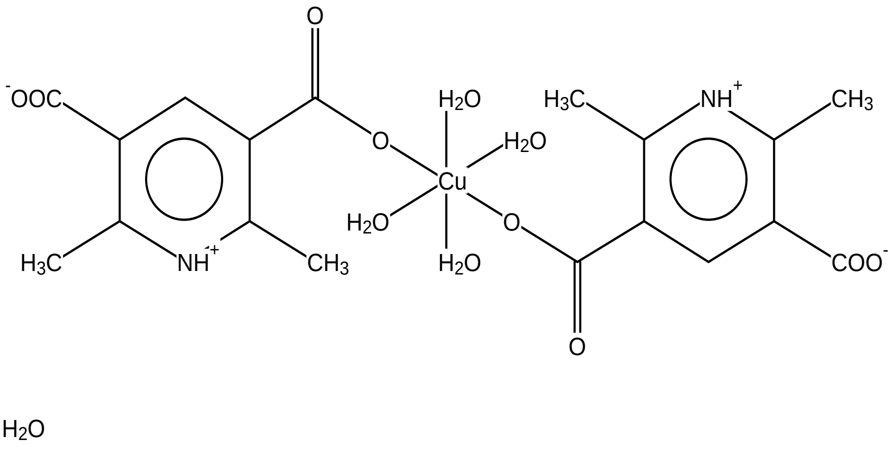

Search: search2 (Wed Jul 26 09:11:25 2017): Hit 205

YOLFOU

Reference:	A.Bouayad, J.-C.Trombe, A.Gleizes (1995) *Inorg.Chim.Acta* ,**230,**1
Formula:	$(C_{12}\ H_{12}\ Ba_2\ Cu_1\ O_{18})n$
Compound Name:	catena-((μ_6-Squarato)-bis(μ_3-squarato)-tetrakis(μ_2-aqua)-diaqua-di-barium-copper(ii))

Space Group:	P-1	***Cell:***	***a*** 7.752(1)	***b*** 10.445(2)	***c*** 7.420(1)
Space Group No.:	2	***(Å,°)***	α 99.24(1)	β 113.20(1)	γ 109.87(1)

R-Factor (%):	2.60	***Temperature(K):***	295	***Density(g/cm³):***	2.661

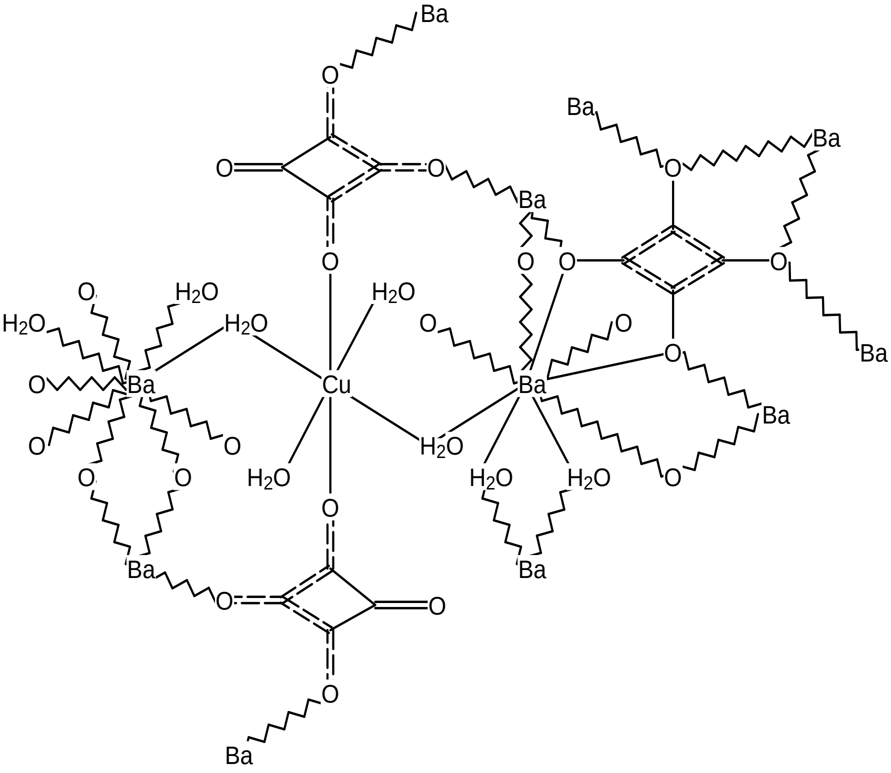

Parameters

Fragment 1

**DIST1 (D)**	1.960
**DIST2 (D)**	1.945
**DIST3 (D)**	1.945
**DIST4 (D)**	1.960
**DIST5 (D)**	2.533
**DIST6 (D)**	2.533

Search: search2 (Wed Jul 26 09:11:25 2017): Hit 206

YOXHOK

Reference: I.Bulimestru, S.Shova, N.Popa, P.Roussel, F.Capet, R.-N.Vannier, N.Djelal, L.Burylo, J.-P.Wignacourt, A.Gulea, K.H.Whitmire (2014) *Chem.Mater.* ,**26,**6092

Formula: $(C_{28}\ H_{44}\ Bi_2\ Cu_1\ N_4\ O_{20})n,n(H_2\ O_1)$

Compound Name: catena-(bis(μ_4-Cyclohexane-1,2-diamine-N,N,N',N'-tetra-acetato)-tetra-aqua-di-bismuth-copper monohydrate)

Space Group:	P-1	***Cell:***	***a*** 7.323(0)		***b*** 8.672(0)		***c*** 14.687(0)
Space Group No.:	2	***(Å,°)***	α 94.23(0)		β 95.06(0)		γ 107.97(0)

R-Factor (%): 1.55 ***Temperature(K):*** 100 ***Density(g/cm^3):*** 2.374

Parameters

Fragment 1

**DIST1 (D)**	1.976
**DIST2 (D)**	1.961
**DIST3 (D)**	1.976
**DIST4 (D)**	1.961
**DIST5 (D)**	2.390
**DIST6 (D)**	2.390

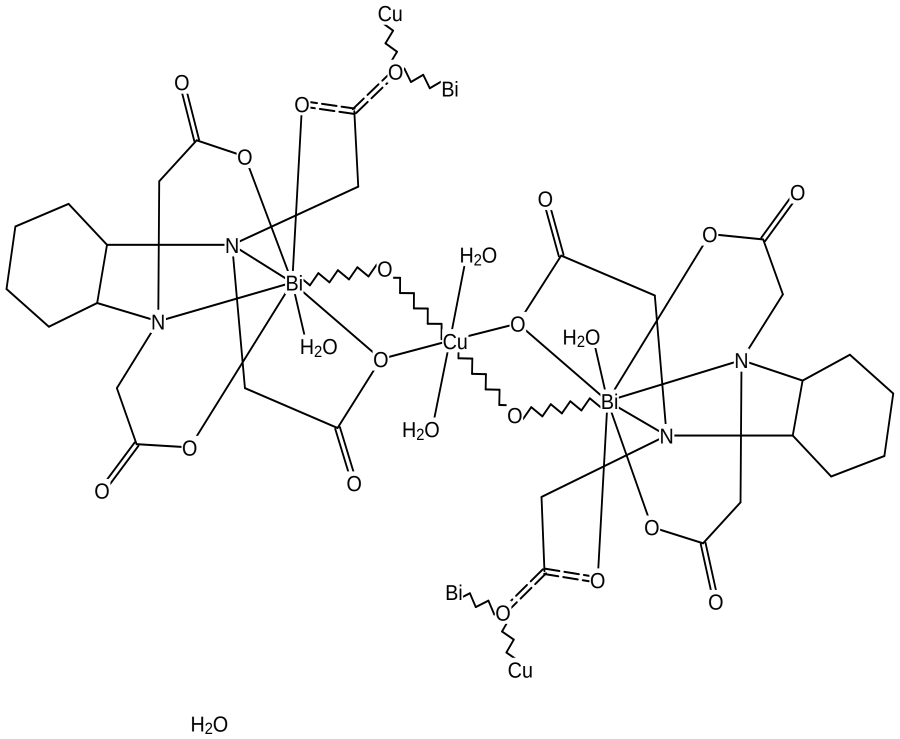

Search: search2 (Wed Jul 26 09:11:25 2017): Hit 207

YUKVOQ

Reference: Ke-Li Hu, M.Kurmoo, Zheming Wang, Song Gao (2009)
Chem.-Eur.J. , **15,** 12050

Formula: $(C_3\ H_3\ Cu_1\ O_6\ ^{1-})n,n(C_1\ H_6\ N_3\ ^{1+})$

Compound Name: catena-(Guanidinium tris(μ_2-formato)-copper(ii))

Space Group:	Pna21	***Cell:***	***a***	8.521(0)	***b***	9.032(0)	***c***	11.350(0)
Space Group No.:	33	***(Å,°)***	α	90.00	β	90.00	γ	90.00

R-Factor (%): 2.80 ***Temperature(K):*** 293 ***Density(g/cm³):*** 1.967

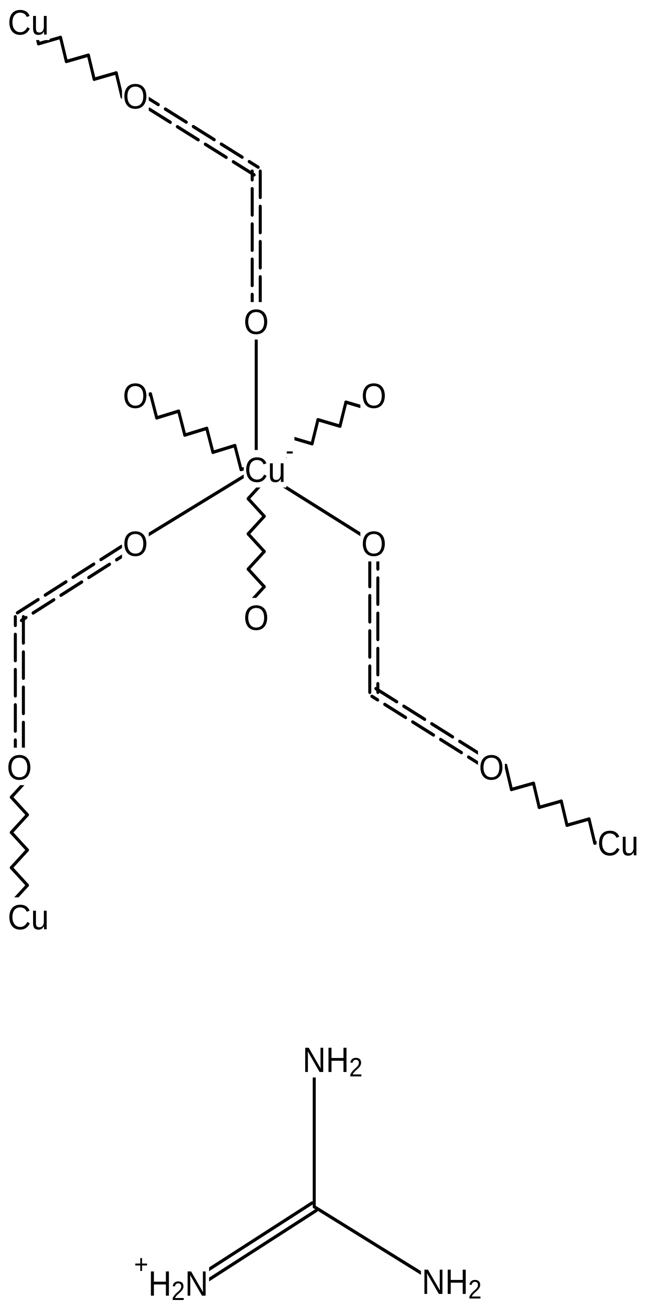

Parameters

Fragment 1

**DIST1 (D)**	1.968
**DIST2 (D)**	1.996
**DIST3 (D)**	1.952
**DIST4 (D)**	2.003
**DIST5 (D)**	2.383
**DIST6 (D)**	2.360

Search: search2 (Wed Jul 26 09:11:25 2017): Hit 208

YUNVIM

Reference:	N.Barba-Behrens, F.Salazar-Garcia, A.M.Bello-Ramirez, E.Garcia-Baez, M.de.J.Rosales-Hoz, R.Contreras, A.Flores-Parra (1994) *Transition Met.Chem.* ,**19,**575								
Formula:	$(C_7 H_{13} Cu_1 N_1 O_{10})n,n(H_2 O_1)$								
Compound Name:	catena-((μ_3-1,3,4,5-Tetrahydroxycyclohexanecarboxylato)-aqua-(nitrato-O)-copper(ii) monohydrate)								
Synonym:	catena-((μ_3-Quinic acid)-aqua-(nitrato-O)-copper(ii) monohydrate)								
Space Group:	P212121	***Cell:***	***a***	9.721(1)	***b***	10.931(1)	***c***	11.646(1)	
Space Group No.:	19	***(Å,°)***	α	90.00	β	90.00	γ	90.00	
R-Factor (%):	2.70	***Temperature(K):***	295	***Density(g/cm³):***	1.893				

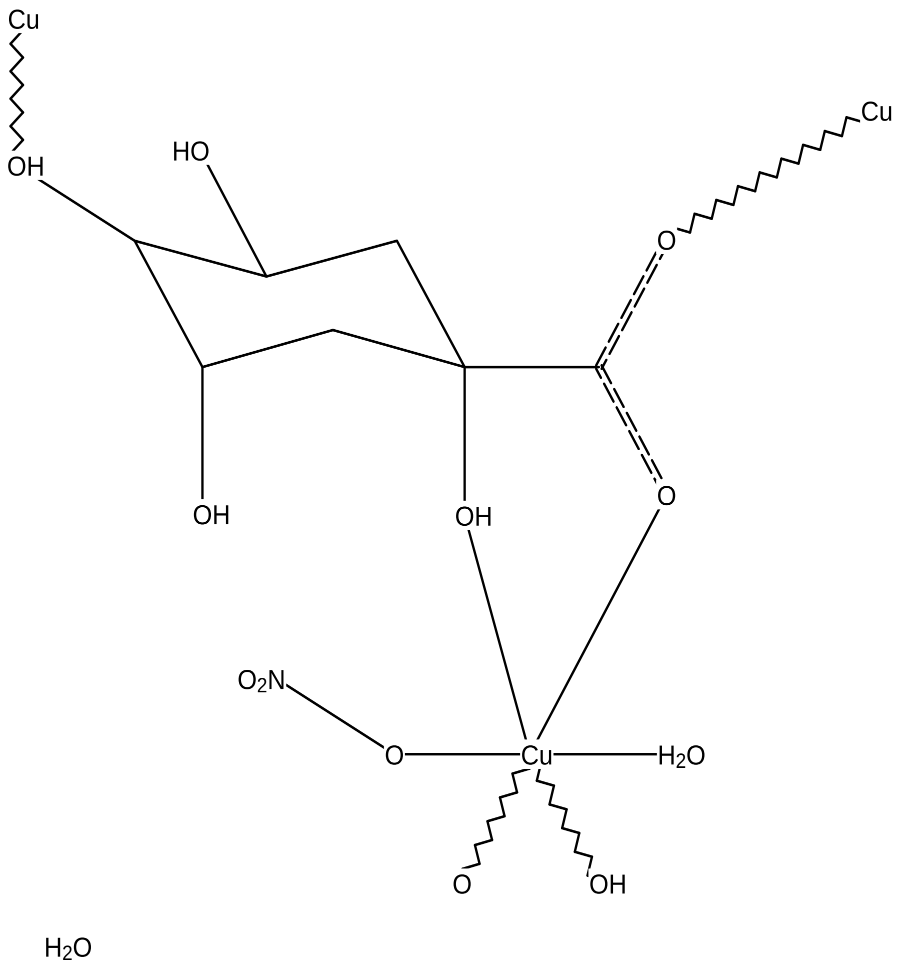

Parameters

Fragment 1

**DIST1 (D)**	1.922
**DIST2 (D)**	1.943
**DIST3 (D)**	1.972
**DIST4 (D)**	1.969
**DIST5 (D)**	2.507
**DIST6 (D)**	2.472

Search: search2 (Wed Jul 26 09:11:25 2017): Hit 209

YURXAM

Reference: J.Landmann, J.A.P.Sprenger, M.Hailmann, V.Bernhardt-Pitchougina, H.Willner, N.Ignat'ev, E.Bernhardt, M.Finze (2015) *Angew.Chem.,Int.Ed.* ,**54,**11259

Formula: $C_{12} H_{36} Cu_1 O_6 S_6{}^{2+}, C_6 B_2 N_6{}^{2-}$

Compound Name: hexakis(dimethyl sulfoxide)-copper(ii) hexacyanodiborate

Space Group:	P-1	***Cell:***	***a***	8.990(1)	***b***	9.580(1)	***c***	9.642(1)
Space Group No.:	2	***(Å,°)***	α	87.55(0)	β	83.57(0)	γ	89.00(0)

R-Factor (%): 2.87 ***Temperature(K):*** 100 ***Density(g/cm³):*** 1.430

Parameters

Fragment 1

**DIST1 (D)**	1.982
**DIST2 (D)**	1.996
**DIST3 (D)**	1.982
**DIST4 (D)**	1.996
**DIST5 (D)**	2.387
**DIST6 (D)**	2.387

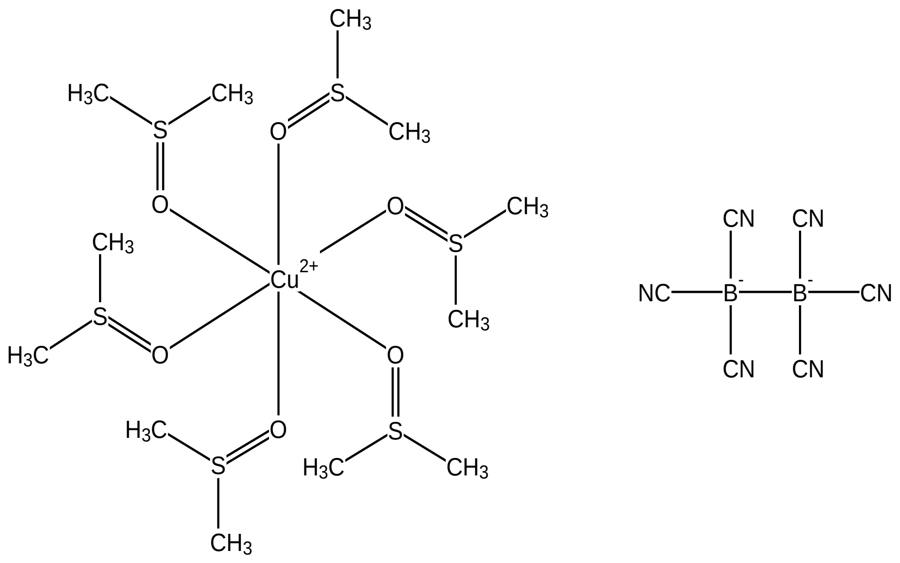

Search: search2 (Wed Jul 26 09:11:25 2017): Hit 210

YUSNIK

Reference: K.V.Waynant, J.D.White, L.Zakharov (2010)
Chem.Commun. ,**46,**5304

Formula: $C_{20} H_{20} Cu_1 O_{10}{}^{2+}$,2($Cl_1 O_4{}^{1-}$)

Compound Name: DIAQUA-BIS(2,7-DIMETHYL-5-OXO-5H-PYRANO(4,3-B)PYRAN-1-IUM-4-OLATO-O,O')-copper(ii) diperchlorate

Space Group:	P21/n	***Cell:***	***a***	9.945(1)	***b***	12.641(1)	***c***	10.459(1)
Space Group No.:	14	***(Å,°)***	α	90.00	β	95.36(0)	γ	90.00

R-Factor (%): 2.38 ***Temperature(K):*** 173 ***Density(g/cm³):*** 1.732

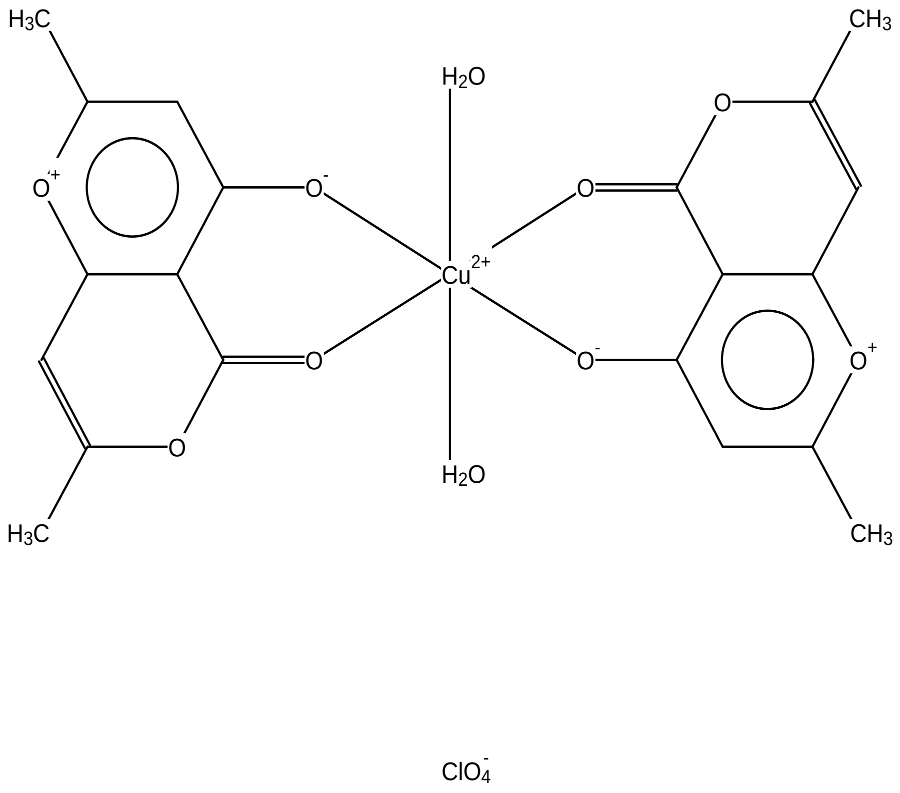

Parameters

Fragment 1

**DIST1 (D)**	1.938
**DIST2 (D)**	2.098
**DIST3 (D)**	1.938
**DIST4 (D)**	2.160
**DIST5 (D)**	2.098
**DIST6 (D)**	2.160

Search: search2 (Wed Jul 26 09:11:25 2017): Hit 211

YUWHII

Reference: T.W.T.Muesmann, A.Mietrach, J.Christoffers, M.S.Wickleder (2010) *Z.Anorg.Allg.Chem.* ,**636,**1307

Formula: $(C_6 H_{18} Cu_2 O_{20} S_4)$n,0.5n$(H_2 O_1)$

Compound Name: catena-((μ_4-Benzene-1,2,4,5-tetrasulfonato)-octa-aqua-di-copper(ii) hemihydrate)

Space Group:	P-1	***Cell:***	***a*** 7.099(1)	***b*** 7.137(1)	***c*** 10.771(1)
Space Group No.:	2	***(Å,°)***	α 98.42(1)	β 102.91(1)	γ 100.69(1)

R-Factor (%): 2.47 ***Temperature(K):*** 293 ***Density(g/cm³):*** 2.185

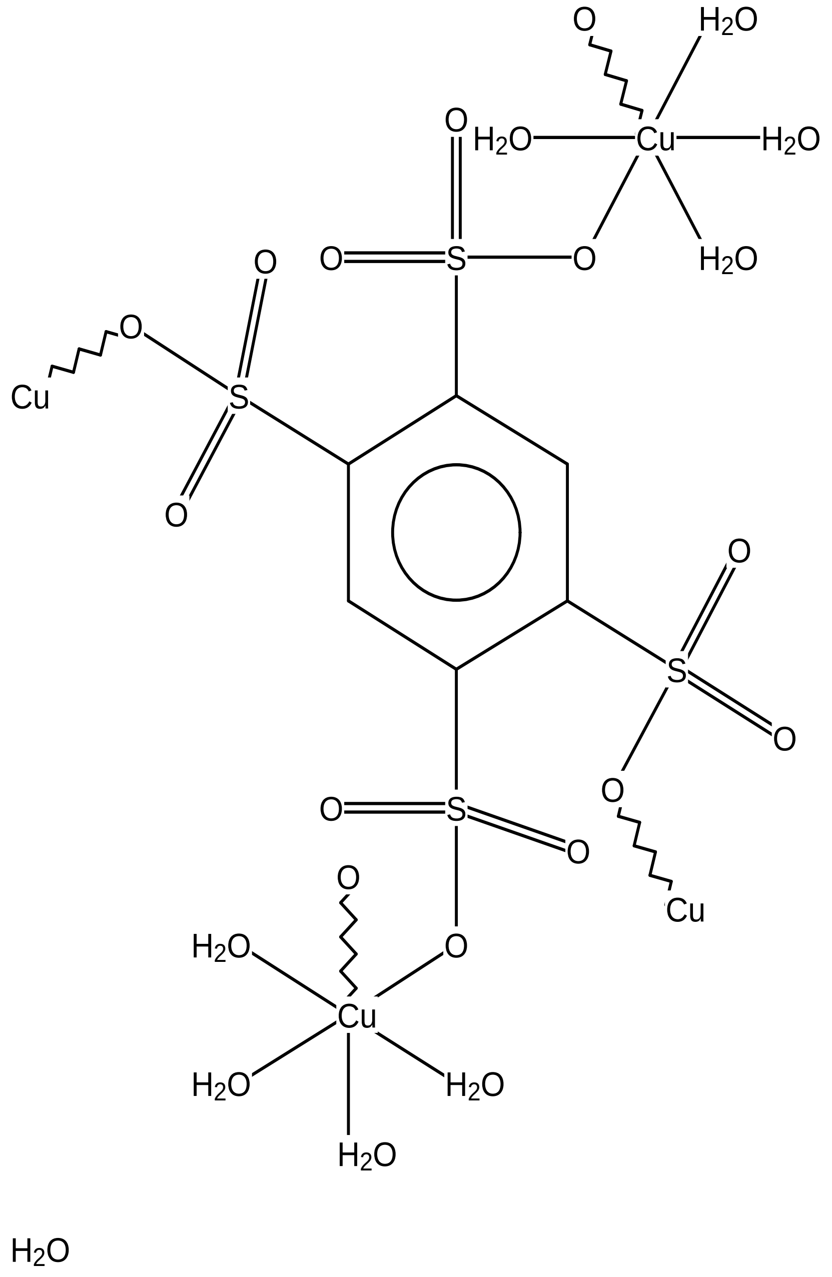

Parameters

Fragment 1

**DIST1 (D)**	1.965
**DIST2 (D)**	1.925
**DIST3 (D)**	1.966
**DIST4 (D)**	1.960
**DIST5 (D)**	2.393
**DIST6 (D)**	2.408

Search: search2 (Wed Jul 26 09:11:25 2017): Hit 212

ZADTEG

Reference:	Haiyan An, Lin Wang, Ying Hu, Tieqi Xu, Yujiao Hou<br>(2016) *Inorg.Chem.* ,**55,**144
Formula:	$H_4\ Co_2\ Mo_{10}\ O_{38}\ ^{6-}$,$H_{12}\ Cu_1\ O_6\ ^{2+}$,4($C_8\ H_8\ N_1\ O_2\ ^{1+}$),5($H_2\ O_1$)
Compound Name:	tetrakis(4-(2-carboxyethenyl)pyridin-1-ium) hexaaqua-copper tetrakis(μ-hydroxo)-tetradecakis(μ-oxo)-icosaoxo-di-cobalt-deca-molybdenum pentahydrate

Space Group:	P1	***Cell:***	***a*** 10.016(2)	***b*** 13.492(2)	***c*** 13.691(2)
Space Group No.:	1	***(Å,°)***	α 104.13(1)	β 94.27(1)	γ 104.85(1)

R-Factor (%):	2.87	***Temperature(K):***	296	***Density(g/cm³):***	2.470

Parameters

Fragment 1

**DIST1 (D)**	1.981
**DIST2 (D)**	2.009
**DIST3 (D)**	1.988
**DIST4 (D)**	2.015
**DIST5 (D)**	2.303
**DIST6 (D)**	2.296

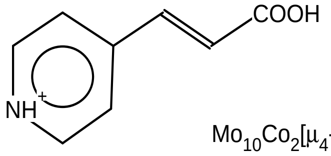

$Mo_{10}Co_2[\mu_4\text{-}O]_6[\mu_3\text{-}O]_4[\mu_2\text{-}HO]_4[\mu_2\text{-}O]_4[O]_{20}^{6-}$

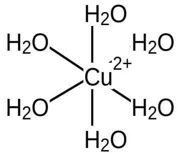

H_2O

Search: search2 (Wed Jul 26 09:11:25 2017): Hit 213

ZEHLED

Reference: C.Vansant, H.O.Desseyn, V.Tangoulis, C.P.Raptopoulou, A.Terzis, S.P.Perlepes (1995) *Polyhedron* ,**14,**2115

Formula: $C_6 H_{12} Cu_1 N_2 O_8$

Compound Name: trans-Diaqua-bis(malonamato-O,O')-copper(ii)

Space Group:	P21/n	***Cell:***	***a***	7.068(1)	***b***	7.502(1)	***c***	9.682(1)
Space Group No.:	14	***(Å,°)***	α	90.00	β	100.90(1)	γ	90.00

R-Factor (%): 1.90 ***Temperature(K):*** 295 ***Density(g/cm³):*** 2.001

Parameters

Fragment 1

**DIST1 (D)**	1.966
**DIST2 (D)**	1.934
**DIST3 (D)**	1.966
**DIST4 (D)**	1.934
**DIST5 (D)**	2.480
**DIST6 (D)**	2.480

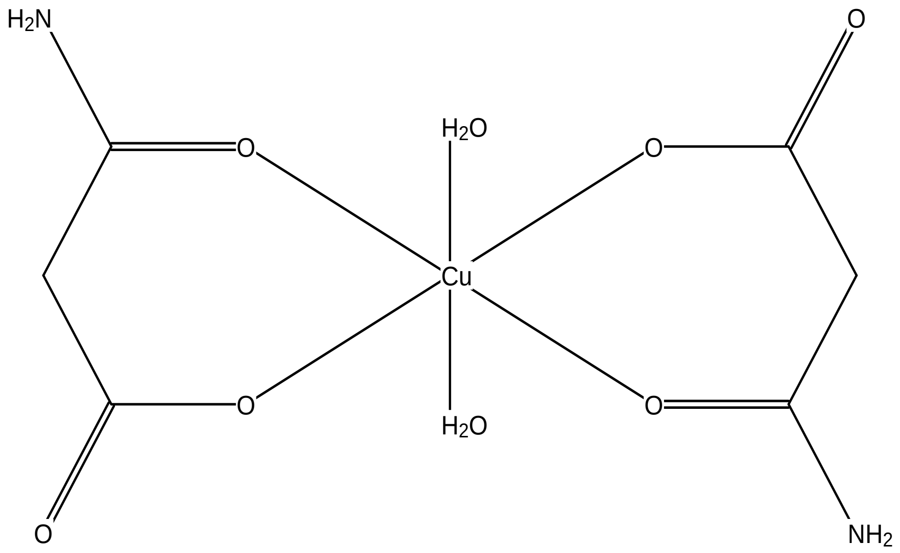

Search: search2 (Wed Jul 26 09:11:25 2017): Hit 214

ZUCYON

Reference: F.Behler, M.S.Wickleder, J.Christoffers (2015) *ARKIVOC* , **16,**64-2

Formula: $C_{12} H_{26} Cu_2 O_{22} S_4$,2($H_2 O_1$)

Compound Name: decaaqua-(μ-biphenyl-3,3',5,5'-tetrasulfonato)-di-copper(ii) dihydrate

Space Group:	P21/c	***Cell:***	***a*** 5.842(0)	***b*** 11.265(0)	***c*** 20.995(1)		
Space Group No.:	14	***(Å,°)***	α 90.00	β 97.50(0)	γ 90.00		

R-Factor (%): 2.37 ***Temperature(K):*** 120 ***Density(g/cm^3):*** 1.973

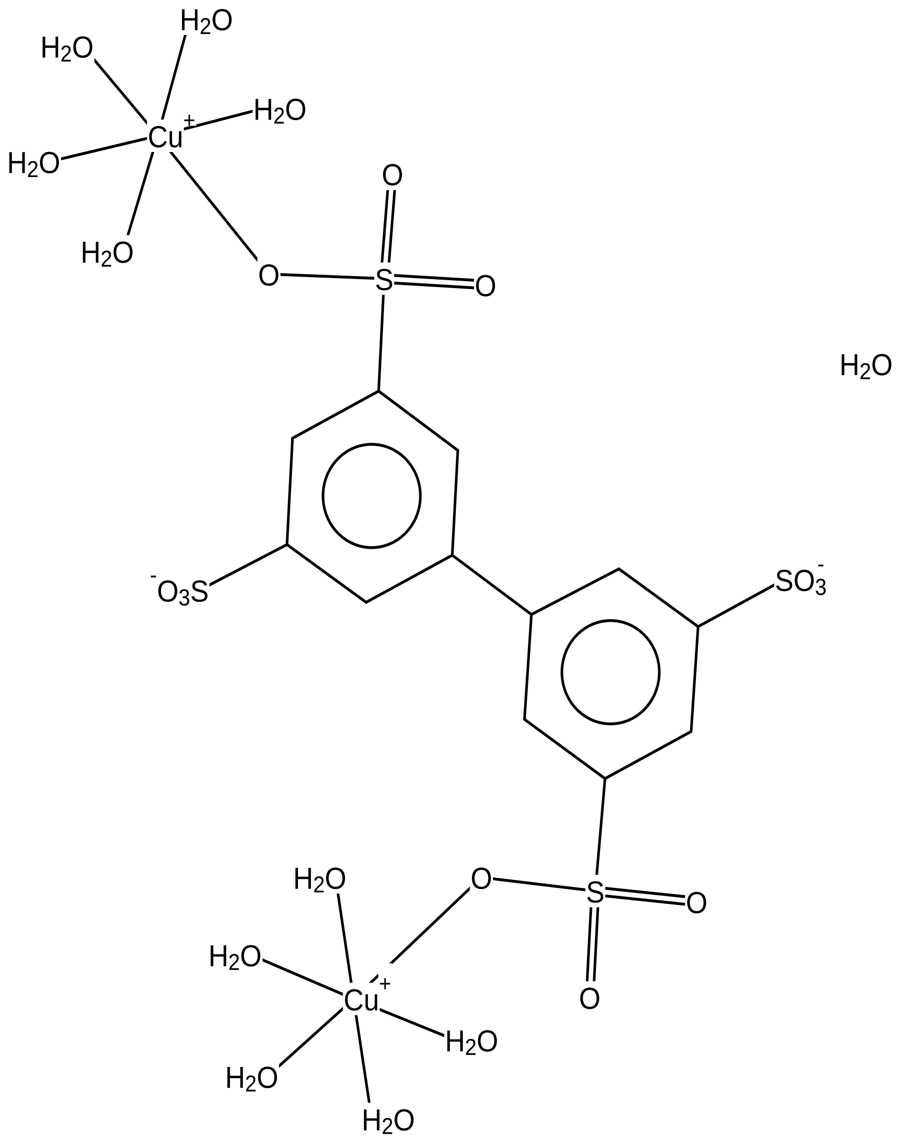

Parameters

Fragment 1

**DIST1 (D)**	1.940
**DIST2 (D)**	1.963
**DIST3 (D)**	1.971
**DIST4 (D)**	1.963
**DIST5 (D)**	2.334
**DIST6 (D)**	2.405

Search: search2 (Wed Jul 26 09:11:25 2017): Hit 215

DAMTOD

Reference:	A.I.Nicasio, F.Montilla, E.Alvarez, R.P.Colodrero, A.Galindo (2017) *Dalton Trans.* ,**46,**471
Formula:	$(C_{18} H_{22} Cu_1 N_4 O_8)n$
Compound Name:	catena-(bis(μ-(S,S)-2-(3-(1-carboxylatoethyl)-1H-imidazol-3-ium-1-yl) propanoato)-copper)

Space Group:	P21212	***Cell:***	***a*** 13.706(1)	***b*** 14.275(1)	***c*** 10.326(0)
Space Group No.:	18	***(Å,°)***	α 90.00	β 90.00	γ 90.00

R-Factor (%): 1.92 ***Temperature(K):*** 193 ***Density(g/cm^3):*** 1.598

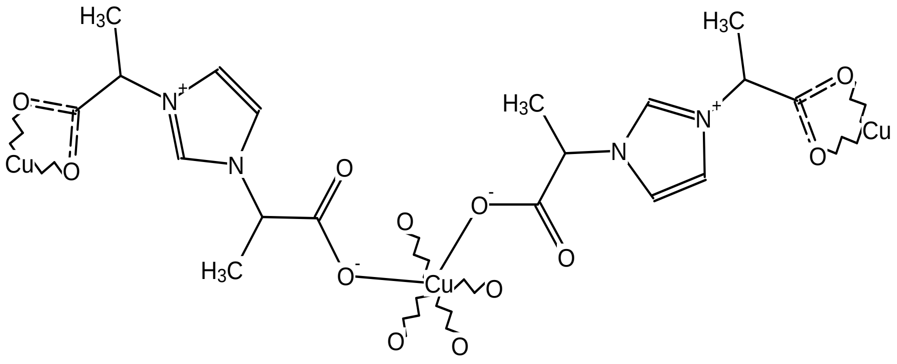

Parameters

Fragment 1

**DIST1 (D)**	1.973
**DIST2 (D)**	1.952
**DIST3 (D)**	1.952
**DIST4 (D)**	1.973
**DIST5 (D)**	2.582
**DIST6 (D)**	2.582

Fragment 2

**DIST1 (D)**	2.008
**DIST2 (D)**	1.960
**DIST3 (D)**	1.960
**DIST4 (D)**	2.008
**DIST5 (D)**	2.629
**DIST6 (D)**	2.629

Search: search2 (Wed Jul 26 09:11:25 2017): Hit 216

IYODEH

Reference: Bowen Cong, Zhanhua Su, Zhifeng Zhao, Wenqi Zhao, Lin Xia, Baibin Zhou (2016) *Polyhedron* ,

Formula: $4(C_7 H_{11} N_2{}^{1+}),As_6 Cu_1 Mo_6 O_{30}{}^{4-}$

Compound Name: tetrakis(4-(dimethylamino)pyridinium) octadecakis(μ-oxo)-dodeca-oxo-hexa-arsenic(iii)-copper(ii)-hexa-molybdenum(vi)

Space Group:	P21/c	***Cell:***	***a***	9.391(0)	***b***	19.964(1)	***c***	14.837(1)
Space Group No.:	14	***(Å,°)***	α	90.00	β	95.98(0)	γ	90.00

R-Factor (%): 2.74 ***Temperature(K):*** 296 ***Density(g/cm^3):*** 2.475

Parameters

Fragment 1

**DIST1 (D)**	2.057
**DIST2 (D)**	1.992
**DIST3 (D)**	2.057
**DIST4 (D)**	1.992
**DIST5 (D)**	2.201
**DIST6 (D)**	2.201

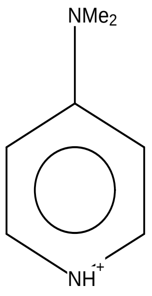

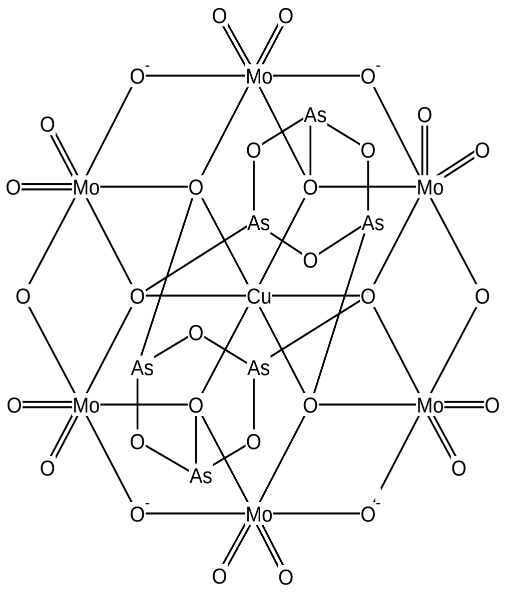

Search: search2 (Wed Jul 26 09:11:25 2017): Hit 217

IZAHUO

Reference:	S.Adhikari, A.Sahana, B.Kumari, Durba Ganguly, S.Das, P.P.Banerjee, G.Banerjee, A.Chattopadhyay, M.Fondo, J.S.Matalobos, P.Brandao, V.Felix, D.Das (2016) *New J.Chem.* ,**40,**10378
Formula:	$C_{14} H_{15} Cu_1 N_2 O_{11} S_1$,2($H_2 O_1$)
Compound Name:	aqua-(6-methyl-2-(oxo)-1,2-dihydropyridine-3-carboxylato)-(6-methyl-2-(oxo)-1,2-dihydropyridine-3-carboxylic acid)-(sulfato)-copper dihydrate

Space Group:	P21	***Cell:***	***a***	8.773(0)	***b***	13.745(0)	***c***	16.607(0)
Space Group No.:	4	***(Å,°)***	α	90.00	β	93.65(0)	γ	90.00

R-Factor (%):	2.55	***Temperature(K):***	100	***Density(g/cm³):***	1.725

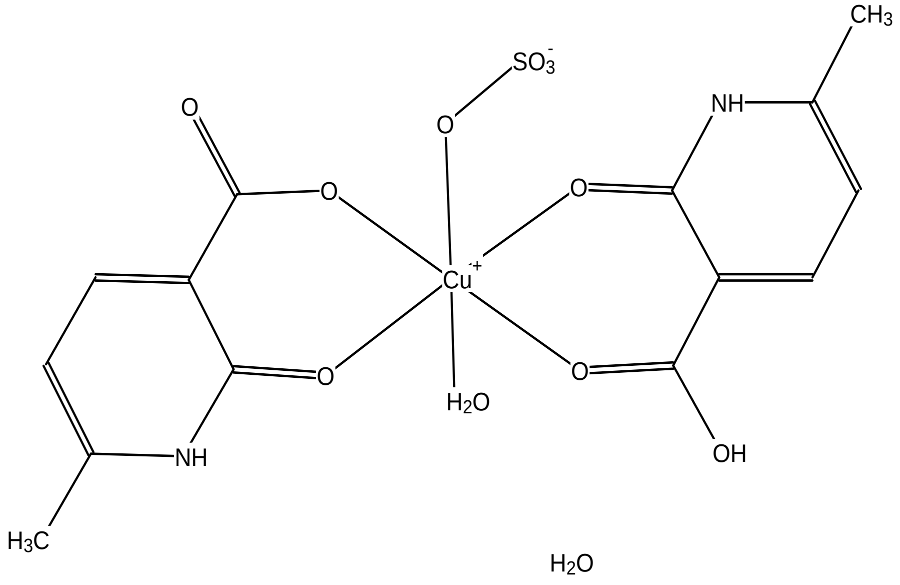

Parameters

Fragment 1

**DIST1 (D)**	1.951
**DIST2 (D)**	1.960
**DIST3 (D)**	1.947
**DIST4 (D)**	1.926
**DIST5 (D)**	2.308
**DIST6 (D)**	2.562

Fragment 2

**DIST1 (D)**	1.938
**DIST2 (D)**	1.937
**DIST3 (D)**	1.959
**DIST4 (D)**	1.937
**DIST5 (D)**	2.269
**DIST6 (D)**	2.596

Search: search2 (Wed Jul 26 09:11:25 2017): Hit 218

IZERIQ

Reference: D.A.Dickie, R.A.Kemp (2016)
Acta Crystallogr.,Sect.E:Cryst.Commun. ,**72,**1780

Formula: $(C_4\ Cu_1\ O_8\ ^{2-})n,2(H_4\ N_1\ ^{1+})$

Compound Name: catena-(bis(ammonium) bis(μ-oxalato)-copper(ii))

Space Group:	P21/c	***Cell:***	***a*** 4.856(0)	***b*** 13.519(0)	***c*** 6.721(0)		
Space Group No.:	14	***(Å,°)***	α 90.00	β 96.99(0)	γ 90.00		

R-Factor (%): 1.79 ***Temperature(K):*** 100 ***Density(g/cm³):*** 2.090

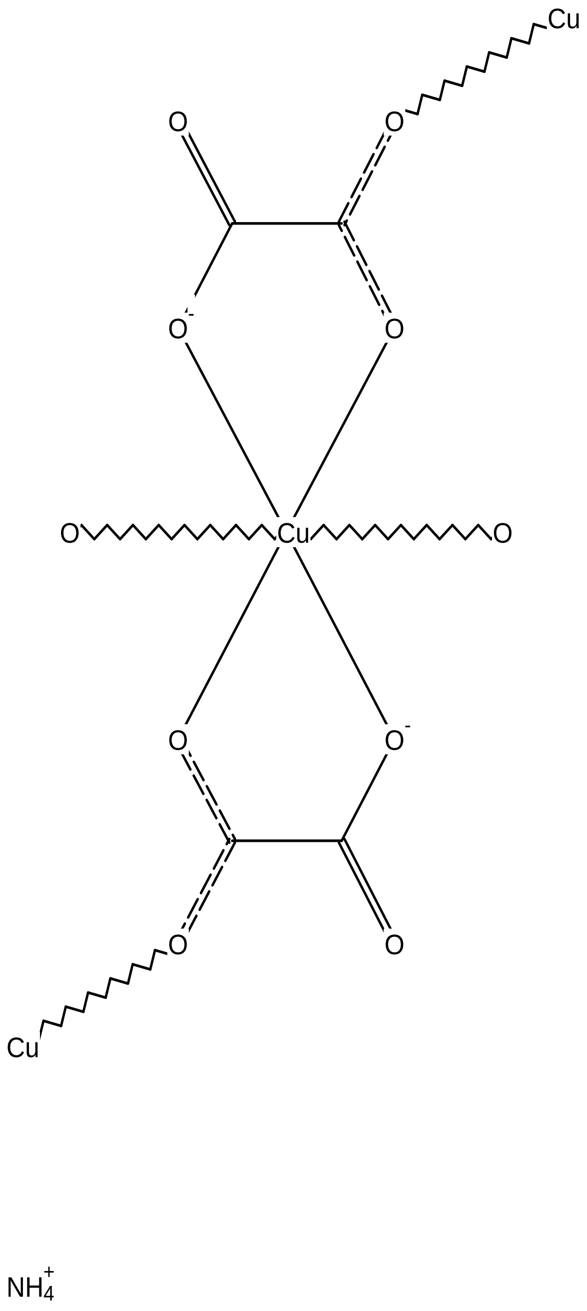

Parameters

Fragment 1

**DIST1 (D)**	1.933
**DIST2 (D)**	1.930
**DIST3 (D)**	1.933
**DIST4 (D)**	1.930
**DIST5 (D)**	2.706
**DIST6 (D)**	2.706

Search: search2 (Wed Jul 26 09:11:25 2017): Hit 219

TEMPIM01

Reference: Zhang Bin (2016)
CSD Communication(Private Communication) ,

Formula: $2(C_4 H_{12} N_1{}^{1+}),(C_4 Cu_1 O_8{}^{2-})n,H_2 O_1$

Compound Name: catena-[bis(tetramethylammonium) (μ-oxalato)-(oxalato)-copper monohydrate]

Space Group:	Pbca	***Cell:***	***a***	11.246(0)	***b***	10.139(0)	***c***	30.992(0)
Space Group No.:	61	***(Å,°)***	α	90.00	β	90.00	γ	90.00

R-Factor (%): 2.73 ***Temperature(K):*** 293 ***Density(g/cm^3):*** 1.526

Parameters

Fragment 1

**DIST1 (D)**	1.968
**DIST2 (D)**	1.954
**DIST3 (D)**	1.981
**DIST4 (D)**	1.975
**DIST5 (D)**	2.319
**DIST6 (D)**	2.432

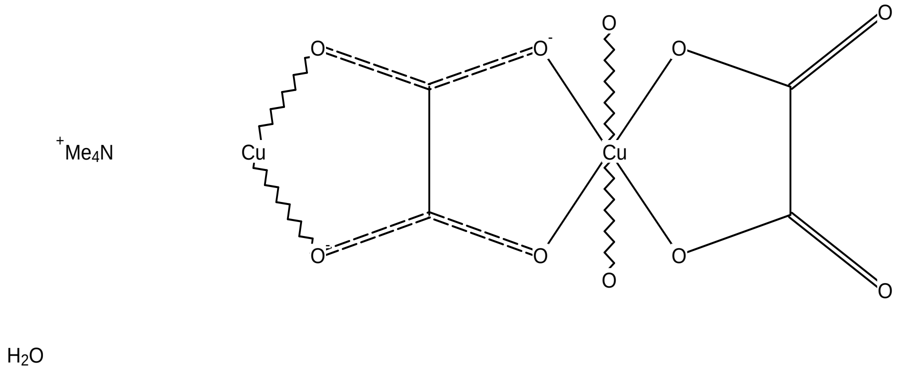

Search: search2 (Wed Jul 26 09:11:25 2017): Hit 220

UCADEK

Reference: Wenjiao Yao, A.Robert Armstrong, Philip Lightfoot (2016)
Z.Anorg.Allg.Chem. ,**642,**1345

Formula: $(C_4\ Cu_1\ Na_2\ O_8)n$

Compound Name: catena-[bis(μ-oxalato)-copper(ii)-di-sodium(i)]

Space Group:	P21/n	***Cell:***	***a***	7.258(0)	***b***	5.771(0)	***c***	8.660(0)
Space Group No.:	14	***(Å,°)***	α	90.00	β	106.95(0)	γ	90.00

R-Factor (%): 2.00 ***Temperature(K):*** 173 ***Density(g/cm³):*** 2.733

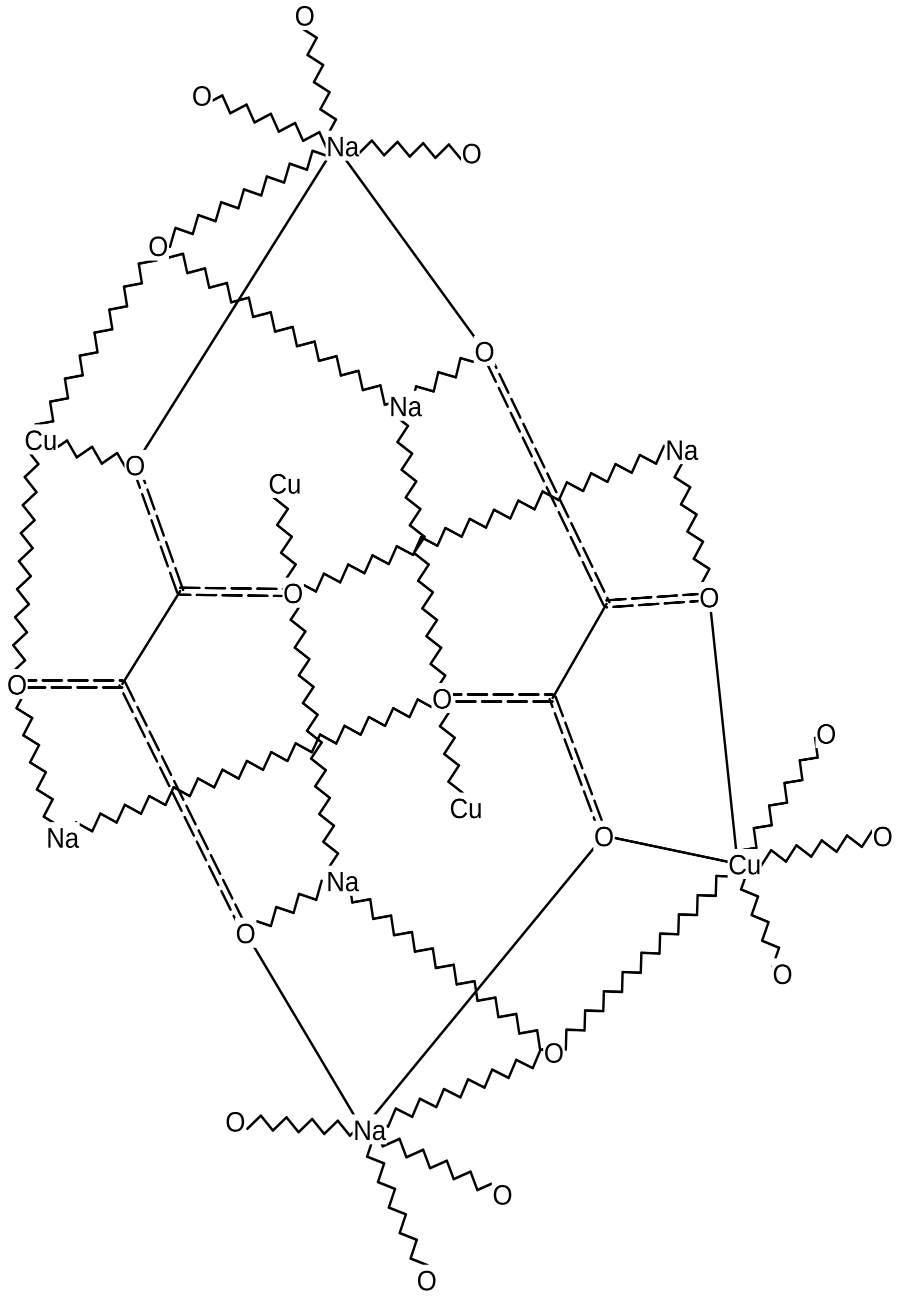

Parameters

Fragment 1

**DIST1 (D)**	1.939
**DIST2 (D)**	1.962
**DIST3 (D)**	1.939
**DIST4 (D)**	1.962
**DIST5 (D)**	2.652
**DIST6 (D)**	2.652

Search: search2 (Wed Jul 26 09:11:25 2017): Hit 221

UXETAU

Reference:	A.I.Vinokur, I.A.Guzei (2016)<br>*CSD Communication(Private Communication)* ,
Formula:	$(H_{16}\ Cu_2\ O_{16}\ S_2)n,2(H_2\ O_1)$
Compound Name:	catena-[bis(μ-sulfato)-octa-aquadi-copper(ii) dihydrate]

Space Group:	P-1	***Cell:***	***a*** 5.963(1)	***b*** 6.084(1)	***c*** 10.623(2)		
Space Group No.:	2	***(Å,°)***	α 77.23(3)	β 82.56(3)	γ 72.50(3)		
R-Factor (%):	2.77	***Temperature(K):***	100	***Density(g/cm^3):***	2.318		

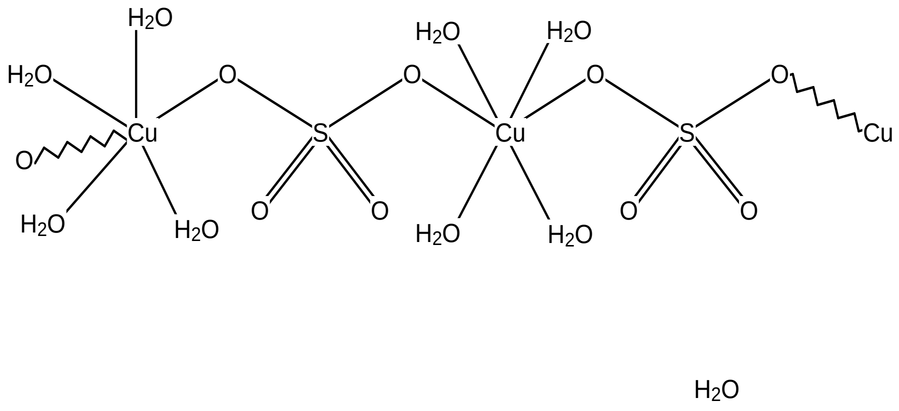

Parameters

Fragment 1

**DIST1 (D)**	1.928
**DIST2 (D)**	1.963
**DIST3 (D)**	1.928
**DIST4 (D)**	1.963
**DIST5 (D)**	2.403
**DIST6 (D)**	2.403

Fragment 2

**DIST1 (D)**	1.970
**DIST2 (D)**	1.962
**DIST3 (D)**	1.970
**DIST4 (D)**	1.962
**DIST5 (D)**	2.363
**DIST6 (D)**	2.363

Search: search2 (Wed Jul 26 09:11:25 2017): Hit 222

UXISAX

Reference: S.Yoneyama, T.Kodama, K.Kikuchi, T.Fujisawa, A.Yamaguchi, A.Sumiyama, Yoshiaki Shuku, Shinobu Aoyagi, W.Fujita (2016) *Dalton Trans.* ,**45,**16774

Formula: $(C_4 H_6 Cu_1 O_6)n$

Compound Name: catena-[bis(μ-glycolato)-copper(ii)]

Space Group:	P21/n	***Cell:***	***a***	5.185(1)	***b***	7.252(1)	***c***	8.875(3)
Space Group No.:	14	***(Å,°)***	α	90.00	β	100.86(0)	γ	90.00

R-Factor (%): 2.13 ***Temperature(K):*** 173 ***Density(g/cm^3):*** 2.165

Parameters

Fragment 1

**DIST1 (D)**	1.928
**DIST2 (D)**	1.966
**DIST3 (D)**	1.928
**DIST4 (D)**	1.966
**DIST5 (D)**	2.435
**DIST6 (D)**	2.435

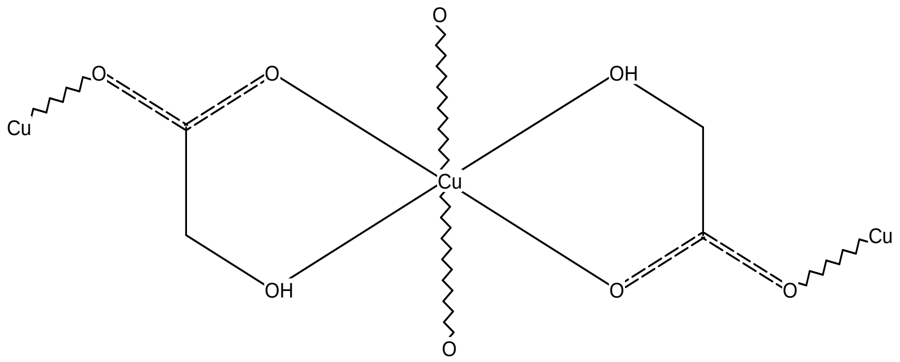

Search: search2 (Wed Jul 26 09:11:25 2017): Hit 223

UXISAX01

Reference:	S.Yoneyama, T.Kodama, K.Kikuchi, T.Fujisawa, A.Yamaguchi, A.Sumiyama, Yoshiaki Shuku, Shinobu Aoyagi, W.Fujita (2016) *Dalton Trans.* ,**45,**16774
Formula:	$(C_4 H_6 Cu_1 O_6)n$
Compound Name:	catena-[bis(μ-glycolato)-copper(ii)]

Space Group:	P21/n	***Cell:***	***a*** 5.079(0)	***b*** 8.692(0)	***c*** 7.705(0)		
Space Group No.:	14	***(Å,°)***	α 90.00	β 107.34(0)	γ 90.00		
R-Factor (%):	2.28	***Temperature(K):***	230	***Density(g/cm³):***	2.185		

Parameters

Fragment 1

**DIST1 (D)**	1.922
**DIST2 (D)**	1.932
**DIST3 (D)**	1.922
**DIST4 (D)**	1.932
**DIST5 (D)**	2.522
**DIST6 (D)**	2.522

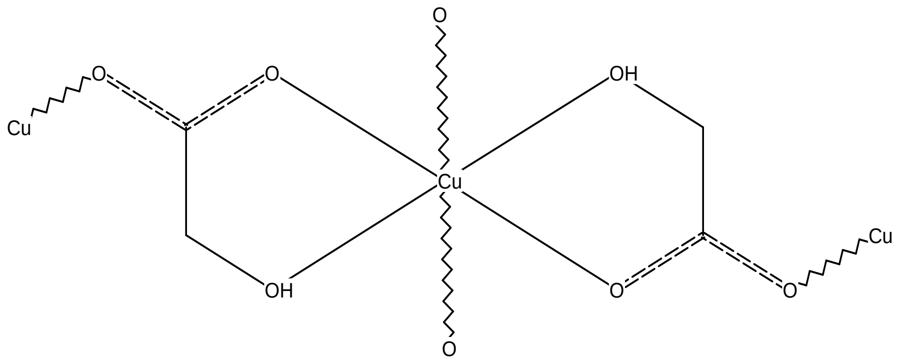

Search: search2 (Wed Jul 26 09:11:25 2017): Hit 224

UXISAX02

Reference: S.Yoneyama, T.Kodama, K.Kikuchi, T.Fujisawa, A.Yamaguchi, A.Sumiyama, Yoshiaki Shuku, Shinobu Aoyagi, W.Fujita (2016) *Dalton Trans.* ,**45,**16774

Formula: $(C_4 H_6 Cu_1 O_6)n$

Compound Name: catena-[bis(μ-glycolato)-copper(ii)]

Space Group:	P21/n	***Cell:***	***a***	5.201(4)	***b***	7.356(5)	***c***	8.839(6)
Space Group No.:	14	***(Å,°)***	α	90.00	β	101.17(0)	γ	90.00

R-Factor (%): 2.68 ***Temperature(K):*** 290 ***Density(g/cm^3):*** 2.139

Parameters

Fragment 1

**DIST1 (D)**	1.926
**DIST2 (D)**	1.960
**DIST3 (D)**	1.926
**DIST4 (D)**	1.960
**DIST5 (D)**	2.439
**DIST6 (D)**	2.439

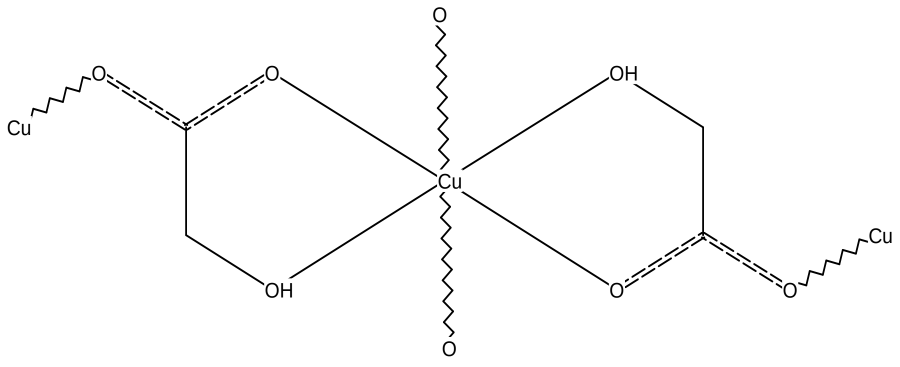

Search: search2 (Wed Jul 26 09:11:25 2017): Hit 225

UXISAX04

Reference: S.Yoneyama, T.Kodama, K.Kikuchi, T.Fujisawa, A.Yamaguchi, A.Sumiyama, Yoshiaki Shuku, Shinobu Aoyagi, W.Fujita (2016) *Dalton Trans.* ,**45,**16774

Formula: $(C_4 H_6 Cu_1 O_6)n$

Compound Name: catena-[bis(μ-glycolato)-copper(ii)]

Space Group:	P21/n	***Cell:***	***a***	5.101(3)	***b***	8.673(5)	***c***	7.747(5)
Space Group No.:	14	***(Å,°)***	α	90.00	β	106.93(0)	γ	90.00

R-Factor (%): 2.81 ***Temperature(K):*** 290 ***Density(g/cm³):*** 2.164

Parameters

Fragment 1

**DIST1 (D)**	1.919
**DIST2 (D)**	1.932
**DIST3 (D)**	1.919
**DIST4 (D)**	1.932
**DIST5 (D)**	2.538
**DIST6 (D)**	2.538

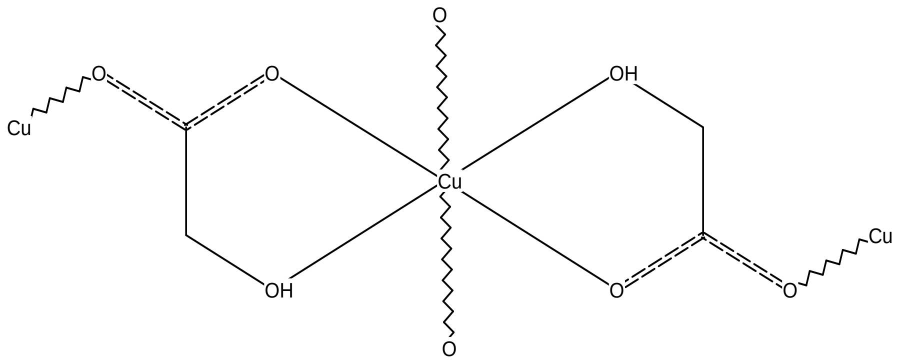

Search: search2 (Wed Jul 26 09:11:25 2017): Hit 226

UXISAX05

Reference: S.Yoneyama, T.Kodama, K.Kikuchi, T.Fujisawa, A.Yamaguchi, A.Sumiyama, Yoshiaki Shuku, Shinobu Aoyagi, W.Fujita (2016) *Dalton Trans.* ,**45,**16774

Formula: $(C_4 H_6 Cu_1 O_6)n$

Compound Name: catena-[bis(μ-glycolato)-copper(ii)]

Space Group:	P21/n	***Cell:***	***a***	5.110(6)	***b***	8.680(1)	***c***	7.748(9)
Space Group No.:	14	***(Å,°)***	α	90.00	β	106.73(1)	γ	90.00

R-Factor (%): 2.64 ***Temperature(K):*** 290 ***Density(g/cm³):*** 2.156

Parameters

Fragment 1

**DIST1 (D)**	1.917
**DIST2 (D)**	1.936
**DIST3 (D)**	1.917
**DIST4 (D)**	1.936
**DIST5 (D)**	2.542
**DIST6 (D)**	2.542

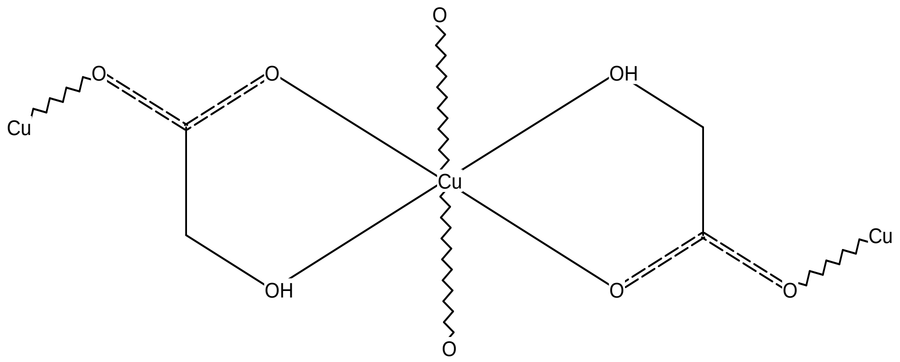

Search: search2 (Wed Jul 26 09:11:25 2017): Hit 227

UXISAX06

Reference: S.Yoneyama, T.Kodama, K.Kikuchi, T.Fujisawa, A.Yamaguchi, A.Sumiyama, Yoshiaki Shuku, Shinobu Aoyagi, W.Fujita (2016) *Dalton Trans.* ,**45,**16774

Formula: $(C_4 H_6 Cu_1 O_6)n$

Compound Name: catena-[bis(μ-glycolato)-copper(ii)]

Space Group:	P21/n	***Cell:***	***a***	5.108(2)	***b***	8.677(3)	***c***	7.755(3)
Space Group No.:	14	***(Å,°)***	α	90.00	β	106.81(0)	γ	90.00

R-Factor (%): 2.65 ***Temperature(K):*** 290 ***Density(g/cm^3):*** 2.156

Parameters

Fragment 1

**DIST1 (D)**	1.919
**DIST2 (D)**	1.935
**DIST3 (D)**	1.919
**DIST4 (D)**	1.935
**DIST5 (D)**	2.539
**DIST6 (D)**	2.539

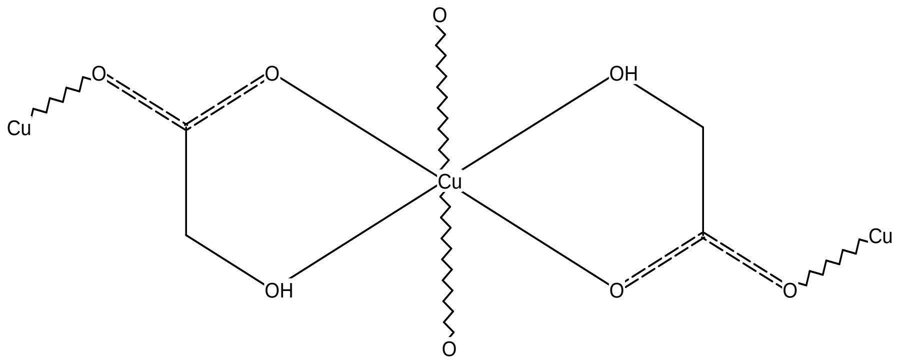

Search: search2 (Wed Jul 26 09:11:25 2017): Hit 228

HARKOD

Reference:	F.Zhang, B.-G.Zhang (2017)<br>*Acta Crystallogr.,Sect.E:Cryst.Commun.* ,**73,**835
Formula:	$(C_{18}\ H_{10}\ Ca_2\ Cu_1\ O_{14})n$
Compound Name:	catena-[bis(μ-benzene-1,3,5-tricarboxylato)-diaqua-di-calcium(ii)-copper(ii)]

Space Group:	P-1	***Cell:***	***a*** 6.664(3)	***b*** 8.754(4)	***c*** 8.925(4)		
Space Group No.:	2	***(Å,°)***	α 103.06(0)	β 110.14(0)	γ 92.78(0)		
R-Factor (%):	2.87	***Temperature(K):***	296	***Density(g/cm³):***	2.091		

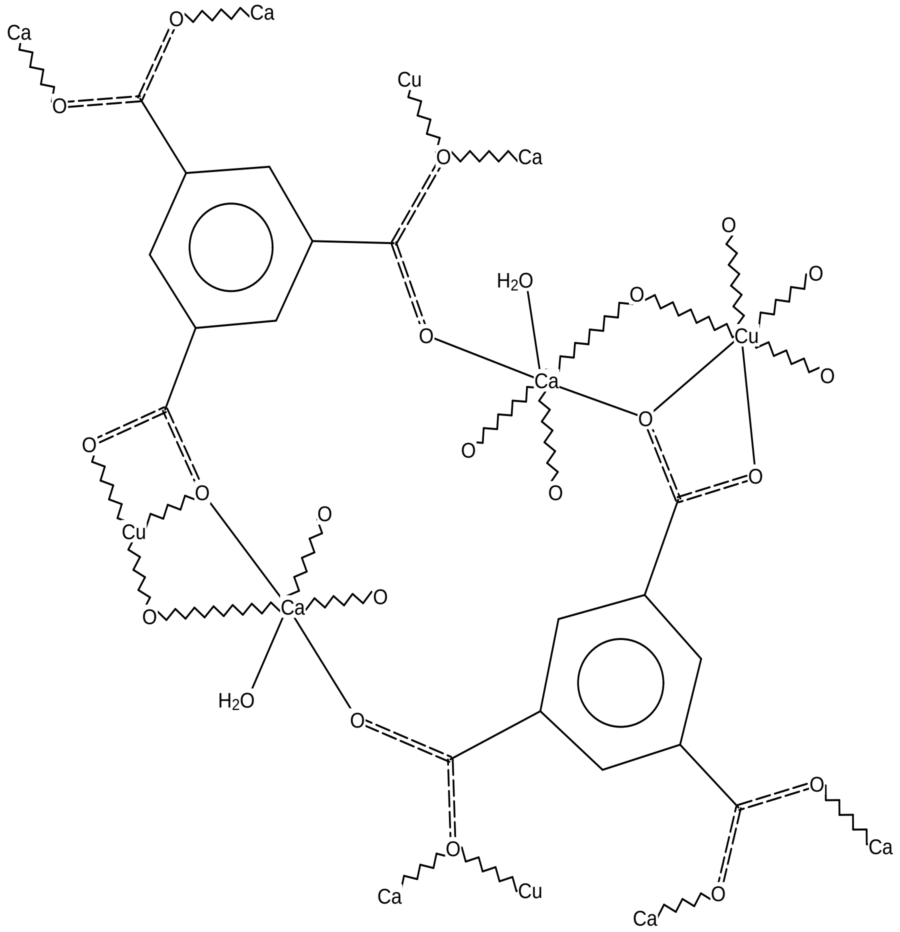

Parameters

Fragment 1

**DIST1 (D)**	1.980
**DIST2 (D)**	1.943
**DIST3 (D)**	1.943
**DIST4 (D)**	1.980
**DIST5 (D)**	2.770
**DIST6 (D)**	2.770

Search: search2 (Wed Jul 26 09:11:25 2017): Hit 229

KARHOD

Reference: G.Smith, U.D.Wermuth (2017)
Acta Crystallogr.,Sect.C:Cryst.Struct.Chem. ,**73,**325

Formula: $2(C_6 H_9 As_1 N_1 O_3{}^{1+}),H_{12} Cu_1 O_6{}^{2+},2(O_4 S_1{}^{2-}),2(H_2 O_1)$

Compound Name: hexaaqua-copper(ii) bis(4-arsonoanilinium) disulfate dihydrate

Space Group:	P21/c	***Cell:***	***a*** 15.078(0)	***b*** 11.230(0)	***c*** 8.676(0)		
Space Group No.:	14	***(Å,°)***	α 90.00	β 99.31(0)	γ 90.00		

R-Factor (%): 2.74 ***Temperature(K):*** 200 ***Density(g/cm³):*** 1.915

Parameters

Fragment 1

**DIST1 (D)**	1.946
**DIST2 (D)**	2.013
**DIST3 (D)**	1.946
**DIST4 (D)**	2.013
**DIST5 (D)**	2.270
**DIST6 (D)**	2.270

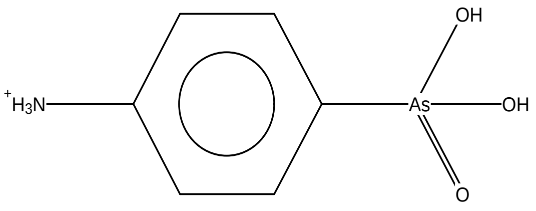

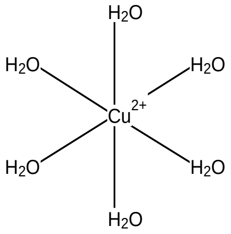

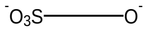

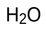

Search: search2 (Wed Jul 26 09:11:25 2017): Hit 230

KEDJAG04

Reference: Zhang Bin (2017)
CSD Communication(Private Communication) ,

Formula: $(C_{12}\ Cu_4\ O_{24}\ ^{4-})n,4(C_6\ H_{16}\ N_1\ ^{1+})$

Compound Name: catena-(tetrakis(Triethylammonium) hexakis(μ_2-oxalato)-tetra-copper)

Space Group:	P21/c	***Cell:***	***a*** 8.682(0)	***b*** 34.180(0)	***c*** 8.582(0)
Space Group No.:	14	***(Å,°)***	α 90.00	β 107.60(0)	γ 90.00

R-Factor (%): 2.85 ***Temperature(K):*** 93 ***Density(g/cm^3):*** 1.630

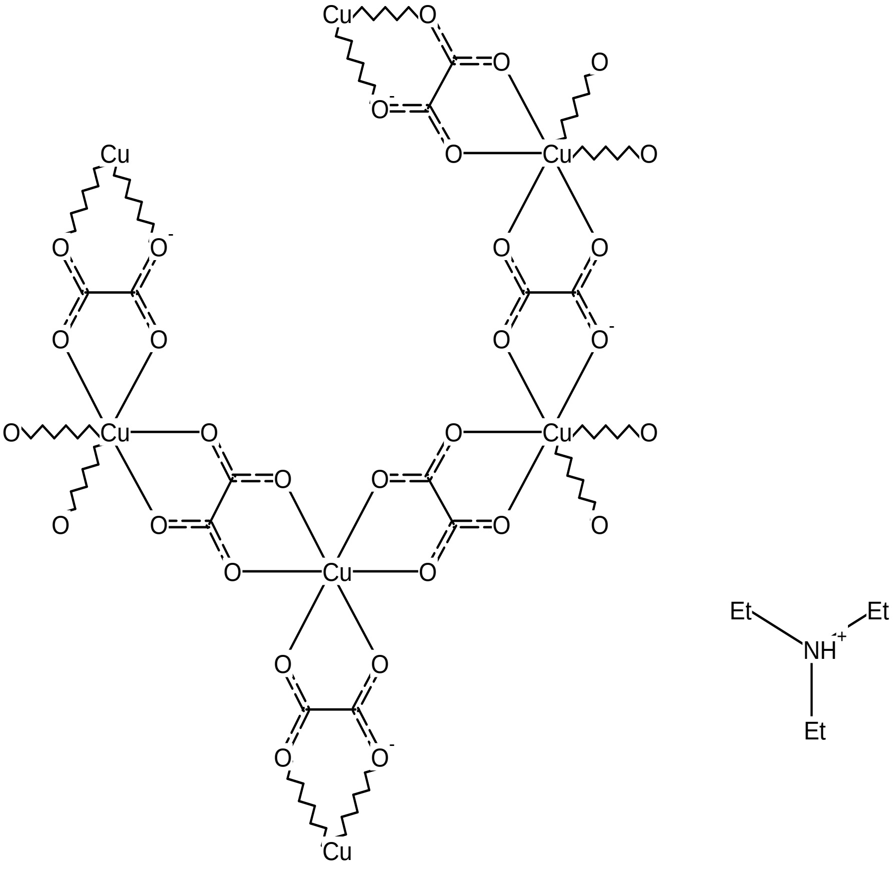

Parameters

Fragment 1

**DIST1 (D)**	1.992
**DIST2 (D)**	1.986
**DIST3 (D)**	1.957
**DIST4 (D)**	1.991
**DIST5 (D)**	2.258
**DIST6 (D)**	2.318

Fragment 2

**DIST1 (D)**	1.976
**DIST2 (D)**	1.997
**DIST3 (D)**	1.955
**DIST4 (D)**	1.995
**DIST5 (D)**	2.228
**DIST6 (D)**	2.331

Search: search2 (Wed Jul 26 09:11:25 2017): Hit 231

KEDJAG10

Reference: Zhang Bin (2017)
CSD Communication(Private Communication) ,

Formula: $(C_{12}\,Cu_4\,O_{24}{}^{4-})n,4(C_6\,H_{16}\,N_1{}^{1+})$

Compound Name: catena-(tetrakis(Triethylammonium) hexakis(μ_2-oxalato)-tetra-copper)

Space Group:	P21/c	***Cell:***	***a*** 8.716(0)	***b*** 34.081(0)	***c*** 8.636(0)
Space Group No.:	14	***(Å,°)***	α 90.00	β 107.63(0)	γ 90.00

R-Factor (%): 2.90 ***Temperature(K):*** 155 ***Density(g/cm³):*** 1.618

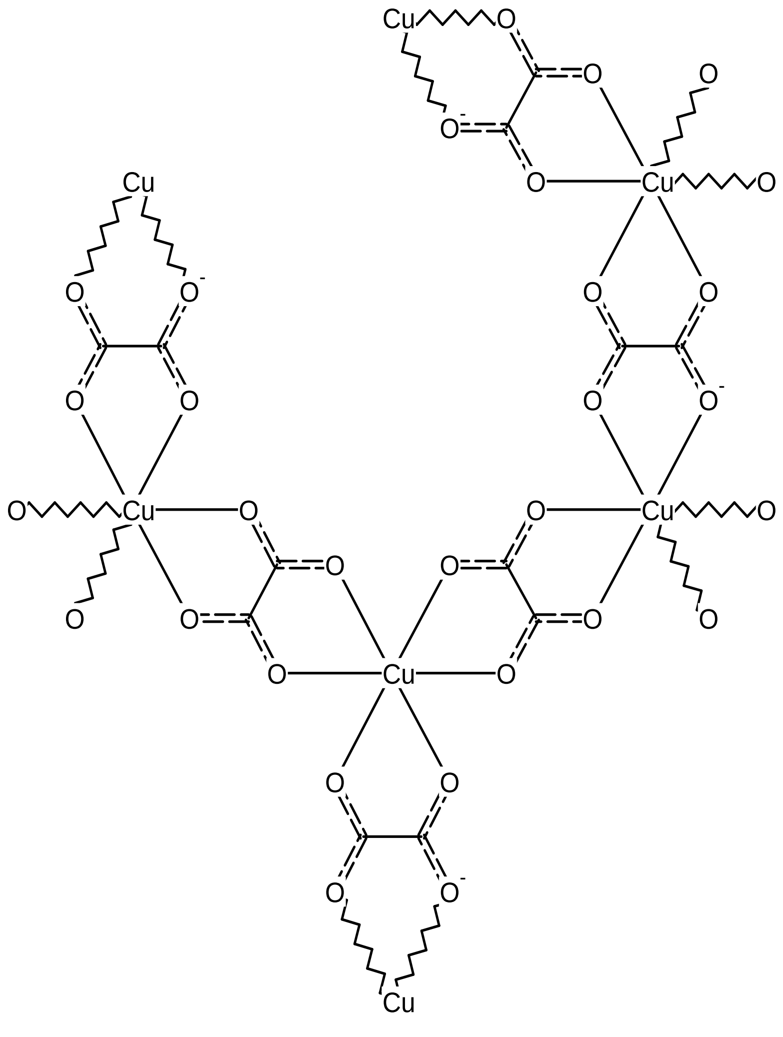

Parameters

Fragment 1

**DIST1 (D)**	2.011
**DIST2 (D)**	2.007
**DIST3 (D)**	1.958
**DIST4 (D)**	1.989
**DIST5 (D)**	2.240
**DIST6 (D)**	2.293

Fragment 2

**DIST1 (D)**	1.976
**DIST2 (D)**	2.020
**DIST3 (D)**	1.958
**DIST4 (D)**	2.013
**DIST5 (D)**	2.209
**DIST6 (D)**	2.304

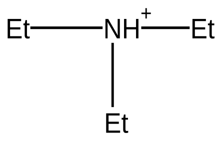

Search: search2 (Wed Jul 26 09:11:25 2017): Hit 232

LAVFEW

Reference: M.Puchonova, M.Mazur, Jan Moncol, Z.Ruzickova, D.Valigura (2017) *J.Mol.Struct.* ,**1137,**706

Formula: $(C_{14} H_{10} Br_4 Cu_1 O_8)n$

Compound Name: catena-(bis(μ-3,5-dibromo-2-hydroxybenzoato)-diaqua-copper(ii))

Space Group:	C2/c	***Cell:***	***a*** 32.419(7)	***b***	4.516(0)	***c***	13.126(3)
Space Group No.:	15	***(Å,°)***	α 90.00	β	106.75(3)	γ	90.00

R-Factor (%): 2.79 ***Temperature(K):*** 150 ***Density(g/cm^3):*** 2.488

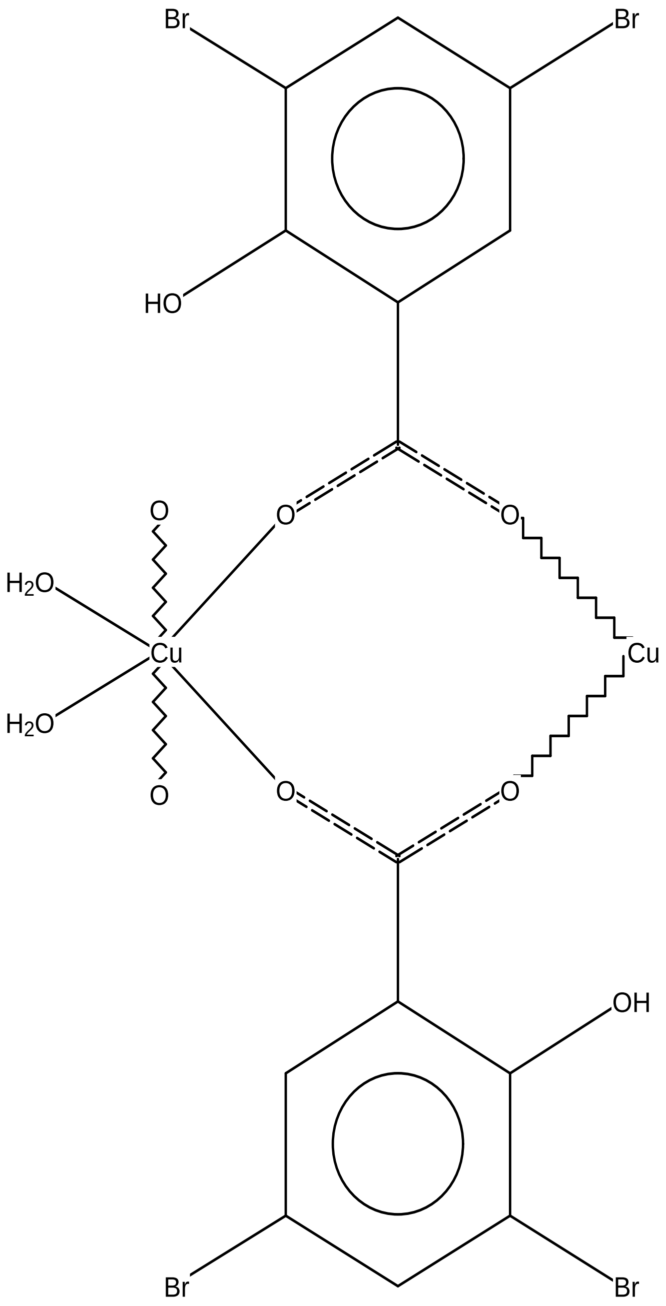

Parameters

Fragment 1

**DIST1 (D)**	1.937
**DIST2 (D)**	1.937
**DIST3 (D)**	1.942
**DIST4 (D)**	1.942
**DIST5 (D)**	2.637
**DIST6 (D)**	2.637
