# Supplementary material for: Electronic structure of two isostructural ‘paddle-wheel’ complexes: a comparative study
Source: Acta Crystallogr B Struct Sci Cryst Eng Mater. 2018 Nov 19;74(Pt 6):681–92. doi: 10.1107/S2052520618013707 (PMC6289242; doi:10.1107/S2052520618013707)
Supplement: Supplementary file 4 [file b-74-00681-sup4.pdf]

STRUCTURAL SCIENCE
CRYSTAL ENGINEERING
MATERIALS

Volume 74 (2018)

Supporting information for article:

Electronic structure of two isostructural `paddle-wheel' complexes: a comparative study

Peter Herich, Lukáš Bučinský, Martin Breza, Marián Gall, Marek Fronc, Václav Petřiček and Jozef Kožíšek

Appendix I.

Table SA1. Comparison of multipole refinement for **(I)** using non-averaged and merged data.

	Non-merged (EXTI aniso)	merged (NO EXTI)	merged (EXTI isotropic)
R(F), R(F^2)	0.0186	0.0118; 0.0281	0.0109; 0.0195
Residual density	+0.747/-0.357	+0.383/-0.750	+0.747/-0.357
No. of diffractions	397256	12311	12311
**Charges**			
Cu [-e]	1.49	2.03	1.59
O1 [-e]	-0.96	-0.98	-0.98
O2 [-e]	-0.99	-1.00	-1.02
O3 [-e]	-1.02	-0.97	-1.02
O4 [-e]	-1.10	-1.02	-1.05
O5 [-e]	-1.23	-1.13	-1.22
**Orbital**			
Z2 [e; %]	2.015(4); 21.5 %	1.91; 21.7 %	2.01; 21.7 %
XZ [e; %]	1.909(4); 20.4 %	1.78; 20.2 %	1.87; 20.2 %
YZ [e; %]	2.086(4) ; 22.3 %	1.93; 21.9 %	2.03; 22.0 %
X2-Y2 [e; %]	1.363(4) ; 14.6 %	1.28; 14.6 %	1.37; 14.8 %
XY [e; %]	1.985(4) ; 22.1 %	1.90; 21.5 %	1.98; 21.4 %
Σ [e]	9.36	8.81	9.26
**AIM at BCP#**			
Cu-O1	0.468(1); 12.462(1)	0.438(3); 12.091(5)	0.468(3);12.299(5)
Cu-O2	0.448(1); 12.800(2)	0.445(3); 12.270(5)	0.475(3); 12.473(5)
Cu-O3	0.435(1); 11.363(1)	0.414(3); 10.949(5)	0.441(3); 11.139(5)
Cu-O4	0.410(1); 10.878(1)	0.339(3); 10.611(5)	0.425(3); 10.760(5)
Cu-O5	0.295(0); 7.026(1)	0.295(3); 6.930(3)	0.295(3); 6.930(3)
Cu-Cu*	0.057(0); 1.468(0)	0.052(0); 1.515(0)	0.060(0); 1.683(0)

ρ_{BCP} [e/Å^3]; $\nabla^2\rho_{BCP}$ [e/Å^5]

Figures SA2. Residual densities in the plane defined by the atoms: *atom1 – atom2 – atom3*.

plane defined		Non-merged (EXTI aniso)		merged (NO EXTI)		merged (EXTI isotropic)
Cu-O1-O3		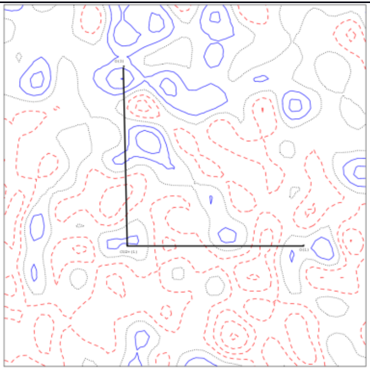		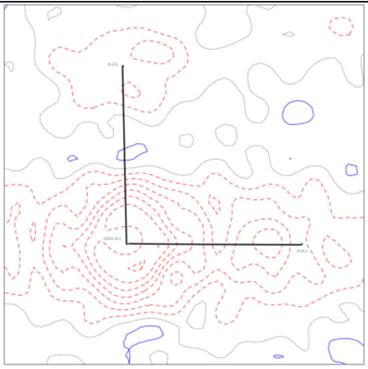		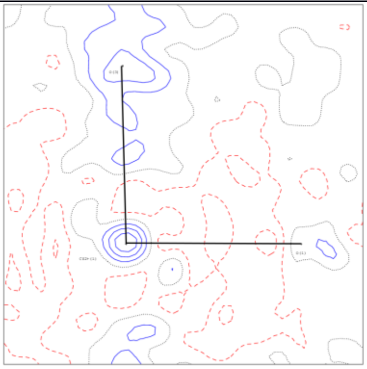
Cu-O2-O4		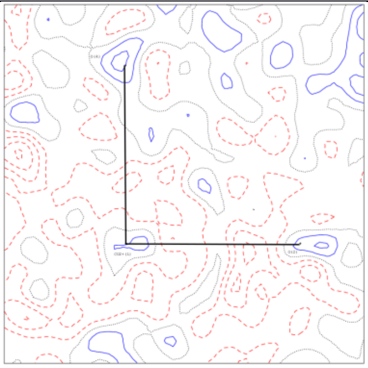		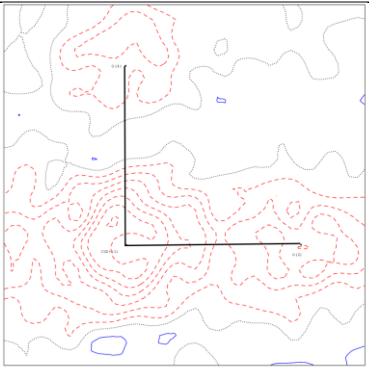		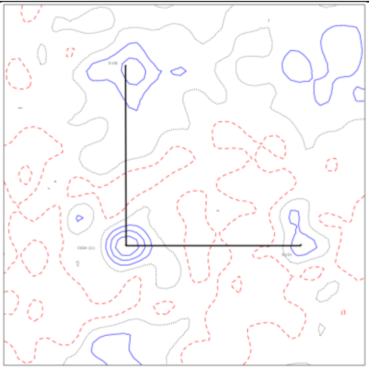

Cu-O1-O5		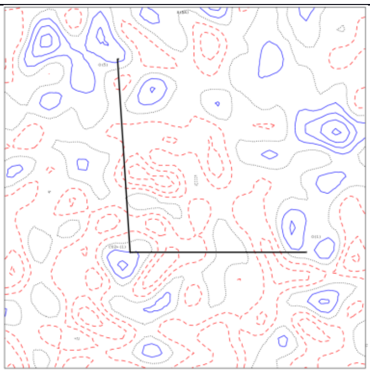		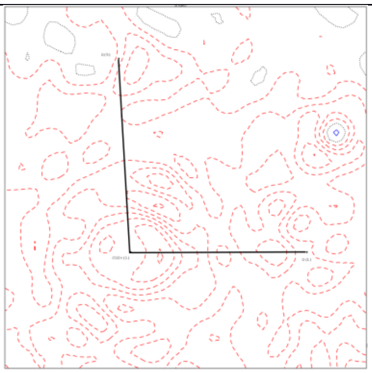		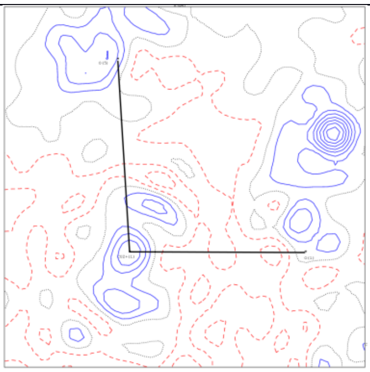

Figures SA3. Error analysis for **(I)**.

		Non-merged (EXTI aniso)		merged (NO EXTI)		merged (EXTI isotropic)
Normal probability distribution plot		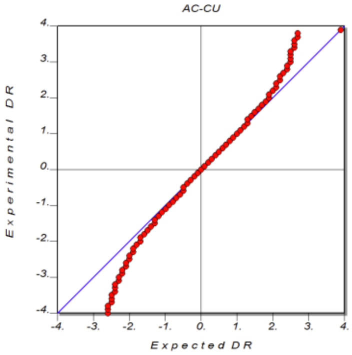		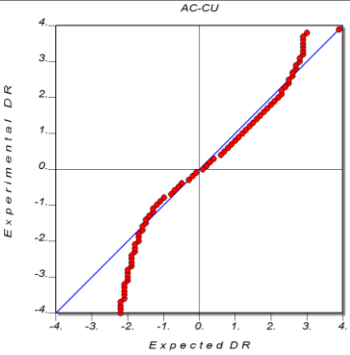		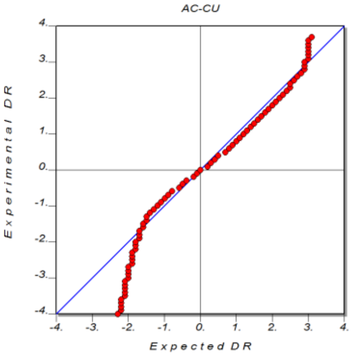
c		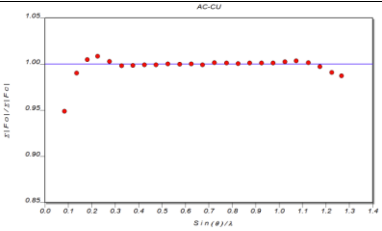		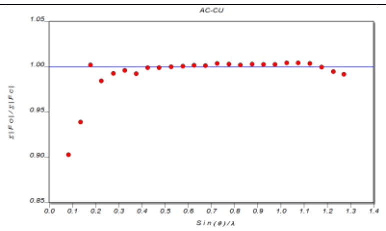		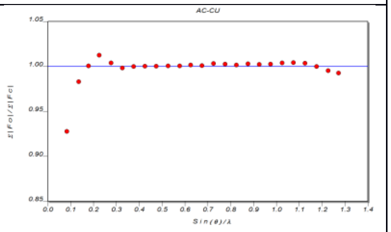

Fractal analysis of the residual density		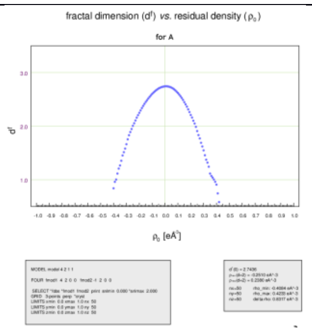		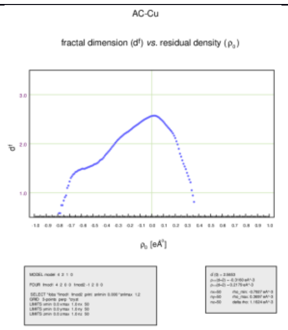		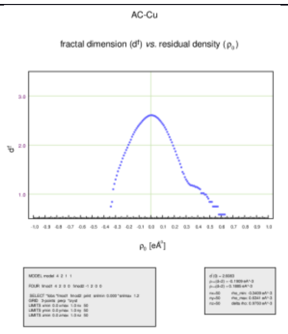
$F_{obs} - F_{calc}$ *vs.* F_{calc}		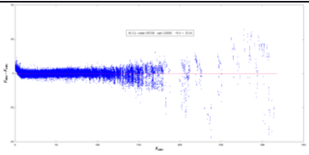		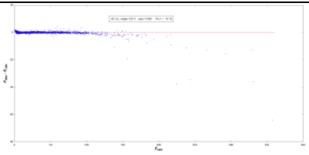		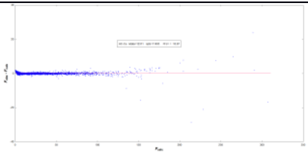
$F_{obs} - F_{calc}$ *vs.* $\sin(\theta)/\lambda$		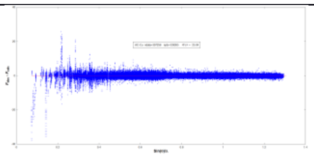		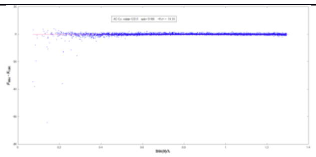		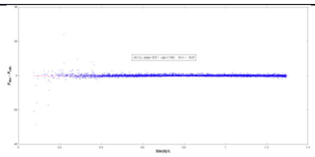

Figures SA4 (see on the next pages). Static electron deformation densities of **(I)** in the plane defined by the atoms O(2)*,O(1), O(3). Contour spacing as in Fig. 2b. Symmetry code: * 1-x, 1-y, 1-z.

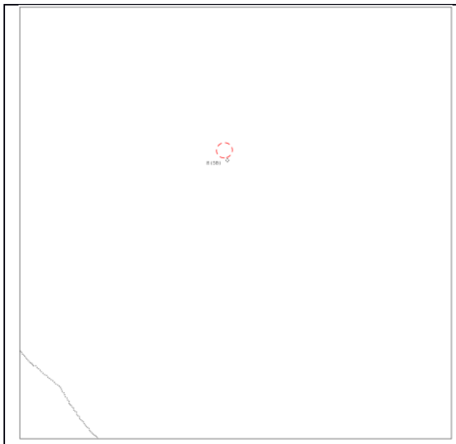	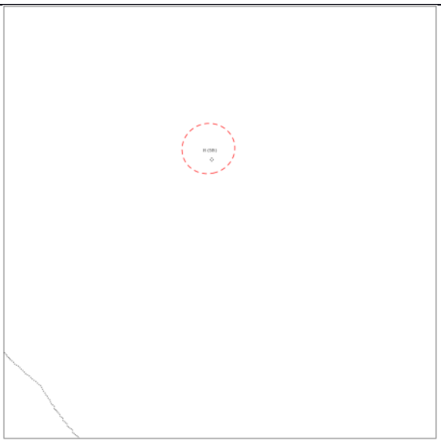	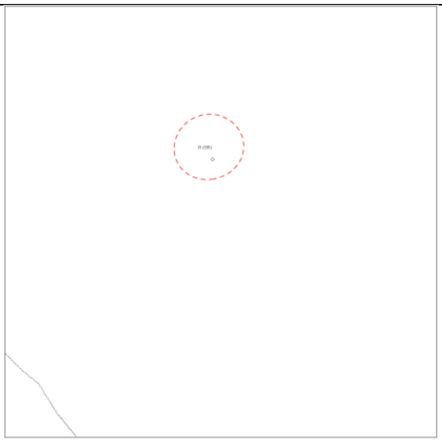
3.984 Å above the plane	3.884 Å above the plane	3.784 Å above the plane
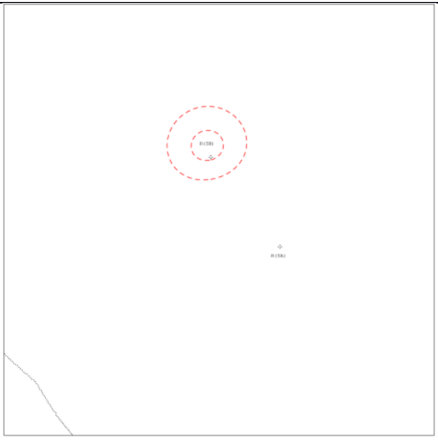	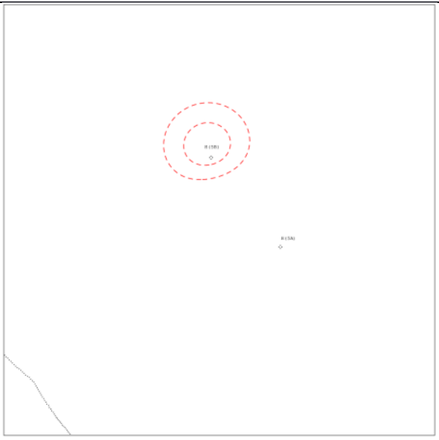	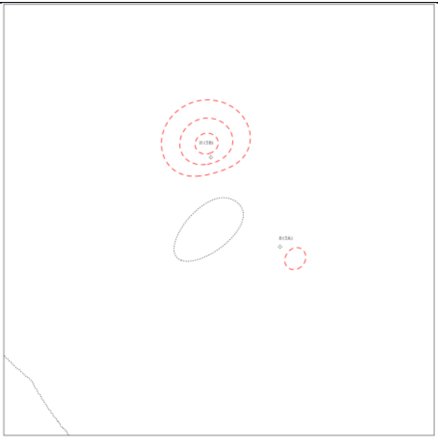
3.684 Å above the plane	3.584 Å above the plane	3.484 Å above the plane
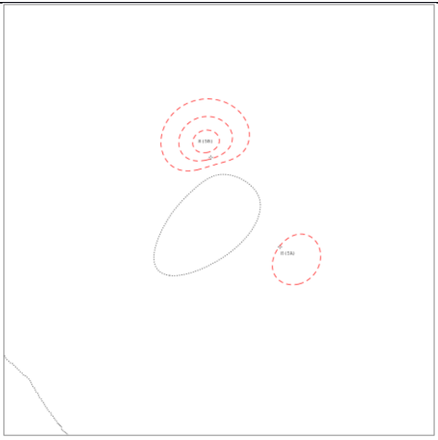	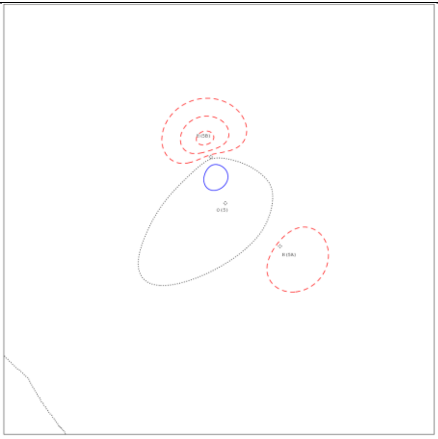	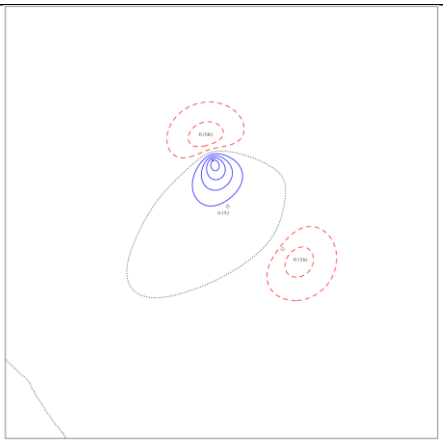
3.384 Å above the plane	3.284 Å above the plane	3.184 Å above the plane

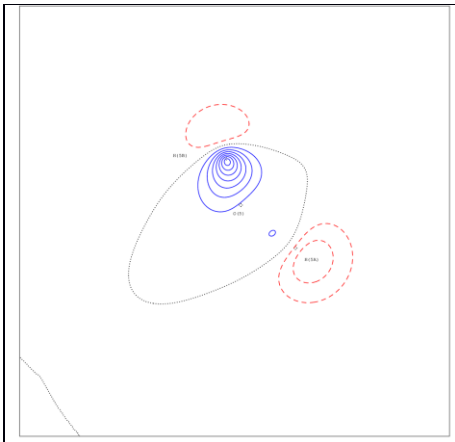	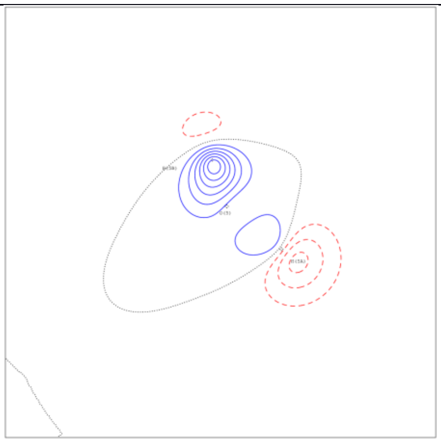	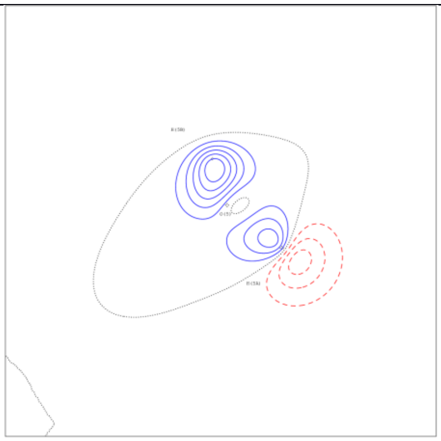
3.084 Å above the plane	2.984 Å above the plane	2.884 Å above the plane
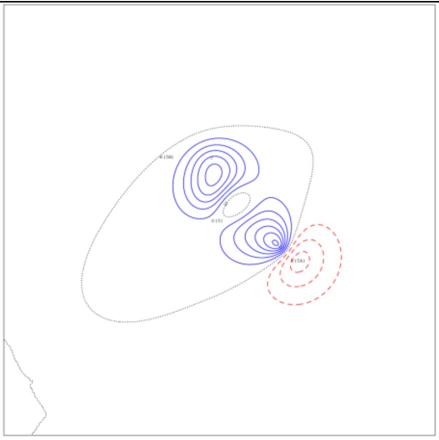	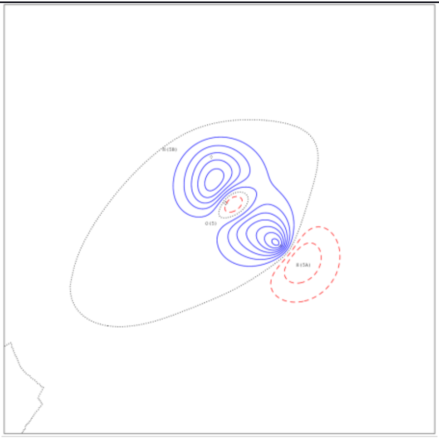	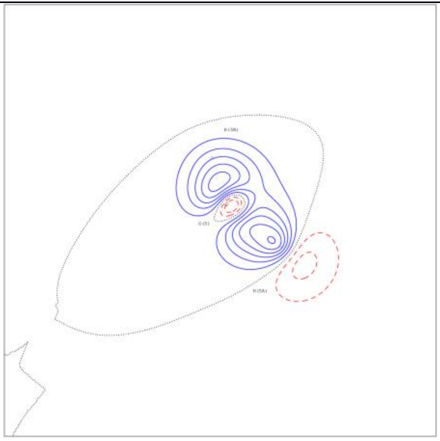
2.784 Å above the plane	2.684 Å above the plane	2.584 Å above the plane
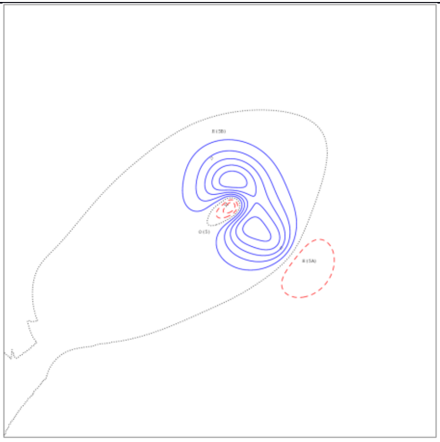	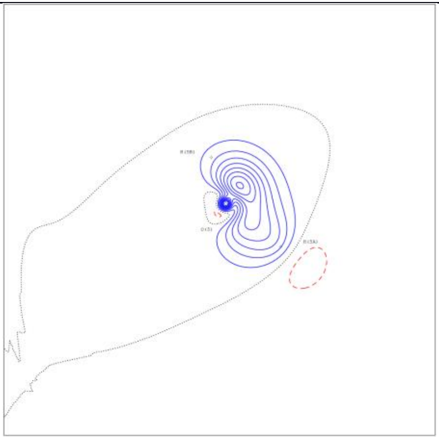	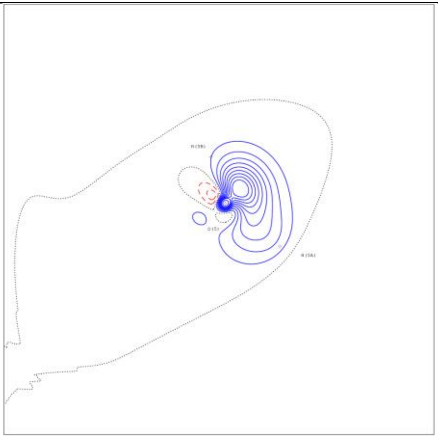
2.484 Å above the plane	2.384 Å above the plane	2.284 Å above the plane

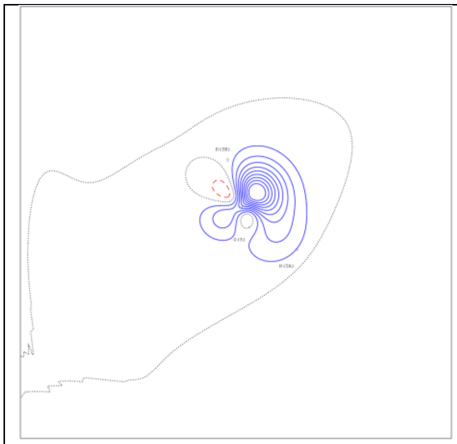	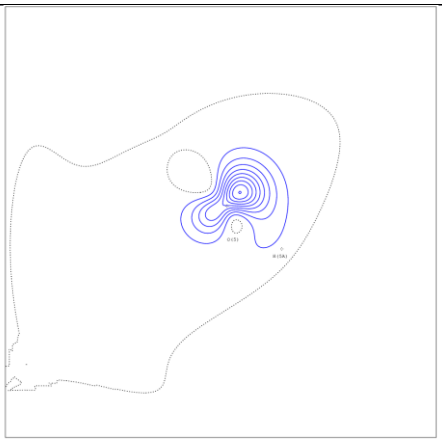	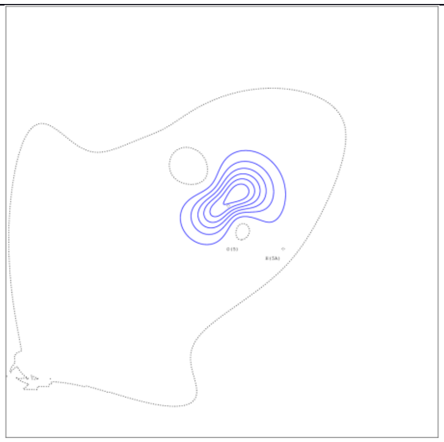
2.184 Å above the plane	2.084 Å above the plane	1.984 Å above the plane
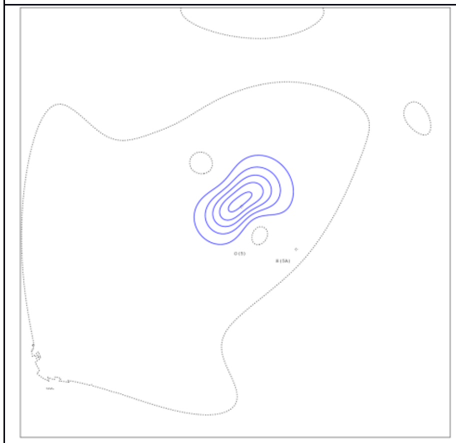	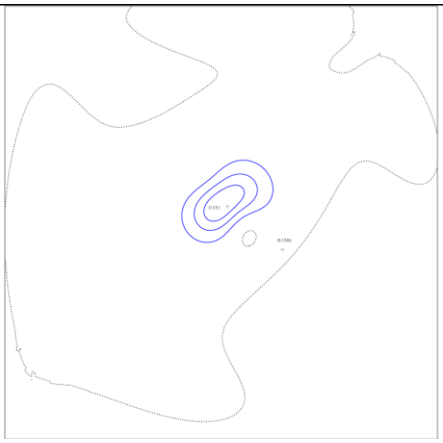	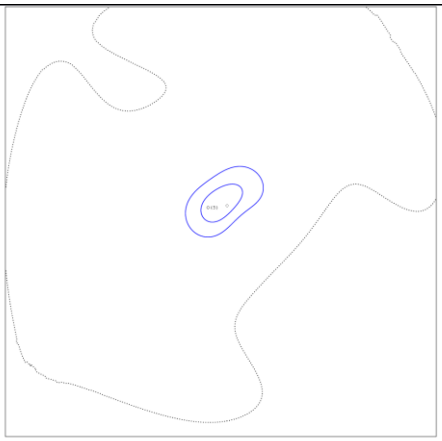
1.884 Å above the plane	1.784 Å above the plane	1.684 Å above the plane
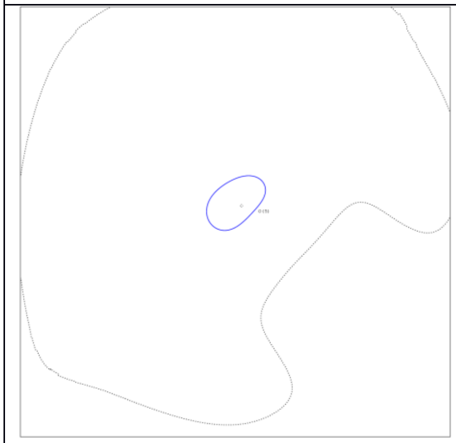	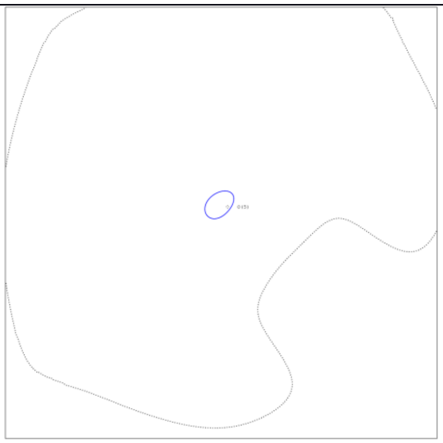	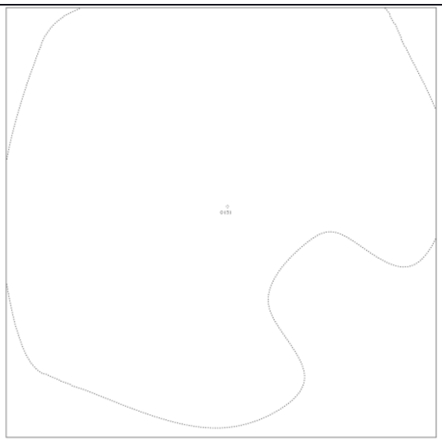
1.584 Å above the plane	1.484 Å above the plane	1.384 Å above the plane

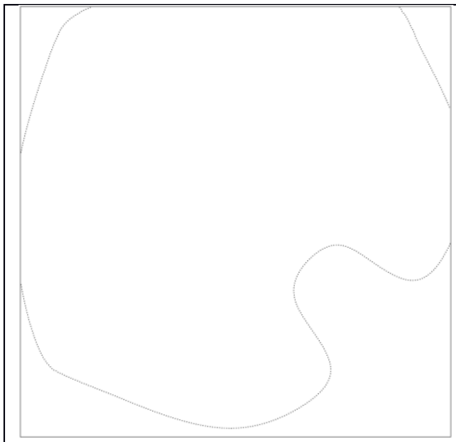	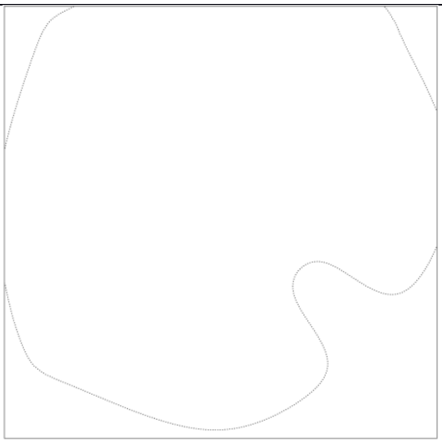	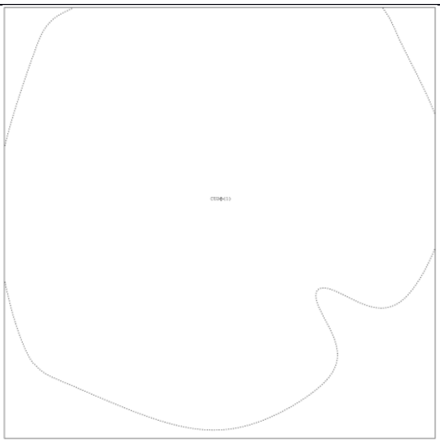
1.284 Å above the plane	1.184 Å above the plane	1.084 Å above the plane
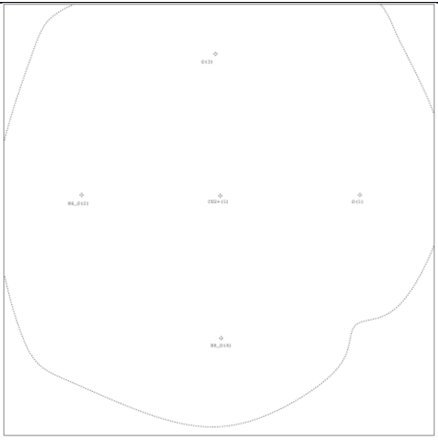	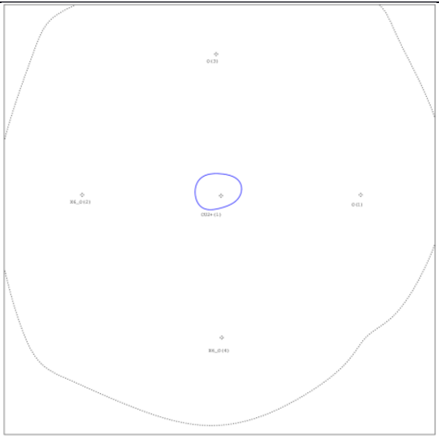	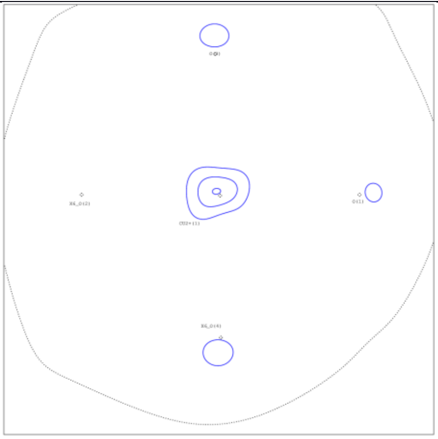
0.984 Å above the plane	0.884 Å above the plane	0.784 Å above the plane
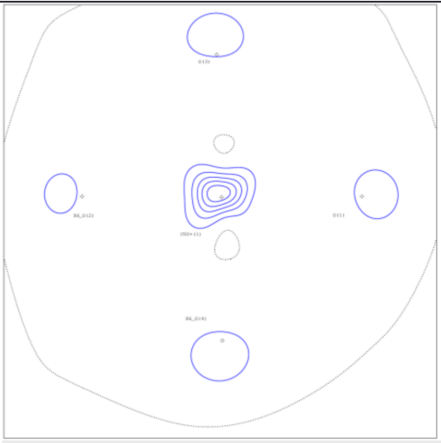	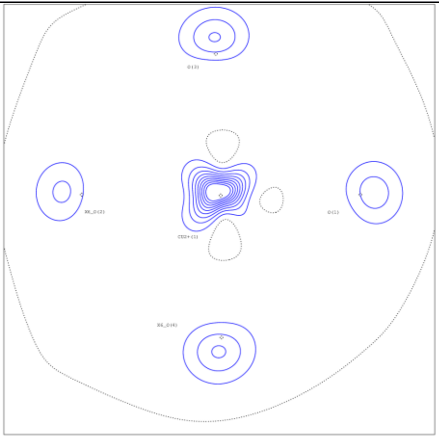	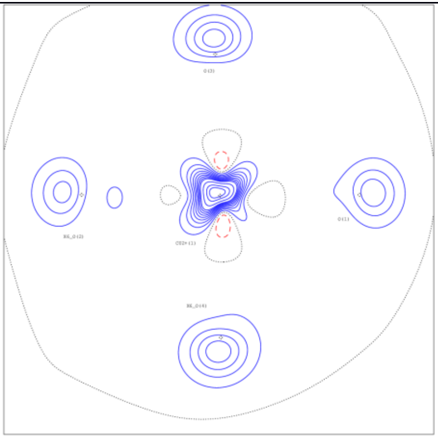
0.684 Å above the plane	0.584 Å above the plane	0.484 Å above the plane

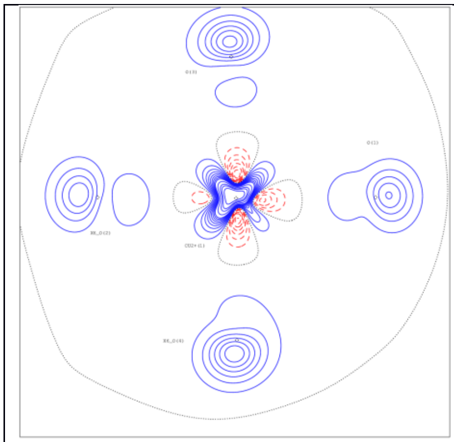	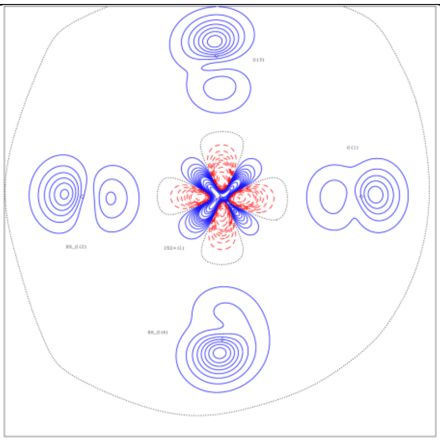	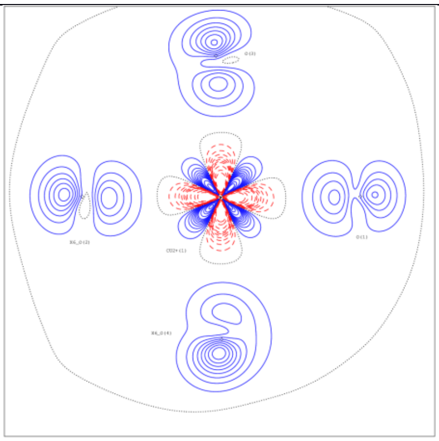
0.384 Å above the plane	0.284 Å above the plane	0.184 Å above the plane
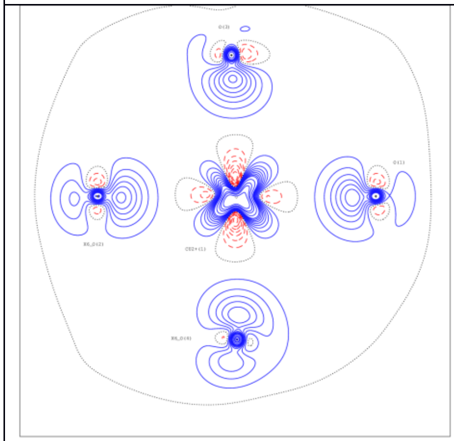	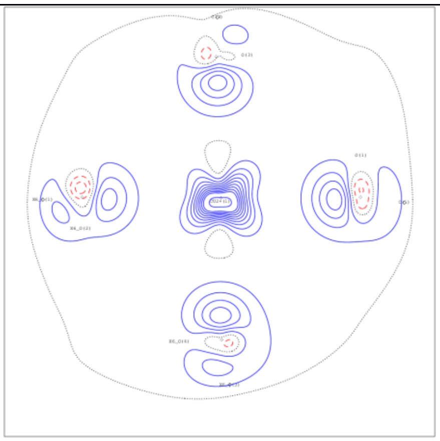	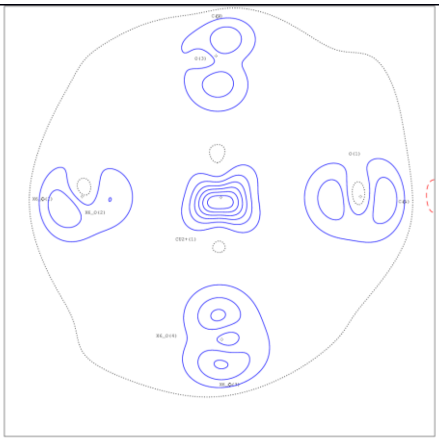
In the plane O(2)*,O(1), O(3)	0.184 Å below the plane	0.284 Å below the plane
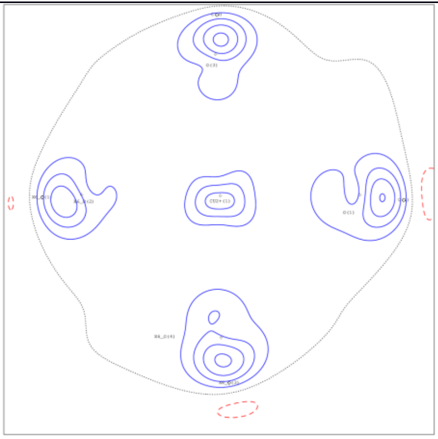	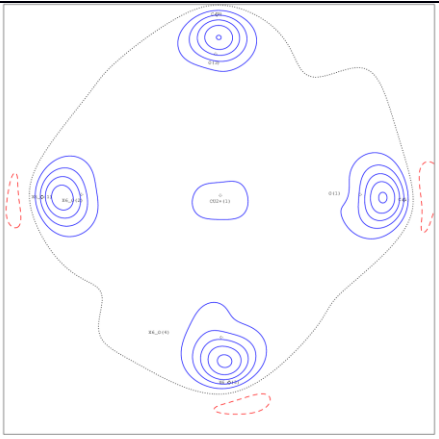	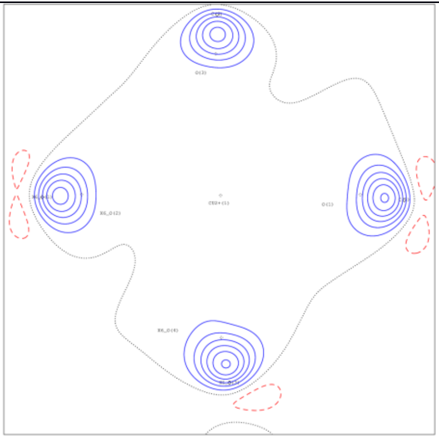
0.384 Å below the plane	0.484 Å below the plane	0.584 Å below the plane

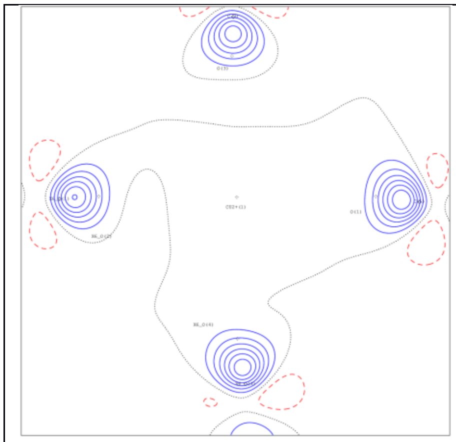	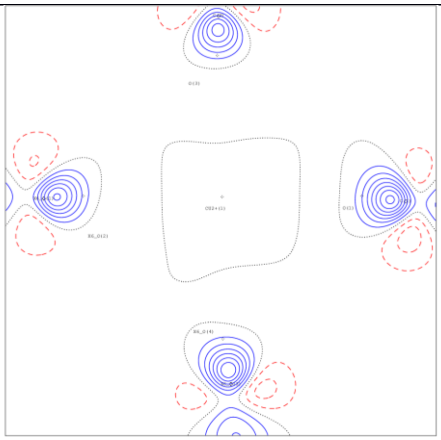	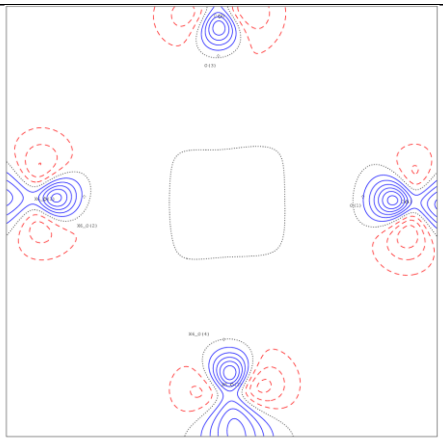
0.684 Å below the plane	0.784 Å below the plane	0.884 Å below the plane
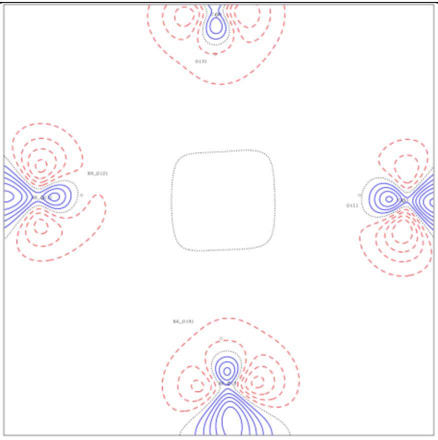	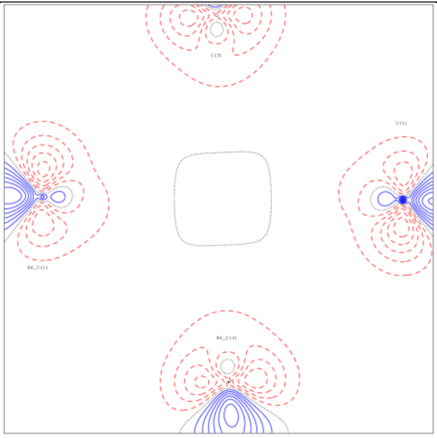	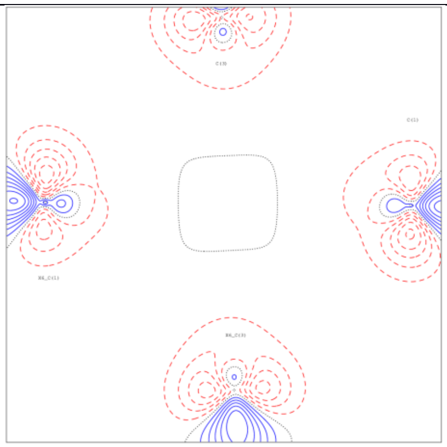
0.984 Å below the plane	1.084 Å below the plane	1.184 Å below the plane
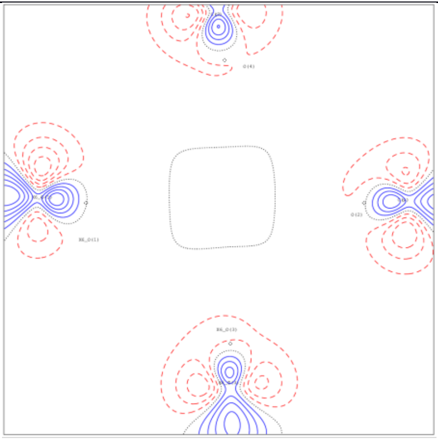	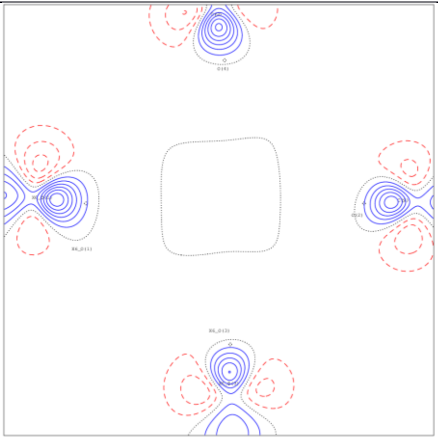	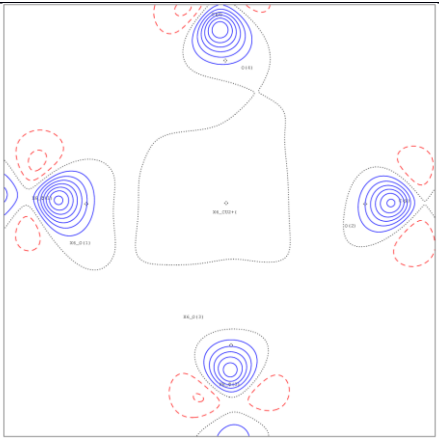
1.284 Å below the plane	1.384 Å below the plane	1.484 Å below the plane

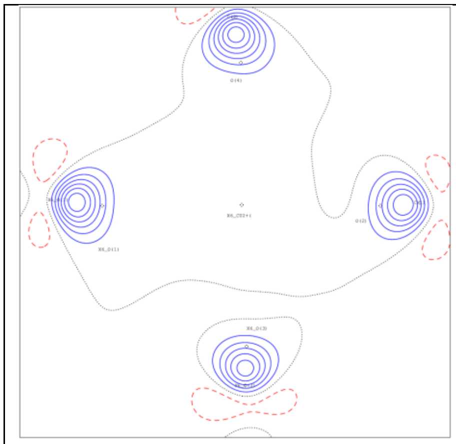	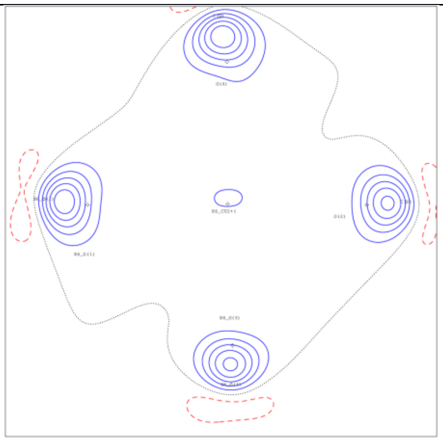	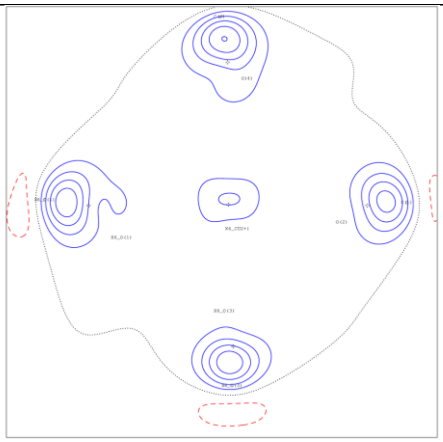
1.584 Å below the plane	1.684 Å below the plane	1.784 Å below the plane
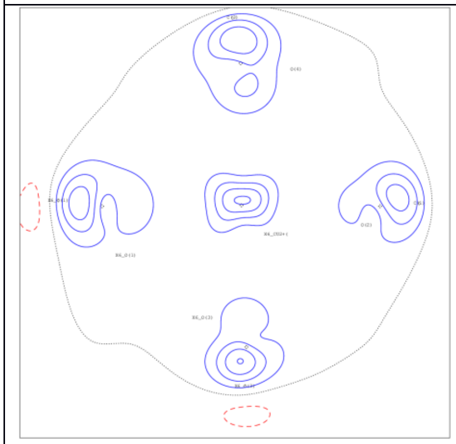	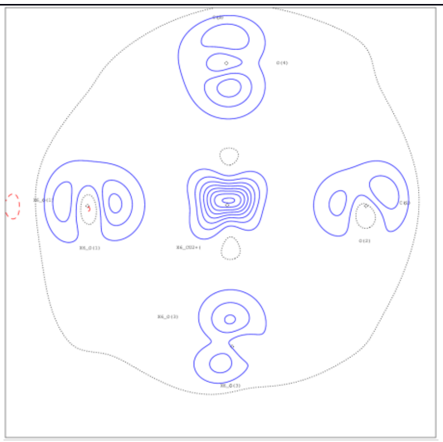	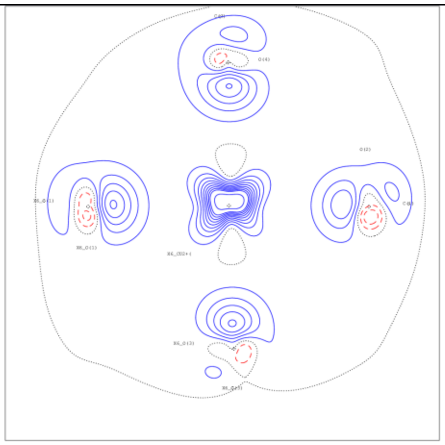
1.884 Å below the plane	1.984 Å below the plane	2.084 Å below the plane
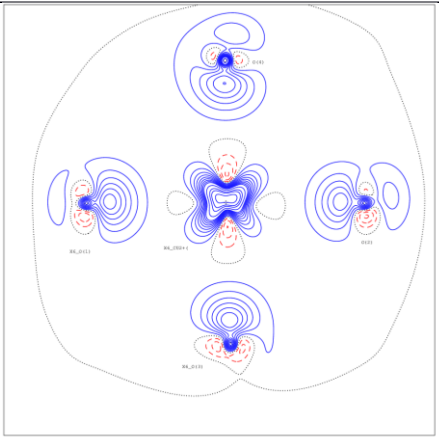	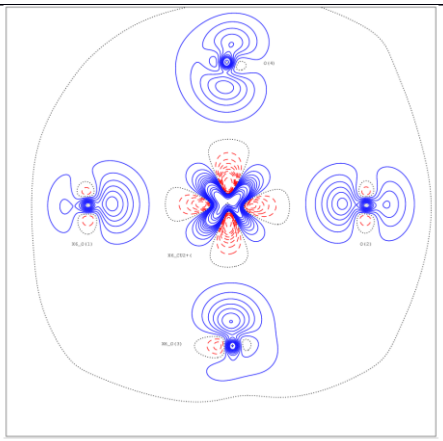	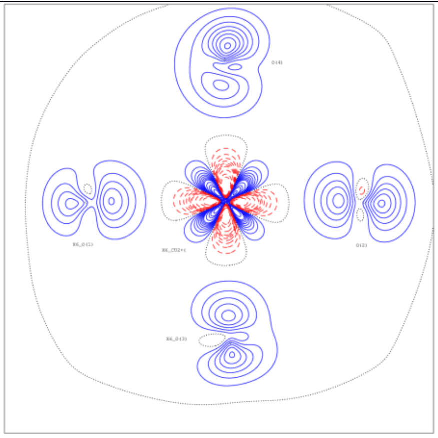
2.184 Å below the plane	2.284 Å below the plane	2.384 Å below the plane

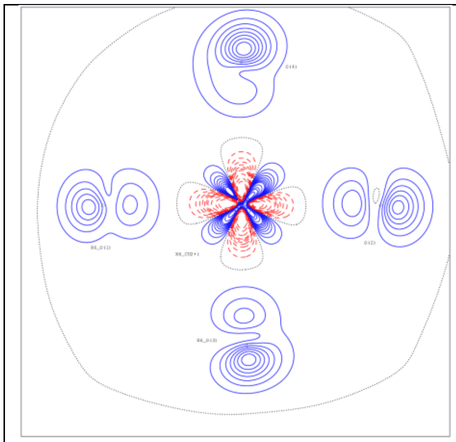	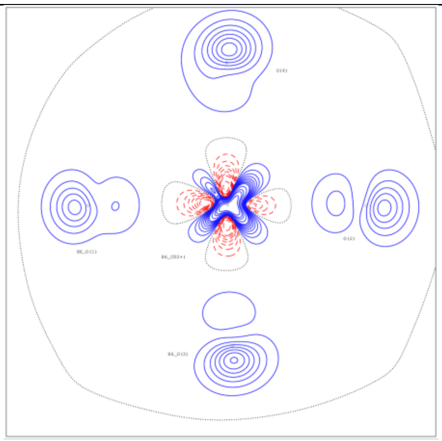	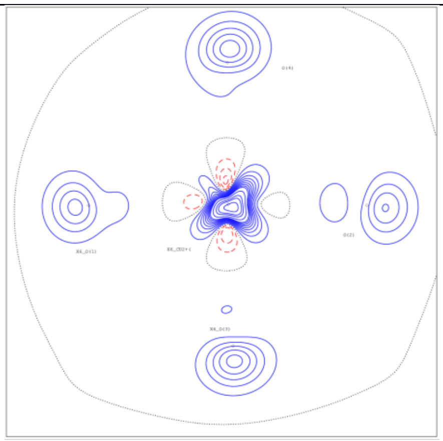
2.484 Å below the plane	2.584 Å below the plane	2.684 Å below the plane
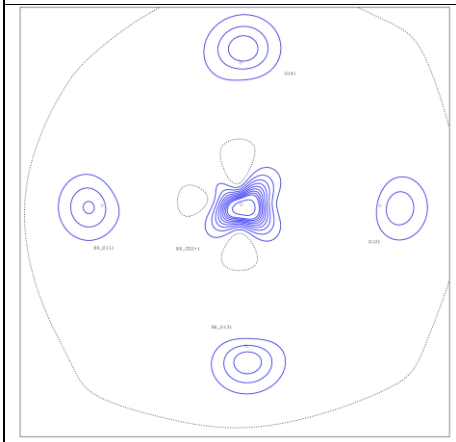	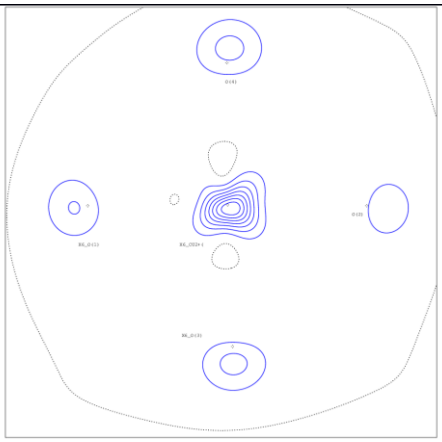	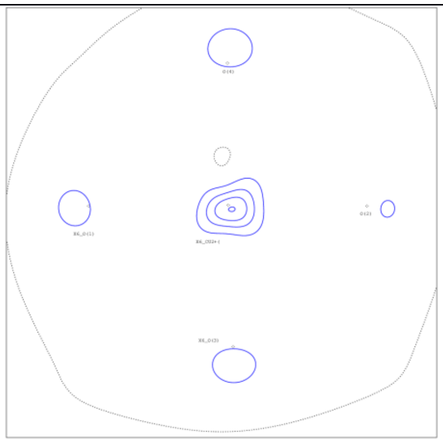
2.784 Å below the plane	2.884 Å below the plane	2.984 Å below the plane
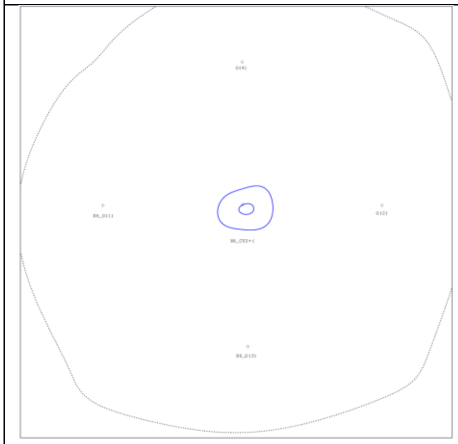	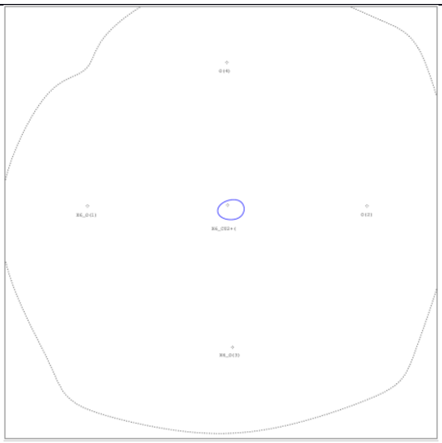	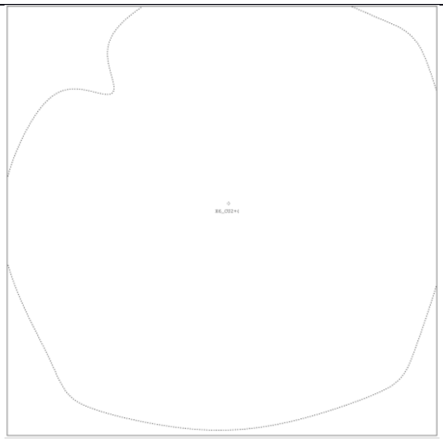
3.084 Å below the plane	3.184 Å below the plane	3.284 Å below the plane
